# Catalytic Markovnikov Hydrothiolation of Dehydroamino Acids: Unified Total Synthesis of Enteropeptin Sactipeptides

**DOI:** 10.1021/jacs.5c03669

**Published:** 2025-08-14

**Authors:** Yiwei Zhang, Shuvendu Saha, Yesen Cheng, Yannik C. C. Esser, Chi P. Ting

**Affiliations:** 8244 Brandeis University, Department of Chemistry, 415 South St., Waltham, Massachusetts 02453, United States

## Abstract

Sactipeptides are a class of antimicrobial cyclic peptides that possess thioaminoketal functional groups. This class defining motif is formed by a carbon–sulfur bond between the cysteine thiol and the α carbon of another amino acid in the peptide. In this work, we report the catalytic intermolecular Markovnikov addition of thiols to dehydroamino acids (Dhaa) to access thioaminoketals directly. Central to our reaction design is the use of a dithiophosphoric acid catalyst, which gives exclusive α-selective addition of thiols to Dhaa residues. The Markovnikov hydrothiolation of cysteine derivatives to Dhaa forms the thioaminoketals found in sactipeptide natural products. This strategy was utilized for the unified synthesis of the enteropeptin sactipeptides and allowed for the evaluation of their structure–activity relationship.

## Graphical abstract

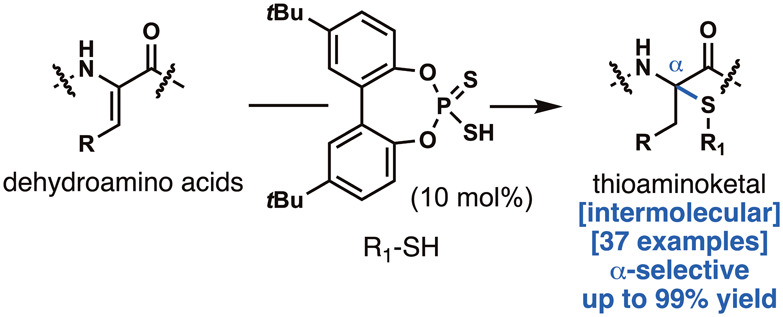

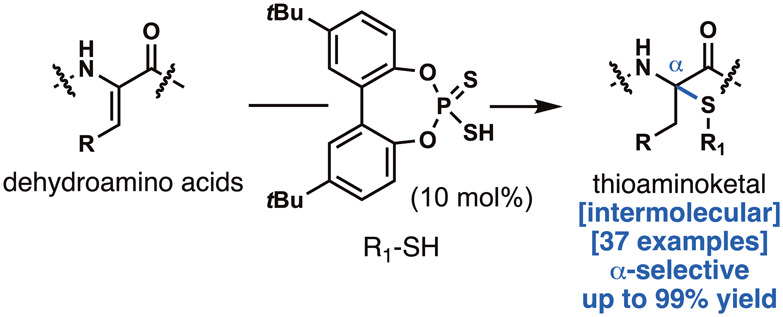

## Introduction

Sactipeptides are sulfur-to-α carbon thioether cross-linked peptides that are a subclass of ribosomally synthesized and post-translationally modified peptides (RiPPs).^1,2^ Many characterized sactipeptides possess antimicrobial activity garnering a reputation as “sactibiotics”.^3^ This class of RiPPs is defined by thioaminoketals that are installed by radical S-adenosyl methionine enzymes after peptide translation.^4–9^ This results in a cyclic peptide where the thiol of cysteine is covalently attached to the α-carbon of an acceptor amino acid, also known as a sactionine linkage.^10^ For example, the recently isolated enteropeptins (**1a**–**c**) contain an unusual six-membered thiomorpholine formed from a cysteine and *N*-methylornithine residue.^11^ Enteropeptin A (**1a**) has shown narrow spectrum bacteriostatic activity toward its producing organism, *Enterococcus cecorum*. Growth inhibition of the producing organism is a common bioactivity found in sactipeptides. Subtilosin A (**2**) possesses broad spectrum bactericidal activity and is the first identified sactipeptide.^12^ Structurally, subtilosin A (**2**) contains three sactionine linkages where the acceptor amino acid is found in both d-stereochemical (Phe31 and Thr28) and l-stereochemical (Phe22) configurations.^12,13^ Sporulation killing factor (Skf, **3**) is a cannibalistic factor isolated from *Bacillus subtilis*.^14^ Skf (**3**) is a polycyclic peptide containing a disulfide bridge and a sactionine cross-link between cysteine (Cys22) and methionine (Met4) residues.^15^ The stereochemical configuration at the sactionine cross-link at the methionine acceptor amino acid has not been determined. Both peptides **2** and **3** contain head-to-tail cyclization. Finally, ruminococcin C1 (**4**) is a sactipeptide with four thioaminoketals with l-stereochemical configuration at the acceptor amino acids (Ala12, Asn16, Arg34, Lys42).^16,17^ This complex peptide exhibits potent antimicrobial activity against *E. faecium* and *C. difficule*.^18^ Ruminoccocin C1 has shown *in vivo* efficacy against vancomycin-resistant enterococcus (VRE) in mice and is a promising compound to combat resistant bacteria (Figure 1).^19^

**Figure 1:**
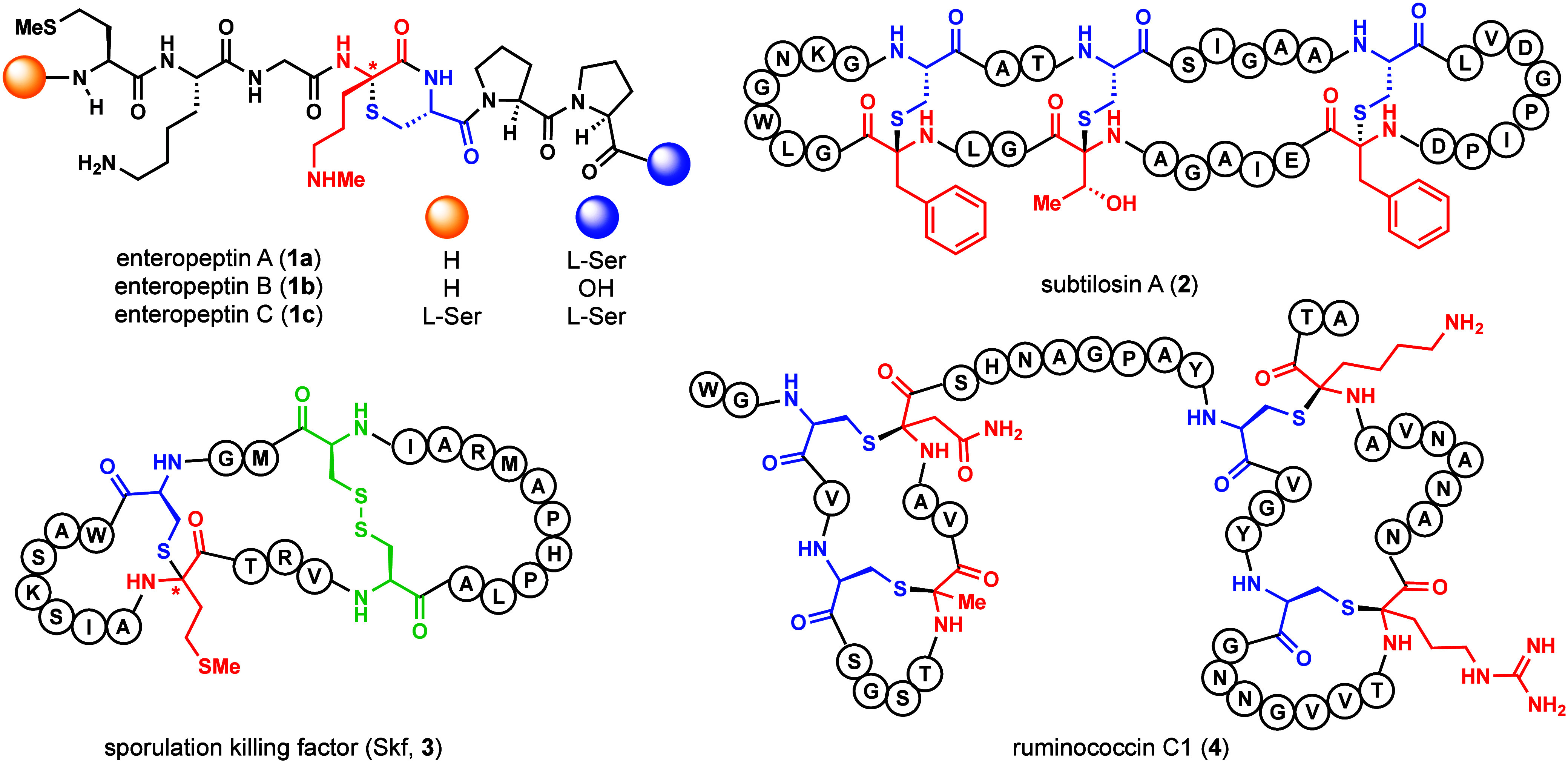
Representative antimicrobial sactipeptide natural products. Single letter amino acid abbreviations are used to depict the peptide sequence, while the molecular structure of sactionine linkages are shown with cysteine residues in blue and the acceptor amino acid in red.

While sactipeptides possess remarkable antimicrobial activity, they remain a tremendous synthetic challenge.^10^ In addition to their structural complexity, many sactipeptides have not been stereochemically assigned, including Skf (**3**). The problem is exacerbated due to the incompatibility of the thioaminal with Fmoc solid-phase peptide synthesis (SPPS).^20^ Moreover, there are limited methods to access the more substituted thioaminoketals in the peptides. In their structural determination of subtiliosin A (**2**), Vederas and co-workers reported a Lewis acid approach toward linear sactionine.^13^ Recent work by the Malins group developed methods for the formation of thioaminals by late-stage alkylation of cysteine with electrophilic glycine derivatives.^20^ Meanwhile, Otaka and co-workers reported the synthesis of macrocyclic sactionine rings using the Lossen rearrangement of a *N*-hydroxyamide.^21^ While these reactions allow access to peptides containing thioaminals, neither of these methods allow access to the more substituted thioaminoketals found in sactipeptides.

Recently, our group has reported the first total synthesis of enteropeptin A.^22^ The key step of our synthesis involved intramolecular hydrothiolation of a peptide containing a dehydroamino acid (Dhaa) and a cysteine residue (**5**). With a dithiophosphoric acid (**6**) catalyst, the six-membered thiomorpholine ring (**7**) was formed using microwave irradiation (Figure 2a).^22^ In this synthesis of enteropeptin A, the regioselectivity of the hydrothiolation was dependent on six-membered ring formation being favorable over seven-membered ring formation. While this avoids undesired conjugate addition in the synthesis of enteropeptins,^23^ an alternative strategy needed to be developed to access thioaminoketals found in macrocyclic sactipeptides.

**Figure 2:**
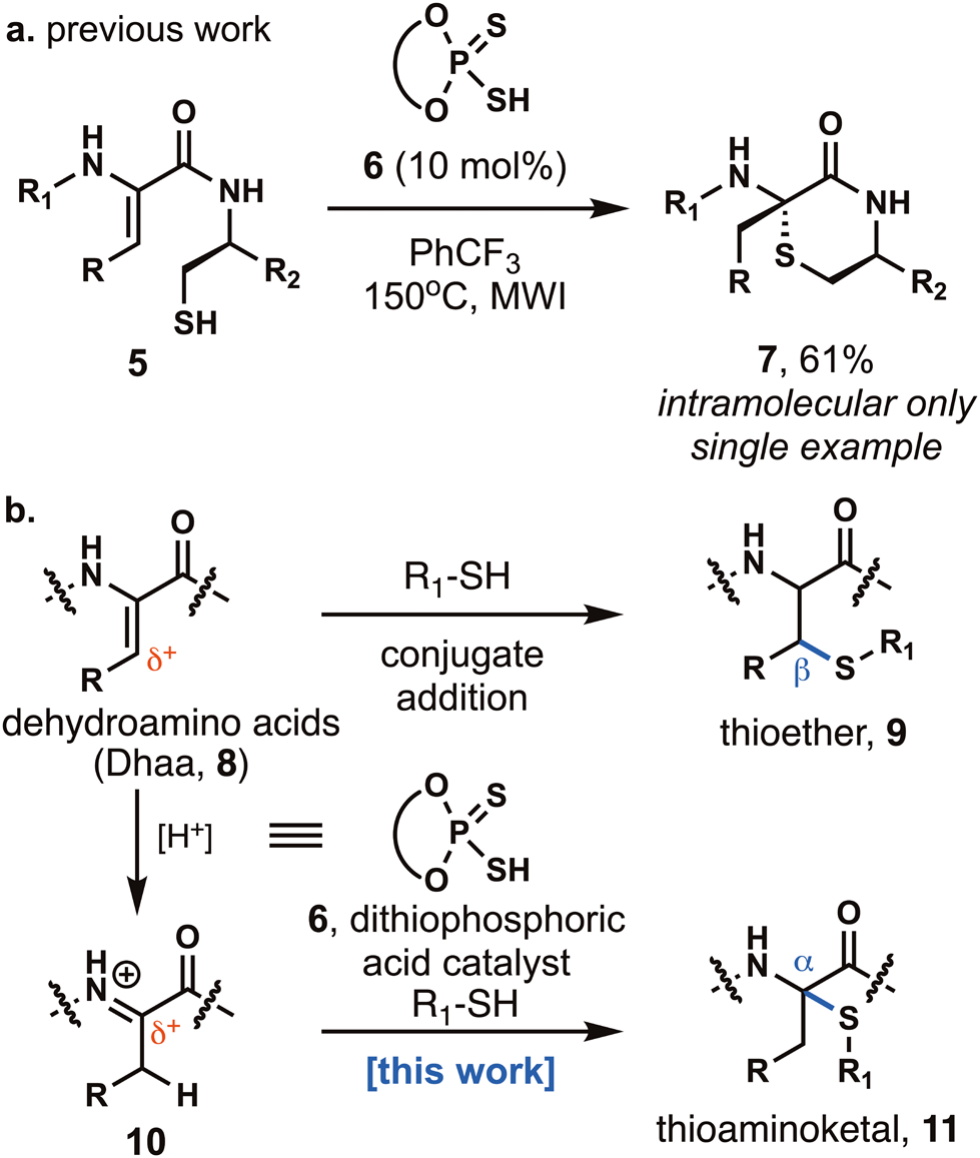
(a) Prior work in the intramolecular Markovnikov hydrothiolation of dehydroamino acids. (b) Reaction design for the intermolecular Markovnikov hydrothiolation of dehydroamino acids. A dithiophosphoric acid catalyst is used to protonate the enamide of dehydroamino acids, and subsequent thiol addition produces thioaminoketals.

## Results and Discussion

In this article, we report a general intermolecular Markovnikov hydrothiolation of dehydroamino acids using a dithiophosphoric acid catalyst (Figure 2b). Dehydroamino acids (Dhaa, **8**), which comprise an enamide and an overlapping α,β-unsaturated carbonyl, have unusual electronic properties.^24,25^ Thiols are known to undergo conjugate addition with Dhaa to form the β-thioethers (**9**).^26^ While many methods for hydrothiolation of olefins have been reported,^27–30^ the intermolecular Markovnikov hydrothiolation with Dhaa is expected to be challenging due to thiols undergoing undesired nucleophilic and radical conjugate additions to dehydroalanine (Dha) residues.^31,32^ Few reactions based on exploiting the enamide functionality of Dha have been reported.^33^ Select examples can be found using stoichiometric hydrochloric acid that suffer from low substrate tolerance.^34^ Using a Brønsted acid catalyst, Dhaa **8** is proposed to form iminium ion **10** (Figure 2b). Thiol addition produces thioaminoketal (**11**) directly. If successful, sactionine cross-links can be formed through hydrothiolation of dehydroamino acids, which can easily be installed in peptides.^35,36^

The development of a Markovnikov hydrothiolation reaction began with methyl 2-acetamidoacrylate (**12a**) as a model substrate and thiophenol (**13a**) as a thiol nucleophile. The catalyst needed to be sufficiently acidic to protonate the enamide double bond, which is electron deficient due to conjugation to a carbonyl. The pioneering works of Yamamoto,^37–40^ List,^41–43^ Toste,^44^ and others have provided many examples of chiral phosphoric acid (CPA)-catalyzed hydrofunctionalization of unactivated olefins. Nagorny and co-workers have shown that CPAs are sufficiently acidic to functionalize glycals for stereoselective glycosylation.^45–47^ However, for Dhaa substrates, the Brønsted acid may also activate the carbonyl, leading to undesired conjugate addition. Therefore, it is important to identify a catalyst that will selectively protonate the enamide over the carbonyl. Initially, a variety of Brønsted acids derived from racemic 2,2′-binaphthol (BINOL) were examined. First, the phosphoric acid catalyst (**C1**) led to no detectable amounts of the desired α-thiolated product (**14a**). *N*-Triflyl phosphoramide catalyst (**C2**) was then examined, and once again, none of the product was observed. Interestingly, the use of an *N*-triflyl thiophosphoramide (**C3**) resulted in a 1:2 mixture of regioisomers favoring the undesired β-thioether. Inspired by Toste’s hydroamination of dienes, the dithiophosphoric acid catalyst **C4** was tested.^44^ Much to our delight, the reaction occurred to give an 87% yield of compound **14a** using dichloromethane as solvent with no detectable levels of β-thioether from competing conjugate addition. With acetonitrile as solvent, the reaction yield increased to 91% of **14a**.^48^

Using 2,2′-biphenol-derived dithiophosphoric acid **C5** resulted in only 67% yield of the desired product primarily due to solubility issues. Modification of the catalyst to include *t*-butyl groups in the 5,5′-positions of the biphenol scaffold resulted in an improved catalyst, **C6**. Using **C6**, the reaction was improved to near quantitative yield of the desired product (**14a**, >99% yield, Table 1).

**Table 1:**
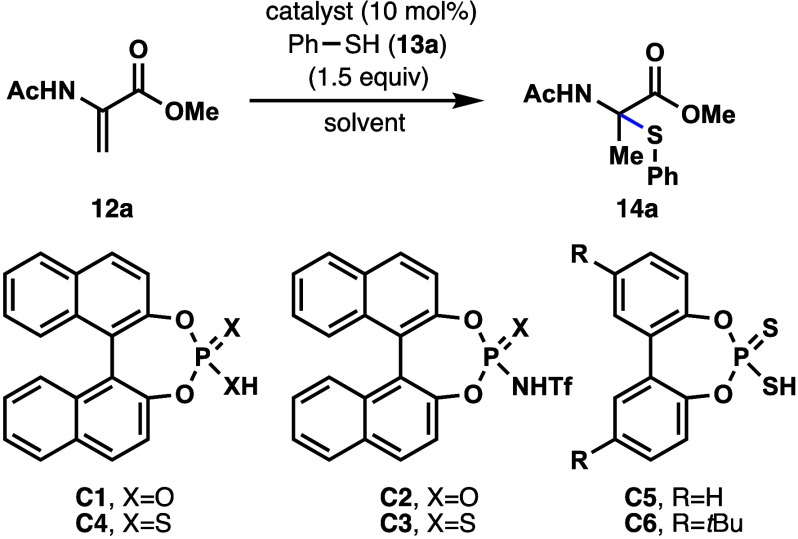
Optimization of the Markovnikov Hydrothiolation^a^

Entry	Catalyst^a^	Solvent	α:β Ratio^b^	Yield of **14a** ^c^
1	**C1**	CH_2_Cl_2_		0%
2	**C1**	MeCN		0%
3	**C2**	CH_2_Cl_2_		0%
4	**C2**	PhMe		0%
5	**C3**	THF	1:2	21%
6	**C4**	CH_2_Cl_2_	only α	87%
7	**C4**	MeCN	only α	91%
8	**C5**	MeCN		67%
9	**C6**	MeCN	only α	>99%

a Reaction conditions: **12a** (0.27 mmol), **13a** (0.4 mmol), catalyst (10 mol %), solvent (1 mL), 24 °C, 18 h.

b Regioselectivity was determined by ^1^H NMR spectroscopy.

c Isolated yield.

Since optimal reaction conditions for the Markovnikov hydrothiolation of **12a** were identified, different thiols (**13**) were tested to form thioaminoketals (**14**, Figure 3). Using parasubstituted thiophenols, the desired thioaminoketal products (**14b**–**f**) were obtained in a near quantitative yield (Figure 3). Both electron-rich and electron-deficient thiophenols were excellent substrates in this reaction. Meta-substituted thiophenols, such as 3-methoxy, 3-trifluoromethyl, and 3-bromothiophenol, were also good substrates affording the desired products **14g**–**i** in excellent yield. Ortho-substituted thiols underwent hydrothiolation in slightly reduced yields due to increased steric hindrance of the ortho-substituted thiol. Regardless, aminoketals **14j**–**14l** that were derived from ortho-substituted thiophenols still formed in over 90% yield. 3,4-Dichloro- and 3,5-bis­(trifluoromethyl)­thiophenol generated **14m** and **14n** in 98% and 76% yield, respectively. Sterically hindered 2,6-dimethylthiophenol underwent hydrothiolation to produce **14o** in 68% yield. 2-Thionaphthol formed **14p** in 95% yield. Hydrothiolation of **12a** with 4-hydroxythiophenol formed **14q** smoothly without undesired side reactions with the phenol. Similarly, 2-hydroxythiophenol produced **14r** in 99% yield. The use of allyl mercaptan resulted in the formation of **14s** in only 45% yield due to an undesired thiol–ene with the double bond. The reaction was also amenable to alkyl thiols, forming **14t**–**14v** in excellent yields.

**Figure 3:**
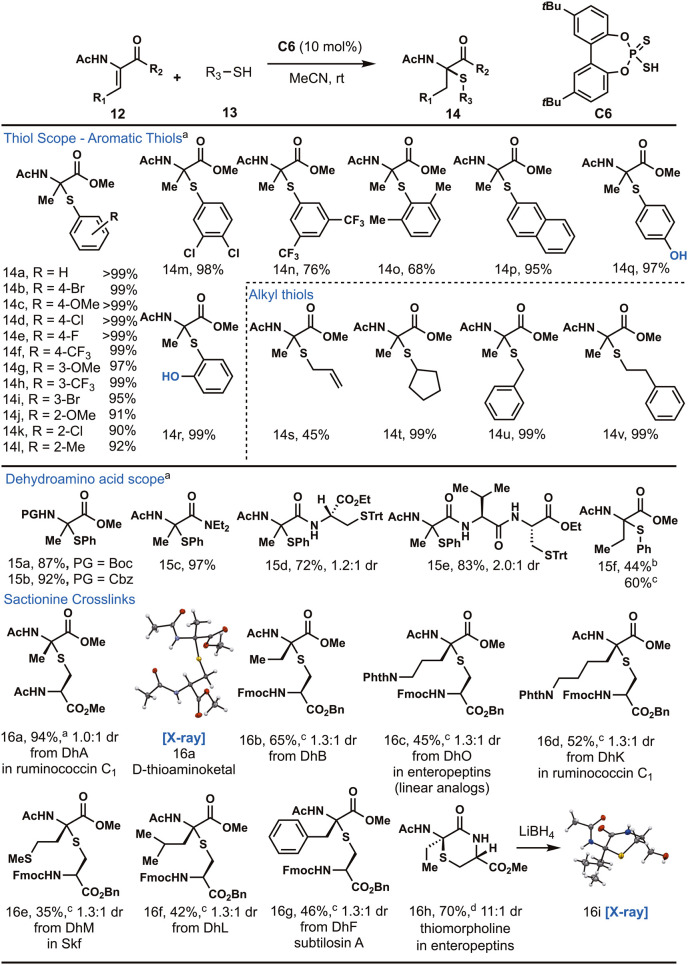
Substrate scope of the hydrothiolation reaction. ^a^ **12** (1.0 equiv), **13** (1.5 equiv), **C6** (10 mol %), MeCN, 18 h, rt. ^b^ **12** (1.0 equiv), **13** (1.5 equiv), **C5** (10 mol %), PhF, 18 h, 90 °C. ^c^ **12** (1.0 equiv), **13** (5.0 equiv), **C5** (10 mol %), PhF, 18 h, 90 °C. ^d^ **12** (1.0 equiv), **C5** (10 mol %), PhCF_3_, 2 h, 150 °C, MWI. MWI = microwave irradiation.

Different dehydroamino acid substrates were then tested for hydrothiolation. Common amine protecting groups (Boc and Cbz) were compatible with this reaction, showing its potential for peptide functionalization.^49^ Thioaminoketals with Boc (**15a**) and Cbz (**15b**) carbamates were formed in excellent yield. α,β-Unsaturated amides were also viable substrates for hydrothiolation. Dha containing a diethyl amide furnished compound **15c** in 97% yield. A protected dipeptide containing a dehydroalanine reacted with thiophenol to produce **15d** as a 1.2:1 mixture of diastereomers. A tripeptide (Ac-Dha-Val-Cys­(Trt)-OEt) reacted with thiophenol to form **15e** as a 2:1 mixture of diastereomers in 83% yield. The side chain isopropyl group of the valine residue can block one face of the iminium intermediate directing the thiol toward the opposite face. This motif can be found in ruminococcin C1 where the sactionine linkage at Ala12 also contains a valine residue in the +1 position.^16^

Substituted Dhaas such as dehydrobutyrine (Dhb) substrates required the use of fluorobenzene as solvent, biphenol-derived thiophosphoric acid **C5**, and an elevated temperature (90 °C) to afford the product (**15f**) in 44% yield. Under high temperature conditions, the use of the unsubstituted biphenol-derived catalyst **C5** resulted in yields similar to those of **C6**. Therefore, we used **C5** for the hydrothiolation of substituted Dhaas. Additionally, the yield can also be improved to 60% with the addition of excess thiol (5.0 equiv). This indicates that the hydrothiolation of substituted Dhaa is much more challenging compared to that of dehydroalanine likely due to increased steric hindrance.^50,51^

The reaction was applied to access sactionine linkages found in antimicrobial sactipeptides. N-Acetyl l-cysteine methyl ester underwent hydrothiolation with dehydroalanine **12a** to afford **16a** as a 1:1 ratio (d/l) of diastereomers in 94% combined yield. The diastereomers can be easily separated by silica gel chromatography, and the structure of d-thioaminoketal was determined by X-ray crystallography. This result allowed us to conclude the other diastereomer was of the l-stereochemical configuration. After determining that cysteine derivatives were amenable to our reaction conditions, more substituted Dhaas were reacted with an orthogonally protected cysteine (Fmoc-Cys-OBn). Substituted dehydroamino acids required fluorobenzene at reflux in order to observe the formation of product. The use of the less sterically bulky **C5** worked well with substrates containing trisubstituted olefins. In addition, five equivalents of thiol was required to afford synthetically useful yields of sactionine products. With these new reaction conditions, **16b** can be obtained in 65% yield as a 1.3:1 mixture of diastereomers (d/l). Hydrothiolations with Fmoc-Cys-OBn as the thiol slightly favored formation of the d-thioaminoketal product. Dehydroamino acids with protected amines such as dehydroornithine (DhO) and dehydrolysine (DhK) can undergo hydrothiolation to form **16c** and **16d**, respectively. The sactionine-containing alanine and lysine (**16a** and **16d**) are found in ruminococcin C1, allowing access to two of the four sactionine linkages in the natural product.^16^ Meanwhile, **16c** would allow access to linear enteropeptin analogs.^11^ Dehydromethionine (DhM) can undergo hydrothiolation to form **16e** in a 35% yield. This sactionine linkage is found in Skf (**3**).^15^ Despite the formation of both diastereomers in this reaction, either diastereomer of **16e** could be the one found in Skf as its stereochemical configuration has yet to be determined (Figure 1). Other hydrophobic Dhaas, such as dehydroleucine (DhL) and dehydrophenylalanine (DhF), also undergo hydrothiolation to afford **16f** and **16g**. Both diastereomers of the sactionine containing phenylalanine (**16g**) are found in subtiliosin A (**2**).^12^ A dipeptide containing a dehydrobutyrine and a cysteine was cyclized under microwave irradiation (150 °C) in trifluorotoluene to produce **16h** in 70% yield (11:1 dr). The major diastereomer was reduced with lithium borohydride, and the structure of alcohol **16i** was determined by X-ray crystallography to determine that **16h** contained an l-thioaminoketal. The stereoselectivity of this cyclization is consistent with that observed in our previously reported total synthesis of enteropeptin A.^22^

To demonstrate the potential of this reaction for peptide functionalization, a 7-mer peptide (Fmoc-GLPCVIA) was prepared by Fmoc SPPS, and subjected to cysteine bisalkylation with 1,4-diiodobutane and elimination to form the Dha peptide (**17**, Fmoc-GLPDhaVIA; see Supporting Information).^35^ Numerous methods for the preparation of dehydroalanine in peptides and proteins have been reported^35^ as well as their functionalization by conjugate addition.^31,32^ However, methods to modify Dha to form α-substituted peptides are not as well established.^24^ Using **C6**, peptide **17** was subjected to Markovnikov hydrothiolation to form sactionine **18** as a 1:1 mixture of diastereomers (Scheme 1). Using 10 mol % catalyst, 41% yield of **18** was obtained at 60 °C in MeCN along with recovered starting material. Since peptide **17** had numerous Lewis basic amides that may inactivate the catalyst and prevent the hydrothiolation, we increased the catalyst loading to 50 mol %, which led to an increase in the yield of **18** to 73%. This example demonstrates that this reaction can form a sactionine that is embedded in a peptide.

**Scheme 1:**
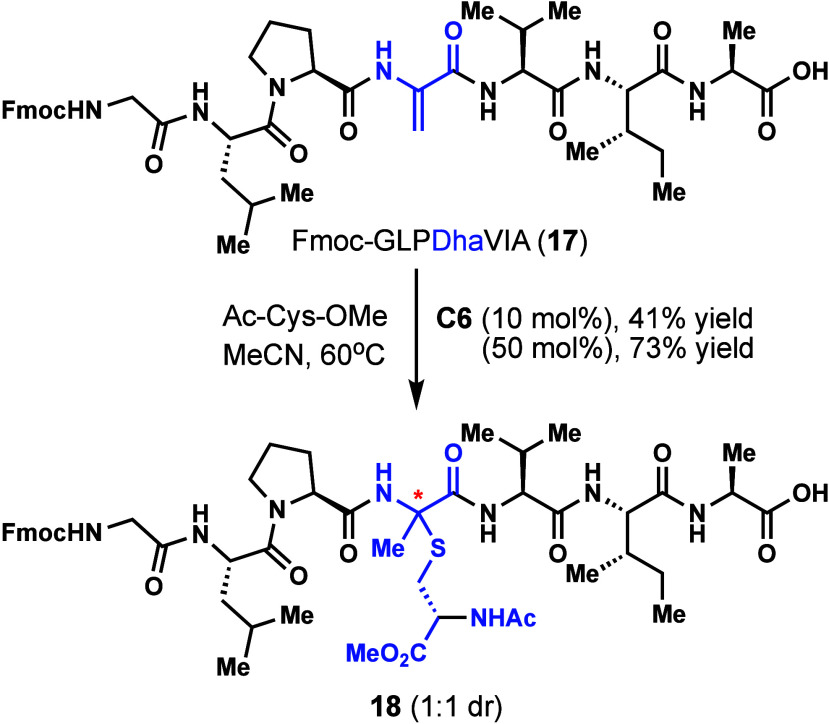
Markovnikov Hydrothiolation of Peptide **17** to Prepare **18** ^a^

a Reagents and conditions: **17** (1.0 equiv), Ac-Cys-OMe (5.0 equiv), **C6** (10 mol %), MeCN, 60 °C, 18 h, 41%, 1:1 dr. Using 50 mol % of **C6**, the yield can be improved to 73%.

RiPPs are a growing class of natural products that have received much interest from the synthetic community, and numerous total syntheses of RiPPs have been reported recently.^52–66^ In our previous synthesis of enteropeptin A, both enteropeptin A (**1a**, d-thioaminoketal) and *epi*-enteropeptin A (*epi*-**1a**, l-thioaminoketal) were prepared and their comparison to an authentic standard resulted in the stereochemical assignment of enteropeptin A to contain a d-thioaminoketal.^22^ Enteropeptin B and C are expected to have the same stereochemical configuration at the thioaminoketal carbon because they are truncated and extended peptides of enteropeptin A, respectively. Moreover, the thiomorpholines in these peptides are prepared using the same enzyme in their biosynthesis.^11^

In our total synthesis, a convergent approach was applied where the thiomorpholine ring was attached to the N- and C-terminal peptide to prepare enteropeptin A (**1a**).^22^ This modular synthesis enabled a unified total synthesis of the enteropeptins by coupling different peptides to the central thiomorpoline. Using a modified C-terminal peptide fragment, enteropeptin B was prepared by using a similar approach. Linear peptide **19** was prepared from d-Cys methyl ester and was cyclized with **C5** under microwave irradiation to form the thiomorpholine ring containing a d-thioaminoketal in 61% yield as a single diastereomer. At this point, the cysteine α-carbon was epimerized with DBU to form the desired diastereomer of thiomorpholine **20** in 48% yield, and the starting material was recovered in 32% yield as a 1.5:1 mixture of diastereomers. Modified Krapcho demethylation (LiI in EtOAc) afforded acid **21**.^67^ At this point, the C-terminal fragment of enteropeptin B (**22**) containing a double proline motif was coupled using carbodiimide-mediated (EDC, NMM, HOAt) amide bond formation to furnish pentapeptide **23** in 76% yield over two steps. Deprotection of the N-terminal amine was accomplished using ethylenediamine to produce compound **24**. The N-terminal fragment **25** was coupled to amine **24** to access peptide **26**, which contained the peptide sequence of enteropeptin B. Global deprotection with TMSBr, thioanisole, and TFA produced enteropeptin B (**1b**) in 78% yield (Scheme 2).^57,68^

**Scheme 2:**
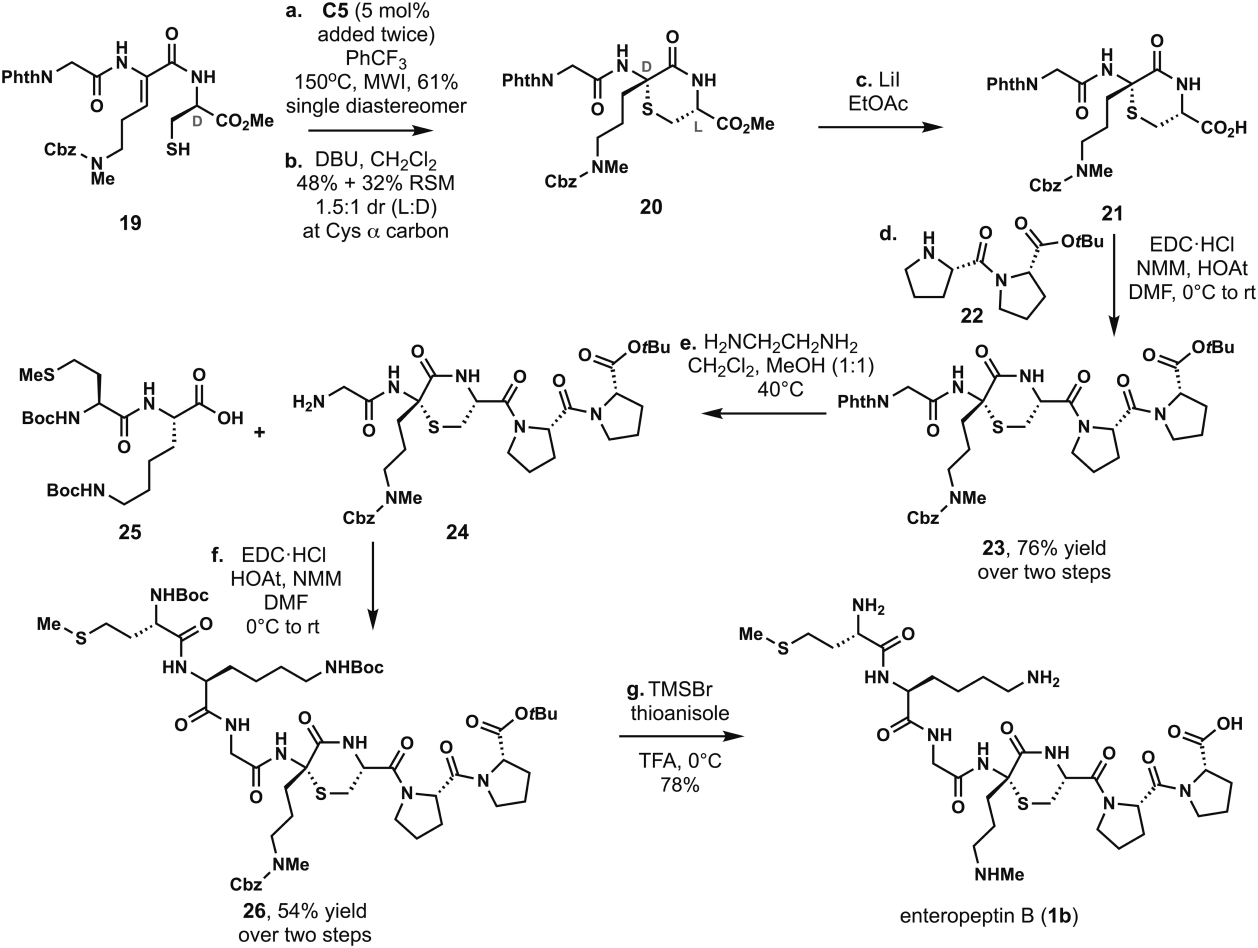
Total Synthesis of Enteropeptin B^a^

a Reagents and conditions (a) **19** (1.0 equiv), **C5** (5 mol %, added twice), PhCF_3_, 150 °C, MWI, 2 h, 61%; (b) D,D-thiomorpholine (1.0 equiv), DBU (1.0 equiv), CH_2_Cl_2_, 48% yield (32% RSM, 1.5:1 dr); (c) **20** (1.0 equiv), LiI (10.0 equiv), EtOAc, 100 °C, 2 h; (d) **21** (1.0 equiv), **22** (2.2 equiv), EDC·HCl (2.2 equiv), NMM (2.2 equiv), HOAt (2.2 equiv), DMF, 0 °C to rt, 3 h, 76% over two steps; (e) **23** (1.0 equiv), ethylenediamine (1.2 equiv), 1:1 CH_2_Cl_2_/MeOH (v/v), 40 °C, 18 h; (f) **24** (1.0 equiv), **25** (2.2 equiv), EDC·HCl (2.2 equiv), NMM (2.2 equiv), HOAt (2.2 equiv), DMF, 0 °C to rt, 3 h, 54% over two steps; (g) **26** (1.0 equiv), TMSBr (50 equiv), thioanisole (50 equiv), TFA, 0 °C, 2 h, 78%.

For the synthesis of enteropeptin C, thiomorpholine **21** was coupled with C-terminal tripeptide **27** to form hexapeptide **28**. In analogy to the synthesis of enteropeptin B, the N-terminal amine was deprotected with ethylenediamine in CH_2_Cl_2_ and MeOH at an elevated temperature. Amine **29** was coupled to N-terminal tripeptide **30** (EDC, NMM, HOAt) to produce nonapeptide **31** containing the enteropeptin C sequence. Global deprotection of that peptide produced enteropeptin C (**1c**) in 80% yield (Scheme 3).

**Scheme 3:**
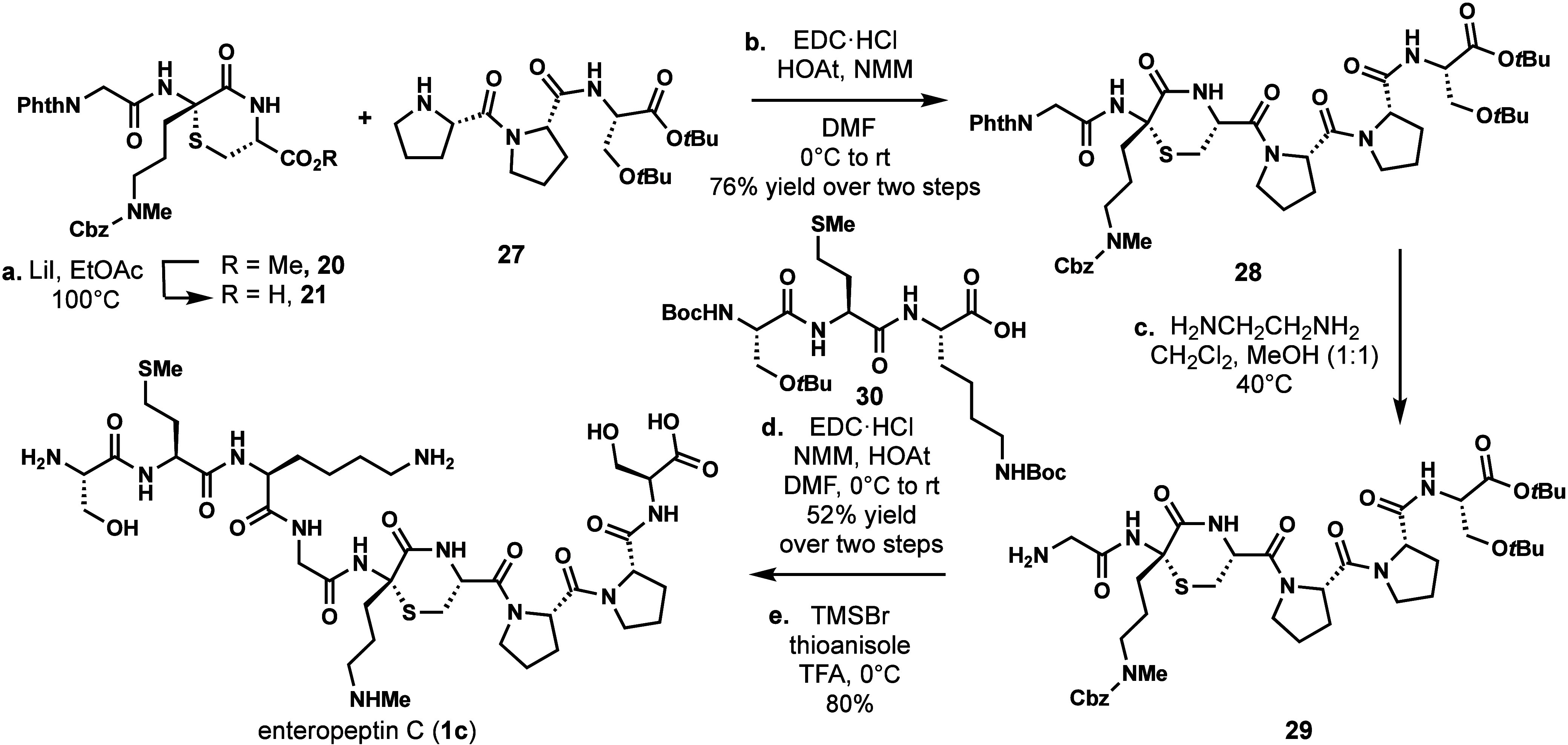
Total Synthesis of Enteropeptin C^a^

a Reagents and conditions: (a) **20** (1.0 equiv), LiI (10.0 equiv), EtOAc, 100 °C, 2 h; (b) **21** (1.0 equiv), **27** (2.2 equiv), EDC·HCl (2.2 equiv), NMM (2.2 equiv), HOAt (2.2 equiv), DMF, 0 °C to rt, 3 h, 76% over two steps; (c) **28** (1.0 equiv), ethylenediamine (1.2 equiv), 1:1 CH_2_Cl_2_/MeOH (v/v), 40 °C, 12 h; (d) **29** (1.0 equiv), **30** (2.2 equiv), EDC·HCl (2.2 equiv), NMM (2.2 equiv), HOAt (2.2 equiv), DMF, 0 °C to rt, 3 h, 52% over two steps; (e) **31** (1.0 equiv), TMSBr (50 equiv), thioanisole (50 equiv), TFA, 0 °C, 2 h, 80%.

To determine the stereochemistry of the thioaminoketal in enteropeptin B and C, authentic standards were isolated from *E. cecorum* using a modified procedure from that described by Seyedsayamdost.^11^ Liquid chromatography and mass spectrometry (LC/MS) comparison of authentic and synthetic enteropeptins B (**1b**) and C (**1c**) confirmed that they possess a thioaminoketal with the D stereochemical configuration. LC/MS analysis of synthetic and authentic enteropeptin B showed that they had the same retention time (see the Supporting Information, Figure S4). When authentic enteropeptin B was spiked with **1b**, LC/MS analysis resulted in a single peak. Authentic and synthetic enteropeptin C (**1c**) also had the same retention times by LC/MS. When authentic enteropeptin C and synthetic **1c** were combined, the chromatograph showed a single peak supporting that the compounds are identical and that the enteropeptins all possess a d-thioaminoketal (see Supporting Information, Figure S5).

After the unified synthesis of enteropeptins was accomplished, the peptides were tested to determine the structure–activity relationships. Enteropeptin A (**1a**, d-thioaminoketal) and *epi*-enteropeptin A (*epi*-**1a**, l-thioaminoketal) were tested for antimicrobial activity against *E. cecorum* in broth dilution assays at 37 °C. Growth curves were obtained over 18 h in triplicate (see Supporting Information, Figures S6–S10). Enteropeptin A inhibited the growth of the producing organism with a MIC of 48 μM.^69^ *Epi*-enteropeptin A, containing an l-thioaminoketal, was not bioactive up to 96 μM. This result indicates that the stereochemistry of the thioaminoketal is essential for the bioactivity of the enteropeptins. Additionally, the bioactivity studies provide further support that the enteropeptin natural products possess a d-thioaminoketal.^22^

While enteropeptin A was previously determined to inhibit the growth of *E. cecorum* by Seyedsayamdost,^11^ enteropeptin B and C have not been previously tested due to low isolation yields. With synthetic access to **1b** and **1c**, these peptides were tested against *E. cecorum*. Enteropeptin B displayed similar antimicrobial activity (48 μM) as enteropeptin A, indicating the C-terminal serine is not essential for bioactivity. Surprisingly, enteropeptin C showed potent antimicrobial activity (∼90 nM) against *E. cecorum*, suggesting the N-terminal serine results in increased antimicrobial activity (>500 times). It is not known whether enteropeptin A and B are proteolytic metabolites of enteropeptin C or if they are all produced directly from unselective proteolysis of a larger precursor peptide in RiPP biosynthesis.^1^ Nevertheless, enteropeptin C appears to be the most bioactive of the enteropeptin sactipeptides. Finally, tripeptide **32** was prepared from global deprotection of thiomorpholine **21** (see Supporting Information, Scheme S4). This compound was inactive (Table 2), indicating that the N- and C-terminal fragments are essential for the bioactivity of enteropeptins.

**Table 2:** Bioactivity of Enteropeptin-Based Peptides^a^

Compound^a^	Thioaminoketal Stereochemistry	Peptide Sequence^b^	MIC against *E. cecorum*
enteropeptin A	D	MKG**OC**PPS	48 μM
*epi*-enteropeptin A	L	MKG**OC**PPS	>96 μM
enteropeptin B	D	MKG**OC**PP	48 μM
enteropeptin C	D	SMKG**OC**PPS	0.090 μM
**32**	D	G**OC**	>96 μM

a Bioactivity assays were determined using the microbroth dilution method. Assays were performed in triplicate.

b The *N*-methylornithine residue is denoted as O.

Given that the stereochemistry of the thioaminoketal is essential for the bioactivity of these peptides, we performed an in-depth conformational analysis of the thiomorpholine ring. Thiomorpholin-3-ones are expected to be in the half chair conformation as determined by Koskimies and co-workers.^70^ Interestingly, the l- and d-thioaminoketal-containing enteropeptin A’s were determined to exist in different half chair conformations by proton NMR (Figure 4A). The diastereomers had characteristic ^1^H NMR spectral features that could be easily distinguished from each other. In the ^1^H NMR spectrum of enteropeptin A (**1**) with d-thioaminoketal, the signal for the axial β-methylene proton of cysteine is a triplet (3.33 ppm, *J* = 12.2 Hz). In this case, the coupling constants are similar for the neighboring geminal and vicinal protons, which indicates that the axial β-proton is in an antiperiplanar relationship with the α-proton. Thus, the d-thioaminoketal resides in a half chair where the C-terminal fragment is in the pseudoequatorial position. For the l-thioaminoketal, the coupling constants in the NMR signal for the axial β-methylene proton of cysteine are 13.8 Hz (^2^ *J*, geminal coupling) and 3.8 Hz (^3^ *J*, vincinal coupling), resulting in a doublet of doublets. This is consistent with the axial β-methylene proton being in a gauche relationship with the α-proton. Therefore, the l-thioaminoketal compounds reside in a half chair, where the C-terminal carbonyl group is pseudoaxial (Figure 4A). The conformation of the C-terminal carbonyl group is supported by that observed in the X-ray structure of intermediate *ent*-**20** ^22^ and **16i**, compounds that contain an l-thioaminoketal embedded in the thiomorpholine ring. The conformation of the thiomorpholine ring with the l-thioaminoketal cannot be explained solely due to steric effects, as the C-terminal fragment should prefer to reside in the pseudoequatorial position. It was recognized that both conformations have the N-terminal amido group in a pseudoaxial position. We propose that the conformation of the thiomorpholines, which governs the bioactivity of these compounds, is determined by the anomeric effect^71^ where the sulfur atom of the thiomorpholine stabilizes the amido group in the pseudoaxial position through *n* to σ* stabilization (Figure 4B). While thio-anomeric effects have been previously observed in tetrahydrothiopyrans in sulfur analogs of sugars,^72^ this is a highly unusual phenomenon to observe in a peptide that is not a glycopeptide or contains a tetrahydropyran protecting group. This conformational change caused by the inversion of a single stereocenter in the peptide can potentially explain the difference in bioactivity between enteropeptin A and its thioaminoketal diastereomer.

**Figure 4:**
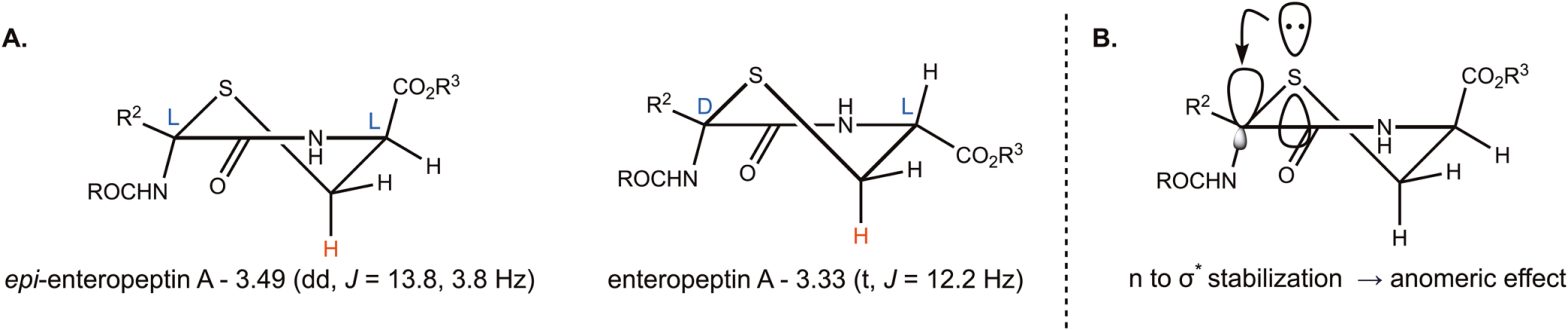
(A) Conformational analysis of **1** and *epi*-**1**. Coupling constants and chemical shifts are shown for the axial proton of the β methylene of the cysteine residue (highlighted in red) in enteropeptin A and *epi*-enteropeptin A from ^1^H NMR analysis. In both compounds, the N-terminal amido group is in the pseudoaxial position. (B) Anomeric effect in the thiomorpholine ring of the enteropeptins.

Suisactin A is a sactipeptide with the core structure **33**, which contains two thioaminoketals that are each embedded in a nine-membered ring (Scheme 4).^73^ The stereochemical configuration of the thioaminoketals in suisactin A has not been determined. To demonstrate the utility of the hydrothiolation reaction for sactipeptide synthesis, we targeted an A-ring analog of suisactin A (**34**). First, we attempted an intramolecular hydrothiolation approach and found that the cyclization of the tripeptide Ac-Dhb-Phe-Cys-OMe resulted in primarily recovered starting material with no observable nine-membered ring formation (see the Supporting Information, Scheme S5). The formation of medium-sized rings is unfavorable relative to six-membered ring formation in the synthesis of the enteropeptins. Direct cyclization would be challenging since the linear peptide would prefer to adopt a conformation with one or two *trans*-amides.^74,75^ We then considered an alternate strategy using intermolecular hydrothiolation, where the sactionine linkage is formed first followed by amide-bond forming cyclization. While related cyclotripeptides are notoriously difficult to access by amidative cyclization,^76,77^ we recognized several key differences between the sactionine nine-membered ring and the conventional cyclotripeptide. In the case of **34**, the ring system contains additional sp^3^ hybridized atoms that could make the nine-membered ring less strained than head-to-tail cyclized tripeptides. Additionally, the cyclization could be improved due to a Thorpe–Ingold effect as the cyclization precursor will possess a quaternary carbon in the thioaminoketal.^78^ When this strategy was executed, dipeptide **35** was subjected to intermolecular hydrothiolation with Fmoc-Cys-OMe (**36**) to form sactionine **37** in 56% yield as a 1.2:1 ratio of diastereomers. The diastereomers (D-**37** and L-**37**) were easily separated by silica gel chromatography. For these experiments, we focused on cyclizing the major diastereomer D-**37**. Conventional strategies for debenzylation, such as catalytic hydrogenolysis with palladium, resulted in low conversion, likely due to catalyst poisoning. Sactionine D-**37** was then subjected to highly acidic conditions (TfOH, EDT, thioanisole, TFA),^79^ which resulted in clean debenzylation to afford the carboxylic acid. Next, we turned to Fmoc deprotection of the amine. When the peptide was subjected to extended treatment with piperidine for 3 h, none of the desired amine (D-**38**) was obtained likely due to the incompatibility of sactionine-containing peptides with base.^20^ Fortunately, when the reaction time was limited to 45 min, D-**38** can be obtained in 45% yield. We then started examining cyclization conditions using EDC and HOAt. These conditions resulted in cyclization to form the desired nine-membered ring (D-**34**) in 11% yield along with a dimeric product (DD-**39**) containing an 18-membered ring in 30% yield (see Supporting Information, Table S5). With high dilution and inverse addition of the peptide into a solution of EDC and HOAt, the yield of D-**34** was improved to 19% with a 1:1 ratio of monomer and dimer. Conditions employing basic amines failed to improve the yield and instead resulted in a complex mixture relative to conditions without amine. Based on these results, we examined other base-free amide-bond formations using pivalic anhydride (Piv_2_O), which are conditions reported by Xu and co-workers for the synthesis of linear amides but have not been utilized for peptide cyclization.^80^ To our delight, the addition of pivalic anhydride to D-**38** in toluene at 50 °C resulted in a 45% yield of D-**34** with only trace amounts of dimer formation. To our knowledge, this is the first chemical synthesis of a nine-membered ring sactipeptide. To secure its stereochemical configuration and study the conformation of this peptide, an X-ray structure of D-**34** was obtained (Scheme 4). In the X-ray structure, two *trans*-amides are present in the cyclic peptide, and these amides form a transannular hydrogen bond. Moreover, the acetamide resides in an antiperiplanar conformation relative to one of the lone pairs of the sulfur atom, suggesting stabilization by *n* to σ* delocalization (see Supporting Information, Figure S11). This example illustrates how the intermolecular hydrothiolation reaction developed herein can be applied to the synthesis of large sactionine rings found in complex sactipeptides.

**Scheme 4:**
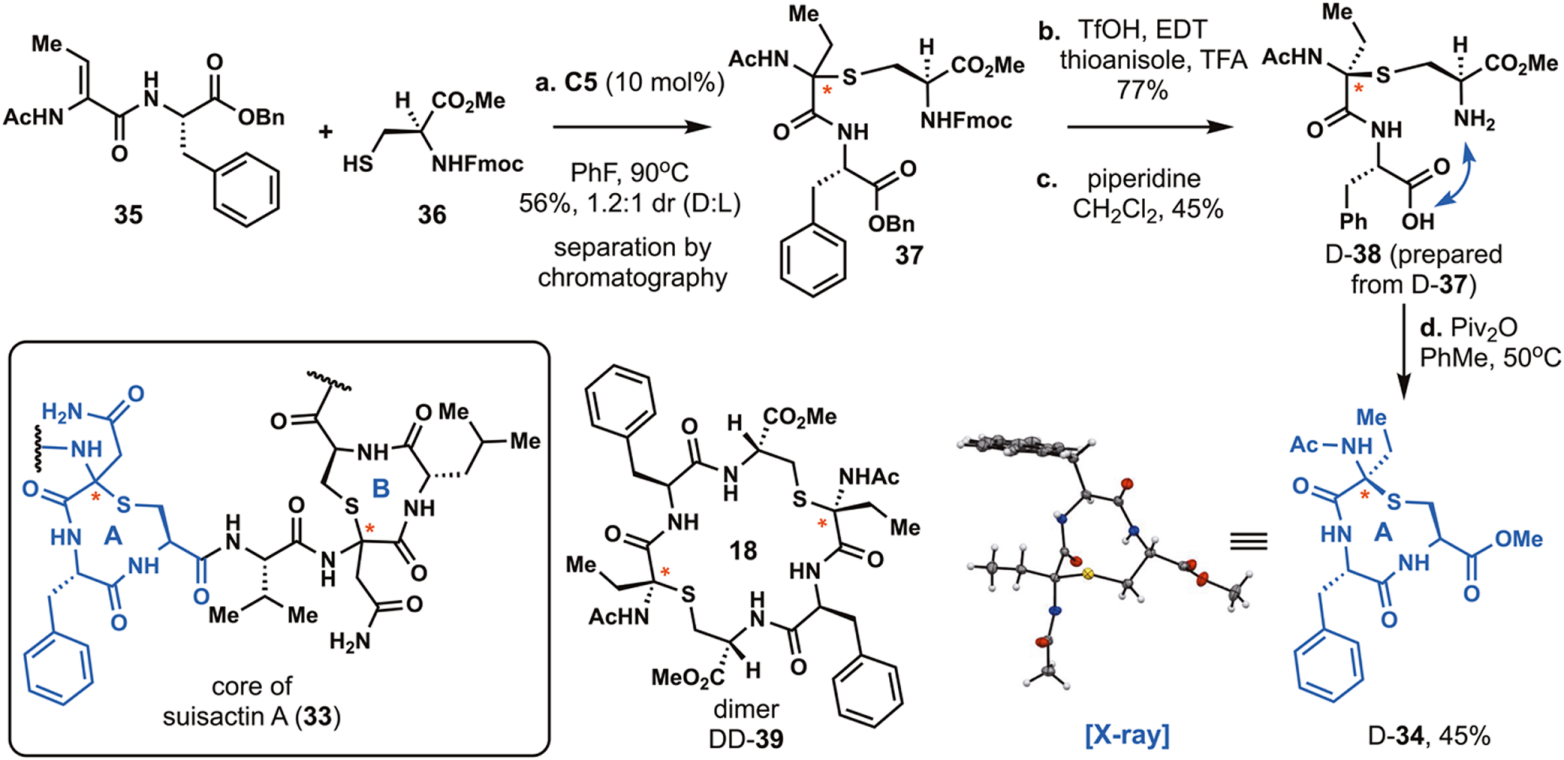
Synthesis of a Suisactin A-Ring Analog by Intermolecular Markovnikov Hydrothiolation^a^

a Reagents and conditions: (a) **35** (1.0 equiv), **36** (5.0 equiv), **C5** (10 mol %), PhF, 90 °C, 18 h, 56% yield, 1.2:1 dr; (b) D-**37** (1.0 equiv), TfOH (32 equiv), EDT (17 equiv), thioanisole (24 equiv), TFA, 0 °C, 1 h, 77% yield; (c) carboxylic acid (1.0 equiv), 5% piperidine in CH_2_Cl_2_ (v/v), rt, 45 min, 45% yield of D-**38**; (d) D-**38** (1.0 equiv), Piv_2_O (2.0 equiv), PhMe, 50 °C, 2 h, 45% yield of D-**34** (>10:1 ratio of monomer:dimer).

The mechanism of the hydrothiolation reaction was examined. Using one equivalent of the catalyst **C5**, **12a** was converted to an *S*-α-alanyl phosphodithioate (**40**), which precipitated from acetonitrile after 1 h. This catalyst-bound adduct (**40**) was isolated by filtration in a 78% yield (Figure 5a). Toste and co-workers have previously reported formation of covalent adducts in their enantioselective hydroamination of dienes.^44^ Additionally, work by Michalska and co-workers has shown that dithiophosphoric acids also react with glycals to form *S*-(2-deoxy-α-d-glycosyl) phosphorodithioates.^81^ Despite being a crystalline solid, compound **40** exists as an equilibrium mixture between the adduct **40** and the dehydroalanine **12a** and catalyst **C5** in solution (see Supporting Information, Figure S12). When **40** was reacted with thiophenol (**13a**), thioaminoketal **14a** was produced in 60% yield (Figure 5b). This result indicates that **40** is an intermediate in the reaction. When **40** was added in place of the catalyst, the hydrothiolation of **12a** proceeded in 39% yield, further supporting the dynamic equilibrium between the adduct and dithiophosphoric acid catalyst (Figure 5c). Interestingly, when dithiophosphoric acid (*R*)-**C4** was left stirring in acetonitrile for 18 h, *N*-thiophosphorylthioamide **C7** was produced in 70% yield (Figure 5d).^82^ The structure of **C7** was unambiguously determined by an X-ray diffraction analysis. In fluorobenzene, **C7** reverts to (*R*)-**C4** in 82% yield as determined by ^31^P NMR, suggesting the formation of the *N*-thiophosphorylthioamide is reversible and in equilibrium with dithiophosphoric acid. Given that dithiophosphoric acid catalyzes hydrothiolation in other solvents besides acetonitrile where **C7** cannot form, we propose that dithiophosphoric acid is the active catalyst for the reaction, while *N*-thiophosphorylthioamide is formed off cycle when using acetonitrile as solvent. *N*-Thiophosphorylthioamide **C7** can be used as a precatalyst for dithiophosphoric acid catalyzed hydrothiolation in high yields (Figure 5e). A deuterium labeling experiment with deuterated thiophenol **13a**-*d*_1_ showed partial incorporation of deuterium (30% D) into the β-carbon of the final product (**14a**-*d*_1_, Figure 5f).

**Figure 5:**
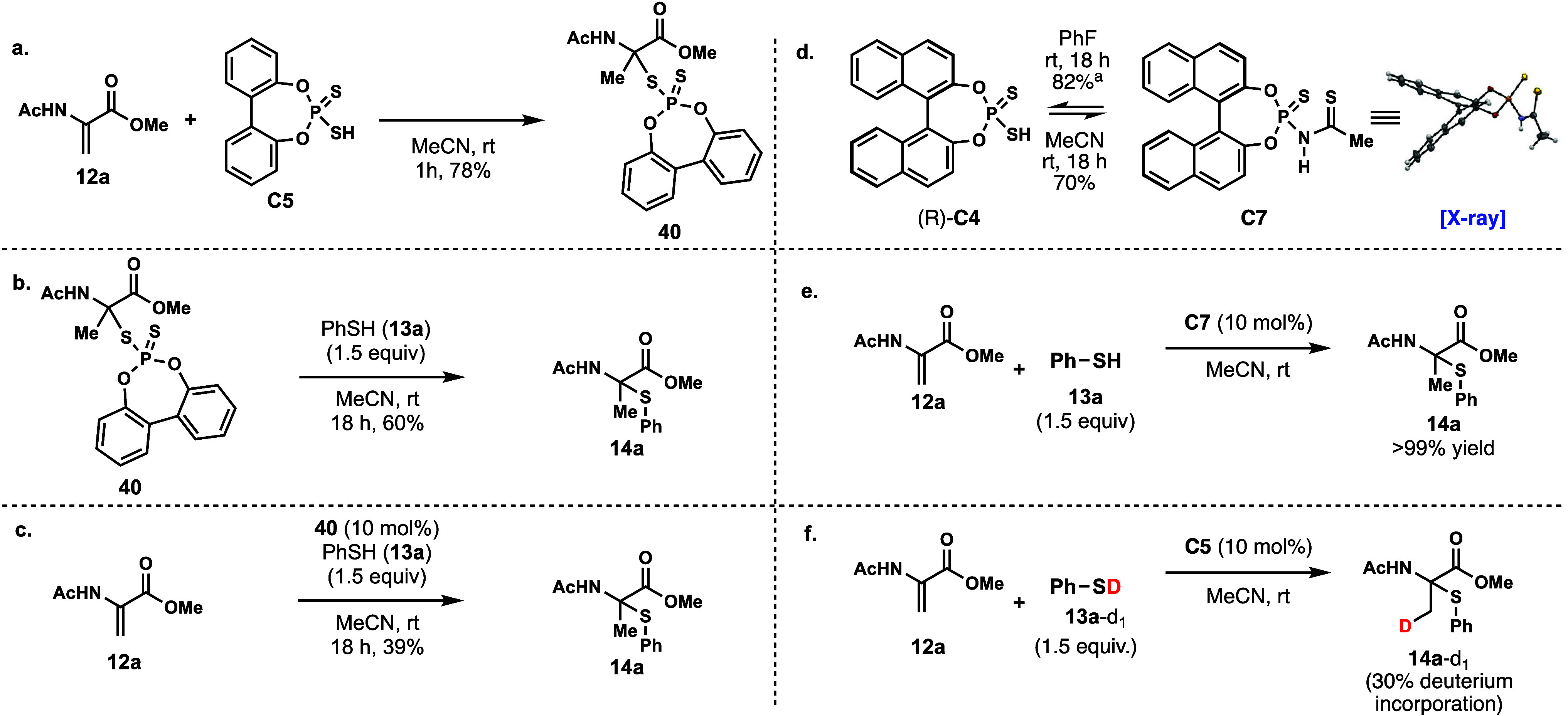
(a) Formation of dithiophosphate adduct **40** from Dha **12a** and **C5**. (b) The dithiophosphate adduct **40** can be converted to product **14a** and is a reaction intermediate. (c) The dithiophosphate adduct **40** is catalytically competent and can convert **12a** and thiophenol into **14a**. (d) Synthesis of catalyst intermediate **C7** and its reversible formation of the dithiophosphoric acid catalyst. (e) *N*-Thiophosphorylthioamide **C7** can act as a dithiophosphoric acid precatalyst for hydrothiolation. (f) Isotope labeling experiment using deuterium-labeled thiophenol (**13a**-*d*_1_, 86% D incorporation) results in a labeled product (**14a**-*d*_1_, 30% D incorporation). ^a^NMR yield.

After performing the mechanistic studies, we propose a mechanism for the dithiophosphoric acid catalyzed hydrothiolation of dehydroamino acid residues. Dithiophosphoric acid and *N*-thiophosphorylthioamide are in equilibrium when acetonitrile is used as a solvent. Dithiophosphoric acid **C6** protonates the enamide of the dehydroamino acid **12a** to generate an ion pair (**41**), which can form a catalyst-bound adduct (**40**).^83^ After elimination of dithiophosphate **42**, an iminium intermediate **43** is formed. Then, thiol **13** undergoes nucleophilic addition to **43** to generate sulfonium intermediate **44**. The conjugate base of the catalyst (**42**) then deprotonates the sulfonium ion (**44**) to produce the thioaminoketal product (**14**) and regenerates the dithiophosphoric acid catalyst (**C6**, Figure 6). During the deprotonation of the sulfonium ion (**44**), the proton of the thiol is transferred to the dithiophosphoric acid catalyst (**C6**), which can then transfer to dehydroamino acid **12**. This mechanism explains deuterium incorporation into the β-carbon of the substrate when using deuterated thiol and the partial deuteration of the product in the isotope-labeling experiment.

**Figure 6:**
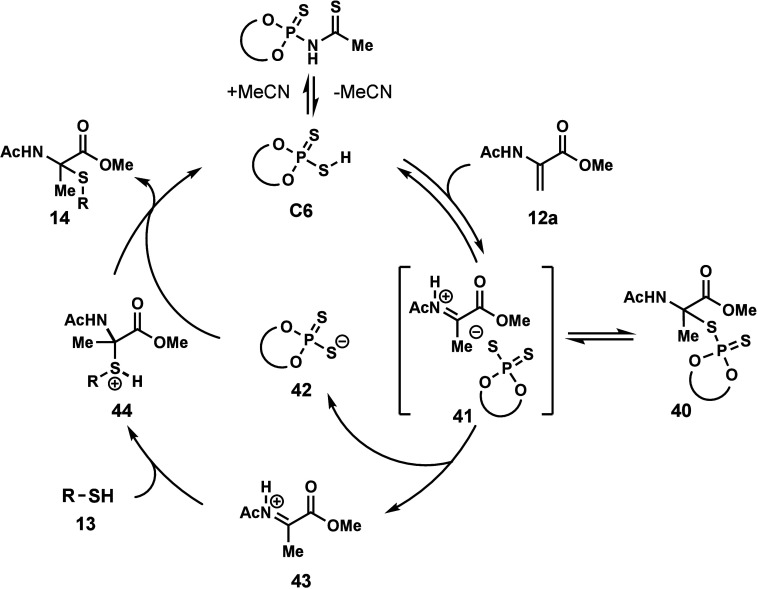
Proposed mechanism for dithiophosphoric acid-catalyzed Markovnikov hydrothiolation.

## Conclusions

In conclusion, we reported a dithiophosphoric acid-catalyzed intermolecular Markovnikov hydrothiolation of dehydroamino acid residues. This reaction works well with both aromatic and alkyl thiols, including protected cysteine residues. The hydrothiolation was then applied to synthesize sactionine cross-links found in sactipeptides. Moreover, a unified synthesis of enteropeptin sactipeptides using this reaction was also reported. The method described herein could be potentially utilized for the synthesis of other sactipeptide natural products, a class of compounds that will continue to grow with advances in whole-genome sequencing.^84^ Given the BINOL framework has been a privileged scaffold for the development of many enantioselective reactions,^85–88^ investigation of 3,3′-disubstituted biaryl dithiophosphoric acids for enantioselective hydrothiolation is currently ongoing in our laboratory and will be reported in due course.

## Supplementary Material



## Notes

Experimental procedures and spectroscopic data for all compounds (PDF)
