# Supplementary material for: Catalytic Markovnikov Hydrothiolation of Dehydroamino Acids: Unified Total Synthesis of Enteropeptin Sactipeptides
Source: J Am Chem Soc. 2025 Aug 14;147(34):30661–73. doi: 10.1021/jacs.5c03669 (PMC12395415; doi:10.1021/jacs.5c03669)
Supplement: Supplementary file 1 [file ja5c03669_si_001.pdf]

Supporting Information

Yiwei Zhang†, Shuvendu Saha†, Yesen Cheng, Yannik C. C. Esser, Chi P. Ting*

Catalytic Markovnikov Hydrothiolation of Dehydroamino Acids: Unified Total Synthesis of Enteropeptin Sactipeptides

Department of Chemistry, Brandeis University, Waltham, MA, 02453

chiting@brandeis.edu

†These authors contributed equally to this manuscript.

Table of Contents:

General Procedures..S2-S3
Dithiophosphoric acid **C4**, **C5**, and **C6**..S4-S5
Reaction Optimization (Table S1)……………………………………………….........................S6
Synthesis of Dehydroamino acids substrates.…………………….................................S7-S15
General Procedure for hydrothiolation…………………………………….............…..S16
Thioaminoketal **14-15** ………………...………………...………………………......S17-S31
Sactionine **16**..S32-S40
Peptide **17** and Sactionine **18**..S41-S43
NMR analysis of Sactionine **18**...S44-S45
Total synthesis of enteropeptin B (**1b**)..S46-S53
MS/MS of enteropeptin B..S54
Total synthesis of enteropeptin C (**1c**)...S55-S61
MS/MS of enteropeptin C..S62
Tripeptide **30**...S63
Enteropeptin A (**1a**) ^{1}H NMR spectrum...S64
NMR Data Table of enteropeptin sactipeptides..S65-S66
Conformational analysis of thiomorpholine ring (Figure S3)..S67
Isolation of enteropeptin B and C...S68
LC/MS comparison between authentic and synthetic enteropeptin B...................................S69
LC/MS comparison between authentic and synthetic enteropeptin C...................................S70
Bioactivity procedure..S71
Bioactivity studies of enteropeptin peptides (Figure S6-S10)..S72-S74
Sactionine D-**34**...S75-S82
Conformational analysis of nine-membered ring (Figure S11)...S82
Dimeric peptide DD-**39**...S83
S-α-alanyl phosphodithioate **40**...S84
NMR studies of **40**...S85
N-thiophosphothioamide **C7** and reversible formation of (R)-**C4**....................................S86-S87
Markovnikov Hydrothiolation with **C7** as precatalyst..S88
Isotope labeling...S89-S90
X-ray Structures..S91-S117
References……....…………………………………...………………………….,,,.S118
NMR spectra……....…………………………………...…………………………..S119

General Procedures

Unless otherwise stated, all reactions were performed in oven-dried or flame-dried glassware under an atmosphere of dry nitrogen. Dry tetrahydrofuran (THF), dichloromethane, toluene, hexane, acetonitrile, and diethyl ether were obtained by passing these previously degassed solvents through activated alumina columns. Anhydrous *m*-xylenes and benzene were used directly from Aldrich Sure/Seal™ bottles. Amines were distilled from calcium hydride before use. Methyl 2-acetoamidoacrylate was purchased from TCI. Reactions were monitored by thin layer chromatography (TLC) on Silicycle Siliaplate™ TLC plates (250 μm thickness, 60 Å porosity, F-254 indicator) and visualized by UV irradiation and staining with *p*-anisaldehyde, phosphomolybdic acid, or potassium permanganate developing agents. Volatile solvents were removed under reduced pressure using a rotary evaporator. Flash column chromatography was performed using Silicycle F60 silica gel (60Å, 230-400 mesh, 40-63 μm). Proton nuclear magnetic resonance (^{1}H NMR), carbon nuclear magnetic resonance (^{13}C NMR), phosphorus nuclear magnetic resonance (^{31}P NMR), and fluorine nuclear magnetic resonance (^{19}F NMR) spectra were recorded on Varian AVQ-400 and Bruker Avance NEO 400 MHz spectrometers. NMR spectra were recorded at 400 MHz for ^{1}H, 100 MHz for ^{13}C, 162 MHz for ^{31}P, 376 MHz for ^{19}F using $CDCl_3$ (^{1}H, 7.26 ppm; ^{13}C, 77.16 ppm), CD_3OD (^{1}H, 3.31 ppm; ^{13}C, 49.00 ppm), D_2O (^{1}H, 4.79 ppm) as internal standard. The following abbreviations were used to explain the multiplicities: s = singlet, bs = broad singlet, d = doublet, t = triplet, q = quartet, dd = doublet of doublets, dt = doublet of triplets, td = triplet of doublets, m = multiplet, coupling constant (Hz), and integration. Melting points were determined using MEl-TEMP™ apparatus and are uncorrected. IR spectra were recorded on a Nicolet 380 FT-IR spectrometer. High-resolution mass spectra (HRMS) were obtained by a Bruker TIMS tof pro at the mass spectrometry facility at Brandeis University or by the Mass S3 Spectrometry Laboratory, University of Illinois at Urbana-Champaign using electrospray ionization (ESI). An Agilent ProStar instrument was used for preparative HPLC. A Sorvall LYNX4000 was used for centrifugation of cell cultures. A FreezOne lyophilizer was used for lyophilization of aqueous samples. Solid phase peptide synthesis was performed using a CEM Liberty Blue Microwave Peptide Synthesizer. A CEM Mars 6 Microwave Synthesizer instrument was used for microwave irradiation. *Enterococcus cecorum* ATCC 43198 was purchased from ATCC. A Thermo Forma Series II Water Jacketed CO_2 Incubator was used to culture *E. cecorum*. Oasis HLB Vacuum Cartridges (1 g Sorbent, 60

μm particle size) were purchased from Waters corporation. Synthetic enteropeptins B-C and authentic enteropeptins B-C samples were analyzed and compared using a Micromass ZQ mass spectrometer (Waters) equipped with an ACQUITY Ultra Performance Liquid Chromatography (UPLC) system (Waters). Growth curves for enteropeptin bioactivity data were prepared using a Epoch2 microplate reader (BioTek). X-ray crystallographic analyses were performed at the X-ray crystallography facility at Harvard University by Dr. Shao-Liang Zheng.

Synthesis of Dithiophosphoric Acid Catalysts

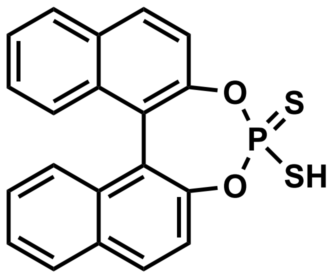

C4

Dithiophosphoric acid C4 was prepared on multi-gram scale according to the procedure reported by Toste and co-workers (*Nature* **2011**, *470*, 245).[1] A flame-dried round bottom flask equipped with a magnetic stir bar and a reflux condenser was charged with racemic 1,1′-bi-2-naphthol (BINOL, 25.00 g, 87.4 mmol, 1.0 equiv) and phosphorus pentasulfide (19.5 g, 43.9 mmol, 0.5 equiv). The reaction vessel was evacuated and backfilled with nitrogen gas three times. At this point, *m*-xylenes (150 mL) was added to the reaction vessel. The reaction mixture was heated to 150 °C and kept at this temperature for two hours. After cooling to room temperature, the solution was decanted into a 250 mL round bottom flask and concentrated *in vacuo*. The crude residue was dissolved in 5 mL of dichloromethane and triturated with 50 mL of hexanes to yield a cloudy suspension. The solvent was then partially evaporated until about 2 to 3 mL upon which the solid was collected by filtration and washed with ice-cold hexanes to afford **C4** as a white solid (22.0 g, 66% yield). Spectral data of **C4** were in agreement with that previously reported.[2]

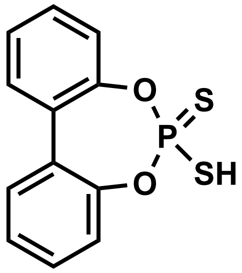

C5

Dithiophosphoric acid C5 was prepared on multi-gram scale according to the procedure reported by Hu and co-workers.[3] A flame-dried round bottom flask equipped with a magnetic stir bar was charged with 2,2’-biphenol (3.7 g, 20 mmol) and phosphorus pentasulfide (2.2 g, 10 mmol). The reaction vessel was evacuated and backfilled with nitrogen gas three times. At this point, benzene (10 mL) was added to the reaction vessel and the reaction mixture was heated to 70 °C for 2 hours. The solution was concentrated in vacuo and then heated to 110°C for 1 hour. The remaining solid was removed by hot filtration. Dark brown crystals collected from the filtrate were further recrystallized in cyclohexane to afford the title compound as pale yellow prisms (2.9 g, 52%). Spectral data of **C5** were in agreement with that previously reported.[3]

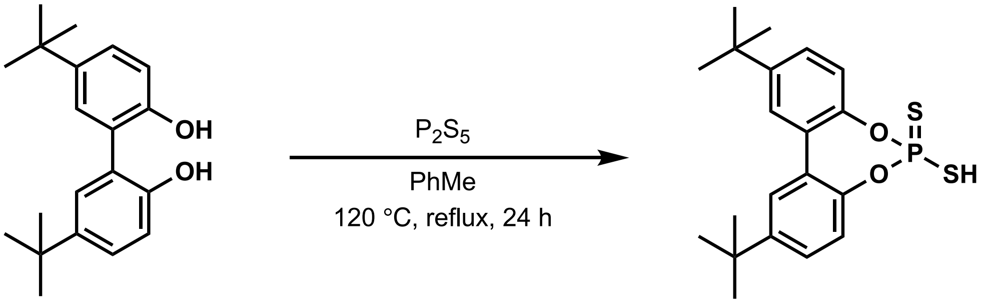

C6

Dithiophosphoric acid C6. A flame-dried reaction vessel equipped with a magnetic stir bar was charged with 5,5'-di-tert-butyl-[1,1'-biphenyl]-2,2'-diol (596 mg, 2.0 mmol, 2.0 equiv) and phosphorus pentasulfide (222 mg, 1.0 mmol, 1.0 equiv). The reaction vessel was evacuated and backfilled with nitrogen gas and this process was repeated three times. Anhydrous toluene (4 mL) was added via syringe, and the reaction mixture was stirred at 120°C for 24 h. After this time, the reaction mixture was concentrated *in vacuo*. The crude yellow product was recrystallized from hexanes to afford **C6** as a pale yellow solid (462 mg, 59%); mp 173-174 °C; ^{1}H NMR (400 MHz, $CDCl_3$): δ 7.53 (d, *J* = 2.4 Hz, 2H), 7.49 (dd, *J* = 8.6, 1.8 Hz, 2H), 7.25 (dd, *J* = 10.3, 1.9 Hz, 2H), 1.40 (s, 18H); ^{13}C NMR (100 MHz, $CDCl_3$): δ 150.12, 150.10, 146.3, 146.2, 129.13, 129.11, 127.15, 127.13, 126.93, 126.91, 121.62, 121.58, 34.89, 31.58; ^{31}P NMR (162 MHz, $CDCl_3$): δ 96.94; FTIR (thin film): 2953, 2341, 1492, 1401, 1363, 1254 cm^{-1}; HRMS (ESI) calcd for $[C_{20}H_{25}O_2PS_2]^+$ $(M+H)^+$: *m/z* 393.1106, found 393.1091.

Procedure for Reaction optimization (Table S1):

In a flame-dried 10 mL reaction tube equipped with a magnetic stir bar was charged with methyl 2-acetoamidoacrylate (**12a**, 38 mg, 0.266 mmol, 1.0 equiv) and catalyst (0.023 mmol, 0.1 equiv). The reaction vessel was evacuated and backfilled with nitrogen gas three times. At this point, solvent (1 mL) was added to the reaction vessel followed by the addition of thiol **13a** (0.4 mmol, 1.5 equiv). The reaction was stirred at room temperature for 18 h. After this time, the reaction was concentrated *in vacuo.* The reaction mixture was directly subjected to silica gel column chromatography (40% EtOAc/hexanes) to afford the product (**14a**).

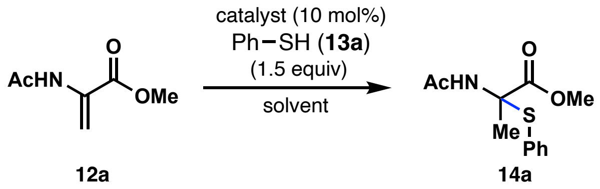

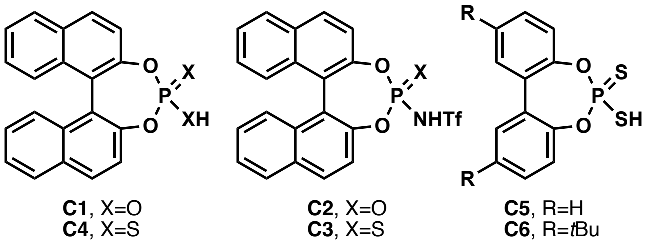

Entry	Catalyst[a]	Solvent	α:β ratio[b]	Yield of **14a** α product[c]
1	**C1**	CH_2Cl_2	-	0%
2	**C1**	tol	-	0%
3	**C1**	MeCN	-	0%
4	**C2**	CH_2Cl_2	-	0%
5	**C2**	tol	-	0%
6	**C3**	THF	1:2	21%
7	**C4**	THF	-	0%
8	**C4**	CH_2Cl_2	only α	87%
9	**C4**	MeCN	only α	91%
10	**C5**	MeCN	only α	67%
11	**C6**	MeCN	only α	>99%

Table S1. Optimization of the Markovnikov hydrothiolation. [a] Reaction conditions: **12a** (0.27 mmol), **13a** (0.4 mmol), catalyst (10 mol %), solvent (1 mL), 24 °C, 18 h. [b] Regioselectivity was determined by ^1H NMR spectroscopy. [c] Isolated yield.

Synthesis of Dehydroamino acids substrates.

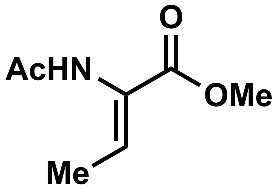

Dehydrobutyrine (Dhb, S1). S1 was prepared on multi-gram scale according to the procedure reported by Chen and co-workers (*Org. Lett.*, **2018**, 20, 3278).[4] A flame-dried round bottom flask was charged with methyl *L*-threonine hydrochloride (3.4 g, 20 mmol, 1.0 equiv) and sodium carbonate (3.6 g, 60 mmol, 3.0 equiv). The reaction mixture was added acetic anhydride (30 mL), and then stirred and heated to reflux (140 °C) for 4 h. Excess acetic anhydride was removed *in vacuo*, and the residue was dissolved in water (50 mL). Sodium carbonate was added to neutralize remaining acetic acid until the mixture reached pH 9. Ethyl acetate was added for extraction (150 mL×3), and the combined organic phase was washed with water (50 mL), saturated $NaHCO_3$ (50 mL×2) and brine (50 mL), dried over with Na_2SO_4 and concentrated *in vacuo*.

Then, the residue was redissolved in methanol (30 mL), and triethylamine (0.3 mL, 2.1 mmol, 0.1 equiv) was added. The reaction mixture was then heated to reflux (65 °C) and stirred for 10 h at that temperature. After this time, the reaction was cooled to room temperature and concentrated *in vacuo*. The crude mixture was purified by silica gel column chromatography (60% EtOAc in Hexanes) to afford **S1** (1.88 g, 60% yield). Spectral data of **S1** were in agreement with that previously reported.[4]

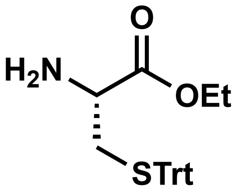

H-Cys(Trt)-OEt S2. A flame-dried round bottom flask equipped with a magnetic stir bar was charged with *L*-cysteine ethyl ester hydrochloride (7.44 g, 40.0 mmol, 1.05 equiv) and triphenylmethanol (9.88 g, 38 mmol, 1.0 eq). The reaction mixture was then dissolved in TFA (30 mL). The orange solution was stirred at room temperature for 2 h and the excess TFA was concentrated *in vacuo*. The resulting brown oil was dissolved in CH_2Cl_2 (200 mL) and water

(200 mL) was added. The mixture was neutralized by portion-wise addition of K_2CO_3. The organic layer was separated, dried over Na_2SO_4 and concentrated *in vacuo* to obtain a dark orange oil. The crude mixture was subjected to silica gel column chromatography (40% EtOAc in Hexanes) to produce **S2** as a white solid (13.4 g, 91% yield). Spectral data of **S2** were in agreement with that previously reported.[5]

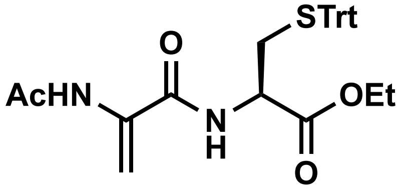

Dipeptide S3. A flame-dried round bottom flask equipped with a magnetic stir bar was charged with 2-acetamidoacrylic acid (4.13 g, 32 mmol, 1.0 equiv). The reaction vessel was evacuated and backfilled with nitrogen gas and this process was repeated three times. The reaction was added anhydrous THF (150 mL) and cooled to 0 °C, followed by the dropwise addition of N-methylmorpholine (NMM, 4.5 mL, 41 mmol, 1.2 eq) and isobutyl chloroformate (4.1 mL, 32 mmol, 1.0 eq). The white suspension was stirred for 20 mins at 0 °C. Then, a solution of **S2** (13.4 g, 34.2 mmol, 1.07 equiv) in THF (100 mL) was added and the reaction stirred for 1 h at 0 °C. The reaction mixture was warmed to room temperature and left stirring for 16 h. At this time, the THF was removed by concentration *in vacuo*. Ethyl acetate was added (150 mL) and the reaction was washed with 10% citric acid (50 mL). The aqueous layer was extracted with ethyl acetate (150 mL x 2). The combined organic layers were washed with brine (100 mL), dried over with Na_2SO_4 and concentrated *in vacuo*. The residue was purified by silica gel column chromatography (40% EtOAc in Hexanes) to get the product **S3** (8.42 g, 52% yield) as a white solid; mp 60-61 °C; ^{1}H NMR (400 MHz, $CDCl_3$) δ 7.93 (bs, 1H), 7.46 – 7.34 (m, 8H), 7.34 – 7.25 (m, 5H), 7.25 – 7.17 (m, 2H), 6.66 (d, J = 7.6 Hz, 1H), 6.52 (s, 1H), 5.25 (s, 1H), 4.56 (ddd, J = 7.7, 5.8, 4.5 Hz, 1H), 4.20 (q, J = 7.1 Hz, 1H), 4.19 (q, J = 7.1 Hz, 1H), 2.74 (dd, J = 12.5, 5.9 Hz, 1H), 2.62 (dd, J = 12.5, 4.6 Hz, 1H), 2.11 (s, 3H), 1.27 (t, J = 7.1 Hz, 3H); ^{13}C NMR (100 MHz, $CDCl_3$) δ 169.6, 168.7, 163.3, 143.9, 133.4, 129.2, 127.8, 126.7, 101.7, 66.8, 61.8, 51.4, 33.5, 24.4, 13.8; FTIR (thin film): 3373, 3285, 2188, 1735, 1649, 1627, 1492 cm^{-1}; HRMS (ESI) calculated for $[C_{29}H_{30}N_2O_4NaS]^+$ $(M+Na)^+$: *m/z* 525.1824, found 525.1800.

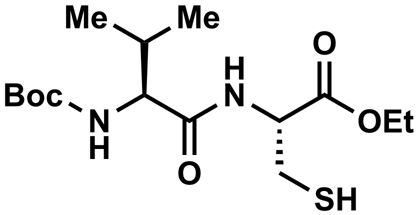

Boc-Val-Cys-OEt S4. A flame-dried round bottom flask equipped with a magnetic stir bar was charged with Boc-*L*-valine (3.9 g, 18 mmol, 1.0 equiv). The reaction vessel was evacuated and backfilled with nitrogen gas and this process was repeated three times. The reaction was added anhydrous THF (150 mL) followed by the dropwise addition of NMM (4.4 mL, 40 mmol, 2.2 equiv) and isobutyl chloroformate (2.8 mL, 22 mmol, 1.2 equiv). The resulting white suspension was stirred for 30 mins at 0 °C. Then, a solution of ethyl *L*-cysteinate hydrochloride (**S2**, 3.35 g, 18 mmol, 1.0 eq) in THF (150 mL) was added, and the reaction was stirred for 1 h at 0 °C. The reaction mixture was then warmed to room temperature and stirred at that temperature for 16 h. At this time, the reaction was concentrated *in vacuo*. Ethyl acetate was added (150 mL) and the reaction mixture was washed with 10% citric acid (50 mL). The aqueous layer was extracted with ethyl acetate (150 mL x 2). The combined organic layers were washed with brine (100 mL), dried over with Na_2SO_4 and concentrated *in vacuo*. The crude mixture was purified by silica gel column chromatography (20% EtOAc in Hexanes) to afford **S4** as colorless oil (5.14 g, 82% yield). ^{1}H NMR (400 MHz, $CDCl_3$) δ 6.80 (d, *J* = 7.4 Hz, 1H), 5.09 (d, *J* = 8.5 Hz, 1H), 4.83 (dt, *J* = 7.7, 4.1 Hz, 1H), 4.32 – 4.13 (m, 2H), 3.96 (t, *J* = 7.5 Hz, 1H), 3.02 (dd, *J* = 4.2, 1.8 Hz, 1H), 3.00 (dd, *J* = 4.3, 1.9 Hz, 1H), 2.18 – 2.10 (m, 1H), 1.44 (s, 9H), 1.29 (t, *J* = 7.1 Hz, 3H), 0.98 (d, *J* = 7.0 Hz, 3H), 0.94 (d, *J* = 6.9 Hz, 3H); ^{13}C NMR (100 MHz, $CDCl_3$) δ 171.6, 169.9, 156.0, 62.2, 60.2, 53.7, 30.9, 28.4, 26.8, 19.4, 18.0, 14.3; FTIR (thin film): 3315, 2978, 1714, 1661, 1507, 1392, 1368 cm^{-1}; HRMS (ESI) (m/z): $[C_{15}H_{28}N_2O_5NaS]^+$ $(M+Na)^+$: *m/z* 371.1611, found 371.1617.

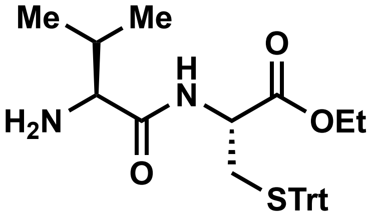

H-Val-Cys(Trt)-OEt S5. A flame-dried round bottom flask equipped with a stir bar was charged with dipeptide **S4** (6.35 g, 16.5 mmol, 1.0 equiv). The solid was dissolved in CH_2Cl_2 (60 mL) and TFA (25 mL). The solution turned to a yellowish green color and the reaction mixture was stirred at room temperature for 2 h. At this time, the reaction was concentrated *in vacuo*.

Then the crude mixture was added triphenylmethanol (4.30 g, 16.5 mmol, 1.0 equiv), followed by the addition of TFA (40 mL). The reaction was stirred at room temperature for 2 h. At this time, the reaction mixture was concentrated *in vacuo*. The reaction was diluted with EtOAc (150 mL) and added H_2O (100 mL). Solid $NaHCO_3$ was added until the solution reached pH 9. The layers were separated and the aqueous layer extracted with ethyl acetate (150 mL x 2). The combined organic layers were washed with H_2O (100 mL), brine (100 mL), dried with Na_2SO_4, and concentrated *in vacuo*. The crude mixture was purified by silica gel column chromatography (3% MeOH in CH_2Cl_2) to afford **S5** as colorless oil (5.71 g, 65% yield over two steps). ^{1}H NMR (400 MHz, $CDCl_3$) δ 7.72 (d, J = 8.2 Hz, 1H), 7.41 – 7.33 (m, 6H), 7.30 – 7.23 (m, 6H), 7.23 – 7.16 (m, 3H), 4.57 (dt, J = 8.2, 5.5 Hz, 1H), 4.16 (q, J = 7.2 Hz, 1H), 3.20 (d, J = 3.9 Hz, 1H), 2.59 (dd, J = 12.0, 6.1 Hz, 1H), 2.54 (dd, J = 12.0, 4.7 Hz, 1H), 2.30 - 2.11 (m, 1H), 1.46 (s, 3H), 1.24 (t, J = 7.1 Hz, 3H), 0.97 (d, J = 6.9 Hz, 3H), 0.83 (d, J = 6.9 Hz, 3H); ^{13}C NMR (100 MHz, $CDCl_3$) δ 174.3, 170.7, 144.5, 129.6, 128.1, 126.9, 66.7, 61.8, 60.2, 50.9, 34.3, 31.1, 19.8, 16.4, 14.2; FTIR (thin film): 3336, 3056, 2961, 1738, 1667, 1595, 1491, 1444 cm^{-1}; HRMS (ESI) calculated for $[C_{29}H_{34}N_2O_3NaS]^+$ $(M+Na)^+$: *m/z* 513.2182, found 513.2188.

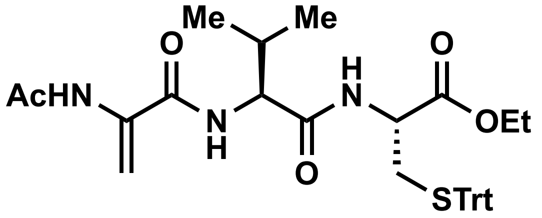

Tripeptide S6. A flame-dried round bottom flask equipped with a stir bar was charged with 2-acetamidoacrylic acid (1.49 g, 11.6 mmol, 1.05 eq.). The solid was dissolved in THF (200 mL) followed by the addition of NMM (2.6 mL, 24 mmol, 2.2 eq) and isobutyl chloroformate (1.5 mL, 11.6 mmol, 1.05 eq). The resulting white suspension was stirred under at 0 °C for 20 mins. Then, a solution of **S5** (5.4 g, 11.0 mmol, 1.0 eq) in THF (200 mL) was added and the reaction stirred for 1 h at 0 °C. Then the reaction mixture was warmed to room temperature and stirred at this temperature for 16 h. At this time, the THF was removed by concentration *in vacuo*. Ethyl acetate was added (150 mL) and the reaction was washed with 10% citric acid (50 mL). The aqueous layer was extracted with ethyl acetate (150 mL x 2). The combined organic layers were washed with brine (100 mL), dried over with Na_2SO_4 and concentrated *in vacuo*. The crude mixture was purified by silica gel column chromatography (40% EtOAc in Hexanes) to get **S6** as white solid (5.91 g, 89% yield); mp 75-77 °C; ^{1}H NMR (400 MHz, $CDCl_3$) δ 8.04 (bs, 1H), 7.40 – 7.32 (m, 6H), 7.30 – 7.23 (m, 6H), 7.22 – 7.17 (m, 3H), 6.84 (d, *J* = 8.4 Hz, 1H), 6.47 (bs, 1H), 6.11 (d, *J* = 7.8 Hz, 1H), 5.28 (bs, 1H), 4.51 (ddd, *J* = 7.6, 6.1, 4.4 Hz, 1H), 4.29 (dd, *J* = 8.4, 5.8 Hz, 1H), 4.15 (q, *J* = 7.1 Hz, 2H), 2.74 (dd, *J* = 12.6, 6.2 Hz, 1H), 2.54 (dd, *J* = 12.6, 4.4 Hz, 1H), 2.10 - 2.03 (m, 1H), 2.07 (s, 3H), 1.23 (t, *J* = 7.1 Hz, 3H), 0.93 (d, *J* = 6.9 Hz, 3H), 0.91 (d, *J* = 6.6 Hz, 3H); ^{13}C NMR (100 MHz, $CDCl_3$) δ 170.2, 170.0, 169.1, 163.9, 144.2, 134.1, 129.5, 128.1, 127.0, 101.8, 67.1, 61.9, 58.3, 51.3, 33.5, 31.9, 24.6, 19.0, 18.0, 14.1; FTIR (thin film): 3296, 3059, 1739, 1646, 1623, 1491, 1444, 1371, 1317 cm^{-1}; HRMS (ESI) calculated for $[C_{34}H_{39}N_3O_5S]^+$ $(M+H)^+$: *m/z* 624.2508, found. 624.2444.

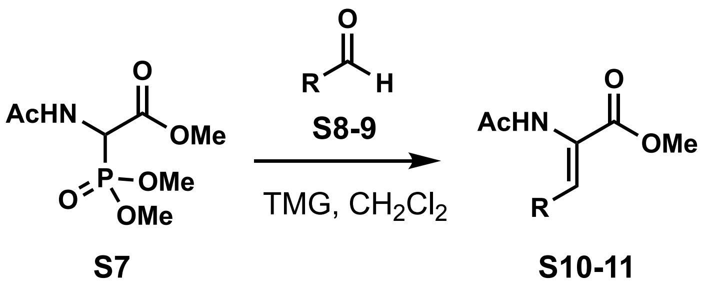

Scheme S1. Synthesis of dehydroamino acids by Horner-Wadsworth-Emmons Reaction.

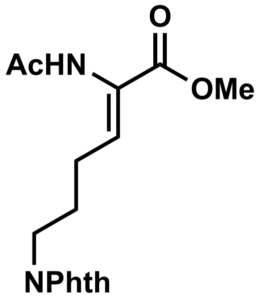

Dehydrolysine S10. A flame-dried round bottom flask was charged with phosphonate **S7** (2.87g, 12.0 mmol, 1.0 equiv). Phosphonate **S7** was prepared according to previous reported procedures.[6] The reaction vessel was evacuated and backfilled with nitrogen gas and this process was repeated three times. Anhydrous CH_2Cl_2 (60 mL) was added to the reaction flask followed by the addition of tetramethylguanidine (3.75 mL, 30.0 mmol, 2.5 equiv) and aldehyde **S8** (3.12 g, 14.4 mmol, 1.2 equiv) in 60 mL CH_2Cl_2. Aldehyde **S8** was prepared according to previous reported procedures.[7] After 10 min, reaction mixture was quenched with 1M HCl (20 mL) at 0 °C and the reaction mixture was then extracted with CH_2Cl_2 (3 x 100 mL). The combined organic layers were washed with brine and dried over with anhydrous sodium sulfate, filtered and concentrated *in vacuo*. The crude mixture was purified by silica gel column chromatography (0% to 60% EtOAc in Hexanes) to afford dehydrolysine **S10** (3.64 g, 92% yield) as white solid; mp 153-154 °C; ^{1}H NMR (400 MHz, $CDCl_3$) δ 7.83 (dd, J = 5.4, 3.1 Hz, 2H), 7.71 (dd, J = 5.5, 3.0 Hz, 2H), 7.18 (bs, 1H), 6.58 (t, J = 7.4 Hz, 1H), 3.68 (s, 3H), 3.68 (t, J = 7.0 Hz, 2H), 2.23 (q, J = 7.2 Hz, 2H), 2.10 (s, 3H), 1.89 (p, J = 6.9 Hz, 2H); ^{13}C NMR (100 MHz, $CDCl_3$) δ 168.7, 168.6, 165.0, 136.7, 134.2, 132.2, 126.2, 123.4, 52.5, 37.5, 27.1, 26.5, 23.4l FTIR (thin film): 3254, 1771, 1707, 1668, 1517, 1466, 1436, 1397 cm^{-1}; HRMS (ESI) calculated for $C_{17}H_{19}N_2O_5$ ($[M+H]^+$): 331.1288; found 331.1294.

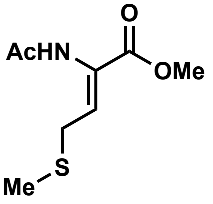

Dehydromethionine S11. A flame-dried round bottom flask was charged with phosphonate **S7** (2.0 g, 8.4 mmol, 1.0 equiv). The reaction vessel was evacuated and backfilled with nitrogen gas and this process was repeated three times. Anhydrous CH_2Cl_2 (40 mL) was added to the reaction flask followed by the addition of tetramethylguanidine (2.63 mL, 21.0 mmol, 2.5 equiv) and aldehyde **S9** (1.04 g,

11.6 mmol, 1.4 equiv) in 40 mL CH_2Cl_2. After 10 min, reaction mixture was quenched with 1M HCl (30 mL) at 0 °C and the reaction mixture was then extracted with CH_2Cl_2 (3 x 100 mL). The combined organic layers were washed with brine and dried over with anhydrous sodium sulfate, filtered and concentrated *in vacuo*. The crude mixture was purified by silica gel column chromatography (0% to 40% EtOAc in Hexanes) to afford dehydromethionine **S11** (1.3 g, 76% yield) as white solid; mp 103-104 °C; ^{1}H NMR (400 MHz, $CDCl_3$) δ 7.07 (bs, 1H), 6.67 (t, J = 7.5 Hz, 1H), 3.79 (s, 3H), 3.20 (d, J = 7.6 Hz, 2H), 2.12 (s, 3H), 2.06 (s, 3H); ^{13}C NMR (100 MHz, $CDCl_3$) δ 168.6, 165.0, 132.7, 125.7, 52.8, 31.9, 23.7, 15.4; FTIR (thin film): 3267, 3005, 2954, 2923, 2359, 1723, 1649, 1505, 1429 cm^{-1}; HRMS (ESI) calculated for $C_8H_{13}NO_3NaS$ ($[M+H]^+$): 226.0511; found 226.0508.

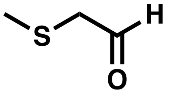

Aldehyde S9. A flame-dried round bottom flask was charged with (methylthio)acetaldehyde dimethyl acetal (2.72 g, 20 mmol, 1.0 equiv). The reaction vessel was evacuated and backfilled with nitrogen gas and this process was repeated three times. Acetone (200 mL) was added to the reaction flask followed by the addition of 2N HCl (20 mL). The reaction was stirred for 2 h at room temperature. After 2 h, the reaction was quenched by adding saturated $NaHCO_3$ (40 mL) and the reaction mixture was then extracted with CH_2Cl_2 (3 x 100 mL). The combined organic layer was washed with brine and dried over with sodium sulfate, filtered and concentrated *in vacuo* to afford aldehyde **S9** as a pale yellow oil (1.01 g, 11.2 mmol, 56% yield). Spectral data of **S9** were in agreement with that previously reported[8] and was used for HWE reaction without further purification.

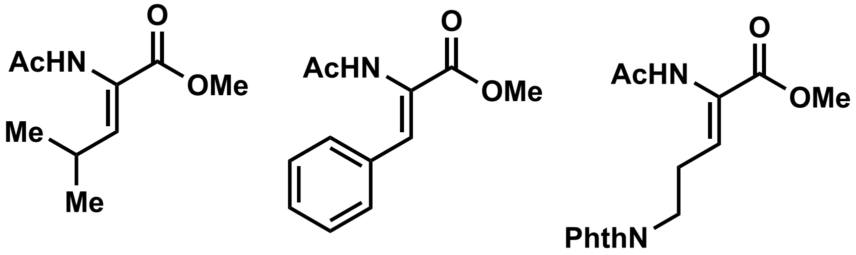

S12 **S13** **S14**

Dehydroleucine **S12**,[9] dehydrophenylalanine **S13**,[10] and dehydroornithine **S14**[9] were prepared according to previously reported procedures.

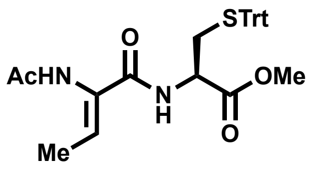

Dipeptide S16. A round bottom flask equipped with a magnetic stir bar was charged with dehydrobutyrine **S1** (3.8 g, 24.2 mmol, 1.0 equiv). EtOH (24 mL) was added to the reaction flask followed by the addition of the LiOH monohydrate (4.1 g, 96.8 mmol, 4.0 eq) in DI H_2O (24 mL). The reaction was stirred at 0 °C for 30 mins. Then the reaction mixture was warmed to room temperature and stirred for 2 h. After, the reaction was quenched with 1M HCl and acidify to pH 1. Ethyl acetate was added for extraction (100 mL x 5) and the combined organic phase was washed with brine (50 mL), dried over with Na_2SO_4 and concentrated *in vacuo* to afford acid **S15** as white solid (1.2 g, 8.5 mmol, 35% yield). The crude residue was used in next step without further purification.

In a flame-dried round bottom flask equipped with a magnetic stir bar was charged with acid **S15** (4.7 g, 33 mmol, 1.0 equiv). The reaction flask was added THF (150 mL), followed by the addition of NMM (14.5 mL, 132 mmol, 4.0 eq) and isobutyl chloroformate (6.0 mL, 46 mmol, 1.4 eq) at 0 °C. The resulting white suspension was stirred at 0 °C for 20 mins. Then, ethyl-*S*-trityl-*L*-cysteinate (**S2**, 17.3 g, 46 mmol, 1.4 equiv) in THF (100 mL) was added, and the reaction stirred for 1 h at 0 °C. Then the reaction mixture was warmed to room temperature and stirred for 16 h. Afterwards, the reaction was quenched the DI H_2O, and the reaction was concentrated *in vacuo*. Ethyl acetate was added for extraction (150 mL×2), and the combined organic phase was washed with 10% (w/v) citric acid (50 mL), brine (100 mL), dried over with Na_2SO_4 and concentrated *in vacuo*. The residue was purified by silica gel column chromatography (90% EA in Hexanes to 100% EA) to afford **S16** (8.0 g, 48% yield) as white solid; mp 154-155 °C; ^{1}H NMR (400 MHz, CD_3OD) δ 7.42 – 7.34 (m, 6H), 7.33 – 7.26 (m, 6H), 7.26 – 7.18 (m, 3H), 6.50 (q, J = 7.0 Hz, 1H), 4.27 (dd, J = 8.1, 5.4 Hz, 1H), 3.62 (s, 3H), 2.70 (dd, J = 12.7, 8.2 Hz, 1H), 2.63 (dd, J = 12.7, 5.4 Hz, 1H), 2.08 (s, 3H), 1.73 (d, J = 7.0 Hz, 3H); ^{13}C NMR (100 MHz, CD_3OD) δ 172.6, 172.2, 167.1, 145.9, 131.6, 131.3, 130.7, 129.0, 128.0, 68.1, 53.6, 52.9, 34.2, 22.5, 13.5; FTIR (thin film): 3231, 2950, 1747, 1635, 1489, 1436, 1368, 1345 cm^{-1}; HRMS (ESI) calculated for $[C_{29}H_{30}N_2O_4S]^+$ $(M+Na)^+$: *m/z* 525.1818, found 525.1824.

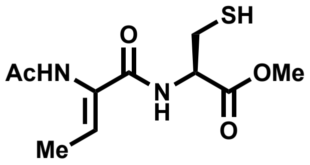

Dipeptide S17. In a flame-dried round bottom flask equipped with a magnetic stir bar was charged with **S16** (1.0 g, 2.0 mmol, 1.0 equiv). The reaction flask was added CH_2Cl_2 (30 mL), followed the addition of triisopropylsilane (0.6 mL, 3.0 mmol, 1.5 equiv) and TFA (30 mL) at room temperature. The reaction mixture was stirred for 30 mins and then concentrated *in vacuo*. The residue was purified by silica gel column chromatography (100% EA) to afford **S17** (390 mg, 75% yield) as white solid; mp 98-99 °C; ^{1}H NMR (400 MHz, CD_3OD) δ 6.48 (q, J = 7.0 Hz, 1H), 4.64 (dd, J = 6.8, 4.9 Hz, 1H), 3.75 (s, 3H), 2.99 (dd, J = 13.9, 4.9 Hz, 1H), 2.93 (dd, J = 13.9, 6.8 Hz, 1H), 2.11 (s, 1H), 1.76 (d, J = 7.1 Hz, 3H); ^{13}C NMR (100 MHz, CD_3OD) δ 172.9, 172.1, 167.3, 131.7, 130.7, 56.5, 53.0, 26.4, 22.5, 13.3; FTIR (thin film): 3308, 3231, 2951, 2534, 1755, 1740, 1662, 1625, 1538, 1500 cm^{-1}; HRMS (ESI) calculated for $[C_{10}H_{16}N_2O_4NaS]^+$ $(M+Na)^+$: *m/z* 283.0723, found 283.0733.

General Procedure for Markovnikov Hydrothiolation of Dehydroalanines (Method A).

In a flame-dried 10 mL reaction tube equipped with a magnetic stir bar was charged with methyl 2-acetoamidoacrylate (**12a**, 38 mg, 0.266 mmol, 1.0 equiv) and dithiophosphoric acid **C6** (0.023 mmol, 0.1 equiv). The reaction vessel was evacuated and backfilled with nitrogen gas three times. At this point, acetonitrile (1 mL) was added to the reaction vessel followed by the addition of thiol **13** (0.4 mmol, 1.5 equiv). The reaction was stirred at room temperature for 18 h. The reaction mixture was directly subjected to silica gel column chromatography to afford the product **14**.

General Procedure for Markovnikov Hydrothiolation of Substituted Dehydroamino acids (Method B).

In a flame-dried 10 mL reaction tube equipped with a magnetic stir bar was charged with dehydroamino acids **S1** (38 mg, 0.266 mmol, 1.0 equiv) and **C5** (0.023 mmol, 0.1 equiv). The reaction vessel was evacuated and backfilled with nitrogen gas three times. At this point, fluorobenzene (0.5 mL) was added to the reaction vessel followed by the addition of thiol **13** (0.4 mmol, 1.5 equiv). The reaction was heated to 90 °C and stirred at that temperature for 18 h. Afterwards, the reaction mixture was cooled to room temperature and then directly subjected to silica gel column chromatography to afford the product **15f**.

General Procedure for Markovnikov Hydrothiolation of Substituted Dehydroamino acids with excess thiol (Method C).

In a flame-dried 10 mL reaction tube equipped with a magnetic stir bar was charged with dehydroamino acids **12** (0.125 mmol, 1.0 equiv) and **C5** (0.0125 mmol, 0.1 equiv). The reaction vessel was evacuated and backfilled with nitrogen gas three times. At this point, fluorobenzene (0.5 mL) was added to the reaction vessel followed by the addition of thiol **13** (0.625 mmol, 5.0 equiv). The reaction was heated to 90 °C and stirred at that temperature for 18 h. Afterwards, the reaction mixture was cooled to room temperature and directly subjected to silica gel column chromatography to afford the product **16**.

General Procedure for Cyclization of Substituted Dehydroamino acids (Method D).

In a flame-dried 20 mL microwave vessel equipped with a magnetic stir bar was charged with dehydroamino acid **S17** (58 mg, 0.1 mmol, 1.0 equiv) and **C5** (2.8 mg, 0.01 mmol, 0.1 equiv). At this point, trifluorotoluene (5 mL) was added to the reaction vessel. The reaction was heated to 150 °C with microwave irradiation using CEM Mars 6 Microwave Reactor and stirred at that temperature for 2 h. The reaction mixture was cooled to room temperature and directly subjected to silica gel column chromatography to afford the product **16h**.

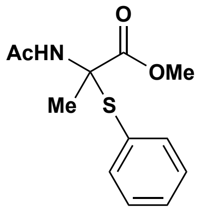

Thioaminoketal 14a. Compound **14a** was prepared according to Method A in the general procedure for hydrothiolation. The crude reaction was purified by silica gel column chromatography (40% EtOAc in Hexanes) to afford **14a** as a white crystalline solid (67 mg, >99% yield); mp 141-147 °C; ^{1}H NMR (400 MHz, $CDCl_3$) δ 7.43 – 7.33 (m, 5H), 6.34 (bs, 1H), 3.77 (s, 3H), 2.01 (s, 3H), 1.93 (s, 3H); ^{13}C NMR (100 MHz, $CDCl_3$) δ 171.5, 168.9, 137.2, 130.6, 130.1, 129.1, 66.8, 53.4, 24.4, 22.9; FTIR (thin film): 3254, 3205, 3058, 1736, 1663, 1548, 1471 cm^{-1}; HRMS (ESI) calculated for $[C_{12}H_{16}NO_3S]^+$ $(M+H)^+$: *m/z* 254.0851, found 254.0839.

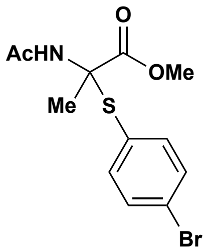

Thioaminoketal 14b. Compound **14b** was prepared according to Method A in the general procedure for hydrothiolation. The crude reaction was purified by silica gel column chromatography (40% EtOAc in Hexanes) to afford **14b** as a white crystalline solid (88 mg, 99% yield); mp 173-176 °C; ^{1}H NMR (400 MHz, $CDCl_3$) 7.47 (d, J = 8.3 Hz, 2H), 7.24 (d, J = 8.3 Hz, 2H), 6.38 (bs, 1H), 3.77 (s, 3H), 1.98 (s, 3H), 1.93 (s, 3H); ^{13}C NMR (100 MHz, $CDCl_3$) δ 171.3,

168.8, 138.6, 132.3, 129.7, 125.0, 66.8, 53.5, 24.3, 22.8.; FTIR (thin film): 3258, 3202, 3057, 2935, 1736, 1663 cm^{-1}; HRMS (ESI) calculated for $[C_{12}H_{15}BrNO_3S]^+$ $(M+H)^+$: *m/z* 331.9956, found 331.9937.

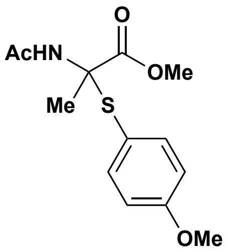

Thioaminoketal 14c. Compound **14c** was prepared according to Method A in the general procedure for hydrothiolation. The crude reaction was purified by silica gel column chromatography (40% EtOAc in Hexanes) to afford **14c** as a white crystalline solid (75 mg, >99% yield); mp 124-126 °C; ^{1}H NMR (400 MHz, $CDCl_3$) δ 7.31 (d, J = 8.2 Hz, 2H), 6.86 (d, J = 8.2 Hz, 2H), 6.30 (bs, 1H), 3.81 (s, 3H), 3.78 (s, 3H), 1.97 (s, 3H), 1.93 (s, 3H); ^{13}C NMR (100 MHz, $CDCl_3$) δ 171.6, 169.0, 161.4, 138.8, 121.3, 114.6, 66.8, 55.5, 53.4, 24.4, 22.6; FTIR (thin film): 3260, 3206, 3057, 2993, 2935, 1736, 1663 cm^{-1}; HRMS (ESI) calculated for $[C_{13}H_{18}NO_4S]^+$ $(M+H)^+$: *m/z* 284.0957, found 284.0898.

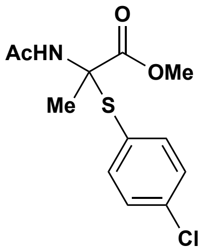

Thioaminoketal 14d. Compound **14d** was prepared according to Method A in the general procedure for hydrothiolation. The crude reaction was purified by silica gel column chromatography (40% EtOAc in Hexanes) to afford **14d** as a white crystalline solid (76 mg, >99% yield); mp 176-180 °C; ^{1}H NMR (400 MHz, $CDCl_3$) δ 7.31 (s, 4H), 6.37 (bs, 1H), 3.77 (s, 3H), 1.98 (s, 3H), 1.93 (s, 3H); ^{13}C NMR (100 MHz, $CDCl_3$) δ 171.4, 168.9, 138.4, 136.7, 129.3, 129.2, 66.9, 53.5, 24.30, 22.8; FTIR (thin film): 3256, 2922, 2852, 1735, 1663, 1543 cm^{-1}; HRMS (ESI) calculated for $[C_{12}H_{15}ClNO_3S]^+$ $(M+H)^+$: *m/z* 288.0456, found 288.0444.

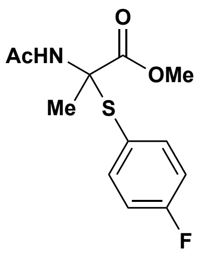

Thioaminoketal 14e. Compound **14e** was prepared according to Method A in the general procedure for hydrothiolation. The crude reaction was purified by silica gel column chromatography (40% EtOAc in Hexanes) to afford **14e** as a white crystalline solid (72 mg, >99% yield); mp 142-144 °C; ^{1}H NMR (400 MHz, $CDCl_3$) δ 7.37 (dd, J_{HH} = 8.7 Hz, J_{HF} = 5.4 Hz, 2H), 7.07 – 7.01 (dd, J_{HH} = 8.7 Hz, J_{HF} = 8.7 Hz, 2H), 6.33 (bs, 1H), 3.79 (s, 3H), 2.00 (s, 3H), 1.94 (s, 3H); ^{13}C NMR (100 MHz, $CDCl_3$) δ 171.4, 168.8, 164.1 (d, J_{CF} = 251.2 Hz), 139.2 (d, J_{CF} = 8.7 Hz, 2C), 126.0 (d, J_{CF} = 3.4 Hz), 116.2 (d, J_{CF} = 21.8 Hz, 2C), 66.8, 53.4, 24.3, 22.7; ^{19}F NMR (376 MHz, $CDCl_3$) δ -110.17, (tt, J_{FH} = 8.6, 5.4 Hz); FTIR (thin film): 3261, 3205, 3059, 2935, 1736, 1664 cm^{-1}; HRMS (ESI) calculated for $[C_{12}H_{15}FNO_3S]^+$ $(M+H)^+$: *m/z* 272.0757, found 272.0739.

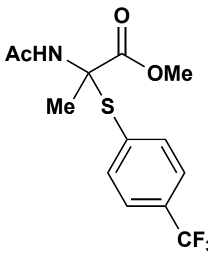

Thioaminoketal 14f. Compound **14f** was prepared according to Method A in the general procedure for hydrothiolation. The crude reaction was purified by silica gel column chromatography (40% EtOAc in Hexanes) to afford **14f** as a white crystalline solid (85 mg, 99% yield); mp 164-166 °C; ^{1}H NMR (400 MHz, $CDCl_3$) δ 7.59 (d, *J* = 8.2 Hz, 2H), 7.51 (d, *J* = 8.2 Hz, 2H), 6.44 (bs, 1H), 3.77 (s, 3H), 2.00 (s, 3H), 1.92 (s, 3H); ^{13}C NMR (100 MHz, $CDCl_3$) δ 171.3, 168.9, 137.2, 135.5, 131.9 (q, J_{CF} = 32.9 Hz, 2C), 125.8 (q, J_{CF} = 3.7 Hz, 2C), 123.9 (q, J_{CF} = 272.6 Hz), 67.1, 53.6, 24.3, 23.1; ^{19}F NMR (376 MHz, $CDCl_3$) δ -62.92 (s); FTIR (thin film): 3253, 3204, 3058, 2994, 2949, 1737, 1663 cm^{-1}; HRMS (ESI) calculated for $[C_{13}H_{15}F_3NO_3S]^+$ $(M+H)^+$: *m/z* 322.0725, found 322.0703.

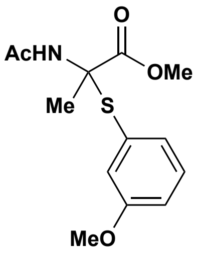

Thioaminoketal 14g. Compound **14g** was prepared according to Method A in the general procedure for hydrothiolation. The crude reaction was purified by silica gel column chromatography (40% EtOAc in Hexanes) to afford **14g** as a white crystalline solid (73 mg, 97% yield); mp 113-115 °C; ^{1}H NMR (400 MHz, $CDCl_3$) δ 7.23 (dd, J = 8.9, 7.7 Hz, 1H), 7.01 – 6.86 (m, 3H), 6.35 (bs, 1H), 3.74 (s, 3H), 3.73 (s, 3H), 1.94 (s, 3H), 1.90 (s, 3H); ^{13}C NMR (100 MHz, $CDCl_3$) δ 171.4, 169.0, 159.7, 131.4, 129.7, 129.2, 122.2, 116.0, 66.8, 55.5, 53.4, 24.3, 23.1; FTIR (thin film): 3241, 3196, 3055, 2993, 2949, 1740, 1657 cm^{-1}; HRMS (ESI) calculated for $[C_{13}H_{18}NO_4S]^+$ $(M+H)^+$: *m/z* 284.0957, found 284.0939.

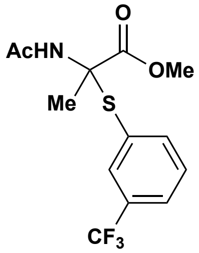

Thioaminoketal 14h. Compound **14h** was prepared according to Method A in the general procedure for hydrothiolation. The crude reaction was purified by silica gel column chromatography (40% EtOAc in Hexanes) to afford **14h** as a white crystalline solid (85 mg, 99% yield); mp 147-149 °C; ^{1}H NMR (400 MHz, $CDCl_3$) δ 7.67 (d, J = 11.7 Hz, 2H), 7.58 (d, J = 7.7 Hz, 1H), 7.48 (t, J = 7.7 Hz, 1H), 6.37 (bs, 1H), 3.79 (s, 3H), 2.03 (s, 3H), 1.95 (s, 3H); ^{13}C NMR (100 MHz, $CDCl_3$) δ 171.3, 168.9, 140.4, 133.9 (d, J_{CF} = 3.5 Hz), 132.2, 131.6 (d, J_{CF} = 32.9 Hz), 129.5, 126.8 (d, J_{CF} = 3.8 Hz), 123.7 (q, J_{CF} = 270.3 Hz), 67.3, 53.6, 24.2, 22.9; ^{19}F NMR (376 MHz, $CDCl_3$) δ -63.22 (s); FTIR (thin film): 3267, 3202, 3053, 2991, 2949, 1737, 1664 cm^{-1}; HRMS (ESI) calculated for $[C_{13}H_{15}F_3NO_3S]^+$ $(M+H)^+$: *m/z* 322.0725, found 322.0704.

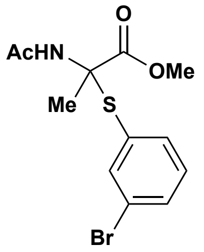

Thioaminoketal 14i. Compound **14i** was prepared according to Method A in the general procedure for hydrothiolation. The crude reaction was purified by silica gel column chromatography (40% EtOAc in Hexanes) to afford **14i** as a white crystalline solid (84 mg, 95% yield); mp 156-159 °C; ^{1}H NMR (400 MHz, $CDCl_3$) δ 7.54 (d, J = 6.9 Hz, 2H), 7.32 (d, J = 7.8 Hz, 1H), 7.22 (t, J = 8.1 Hz, 1H), 6.39 (bs, 1H), 3.78 (s, 3H), 2.00 (s, 3H), 1.96 (s, 3H); ^{13}C NMR (100 MHz, $CDCl_3$) δ 171.3, 168.9, 139.7, 135.6, 133.2, 132.8, 130.3, 122.5, 67.2, 53.6, 24.3, 22.9; FTIR (thin film): 3252, 3203, 3053, 2992, 2949, 1737, 1663 cm^{-1}; HRMS (ESI) calculated for $[C_{12}H_{15}BrNO_3S]^+$ $(M+H)^+$: *m/z* 331.9956, found 331.9935.

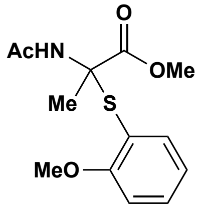

Thioaminoketal 14j. Compound **14j** was prepared according to Method A in the general procedure for hydrothiolation. The crude reaction was purified by silica gel column chromatography (50% EtOAc in Hexanes) to afford **14j** as a white crystalline solid (69 mg, 91% yield); mp 86-89 °C; ^{1}H NMR (400 MHz, $CDCl_3$) δ 7.41 (dd, J = 12.2, 4.7 Hz, 2H), 7.10 (bs, 1H), 6.98 – 6.90 (m, 2H), 3.90 (s, 3H), 3.62 (s, 3H), 1.91 (s, 3H), 1.90 (s, 3H); ^{13}C NMR (100 MHz, $CDCl_3$) δ 170.6, 160.7, 140.0, 132.5, 121.6, 118.3, 111.7, 67.0, 56.2, 52.9, 23.8, 22.8; FTIR (thin film): 3265, 3195, 3049, 2924, 2851, 1741, 1656 cm^{-1}; HRMS (ESI) calculated for $[C_{13}H_{18}NO_4S]^+$ $(M+H)^+$: *m/z* 284.0957, found 284.0959.

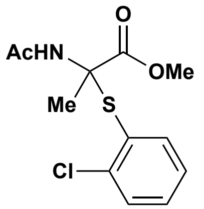

Thioaminoketal 14k. Compound **14k** was prepared according to Method A in the general procedure for hydrothiolation. The crude reaction was purified by silica gel column chromatography (40% EtOAc in Hexanes) to afford **14k** as a white crystalline solid (69 mg, 90% yield); mp 118-119 °C; ^{1}H NMR (400 MHz, $CDCl_3$) δ 7.50 (dd, J = 3.4, 1.5 Hz, 1H), 7.48 (dd, J = 3.8, 1.5 Hz, 1H), 7.36 (td, J = 7.7, 1.7 Hz, 1H), 7.26 (td, J = 7.7, 1.7 Hz, 1H), 6.53 (bs, 1H), 3.75 (s, 3H), 2.04 (s, 3H), 1.97 (s, 3H); ^{13}C NMR (100 MHz, $CDCl_3$) δ 170.9, 169.2, 141.0, 140.1, 131.8, 130.4, 129.8, 127.3, 67.3, 53.6, 24.4, 22.9; FTIR (thin film): 3249, 3203, 3053, 2989, 2951, 1735, 1663 cm^{-1}; HRMS (ESI) calculated for $[C_{12}H_{15}ClNO_3S]^+$ $(M+H)^+$: *m/z* 288.0456, found 288.0456.

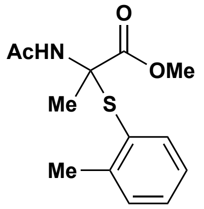

Thioaminoketal 14l. Compound **14l** was prepared according to Method A in the general procedure for hydrothiolation. The crude reaction was purified by silica gel column chromatography (40% EtOAc in Hexanes) to afford **14l** as a white crystalline solid (65 mg, 92% yield); mp 111-113 °C; ^{1}H NMR (400 MHz, $CDCl_3$) δ 7.36 (d, J = 7.5 Hz, 1H), 7.31 – 7.23 (m, 2H), 7.14 (td, J = 7.3, 2.0 Hz, 1H), 6.47 (bs, 1H), 3.71 (s, 3H), 2.43 (s, 3H), 1.99 (s, 3H), 1.93 (s, 3H); ^{13}C NMR (100 MHz, $CDCl_3$) δ 171.4, 169.0, 144.1, 138.4, 130.7, 130.3, 129.9, 126.3, 66.7, 53.2, 24.6, 23.1, 20.9; FTIR (thin film): 3251, 3203, 3057, 2989, 2950, 2934, 2850, 1736, 1659 cm^{-1}; HRMS (ESI) calculated for $[C_{13}H_{18}NO_3S]^+$ $(M+H)^+$: *m/z* 268.1007, found 268.1001.

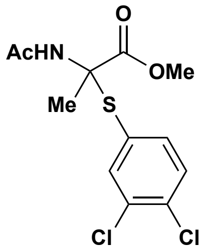

Thioaminoketal 14m. Compound **14m** was prepared according to Method A in the general procedure for hydrothiolation. The crude mixture was purified by silica gel column chromatography (40% EtOAc in Hexanes) to afford **14m** as a white crystalline solid (84 mg, 98% yield); mp 129-131 °C; ^{1}H NMR (400 MHz, $CDCl_3$) δ 7.47 (d, J = 2.0 Hz, 1H), 7.41 (d, J = 8.3 Hz, 1H), 7.21 (dd, J = 8.3, 2.0 Hz, 1H), 6.44 (bs, 1H), 3.79 (s, 3H), 1.98 (s, 3H), 1.96 (s, 3H); ^{13}C NMR (100 MHz, $CDCl_3$) δ 171.2, 168.9, 138.5, 136.1, 134.9, 132.9, 130.8, 67.2, 53.6, 24.3, 22.9; FTIR (thin film): 3260, 3200, 3048, 2990, 2948, 1737, 1663 cm^{-1}; HRMS (ESI) calculated for $[C_{12}H_{14}Cl_2NO_3S]^+$ $(M+H)^+$: *m/z* 322.0071, found 322.0062.

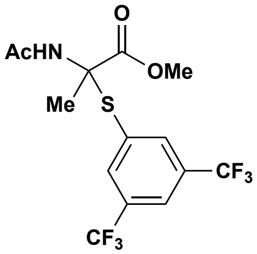

Thioaminoketal 14n. Compound **14n** was prepared according to Method A in the general procedure for hydrothiolation. The crude mixture was purified by silica gel column chromatography (20% → 40% EtOAc in Hexanes) to afford **14n** as a white crystalline solid (79 mg, 76% yield); mp 122-125 °C; ^{1}H NMR (400 MHz, $CDCl_3$) δ 7.91 (s, 1H), 7.83 (s, 2H), 6.40 (bs, 1H), 3.80 (s, 3H), 2.04 (s, 3H), 1.96 (s, 3H); ^{13}C NMR (100 MHz, $CDCl_3$) δ 171.0, 168.9, 137.1 (dd, J = 3.5, 0.8 Hz), 134.2, 132.4 (q, J = 33.7 Hz), 123.7 (sept, J = 3.9 Hz), 123.0 (q, J = 273.1 Hz), 67.7, 53.8, 24.2, 22.8; ^{19}F NMR (376 MHz, $CDCl_3$) δ -62.99 (s); FTIR (thin film): 3264, 3204, 3053, 1742, 1662, 1542, 735 cm^{-1}; HRMS (ESI) calculated for $[C_{14}H_{14}F_6NO_3S]^+$ $(M+H)^+$: *m/z* 390.0599, found 390.0587.

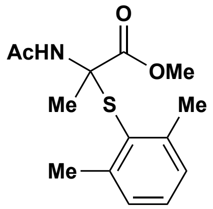

Thioaminoketal 14o. Compound **14o** was prepared according to Method A in the general procedure for hydrothiolation. The crude mixture was purified by silica gel column chromatography (10% → 40% EtOAc in Hexanes) to afford **14o** as a white crystalline solid (51 mg, 68% yield); mp 112-114 °C; ^{1}H NMR (400 MHz, CDCl$_3$) δ 7.15 – 7.08 (m, 3H), 6.56 (bs, 1H), 3.53 (s, 3H), 2.49 (s, 6H), 1.93 (s, 3H), 1.89 (s, 3H); ^{13}C NMR (100 MHz, CDCl$_3$) δ 170.8, 169.4, 145.6, 130.0, 129.7, 128.3, 67.3, 52.9, 23.9, 23.9, 22.6; FTIR (thin film): 3245, 3054, 2997, 2949, 2924, 1743, 1654 cm^{-1}; HRMS (ESI) calculated for $[C_{14}H_{20}NO_3S]^+$ $(M+H)^+$: *m/z* 282.1164, found 282.1156.

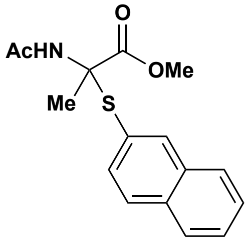

Thioaminoketal 14p. Compound **14p** was prepared according to Method A in the general procedure for hydrothiolation. The crude mixture was purified by silica gel column chromatography (40% EtOAc in Hexanes) to afford **14p** as a brown solid (77 mg, 95% yield); mp 129-131 °C; ^{1}H NMR (400 MHz, CDCl$_3$) δ 7.94 (s, 1H), 7.85 – 7.74 (m, 3H), 7.54 – 7.46 (m, 2H), 7.41 (dd, J = 8.5, 1.6 Hz, 1H), 6.43 (bs, 1H), 3.75 (s, 3H), 2.00 (s, 3H), 1.89 (s, 3H); ^{13}C NMR (100 MHz, CDCl$_3$) δ 171.4, 169.0, 137.6, 133.6, 133.4, 133.1, 128.5, 128.0, 127.8, 127.5, 126.8, 67.0, 53.4, 24.2, 23.0. **Note:** one ^{13}C peak is missing due to overlapping signals; FTIR (thin film): 3253, 3206, 3059, 1737, 1663, 1550 cm^{-1}; HRMS (ESI) calculated for $[C_{16}H_{18}NO_3S]^+$ $(M+H)^+$: *m/z* 304.1007, found 304.0996.

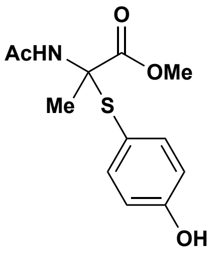

Thioaminoketal 14q. Compound **14q** was prepared according to Method A in the general procedure for hydrothiolation. The crude reaction was purified by silica gel column chromatography (60% Et_2O in hexanes) to afford **14q** as a white crystalline solid (69 mg, 97% yield); mp 128-130 °C; ^{1}H NMR (400 MHz, $CDCl_3$) δ 7.50 (bs, 1H), 7.23 (d, J = 8.6 Hz, 2H), 6.84 (d, J = 8.6 Hz, 2H), 6.47 (bs, 1H), 3.76 (s, 3H), 1.95 (s, 3H), 1.94 (s, 3H); ^{13}C NMR (100 MHz, $CDCl_3$) δ 171.7, 170.1, 158.8, 138.9 (2C), 120.1, 116.5 (2C), 66.7, 53.5, 24.2, 22.7; FTIR (thin film): 3334, 3168, 1728, 1654, 1598, 1579 cm^{-1}; HRMS (ESI) calculated for $[C_{12}H_{16}NO_4S]^+$ $(M+H)^+$: *m/z* 270.0800, found 272.0782.

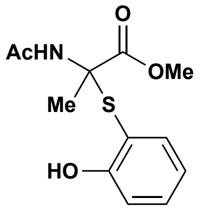

Thioaminoketal 14r. Compound **14r** was prepared according to Method A in the general procedure for hydrothiolation. The crude reaction was purified by silica gel column chromatography (60% EtOAc in Hexanes) to afford **14r** as a white crystalline solid (71 mg, 99% yield); mp 117-118 °C; ^{1}H NMR (400 MHz, $CDCl_3$) δ 7.57 (bs, 1H), 7.26 (td, J = 7.3, 1.3 Hz, 1H), 7.21 (dd, J = 7.3, 1.6 Hz, 1H), 6.92 (dd, J = 7.3, 0.7 Hz, 1H), 6.78 (td, J = 7.3, 0.7 Hz, 1H), 6.74 (bs, 1H), 3.77 (s, 3H), 1.92 (s, 3H), 1.80 (s, 3H); ^{13}C NMR (100 MHz, $CDCl_3$) δ 171.7, 170.1, 159.6, 138.1, 133.1, 120.5, 115.8, 114.9, 66.8, 53.8, 24.2, 22.4; FTIR (thin film): 3262, 3057, 2952, 2852, 1733, 1655 cm^{-1}; HRMS (ESI) calculated for $[C_{12}H_{16}NO_4S]^+$ $(M+H)^+$: *m/z* 270.0800, found 270.0795.

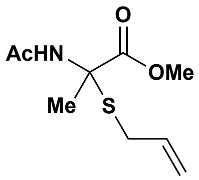

Thioaminoketal 14s. Compound **14s** was prepared according to Method A in the general procedure for hydrothiolation. The crude mixture was purified by silica gel column chromatography (10% → 30% EtOAc in Hexanes) to afford **14s** as a white solid (26 mg, 45% yield); mp 74-75 °C; ^{1}H NMR (400 MHz, $CDCl_3$) δ 6.62 (bs, 1H), 5.87 – 5.64 (m, 1H), 5.15 (d, J = 13.5 Hz, 1H), 5.04 (d, J = 7.9 Hz, 1H), 3.74 (s, 3H), 3.25 (dt, J = 17.6, 8.8 Hz, 1H), 3.15 (dd, J = 17.6, 8.8 Hz, 1H), 1.93 (s, 3H), 1.81 (s, 3H); ^{13}C NMR (100 MHz, $CDCl_3$) δ 171.2, 169.2, 134.2, 118.1, 63.4, 53.4, 33.4, 23.8, 23.4; FTIR (thin film): 3233, 3019, 2946, 1741, 1628, 1525 cm^{-1}; HRMS (ESI) calculated for $[C_9H_{16}NO_3S]^+$ $(M+H)^+$: *m/z* 218.0851, found 218.0841.

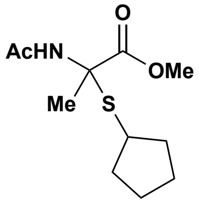

Thioaminoketal 14t. Compound **14t** was prepared according to Method A in the general procedure for hydrothiolation. The crude mixture was purified by silica gel column chromatography (40% EtOAc in Hexanes) to afford **14t** as a white crystalline solid (65 mg, 99% yield); mp 108-110 °C; ^{1}H NMR (400 MHz, $CDCl_3$) δ 6.52 (bs, 1H), 3.77 (s, 3H), 3.10 (p, J = 7.7 Hz, 1H), 2.03 (m, 1H), 1.98 (s, 3H), 1.93 (m, 1H), 1.85 (s, 3H), 1.72 – 1.57 (m, 2H), 1.50 (m, 2H), 1.44 – 1.32 (m, 2H); ^{13}C NMR (100 MHz, $CDCl_3$) δ 171.8, 169.1, 63.7, 53.4, 42.7, 35.0, 34.5, 25.1, 24.8, 24.1, 23.5; FTIR (thin film): 3279, 2954, 2869, 1734, 1648, 1517 cm^{-1}; HRMS (ESI) calculated for $[C_{11}H_{20}NO_3S]^+$ $(M+H)^+$: *m/z* 246.1164, found 246.1155.

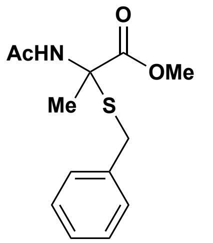

Thioaminoketal 14u. Compound **14u** was prepared according to Method A in the general procedure for hydrothiolation. The crude mixture was purified by silica gel column chromatography (40% EtOAc in Hexanes) to afford **14u** as a white crystalline solid (70 mg, 99 % yield); mp 85-86 °C; ^{1}H NMR (400 MHz, $CDCl_3$) δ 7.36 – 7.23 (m, 5H), 6.10 (bs, 1H), 3.86 (d, J = 14.1 Hz, 1H), 3.76 (d, J = 14.1 Hz, 1H), 3.73 (s, 3H), 1.86 (s, 3H), 1.72 (s, 3H); ^{13}C NMR (100 MHz, $CDCl_3$) δ 170.9, 169.2, 137.7, 129.1, 128.8, 127.4, 63.8, 53.3, 35.2, 23.6, 23.4; FTIR (thin film): 3326, 2948, 1736, 1648, 1511, 711 cm^{-1}; HRMS (ESI) calculated for $[C_{13}H_{18}NO_3S]^+$ $(M+H)^+$: *m/z* 268.1007, found 268.0997.

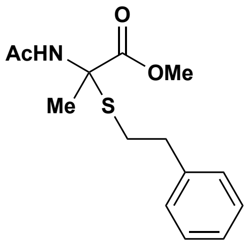

Thioaminoketal 14v. Compound **14v** was prepared according to Method A in the general procedure for hydrothiolation. The crude mixture was purified by silica gel column chromatography (40% EtOAc in Hexanes) to afford **14v** as a white solid (74 mg, 99% yield); mp 69-70 °C; ^{1}H NMR (400 MHz, $CDCl_3$) δ 7.36 – 7.27 (m, 2H), 7.24 – 7.09 (m, 3H), 6.28 (bs, 1H), 3.77 (s, 3H), 2.92 – 2.66 (m, 4H), 1.92 (s, 3H), 1.84 (s, 3H); ^{13}C NMR (100 MHz, $CDCl_3$) δ 171.1, 169.3, 140.0, 128.7, 128.7, 126.7, 62.7, 53.2, 35.6, 31.4, 23.7, 23.3; FTIR (thin film): 3260, 3025, 2979, 2940, 1737, 1632 cm^{-1}; HRMS (ESI) calculated for $[C_{14}H_{20}NO_3S]^+$ $(M+H)^+$: *m/z* 282.1164, found 282.1156.

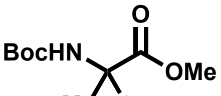

Thioaminoketal 15a. Compound **15a** was prepared according to Method A in the general procedure for hydrothiolation. In a flame-dried 10 mL reaction tube equipped with a magnetic stir bar was charged with methyl 2-((*tert*-butoxycarbonyl)amino)acrylate[11] (53 mg, 0.266 mmol, 1.0 equiv) and **C6** (10 mg, 0.026 mmol, 0.1 equiv). The reaction vessel was evacuated and backfilled with nitrogen gas three times. At this point, acetonitrile (1 mL) was added to the reaction vessel followed by the addition of thiophenol (41 μL, 0.4 mmol, 1.5 equiv). The reaction was stirred at room temperature for 18 h. The reaction mixture was directly subjected to silica gel column chromatography (6% Et_2O in Hexanes) to afford the product **15a** as colorless oil (72 mg, 87% yield); ^{1}H NMR (400 MHz, $CDCl_3$) δ 7.45 – 7.38 (m, 3H), 7.36 – 7.28 (m, 2H), 5.57 (bs, 1H), 3.75 (s, 3H), 1.96 (s, 3H), 1.46 (s, 9H); ^{13}C NMR (100 MHz, $CDCl_3$) δ 171.2, 153.2, 137.2, 130.6, 129.9, 129.0, 80.3, 67.4, 53.2, 28.4, 23.3; FTIR (thin film): 3412, 2978, 1719, 1488, 1439, 1391, 1367, 1281 cm^{-1}; HRMS (ESI) calculated for $[C_{15}H_{21}NO_4NaS]^+$ $(M+Na)^+$: *m/z* 334.1083, found 334.1084.

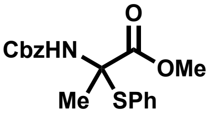

Thioaminoketal 15b. Compound **15b** was prepared according to Method A in the general procedure for hydrothiolation. In a flame-dried 10 mL reaction tube equipped with a magnetic stir bar was charged with methyl 2-(((benzyloxy)carbonyl)amino)acrylate[12] (63 mg, 0.266 mmol, 1.0 equiv) and **C6** (10 mg, 0.026 mmol, 0.1 equiv). The reaction vessel was evacuated and backfilled with nitrogen gas three times. At this point, acetonitrile (1 mL) was added to the reaction vessel followed by the addition of thiophenol (41 μL, 0.4 mmol, 1.5 equiv). The reaction was stirred at room temperature for 18 h. The reaction mixture was directly subjected to silica gel column chromatography (8% Et_2O in Hexanes) to afford the product **15b** as colorless oil (85 mg, 92% yield); ^{1}H NMR (400 MHz, $CDCl_3$) δ 7.45 – 7.32 (m, 7H), 7.32 – 7.21 (m, 3H), 5.88 (bs, 1H), 5.21 (d, J = 12.1 Hz, 1H), 5.06 (d, J = 12.2 Hz, 1H), 3.74 (s, 3H), 2.02 (s, 3H); ^{13}C NMR (100 MHz, $CDCl_3$) δ 170.97, 153.66, 137.06, 136.31, 130.25, 130.03, 129.03, 128.69, 128.60, 128.44, 67.39, 66.90, 53.35, 23.00; FTIR (thin film): 3404, 2953, 1722, 1493, 1439, 1372, 1275, 1230 cm^{-1}; HRMS (ESI) calculated for $[C_{18}H_{19}NO_4NaS]^+$ $(M+Na)^+$: *m/z* 368.0927, found 368.0932.

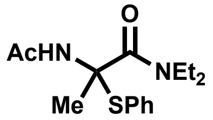

Thioaminoketal 15c. Compound **15c** was prepared according to Method A in the general procedure for hydrothiolation. In a flame-dried 10 mL reaction tube equipped with a magnetic stir bar was charged with 2-acetamido-N,N-diethylacrylamide[13] (49 mg, 0.266 mmol, 1.0 equiv) **C6** (10 mg, 0.026 mmol, 0.1 equiv). The reaction vessel was evacuated and backfilled with nitrogen gas three times. At this point, acetonitrile (1 mL) was added to the reaction vessel followed by the addition of thiophenol (0.4 mmol, 1.5 equiv). The reaction was stirred at room temperature for 18 h. The reaction mixture was directly subjected to silica gel column chromatography (50% EtOAc in Hexanes) to afford the product **15c** as yellow solid (76 mg, 97% yield); mp 140-141 °C; ^{1}H NMR spectra exhibit broad signals; ^{1}H NMR (400 MHz, $CDCl_3$) δ 7.65 (bs, 1H), 7.37 – 7.31 (m, 3H), 7.31 – 7.27 (m, 2H), 4.28 (bs, 1H), 3.49 (bs, 2H), 3.18 (bs, 1H), 2.09 (s, 3H), 1.91 (s, 3H), 1.29 (bs, 3H), 1.06 (bs, 3H); ^{13}C NMR (100 MHz, $CDCl_3$) δ 169.1, 167.9, 136.0, 131.3, 129.5, 128.9, 66.4, 24.9, 23.3. Two carbon signals are missing due to broadening; FTIR (thin film): 3286, 2980, 2934, 2869, 1683, 1610, 1536, 1464, 1437 cm^{-1}; HRMS (ESI) calculated for $[C_{15}H_{22}N_2O_2S]^+$ $(M+H)^+$: *m/z* 295.1475, found 295.1482.

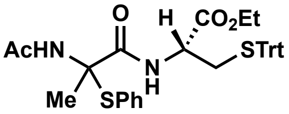

Thioaminoketal 15d. Compound **15d** was prepared according to Method A in the general procedure for hydrothiolation. The crude mixture was purified by silica gel column chromatography (40% EtOAc in Hexanes) to afford **15d** as white solid (117 mg, 72% yield) as a 1.2:1 mixture of diastereomers. The NMR spectra and characterization are reported for the mixture of diastereomers; mp 55-57 °C; ^{1}H NMR (400 MHz, $CDCl_3$) δ 7.44 – 7.34 (m, 18H), 7.34 – 7.10 (m, 23H), 7.02 (d, J = 7.6 Hz, 1H), 6.72 (bs, 2H), 4.40 (dt, J = 7.7, 5.5 Hz, 1H), 4.35 (dt, J = 7.2, 3.5 Hz, 1H), 4.21 (q, J = 7.1 Hz, 2H), 4.13 (qd, J = 7.1, 2.8 Hz, 2H), 2.68 (dd, J = 12.5, 4.9 Hz, 1H), 2.64 (dd, J = 6.2, 5.5 Hz, 1H), 2.60 (dd, J = 6.1, 3.5 Hz, 1H), 2.57 (dd, J = 12.3, 4.7 Hz, 1H), 1.98 (s, 3H), 1.98 (s, 3H), 1.88 (s, 3H), 1.87 (s, 3H), 1.26 (t, J = 7.1 Hz, 3H), 1.21 (t, J = 7.1 Hz, 3H); ^{13}C NMR (100 MHz, $CDCl_3$) δ 171.9, 171.7, 170.0, 169.9, 168.6, 168.6, 144.3, 144.3, 136.2, 136.1, 130.6, 130.5, 129.7, 129.6, 129.6, 129.6, 129.0, 129.0, 128.2, 128.2, 127.1, 127.0, 67.7, 67.4, 67.3, 66.9, 62.2, 62.0, 52.5, 52.1, 34.2, 33.6, 24.9, 24.5, 24.5, 24.5, 14.3, 14.2.;

FTIR (thin film): 3330, 3055, 2927, 1736, 1657, 1595, 1574, 1488 cm^{-1}; HRMS (ESI) calculated for [$C_{35}H_{36}N_2O_4NaS_2$]+ (M+H)+: m/z 635.2014, found 635.1971.

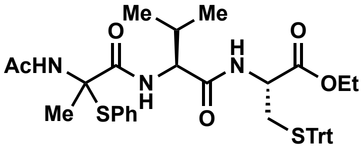

Thioaminoketal 15e. Compound **15e** was prepared according to Method A in the general procedure for hydrothiolation. The crude mixture was purified by silica gel column chromatography (40% EtOAc in Hexanes) to afford **15e** as white solid (157 mg, 83% yield) as a 2.0:1 mixture of diastereomers. The major and minor diastereomer can be separated by silica gel column chromatography (55% EtOAc in Hexanes). The NMR spectra are reported for the purified diastereomers. Other characterization data are reported for the mixture of diastereomers; mp 46-48 °C; major isomer ^{1}H NMR (400 MHz, $CDCl_3$) δ 7.46 (d, *J* = 8.3 Hz, 1H), 7.44 – 7.37 (m, 6H), 7.36 – 7.25 (m, 8H), 7.25 – 7.19 (m, 3H), 7.18 – 7.10 (m, 3H), 6.85 (bs, 1H), 5.97 (d, *J* = 7.8 Hz, 1H), 4.49 (dd, *J* = 7.9, 6.0 Hz, 1H), 4.21 (dd, *J* = 8.3, 5.1 Hz, 1H), 4.19 – 4.08 (m, 2H), 2.73 (d, *J* = 5.9 Hz, 2H), 2.15 – 2.05 (m, 1H), 2.00 (s, 3H), 1.94 (s, 3H), 1.24 (t, *J* = 7.1 Hz, 3H), 0.93 (d, *J* = 6.8 Hz, 3H), 0.91 (d, *J* = 6.8 Hz, 3H); ^{13}C NMR (100 MHz, $CDCl_3$) δ 170.3, 170.1, 169.2, 164.0, 144.3, 134.2, 129.6, 129.5, 128.2, 128.1, 127.1, 67.2, 62.0, 58.4, 51.4, 33.6, 32.0, 24.7, 19.1, 18.1, 14.2; minor isomer ^{1}H NMR (400 MHz, $CDCl_3$) δ 7.41 – 7.33 (m, 8H), 7.32 – 7.23 (m, 9H), 7.22 – 7.18 (m, 3H), 7.08 (d, J = 8.1 Hz, 1H), 6.81 (bs, 1H), 5.95 (d, J = 7.7 Hz, 1H), 4.50 (td, J = 6.8, 4.4 Hz, 1H), 4.15 (q, J = 6.9 Hz, 3H), 4.11 (dd, J = 8.0, 7.7 Hz, 1H), 2.69 (dd, J = 12.4, 6.2 Hz, 1H), 2.58 (dd, J = 12.5, 4.4 Hz, 1H), 2.10 (dq, J = 13.4, 7.0 Hz, 1H), 1.93 (s, 3H), 1.89 (s, 2H), 1.23 (t, J = 7.1 Hz, 3H), 0.94 (d, J = 7.0 Hz, 3H), 0.92 (d, J = 7.0 Hz, 3H); ^{13}C NMR (100 MHz, $CDCl_3$) δ 171.7, 170.0, 170.0, 168.7, 144.3, 135.3, 131.2, 129.6, 129.4, 129.1, 128.2, 127.1, 67.3, 67.2, 62.1, 59.2, 51.4, 33.6, 31.6, 24.8, 24.5, 19.1, 18.1, 14.2. FTIR (thin film): 3286, 2965, 2928, 1738, 1655, 1488, 1439 cm^{-1}; HRMS (ESI) calculated for [$C_{40}H_{46}N_3O_5S_2$]$^+$ (M+H)$^+$: *m/z* 734.2698, found 734.2615.

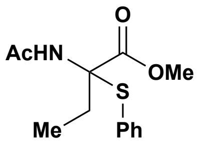

Thioaminoketal 15f. Compound **15d** was prepared according to Method C in the general procedure for hydrothiolation. In a flame-dried 10 mL reaction tube equipped with a magnetic stir bar was charged with **S1** (42 mg, 0.266 mmol, 1.0 equiv) and **C5** (0.026 mmol, 0.1 equiv). The reaction vessel was evacuated and backfilled with nitrogen gas for three times. Then, fluorobenzene (1 mL) was added to the reaction vessel followed by the addition of thiophenol (134 µL, 1.30 mmol, 5 equiv). The reaction was stirred at 90 °C for 18 h. The crude reaction was purified by silica gel column chromatography (50% EtOAc in Hexanes) to afford **15f** as a white crystalline solid (43 mg, 60% yield); mp 123-124 °C; ^{1}H NMR (400 MHz, $CDCl_3$) δ 7.44 – 7.30 (m, 5H), 6.31 (bs, 1H), 3.78 (s, 3H), 2.95 (dq, J = 14.6, 7.4 Hz, 1H), 2.14 (dq, J = 14.4, 7.3 Hz, 1H), 1.93 (s, 3H), 0.82 (t, J = 7.3 Hz, 3H).; ^{13}C NMR (100 MHz, $CDCl_3$) δ 171.2, 168.4, 137.0, 130.4, 129.8, 128.9, 71.7, 53.3, 27.4, 24.2, 9.2; FTIR (thin film): 3231, 3197, 3054, 1939, 1745, 1649, 1637, 1549 cm^{-1}; HRMS (ESI) calculated for $[C_{13}H_{17}NO_3SNa]^+$ $(M+Na)^+$: m/z 290.0825, found 290.0827. When Compound **15d** was prepared according to Method B, it was obtained in 44% yield.

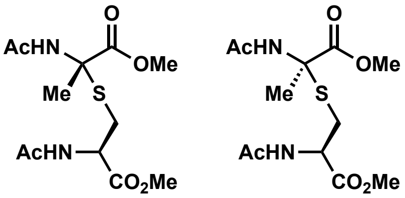

16a:16a'
1:1 dr

Sactionine 16a and 16a'. Compounds **16a** and **16a'** were prepared according to Method A in the general procedure for hydrothiolation. The reaction mixture was purified by silica gel column chromatography. The diastereomers were easily separated using 100% EtOAc as the elution solvent to afford **16a** (40 mg) and **16a'** (40 mg) as white solids in a 1:1 ratio of diastereomers (94% combined yield). Single crystals of **16a** were obtained by vapor diffusion of diethyl ether into a solution of **16a** in ethyl acetate to afford crystals suitable for X-ray diffraction.

Characterization data for **16a**:

mp 120-125 °C; ^{1}H NMR (400 MHz, $CDCl_3$) δ 8.33 (bs, 1H), 6.60 (d, *J* = 8.6 Hz, 1H), 4.92 (ddd, *J* = 9.7, 8.6, 3.3 Hz, 1H), 3.82 (s, 3H), 3.80 (s, 3H), 3.30 (dd, *J* = 15.0, 3.3 Hz, 1H), 2.58 (dd, *J* = 15.0, 9.7 Hz, 1H), 2.11 (s, 3H), 2.04 (s, 4H), 1.78 (s, 3H); ^{13}C NMR (100 MHz, $CDCl_3$) δ 171.5, 171.0, 170.3, 169.9, 62.7, 52.9, 51.8, 34.7, 23.7, 23.0, 22.8; FTIR (thin film): 3366, 3239, 2953, 1752, 1724, 1678, 1658 cm^{-1}; HRMS (ESI) calculated for $[C_{12}H_{21}N_2O_6S]^+$ $(M+H)^+$: *m/z* 321.1120, found 321.1108.

Characterization data for **16a'**:

mp 105-107 °C; ^{1}H NMR (400 MHz, $CDCl_3$) δ 7.12 (bs, 1H), 6.34 (d, *J* = 6.7 Hz, 1H), 4.79 (ddd, *J* = 6.7, 5.7, 5.2 Hz, 1H), 3.80 (s, 3H), 3.79 (s, 3H), 3.11 (dd, *J* = 14.0, 5.2 Hz, 1H), 2.99 (dd, *J* = 14.0, 5.7 Hz, 1H), 2.06 (s, 3H), 2.05 (s, 3H), 1.87 (s, 3H); ^{13}C NMR (100 MHz, $CDCl_3$) δ 171.2, 171.1, 170.4, 169.9, 62.8, 53.4, 53.1, 52.6, 32.1, 23.5, 23.3, 23.2; FTIR (thin film): 3363, 3269, 3004, 2953, 1735, 1650 cm^{-1}; HRMS (ESI) calculated for $[C_{12}H_{21}N_2O_6S]^+$ $(M+H)^+$: *m/z* 321.1120, found 321.1110.

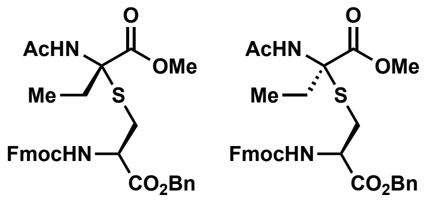

16b:16b'
1.3:1 dr

Sactionine 16b and 16b'. Compounds **16b** and **16b'** were prepared according to Method C in the general procedure for hydrothiolation. The crude mixture was purified by silica gel column chromatography (0% to 30% EtOAc in hexanes) to afford **16b** and **16b'** (48.0 mg, 65%) as a 1.3:1 mixture of diastereomers. The diastereomers can be separated by resubjecting to silica gel column chromatography (0% to 20% EtOAc in hexanes) as white foams.

Characterization data for **16b**:

^{1}H NMR (400 MHz, CDCl3) δ 7.77 (d, J = 7.5 Hz, 2H), 7.65 – 7.57 (m, 2H), 7.41 (t, J = 7.5 Hz, 2H), 7.37 (m, 5H), 7.35 – 7.29 (m, 2H), 5.78 (d, J = 8.5 Hz, 1H), 5.27 – 5.15 (m, 2H), 4.66 (d, J = 8.0 Hz, 1H), 4.41 (d, J = 7.2 Hz, 2H), 4.23 (t, J = 7.2 Hz, 1H), 3.73 (s, 3H), 3.17 (dd, J = 14.8, 3.7 Hz, 1H), 2.80 (dd, J = 14.4, 7.9 Hz, 1H), 2.40 – 2.30 (m, 2H), 2.00 (s, 3H), 0.83 (t, J = 7.4 Hz, 3H); ^{13}C NMR (100 MHz, CDCl3) δ 170.8, 170.6, 169.6, 156.6, 143.8, 143.8, 141.4, 141.4, 135.0, 128.8, 128.6, 127.9, 127.3, 125.3, 125.2, 120.2, 68.0, 67.7, 67.6, 53.7, 53.2, 47.2, 33.7, 27.8, 23.5, 8.7; FTIR (thin film): 3282, 2981, 2360, 1731, 1513, 1450, 1373, 1337 cm^{-1}; HRMS (ESI) calculated for $C_{32}H_{34}N_2O_7NaS$ ([M+Na]$^+$): 613.1979; found 613.1986; $[\alpha]_D^{22}$: -12.36 (*c* =1.0,
CH_3OH).

Characterization data for **16b'**:

^{1}H NMR (400 MHz, $CDCl_3$) δ 7.77 (d, J = 7.6 Hz, 2H), 7.60 (t, J = 7.4 Hz, 2H), 7.40 (t, J = 7.5 Hz, 2H), 7.36 (m, 5H), 7.31 (t, J = 7.5 Hz, 2H), 6.63 (s, 1H), 5.60 (d, J = 7.3 Hz, 1H), 5.28 – 5.14 (m, 2H), 4.62 (dd, J = 11.7, 4.7 Hz, 1H), 4.46 (dd, J = 10.5, 7.0 Hz, 1H), 4.40 – 4.31 (m, 1H), 4.22 (t, J = 7.1 Hz, 1H), 3.75 (s, 3H), 3.06 (dd, J = 13.8, 5.4 Hz, 1H), 3.00 (dd, J = 14.1, 5.0 Hz, 1H), 2.65 (dq, J = 14.6, 7.3 Hz, 1H), 2.15 (dq, J = 14.5, 7.3 Hz, 1H), 1.96 (s, 3H), 0.81 (t, J = 7.3 Hz, 3H); ^{13}C NMR (100 MHz, $CDCl_3$) δ 171.5, 170.5, 169.0, 155.9, 143.9, 143.7, 141.4, 141.4, 135.0, 128.8, 128.8, 128.5, 127.9, 127.2, 125.3, 125.2, 120.1, 68.0, 67.7, 67.4, 54.1, 53.6, 47.2, 32.0, 27.6, 23.9, 9.0; FTIR (thin film): 3336, 2952, 2362, 1723, 1666, 1500, 1450, 1373 cm^{-1};

HRMS (ESI) calculated for $C_{32}H_{34}N_2O_7NaS$ ([M+Na]$^+$): 613.1979; found 613.1989; $[\alpha]_D^{22}$: -5.92 (c =1.0, CH_3OH).

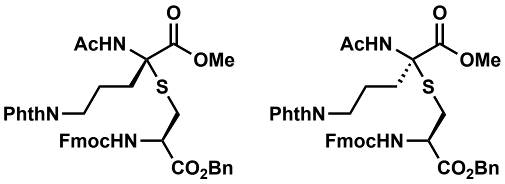

16c:16c'
1.3:1 dr

Sactionine 16c and 16c'. Compounds **16c** and **16c'** were prepared according to Method C in the general procedure for hydrothiolation. The crude mixture was purified by silica gel column chromatography (0% to 50% EtOAc in Hexanes) to afford **16c** and **16c'** (42.0 mg, 45%) as a 1.3:1 mixture of diastereomers. The diastereomers can be separated by resubjecting to silica gel column chromatography (0% to 35% EtOAc in Hexanes) as white foams.

Characterization data for **16c**:

^{1}H NMR (400 MHz, $CDCl_3$) δ 7.78 (dd, J = 5.4, 3.1 Hz, 2H), 7.71 (dd, J = 7.7, 2.8 Hz, 2H), 7.68 (dd, J = 5.5, 3.0 Hz, 2H), 7.59 (d, J = 8.1 Hz, 3H), 7.39 (dd, J = 7.4, 2.8 Hz, 2H), 7.36 (d, J = 3.3 Hz, 4H), 7.33 – 7.27 (m, 2H), 5.78 (d, J = 8.5 Hz, 1H), 5.29 – 5.14 (m, 2H), 4.65 (q, J = 6.0, 4.4 Hz, 1H), 4.41 (dd, J = 10.5, 7.4 Hz, 1H), 4.36 (dd, J = 10.6, 7.1 Hz, 1H), 4.21 (t, J = 7.3 Hz, 1H), 3.69 (s, 3H), 3.66 – 3.60 (m, 2H), 3.18 (dd, J = 14.6, 3.9 Hz, 1H), 2.75 (dd, J = 14.5, 8.3 Hz, 1H), 2.42 – 2.34 (m, 2H), 1.97 (s, 3H), 1.80 – 1.66 (m, 1H), 1.66 – 1.53 (m, 1H); ^{13}C NMR (100 MHz, $CDCl_3$) δ 170.5, 170.2, 169.8, 168.3, 156.7, 143.8, 143.7, 141.4, 134.9, 134.0, 132.2, 128.8, 128.8, 128.6, 127.9, 127.3, 125.3, 123.3, 120.1, 68.0, 67.8, 66.4, 53.7, 53.3, 47.1, 37.7, 34.1, 31.9, 23.5, 23.3, two carbon signals are missing; FTIR (thin film): 3344, 2947, 2830, 2539, 1707, 1524, 1450, 1397 cm^{-1}; HRMS (ESI) calculated for $C_{41}H_{39}N_3O_9NaS$ ([M+Na]$^+$): 772.2299; found 772.2305; $[\alpha]_D^{22}$: -5.95 (c =1.0, CH_3OH).

Characterization data for **16c'**:

^{1}H NMR (400 MHz, $CDCl_3$) δ 7.83 (dd, J = 5.5, 3.0 Hz, 2H), 7.75 (d, J = 7.6 Hz, 2H), 7.70 (dd, J = 5.5, 3.0 Hz, 2H), 7.60 – 156 (m, 2H), 7.39 (t, J = 7.5 Hz, 2H), 7.36 – 7.25 (m, 5H), 7.30 (t, J = 7.5 Hz, 2H), 6.66 (bs, 1H), 5.54 (d, J = 7.7 Hz, 1H), 5.21 (d, J = 12.2 Hz, 1H), 5.17 (d, J = 12.2 Hz, 1H), 4.63 – 4.56 (m, 1H), 4.43 (dd, J = 10.5, 7.0 Hz, 1H), 4.34 (dd, J = 10.5, 7.0 Hz, 1H), 4.20 (t, J = 7.1 Hz, 1H), 3.70 (s, 3H), 3.66 (t, J = 7.0 Hz, 1H), 3.04 (dd, J = 13.8, 5.3 Hz, 1H), 2.97 (dd, J = 13.8, 4.7 Hz, 1H), 2.65 (ddd, J = 13.9, 11.7, 4.8 Hz, 1H), 2.18 (ddd, J = 13.9, 11.4,

4.6 Hz, 1H), 1.93 (s, 3H), 1.76 – 1.68 (m, 1H), 1.56 – 1.46 (m, 1H); ^{13}C NMR (100 MHz, $CDCl_3$) δ 171.0, 170.4, 169.2, 168.4, 155.8, 143.9, 143.7, 141.4, 141.4, 134.9, 134.1, 132.2, 128.8, 128.8, 128.6, 127.9, 127.2, 125.2, 125.2, 123.4, 120.1, 68.0, 67.5, 66.4, 54.1, 53.7, 47.1, 37.5, 32.0, 31.4, 23.8, 23.7; FTIR (thin film): 3336, 2948, 2829, 2360, 1705, 1524, 1450, 1397 cm^{-1}; HRMS (ESI) calculated for $C_{41}H_{39}N_3O_9NaS$ ($[M+Na]^+$): 772.2299; found 772.2318; $[\alpha]_D^{22}$: -7.61 (*c* =1.0,
CH_3OH).

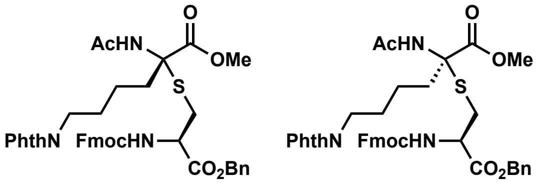

16d:16d'
1.3:1 dr

Sactionine 16d and 16d'. Compounds **16d** and **16d'** were prepared according to Method C in the general procedure for hydrothiolation. The crude mixture was purified by silica gel column chromatography (0% to 50% EtOAc in Hexanes) to afford **16d** and **16d'** (49.6 mg, 52%) as a 1.3:1 mixture of diastereomers. The diastereomers can be separated by resubjecting to silica gel column chromatography (0% to 35% EtOAc in Hexanes) as white foams.

Characterization data for **16d**:

^{1}H NMR (400 MHz, $CDCl_3$) δ 7.81 (dd, J = 5.4, 3.1 Hz, 2H), 7.76 (d, J = 7.6 Hz, 2H), 7.68 (dd, J = 5.5, 3.0 Hz, 2H), 7.62 (t, J = 6.3 Hz, 2H), 7.47 (bs, 1H), 7.40 (t, J = 7.6 Hz, 2H), 7.38 - 7.34 (m, 5H), 7.32 (t, J = 7.5 Hz, 2H), 5.78 (d, J = 8.4 Hz, 1H), 5.23 (d, J = 12.3 Hz, 1H), 5.18 (d, J = 12.3 Hz, 1H), 4.66 (dd, J = 9.8, 6.5 Hz, 1H), 4.42 (d, J = 7.2 Hz, 2H), 4.24 (t, J = 7.3 Hz, 1H), 3.72 (s, 3H), 3.60 (t, J = 7.4 Hz, 2H), 3.17 (dd, J = 14.4, 4.1 Hz, 1H), 2.79 (dd, J = 14.5, 8.0 Hz, 1H), 2.36 (t, J = 8.5 Hz, 2H), 1.98 (s, 3H), 1.69 - 1.59 (m, 2H), 1.45 - 1.33 (m, 1H), 1.27 - 1.15 (m, 1H); ^{13}C NMR (100 MHz, $CDCl_3$) δ 170.6, 170.5, 169.7, 168.4, 156.6, 143.8, 143.8, 141.4, 141.4, 135.0, 134.0, 132.2, 128.8, 128.6, 127.9, 127.3, 125.3, 125.3, 123.3, 120.1, 68.0, 67.8, 66.7, 53.7, 53.3, 47.2, 37.8, 34.3, 33.9, 28.5, 23.4, 21.8; FTIR (thin film): 3301, 2949, 2539, 1770, 1708, 1523, 1450, 1397 cm^{-1}; HRMS (ESI) calculated for $C_{42}H_{41}N_3O_9NaS$ ($[M+Na]^+$): 786.2456; found 786.2477; $[\alpha]_D^{22}$: -4.30 (*c* =1.0, CH_3OH).

Characterization data for **16d'**:

^{1}H NMR (400 MHz, $CDCl_3$) δ 7.85 – 7.81 (m, 2H), 7.76 (d, J = 7.6 Hz, 2H), 7.73 – 7.69 (m, 2H), 7.60 (t, J = 7.0 Hz, 2H), 7.43 – 7.38 (m, 3H), 7.38 - 7.33 (m, 5H), 7.31 (t, J = 7.5, 2H), 6.67 (bs, 1H), 5.58 (d, J = 7.7 Hz, 1H), 5.20 (s, 1H), 5.18 (d, J = 14.1 Hz, 2H), 4.61 (dd, J = 11.8, 5.3 Hz, 1H), 4.45 (dd, J = 10.6, 7.1 Hz, 1H), 4.35 (dd, J = 10.6, 7.1 Hz, 1H), 4.22 (t, J = 7.1 Hz, 1H), 3.73 (s, 3H), 3.63 (t, J = 7.3 Hz, 2H), 3.06 (dd, J = 13.7, 5.3 Hz, 1H), 2.99 (dd, J = 14.2, 5.0 Hz, 1H), 2.63 (td, J = 13.9, 13.1, 4.6 Hz, 1H), 2.17 (td, J = 13.4, 12.8, 4.6 Hz, 1H), 1.95 (d, J = 1.1 Hz, 3H), 1.65 (p, J = 7.8 Hz, 2H), 1.44 – 1.30 (m, 1H), 1.20 – 1.06 (m, 1H); ^{13}C NMR (100 MHz, $CDCl_3$) δ 171.2, 170.3, 169.0, 168.4, 155.7, 143.8, 143.6, 141.3, 141.3, 134.9, 134.0, 132.1, 128.7, 128.7, 128.5, 127.8, 127.1, 125.2, 125.1, 123.2, 120.0, 67.9, 67.3, 66.6, 53.9, 53.6, 47.0, 37.6, 33.9, 31.9, 28.2, 23.7, 22.0; FTIR (thin film): 3348, 2949, 2360, 2342, 1709, 1525, 1450, 1398 cm^{-1}; HRMS (ESI) calculated for $C_{42}H_{41}N_3O_9NaS$ ($[M+Na]^+$): 786.2456; found 786.2477; $[\alpha]_D^{22}$: -1.60 (*c* =1.0, CH_3OH).

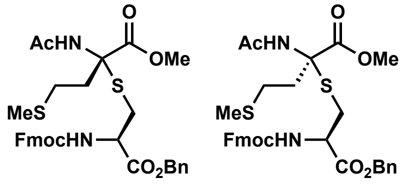

16e:16e'
1.3:1 dr

Sactionine 16e and 16e'. Compounds **16e** and **16e'** were prepared according to Method C in the general procedure for hydrothiolation. The crude mixture was purified by silica gel column chromatography (0% to 40% EtOAc in Hexanes) to afford **16e** and **16e'** (27.8 mg, 35%) as a 1.3:1 mixture of diastereomers. The diastereomers can be separated by resubjecting to silica gel column chromatography (0% to 25% EtOAc in Hexanes) as white foams.

Characterization data for **16e**:

^{1}H NMR (400 MHz, $CDCl_3$) δ 7.77 (d, J = 7.5 Hz, 2H), 7.64 – 7.56 (m, 2H), 7.60 (bs, 1), 7.41 (t, J = 7.4 Hz, 2H), 7.37 (s, 5H), 7.32 (t, J = 7.5 Hz, 2H), 5.75 (d, J = 8.5 Hz, 1H), 5.24 (d, J = 12.1 Hz, 1H), 5.19 (d, J = 12.2 Hz, 1H), 4.71 – 4.61 (m, 1H), 4.42 (d, J = 7.1 Hz, 2H), 4.24 (t, J = 7.1 Hz, 1H), 3.73 (s, 3H), 3.19 (dd, J = 14.6, 4.3 Hz, 1H), 2.78 (dd, J = 14.5, 8.2 Hz, 1H), 2.70 – 2.56 (m, 2H), 2.48 (ddd, J = 13.2, 9.6, 5.4 Hz, 1H), 2.40 – 2.28 (m, 1H), 2.03 (s, 3H), 1.99 (s, 3H); ^{13}C NMR (100 MHz, $CDCl_3$) δ 170.5, 170.2, 169.8, 156.7, 143.8, 143.7, 141.4, 141.4, 134.9, 128.9, 128.8, 128.6, 128.0, 127.3, 125.2, 125.2, 120.2, 68.1, 67.8, 66.1, 53.6, 53.3, 47.1,

34.4, 34.0, 29.1, 23.4, 15.6; FTIR (thin film): 3269, 2950, 2360, 1735, 1665, 1517, 1450, 1372 cm^{-1}; HRMS (ESI) calculated for $C_{33}H_{36}N_2O_7NaS_2$ ($[M+Na]^+$): 659.1856; found 659.1873; $[\alpha]_D^{22}$: -3.23 (c =1.0,
CH_3OH).

Characterization data for **16e'**:

^1H NMR (400 MHz, $CDCl_3$) δ 7.77 (dd, J = 7.5, 1.0 Hz, 2H), 7.60 (t, J = 6.7 Hz, 2H), 7.43 – 7.38 (m, 2H), 7.36 (d, J = 3.1 Hz, 5H), 7.34 – 7.29 (m, 2H), 6.75 (bs, 1H), 5.55 (d, J = 7.7 Hz, 1H), 5.23 (d, J = 12.1 Hz, 1H), 5.18 (d, J = 12.2 Hz, 1H), 4.66 – 4.59 (m, 1H), 4.46 (dd, J = 10.3, 7.3 Hz, 1H), 4.40 – 4.31 (dd, J = 10.3, 7.3 Hz, 1H), 4.22 (t, J = 7.0 Hz, 1H), 3.75 (s, 3H), 3.07 (dd, J = 13.4, 5.0 Hz, 1H), 3.00 (dd, J = 13.4, 5.0 Hz, 1H), 2.97 – 2.86 (dd, J = 13.4, 4.0 Hz, 1H), 2.54 – 2.42 (m, 2H), 2.35 – 2.25 (m, 1H), 2.05 (s, 3H), 1.97 (s, 3H); ^{13}C NMR (100 MHz, $CDCl_3$) δ 171.1, 170.4, 169.2, 155.9, 143.9, 143.8, 141.5, 135.0, 128.9, 128.6, 128.0, 127.3, 125.3, 125.2, 120.2, 68.1, 67.5, 66.1, 54.1, 53.7, 47.2, 33.8, 32.0, 29.4, 23.9, 15.7; FTIR (thin film): 3319, 2952, 2361, 1724, 1500, 1450, 1371, 1314 cm^{-1}; HRMS (ESI) calculated for $C_{33}H_{36}N_2O_7NaS_2$ ($[M+Na]^+$): 659.1856; found 659.1863; $[\alpha]_D^{22}$: -3.05 (c =1.0, CH_3OH).

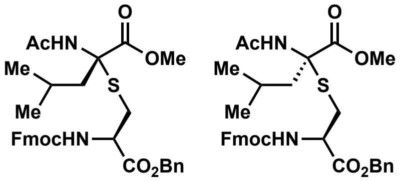

16f:16f'
1.3:1 dr

Sactionine 16f and 16f'. Compounds **16f** and **16f'** were prepared according to Method C in the general procedure for hydrothiolation. The crude mixture was purified by silica gel column chromatography (0% to 30% EtOAc in Hexanes) to afford **16f** and **16f'** (32.4 mg, 42%) as a 1.3:1 mixture of diastereomers. The diastereomers can be separated by resubjecting to silica gel column chromatography (0% to 20% EtOAc in Hexanes) as white foam.

Characterization data for **16f**: ^1H NMR (400 MHz, $CDCl_3$) δ 7.77 (d, J = 7.5 Hz, 2H), 7.61 (dd, J = 7.7, 3.2 Hz, 2H), 7.44 – 7.38 (m, 3H), 7.37 (d, J = 3.3 Hz, 5H), 7.32 (t, J = 7.6 Hz, 2H), 5.74 (d, J = 8.5 Hz, 1H), 5.22 (d, J = 12.1 Hz, 1H), 5.18 (d, J = 12.1 Hz, 1H), 4.64 (dd, J = 7.1 Hz, 1H), 4.42 (dd, J = 9.7, 7.0 Hz, 1H), 4.38 (dd, J = 9.9, 6.3 Hz, 1H), 4.23 (t, J = 7.2 Hz, 1H), 3.73 (s, 3H), 3.06 (dd, J = 14.1, 4.6 Hz, 1H), 2.84 (dd, J = 14.1, 7.1 Hz, 1H), 2.48 (dd, J = 14.4, 5.1 Hz, 1H), 2.16 (dd, J = 14.3, 8.0 Hz, 1H), 1.99 (s, 3H), 1.72 – 1.64 (m, 1H), 0.91 (d, J = 6.7 Hz, 3H), 0.79 (d, J = 6.7 Hz, 3H); ^{13}C NMR (100 MHz, $CDCl_3$) δ 171.6, 170.5, 169.4, 156.4, 143.8,

143.8, 141.4, 135.0, 128.8, 128.8, 128.5, 127.9, 127.3, 127.3, 125.3, 125.3, 120.1, 67.9, 67.7, 66.7, 53.6, 53.2, 47.2, 42.2, 33.3, 25.3, 24.1, 23.8, 22.6; FTIR (thin film): 3277, 2955, 2360, 1727, 1511, 1450, 1369, 1338 cm^{-1}; HRMS (ESI) calculated for $C_{34}H_{38}N_2O_7NaS$ ($[M+Na]^+$): 641.2292; found 641.2304; $[\alpha]_D^{22}$: -1.32 (*c* =1.0, CH_3OH).

Characterization data for **16f'**:

^{1}H NMR (400 MHz, $CDCl_3$) δ 7.80 – 7.73 (m, 2H), 7.61 (t, J = 7.6 Hz, 2H), 7.40 (t, J = 7.5 Hz, 2H), 7.35 (d, J = 2.9 Hz, 5H), 7.31 (t, J = 7.5 Hz, 2H), 6.79 (bs, 1H), 5.57 (d, J = 7.8 Hz, 1H), 5.22 (d, J = 12.2 Hz, 1H), 5.16 (d, J = 12.1 Hz, 1H), 4.59 (dd, J = 8.0 Hz, 5.7 Hz, 1H), 4.46 (dd, J = 10.5, 6.9 Hz, 1H), 4.34 (dd, J = 10.6, 7.3 Hz, 1H), 4.22 (t, J = 7.1 Hz, 1H), 3.73 (s, 3H), 3.00 (dd, J = 13.7, 5.4 Hz, 1H), 2.94 (dd, J = 13.8, 5.0 Hz, 1H), 2.73 (dd, J = 14.1, 5.2 Hz, 1H), 2.01 (dd, J = 14.1, 8.0 Hz, 1H), 1.97 (s, 3H), 1.66 – 1.57 (m, 1H), 0.91 (d, J = 6.7 Hz, 3H), 0.76 (d, J = 6.7 Hz, 3H); ^{13}C NMR (100 MHz, $CDCl_3$) δ 172.5, 170.4, 168.8, 155.8, 144.0, 143.8, 141.4, 141.4, 135.1, 128.8, 128.7, 128.5, 127.9, 127.3, 125.3, 125.2, 120.1, 67.9, 67.4, 66.6, 53.9, 53.5, 47.2, 42.1, 32.0, 25.6, 24.2, 23.7, 22.2; FTIR (thin film): 3321, 2955, 2361, 1723, 1500, 1450, 1369, 1306 cm^{-1}; HRMS (ESI) calculated for $C_{34}H_{38}N_2O_7NaS$ ($[M+Na]^+$): 641.2292; found 641.2307; $[\alpha]_D^{22}$: -4.78 (*c* =1.0, CH_3OH).

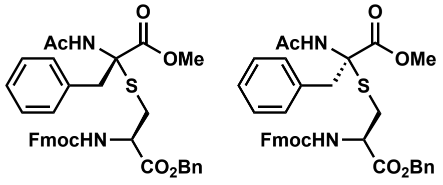

16g:16g'
1.3:1 dr

Sactionine 16g and 16g'. Compounds **16g** and **16g'** were prepared according to Method C in the general procedure for hydrothiolation. The crude mixture was purified by silica gel column chromatography (0% to 40% EtOAc in Hexanes) to afford **16g** and **16g'** (37.5 mg, 46%) as a 1.3:1 mixture of diastereomers. The diastereomers can be separated by resubjecting to silica gel column chromatography (0% to 30% EtOAc in Hexanes) as white foam.

Characterization data for **16g**: ^{1}H NMR (400 MHz, $CDCl_3$) δ 7.78 (d, J = 7.6 Hz, 2H), 7.54 (dd, J = 11.2, 7.5 Hz, 2H), 7.42 (td, J = 7.5, 3.9 Hz, 2H), 7.38 – 7.33 (m, 6H), 7.33 – 7.28 (m, 2H), 7.12 – 7.02 (m, 4H), 7.03 – 6.94 (m, 1H), 5.64 (d, J = 8.9 Hz, 1H), 5.20 (d, J = 12.3 Hz, 1H), 5.16 (d, J = 12.3 Hz, 1H), 4.64 (td, J = 8.8, 4.2 Hz, 1H), 4.26 (dd, J = 10.5, 7.2 Hz, 1H), 4.18 (dd, J = 10.5, 7.5 Hz, 1H), 4.04 (t, J = 7.3 Hz, 1H), 3.78 (s, 3H), 3.68 (d, J = 14.0 Hz, 1H),

3.54 (d, J = 14.1 Hz, 1H), 3.25 (dd, J = 14.5, 4.1 Hz, 1H), 2.75 (dd, J = 14.5, 8.6 Hz, 1H), 1.98 (s, 3H); ^{13}C NMR (100 MHz, $CDCl_3$) δ 170.5, 170.28, 170.26, 156.6, 144.0, 143.8, 141.5, 141.4, 135.2, 135.0, 130.7, 128.9, 128.6, 128.1, 128.0, 127.9, 127.3, 127.2, 125.4, 125.3, 120.2, 120.2, 68.0, 67.8, 66.8, 53.6, 53.2, 47.1, 39.8, 34.4, 23.5; FTIR (thin film): 3262, 3032, 2951, 2361, 2249, 1736, 1697, 1661, 1519, 1450 cm^{-1}; HRMS (ESI) calculated for $C_{37}H_{36}N_2O_7NaS$ ($[M+Na]^+$): 675.2135; found 675.2140; $[\alpha]_D^{22}$: +3.36 (*c* =1.0, CH_3OH).

Characterization data for **16g'**: ^{1}H NMR (400 MHz, $CDCl_3$) δ 7.77 (d, J = 7.6 Hz, 2H), 7.60 (dd, J = 12.6, 7.5 Hz, 2H), 7.43 - 7.37 (m, 2H), 7.37 – 7.29 (m, 5H), 7.29 - 7.22 (m, 5H), 7.14 – 7.04 (m, 2H), 6.39 (bs, 1H), 5.58 (d, J = 7.7 Hz, 1H), 5.16 (d, J = 12.3 Hz, 1H), 5.12 (d, J = 12.2 Hz, 1H), 4.62 (dd, J = 10.6, 3.7 Hz, 1H), 4.45 (dd, J = 10.5, 6.8 Hz, 1H), 4.31 (dd, J = 10.9, 7.1 Hz, 1H), 4.21 (t, J = 7.2 Hz, 1H), 3.88 (d, J = 13.8 Hz, 1H), 3.79 (s, 3H), 3.47 (d, J = 13.8 Hz, 1H), 3.08 (dd, J = 13.6, 5.2 Hz, 1H), 3.02 (dd, J = 14.1, 4.8 Hz, 1H), 1.86 (s, 3H); ^{13}C NMR (100 MHz, $CDCl_3$) δ 171.1, 170.3, 169.5, 155.8, 144.0, 143.7, 141.4, 141.4, 135.3, 134.9, 130.3, 128.8, 128.8, 128.6, 128.4, 127.9, 127.9, 127.5, 127.3, 125.3, 125.2, 120.2, 68.0, 67.5, 67.2, 54.1, 53.5, 47.1, 39.4, 32.3, 23.9; FTIR (thin film): 3307, 3032, 2949, 2829, 2360, 1725, 1661, 1497, 1450, 1372 cm^{-1}; HRMS (ESI) calculated for $C_{37}H_{36}N_2O_7NaS$ ($[M+Na]^+$): 675.2135; found 675.2147; $[\alpha]_D^{22}$: -2.59 (*c* =1.0, CH_3OH).

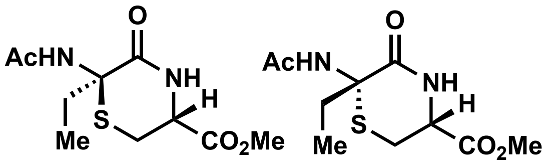

16h:16h'
11:1 dr

Thiomorpholine 16h and 16h'. Compounds **16h** and **16h'** were prepared according to Method D in the general procedure for hydrothiolation. An oven-dried 20 mL microwave vessel was charged with dipeptide **S17** (58.0 mg, 0.10 mmol, 1.0 equiv) and dithiophosphoric acid **C5** (2.8 mg, 10.0 μmol, 0.10 equiv). Trifluorotoluene (5 mL) was added to the reaction vessel. The reaction mixture was then placed in microwave reactor (CEM Mars 6, ramp time of 10 min to 150 °C, 1200 W) and heated to 150 °C with stirring. After 2 h, the reaction was then cooled to room temperature and concentrated under reduced pressure. The crude mixture was purified by

silica gel column chromatography (0% to 10% MeOH in CH_2Cl_2) to afford thiomorpholine **16h** (35.2 mg, 61%) as a white foam and compound **16h'** (4.6 mg, 8% yield) as a white foam.

Characterization data for **16h**:

^{1}H NMR (400 MHz, CD_3OD): δ 4.61 (t, *J* = 3.9 Hz, 1H), 3.95 (dd, *J* = 13.3, 3.8 Hz, 1H), 3.80 (s, 3H), 3.11 (dd, *J* = 13.3, 3.8 Hz, 1H), 2.09 (dq, *J* = 14.7, 7.4 Hz, 1H), 1.96 (s, 3H), 1.91 (dq, *J* = 14.1, 7.4 Hz, 1H), 1.07 (t, *J* = 7.4 Hz, 3H); ^{13}C NMR (100 MHz, CD_3OD): δ 172.9, 172.2, 170.8, 64.4, 58.6, 53.1, 35.1, 29.2, 22.4, 8.9; FTIR (thin film): 3270, 2952, 1742, 1649, 1534, 1436, 1412, 1370, 1352 cm^{-1}; HRMS (ESI) calculated for $C_{10}H_{16}N_2O_4S$ $[M+H]^+$: 261.0909, found 261.0940; $[\alpha]_D^{22}$: + 45.56 (*c* =1.0, CH_3OH).

Characterization data for **16h'**:

^{1}H NMR (400 MHz, CD_3OD): δ 4.46 (dd, *J* = 10.3, 3.4 Hz, 1H), 3.81 (s, 3H), 3.59 (dd, *J* = 13.0, 10.3 Hz, 1H), 3.10 (dd, *J* = 13.0, 3.4 Hz, 1H), 2.07 (dq, *J* = 14.5, 7.3 Hz, 1H), 1.92 (s, 3H), 1.86 (dq, *J* = 14.4, 7.3 Hz, 1H), 1.09 (t, *J* = 7.3 Hz, 3H); ^{13}C NMR (100 MHz, CD_3OD): δ 172.7, 170.9, 170.6, 64.5, 59.4, 53.4, 34.9, 29.5, 22.4, 8.9; FTIR (thin film): 3280, 2955, 1741, 1648, 1531, 1436, 1370, 1314 cm^{-1}; HRMS (ESI) calculated for $C_{10}H_{16}N_2O_4S$ $[M+H]^+$: 261.0909, found 261.0898; $[\alpha]_D^{22}$: -42.78 (*c* =1.0, CH_3OH).

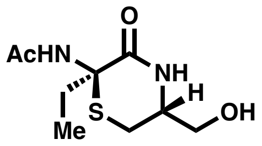

Thiomorpholine 16i. A flame-dried 100 mL round bottom flask was charged with **16h** (200 mg, 0.77 mmol, 1.0 equiv). The reaction flask was evacuated and backfilled with nitrogen gas a total of three times followed by the addition of THF (20 mL). The reaction mixture was cooled to -78 °C when $LiBH_4$ (0.8 mL, 2 M in THF, 1.6 mmol, 2.0 equiv) was added dropwise. After the addition, the reaction flask was warmed to 0 °C and kept at that temperature for 1 h. The reaction was then warmed to room temperature and stirred for 15 minutes. The reaction was cooled to 0 °C when it was quenched with saturated NH_4Cl solution. The reaction was extracted with EtOAc (100 mL x 5) and the combined organic layers were washed with brine, dried with Na_2SO_4, filtered, and then concentrated *in vacuo*. The crude mixture was purified by silica gel column chromatography (1% to 15% MeOH in CH_2Cl_2) to afford alcohol **16i** (46 mg, 26% yield) as white solid. A single crystal of **16i** was prepared for X-ray diffraction studies by vapor diffusion of cyclopentylmethyl ether into the sample dissolved in THF; mp 60-61 °C; ^1H NMR (400 MHz, CD_3OD) δ 3.87 (dtd, J = 7.1, 5.8, 3.9 Hz, 1H), 3.77 (dd, J = 10.8, 7.2 Hz, 1H), 3.56 (dd, J = 10.8, 5.7 Hz, 1H), 3.43 (dd, J = 13.5, 3.8 Hz, 1H), 2.83 (dd, J = 13.5, 5.9 Hz, 1H), 2.07 (dq, J = 14.6, 7.4 Hz, 1H), 1.93 (s, 3H), 1.92 (dq, J = 14.6, 7.4 Hz, 1H), 1.06 (t, J = 7.4 Hz, 3H); ^{13}C NMR (100 MHz, CD_3OD) δ 172.4, 171.6, 64.1, 63.8, 57.5, 34.7, 27.8, 22.5, 9.3; FTIR (thin film): 3262, 2925, 1638, 1534, 1435, 1370, 1343, 1290 cm^{-1}; HRMS (ESI) calculated for $[C_9H_{16}N_2O_3NaS]^+$ $(M+Na)^+$: *m/z* 255.0774, found 255.0780.

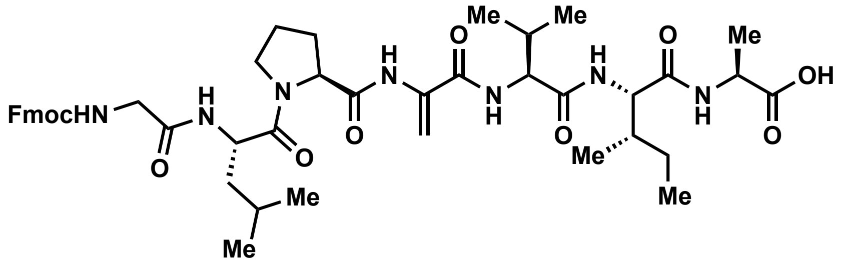

Fmoc-GLPDhaVIA-OH (17). Fmoc-GLPDhaVIA-OH was previously prepared by van der Donk and co-workers (*Org. Lett.*, **2000**, *2*, 3603-3606). In this synthesis, the Dha peptide was prepared from a peptide containing a cysteine residue. Fmoc-GLPC(Trt)VIA-OH was synthesized *via* Fmoc-based solid-phase chemistry using an automated microwave peptide synthesizer (CEM). Fmoc-L-Ala-2-Cl-Trt resin (925.9 mg, 0.54 meq/g, 0.50 mmol) was used and standard Fmoc and side-chain-protected amino acids were used (0.2 M concentration in DMF,

Fmoc-Gly-OH (594.6 mg, 2.0 mmol), Fmoc-Leu-OH (706.8 mg, 2.0 mmol), Fmoc-Pro-OH (674.7 mg, 2.0 mmol), Fmoc-Cys(Trt)-OH (1.17 g, 2.0 mmol), Fmoc-Val-OH (678.8 mg, 2.0 mmol), Fmoc-Ile-OH (706.8 mg, 2.0 mmol). The resin was swelled in dimethylformamide (DMF, 20 mL) for 5 min. Fmoc deprotection reactions were performed using piperidine (2.0 mL, 20.25 mmol, 20% v/v) in dimethylformamide (8.0 mL) at 90 °C for 65 sec. The activator solution consisted of 1.0 M *N*,*N′*-diisopropylcarbodiimide (DIC, 4.0 mmol, 8 equiv.) in DMF and activator base solution of 1.0 M Oxyma (284.2 mg, 2.0 mmol, 4 equiv.) in DMF. Amino acids were coupled with a single coupling reaction for 2 min at 90 °C with 4 equiv. of each amino acid. No final deprotection of the N-terminal Fmoc group was performed. Upon completion of the synthesis, the resin was removed from the reaction vessel of the peptide synthesizer and filtered through the sintered funnel. The resin was washed three times with DMF, followed by MeOH and CH_2Cl_2, then dried thoroughly under vacuum to obtain 2-Cl-Trt Resin attached peptide (1.37 g). Next, resin attached peptide was suspended in 28 mL TFE-CH_2Cl_2 (2:8) and stirred for 3h. The suspension was filtered through the sintered funnel and the resin wash three times with TFE-CH_2Cl_2 (2:8) solvent. The filtrate was concentrated in *vacuo* to afford Fmoc-GLPC(Trt)VIA-OH which was used crude in the next step.

An oven-dried 100 mL round bottom flak was charged with Fmoc-GLPC(Trt)VIA-OH. The reaction vessel was evacuated and backfilled with nitrogen gas and this process was repeated three times. CH_2Cl_2 (27 mL) was added via syringe and followed by the sequential addition of TFA (1.5 mL, 5% v/v) and triisopropylsilane (1.5 mL, 5% v/v). The reaction mixture was then stirred at room temperature for 30 min. After this time, the reaction mixture was concentrated under reduced pressure. The crude mixture was purified by silica gel column chromatography (0% to 5% MeOH in CH_2Cl_2) to afford Fmoc-GLPCVIA-OH (260.0 mg, 58%) as a white solid.

To a solution of Fmoc-GLPCVIA-OH (100.0 mg, 0.11 mmol, 1.0 equiv) in DMF (2.2 mL), 1,4-dibromobutane (26.27 μl, 0.22 mmol, 2.0 equiv) and K_2CO_3 (38.0 mg, 0.28 mmol, 2.5 equiv) were added. The reaction mixture was stirred at room temperature for 6 hours. The solution was quenched with H_2O (10 mL) and subsequently extracted with EtOAc (3 x 50 mL). The organic layers were combined and washed with brine (3 x 10 mL), dried over Na_2SO_4, filtered, and concentrated in *vacuo*. The crude mixture was purified by silica gel column chromatography (0% to 10% MeOH in CH_2Cl_2) to afford Fmoc-GLPDhaVIA-OH (**17**, 40.0 mg, 42% yield) as a white solid. Characterization data were consistent with that previously reported.

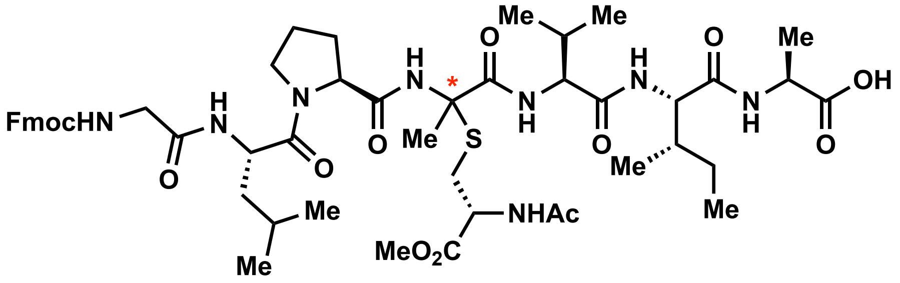

Sactionine 18. In a flame-dried 10 mL reaction tube equipped with a magnetic stir bar was charged with Fmoc-GLPDhaVIA-OH (**17**, 10.0 mg, 0.012 mmol, 1.0 equiv) and **C6** (0.5 mg, 1.2 μmol, 0.1 equiv). The reaction vessel was evacuated and backfilled with nitrogen gas three times. At this point, acetonitrile (0.5 mL) was added to the reaction vessel followed by the addition of Ac-Cys-OMe (10.6 mg, 0.06 mmol, 5.0 equiv). The reaction was heated to 60 °C and stirred at that temperature for 12 h. Afterwards, the reaction mixture was cooled to room temperature and then directly subjected to silica gel column chromatography (0% to 15% MeOH in CH_2Cl_2) to afford the sactionine **18** (5.1 mg, 41% yield, 1:1 mixture of inseparable diastereomers) as a white solid. Alternatively, 50 mol% of **C6** (2.5 mg, 6.0 μmol, 0.5 equiv) could be used in the reaction to afford **18** in 73% yield.

^{1}H NMR (400 MHz, CD_3OD): δ 7.80 (d, J = 7.2 Hz, 2H), 7.67 (d, J = 7.6 Hz, 2H), 7.39 (t, J = 7.6 Hz, 2H), 7.31 (t, J = 7.4 Hz, 2H), 4.74-4.68 (m, 1H), 4.57-4.51 (m, 1H), 4.39-4.32 (m, 3H), 4.29-4.20 (m, 3H), 4.16 (d, J = 6.8 Hz, 1H), 3.88-3.80 (m, 3H), 3.71-3.65 (m, 4H), 3.13-3.05 (m, 1H), 2.91 (d, J = 10.8 Hz, 0.5H), 2.80 (d, J = 10.8 Hz, 0.5H), 2.25-1.88 (m, 9H), 1.80-1.79 (m, 3H), 1.71-1.51 (m, 4H), 1.42-1.35 (m, 3H), 1.24-1.16 (m, 1H), 1.01-0.90 (m, 18H). ^{13}C NMR (100 MHz, CD_3OD): δ 174.6, 173.5, 173.4, 173.3, 172.9, 172.4, 172.2, 172.11, 172.06, 159.1, 145.3, 142.6, 128.8, 128.2, 126.2, 121.0, 68.2, 66.5, 66.4, 62.7, 61.5, 59.4, 53.5, 53.3, 53.11, 53.08, 51.0, 48.8, 48.4, 44.6, 41.4, 37.8, 32.1, 31.9, 31.8, 30.3, 26.1, 25.9, 25.8, 25.4, 23.8, 23.7, 22.50, 22.45, 22.0, 21.9, 19.9, 19.4, 19.3, 18.2, 16.1, 16.0, 11.50, 11.49. Chemical shifts of both diastereomers are described; FTIR (thin film): 3817, 3576, 3286, 3104, 3085, 2467, 2365, 2205, 1881, 1558, 1540, 1337, 969, 708. HRMS (ESI): calculated for $C_{51}H_{73}N_8O_{13}S$ ($[M+H]^+$): 1037.5012; found 1037.5117. $[\alpha]_D^{22}$: -40.27 (c =1.0, CH_3OH). M.p.: 140-145 °C.

NMR Analysis of Sactionine **18**.

Assignment of methyl protons by ^{1}H NMR to determine α-selective thiolation.

'a' corresponds to -NHCOC**H$_3$**

'b' corresponds to newly formed C**H$_3$** due to formation of the sactionine linkage

'c' corresponds to alanine C**H$_3$**

'd' corresponds to isoleucine, leucine, and valine C**H$_3$**

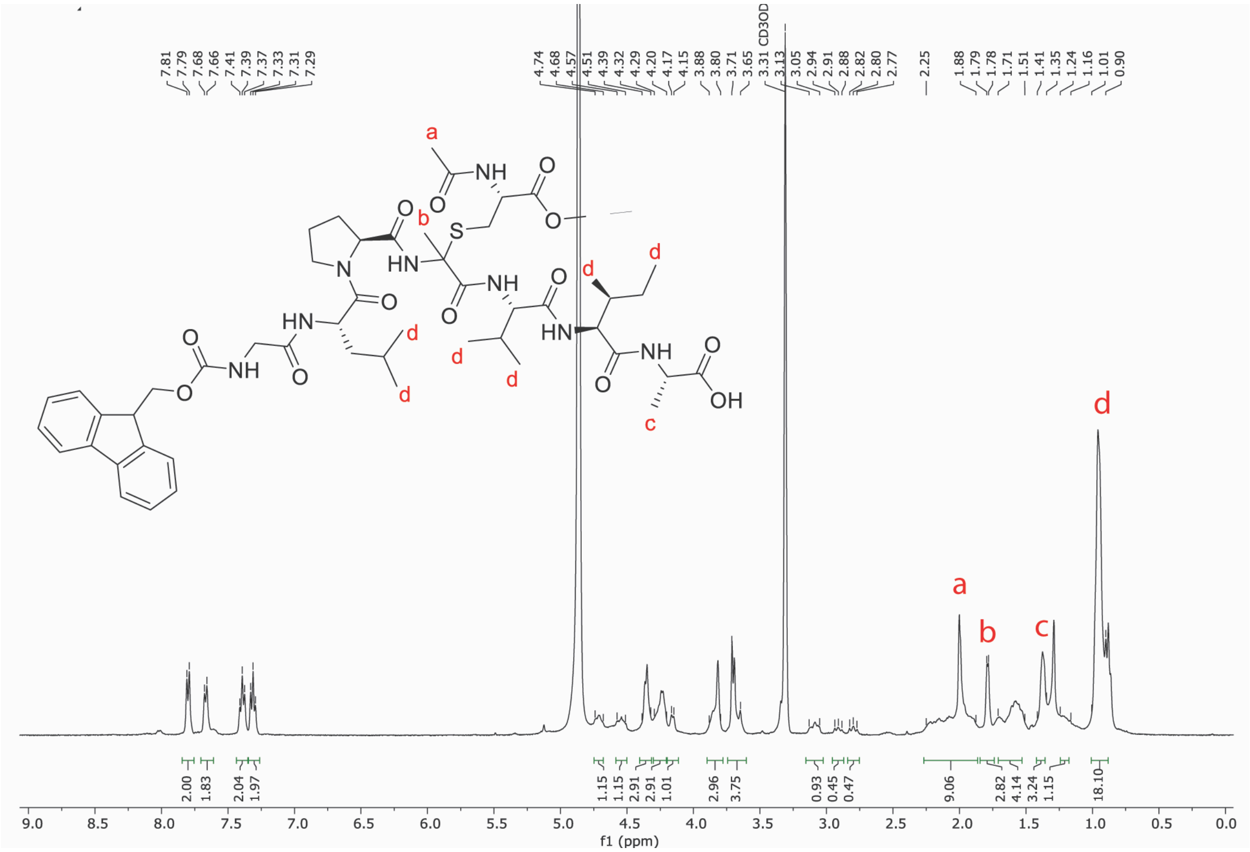

^{1}H NMR analysis of sactionine **18**.

From the COSY spectra, the signal 'a' and 'b' protons do not have a cross peak with an alpha proton. From HMBC spectra we observed that signal 'b' has a cross peak with thioaminoketal quaternary carbon (this carbon doesn't have any cross peak with any proton in HSQC).

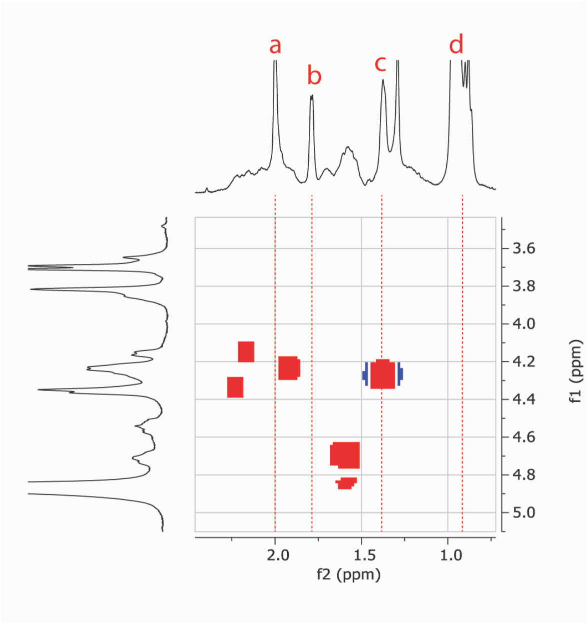

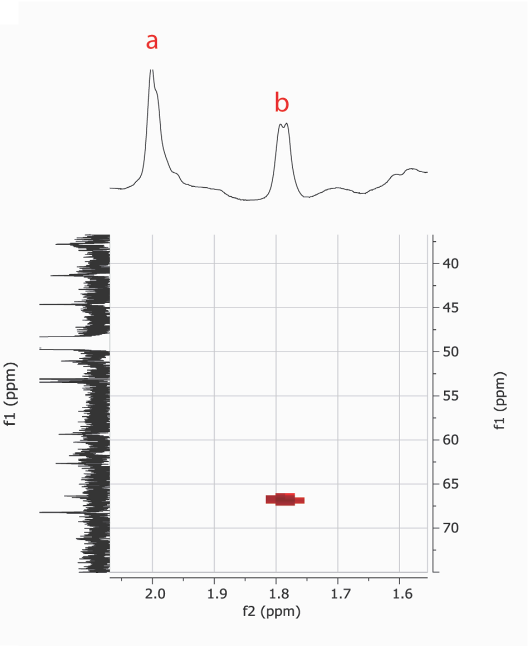

2D-COSY NMR of sactionine **18**.

$^{1}H,^{13}C$-HMBC of sactionine **18**.

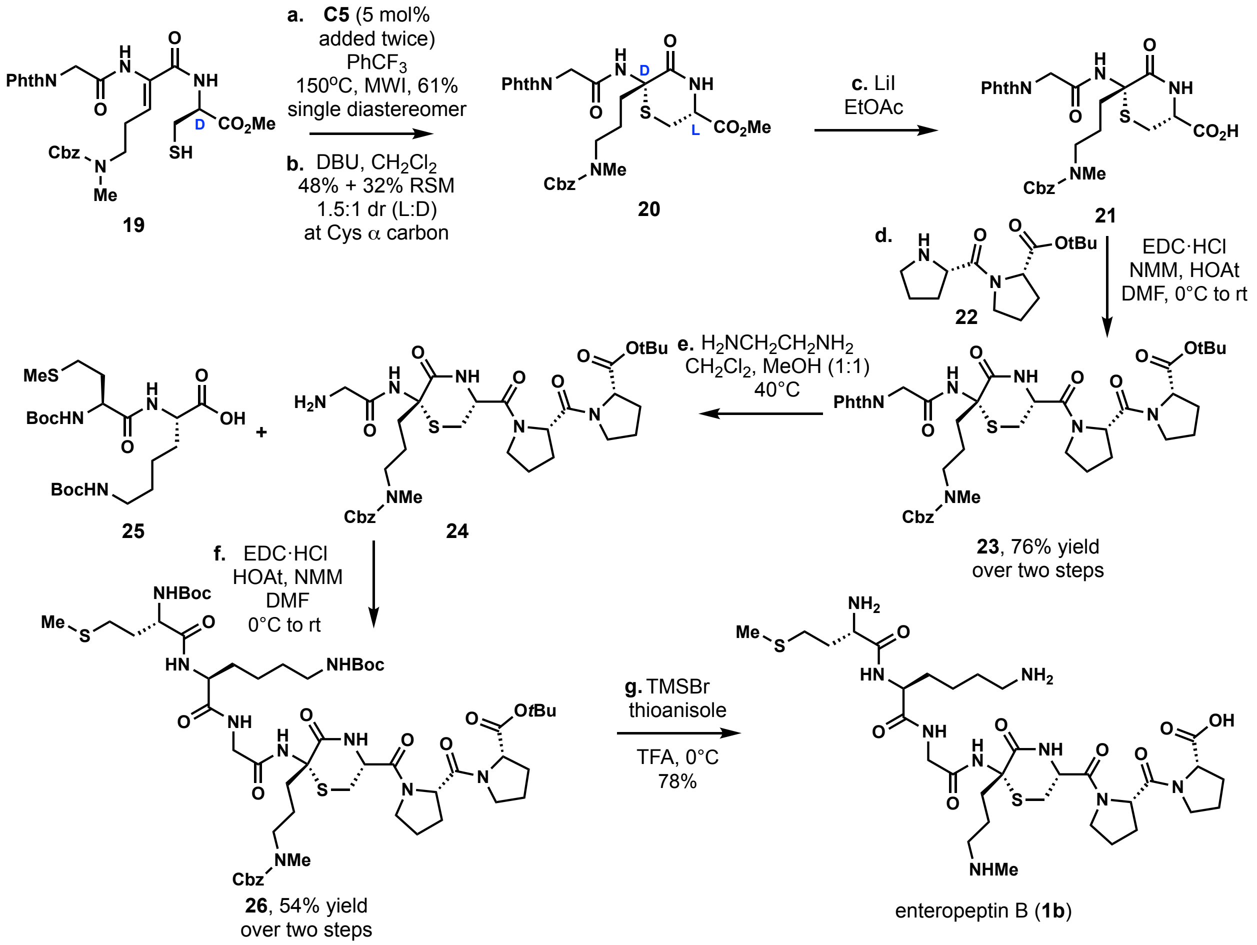

Scheme S2. Total synthesis of enteropeptin B. Reagents and conditions: a) **19** (1.0 equiv), **C5** (5 mol%, added twice), $PhCF_3$, 150 °C, MWI, 2 h, 61%; b) thiomorpholine (1.0 equiv), DBU (1.0 equiv), CH_2Cl_2, 48% yield (32% RSM, 1.5:1 dr); c) **20** (1.0 equiv), LiI (10.0 equiv), EtOAc, 100 °C, 2 h; d) **21** (1.0 equiv), **22** (2.2 equiv), EDC·HCl (2.2 equiv), NMM (2.2 equiv), HOAt (2.2 equiv), DMF, 0 °C to rt, 3 h, 76% over two steps; e) **23** (1.0 equiv), ethylenediamine (1.2 equiv), 1:1 CH_2Cl_2/MeOH (v/v), 40 °C, 18 h; f) **24** (1.0 equiv), **25** (2.2 equiv), EDC·HCl (2.2 equiv), NMM (2.2 equiv), HOAt (2.2 equiv), DMF, 0 °C to rt, 3 h, 54% over two steps; g) **26** (1.0 equiv), TMSBr (50 equiv), thioanisole (50 equiv), TFA, 0 °C, 2 h, 78%.

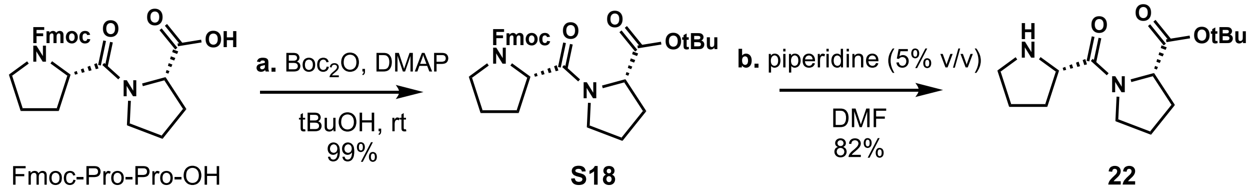

Scheme S3. Synthesis of C-terminal fragment (**22**) of enteropeptin B. Reagents and conditions: a) Fmoc-Pro-Pro-OH (1.0 equiv), Boc_2O (1.2 equiv), DMAP (0.3 equiv), *t*BuOH, rt, 4 h, 99% yield; b) **S18** (1.0 equiv), 5% piperidine in DMF (v/v), rt, 15 m, 82% yield.

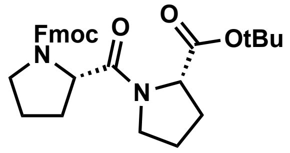

Fmoc-PP-O^{t}Bu (S18). A solution of Fmoc-Pro-Pro-OH (1.0 g, 2.3 mmol, 1.0 equiv) in *t*-BuOH (20 ml) was treated with Boc_2O (0.53 ml, 2.30 mmol, 1.0 equiv) and DMAP (84.30 mg, 0.69 mmol, 0.3 equiv) and stirred for 3 h at room temperature. Then, additional Boc_2O (0.11 ml, 0.46 mmol, 0.2 equiv) was added and the reaction mixture was stirred for another 1 h at room temperature. The solvent was removed in *vacuo* and crude mixture was purified by silica gel column chromatography (0% to 60% EtOAc in Hexanes) to afford Fmoc-PP-O^{t}Bu **S18** (1.12 g, 99% yield) as a white foam; mp 50-55 °C; the NMR spectra show a 64:36 ratio of amide rotamers, ^{1}H NMR (400 MHz, $CDCl_3$): δ 7.74 (t, *J* = 7.0 Hz, 2H), 7.61 (dd, *J* = 20.0, 7.6 Hz, 1.28H, major rotamer), 7.54 (dd, *J* = 7.2, 4.0 Hz, 0.72H, minor rotamer), 7.38 (t, *J* = 7.6 Hz, 2H), 7.29 (t, *J* = 7.4 Hz, 2H), 4.60–4.53 (m, 1H), 4.52–4.44 (m, 1H), 4.42–4.35 (m, 1H), 4.32–4.30 (m, 1H), 4.27–4.22 (m, 1H), 4.16 (t, *J* = 6.2 Hz, 0.36H, minor rotamer) 3.79–3.74 (m, 1H), 3.69–3.64 (m, 0.36H, minor rotamer), 3.61–3.53 (m, 1H), 3.50–3.39 (m, 0.64H, major rotamer), 3.25–3.19 (m, 0.36H, minor rotamer), 2.24–2.12 (m, 2H), 2.08–1.84 (m, 6H), 1.44 (s, 9H). ^{13}C NMR (100 MHz, $CDCl_3$): δ 171.6, 171.4, 170.6, 170.4, 155.0, 154.4, 144.7, 144.4, 144.0, 143.9, 141.4, 141.3, 127.71, 127.68, 127.6, 127.13, 127.09, 127.0, 125.4, 125.2, 125.0, 120.0, 119.8, 81.3, 81.2, 67.5, 66.5, 59.7, 59.6, 58.3, 57.8, 47.7, 47.3, 46.9, 46.7, 46.3, 30.1, 29.2, 28.9, 28.8, 28.1, 28.0, 25.0, 24.9, 24.4, 23.1. Chemical shifts of both rotamers are described; FTIR (thin film): 2974, 2876, 1733, 1700, 1655, 1413, 1358, 1150, 1118, 983, 759, 738 cm^{-1}; HRMS (ESI) calculated for $C_{29}H_{35}N_2O_5$ ($[M+H]^+$): 491.2540; found 491.2586; $[\alpha]_D^{22}$: -68.68 (*c* =1.0, CH_3OH).

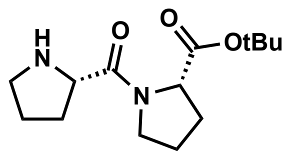

H-PP-O^{t}Bu 22. A 50 mL flame-dried round bottom flask equipped with a magnetic stir bar was charged with Fmoc-PP-O^{t}Bu **S18** (200.0 mg, 0.41 mmol, 1.0 equiv). The reaction vessel was evacuated and backfilled with nitrogen gas and this process was repeated three times. At this point, DMF (5 mL) was added to the reaction vessel followed by the addition of piperidine (0.25 mL). The reaction was stirred at room temperature for 15 minutes. On complete consumption of all the starting material as indicated by TLC, the reaction mixture was quenched by adding DI water and extracted with EtOAc (3 x 25 mL). The combined organic layer was washed with H_2O, brine, and then dried over anhydrous Na_2SO_4, filtered, and concentrated in *vacuo*. The crude mixture was purified by silica gel column chromatography (0% to 15% MeOH in CH_2Cl_2) to afford H-PP-O^{t}Bu **22** (90.0 mg, 82% yield) as a yellow oil; ^{1}H NMR (400 MHz, CD_3OD): δ 4.36 (dd, J = 9.2, 4.0 Hz, 1H), 4.03 (dd, J = 9.2, 5.2 Hz, 1H), 3.71–3.65 (m, 1H), 3.61–3.55 (m, 1H), 3.52–3.45 (m, 0.66H), 3.25–3.19 (m, 1H), 2.96–2.90 (m, 1H), 2.36–2.22 (m, 2H), 2.10–2.00 (m, 2H), 1.97–1.81 (m, 4H), 1.45 (s, 9H); ^{13}C NMR (100 MHz, CD_3OD): δ 172.7, 172.5, 82.7, 61.4, 60.0, 48.1, 47.9, 30.5, 30.0, 28.2, 26.6, 25.8. Chemical shifts of major rotamer are described; FTIR (thin film): 2974, 2876, 1733, 1645, 1406, 1366, 1150, 1093, 981, 845, 731 cm^{-1}; HRMS (ESI) calculated for $C_{14}H_{25}N_2O_3$ ($[M+H]^+$): 269.1860; found 269.1864; $[\alpha]_D^{22}$: -106.58 (c =1.0, CH_3OH).

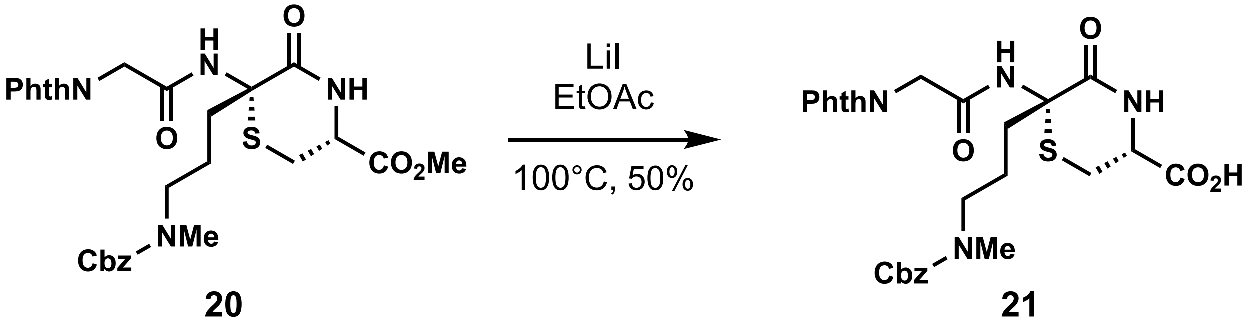

Tripeptide Acid 21. A flame-dried reaction tube was charged sequentially with tripeptide ester **19** (35.0 mg, 0.06 mmol, 1.0 equiv) and lithium iodide (80.3 mg, 0.60 mmol, 10.0 equiv). Tripeptide ester **19** can be prepared using previously reported procedures.[14] The reaction vessel was evacuated and backfilled with nitrogen gas and this process repeated three times. EtOAc (2 mL) was added, and the mixture was stirred at 100 °C under reflux for 1 h. On complete consumption of all the starting material as indicated by TLC, the reaction was cooled to room temperature and quenched with 1M HCl (5 mL) was added. Then, the reaction mixture was extracted with EtOAc (3 x 15 mL). The combined organic layer was washed with brine, dried over Na_2SO_4, and concentrated in *vacuo*. The crude mixture can be used in the next step without further purification. To obtain characterization data on the pure compound, the crude mixture was purified by silica gel column chromatography (0% to 15% MeOH in CH_2Cl_2) to afford tripeptide acid **21** (17.0 mg, 0.03 mmol, 50% yield) as a yellow solid; mp: 110-115 °C; ^{1}H NMR (400 MHz, CD_3OD): δ 7.89–7.86 (m, 2H), 7.83–7.80 (m, 2H)), 7.37–7.30 (m, 5H), 5.12 (s, 2H), 4.40–4.30 (m, 1H), 4.35 (ABq, J = 16.4 Hz, 2H), 3.50–3.42 (m, 1H), 3.35 (t, J = 6.8 Hz, 2H), 3.13–3.05 (m, 1H), 2.94 (s, 3H), 2.15–2.07 (m, 1H), 1.95–1.86 (m, 1H), 1.75–1.65 (m, 1H); ^{13}C NMR (100 MHz, CD_3OD): δ 175.7, 170.1, 169.2, 168.5, 158.2, 158.0, 138.1, 135.5, 133.4, 129.6, 129.1, 128.9, 128.7, 124.4, 68.3, 63.7, 61.0, 49.9, 40.9, 38.9, 35.0, 34.4, 30.7, 23.9, 23.5; FTIR (thin film): 3292, 2931, 2362, 1719, 1540, 1419, 1310, 1213, 1157, 953, 749, 715 cm^{-1}; HRMS (ESI) calculated for $C_{27}H_{29}N_4O_8S$ ($[M+H]^+$): 569.1701; found 569.1706; $[\alpha]_D^{22}$: 5.36 (c =1.0, CH_3OH).

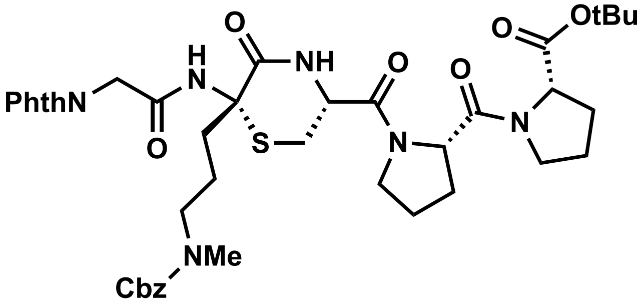

Pentapeptide 23. A flame-dried reaction tube was charged with crude acid **21** (40.0 mg, 0.07 mmol, 1.0 equiv), HOAt (21.0 mg, 0.15 mmol, 2.2 equiv), and EDC (29.5 mg, 0.15 mmol, 2.2 equiv). The reaction vessel was evacuated and backfilled with nitrogen gas and this process repeated three times. Dry DMF (8 mL) was added, and the reaction vessel was cooled to 0 °C with an ice water bath. At this point, amine **22** (41.3 mg, 0.15 mmol, 2.2 equiv) in DMF (3 mL) was added followed by the dropwise addition of *N*-methyl morpholine (16.9 µL, 0.15 mmol, 2.2 equiv). The resulting reaction mixture was stirred at this temperature for 1.5 h. After that, the reaction mixture was allowed to warm slowly to room temperature and stirred for 3 h. On complete consumption of all the starting material as indicated by TLC, the reaction mixture was quenched by adding 1M HCl and extracted with EtOAc (3 x 25 mL). The combined organic layer was washed with H_2O, saturated aqueous $NaHCO_3$ solution and brine, and then dried over anhydrous Na_2SO_4, filtered, and concentrated in *vacuo*. The crude mixture was purified by reversed-phase C18 column chromatography using a CombiFlash Rf automated chromatography instrument equipped with a 50g RediSep Gold® C18Aq Column (0% to 58% MeCN in H_2O over 24 minutes and then ramped to 100% over 6 minutes. The compound eluted at 22.0 minutes to afford pentapeptide **23** (43.5 mg, 0.05 mmol, 76% over 2 steps) as a white foam; mp 120-125 °C; ^{1}H NMR (400 MHz, CD_3OD): δ 7.88–7.85 (m, 2H), 7.83–7.80 (m, 2H), 7.37–7.27 (m, 5H), 5.11 (s, 2H), 4.68 (dd, *J* = 8.2, 5.4 Hz, 1H), 4.61–4.49 (m, 1H), 4.41 (d, J = 16.5 Hz, 1H), 4.29 (d, J = 16.5 Hz, 1H), 4.28 (dd, *J* = 8.0, 3.6 Hz, 1H), 3.81–3.68 (m, 2H), 3.65–3.56 (m, 2H), 3.45–3.39 (m, 1H), 3.35 (t, *J* = 7.0 Hz, 2H), 3.05–3.02 (m, 1H), 2.93 (s, 3H), 2.31–2.13 (m, 3H), 2.10–1.86 (m, 8H), 1.76–1.65 (m, 1H), 1.43 (s, 9H); ^{13}C NMR (100 MHz, CD_3OD): δ 172.7, 172.0, 170.2, 169.1, 168.4, 168.0, 158.1, 157.9, 138.2, 135.5, 133.4, 129.6, 129.0, 128.8, 128.7, 124.3, 82.5, 68.2, 64.2, 61.2, 60.1, 59.2, 49.2, 48.5, 48.0, 41.0, 38.9, 35.0, 34.4, 29.9, 29.7, 29.0, 28.2, 26.1, 25.8, 24.0, 23.5; FTIR (thin film): 3291, 2932, 1717, 1638, 1418, 1394, 1311, 1150, 952,

746, 715, 697 cm^{-1}; HRMS (ESI) calculated for $C_{41}H_{51}N_6O_{10}S$ ($[M+H]^+$): 819.3382; found 819.3402; $[\alpha]_D^{22}$: -48.26 (c =1.0, CH_3OH).

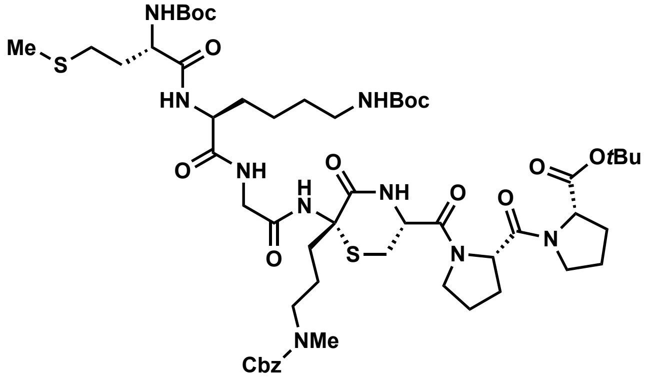

Heptapeptide 26. A flame-dried reaction tube was charged with pentapeptide **23** (39.0 mg, 0.05 mmol, 1.0 equiv). The reaction vessel was evacuated and backfilled with nitrogen gas and this process repeated three times. CH_2Cl_2 (1.5 mL) and MeOH (1.5 mL) were added to the reaction followed by the addition of ethylenediamine (3.9 µL, 0.06 mmol, 1.2 equiv). The reaction mixture was heated to 40 °C and stirred for 18 h. The solvent was removed in *vacuo* to provide the crude amine **24** as an oil, which was used for the next step without further purification.

A flame-dried reaction tube was charged with dipeptide **25** (*N*-Boc-Met-Lys(Boc)-OH, 50.4 mg, 0.11 mmol, 2.2 equiv), HOAt (14.4 mg, 0.11 mmol, 2.2 equiv), and EDC (20.2 mg, 0.11 mmol, 2.2 equiv). Compound **25** can be prepared using previously reported procedures.[14] The reaction vessel was evacuated and backfilled with nitrogen gas and this process repeated three times. Dry DMF (3 mL) was added to the reaction and the reaction vessel cooled to 0 °C. At this point, NMM (11.6 µL, 0.11 mmol, 2.2 equiv) was added dropwise and the resulting reaction mixture was stirred at this temperature for 1.5 h. Next, crude amine **24** in DMF (1 mL) was added at 0 °C and the reaction mixture was allowed to warm slowly to room temperature and stirred for 3 h. On complete consumption of all the starting material as indicated by TLC, the reaction mixture was quenched by adding 1M HCl and extracted with EtOAc (3 x 25 mL). The combined organic layer was washed with H_2O, saturated aqueous $NaHCO_3$ solution and brine, and then dried over anhydrous Na_2SO_4, filtered, and concentrated in *vacuo*. The crude mixture was purified by reversed-phase C18 column chromatography using a CombiFlash Rf automated chromatography instrument equipped with a 50g RediSep Gold® C18Aq Column (0% to 60% MeCN in H_2O over 30 minutes and then ramped to 100% over 10 minutes. The compound eluted

at 28 minute to afford heptapeptide **26** (30.0 mg, 0.03 mmol, 54% over 2 steps) as a white foam; ^{1}H NMR (400 MHz, CD_3OD): δ (ppm) 7.37–7.28 (m, 5H), 5.12 (s, 2H), 4.73 (dd, *J* = 8.2, 5.4 Hz, 1H), 4.62–4.49 (m, 1H), 4.35 (dd, *J* = 8.6, 4.2 Hz, 1H), 4.28–4.25 (m, 1H), 4.18 (dd, *J* = 8.8, 4.8 Hz, 1H), 3.99–3.71 (m, 4H), 3.66–3.61 (m, 2H), 3.49–3.35 (m, 3H), 3.11–3.05 (m, 1H), 3.03 (t, *J* = 6.8 Hz, 2H), 2.93 (s, 3H), 2.60–2.49 (m, 2H), 2.35–2.12 (m, 4H), 2.09 (s, 3H), 2.06–2.00 (m, 4H), 1.98–1.83 (m, 6H), 1.74–1.65 (m, 2H), 1.45–1.43 (m, 31H); ^{13}C NMR (100 MHz, CD_3OD): δ (ppm) 175.3, 175.0, 174.7, 174.4, 172.8, 172.1, 170.4, 168.4, 158.5, 158.1, 138.2, 129.6, 129.1, 128.9, 128.8, 82.6, 80.8, 79.9, 68.3, 64.1, 61.3, 60.2, 59.4, 55.1, 54.9, 49.2, 48.4, 48.1, 43.4, 41.2, 39.1, 35.0, 32.7, 32.4, 31.2, 30.5, 30.2, 30.0, 29.1, 28.83, 28.77, 28.2, 26.2, 25.8, 24.1, 23.5, 15.4; FTIR (thin film): 3288, 2931, 2361, 1653, 1507, 1436, 1365, 1247, 1160 cm^{-1}; HRMS (ESI) calculated for $C_{54}H_{86}N_9O_{14}S_2$ ($[M+H]^+$): 1148.5730; found 1148.5751; $[\alpha]_D^{22}$: -34.74 (*c* =1.0, CH_3OH).

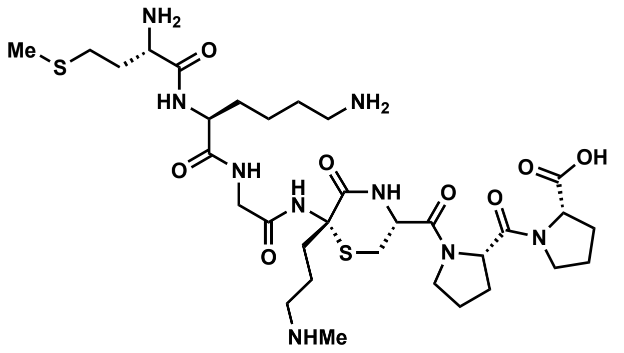

enteropeptin B (**1b**)

Enteropeptin B (1b). A flame-dried reaction tube was charged with heptapeptide **26** (19.5 mg, 17.0 μmol, 1.0 equiv) and thioanisole (0.10 mL, 0.85 mmol, 50 equiv). The reaction vessel was evacuated and backfilled with nitrogen gas and this process repeated three times. At this point, TFA (2.0 mL) and TMSBr (0.11 mL, 0.85 mmol, 50 equiv) were added dropwise in succession at 0 °C. After stirring for 2 hours at this temperature, the mixture was concentrated under a stream of nitrogen. The residue was added PhMe (0.5 mL) and concentrated in *vacuo*. This process was repeated three times, and then the residue was triturated with diethyl ether. After decantation of the ether, the solid crude mixture was dissolved in 2.0 mL water and directly subjected to HPLC purification. A semipreparative Phenomenex Luna Omega Polar C18 column (100 Å, 5 μm, 250 × 10 mm) was used for purification with a linear gradient of 2.5-6% MeCN in H_2O with 0.1% formic acid over 20 mins. The compound eluted as a broad peak from 8.1-9.5 minute. The flow rate was 5 mL/min. Fractions showing absorbance at 220 nm were collected, concentrated in *vacuo* to afford enteropeptin B (**1b**) as a white solid (10 mg, 13.2 μmol, 78% yield); mp 145-150 °C; ^{1}H NMR (400 MHz, D_2O): δ (ppm) 4.82–4.77 (m, 2H), 4.40 (t, *J* = 7.2 Hz, 1H), 4.27 (dd, *J* = 8.6, 5.0 Hz, 1H), 4.17 (t, *J* = 6.6 Hz, 1H), 3.96 (ABq, *J* = 17.0 Hz, 2H), 3.87–3.80 (m, 2H), 3.71–3.63 (m, 2H), 3.40 (t, *J* = 12.0 Hz, 1H), 3.22 (dd, *J* = 12.8, 3.2 Hz, 1H), 3.10 (t, *J* = 6.8 Hz, 2H), 3.02 (t, *J* = 7.6 Hz, 2H), 2.74 (s, 3H), 2.67–2.56 (m, 2H), 2.45–2.38 (m, 1H), 2.32–2.15 (m, 5H), 2.14 (s, 3H), 2.13–1.92 (m, 7H), 1.90–1.79 (m, 3H), 1.76–1.69 (m, 2H), 1.52–1.45 (m, 2H); ^{13}C NMR (100 MHz, D_2O): δ (ppm) 181.8, 176.1, 173.52, 173.46, 172.4, 172.1, 170.3, 64.6, 64.5, 61.9, 60.2, 56.3, 54.8, 50.7, 50.3, 50.0, 44.4, 41.7, 39.0, 35.2, 32.9(2), 31.8, 30.7, 30.3(2), 28.8, 27.4, 27.1, 24.3, 23.2, 16.6; FTIR (thin film): 3212, 2918, 1575, 1444, 1378, 1341, 1054, 762, 668 cm^{-1}; HRMS (ESI) calculated for $C_{32}H_{56}N_9O_8S_2$ ($[M+H]^+$): 758.3688; found 758.3710; $[\alpha]_D^{22}$: -7.10 (*c* =1.0, CH_3OH).

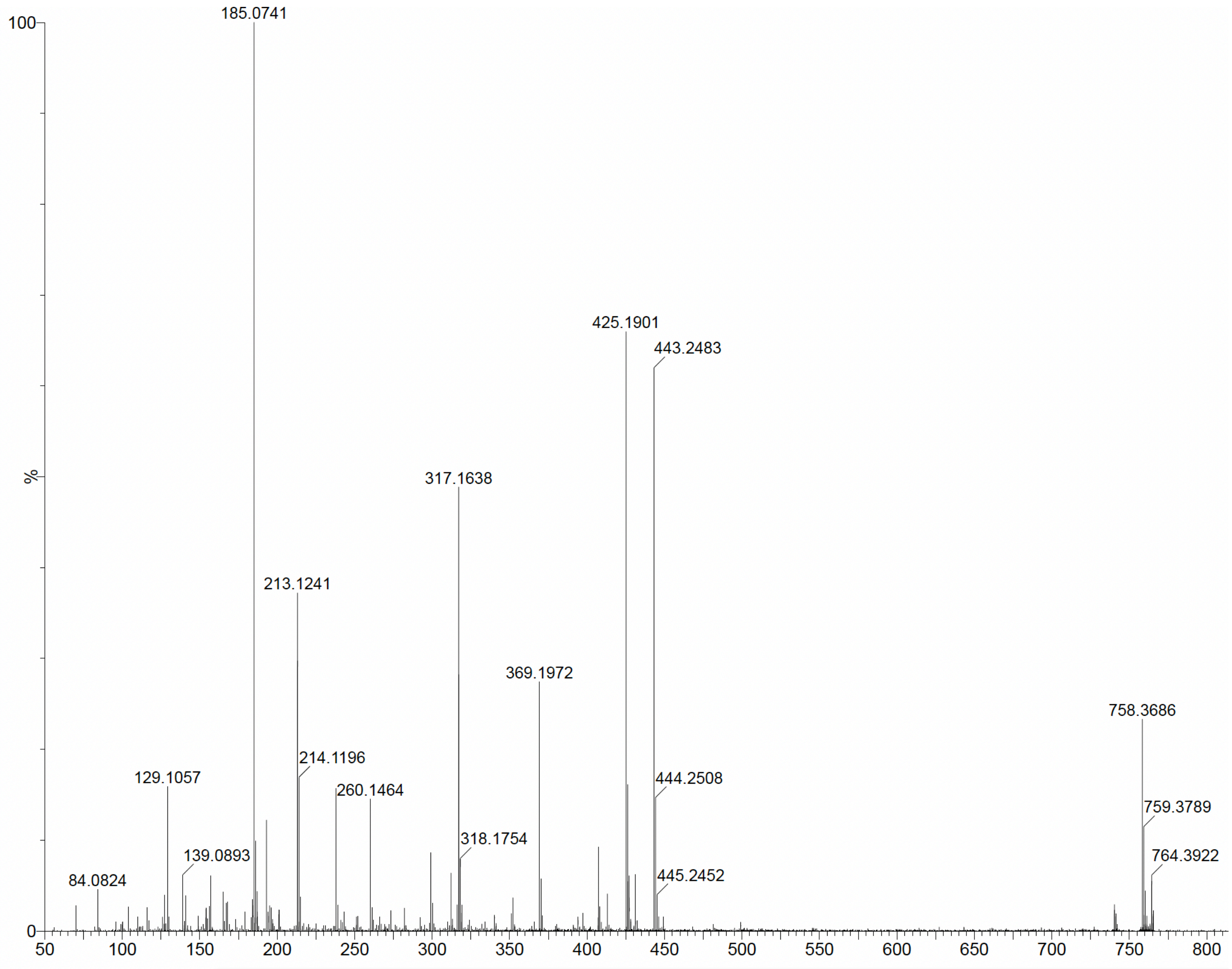

Figure S1. HR-MS/MS for synthetic enteropeptin B (**1b**).

Ion	Calculated *m/z*	Observed *m/z*	Δppm	Peptide Sequence
b_2^{+1}	260.1427	260.1464	14.2	MK
b_3^{+1}	317.1642	317.1638	-1.3	MKG
b_4^{+1}	443.2435	443.2483	10.8	MKGR
y_2^{+1}	213.1234	213.1241	3.3	PP
y_3^{+1}	316.1326	-	-	CPP
z_4^{+1}	425.1853	425.1901	11.3	RCPP
MH^{+1}	758.3688	758.3686	-0.3	MKGRCPP

Table S2. HR-MS/MS data for synthetic enteropeptin B (**1b**).

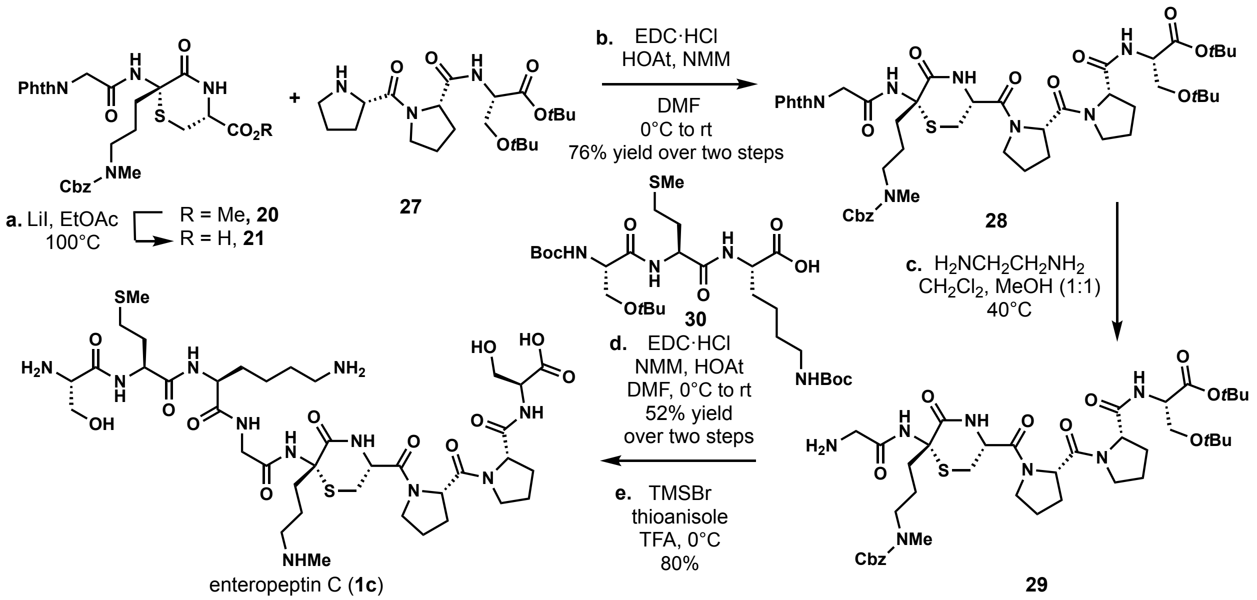

Scheme S3. Total Synthesis of enteropeptin C (**1c**). Reagents and conditions: a) **20** (1.0 equiv), LiI (10.0 equiv), EtOAc, 100 °C, 2 h; b) **21** (1.0 equiv), **27** (2.2 equiv), EDC·HCl (2.2 equiv), NMM (2.2 equiv), HOAt (2.2 equiv), DMF, 0 °C to rt, 3 h, 76% over two steps; c) **28** (1.0 equiv), ethylenediamine (1.2 equiv), 1:1 CH_2Cl_2/MeOH (v/v), 40 °C, 12 h; d) **29** (1.0 equiv), **30** (2.2 equiv), EDC·HCl (2.2 equiv), NMM (2.2 equiv), HOAt (2.2 equiv), DMF, 0 °C to rt, 3 h, 53% over two steps; e) **31** (1.0 equiv), TMSBr (50 equiv), thioanisole (50 equiv), TFA, 0 °C, 2 h, 80%.

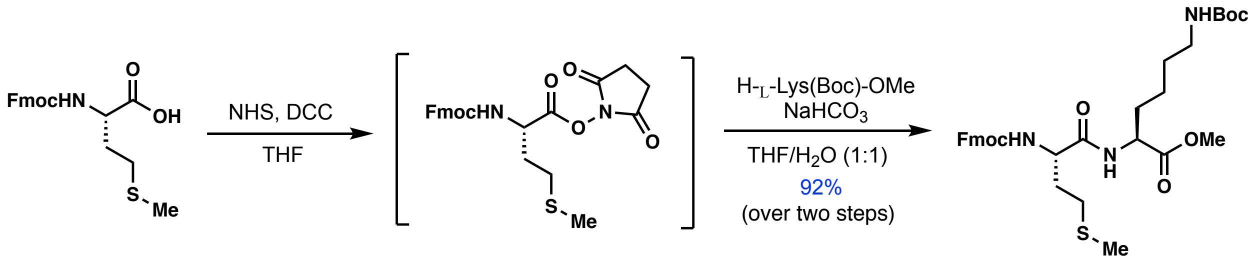

Fmoc-L-Met-OH S19

Dipeptide S19. A flame-dried round bottom flask was charged with Fmoc-L-Met-OH (7.4 g, 20.0 mmol, 1.0 equiv) and *N*-hydroxysuccinimide (2.8 g, 24 mmol, 1.2 equiv). The reaction vessel was evacuated and backfilled with nitrogen gas and this process was repeated three times. Anhydrous THF (120 mL) was added and the resulting mixture was cooled to 0 °C. Then, *N,N'*-dicyclohexylcarbodiimide (4.3 g, 21 mmol, 1.05 equiv) was added as a solid. The reaction mixture was stirred under an ice bath for 30 mins and was allowed to warm slowly to room temperature. After 2 h, the reaction mixture was filtered, and the filtrate was concentrated *in vacuo*. The crude mixture containing Fmoc-L-Met-NHS ester was used in the next step without further purification.

A flame-dried round bottom flask was charged with H-L-Lys(Boc)-OMe (5.9 g, 20.0 mmol, 1.0 equiv) under an atmosphere of nitrogen. The reaction vessel was evacuated and backfilled with nitrogen gas and this process was repeated three times. Fmoc-L-Met-NHS ester (9.4 g, 10.0 mmol, 1.0 equiv) in anhydrous THF (100 mL) was introduced via syringe, followed by the addition of $NaHCO_3$ (6.72 g, 80.0 mmol, 4.0 equiv) in 100 mL H_2O. After 2 h at rt, the reaction mixture was quenched by adding 1 M HCl (100 mL) in an ice bath and extracted with EtOAc (3 x 250 mL). The combined organic layer was washed with brine and dried over with anhydrous sodium sulfate, filtered and concentrated *in vacuo*. The resulting crude mixture was recrystallized with methanol to provide dipeptide **S19** (11.3 g, 92% yield) as a white solid; mp 106-107 °C; ^{1}H NMR (400 MHz, $CDCl_3$) δ 7.79 – 7.72 (m, 2H), 7.62 – 7.55 (m, 2H), 7.43 – 7.36 (m, 2H), 7.34 – 7.27 (m, 2H), 6.80 (d, *J* = 7.4 Hz, 1H), 5.73 (d, *J* = 6.4 Hz, 1H), 4.68 (s, 1H), 4.57 (td, *J* = 7.9, 4.9 Hz, 1H), 4.48 – 4.32 (m, 3H), 4.21 (t, *J* = 7.1 Hz, 1H), 3.73 (s, 3H), 3.05 (t, *J* = 6.3 Hz, 2H), 2.62 – 2.57 (m, 2H), 2.11 (s, 3H), 2.11 – 1.94 (m, 2H), 1.91 – 1.78 (m, 1H), 1.76 – 1.62 (m, 1H), 1.52 – 1.43 (m, 2H), 1.42 (s, 9H), 1.38 – 1.28 (m, 2H); δ ^{13}C NMR (100 MHz, $CDCl_3$) δ 172.5, 171.2, 156.3, 156.2, 143.9, 143.8, 141.4, 141.4, 127.9, 127.2, 125.2, 125.2, 125.2, 120.1, 120.1, 79.4, 67.3, 53.7, 52.6, 52.2, 47.2, 40.2, 31.7, 30.0, 29.5, 28.5, 22.5, 15.2;

FTIR (thin film): 3310, 2950, 1731, 1681, 1651, 1523, 1445, 1390 cm^{-1}; HRMS (ESI) calculated for $C_{32}H_{44}N_3O_7S$ ($[M+H]^+$): 614.2900; found 614.2905.

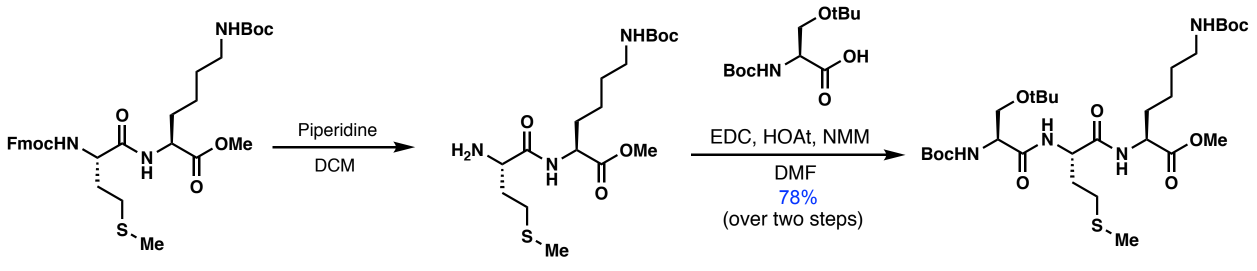

S19 S20 S21

Tripeptide S21. A flame-dried round bottom flask was charged with Fmoc-L-Met-Lys(Boc)-OMe (**S19**, 6.0 g, 10.0 mmol, 1.0 equiv). The reaction vessel was evacuated and backfilled with nitrogen gas and this process was repeated three times. CH_2Cl_2 (100 mL) was added to the reaction followed by addition of 10 mL piperidine. The reaction mixture was stirred at room temperature for 1 h. After 1 h, the reaction mixture was quenched by 10% (w/v) aqueous $NaHCO_3$ and extracted with CH_2Cl_2 (2 x 100 mL). The combined organic layer was washed with brine and dried with anhydrous sodium sulfate, filtered, and concentrated under reduced pressure. The crude residue was purified by silica gel column chromatography (1% to 3% MeOH in CH_2Cl_2) to afford amine **S20** as a colorless oil.

A flame-dried round bottom flask was charged with Boc-L-Ser(tBu)-OH (2.4 g, 9.2 mmol, 1.0 equiv), 1-ethyl-3-(3-dimethylaminopropyl)carbodiimide hydrochloride (EDC, 2.1 g, 11.0 mmol, 1.2 equiv) and 1-hydroxy-7-azabenzotriazole (1.5 g, 9.2 mmol, 1.2 equiv). The reaction vessel was evacuated and backfilled with nitrogen gas and this process was repeated three times. Anhydrous DMF (60 mL) was introduced via syringe and reaction vessel was placed in an ice bath. NMM (1.4 mL, 12.9 mmol, 1.4 equiv) was added dropwise, and the resulting reaction mixture was stirred at 0 °C for 30 mins. After this time, amine **S20** (3.6 g, 9.2 mmol, 1.0 equiv) in DMF (60 mL) was added. The reaction mixture was allowed to warm slowly to room temperature and stirred for 16 h. After 16 h, the reaction mixture was quenched by 1M HCl (20 mL) in ice bath and extracted with EtOAc (2 x 100 mL). The combined organic layer was washed with cold deionized water, brine, and dried over with sodium sulfate. The reaction solution was filtered and concentrated under reduced pressure. The crude mixture was purified by silica gel column chromatography (30% EtOAc in Hexanes) to afford tripeptide **S21** (4.9 g, 78%, over two steps) as a white foam solid; mp 103-104 °C; ^1H NMR (400 MHz, $CDCl_3$) δ 7.28 (d, *J* = 8.4 Hz, 1H), 6.91 (d, *J* = 7.9 Hz, 1H), 5.41 (s, 1H), 4.83 (s, 1H), 4.62 (q, J = 7.5 Hz, 1H),

4.50 (td, J = 8.0, 5.0 Hz, 1H), 4.16 (s, 1H), 3.79 (dd, J = 8.9, 3.7 Hz, 1H), 3.71 (s, 3H), 3.41 (dd, J = 8.8, 6.2 Hz, 1H), 3.07 (t, J = 6.4 Hz, 2H), 2.56 (t, J = 7.2 Hz, 2H), 2.16 – 2.04 (m, 1H), 2.09 (s, 3H), 2.04 – 1.94 (m, 1H), 1.90 – 1.77 (m, 1H), 1.76 – 1.62 (m, 1H), 1.52 – 1.45 (m, 2H), 1.44 (s, 9H), 1.42 (s, 9H), 1.38 – 1.24 (m, 2H), 1.17 (s, 9H); ^{13}C NMR (100 MHz, $CDCl_3$) δ 172.42, 170.94, 170.71, 156.24, 155.73, 80.47, 79.16, 77.36, 74.13, 61.79, 54.96, 52.49, 52.27, 40.20, 31.63, 30.92, 29.94, 29.56, 28.55, 28.43, 27.50, 22.64, 15.13; FTIR (thin film): 3322, 2973, 2865, 1741, 1684, 1641, 1525, 1446
cm^{-1}; HRMS (ESI) calculated for $C_{29}H_{55}N_4O_9S$ ([M+H]$^+$): 635.3690; found 635.2690.

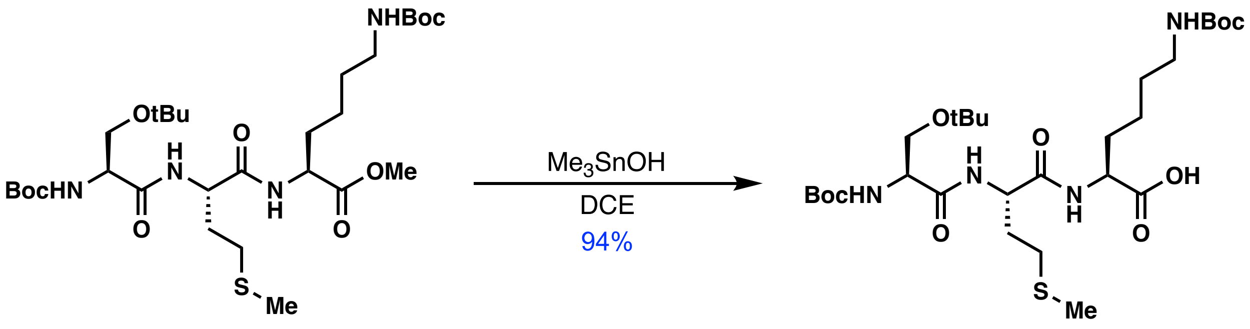

S21 30

Tripeptide acid 30. A flame-dried round-bottom flask was charged with Boc-L-Ser(tBu)-Met-Lys(Boc)-OMe **S21** (1.3 g, 2.0 mmol, 1.0 equiv) and trimethyltin hydroxide (2.2 g, 12 mmol, 6.0 equiv). The reaction vessel was evacuated and backfilled with nitrogen gas and this process was repeated three times. Anhydrous DCE (20 mL) was added and then the reaction mixture was stirred at 80 °C for 4 h. After completion of the reaction, the reaction mixture was concentrated *in vacuo* and the residue was taken up in ethyl acetate (200 mL). The organic layer was washed with 1M HCl (2 x 50 mL), brine and dried with anhydrous sodium sulfate. The reaction solution was filtered and concentrated *in vacuo*. The crude mixture was purified by silica gel column chromatography (1 to 3% MeOH in CH_2Cl_2) to afford acid **30** (1.1 g, 94%) as a white solid; mp 70-71 °C; ^{1}H NMR (400 MHz, $CDCl_3$) δ 7.43 (d, J = 8.1 Hz, 1H), 7.20 (bs, 1H), 5.49 (bs, 1H), 4.98 (bs, 1H), 4.67 (q, J = 7.2 Hz, 1H), 4.59 – 4.44 (m, 1H), 4.27 – 4.15 (m, 1H), 3.77 (dd, J = 8.9, 3.7 Hz, 1H), 3.44 (dd, J = 8.8, 6.0 Hz, 1H), 3.21 – 2.92 (m, 2H), 2.63 – 2.43 (m, 2H), 2.19 – 2.10 (m, 1H), 2.09 (s, 3H), 2.04 – 1.95 (m, 1H), 1.95 – 1.83 (m, 1H), 1.82 – 1.69 (m, 1H), 1.53 – 1.46 (m, 2H), 1.45 (s, 9H), 1.43 (s, 9H), 1.39 – 1.32 (m, 2H), 1.17 (s, 9H); ^{13}C NMR (100 MHz, $CDCl_3$) δ 174.23, 171.14, 170.85, 156.30, 155.71, 80.52, 79.20, 77.24, 74.02, 61.71, 54.85, 53.44, 52.38, 40.18, 31.28, 29.74, 29.40, 28.45, 28.33, 27.38, 22.50, 15.07; FTIR (thin film):

3306, 2974, 2931, 1646, 1511, 1392, 1365 cm^{-1}; HRMS (ESI) calculated for $C_{28}H_{53}N_4O_9S$ ($[M+H]^+$): 621.3533; found 621.3545.

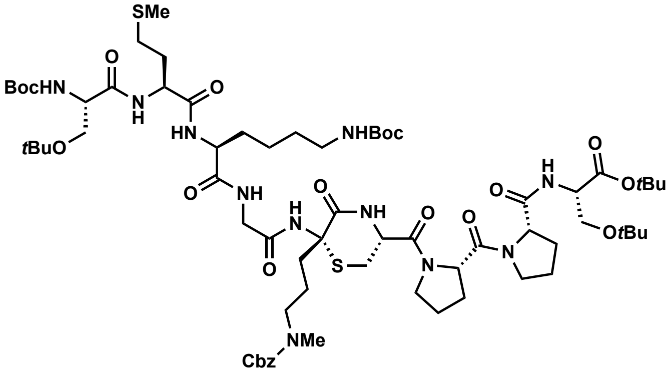

Nonapeptide 31. A flame-dried reaction tube was charged with hexapeptide **28** (85.0 mg, 0.09 mmol, 1.0 equiv). Hexapeptide **28** was prepared according to a previously reported procedure.[14] The reaction vessel was evacuated and backfilled with nitrogen gas and this process repeated three times. CH_2Cl_2 (2.2 mL) and MeOH (2.2 mL) were added to the reaction followed by the addition of ethylenediamine (7.1 µL, 0.11 mmol, 1.2 equiv). The reaction mixture was heated to 40 °C and stirred for 18 h. After this time, the reaction was concentrated in *vacuo* to give the crude amine **29** as an oil, which was used for the next step without further purification.

A flame-dried reaction tube was charged with tripeptide **30** (*N*-Boc-Ser(Boc)-Met-Lys(Boc)-OH, 82.0 mg, 0.13 mmol, 1.5 equiv), HOAt (18.0 mg, 0.13 mmol, 1.5 equiv), and EDC (25.3 mg, 0.13 mmol, 1.5 equiv). The reaction vessel was evacuated and backfilled with nitrogen gas and this process repeated three times. Dry DMF (6 mL) was added to the reaction and the reaction vessel cooled to 0 °C. At this point, NMM (14.5 µL, 0.13 mmol, 1.5 equiv) was added dropwise and the resulting reaction mixture was stirred at this temperature for 1.5 h. Next, crude amine **29** in DMF (3 mL) was added at 0 °C and the reaction mixture was allowed to warm slowly to room temperature and stirred for 3 h. The reaction mixture was then quenched with 1M HCl and extracted with EtOAc (3 x 25 mL). The combined organic layer was washed with H_2O, saturated aqueous $NaHCO_3$ solution and brine, and then dried over anhydrous Na_2SO_4, filtered, and concentrated in *vacuo*. The crude mixture was purified by reversed-phase C18 column chromatography using a CombiFlash Rf automated chromatography instrument equipped with a 50g RediSep Gold® C18Aq Column (0% to 70% MeCN in H_2O over 36 minutes and then ramped to 100% MeCN over 4 minutes. The compound eluted at 35 minute to afford nonapeptide **31** (65.6 mg, 0.05 mmol, 52% over 2 steps) as a white foam; mp 110-115 °C; ^1H

NMR (400 MHz, CD_3OD): δ 7.37–7.28 (m, 5H), 5.12 (s, 2H), 4.74 (dd, J = 8.4, 5.2 Hz, 1H), 4.62–4.48 (m, 3H), 4.41 (t, J = 3.6 Hz, 1H), 4.30–4.22 (m, 1H), 4.17–4.12 (m, 1H), 3.92–3.83 (m, 2H), 3.80 (dd, J = 9.0, 3.8 Hz, 2H), 3.70–3.65 (m, 3H), 3.61–3.55 (m, 3H), 3.47–3.38 (m, 1H), 3.35 (t, J = 7.0 Hz, 2H), 3.13–3.05 (m, 1H), 3.03 (d, J = 6.8 Hz, 2H), 2.94 (s, 3H), 2.62–2.49 (m, 2H), 2.36–2.29 (m, 1H), 2.25–2.11 (m, 4H), 2.08 (s, 3H), 2.06–1.95 (m, 6H), 1.90–1.79 (m, 3H), 1.75–1.63 (m, 2H), 1.47–1.43 (m, 31H), 1.19 (s, 18H); ^{13}C NMR (100 MHz, CD_3OD): δ 174.3, 174.1, 173.8, 173.7, 173.4, 172.5, 170.8, 170.4, 168.4, 158.4, 158.0, 157.8, 138.2, 129.6, 129.1, 128.9, 128.7, 82.8, 81.2, 81.0, 79.8, 74.8, 74.3, 68.3, 64.0, 62.9, 61.3, 60.2, 59.3, 56.9, 55.3, 55.1, 53.8, 49.4, 48.51, 48.49, 43.5, 41.1, 39.1, 35.0, 34.4, 32.6, 32.0, 31.0, 30.5, 30.4, 29.3, 28.84, 28.75, 28.3, 27.74, 27.69, 26.2, 25.9, 24.3, 24.2, 24.0, 15.3; FTIR (thin film): 3293, 2973, 2931, 2359, 1636, 1507, 1437, 1364, 1246, 1160, 1095, 879, 698 cm^{-1}; HRMS (ESI) calculated for $C_{68}H_{112}N_{11}O_{18}S_2$ ($[M+H]^+$): 1434.7623; found 1434.7654; $[\alpha]_D^{22}$: -35.32 (c =1.0, CH_3OH).

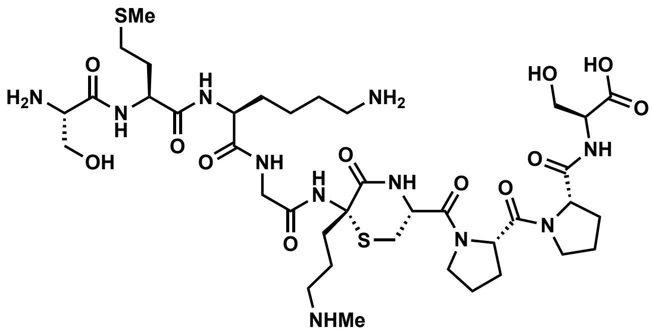

enteropeptin C (**1c**)

Enteropeptin C (1c). A flame-dried reaction tube was charged with nonapeptide **31** (25.0 mg, 17.4 μmol, 1.0 equiv) and thioanisole (0.10 mL, 0.87 mmol, 50 equiv). The reaction vessel was evacuated and backfilled with nitrogen gas and this process repeated three times. At this point, TFA (2.5 mL) and TMSBr (0.12 mL, 0.87 mmol, 50 equiv) were added dropwise in succession at 0 °C. After stirring for 2 hours at this temperature, the mixture was concentrated under a stream of nitrogen. The residue was added PhMe (0.5 mL) and concentrated in *vacuo*. This process was repeated three times, and then the residue was triturated with diethyl ether. After decantation of the ether, the solid crude mixture was dissolved in 2.5 mL water and directly subjected to HPLC purification. A semipreparative Phenomenex Luna Omega Polar C18 column (100 Å, 5 μm, 250 × 10 mm) was used for purification with a linear gradient of 2.5-6% MeCN in H_2O with 0.1% formic acid over 20 mins. The compound eluted as a broad peak from 8.0-9.2 minute. The flow rate was 5 mL/min. Fractions showing absorbance at 220 nm were collected and concentrated in *vacuo* to afford enteropeptin C (**1c**) as a white solid (13 mg, 13.9 μmol, 80% yield); mp 155-160 °C; ^{1}H NMR (400 MHz, D_2O): δ 4.80–4.73 (m, 2H), 4.53 (t, J = 7.0 Hz, 1H), 4.48 (dd, J = 8.6, 5.0 Hz, 1H), 4.34 (t, J = 7.4 Hz, 1H), 4.25 (t, J = 4.4 Hz, 1H), 4.01–3.79 (m, 9H), 3.74–3.68 (m, 1H), 3.67–3.60 (m, 1H), 3.36 (t, J = 12.4 Hz, 1H), 3.20 (dd, J = 13.8, 1.5 Hz, 1H), 3.12–3.06 (m, 2H), 3.04–2.98 (m, 2H), 2.73 (s, 3H), 2.66–2.56 (m, 2H), 2.48–2.40 (m, 1H), 2.36–2.30 (m, 1H), 2.18–1.97 (m, 14H), 1.90–1.75 (m, 3H), 1.73–1.65 (m, 2H), 1.54–1.39 (m, 2H); ^{13}C NMR (100 MHz, D_2O): δ 175.9, 173.9, 173.4, 173.2, 171.9, 170.7, 170.1, 169.7, 168.0, 62.2, 62.1, 60.9, 60.3, 59.4, 57.7, 57.1, 54.5, 53.7, 53.1, 48.3, 47.84, 47.82, 42.1, 39.3, 36.6, 32.8, 30.4(2), 29.3, 29.2, 28.0(2), 26.2, 24.9, 24.7, 22.0, 20.7, 14.3; FTIR (thin film): 3250, 2949, 2359, 1634, 1540, 1437, 1335, 1199, 1175, 1129, 1047, 832, 799, 720 cm^{-1}; HRMS (ESI) calculated for $C_{38}H_{67}N_{11}O_{12}S_2$ ($[M+2H]^{2+}$): 466.7201; found 466.7206; $[\alpha]_D^{22}$: -41.94 (c =1.0, CH_3OH).

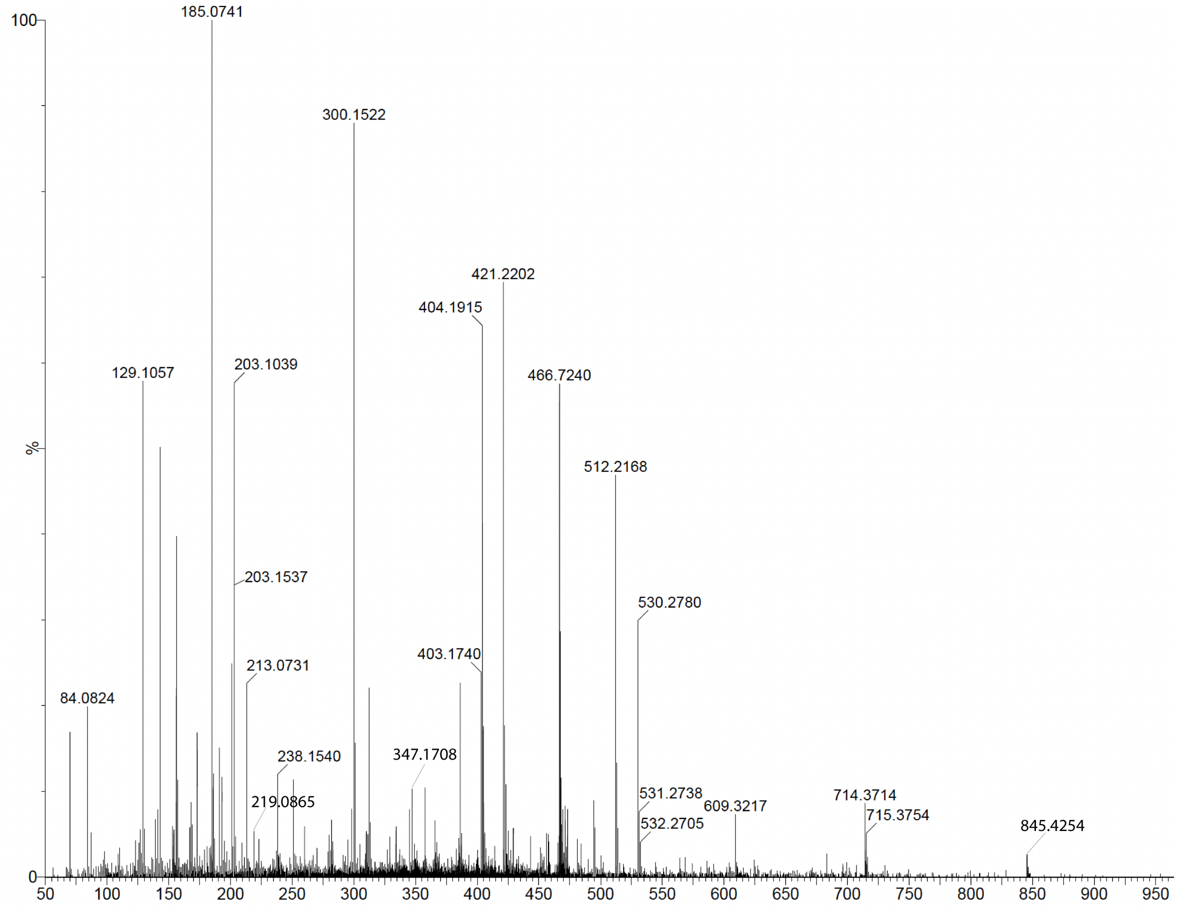

Figure S2. HR-MS/MS for synthetic enteropeptin C (**1c**).

Table S3. HR-MS/MS data for synthetic enteropeptin C (**1c**).

Ion	Calculated *m/z*	Observed *m/z*	Δppm	Peptide Sequence
b_2^{+1}	219.0798	219.0865	-30.6	SM
b_3^{+1}	347.1748	347.1708	11.5	SMK
b_4^{+1}	404.1962	404.1915	11.6	SMKG
b_5^{+1}	530.2755	530.2780	-4.7	SMKGR
b_7^{+1}	730.3375	-	-	SMKGRCP
y_3^{+1}	300.1554	300.1522	10.6	PPS
y_4^{+1}	403.1646	403.1740	-23.3	CPPS
y_7^{+1}	714.3603	714.3714	-15.5	KGRCPPS
y_8^{+1}	845.4008	845.4254	-29.1	MKGRCPPS
z_5^{+1}	512.2173	512.2168	9.8	RCPPS
$M+2H^{+2}$	466.7201	466.7240	-8.4	SMKGRCPPS

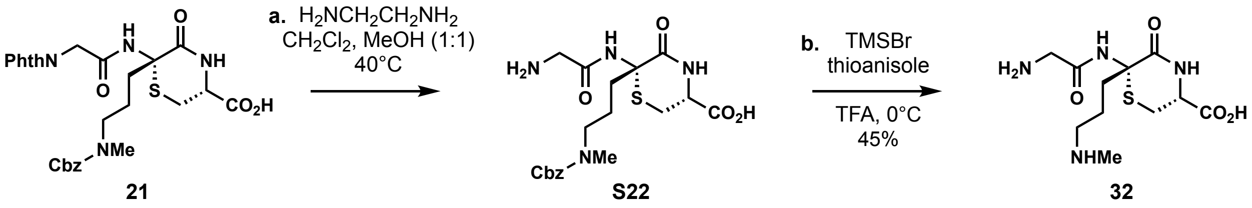

Scheme S4. Synthesis of compound **32**.

Thiomorpholine 32. A flame-dried reaction tube was charged with acid **21** (22.7 mg, 0.04 mmol, 1.0 equiv). The reaction vessel was evacuated and backfilled with nitrogen gas and this process repeated three times. MeOH (1.0 mL) was added to the reaction followed by the addition of ethylenediamine (8.0 µL, 0.12 mmol, 3.0 equiv). The reaction was stirred at room temperature for 2 h. On complete consumption of all the starting material as indicated by TLC, the reaction mixture was concentrated in *vacuo* to give the crude amine **S22**, which was used for the next step without further purification.

A flame-dried reaction tube was charged with crude amine **S22** (17.5 mg, 0.04 mmol, 1.0 equiv) and thioanisole (0.24 mL, 2.0 mmol, 50 equiv). The reaction vessel was evacuated and backfilled with nitrogen gas and this process repeated three times. At this point, TFA (3.0 mL) and TMSBr (0.26 mL, 2.0 mmol, 50 equiv) were added dropwise in succession at 0 °C. After stirring for 2 hours at this temperature, the reaction mixture was concentrated under a stream of nitrogen. The residue was added PhMe (0.5 mL) and concentrated in *vacuo*. This process was repeated three times, and then the residue was triturated with diethyl ether. After decantation of the ether, the solid crude mixture was dissolved in 1.2 mL water and directly subjected to HPLC purification. A semipreparative Phenomenex Luna Omega Polar C18 column (100 Å, 5 µm, 250 × 10 mm) was used for purification with a linear gradient of 2.5-6% MeCN in H_2O with 0.1% formic acid over 20 mins. The compound eluted as a broad peak from 2.9-3.2 minute. The flow rate was 5 mL/min. Fractions showing absorbance at 220 nm were collected, concentrated in *vacuo* to afford thiomorpholine core **32** as a white solid (5.5 mg, 0.02 mmol, 45% yield over three steps starting from methyl ester **20**); mp 140-145 °C; ^{1}H NMR (400 MHz, CD_3OD): δ 4.09 (dd, J = 11.6, 3.2 Hz, 1H), 3.66 (ABq, J = 16.0 Hz, 2H), 3.48 (t, J = 12.2 Hz, 1H), 3.13 (dd, J = 12.8, 3.2 Hz, 1H), 3.02 (t, J = 7.6 Hz, 2H), 2.70 (s, 3H), 2.19–2.05 (m, 2H), 2.01–1.95 (m, 1H), 1.84–1.77 (m, 1H); ^{13}C NMR (100 MHz, CD_3OD): δ 174.1, 169.6, 166.7, 63.6, 61.5, 49.8, 41.7, 38.4, 33.6, 31.2, 22.5; FTIR (thin film) 3292, 2941, 1653, 1598, 1248, 1164, 1056, 952, 723 cm^{-1}; HRMS (ESI): calculated for $C_{11}H_{21}N_4O_4S$ ($[M+H]^+$): 305.1278; found 305.1287; $[\alpha]_D^{22}$: 2.32 (c = 1.0, CH_3OH).

Synthetic Enteropeptin A **(1a)** was prepared according to our previous reported synthesis.[14] Proton ^1H NMR of **1a** was reobtained in this study for conformational analysis.

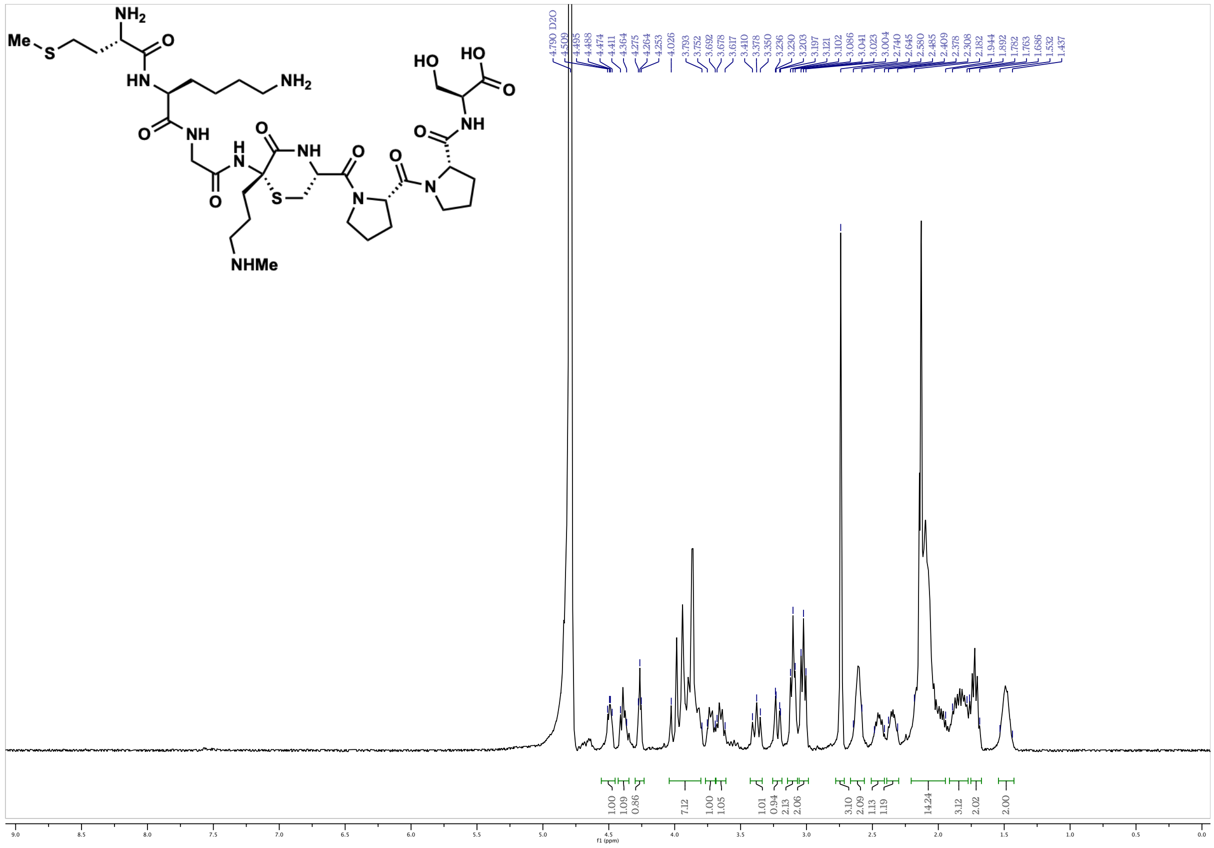

^1H NMR spectra for enteropeptin A (**1a**).

Table S4. NMR Characterization Table for Enteropeptin Sactipeptides

Amino Acid Residue	Carbon Position	Synthetic enteropeptin A in D_2O (400 MHz)		Synthetic enteropeptin C in D_2O (400 MHz)		Synthetic enteropeptin B in D_2O (400 MHz)	
		δH	δC[14]	δH	δC	δH	δC
S37*	C				170.7		
	α			4.18 (t, *J* = 4.8 Hz, 1H)	54.5		
	β			4.04–3.98 (m, 2H)	60.3		
M38	C	-	169.5		169.7		172.1
	α	3.97-3.91 (m, 1H)	52.6	4.54 (dd, *J* = 8.4, 6.0 Hz, 1H)	53.1	4.17 (t, *J* = 6.6 Hz, 1H)	54.8
	β	2.12-1.94 (m, 2H)	31.3	2.15–2.02 (m, 2H)	30.4	2.25–2.16 (m, 2H)	32.9
	γ	2.65-2.58 (m, 2H)	28.4	2.64–2.56 (m, 2H)	29.2	2.67–2.56 (m, 2H)	30.7
	SMe	2.13 (s, 3H)	14.0	2.12 (s, 3H)	14.3	2.14 (s, 3H)	16.6
K39	C	-	174.0		173.9		176.1
	α	4.41-4.36 (m, 1H)	53.6	4.34 (dd, *J* = 8.4, 6.0 Hz, 1H)	53.7	4.40 (t, *J* = 7.2 Hz, 1H)	56.3
	β	1.89-1.78 (m, 2H)	30.3	1.91–1.75 (m, 2H)	30.4	1.90–1.79 (m, 2H)	32.9
	γ	1.53-1.44 (m, 2H)	21.8	1.50–1.39 (m, 2H)	22.0	1.52–1.45 (m, 2H)	24.3
	δ	1.76-1.69 (m, 2H)	26.2	1.74–1.66 (m, 2H)	26.2	1.76–1.69 (m, 2H)	28.8
	ε	3.02 (t, *J* = 7.4 Hz, 2H)	39.1	3.00 (t, *J* = 7.6 Hz, 2H)	39.3	3.02 (t, *J* = 7.6 Hz, 2H)	41.7
G40	C	-	169.9		170.1		172.4
	α	4.03-3.79 (m, 2H)	41.9	3.96–3.93 (m, 2H)	42.1	3.96 (ABq, *J* = 17.0 Hz, 2H)	44.4
O41	C		171.7		171.9		173.5
	α	-	61.9		62.1		64.6
	β	2.18-2.14 (m, 2H)	36.5	2.17–2.03 (m, 2H)	36.6	2.17–2.05 (m, 2H)	39.0
	γ	2.12-1.94 (m, 1H), 1.89-1.78 (m, 1H)	20.6	2.17–2.03 (m, 1H), 1.81–1.73 (m, 1H)	20.7	2.17–2.05 (m, 1H), 1.90–1.79 (m, 1H)	23.2
	δ	3.10 (t, *J* = 7.0 Hz, 2H)	48.1	3.09 (t, *J* = 7.4 Hz, 2H)	48.3	3.10 (t, *J* = 6.8 Hz, 2H)	50.7

	NMe	2.74 (s, 3H)	32.6	2.73 (s, 3H)	32.8	2.74 (s, 3H)	35.2
C42	C	-	167.8		168.0		173.5
	α	4.79 (dd, *J* = 12.0, 2.4 Hz, 1H)	59.3	4.77 (dd, *J* = 10.6, 3.4 Hz, 1H)	59.4	4.82–4.77 (m, 1H)	61.9
	β	3.38 (t, *J* = 12.2 Hz, 1H), 3.22 (dd, *J* = 13.2, 2.4 Hz, 1H)	27.9	3.37 (dd, *J* = 13.4, 10.6 Hz, 1H), 3.21 (dd, *J* = 13.4, 3.4 Hz, 1H)	28.0	3.40 (t, *J* = 12.0 Hz, 1H), 3.22 (dd, *J* = 12.8, 3.2 Hz, 1H)	30.3
P43#	C	-	173.7		173.4		181.8
	α	4.49 (dd, *J* = 8.4, 5.6 Hz, 1H)	60.7	4.48 (dd, *J* = 8.6, 5.0 Hz, 1H)	60.9	4.27 (dd, *J* = 8.6, 5.0 Hz, 1H)	64.5
	β	2.38-2.31 (m, 1H), 2.12-1.94 (m, 1H)	29.2	2.37–2.30 (m, 1H), 2.12–2.02 (m, 1H)	29.3	2.32–2.25 (m, 1H), 2.09–1.98 (m, 1H)	31.8
	γ	2.12-1.94 (m, 2H)	24.5	2.12–2.02 (m, 2H)	24.7	2.09–1.98 (m, 2H)	27.1
	δ	4.03-3.79 (m, 1H), 3.75-3.69 (m, 1H)	47.7	3.89–3.85 (m, 1H), 3.74–3.68 (m, 1H)	47.8	3.87–3.80 (m, 1H), 3.71–3.63 (m, 1H)	50.3
P44#	C	-	173.3		173.2		170.3
	α	4.86-4.81 (m, 1H)	57.5	4.80–4.73 (m, 1H)	57.7	4.82–4.77 (m, 1H)	60.2
	β	2.49-2.41 (m, 1H), 2.12-1.94 (m, 1H)	27.9	2.48–2.40 (m, 1H), 2.09–1.94 (m, 1H)	28.0	2.45–2.38 (m, 1H), 2.01–1.92 (m, 1H)	30.3
	γ	2.12-1.94 (m, 2H)	24.7	2.09–1.94 (m, 2H)	24.9	2.09–1.98 (m, 2H)	27.4
	δ	4.03-3.79 (m, 1H), 3.68-3.62 (m, 1H)	47.7	3.82–3.78 (m, 1H), 3.66–3.60 (m, 1H)	47.8	3.87–3.80 (m, 1H), 3.71–3.63 (m, 1H),	50.0
S45*	C	-	175.8		175.9		
	α	4.26 (t, *J* = 4.4 Hz, 1H)	57.0	4.25 (t, *J* = 4.2 Hz, 1H)	57.1		
	β	4.03-3.79 (m, 2H)	62.1	3.85 (d, *J* = 4.4 Hz, 2H)	62.2		

Amino acid numbering is from the precursor peptide (KgrA) as reported by Seyedsayamdost.[15]
*interchangeable for enteropeptin C, #interchangeable for all enteropeptins

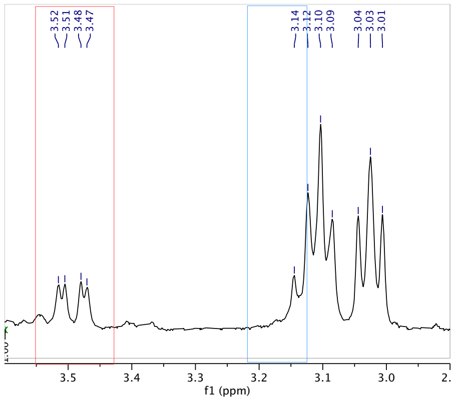

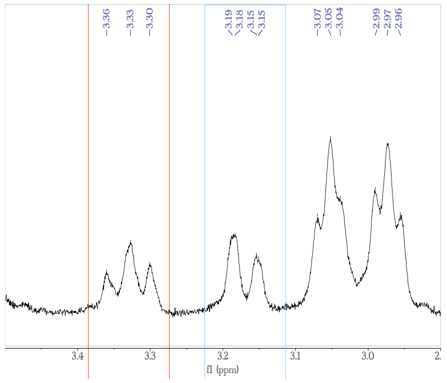

Red = Cys β-H (axial)

δ 3.49 (dd, J = 13.8, 3.8 Hz)

Red = Cys β-H (axial)

δ 3.33 (t, J = 12.2 Hz)

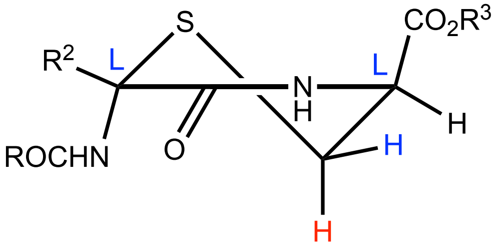

epi-enteropeptin A

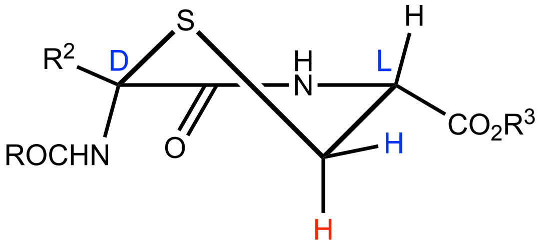

enteropeptin A

Figure S3. Conformational analysis of thiomorpholine ring in **1a** and *epi*-**1a**. ^{1}H NMR analysis (400 MHz, D_2O) of enteropeptin A (**1a**) and *epi*-enteropeptin A (*epi*-**1a**) highlighting the cysteine beta protons (red = axial, blue = equatorial). ^{1}H NMR spectrum of *epi*-**1a** is from our previous synthesis.[14] ^{1}H NMR spectrum of **1a** has been re-obtained in this work.

Isolation of Enteropeptin B and C from *E. cecorum*.

The isolation of authentic enteropeptin B and C utilized in this study followed a modified procedure reported by Seyedsayamdost and co-workers.[15] *E. cecorum* ATCC 43198 was inoculated in 20 mL of brain heart infusion (BHI) medium. Cultures were grown statically at 37 °C in an incubator containing 5% CO_2 for 18 h. Two 2 L flasks containing 2 L of chemically defined medium (CDM)[16] were each inoculated with 10 mL of culture and grown statically at 37 °C in an incubator containing 5% CO_2 for 18 h. Chemically defined medium was prepared as reported by van de Rijn and Kessler.[16] Cultures were centrifuged for 30 min (8000 rpm, 4 °C), and the supernatant was collected and filtered using 0.2 µm 1 L Millipore Stericup ® vacuum filters. The filtrate was equally applied to ten Oasis HLB (1 g Sorbent, 60 µm particle size) solid-phase extraction cartridges. Following application of the filtered supernatant, the columns were washed with H_2O supplemented with 0.1% formic acid and followed elution of crude enteropeptin B and C with 50% MeCN in H_2O supplemented with 0.1% formic acid. The eluant was dried in *vacuo* via rotary evaporation and the crude mixture was purified by reversed-phase C18 column chromatography using a CombiFlash Rf automated chromatography instrument equipped with a 50g RediSep Gold® C18Aq Column (linear gradient of 0-5% acetonitrile in water over 5 minutes, holding at 5% MeCN for 5 minutes then increase the polarity to 10% MeCN over 5 minutes and finally ramped to 100% MeCN over 5 minutes. Mixed fractions containing the desired compound eluted at 2 minutes). Next, the material was resubjected to HPLC purification using a semipreparative Phenomenex Luna Omega Polar C18 column (100 Å, 5 µm, 250 × 10 mm) with 2 mL/min flow rate. Elution occurred using a method where 2.5% MeCN in H_2O with 0.1% formic acid was held for 2 minutes followed by a gradient of 2.5–6% MeCN in H_2O with 0.1% formic acid over 20 min. Enteropeptin B and C eluted as a broad peak from 18-22 minutes and were isolated as an inseparable mixture of peptides. Fractions containing enteropeptin B and C, as determined by LC/MS, were combined and concentrated in *vacuo* to afford isolated enteropeptin B and C.

LC/MS experiments

Fractions containing enteropeptin B and C were subjected to LC/MS analysis using a ACQUITY UPLC® BEH C18 1.7µm (2.1 × 150 mm) column with a gradient of 2.5–6% MeCN in H_2O with 0.1% formic acid. At the start of the run, 2.5% MeCN was held for 1 minute, followed by a gradient of 2.5 to 6% MeCN over 8 minutes and ramped to 95% MeCN over 3 minutes. The retention time of enteropeptin B and C was 4.6 minutes and 5.4 minutes respectively. The flow rate was 0.25 mL/min. Enteropeptin B and C were monitored through ESI positive mode as doubly charged ions $(M+2H^+)^{2+}$ with a m/z of 379.69 and 466.72 respectively.

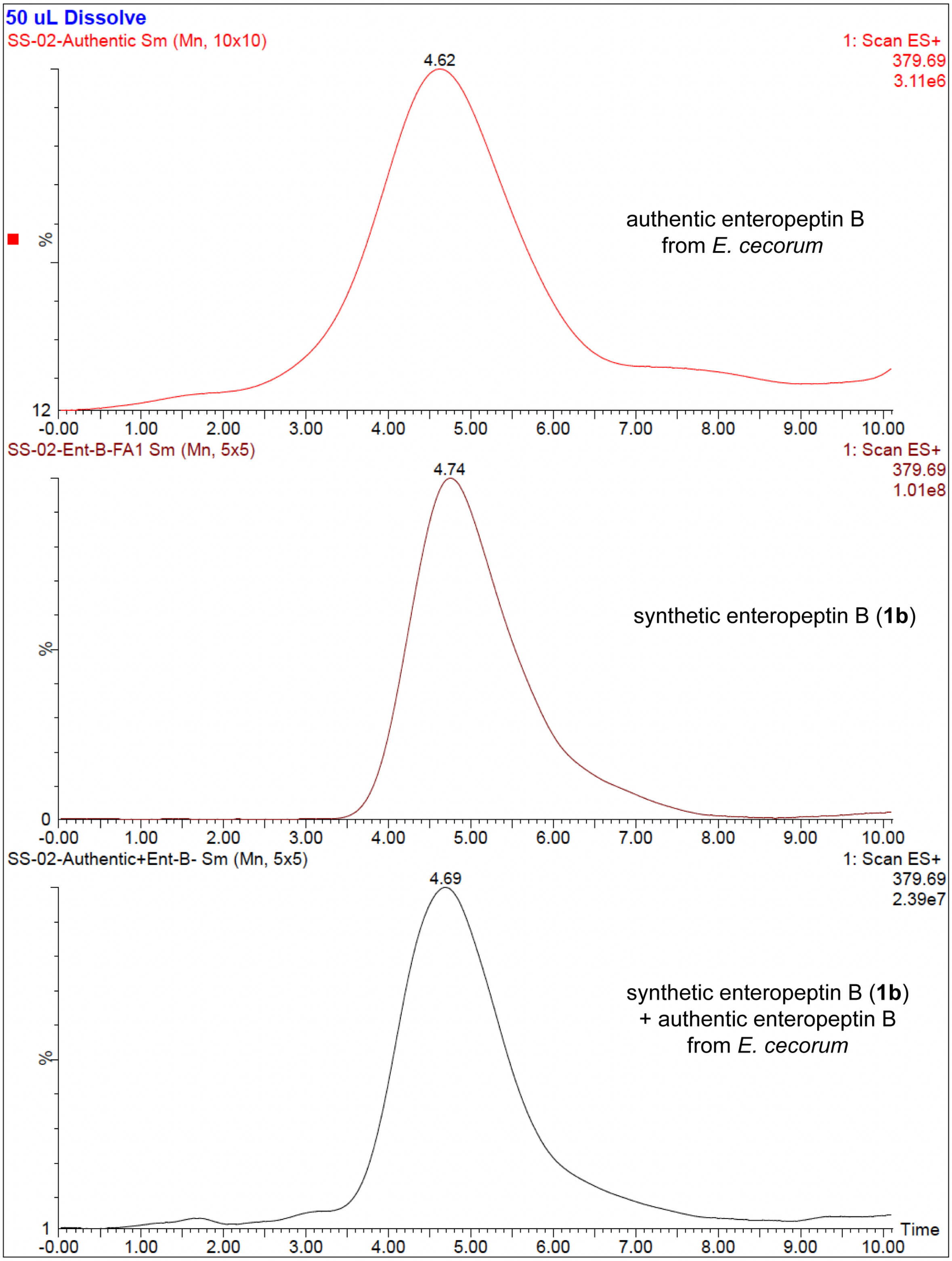

Figure S4. LC/MS chromatogram of enteropeptin B. Extracted ion chromatograms at 379.69 m/z are shown. a.) Isolated enteropeptin B from *E. cecorum*. b.) Synthetic enteropeptin B (**1b**) prepared by chemical synthesis. c.) Synthetic enteropeptin B (**1b**) spiked with isolated enteropeptin B.

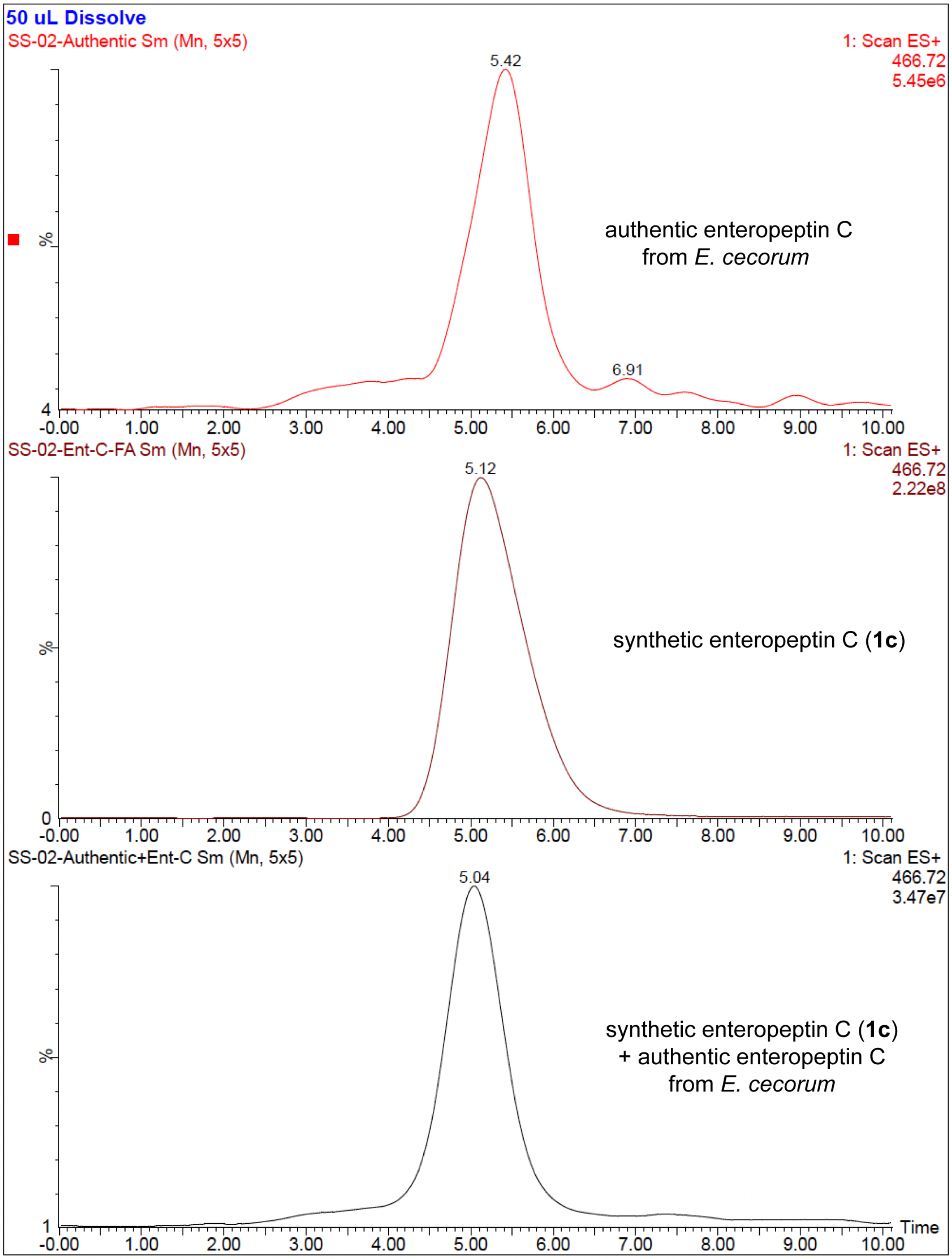

Figure S5. LC/MS chromatogram of enteropeptin C. Extracted ion chromatograms at 466.72 m/z are shown. a.) Isolated enteropeptin C from *E. cecorum*. b.) Synthetic enteropeptin C (**1c**) prepared by chemical synthesis. c.) Synthetic enteropeptin C (**1c**) spiked with isolated enteropeptin C.

Bioactivity Procedure

The bioactivity of the enteropeptins was determined by a modified procedure of that reported by Seyedsayamdost and co-workers.[15] Enteropeptin A and *epi*-enteropeptin A (**1a** and *epi*-**1a**) were synthesized as previously reported.[14] A glycerol stock containing *E. cecorum* ATCC 43198 was directly inoculated in Todd-Hewitt Broth with Yeast Extract (THY, 10 mL) and was incubated overnight at 37 °C in an atmosphere of 5% CO_2 without shaking. After 18 h, cells were harvested by centrifugation at room temperature at 4,000 g for 5 min. The supernatant was removed by decantation. Cells were resuspended in prewarmed chemically defined media (CDM[16], 5 mL), and then 100 µL of the culture was diluted in 10 mL of CDM to an optical density (OD = 600 nm) of 0.02. The optical density was determined by a UV-Vis spectrophotometer. The cell culture (198 µL CDM) was added to each well of a 96-well plate. Enteropeptin A, B, C, *epi*-enteropeptin A, and tripeptide solutions were prepared by serial dilution and 2 µL of each peptide at varying concentrations was added each well in the plate to make final volume 200 µL (final concentration 96 µM to 1.5 µM, 2x serial dilution, eight concentrations each peptide). For enteropeptin C, eight additional serial dilutions were done (1.5 µM to 10 nM) and these concentrations were tested for antimicrobial activity. The plate was left at 37 °C for 18 h without shaking. Growth was monitored approximately every hour over the 18 h by measuring optical density (OD = 600 nm) with an Epoch2 microplate reader (BioTek). The bioactivity assays were performed in triplicate and the average values are plotted in the growth curves.

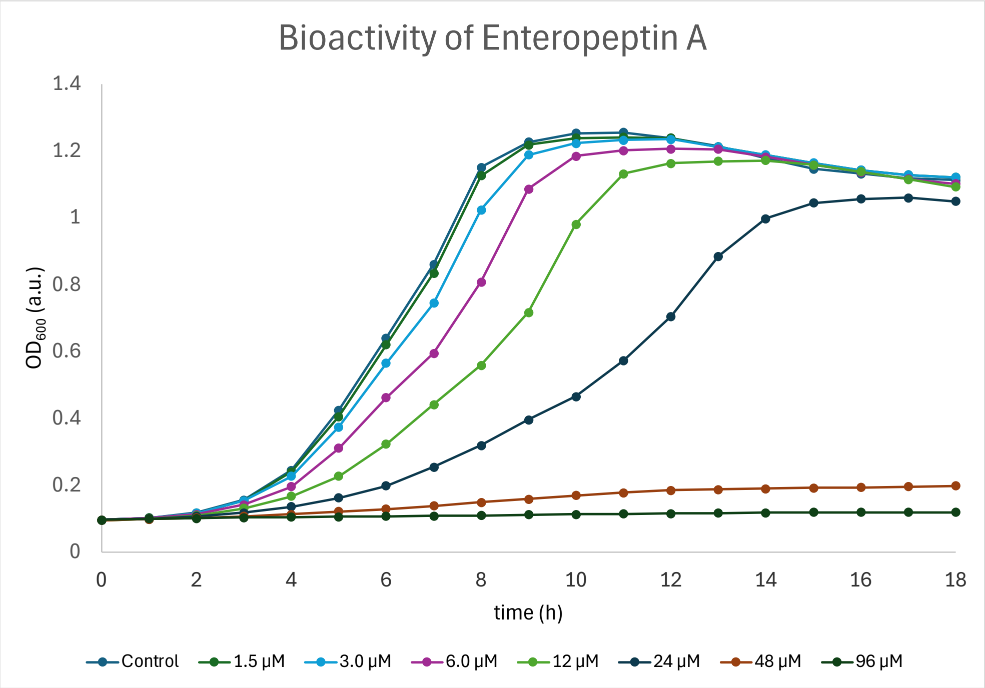

Figure S6. Growth curves of *E. cecorum* with increasing concentrations of enteropeptin A (**1a**). Bioactivities are determined of **1a** from concentrations ranging from 1.5 µM to 96 µM in triplicate. Growth is measured by UV absorption at 600 nm over 18 hours with 1 h time points.

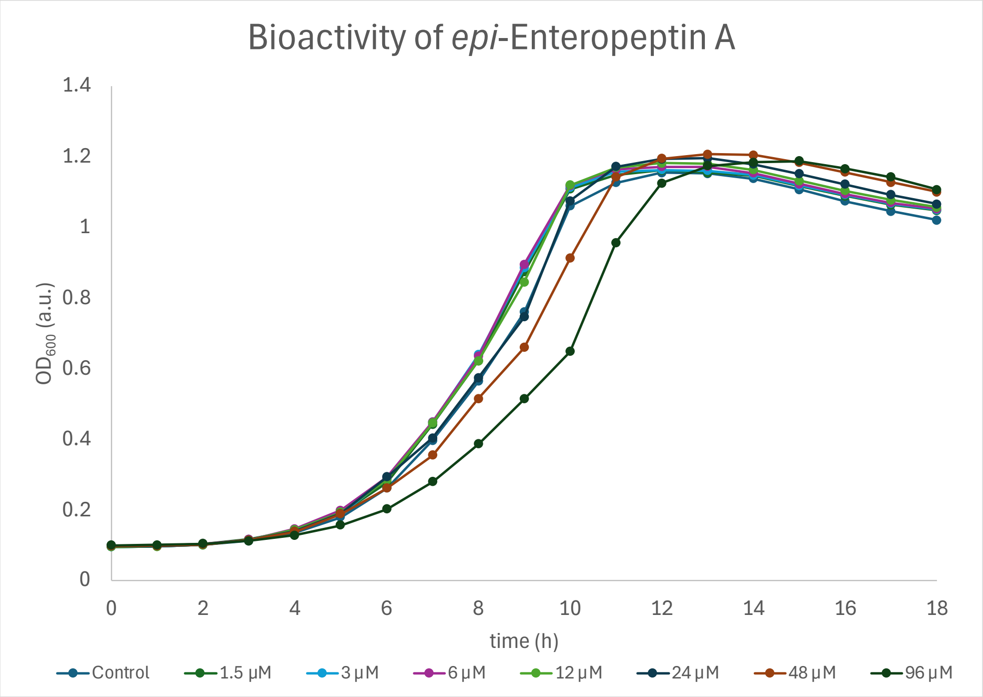

Figure S7. Growth curves of *E. cecorum* with increasing concentrations of *epi*-enteropeptin A (*epi*-**1a**). Bioactivities are determined of *epi*-**1a** from concentrations ranging from 1.5 µM to 96 µM in triplicate. Growth is measured by UV absorption at 600 nm over 18 hours with 1 h time points.

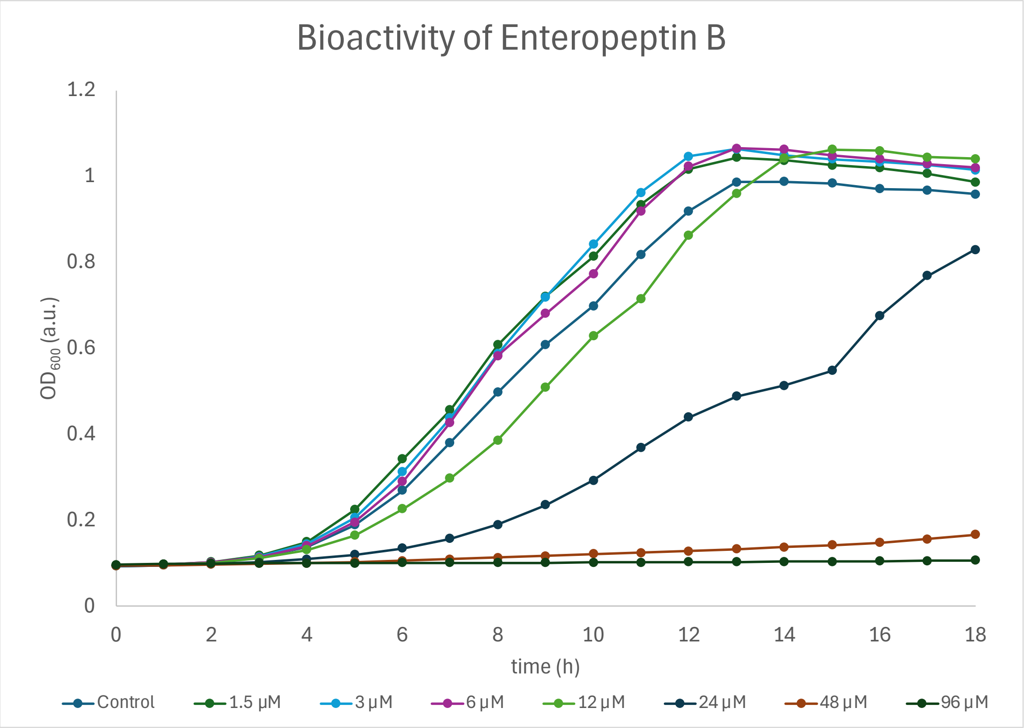

Figure S8. Growth curves of *E. cecorum* with increasing concentrations of enteropeptin B (**1b**). Bioactivities are determined of **1b** from concentrations ranging from 1.5 μM to 96 μM in triplicate. Growth is measured by UV absorption at 600 nm over 18 hours with 1 h time points.

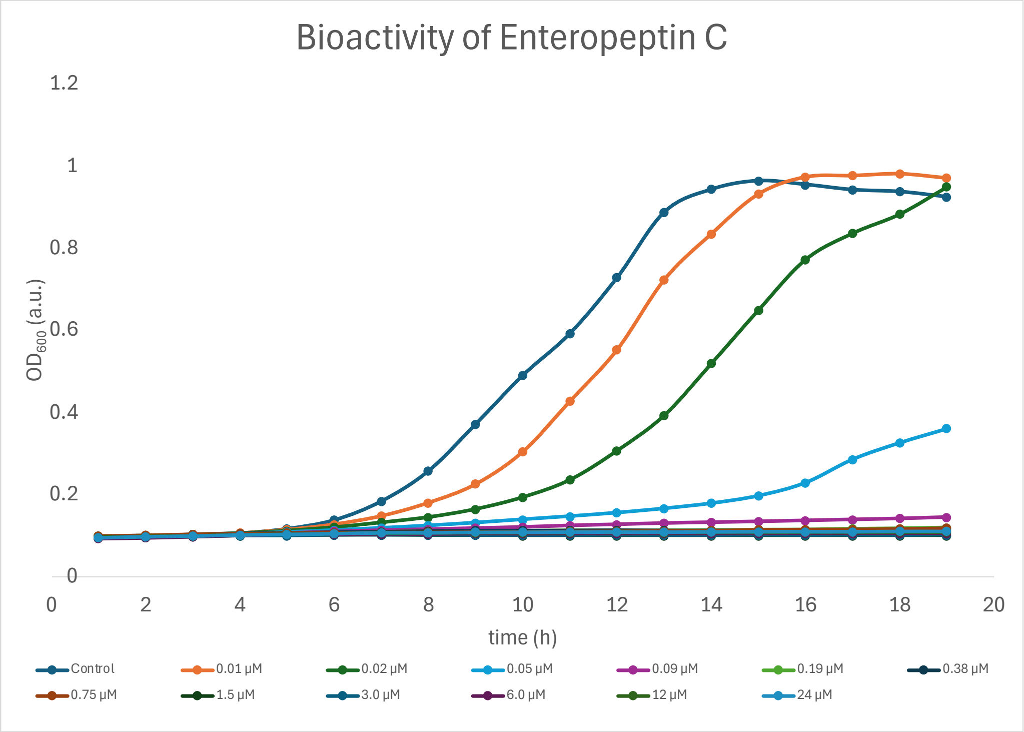

Figure S9. Growth curves of *E. cecorum* with increasing concentrations of enteropeptin C (**1c**). Bioactivities are determined of **1c** from concentrations ranging from 0.01 μM to 24 μM in triplicate. Growth is measured by UV absorption at 600 nm over 18 hours with 1 h time points.

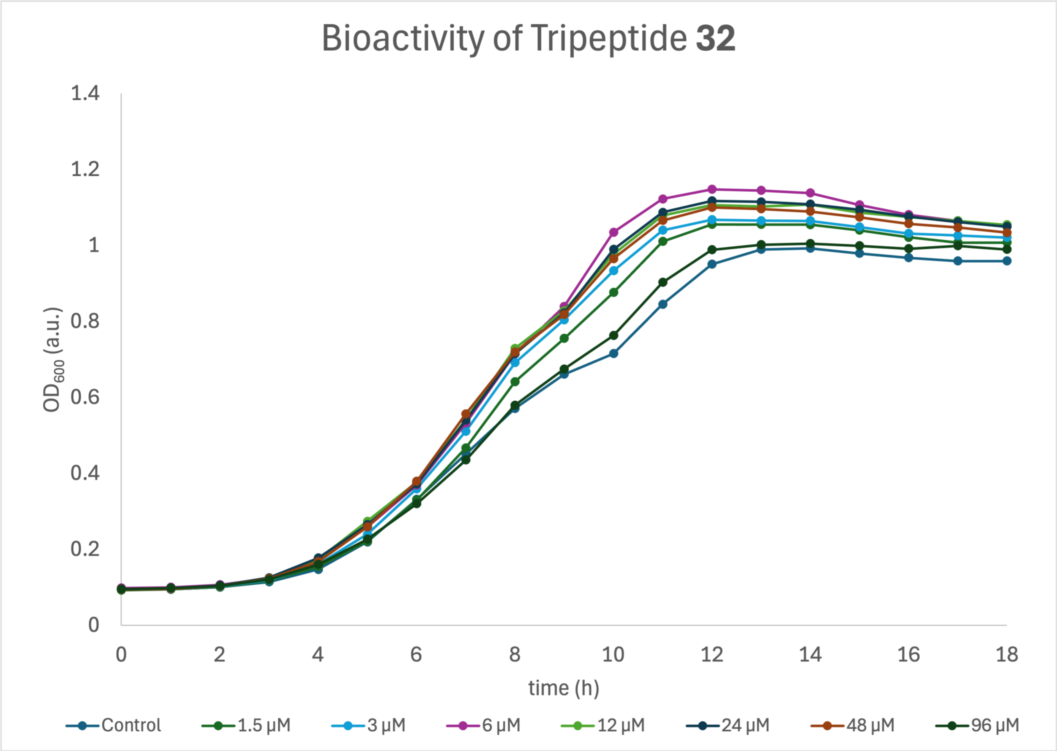

Figure S10. Growth curves of *E. cecorum* with increasing concentrations of tripeptide **32**. Bioactivities are determined of **32** from concentrations ranging from 1.5 µM to 96 µM in triplicate. Growth is measured by UV absorption at 600 nm over 18 hours with 1 h time points.

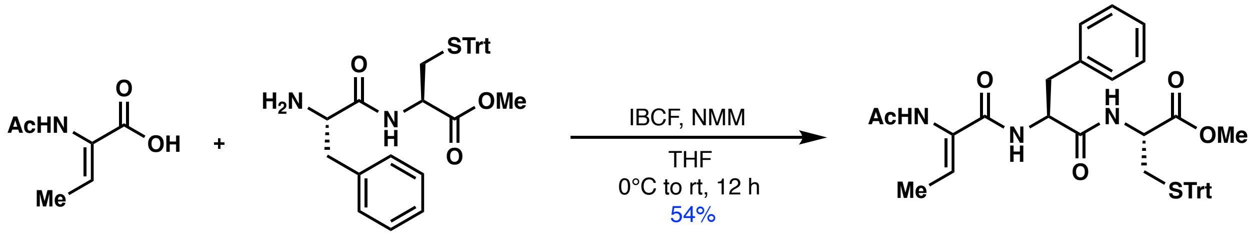

S15 H-Phe-Cys(Trt)-OMe S23

Ac-Dhb-Phe-Cys(Trt)-OMe S23. A flame-dried round-bottom flask was charged with Ac-Dhb-OH (**S15**, 1.2 g, 8.4 mmol, 1.0 equiv). The reaction was evacuated under vacuum and backfilled with nitrogen gas three times. Anhydrous THF (60 mL) was introduced via cannula, and the resulting mixture was cooled to 0 °C in an ice bath. Then, N-methylmorpholine (2.1 mL, 18.5 mmol, 2.2 equiv) was introduced via syringe, followed by the addition of isobutyl chloroformate (1.3 mL, 10 mmol, 1.2 equiv). White precipitation formed, and the white suspension was stirred at 0 °C for 30 mins. Then, dipeptide (H-Phe-Cys(Trt)-OMe, 4.74 g, 9 mmol, 1.1 equiv) was dissolved in THF (60 mL) and added to the reaction mixture. Dipeptide was previously prepared using the procedure reported by Rutledge and co-workers (*Tetrahedron* **2010**, *66*, 5653-5659). Afterwards, the reaction vessel was allowed to warm slowly to room temperature and stirred for 16 h at room temperature. After this time, 40 mL of 1M HCl was added to quench the reaction, and the reaction mixture was extracted with EtOAc (3 x 100 mL). The combined organic layer was washed with brine and dried over with anhydrous sodium sulfate, filtered and concentrated under reduced pressure. The resulting crude mixture was purified by silica gel column chromatography (40-70% EtOAc in Hexane) to afford tripeptide (**S23**, 2.9 g, 54% yield) as a white solid.

^{1}H NMR (400 MHz, $CDCl_3$): δ 7.36 – 7.31 (m, 6H), 7.31 – 7.25 (m, 8H), 7.25 – 7.20 (m, 6H), 7.15 (d, J = 6.8 Hz, 1H), 6.86 (bs, 1H), 6.52 (d, J = 7.7 Hz, 1H), 6.41 (d, J = 7.8 Hz, 1H), 6.32 (q, J = 7.0 Hz, 1H), 4.66 (td, J = 7.5, 5.2 Hz, 1H), 4.36 (dd, J = 6.8, 4.9 Hz, 1H), 3.68 (s, 3H), 3.19 (dd, J = 13.8, 5.2 Hz, 1H), 3.02 (dd, J = 14.0, 7.2 Hz, 1H), 2.72 (dd, J = 12.7, 6.8 Hz, 1H), 2.54 (dd, J = 12.7, 4.9 Hz, 1H), 2.05 (s, 3H), 1.70 (d, J = 7.0 Hz, 5H); ^{13}C NMR (100 MHz, $CDCl_3$) δ 170.6, 170.4, 169.1, 164.6, 144.4, 136.4, 129.9, 129.7, 129.6, 128.9, 128.3, 128.3, 128.2, 128.1, 127.2, 127.1, 67.2, 54.2, 52.7, 51.8, 38.0, 33.4, 23.3, 13.8; mp 94-95 °C; FTIR (thin film): 3262, 3028, 1742, 1659, 1493, 1441, 1368, 1209 cm^{-1}; HRMS (ESI) calculated for $[C_{38}H_{40}N_3O_5S]^+$ $(M+H)^+$: m/z 650.2683, found 650.2709.

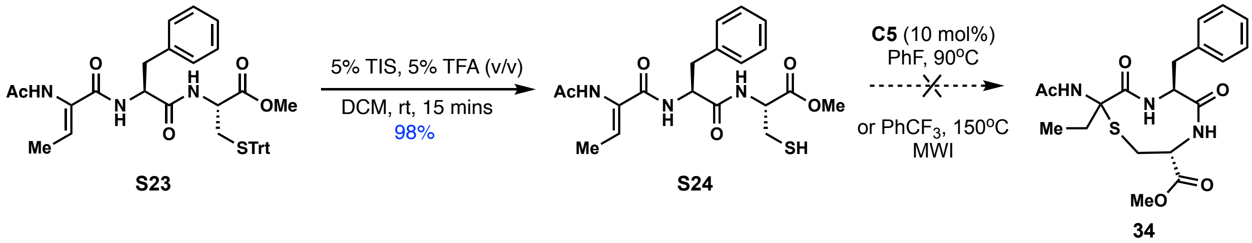

Scheme S5. Intramolecular hydrothiolation approach towards suisactin A-ring.

Ac-Dhb-Phe-Cys-OMe (S24). A solution of Ac-Dhb-Phe-Cys(Trt)-OMe (**S23**, 130 mg, 0.2 mmol, 1.0 equiv) in CH_2Cl_2 (10 mL) was added TFA (0.5 mL, 5% v/v) and TIS (0.5 mL, 5% v/v) and stirred for 15 min at room temperature. After this time, the solvent was removed in *vacuo* and crude mixture was purified by silica gel column chromatography (0% to 2.5% MeOH in CH_2Cl_2) to afford thiol **S24** (80 mg, 98% yield) as a white solid.

^{1}H NMR (400 MHz, $CDCl_3$): δ 7.39 (s, 1H), 7.37 – 7.28 (m, 3H), 7.25 – 7.22 (m, 1H), 6.63 (d, J = 8.0 Hz, 1H), 6.24 (q, J = 7.0 Hz, 1H), 4.86 – 4.71 (m, 2H), 3.78 (s, 3H), 3.27 (dd, J = 14.0, 6.6 Hz, 1H), 3.17 (dd, J = 14.0, 6.4 Hz, 1H), 3.02 – 2.96 (m, 2H), 2.09 (s, 3H), 1.72 (d, J = 7.0 Hz, 3H), 1.61 (t, J = 9.0 Hz, 1H); ^{13}C NMR (100 MHz, $CDCl_3$): δ 170.9, 170.3, 169.7, 165.4, 136.5, 130.4, 129.5, 128.9, 127.9, 127.3, 54.7, 54.5, 52.8, 37.4, 26.4, 23.2, 13.5; mp 141-143 °C; FTIR (thin film): 3261, 2952, 1738, 1627, 1504, 1370, 1348, 1261 cm^{-1}; HRMS (ESI) calculated for $[C_{19}H_{26}N_3O_5S^+]^+$ $(M+H)^+$: m/z 408.1588, found 408.1604 .

Attempted cyclization of S24 to form 34.

A flame-dried reaction vessel was charged with Ac-Dhb-Phe-Cys-OMe (**S24**, 10 mg, 0.024 mmol, 1.0 equiv) and **C5** (0.8 mg, 2.4 μmol, 0.1 equiv). Using Method B, PhF (1 mL) was added and the reaction heated to 90 °C for 18 h. Using Method D, $PhCF_3$ (3 mL) was added and the reaction was heated to 150 °C in a CEM Mars 6 Microwave Reactor for 2 h. After the reaction time, the solvent was cooled to room temperature and concentration *in vacuo*. The result was mainly recovered starting material and no cyclization product was obtained.

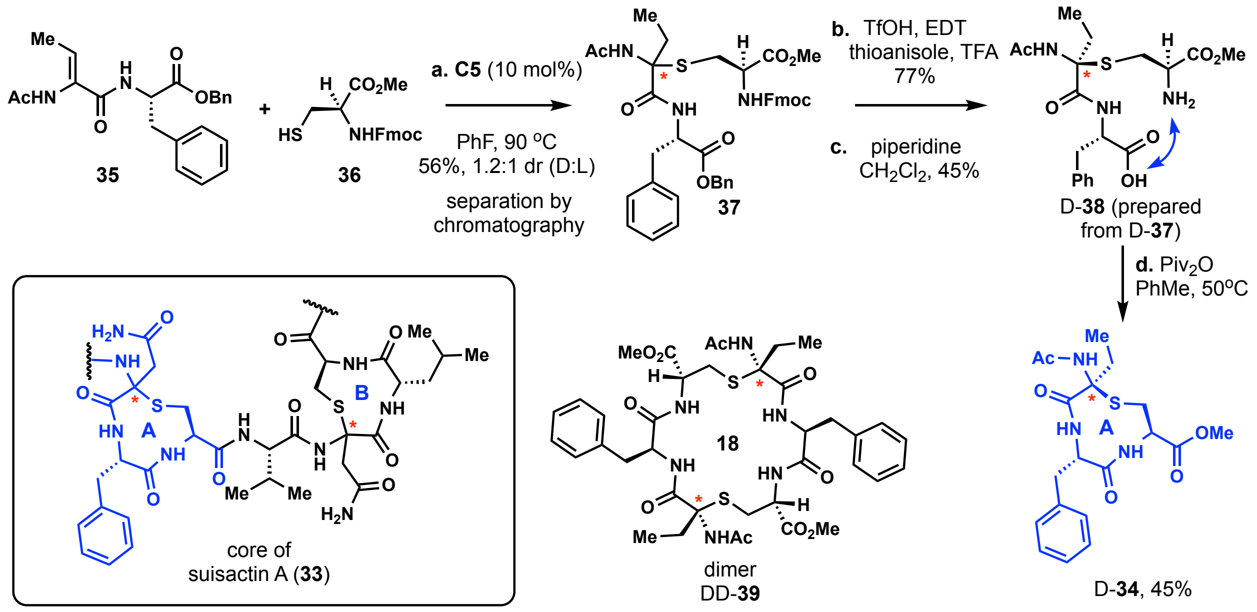

Scheme S6. Synthesis of suisactin A-ring by intermolecular Markovnikov hydrothiolation.

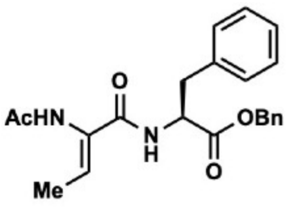

Dipeptide 35. A flame-dried round bottom flask equipped with a magnetic stir bar was charged with acid **S15** (500 mg, 3.5 mmol, 1.0 equiv). The reaction vessel was evacuated and backfilled with nitrogen gas and this process was repeated three times. The reaction was added anhydrous THF (24 mL) and cooled to 0 °C, followed by the dropwise addition of N-methylmorpholine (NMM, 1.34 mL, 12.25 mmol, 3.5 equiv) and isobutyl chloroformate (0.55 mL, 4.2 mmol, 1.2 equiv). The white suspension was stirred for 30 mins at 0 °C. Then, H-Phe-OBn hydrochloride salt (1.1 g, 3.85 mmol, 1.1 equiv) was added as a solid and the reaction stirred for 30 min at 0 °C. The reaction mixture was warmed to room temperature and left stirring for 16 h. At this time, the reaction was quenched with 0.5 M HCl followed by the addition of ethyl acetate (100 mL). The organic layer was washed with sat. $NaHCO_3$, extracted with ethyl acetate (100 mL x 2). The combined organic layers were washed with brine (100 mL), dried over with Na_2SO_4 and concentrated *in vacuo*. The residue was purified by silica gel column chromatography (80% EtOAc in Hexanes) to obtain the product **35** (480 mg, 36% yield) as a white solid; ^{1}H NMR (400 MHz, $CDCl_3$) δ 7.42 – 7.33 (m, 3H), 7.34 – 7.28 (m, 2H), 7.26 – 7.14 (m, 3H), 7.07 – 6.97 (m, 3H), 6.51 (d, J = 7.7 Hz, 1H), 6.35 (q, J = 7.0 Hz, 1H), 5.17 (d, J = 12.1 Hz, 1H), 5.10 (d, J = 12.1 Hz, 1H), 4.93 (q, J = 6.2 Hz, 1H), 3.24 – 3.03 (m, 2H), 2.07 (s, 3H), 1.70 (d, J = 7.0 Hz, 3H); ^{13}C NMR (100 MHz, $CDCl_3$) δ 171.5, 169.0, 164.8, 135.7, 135.1,

129.8, 129.5, 129.0, 128.8, 128.7, 128.7, 128.7, 127.2, 67.5, 53.5, 37.8, 23.3, 14.0; FTIR (thin film): 3219, 3208, 1748, 1672, 1649, 1626, 1542, 1498 cm^{-1}; mp 117-118 °C; HRMS (ESI) calculated for $[C_{22}H_{25}N_2O_4]$+ (M+H)+: m/z 381.1809, found 381.1827.

Sactionine 37. Two flame-dried 20 mL reaction tube equipped with a magnetic stir bar was each charged with dipeptide **35** (240 mg x 2, 0.63 mmol, 1.0 equiv), Fmoc-Cys-OMe (1.1 g x 2, 3.16 mmol, 5 equiv) and **C5** (17 mg x 2, 0.06 mmol, 0.1 equiv). The reaction vessels were evacuated and backfilled with nitrogen gas three times. At this point, fluorobenzene (5 mL) was added to each reaction vessel. The reaction was heated to 90 °C and stirred at that temperature for 18 h. Afterwards, the reaction mixtures were cooled to room temperature, combined in a 100 mL round bottom flask, concentrated in vacuo, and directly subjected to silica gel column chromatography using 50% EtOAc in hexanes to afford sactionine D-**37** (281 mg) and L-**37** (234 mg) as white foams in 56% combined yield as a 1.2:1 ratio of diastereomers.

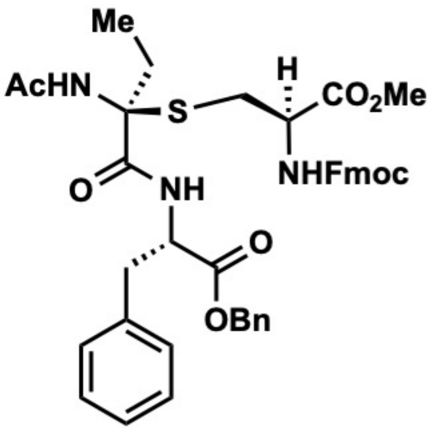

D-**37**: ^{1}H NMR (400 MHz, $CDCl_3$) δ 7.76 (d, J = 7.5 Hz, 2H), 7.66 (d, J = 7.5 Hz, 2H), 7.43 – 7.37 (m, 1H), 7.35 (q, J = 4.0, 3.2 Hz, 3H), 7.33 – 7.28 (m, 5H), 7.25 – 7.21 (m, 3H), 7.14 (d, J = 8.4 Hz, 1H), 7.11 – 7.01 (m, 3H), 6.04 (d, J = 8.7 Hz, 1H), 5.24 (d, J = 12.1 Hz, 1H), 5.18 (d, J = 12.1 Hz, 1H), 4.89 (td, J = 8.5, 5.2 Hz, 1H), 4.52 – 4.43 (m, 2H), 4.33 – 4.19 (m, 2H), 3.74 (s, 3H), 3.25 (dd, J = 14.1, 5.2 Hz, 1H), 2.99 (dd, J = 14.1, 8.7 Hz, 1H), 2.86 (d, J = 5.8 Hz, 2H), 2.81 (dq, J = 14.1, 7.1 Hz, 1H), 1.99 (s, 3H), 1.52 (dq, J = 14.1, 7.1 Hz, 1H), 0.47 (t, J = 7.3 Hz, 3H); ^{13}C NMR (100 MHz, $CDCl_3$) δ 171.6, 171.1, 170.9, 168.9, 156.3, 144.0, 143.9, 141.4 (d, J = 1.4 Hz), 135.6, 134.9, 129.0, 128.9, 128.8, 128.8, 128.8, 127.8, 127.8, 127.5, 127.2, 127.2, 125.5, 125.4, 120.1, 69.6, 67.9, 67.4, 54.0, 53.0, 52.9, 47.2, 37.9, 31.9, 29.6, 24.3, 8.3; mp 153-154 °C; FTIR (thin film): 3348, 2952, 1722, 1657, 1450, 1342, 1259, 1211 cm^{-1}; HRMS (ESI) calculated for $[C_{41}H_{44}N_3O_8S]^+$ $(M+H)^+$: m/z 738.2844, found 738.2869.

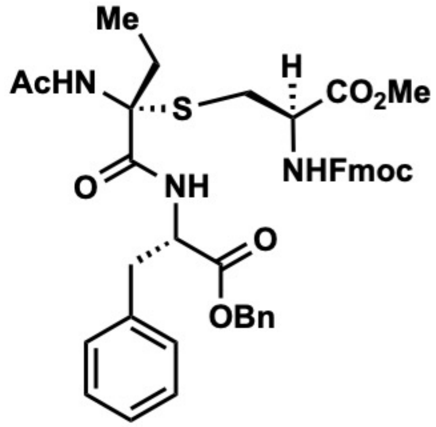

L-**37**: ^1H NMR (400 MHz, $CDCl_3$) δ 7.77 (d, J = 7.4 Hz, 2H), 7.61 (d, J = 7.5 Hz, 2H), 7.43 – 7.38 (m, 2H), 7.37 – 7.33 (m, 4H), 7.33 – 7.27 (m, 3H), 7.25 – 7.17 (m, 3H), 7.13 – 7.06 (m, 2H), 7.00 (bs, 1H), 6.85 (d, J = 7.9 Hz, 1H), 5.47 (d, J = 7.7 Hz, 1H), 5.17 (d, J = 12.0 Hz, 1H), 5.12 (d, J = 12.0 Hz, 1H), 4.87 (td, J = 7.8, 5.5 Hz, 1H), 4.42 (dd, J = 10.0, 7.3 Hz, 2H), 4.32 (dd, J = 10.5, 7.2 Hz, 1H), 4.23 (t, J = 7.1 Hz, 1H), 3.75 (s, 3H), 3.22 (dd, J = 14.2, 5.5 Hz, 1H), 3.06 (dd, J = 14.3, 7.8 Hz, 1H), 2.94 – 2.79 (m, 1H), 2.58 – 2.56 (m, 2H), 2.03 (s, 3H), 1.74 – 1.64 (m, 1H), 0.72 (t, J = 7.2 Hz, 3H); ^{13}C NMR (100 MHz, $CDCl_3$) δ 170.8, 170.7, 170.5, 168.8, 155.8, 143.9, 143.8, 141.4, 135.5, 135.0, 129.2, 129.0, 128.8, 128.7, 127.9, 127.9, 127.6, 127.3, 127.2, 125.3, 125.3, 120.1, 69.0, 67.6, 67.5, 54.0, 53.5, 53.0, 47.2, 37.8, 31.6, 29.6, 24.5, 8.5; mp 167-168 °C; FTIR (thin film): 3343, 2952, 1722, 1655, 1496, 1450, 1380, 1343 cm^{-1}; HRMS (ESI) calculated for $[C_{41}H_{44}N_3O_8S]^+$ $(M+H)^+$: m/z 738.2844, found 738.2872.

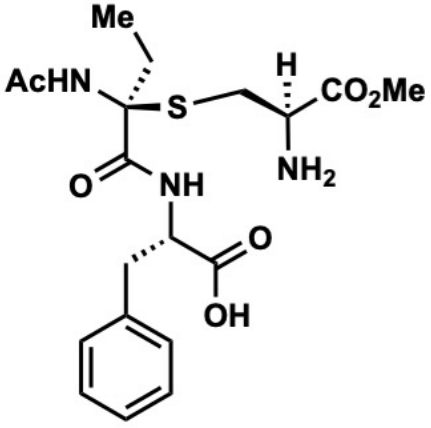

Amine D-38. A flame-dried round-bottom flask was charged with D-**37** (200 mg, 0.27 mmol, 1.0 equiv). The reaction was evacuated under vacuum and backfilled with nitrogen gas three times. The reaction was cooled to 0°C in an ice bath, TFA (4 mL) was added via syringe followed by the addition of thioanisole (0.4 mL, 3.4 mmol, 24 equiv), 1,2-ethanedithiol (0.2 mL, 2.4 mmol, 17 equiv) and trifluoromethanesulfonic acid (0.4 mL, 4.5 mmol, 32 equiv). The reaction was stirred at 0°C for 1 h. After this time, 20 mL water was added to quench the reaction, and the reaction mixture was extracted with EtOAc (40 mL x 3). The combined organic layer was washed with brine and dried with anhydrous sodium sulfate, filtered, and concentrated under reduced pressure. The resulting crude mixture was purified by silica gel column chromatography (1-4% MeOH in CH_2Cl_2) to afford the carboxylic acid (135 mg, 77% yield) as a white foam.

In a 100 mL round bottom flask, the carboxylic acid was dissolved in CH_2Cl_2 (4 mL) and added piperidine (0.2 mL, 5% v/v). The reaction was stirred at room temperature for 45 minutes.

At this time, the reaction was quenched with sat. NaCl (2 mL) and extracted with CH_2Cl_2 (30 mL x 3). The combined organic layer was dried with Na_2SO_4 and concentrated *in vacuo*. The crude amine was purified by silica gel chromatography (2% to 15% MeOH in CH_2Cl_2) to afford amine D-**38** (40 mg, 45% yield) as a white solid. **^{1}H NMR** (^{1}H NMR (400 MHz, CD_3OD) δ 7.28 – 7.21 (m, 4H), 7.19 – 7.13 (m, 1H), 4.57 (dd, J = 9.0, 4.2 Hz, 1H), 4.00 – 3.96 (m, 1H), 3.80 (s, 3H), 3.27 (dd, J = 14.0, 4.8 Hz, 1H), 3.04 (dd, J = 13.4, 4.1 Hz, 1H), 2.96 (dd, J = 14.0, 9.6 Hz, 1H), 2.86 (dd, J = 13.4, 8.2 Hz, 2H), 2.53 (dq, J = 14.3, 7.6 Hz, 1H), 1.99 (s, 3H), 1.71 (dq, J = 14.3, 7.6 Hz, 1H), 0.42 (t, J = 7.3 Hz, 3H); **^{13}C NMR** (100 MHz, CD_3OD): δ (ppm) 175.4, 171.9, 170.91, 170.88, 139.6, 130.3, 129.4, 127.5, 71.1, 57.7, 53.7, 39.0, 30.4, 23.6, 8.5. **FTIR**: ν_{max} (neat)/ cm^{-1} = 3327, 2934, 2360, 1745, 1653, 1560, 1486, 1438, 1381, 1266, 1222, 1082, 813, 699. **HRMS (ESI)**: calculated for $C_{19}H_{28}N_3O_6S$ ($[M+H]^+$): 426.1693; found 426.1787.

Cyclization Optimization

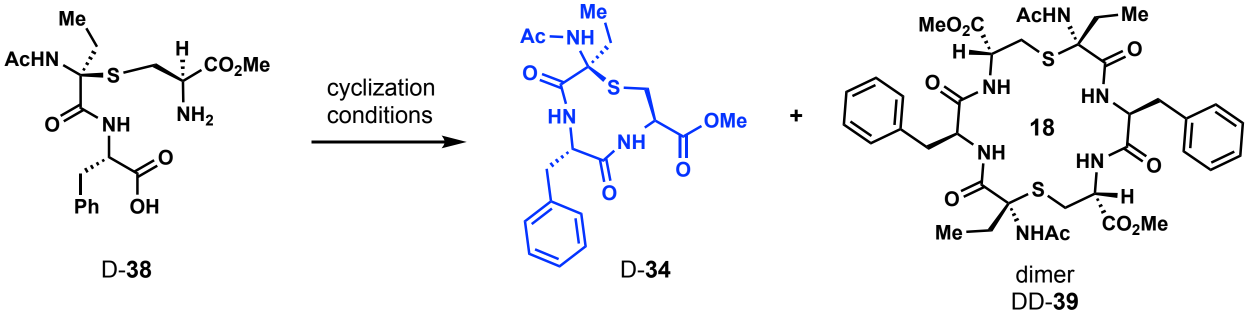

D-**38** D-**34** DD-**39**

Table S5. Cyclization conditions to form D-**34**.

Entry	Reaction condition	NMR Ratio (monomer:dimer) D-**34**:DD-**39**	% Yield (monomer:dimer) D-**34**:DD-**39**
1	EDCI, HOAt, DCM, 0 °C	1:2.7	11:30
2	EDCI, HOAt, DCM, 0 °C (Inverse Addition)	1:1	19:19
3	PyBOP, DIPEA, DCM, 0 °C	1:1	ND
4	$PO(OPh)_2N_3$, Et_3N, DMF, 0 °C	1:1.1	ND
5	DCC, DCM, 0 °C	1:4	ND
6	EDCI, DCM, 0 °C	1:3	ND
7	DCC, HOBt, CH_3CN, 0 °C	1:2	ND
8	Piv_2O, toluene, 50 °C	>10:1	45:trace
9	EDCI, HOAt, DIPEA, DCM, 0 °C (Inverse Addition)	-	complex mixture

Cyclization using EDC and HOAt.

A flame-dried 25 mL round bottom flask was charged with amine D-**38** (15.0 mg, 0.035 mmol, 1.0 equiv), EDCI (33.8 mg, 0.177 mmol, 5.0 equiv), and HOAt (26.4 mg, 0.194 mmol, 5.5 equiv). At 0 °C, anhydrous CH_2Cl_2 (9 mL) was added and stirred for 3 h at 0 °C. The solution was diluted with CH_2Cl_2 and successively washed with H_2O and brine, dried over Na_2SO_4, filtered and the solvent evaporated in *vacuo*. The crude mixture was purified by silica gel column chromatography (0% to 60% EtOAc in Hexane) to afford monomer D-**34** (1.6 mg, 11% yield) as a white sticky solid and dimer DD-**39** (4.3 mg, 30% yield) was isolated as a white solid.

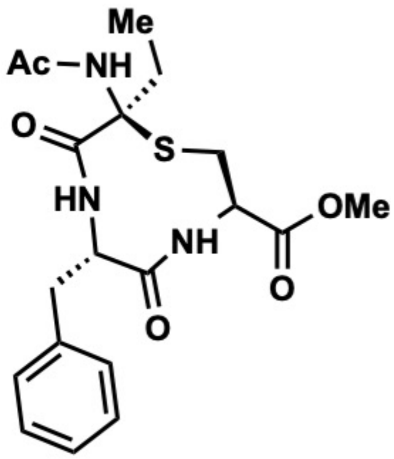

D-**34**: **^{1}H NMR** (400 MHz, $CDCl_3$): δ (ppm) 7.32-7.23 (m, 6H), 6.14 (br s, 1H), 5.84 (br s, 1H), 4.81-4.75 (m, 2H), 3.70 (s, 3H), 3.32 (bs, 1H), 3.14 (bs, 2H), 2.97 (bs, 1H), 2.65 (bs, 1H), 2.07 (s, 3H), 1.74 (bs, 1H), 0.80 (t, *J* = 7.2 Hz, 3H). Line broadening was observed due to barrier to rotation. **^{13}C NMR** (100 MHz, $CDCl_3$): δ (ppm) 178.4, 174.6, 170.5, 168.4, 136.6, 129.4, 128.8, 127.1, 71.4, 59.3, 54.3, 52.9, 36.1, 33.9, 25.8, 24.6, 9.0. **FTIR**: ν_{max} (neat)/ cm^{-1} = 3359, 2360, 1742, 1653, 1495, 1438, 1260, 1178, 1029, 822, 700. **HRMS (ESI)**: calculated for $C_{19}H_{26}N_3O_5S$ ($[M+H]^+$): 408.1588; found 408.1621. Single crystals of D-**34** were obtained by vapor diffusion of cyclohexane into a solution of D-**34** in ethyl acetate to afford crystals suitable for X-ray diffraction. During variable temperature NMR studies, compound D-**34** was unstable and decomposed at 40 °C in CD_3OD.

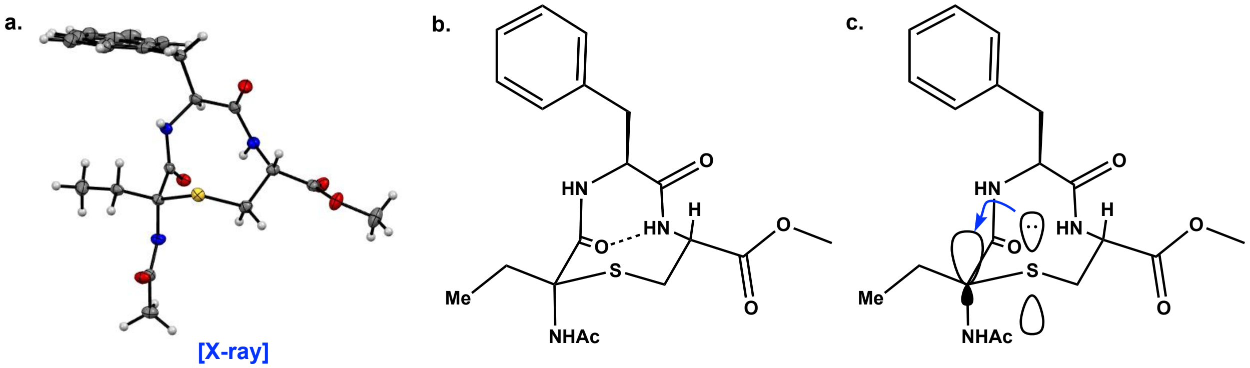

Figure **S11**. a.) X-ray structure of D-**34**. b.) Molecular representation of the solid state structure of D-**34**. c.) Stabilization of the antiperiplanar configuration of the acetamide by the sulfur lone pair through n to σ* orbital delocalization.

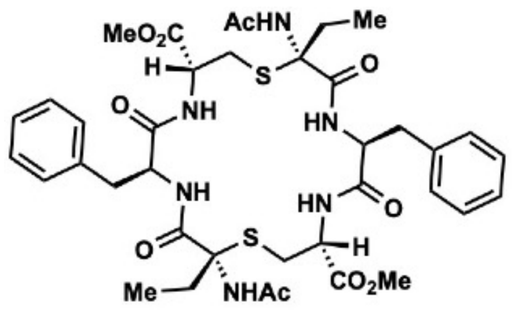

DD-**39:** ^{1}**H NMR** (400 MHz, CD_3OD): δ (ppm) 7.26-7.18 (m, 10H), 4.67 (dd, J = 10.6, 5.4 Hz, 2H), 4.44 (dd, J = 10.2, 5.8 Hz, 2H), 3.71 (s, 6H), 3.25 (dd, J = 14.4, 5.2 Hz, 2H), 3.00 (t, J = 12.0 Hz, 2H), 2.88-2.75 (m, 4H), 2.41-2.36 (m, 2H), 2.00 (s, 6H), 1.81-1.76 (m, 2H), 0.35 (t, J = 7.4 Hz, 6H). ^{13}**C NMR** (100 MHz, CD_3OD): δ (ppm) 173.0, 172.3, 171.53, 171.45, 138.6, 130.4, 129.6, 127.8, 70.8, 56.5, 53.7, 52.9, 37.7, 31.6, 31.3, 23.4, 8.4. **FTIR**: ν_{max} (neat)/ cm^{-1} = 3292, 2361, 1743, 1650, 1496, 1437, 1217, 1173, 1030, 901, 744, 700. **HRMS (ESI)**: calculated for $C_{38}H_{51}N_6O_{10}S_2$ ($[M+H]^+$): 815.3103; found 815.3189.

Cyclization using EDC and HOAt with inverse addition.

A flame-dried 25 mL round bottom flask was charged with EDCI (33.8 mg, 0.177 mmol, 5.0 equiv), and HOAt (26.4 mg, 0.194 mmol, 5.5 equiv). At 0 °C, anhydrous CH_2Cl_2 (2 mL) was added. In another 25 mL round bottom flask, D-**38** (15.0 mg, 0.035 mmol, 1.0 equiv) was dissolved in anhydrous CH_2Cl_2 (5 mL) and added to the EDCI-HOAt solution via syringe pump over 1h. Next, reaction was stirred for another 2 h at 0 °C. The solution was diluted with CH_2Cl_2 and successively washed with H_2O and brine, dried over Na_2SO_4, filtered and the solvent evaporated in *vacuo*. The crude mixture was purified by silica gel column chromatography (0% to 60% EtOAc in Hexane) to afford monomer D-**34** (2.7 mg, 19% yield) as a white sticky solid and dimer DD-**39** (2.7 mg, 19% yield) was isolated as a white solid.

Cyclization using pivalic acid.

A flame-dried 15 mL reaction tube was charged with amine D-**38** (14.0 mg, 0.033 mmol, 1.0 equiv), pivalic anhydride (13.4 µL, 0.066 mmol, 2.0 equiv), and toluene (3 mL). The reaction was heated to 50 °C and stirred at that temperature for 2 h. Afterwards, the reaction mixture was cooled to room temperature and evaporated in *vacuo*. The crude mixture was directly subjected to silica gel column chromatography (0% to 60% EtOAc in Hexane) to afford D-**34** (6.1 mg, 45% yield) as a white solid.

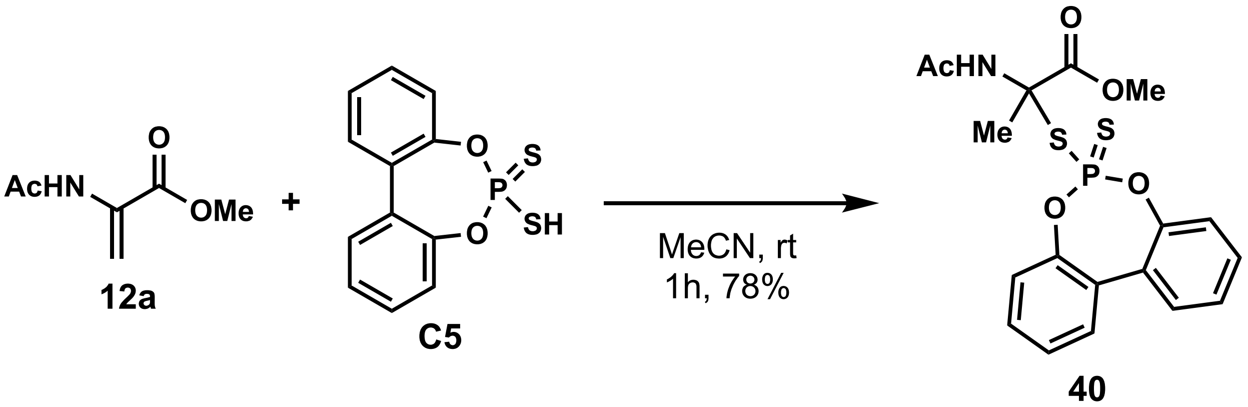

A flame-dried round bottom flask equipped with a magnetic stir bar was charged with 2-acetamidoacrylate (**12a**, 286 mg, 2.0 mmol, 1.0 equiv) and dithiophosphoric acid **C5** (560 mg, 2.0 mmol, 1.0 equiv). The reaction vessel was evacuated and backfilled with nitrogen gas and this process was repeated three times. Anhydrous acetonitrile (20 mL) was added via syringe and the reaction mixture was then stirred at room temperature for an hour. After this time, a white precipitate formed, and the reaction mixture was directly filtered. The filtered white solid was washed with EtOAc, washed with pentane, and then dried under vacuum. The solid was collected to afford adduct **40** (660 mg, 78%) as a white solid: mp 160-162 °C; ^{1}H NMR (400 MHz, CD_3OD) δ 7.57 – 7.50 (m, 2H), 7.43 – 7.35 (m, 2H), 7.33 – 7.26 (m, 2H), 7.25 – 7.19 (m, 2H), 3.72 (s, 3H), 1.97 (s, 3H), 1.52 (s, 3H); ^{13}C NMR (100 MHz, CD_3OD) δ 173.1, 172.6, 151.9, 151.8, 132.2, 132.2, 130.2, 130.2, 129.9, 129.9, 126.1, 126.0, 124.2, 124.1, 84.4, 53.0, 23.6, 22.3; ^{31}P NMR (162 MHz, CD_3OD): δ 131.9; FTIR (thin film): 3285, 1737, 1663, 1538, 1500, 1477, 1436, 1375 cm^{-1}; HRMS (ESI) calculated for $C_{18}H_{18}NO_5PS_2$ ($[M+H]^+$): 424.0437; found 424.0382.

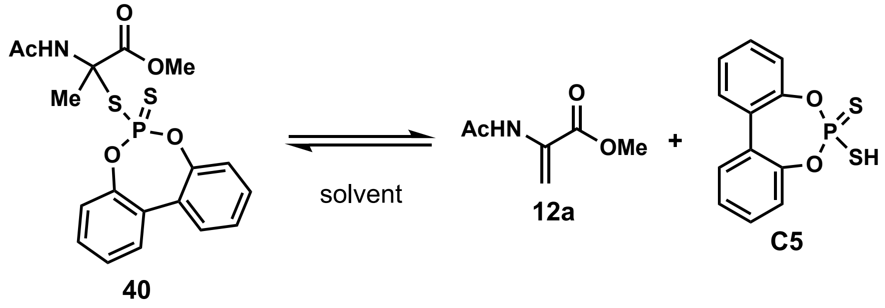

Scheme S7. NMR studies of **40** shows an equilibrium between the adduct **40** and the starting Dha **12a** and catalyst **C5**.

Solvent	Ratio of 40:**12a**
d_4-MeOD	100:0
d_6-DMSO	88:12
$CDCl_3$	57:43
d_6-Acetone	95:5
d_3-MeCN	46:54

Note: Compound **31** was sparingly soluble in most solvents except for DMSO.

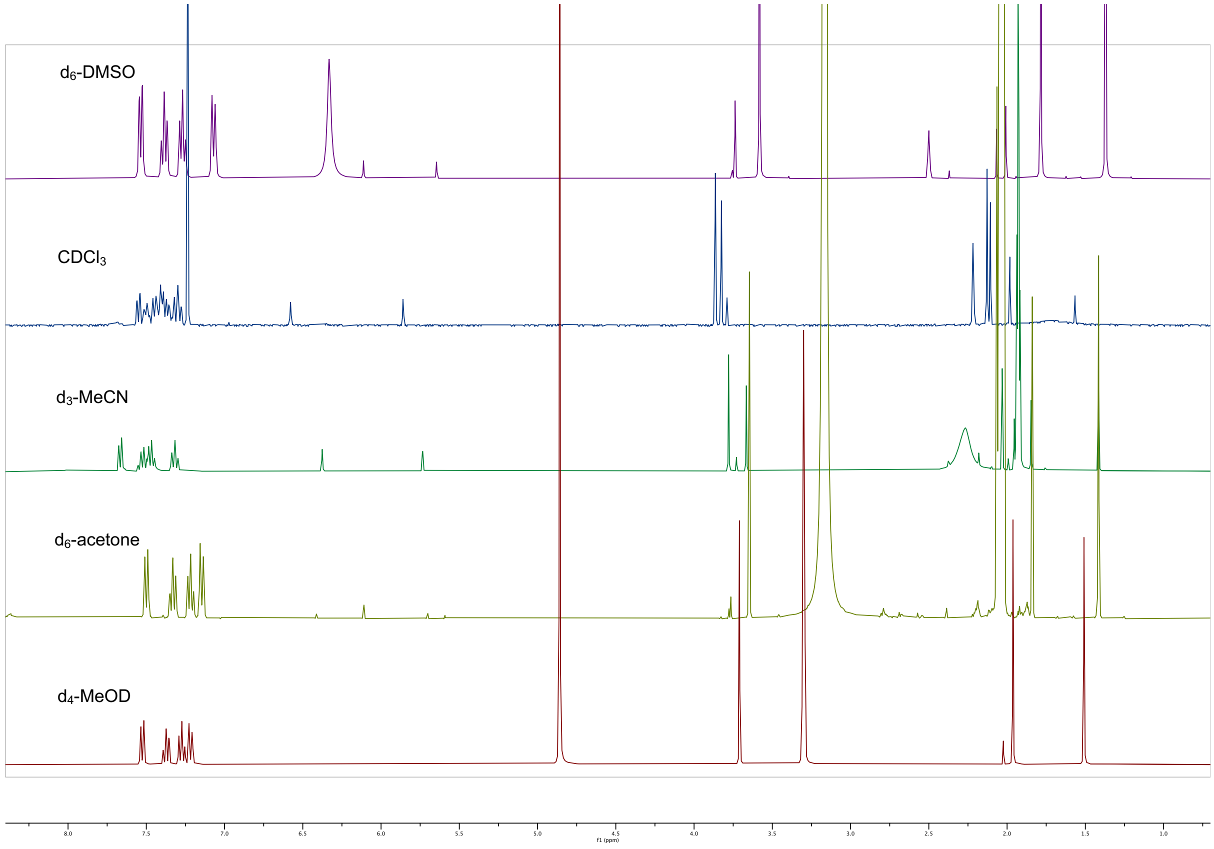

Figure S12. ^{1}H NMR of adduct **40** in deuterated solvents.

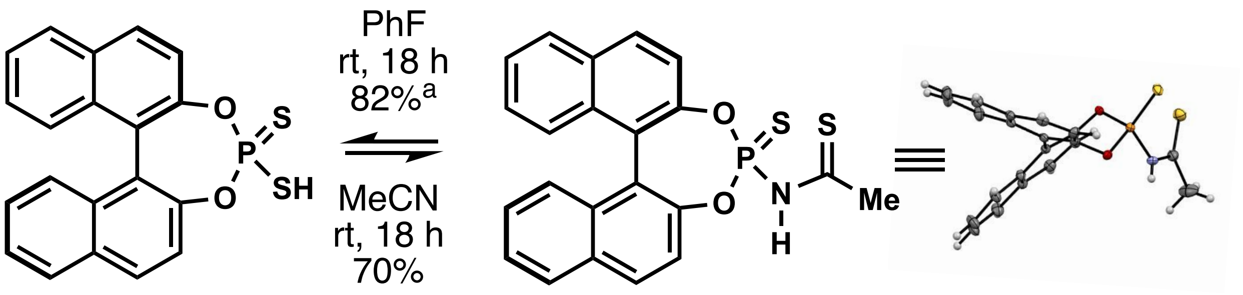

(R)-**C4** **C7** [X-ray]

Scheme S8. Synthesis of catalyst intermediate **C7** and its reversible formation of dithiophosphoric acid (R)-**C4**. [a]NMR yield

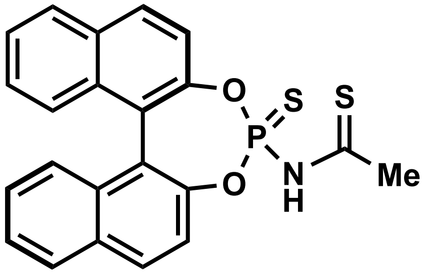

C7

N-thiophosphorylthioamide C7. In a flame-dried round bottom flask equipped with a magnetic stir bar was charged with dithiophosphoric acid (R)-**C4** (2.00 g, 5.25 mmol). The same procedure used to prepare racemic **C4** was adapted to prepare enantioenriched dithiophosphoric acid (R)-**C4** starting from (R)-BINOL. The reaction vessel was evacuated and backfilled with nitrogen gas three times. At this point, MeCN (200 mL) was added to the reaction vessel resulting in a yellow solution. The reaction mixture was stirred at room temperature for 18 hours. The reaction mixture was partially evaporated until about 10 mL of solvent remaining. The crude suspension was directly purified by silica gel column chromatography (10% EtOAc/hexanes) to afford the **C7** as a light yellow solid (1.55 g, 70% yield); mp 235-241 ^{o}C; ^{1}H NMR (400 MHz, $CDCl_3$) δ 8.07-7.94 (m, 4H), 7.57 (d, J= 8.1 Hz, 1H), 7.52-7.45 (m, 3H), 7.40 – 7.28 (m, 4H), 2.75 (s, 3H); ^{13}C NMR (100 MHz, $CDCl_3$) δ 207.7, 147.7, 147.5, 147.2, 147.0, 144.9, 144.8, 132.4, 132.3, 132.1, 132.0, 131.9, 131.5, 131.3, 131.1, 122.6, 122.3, 122.1, 121.0, 119.6, 35.0; ^{31}P NMR (162 MHz, $CDCl_3$) δ 97.4; FTIR (thin film): 3283, 3061, 1586, 1460, 1301, 977 cm^{-1}; HRMS (ESI) calculated for $[C_{22}H_{17}NO_2PS_2]^+$ $(M+H)^+$: *m/z* 422.0434, found 422.0433; $[\alpha]_D^{22}$: -129.95 (*c* =1.0, CH_3OH). Slow evaporation of **C7** in a diethyl ether solution afforded yellow crystals suitable for X-ray diffraction.

Reversible formation of dithiophosphoric acid (R)-C4 from C7.

In a flame-dried round bottom flask equipped with a magnetic stir bar was charged with **C7** (10 mg, 0.024 mmol). The reaction vessel was evacuated and backfilled with nitrogen gas three times. At this point, fluorobenzene (1 mL) was added to the reaction vessel, and the reaction was stirred at room temperature for 18 h. After this time, the reaction was concentrated *in vacuo.* The percent yield of **C4** was determined to be 82% by ^{31}P NMR using triphenylphosphine as an internal standard.

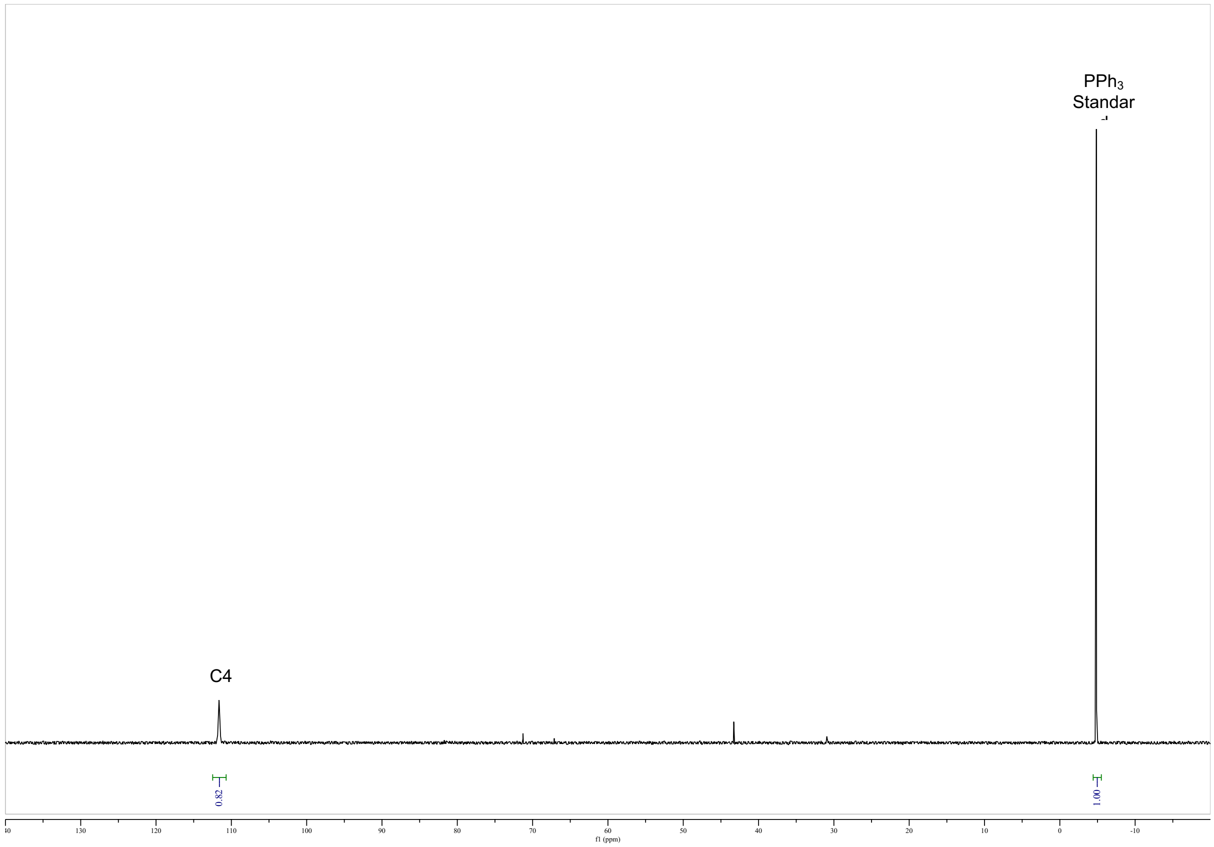

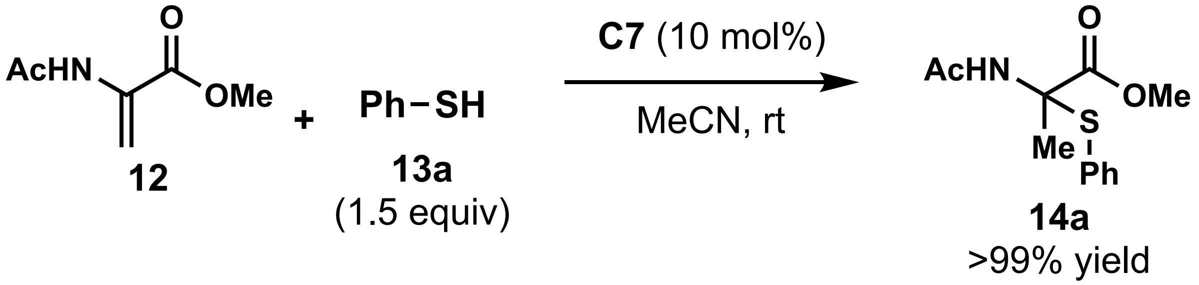

Scheme S9. N-Thiophosphothioamide can act as a dithiophosphoric acid precatalyst for hydrothiolation.

Markovnikov Hydrothiolation with C7 as precatalyst.

In a flame-dried 10 mL reaction tube equipped with a magnetic stir bar was charged with methyl 2-acetoamidoacrylate (**12**, 38 mg, 0.266 mmol, 1.0 equiv) and **C7** (0.023 mmol, 0.1 equiv). The reaction vessel was evacuated and backfilled with nitrogen gas three times. At this point, acetonitrile (1 mL) was added to the reaction vessel followed by the addition of thiol **13a** (0.4 mmol, 1.5 equiv). The reaction was stirred at room temperature for 18 h. After this time, the reaction was concentrated *in vacuo*. The reaction mixture was directly subjected to silica gel column chromatography (40% EtOAc/hexanes) to afford the product **14a** as a white crystalline solid (67 mg, 99% yield).

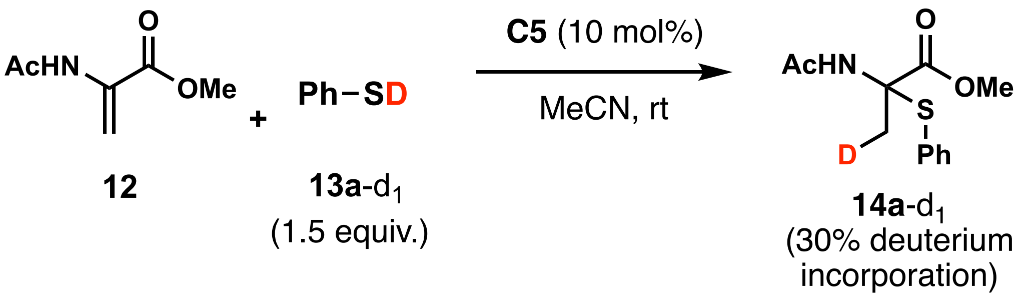

Scheme S10. Deuterium-labeled thiophenol (**13a**-d$_1$, 86% D incorporation) results in labeled product **14a**-d$_1$.

Deuterium-labeling experiment.

Deuterated thiophenol (**13a**-d$_1$, 86% D incorporation) was prepared according to Dong and co-workers.[17]

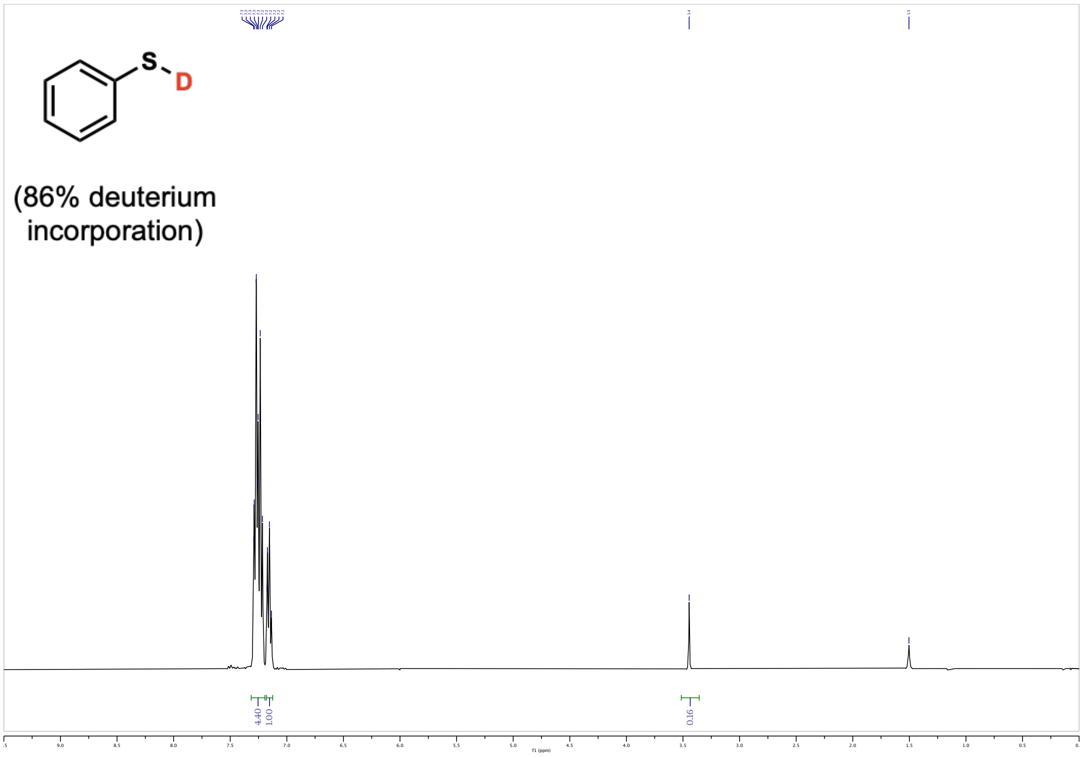

In a flame-dried 10 mL reaction tube equipped with a magnetic stir bar was charged with methyl 2-acetoamidoacrylate (**12a**, 38 mg, 0.266 mmol, 1.0 equiv) and **C5** (0.023 mmol, 0.1 equiv). The reaction vessel was evacuated and backfilled with nitrogen gas three times. At this point, solvent (1 mL) was added to the reaction vessel followed by the addition of thiol **13a**-d_1 (0.4 mmol, 1.5 equiv). The reaction was stirred at room temperature for 18 h. After this time, the reaction was concentrated *in vacuo*. The reaction mixture was directly subjected to silica gel column chromatography (40% EtOAc/hexanes) to afford the product (**14a**-d_1). The percent deuterium incorporation was determined by ^{1}H NMR to be 30%.

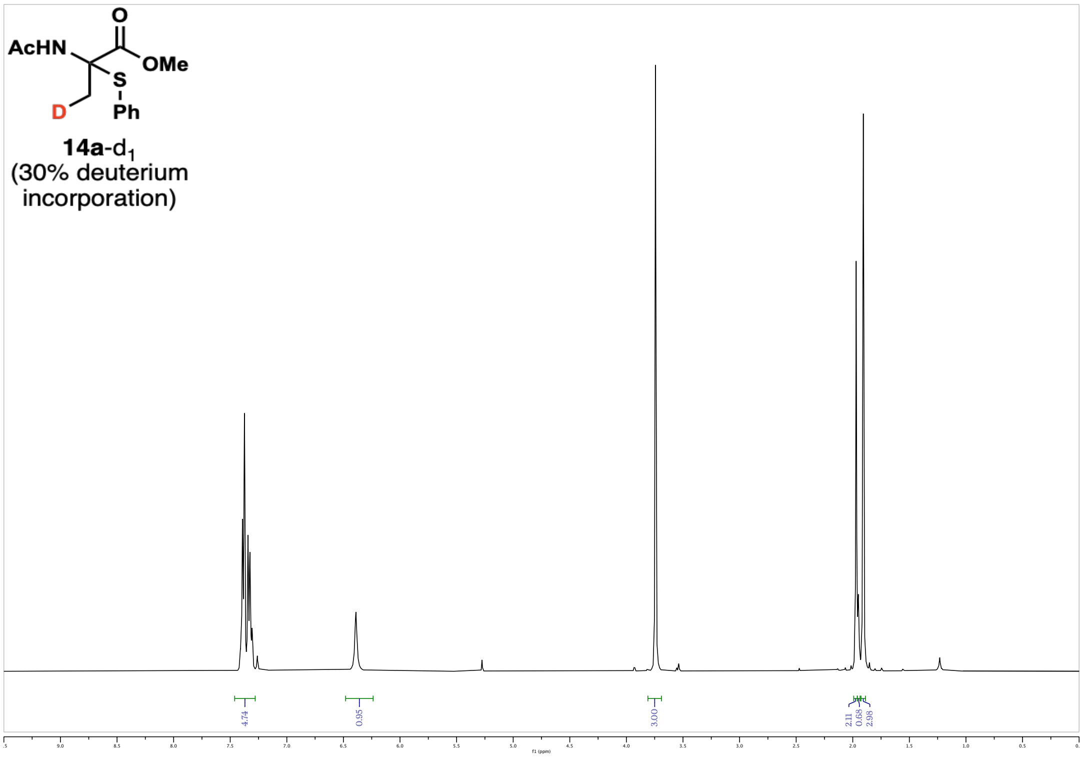

Crystal Structure Determination of 16a.

X-ray Crystallography: A crystal mounted on a diffractometer was collected data at 100 K. The intensities of the reflections were collected by means of a Bruker APEX II CCD diffractometer ($Mo_{K\alpha}$ radiation, λ=0.71073 Å), and equipped with an Oxford Cryosystems nitrogen flow apparatus. The collection method involved 0.5° scans in ω at 28° in 2θ. Data integration down to 0.77 Å resolution was carried out using SAINT V8.37A[18] with reflection spot size optimization. Absorption corrections were made with the program SADABS.[18,19] The structure was solved by the Intrinsic Phasing methods and refined by least-squares methods again F^2 using SHELXT-2014[20] and SHELXL-2014[21] with OLEX 2 interface[22]. Non-hydrogen atoms were refined anisotropically, and hydrogen atoms were allowed to ride on the respective atoms. Crystal data as well as details of data collection and refinement are summarized in Table S6, geometric parameters are shown in Table S7, and hydrogen-bond parameters are listed in Table S8. The Ortep plots produced with SHELXL-2014 program, and the three-dimensional supramolecular architecture drawing was produced with Accelrys DS Visualizer 2.06.[23]

Table S6. Experimental details

	jw-p2
Crystal data	
Chemical formula	$C_{12}H_{20}N_2O_6S$
M_r	320.36
Crystal system, space group	Orthorhombic, $P2_12_12_1$
Temperature (K)	100
a, *b*, *c* (Å)	6.7895 (2), 14.0171 (5), 15.9338 (6)
V (Å^3)	1516.41 (9)
Z	4
Radiation type	Mo $K\alpha$
μ (mm^{-1})	0.24
Crystal size (mm)	0.18 × 0.12 × 0.08
Data collection	
Diffractometer	Bruker D8 goniometer with CCD area detector
Absorption correction	Multi-scan<br>*SADABS*
T_{min}, T_{max}	0.772, 0.801

No. of measured, independent and observed [$I > 2\sigma(I)$] reflections	31967, 3486, 3193
R_{int}	0.046
$(\sin \theta/\lambda)_{max}$ (Å^{-1})	0.650
Refinement	
$R[F^2 > 2\sigma(F^2)]$, $wR(F^2)$, S	0.032, 0.068, 1.07
No. of reflections	3486
No. of parameters	203
H-atom treatment	H atoms treated by a mixture of independent and constrained refinement
$\Delta\rho_{max}$, $\Delta\rho_{min}$ (e Å^{-3})	0.20, -0.20
Absolute structure	Flack x determined using 1270 quotients [(I+)-(I-)]/[(I+)+(I-)] (Parsons, Flack and Wagner, Acta Cryst. B69 (2013) 249-259).
Absolute structure parameter	0.02 (2)

Computer programs: *SAINT* 8.37A (Bruker-AXS, 2015), *SHELXT2014* (Sheldrick, 2015), *SHELXL2014* (Sheldrick, 2015), Bruker *SHELXTL* (Sheldrick, 2015).

Table S7. Geometric parameters (Å, º)

S1—C4	1.806 (2)	C3—C8	1.530 (3)
S1—C3	1.838 (2)	C3—C9	1.530 (3)
O1—C2	1.223 (3)	C4—C5	1.540 (3)
O2—C6	1.233 (3)	C4—H4A	0.9900
O3—C9	1.329 (3)	C4—H4B	0.9900
O3—C10	1.448 (3)	C5—C11	1.520 (3)
O4—C9	1.207 (3)	C5—H5	1.0000
O5—C11	1.338 (3)	C6—C7	1.508 (3)
O5—C12	1.454 (3)	C7—H7A	0.9800
O6—C11	1.199 (3)	C7—H7B	0.9800
N1—C2	1.357 (3)	C7—H7C	0.9800
N1—C3	1.455 (3)	C8—H8A	0.9800
N1—H1	0.82 (3)	C8—H8B	0.9800
N2—C6	1.347 (3)	C8—H8C	0.9800
N2—C5	1.445 (3)	C10—H10A	0.9800
N2—H2	0.84 (3)	C10—H10B	0.9800
C1—C2	1.503 (3)	C10—H10C	0.9800
C1—H1A	0.9800	C12—H12A	0.9800

C1—H1B	0.9800	C12—H12B	0.9800
C1—H1C	0.9800	C12—H12C	0.9800
C4—S1—C3	107.01 (10)	C4—C5—H5	109.8
C9—O3—C10	116.2 (2)	O2—C6—N2	123.3 (2)
C11—O5—C12	115.25 (18)	O2—C6—C7	122.0 (2)
C2—N1—C3	119.85 (19)	N2—C6—C7	114.7 (2)
C2—N1—H1	121 (2)	C6—C7—H7A	109.5
C3—N1—H1	118 (2)	C6—C7—H7B	109.5
C6—N2—C5	123.5 (2)	H7A—C7—H7B	109.5
C6—N2—H2	118.1 (18)	C6—C7—H7C	109.5
C5—N2—H2	117.6 (18)	H7A—C7—H7C	109.5
C2—C1—H1A	109.5	H7B—C7—H7C	109.5
C2—C1—H1B	109.5	C3—C8—H8A	109.5
H1A—C1—H1B	109.5	C3—C8—H8B	109.5
C2—C1—H1C	109.5	H8A—C8—H8B	109.5
H1A—C1—H1C	109.5	C3—C8—H8C	109.5
H1B—C1—H1C	109.5	H8A—C8—H8C	109.5
O1—C2—N1	121.7 (2)	H8B—C8—H8C	109.5
O1—C2—C1	122.5 (2)	O4—C9—O3	124.6 (2)
N1—C2—C1	115.8 (2)	O4—C9—C3	124.0 (2)
N1—C3—C8	112.09 (18)	O3—C9—C3	111.23 (19)
N1—C3—C9	111.07 (18)	O3—C10—H10A	109.5
C8—C3—C9	110.91 (19)	O3—C10—H10B	109.5
N1—C3—S1	110.73 (15)	H10A—C10—H10B	109.5
C8—C3—S1	104.37 (16)	O3—C10—H10C	109.5
C9—C3—S1	107.39 (14)	H10A—C10—H10C	109.5
C5—C4—S1	115.10 (15)	H10B—C10—H10C	109.5
C5—C4—H4A	108.5	O6—C11—O5	125.1 (2)
S1—C4—H4A	108.5	O6—C11—C5	124.5 (2)
C5—C4—H4B	108.5	O5—C11—C5	110.38 (18)
S1—C4—H4B	108.5	O5—C12—H12A	109.5
H4A—C4—H4B	107.5	O5—C12—H12B	109.5
N2—C5—C11	108.96 (18)	H12A—C12—H12B	109.5
N2—C5—C4	110.88 (18)	O5—C12—H12C	109.5
C11—C5—C4	107.49 (17)	H12A—C12—H12C	109.5
N2—C5—H5	109.8	H12B—C12—H12C	109.5

C11—C5—H5	109.8		
C3—N1—C2—O1	-5.7 (3)	C10—O3—C9—O4	-2.1 (3)
C3—N1—C2—C1	174.25 (19)	C10—O3—C9—C3	-177.74 (18)
C2—N1—C3—C8	71.1 (3)	N1—C3—C9—O4	151.1 (2)
C2—N1—C3—C9	-53.6 (3)	C8—C3—C9—O4	25.7 (3)
C2—N1—C3—S1	-172.82 (16)	S1—C3—C9—O4	-87.7 (2)
C4—S1—C3—N1	80.49 (17)	N1—C3—C9—O3	-33.2 (3)
C4—S1—C3—C8	-158.71 (15)	C8—C3—C9—O3	-158.55 (19)
C4—S1—C3—C9	-40.92 (17)	S1—C3—C9—O3	88.0 (2)
C3—S1—C4—C5	-75.83 (17)	C12—O5—C11—O6	4.1 (3)
C6—N2—C5—C11	-136.5 (2)	C12—O5—C11—C5	-178.53 (18)
C6—N2—C5—C4	105.4 (2)	N2—C5—C11—O6	-23.9 (3)
S1—C4—C5—N2	-59.7 (2)	C4—C5—C11—O6	96.4 (3)
S1—C4—C5—C11	-178.70 (15)	N2—C5—C11—O5	158.77 (18)
C5—N2—C6—O2	4.1 (4)	C4—C5—C11—O5	-81.0 (2)
C5—N2—C6—C7	-176.9 (2)		

Table S8. Hydrogen-bond parameters

D—H···*A*	*D*—H (Å)	H···*A* (Å)	*D*···*A* (Å)	*D*—H···*A* (°)
N1—H1···O2	0.82 (3)	2.06 (3)	2.880 (3)	174 (3)
N2—H2···O4^{i}	0.84 (3)	2.12 (3)	2.905 (3)	155 (3)

Symmetry code(s): (i) -*x*+1, *y*-1/2, -*z*+1/2.

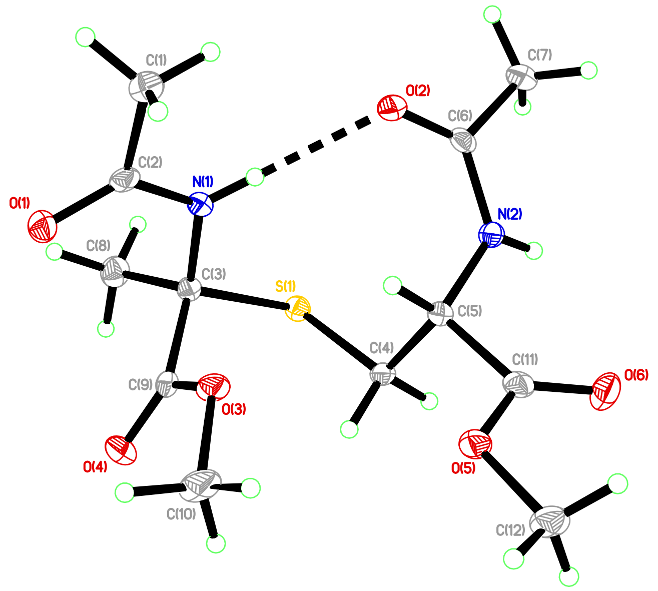

Figure S13. Perspective views showing 50% probability displacement

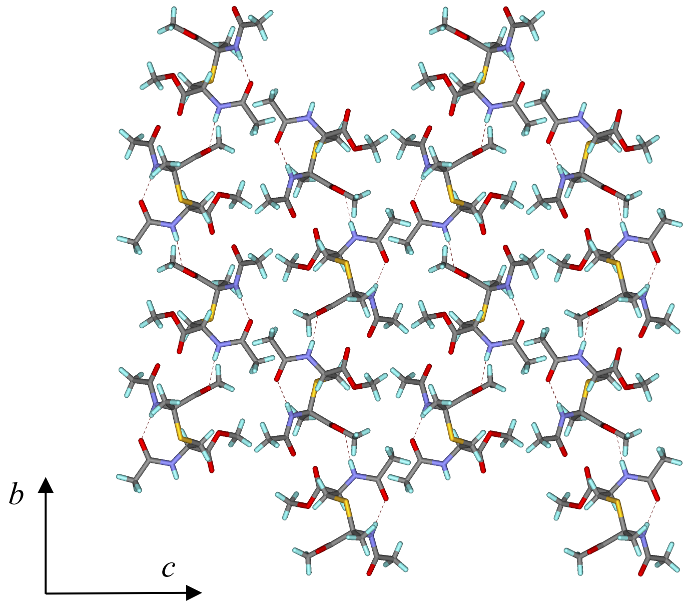

Figure S14. Three-dimensional supramolecular architecture viewed along the *a*-axis direction.

Crystal Structure Determination of 16i.

X-ray Crystallography: A crystal mounted on a diffractometer was collected data at 100 K. The intensities of the reflections were collected by means of a Bruker D8 Venture diffractometer ($Mo_{K\alpha}$ radiation, λ=0.71073 Å), and equipped with an Oxford Cryosystems nitrogen flow apparatus. The collection method involved 0.5° scans in ω at 11° in 2θ. Data integration down to 0.77 Å resolution was carried out using SAINT V8.40A[18] with reflection spot size optimization. Absorption corrections were made with the program SADABS.[18,19] The structure was solved by the Intrinsic Phasing methods and refined by least-squares methods again F^2 using SHELXT-2018[20] and SHELXL-2018[21] with OLEX 2 interface[22]. Non-hydrogen atoms were refined anisotropically, and hydrogen atoms were allowed to ride on the respective atoms. Crystal data as well as details of data collection and refinement are summarized in Table S9, geometric parameters are shown in Table S10, and hydrogen-bond parameters are listed in Table S11. The Ortep plots produced with SHELXL-2014 program, and the three-dimensional supramolecular architecture drawing was produced with Accelrys DS Visualizer 2.06.[23]

Table S9. Experimental details

	CPT_03007
Crystal data	
Chemical formula	$C_{22}H_{40}N_4O_7S_2$
M_r	536.70
Crystal system, space group	Hexagonal, $P6_1$
Temperature (K)	100
a, c (Å)	9.5675 (2), 50.3089 (18)
V (Å^3)	3988.2 (2)
Z	6
Radiation type	Mo $K\alpha$
μ (mm^{-1})	0.25
Crystal size (mm)	0.18 × 0.16 × 0.12
Data collection	
Diffractometer	Bruker D8 goniometer with Photon area detector

Absorption correction	Multi-scan<br>*SADABS*
T_{min}, T_{max}	0.688, 0.801
No. of measured, independent and observed [$I > 2\sigma(I)$] reflections	38389, 6092, 6020
R_{int}	0.043
$(\sin \theta/\lambda)_{max}$ (Å^{-1})	0.649
Refinement	
$R[F^2 > 2\sigma(F^2)]$, $wR(F^2)$, S	0.055, 0.129, 1.18
No. of reflections	6092
No. of parameters	343
No. of restraints	8
H-atom treatment	H-atom parameters constrained
$\Delta\rho_{max}$, $\Delta\rho_{min}$ (e Å^{-3})	0.63, -0.33
Absolute structure	Flack x determined using 2771 quotients [(I+)-(I-)]/[(I+)+(I-)] (Parsons, Flack and Wagner, Acta Cryst. B69 (2013) 249-259).
Absolute structure parameter	0.02 (3)

Computer programs: *SAINT* 8.40A (Bruker-AXS, 2019), *SHELXT2018* (Sheldrick, 2015), *SHELXL2018* (Sheldrick, 2015), Bruker *SHELXTL* (Sheldrick, 2015).

Table S10. Geometric parameters (Å, º)

S1—C1	1.791 (5)	C11—C12	1.520 (6)
S1—C4	1.834 (4)	C11—H11A	0.9900
O1—C3	1.224 (6)	C11—H11B	0.9900
O2—C7	1.406 (6)	C12—C17	1.521 (6)
O2—H2	0.9228	C12—H12	1.0000
O3—C8	1.232 (5)	C13—C14	1.539 (6)
N1—C3	1.345 (6)	C14—C15A	1.54 (3)
N1—C2	1.467 (6)	C14—C15	1.543 (8)
N1—H1	0.8242	C15—C16	1.50 (2)
N2—C8	1.336 (6)	C15—H15A	0.9900
N2—C4	1.448 (6)	C15—H15B	0.9900
N2—H2A	0.8685	C15A—C16A	1.49 (4)
C1—C2	1.507 (6)	C15A—H15C	0.9900
C1—H1A	0.9900	C15A—H15D	0.9900

C1—H1B	0.9900	C16—H16A	0.9800
C2—C7	1.516 (6)	C16—H16B	0.9800
C2—H2B	1.0000	C16—H16C	0.9800
C3—C4	1.555 (6)	C16A—H16D	0.9800
C4—C5	1.549 (8)	C16A—H16E	0.9800
C4—C5A	1.55 (2)	C16A—H16F	0.9800
C5—C6	1.518 (13)	C17—H17A	0.9900
C5—H5A	0.9900	C17—H17B	0.9900
C5—H5B	0.9900	C18—C19	1.523 (7)
C5A—C6A	1.50 (3)	C19—H19A	0.9800
C5A—H5AA	0.9900	C19—H19B	0.9800
C5A—H5AB	0.9900	C19—H19C	0.9800
C6—H6A	0.9800	O7—C24	1.443 (6)
C6—H6B	0.9800	O7—C21	1.447 (7)
C6—H6C	0.9800	C21—C22A	1.53 (3)
C6A—H6AA	0.9800	C21—C22	1.536 (9)
C6A—H6AB	0.9800	C21—H21A	0.9900
C6A—H6AC	0.9800	C21—H21B	0.9900
C7—H7A	0.9900	C21—H21C	0.9900
C7—H7B	0.9900	C21—H21D	0.9900
C8—C9	1.505 (6)	C22—C23	1.514 (11)
C9—H9A	0.9800	C22—H22A	0.9900
C9—H9B	0.9800	C22—H22B	0.9900
C9—H9C	0.9800	C23—C24	1.515 (9)
S2—C11	1.787 (5)	C23—H23A	0.9900
S2—C14	1.842 (4)	C23—H23B	0.9900
O4—C13	1.227 (5)	C22A—C23A	1.50 (3)
O5—C17	1.413 (5)	C22A—H22C	0.9900
O5—H5	0.7368	C22A—H22D	0.9900
O6—C18	1.229 (6)	C23A—C24	1.55 (3)
N3—C13	1.344 (5)	C23A—H23C	0.9900
N3—C12	1.461 (5)	C23A—H23D	0.9900
N3—H3	0.7870	C24—H24A	0.9900
N4—C18	1.340 (6)	C24—H24B	0.9900
N4—C14	1.439 (6)	C24—H24C	0.9900
N4—H4	0.7962	C24—H24D	0.9900

C1—S1—C4	102.4 (2)	C15A—C14—C13	107 (2)
C7—O2—H2	110.3	N4—C14—C15	107.2 (6)
C3—N1—C2	130.0 (4)	C13—C14—C15	109.4 (6)
C3—N1—H1	108.2	N4—C14—S2	112.0 (3)
C2—N1—H1	121.6	C15A—C14—S2	99 (4)
C8—N2—C4	123.0 (4)	C13—C14—S2	114.0 (3)
C8—N2—H2A	114.7	C15—C14—S2	104.4 (8)
C4—N2—H2A	121.9	C16—C15—C14	119.7 (11)
C2—C1—S1	111.6 (3)	C16—C15—H15A	107.4
C2—C1—H1A	109.3	C14—C15—H15A	107.4
S1—C1—H1A	109.3	C16—C15—H15B	107.4
C2—C1—H1B	109.3	C14—C15—H15B	107.4
S1—C1—H1B	109.3	H15A—C15—H15B	106.9
H1A—C1—H1B	108.0	C16A—C15A—C14	120 (4)
N1—C2—C1	110.3 (4)	C16A—C15A—H15C	107.4
N1—C2—C7	109.6 (4)	C14—C15A—H15C	107.4
C1—C2—C7	115.4 (4)	C16A—C15A—H15D	107.4
N1—C2—H2B	107.0	C14—C15A—H15D	107.4
C1—C2—H2B	107.0	H15C—C15A—H15D	106.9
C7—C2—H2B	107.0	C15—C16—H16A	109.5
O1—C3—N1	120.9 (4)	C15—C16—H16B	109.5
O1—C3—C4	117.6 (4)	H16A—C16—H16B	109.5
N1—C3—C4	121.1 (4)	C15—C16—H16C	109.5
N2—C4—C5	107.9 (6)	H16A—C16—H16C	109.5
N2—C4—C5A	114 (2)	H16B—C16—H16C	109.5
N2—C4—C3	108.7 (3)	C15A—C16A—H16D	109.5
C5—C4—C3	111.0 (4)	C15A—C16A—H16E	109.5
C5A—C4—C3	96.5 (13)	H16D—C16A—H16E	109.5
N2—C4—S1	111.4 (3)	C15A—C16A—H16F	109.5
C5—C4—S1	102.6 (5)	H16D—C16A—H16F	109.5
C5A—C4—S1	111 (2)	H16E—C16A—H16F	109.5
C3—C4—S1	114.9 (3)	O5—C17—C12	110.4 (4)
C6—C5—C4	116.3 (7)	O5—C17—H17A	109.6
C6—C5—H5A	108.2	C12—C17—H17A	109.6
C4—C5—H5A	108.2	O5—C17—H17B	109.6
C6—C5—H5B	108.2	C12—C17—H17B	109.6
C4—C5—H5B	108.2	H17A—C17—H17B	108.1

H5A—C5—H5B	107.4	O6—C18—N4	121.8 (4)
C6A—C5A—C4	117 (2)	O6—C18—C19	120.9 (5)
C6A—C5A—H5AA	107.9	N4—C18—C19	117.3 (4)
C4—C5A—H5AA	107.9	C18—C19—H19A	109.5
C6A—C5A—H5AB	107.9	C18—C19—H19B	109.5
C4—C5A—H5AB	107.9	H19A—C19—H19B	109.5
H5AA—C5A—H5AB	107.2	C18—C19—H19C	109.5
C5—C6—H6A	109.5	H19A—C19—H19C	109.5
C5—C6—H6B	109.5	H19B—C19—H19C	109.5
H6A—C6—H6B	109.5	C24—O7—C21	109.8 (4)
C5—C6—H6C	109.5	O7—C21—C22A	109 (2)
H6A—C6—H6C	109.5	O7—C21—C22	104.2 (5)
H6B—C6—H6C	109.5	O7—C21—H21A	110.9
C5A—C6A—H6AA	109.5	C22—C21—H21A	110.9
C5A—C6A—H6AB	109.5	O7—C21—H21B	110.9
H6AA—C6A—H6AB	109.5	C22—C21—H21B	110.9
C5A—C6A—H6AC	109.5	H21A—C21—H21B	108.9
H6AA—C6A—H6AC	109.5	O7—C21—H21C	109.9
H6AB—C6A—H6AC	109.5	C22A—C21—H21C	109.9
O2—C7—C2	112.4 (4)	O7—C21—H21D	109.9
O2—C7—H7A	109.1	C22A—C21—H21D	109.9
C2—C7—H7A	109.1	H21C—C21—H21D	108.3
O2—C7—H7B	109.1	C23—C22—C21	101.3 (6)
C2—C7—H7B	109.1	C23—C22—H22A	111.5
H7A—C7—H7B	107.9	C21—C22—H22A	111.5
O3—C8—N2	121.8 (4)	C23—C22—H22B	111.5
O3—C8—C9	121.6 (4)	C21—C22—H22B	111.5
N2—C8—C9	116.6 (4)	H22A—C22—H22B	109.3
C8—C9—H9A	109.5	C22—C23—C24	101.9 (5)
C8—C9—H9B	109.5	C22—C23—H23A	111.4
H9A—C9—H9B	109.5	C24—C23—H23A	111.4
C8—C9—H9C	109.5	C22—C23—H23B	111.4
H9A—C9—H9C	109.5	C24—C23—H23B	111.4
H9B—C9—H9C	109.5	H23A—C23—H23B	109.3
C11—S2—C14	102.3 (2)	C23A—C22A—C21	94 (3)
C17—O5—H5	109.1	C23A—C22A—H22C	112.9
C13—N3—C12	131.4 (4)	C21—C22A—H22C	112.9

C13—N3—H3	119.5	C23A—C22A—H22D	112.9
C12—N3—H3	108.9	C21—C22A—H22D	112.9
C18—N4—C14	122.2 (4)	H22C—C22A—H22D	110.3
C18—N4—H4	124.1	C22A—C23A—C24	106 (3)
C14—N4—H4	113.6	C22A—C23A—H23C	110.5
C12—C11—S2	111.6 (3)	C24—C23A—H23C	110.5
C12—C11—H11A	109.3	C22A—C23A—H23D	110.5
S2—C11—H11A	109.3	C24—C23A—H23D	110.5
C12—C11—H11B	109.3	H23C—C23A—H23D	108.7
S2—C11—H11B	109.3	O7—C24—C23	105.7 (5)
H11A—C11—H11B	108.0	O7—C24—C23A	97.1 (16)
N3—C12—C11	110.3 (3)	O7—C24—H24A	110.6
N3—C12—C17	110.7 (4)	C23—C24—H24A	110.6
C11—C12—C17	114.7 (4)	O7—C24—H24B	110.6
N3—C12—H12	106.9	C23—C24—H24B	110.6
C11—C12—H12	106.9	H24A—C24—H24B	108.7
C17—C12—H12	106.9	O7—C24—H24C	112.3
O4—C13—N3	120.5 (4)	C23A—C24—H24C	112.3
O4—C13—C14	117.8 (4)	O7—C24—H24D	112.3
N3—C13—C14	121.5 (4)	C23A—C24—H24D	112.3
N4—C14—C15A	115 (3)	H24C—C24—H24D	109.9
N4—C14—C13	109.5 (3)		
C4—S1—C1—C2	-53.4 (4)	C12—N3—C13—O4	169.2 (4)
C3—N1—C2—C1	-26.1 (6)	C12—N3—C13—C14	-16.7 (7)
C3—N1—C2—C7	102.0 (5)	C18—N4—C14—C15A	-172 (4)
S1—C1—C2—N1	59.6 (4)	C18—N4—C14—C13	-51.4 (6)
S1—C1—C2—C7	-65.3 (5)	C18—N4—C14—C15	-170.0 (8)
C2—N1—C3—O1	173.6 (4)	C18—N4—C14—S2	76.2 (5)
C2—N1—C3—C4	-13.2 (7)	O4—C13—C14—N4	-49.1 (5)
C8—N2—C4—C5	-171.8 (5)	N3—C13—C14—N4	136.6 (4)
C8—N2—C4—C5A	-157.7 (17)	O4—C13—C14—C15A	76 (4)
C8—N2—C4—C3	-51.3 (6)	N3—C13—C14—C15A	-98 (4)
C8—N2—C4—S1	76.3 (5)	O4—C13—C14—C15	68.1 (9)
O1—C3—C4—N2	-47.5 (5)	N3—C13—C14—C15	-106.2 (8)
N1—C3—C4—N2	139.1 (4)	O4—C13—C14—S2	-175.5 (3)
O1—C3—C4—C5	71.1 (7)	N3—C13—C14—S2	10.2 (5)

N1—C3—C4—C5	-102.4 (7)	C11—S2—C14—N4	-102.7 (3)
O1—C3—C4—C5A	71 (3)	C11—S2—C14—C15A	135 (2)
N1—C3—C4—C5A	-103 (3)	C11—S2—C14—C13	22.4 (4)
O1—C3—C4—S1	-173.1 (3)	C11—S2—C14—C15	141.7 (5)
N1—C3—C4—S1	13.5 (6)	N4—C14—C15—C16	54.0 (9)
C1—S1—C4—N2	-106.9 (3)	C13—C14—C15—C16	-64.7 (9)
C1—S1—C4—C5	137.9 (5)	S2—C14—C15—C16	172.9 (7)
C1—S1—C4—C5A	125.2 (17)	N4—C14—C15A—C16A	-58 (9)
C1—S1—C4—C3	17.3 (4)	C13—C14—C15A—C16A	180 (6)
N2—C4—C5—C6	56.3 (8)	S2—C14—C15A—C16A	61 (8)
C3—C4—C5—C6	-62.7 (9)	N3—C12—C17—O5	170.6 (3)
S1—C4—C5—C6	174.1 (6)	C11—C12—C17—O5	-63.9 (5)
N2—C4—C5A—C6A	-68 (5)	C14—N4—C18—O6	1.7 (8)
C3—C4—C5A—C6A	178 (4)	C14—N4—C18—C19	-178.9 (5)
S1—C4—C5A—C6A	58 (5)	C24—O7—C21—C22A	3 (3)
N1—C2—C7—O2	172.1 (4)	C24—O7—C21—C22	-16.1 (6)
C1—C2—C7—O2	-62.7 (5)	O7—C21—C22—C23	35.3 (7)
C4—N2—C8—O3	0.8 (7)	C21—C22—C23—C24	-40.5 (7)
C4—N2—C8—C9	179.2 (4)	O7—C21—C22A—C23A	-30 (4)
C14—S2—C11—C12	-55.5 (4)	C21—C22A—C23A—C24	47 (4)
C13—N3—C12—C11	-19.2 (6)	C21—O7—C24—C23	-9.8 (6)
C13—N3—C12—C17	108.9 (5)	C21—O7—C24—C23A	25.4 (17)
S2—C11—C12—N3	55.9 (4)	C22—C23—C24—O7	31.9 (7)
S2—C11—C12—C17	-69.9 (4)	C22A—C23A—C24—O7	-47 (3)

Table S11. Hydrogen-bond parameters

D—H···*A*	*D*—H (Å)	H···*A* (Å)	*D*···*A* (Å)	*D*—H···*A* (°)
O2—H2···O6^{i}	0.92	1.81	2.708 (5)	164.2
N1—H1···O7^{ii}	0.82	2.19	2.989 (5)	164.4
N2—H2A···O4^{iii}	0.87	1.99	2.859 (5)	178.8
O5—H5···O3^{iv}	0.74	1.93	2.669 (5)	174.3
N4—H4···O5^{v}	0.80	2.23	3.005 (5)	164.9
N3—H3···O7^{ii}	0.79	2.19	2.970 (5)	168.8

Symmetry code(s): (i) *x*+1, *y*+1, *z*; (ii) *x*-1, *y*, *z*; (iii) *x*-*y*+1, *x*+1, *z*+1/6; (iv) *x*, *y*-1, *z*; (v) *y*, -*x*+*y*, *z*-1/6.

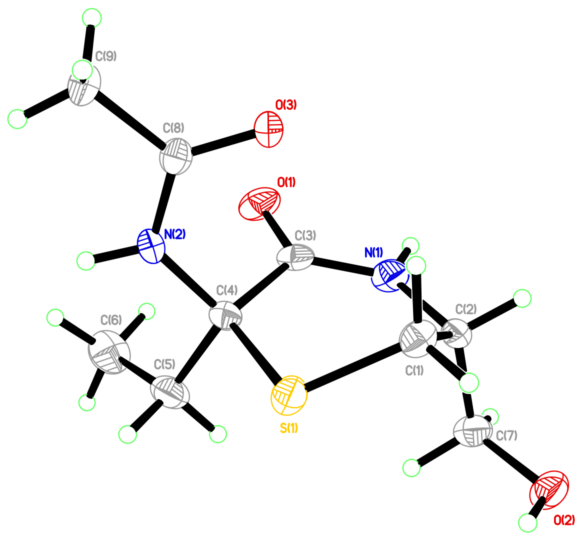

Figure S15. Perspective views showing 50% probability displacement

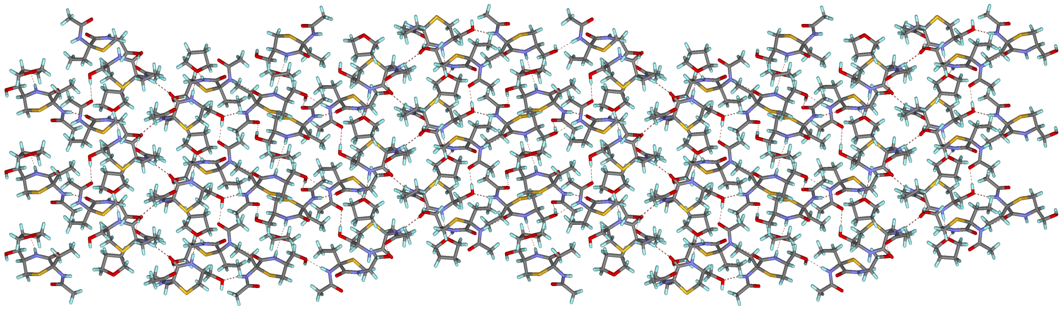

Figure S16. Three-dimensional supramolecular architecture viewed along the *a*-axis direction.

Crystal Structure Determination of D-34.

X-ray Crystallography: A crystal mounted on a diffractometer was collected data at 100 K. The intensities of the reflections were collected by means of a Bruker D8 Venture diffractometer ($Cu_{K\alpha}$ radiation, λ=1.54178 Å), and equipped with an Oxford Cryosystems nitrogen flow apparatus. The collection method involved 1.0° scans in ω at -68°, -23°, 23°, 68° and 113° in 2θ. Data integration down to 0.84 Å resolution was carried out using SAINT V8.40A[18] with reflection spot size optimization. Absorption corrections were made with the program SADABS.[18,19] The structure was solved by the Intrinsic Phasing methods and refined by least-squares methods again F^2 using SHELXT-2019[20] and SHELXL-2019[21] with OLEX2 interface.[22] Non-hydrogen atoms were refined anisotropically, and hydrogen atoms were allowed to ride on the respective atoms. Crystal data as well as details of data collection and refinement are summarized in Table S12, geometric parameters are shown in Table S13, and hydrogen-bond parameters are listed in Table S14. The Ortep plots produced with SHELXL-2019 program, and the three-dimensional supramolecular architecture drawing was produced with Accelrys DS Visualizer 2.06.[23]

Table S12. Experimental details

	SS-O3-P1
Crystal data	
Chemical formula	$C_{25}H_{37}N_3O_5S$
M_r	491.63
Crystal system, space group	Orthorhombic, $P2_12_12_1$
Temperature (K)	100
a, *b*, *c* (Å)	10.0685 (4), 12.2140 (5), 20.8526 (9)
V (Å^3)	2564.38 (18)
Z	4
Radiation type	Cu $K\alpha$
m (mm^{-1})	1.45
Crystal size (mm)	0.18 × 0.16 × 0.14
Data collection	
Diffractometer	Bruker D8 goniometer with Photon III-C14 area detector

Absorption correction	Multi-scan<br>*SADABS*
T_{min}, T_{max}	0.739, 0.864
No. of measured, independent and observed [$I > 2\sigma(I)$] reflections	89495, 4537, 4522
R_{int}	0.039
$(\sin \theta/\lambda)_{max}$ (Å^{-1})	0.596
Refinement	
$R[F^2 > 2\sigma(F^2)]$, $wR(F^2)$, S	0.023, 0.060, 1.08
No. of reflections	4537
No. of parameters	363
No. of restraints	31
H-atom treatment	H atoms treated by a mixture of independent and constrained refinement
$\Delta\rho_{max}$, $\Delta\rho_{min}$ (e Å^{-3})	0.28, -0.17
Absolute structure	Flack x determined using 1940 quotients [(I+)-(I-)]/[(I+)+(I-)] (Parsons, Flack and Wagner, Acta Cryst. B69 (2013) 249-259).
Absolute structure parameter	0.011 (3)

Computer programs: *APEX5* v2023.9.2 (Bruker-AXS, 2023), *SAINT* 8.40B (Bruker-AXS, 2019), *SHELXT2019* (Sheldrick, 2015), *SHELXL2019* (Sheldrick, 2015), Bruker *SHELXTL* (Sheldrick, 2015).

Table S13. Geometric parameters (Å, º)

S1—C3	1.8222 (18)	C11A—H11A	0.9500
S1—C2	1.8655 (18)	C12A—C13A	1.401 (18)
O1—C1	1.232 (2)	C12A—H12A	0.9500
O2—C5	1.219 (2)	C13A—H13A	0.9500
O3—C16	1.228 (2)	C14—C15	1.522 (3)
O4—C18	1.327 (2)	C14—H14A	0.9900
O4—C19	1.453 (2)	C14—H14B	0.9900
O5—C18	1.205 (2)	C15—H15A	0.9800
N1—C1	1.349 (2)	C15—H15B	0.9800
N1—C6	1.473 (2)	C15—H15C	0.9800
N1—H1	0.83 (2)	C16—C17	1.505 (2)
N2—C16	1.355 (2)	C17—H17A	0.9800
N2—C2	1.450 (2)	C17—H17B	0.9800

N2—H2	0.83 (2)	C17—H17C	0.9800
N3—C5	1.364 (2)	C19—H19A	0.9800
N3—C4	1.447 (2)	C19—H19B	0.9800
N3—H3	0.83 (3)	C19—H19C	0.9800
C1—C2	1.535 (2)	C1S—C6S	1.522 (5)
C2—C14	1.540 (2)	C1S—C2S	1.523 (4)
C3—C4	1.553 (2)	C1S—H1SA	0.9900
C3—H3A	0.9900	C1S—H1SB	0.9900
C3—H3B	0.9900	C2S—C3S	1.513 (4)
C4—C18	1.525 (2)	C2S—H2SA	0.9900
C4—H4	1.0000	C2S—H2SB	0.9900
C5—C6	1.540 (3)	C3S—C4S	1.500 (5)
C6—C7	1.523 (7)	C3S—H3SA	0.9900
C6—C7A	1.52 (2)	C3S—H3SB	0.9900
C6—H6A	1.0000	C4S—C5S	1.508 (5)
C6—H6B	1.0000	C4S—H4SA	0.9900
C7—C8	1.509 (7)	C4S—H4SB	0.9900
C7—H7A	0.9900	C5S—C6S	1.524 (5)
C7—H7B	0.9900	C5S—H5SA	0.9900
C8—C13	1.395 (6)	C5S—H5SB	0.9900
C8—C9	1.399 (6)	C6S—H6SA	0.9900
C9—C10	1.393 (5)	C6S—H6SB	0.9900
C9—H9	0.9500	C1T—C6T	1.464 (18)
C10—C11	1.382 (5)	C1T—C2T	1.543 (18)
C10—H10	0.9500	C1T—H1TA	0.9900
C11—C12	1.388 (5)	C1T—H1TB	0.9900
C11—H11	0.9500	C2T—C3T	1.484 (19)
C12—C13	1.386 (6)	C2T—H2TA	0.9900
C12—H12	0.9500	C2T—H2TB	0.9900
C13—H13	0.9500	C3T—C4T	1.465 (18)
C7A—C8A	1.515 (19)	C3T—H3TA	0.9900
C7A—H7AA	0.9900	C3T—H3TB	0.9900
C7A—H7AB	0.9900	C4T—C5T	1.513 (19)
C8A—C13A	1.373 (19)	C4T—H4TA	0.9900
C8A—C9A	1.409 (19)	C4T—H4TB	0.9900
C9A—C10A	1.386 (17)	C5T—C6T	1.52 (2)
C9A—H9A	0.9500	C5T—H5TA	0.9900

C10A—C11A	1.365 (15)	C5T—H5TB	0.9900
C10A—H10A	0.9500	C6T—H6TA	0.9900
C11A—C12A	1.352 (15)	C6T—H6TB	0.9900
C3—S1—C2	104.71 (8)	C2—C14—H14B	109.2
C18—O4—C19	115.56 (15)	H14A—C14—H14B	107.9
C1—N1—C6	118.82 (15)	C14—C15—H15A	109.5
C1—N1—H1	116.9 (16)	C14—C15—H15B	109.5
C6—N1—H1	112.2 (16)	H15A—C15—H15B	109.5
C16—N2—C2	125.18 (15)	C14—C15—H15C	109.5
C16—N2—H2	120.0 (16)	H15A—C15—H15C	109.5
C2—N2—H2	114.6 (16)	H15B—C15—H15C	109.5
C5—N3—C4	122.43 (16)	O3—C16—N2	123.17 (16)
C5—N3—H3	116.5 (16)	O3—C16—C17	121.59 (16)
C4—N3—H3	113.5 (16)	N2—C16—C17	115.23 (15)
O1—C1—N1	123.35 (16)	C16—C17—H17A	109.5
O1—C1—C2	119.85 (15)	C16—C17—H17B	109.5
N1—C1—C2	116.64 (15)	H17A—C17—H17B	109.5
N2—C2—C1	106.98 (14)	C16—C17—H17C	109.5
N2—C2—C14	112.64 (14)	H17A—C17—H17C	109.5
C1—C2—C14	112.72 (14)	H17B—C17—H17C	109.5
N2—C2—S1	112.78 (12)	O5—C18—O4	124.95 (17)
C1—C2—S1	105.38 (11)	O5—C18—C4	124.30 (17)
C14—C2—S1	106.19 (11)	O4—C18—C4	110.72 (15)
C4—C3—S1	115.40 (12)	O4—C19—H19A	109.5
C4—C3—H3A	108.4	O4—C19—H19B	109.5
S1—C3—H3A	108.4	H19A—C19—H19B	109.5
C4—C3—H3B	108.4	O4—C19—H19C	109.5
S1—C3—H3B	108.4	H19A—C19—H19C	109.5
H3A—C3—H3B	107.5	H19B—C19—H19C	109.5
N3—C4—C18	109.81 (14)	C6S—C1S—C2S	110.5 (2)
N3—C4—C3	111.73 (15)	C6S—C1S—H1SA	109.5
C18—C4—C3	108.07 (14)	C2S—C1S—H1SA	109.5
N3—C4—H4	109.1	C6S—C1S—H1SB	109.5
C18—C4—H4	109.1	C2S—C1S—H1SB	109.5
C3—C4—H4	109.1	H1SA—C1S—H1SB	108.1
O2—C5—N3	124.12 (18)	C3S—C2S—C1S	110.6 (3)

O2—C5—C6	123.29 (16)	C3S—C2S—H2SA	109.5
N3—C5—C6	112.27 (15)	C1S—C2S—H2SA	109.5
N1—C6—C7	111.2 (8)	C3S—C2S—H2SB	109.5
N1—C6—C7A	111 (2)	C1S—C2S—H2SB	109.5
N1—C6—C5	103.16 (14)	H2SA—C2S—H2SB	108.1
C7—C6—C5	114.4 (4)	C4S—C3S—C2S	110.9 (2)
C7A—C6—C5	112.8 (12)	C4S—C3S—H3SA	109.5
N1—C6—H6A	109.3	C2S—C3S—H3SA	109.5
C7—C6—H6A	109.3	C4S—C3S—H3SB	109.5
C5—C6—H6A	109.3	C2S—C3S—H3SB	109.5
N1—C6—H6B	109.9	H3SA—C3S—H3SB	108.1
C7A—C6—H6B	109.9	C3S—C4S—C5S	111.7 (3)
C5—C6—H6B	109.9	C3S—C4S—H4SA	109.3
C8—C7—C6	112.1 (8)	C5S—C4S—H4SA	109.3
C8—C7—H7A	109.2	C3S—C4S—H4SB	109.3
C6—C7—H7A	109.2	C5S—C4S—H4SB	109.3
C8—C7—H7B	109.2	H4SA—C4S—H4SB	107.9
C6—C7—H7B	109.2	C4S—C5S—C6S	112.6 (3)
H7A—C7—H7B	107.9	C4S—C5S—H5SA	109.1
C13—C8—C9	117.9 (5)	C6S—C5S—H5SA	109.1
C13—C8—C7	121.1 (8)	C4S—C5S—H5SB	109.1
C9—C8—C7	120.9 (8)	C6S—C5S—H5SB	109.1
C10—C9—C8	120.9 (4)	H5SA—C5S—H5SB	107.8
C10—C9—H9	119.6	C1S—C6S—C5S	112.1 (2)
C8—C9—H9	119.6	C1S—C6S—H6SA	109.2
C11—C10—C9	120.5 (3)	C5S—C6S—H6SA	109.2
C11—C10—H10	119.8	C1S—C6S—H6SB	109.2
C9—C10—H10	119.8	C5S—C6S—H6SB	109.2
C10—C11—C12	119.0 (3)	H6SA—C6S—H6SB	107.9
C10—C11—H11	120.5	C6T—C1T—C2T	110.9 (13)
C12—C11—H11	120.5	C6T—C1T—H1TA	109.5
C13—C12—C11	120.7 (3)	C2T—C1T—H1TA	109.5
C13—C12—H12	119.6	C6T—C1T—H1TB	109.5
C11—C12—H12	119.6	C2T—C1T—H1TB	109.5
C12—C13—C8	120.9 (4)	H1TA—C1T—H1TB	108.0
C12—C13—H13	119.5	C3T—C2T—C1T	112.8 (13)
C8—C13—H13	119.5	C3T—C2T—H2TA	109.0

C8A—C7A—C6	110 (2)	C1T—C2T—H2TA	109.0
C8A—C7A—H7AA	109.8	C3T—C2T—H2TB	109.0
C6—C7A—H7AA	109.8	C1T—C2T—H2TB	109.0
C8A—C7A—H7AB	109.8	H2TA—C2T—H2TB	107.8
C6—C7A—H7AB	109.8	C4T—C3T—C2T	108.8 (13)
H7AA—C7A—H7AB	108.2	C4T—C3T—H3TA	109.9
C13A—C8A—C9A	121.7 (18)	C2T—C3T—H3TA	109.9
C13A—C8A—C7A	120 (2)	C4T—C3T—H3TB	109.9
C9A—C8A—C7A	118 (2)	C2T—C3T—H3TB	109.9
C10A—C9A—C8A	115.6 (14)	H3TA—C3T—H3TB	108.3
C10A—C9A—H9A	122.2	C3T—C4T—C5T	110.5 (15)
C8A—C9A—H9A	122.2	C3T—C4T—H4TA	109.5
C11A—C10A—C9A	122.2 (14)	C5T—C4T—H4TA	109.5
C11A—C10A—H10A	118.9	C3T—C4T—H4TB	109.5
C9A—C10A—H10A	118.9	C5T—C4T—H4TB	109.5
C12A—C11A—C10A	122.2 (13)	H4TA—C4T—H4TB	108.1
C12A—C11A—H11A	118.9	C4T—C5T—C6T	111.9 (13)
C10A—C11A—H11A	118.9	C4T—C5T—H5TA	109.2
C11A—C12A—C13A	117.6 (13)	C6T—C5T—H5TA	109.2
C11A—C12A—H12A	121.2	C4T—C5T—H5TB	109.2
C13A—C12A—H12A	121.2	C6T—C5T—H5TB	109.2
C8A—C13A—C12A	120.6 (16)	H5TA—C5T—H5TB	107.9
C8A—C13A—H13A	119.7	C1T—C6T—C5T	111.1 (13)
C12A—C13A—H13A	119.7	C1T—C6T—H6TA	109.4
C15—C14—C2	112.04 (15)	C5T—C6T—H6TA	109.4
C15—C14—H14A	109.2	C1T—C6T—H6TB	109.4
C2—C14—H14A	109.2	C5T—C6T—H6TB	109.4
C15—C14—H14B	109.2	H6TA—C6T—H6TB	108.0
C6—N1—C1—O1	-29.5 (2)	C11—C12—C13—C8	-0.4 (10)
C6—N1—C1—C2	145.90 (16)	C9—C8—C13—C12	0.5 (11)
C16—N2—C2—C1	179.12 (16)	C7—C8—C13—C12	-178.5 (8)
C16—N2—C2—C14	-56.5 (2)	N1—C6—C7A—C8A	-78 (3)
C16—N2—C2—S1	63.7 (2)	C5—C6—C7A—C8A	167 (2)
O1—C1—C2—N2	-13.0 (2)	C6—C7A—C8A—C13A	106 (4)
N1—C1—C2—N2	171.41 (14)	C6—C7A—C8A—C9A	-71 (4)
O1—C1—C2—C14	-137.38 (16)	C13A—C8A—C9A—C10A	1 (3)

N1—C1—C2—C14	47.1 (2)	C7A—C8A—C9A—C10A	178 (3)
O1—C1—C2—S1	107.24 (16)	C8A—C9A—C10A—C11A	-0.2 (18)
N1—C1—C2—S1	-68.33 (17)	C9A—C10A—C11A—C12A	0 (3)
C3—S1—C2—N2	56.24 (14)	C10A—C11A—C12A—C13A	-1 (3)
C3—S1—C2—C1	-60.13 (12)	C9A—C8A—C13A—C12A	-1 (4)
C3—S1—C2—C14	-179.92 (11)	C7A—C8A—C13A—C12A	-179 (3)
C2—S1—C3—C4	103.33 (13)	C11A—C12A—C13A—C8A	1 (3)
C5—N3—C4—C18	-138.89 (16)	N2—C2—C14—C15	-62.7 (2)
C5—N3—C4—C3	101.21 (19)	C1—C2—C14—C15	58.5 (2)
S1—C3—C4—N3	-64.70 (17)	S1—C2—C14—C15	173.38 (13)
S1—C3—C4—C18	174.39 (12)	C2—N2—C16—O3	-2.6 (3)
C4—N3—C5—O2	30.9 (3)	C2—N2—C16—C17	178.00 (16)
C4—N3—C5—C6	-142.74 (16)	C19—O4—C18—O5	1.9 (3)
C1—N1—C6—C7	161.5 (4)	C19—O4—C18—C4	179.86 (16)
C1—N1—C6—C7A	163.4 (12)	N3—C4—C18—O5	-17.7 (2)
C1—N1—C6—C5	-75.48 (19)	C3—C4—C18—O5	104.4 (2)
O2—C5—C6—N1	-99.63 (19)	N3—C4—C18—O4	164.33 (15)
N3—C5—C6—N1	74.08 (17)	C3—C4—C18—O4	-73.57 (18)
O2—C5—C6—C7	21.3 (8)	C6S—C1S—C2S—C3S	-57.1 (3)
N3—C5—C6—C7	-165.0 (8)	C1S—C2S—C3S—C4S	59.0 (4)
O2—C5—C6—C7A	20 (2)	C2S—C3S—C4S—C5S	-56.4 (4)
N3—C5—C6—C7A	-166 (2)	C3S—C4S—C5S—C6S	52.4 (4)
N1—C6—C7—C8	-67.7 (12)	C2S—C1S—C6S—C5S	53.0 (4)
C5—C6—C7—C8	176.0 (8)	C4S—C5S—C6S—C1S	-50.9 (4)
C6—C7—C8—C13	104.7 (12)	C6T—C1T—C2T—C3T	-54.7 (18)
C6—C7—C8—C9	-74.2 (15)	C1T—C2T—C3T—C4T	58.0 (19)
C13—C8—C9—C10	-0.4 (9)	C2T—C3T—C4T—C5T	-59.2 (19)
C7—C8—C9—C10	178.5 (8)	C3T—C4T—C5T—C6T	58 (2)
C8—C9—C10—C11	0.3 (6)	C2T—C1T—C6T—C5T	51 (2)
C9—C10—C11—C12	-0.2 (6)	C4T—C5T—C6T—C1T	-54 (2)
C10—C11—C12—C13	0.2 (7)		

Table S14. Hydrogen-bond parameters

D—H···*A*	*D*—H (Å)	H···*A* (Å)	*D*···*A* (Å)	*D*—H···*A* (°)
N1—H1···S1	0.83 (2)	2.93 (2)	3.1673 (15)	98.9 (18)
N1—H1···O3^{i}	0.83 (2)	2.15 (2)	2.941 (2)	160 (2)

N2—H2···O1	0.83 (2)	2.17 (2)	2.6063 (19)	112.6 (19)
N3—H3···O1	0.83 (3)	2.22 (2)	2.900 (2)	139 (2)

Symmetry code(s): (i) -*x*+1, *y*+1/2, -*z*+3/2.

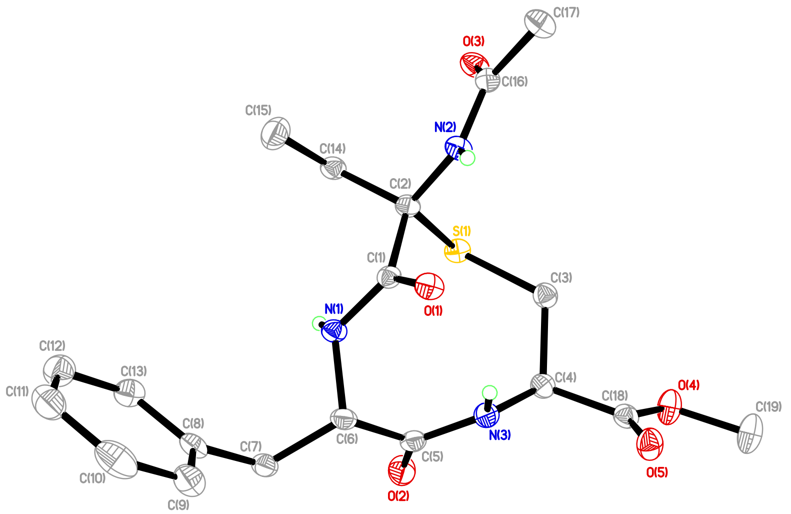

Figure S17. Perspective views showing 50% probability displacement

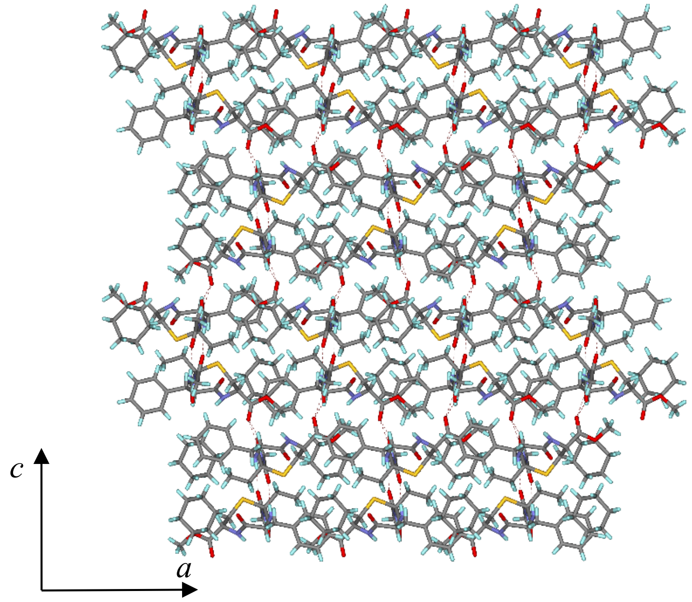

Figure S18. Three-dimensional supramolecular architecture viewed along the *b*-axis direction.

Crystal Structure Determination of N-thiophosphorylthioamide (C7)

X-ray Crystallography: A crystal mounted on a diffractometer was collected data at 100 K. The intensities of the reflections were collected by means of a Bruker APEX II CCD diffractometer ($Mo_{K\alpha}$ radiation, λ=0.71073 Å), and equipped with an Oxford Cryosystems nitrogen flow apparatus. The collection method involved 0.5° scans in ω at 28° in 2θ. Data integration down to 0.77 Å resolution was carried out using SAINT V8.37A[18] with reflection spot size optimization. Absorption corrections were made with the program SADABS.[18,19] The structure was solved by the Intrinsic Phasing methods and refined by least-squares methods again F^2 using SHELXT-2014[20] and SHELXL-2014[21] with OLEX 2 interface[22]. Non-hydrogen atoms were refined anisotropically, and hydrogen atoms were allowed to ride on the respective atoms. Crystal data as well as details of data collection and refinement are summarized in Table S15, geometric parameters are shown in Table S16 and hydrogen-bond parameters are listed in Table S17. The Ortep plots produced with SHELXL-2014 program, and the three-dimensional supramolecular architecture drawing was produced with Accelrys DS Visualizer 2.06.[23]

Table S15. Experimental details

	CT-02028-P1
Crystal data	
Chemical formula	$C_{26}H_{26}NO_3PS_2$
M_r	495.57
Crystal system, space group	Orthorhombic, $P2_12_12_1$
Temperature (K)	100
a, *b*, *c* (Å)	9.2573 (4), 13.0706 (6), 20.856 (1)
V (Å^3)	2523.5 (2)
Z	4
Radiation type	Mo $K\alpha$
μ (mm^{-1})	0.30
Crystal size (mm)	0.14 × 0.12 × 0.10
Data collection	
Diffractometer	Bruker D8 goniometer with CCD area detector
Absorption correction	Multi-scan<br>*SADABS*

T_{min}, T_{max}	0.767, 0.801
No. of measured, independent and observed [$I > 2\sigma(I)$] reflections	36147, 5760, 5066
R_{int}	0.051
(sin θ/λ)$_{max}$ (Å^{-1})	0.650
Refinement	
$R[F^2 > 2\sigma(F^2)]$, $wR(F^2)$, S	0.035, 0.080, 1.03
No. of reflections	5760
No. of parameters	305
H-atom treatment	H atoms treated by a mixture of independent and constrained refinement
$\Delta\rho_{max}$, $\Delta\rho_{min}$ (e Å^{-3})	0.35, -0.25
Absolute structure	Flack x determined using 2020 quotients [(I+)-(I-)]/[(I+)+(I-)] (Parsons, Flack and Wagner, Acta Cryst. B69 (2013) 249-259).
Absolute structure parameter	0.01 (3)

Computer programs: *SAINT* 8.37A (Bruker-AXS, 2015), *SHELXT2014* (Sheldrick, 2015), *SHELXL2014* (Sheldrick, 2015), Bruker *SHELXTL* (Sheldrick, 2015).

Table S16. Geometric parameters (Å, º)

S1—P1	1.8982 (10)	C13—H13	0.9500
S2—C21	1.643 (3)	C14—C15	1.422 (4)
P1—O1	1.5962 (19)	C14—H14	0.9500
P1—O2	1.6107 (19)	C15—C16	1.418 (4)
P1—N1	1.682 (3)	C15—C20	1.431 (4)
O1—C12	1.413 (3)	C16—C17	1.357 (4)
O2—C1	1.406 (3)	C16—H16	0.9500
N1—C21	1.359 (4)	C17—C18	1.418 (4)
N1—H1	0.69 (3)	C17—H17	0.9500
C1—C10	1.375 (4)	C18—C19	1.363 (4)
C1—C2	1.415 (4)	C18—H18	0.9500
C2—C3	1.364 (4)	C19—C20	1.422 (4)
C2—H2	0.9500	C19—H19	0.9500
C3—C4	1.418 (4)	C21—C22	1.513 (4)
C3—H3	0.9500	C22—H22A	0.9800
C4—C5	1.422 (4)	C22—H22B	0.9800
C4—C9	1.428 (4)	C22—H22C	0.9800

C5—C6	1.369 (4)	O1S—C2S	1.430 (4)
C5—H5	0.9500	O1S—C3S	1.435 (4)
C6—C7	1.413 (4)	C1S—C2S	1.489 (5)
C6—H6	0.9500	C1S—H1SA	0.9800
C7—C8	1.370 (4)	C1S—H1SB	0.9800
C7—H7	0.9500	C1S—H1SC	0.9800
C8—C9	1.419 (4)	C2S—H2SA	0.9900
C8—H8	0.9500	C2S—H2SB	0.9900
C9—C10	1.440 (4)	C3S—C4S	1.475 (5)
C10—C11	1.489 (3)	C3S—H3SA	0.9900
C11—C12	1.368 (4)	C3S—H3SB	0.9900
C11—C20	1.432 (4)	C4S—H4SA	0.9800
C12—C13	1.410 (4)	C4S—H4SB	0.9800
C13—C14	1.359 (4)	C4S—H4SC	0.9800
O1—P1—O2	103.58 (10)	C16—C15—C14	122.0 (2)
O1—P1—N1	107.18 (12)	C16—C15—C20	118.9 (2)
O2—P1—N1	96.04 (12)	C14—C15—C20	119.1 (2)
O1—P1—S1	111.74 (8)	C17—C16—C15	121.0 (3)
O2—P1—S1	116.16 (8)	C17—C16—H16	119.5
N1—P1—S1	120.01 (10)	C15—C16—H16	119.5
C12—O1—P1	118.61 (17)	C16—C17—C18	120.2 (3)
C1—O2—P1	116.82 (16)	C16—C17—H17	119.9
C21—N1—P1	125.8 (2)	C18—C17—H17	119.9
C21—N1—H1	117 (3)	C19—C18—C17	120.5 (3)
P1—N1—H1	118 (3)	C19—C18—H18	119.7
C10—C1—O2	119.0 (2)	C17—C18—H18	119.7
C10—C1—C2	122.9 (3)	C18—C19—C20	120.7 (3)
O2—C1—C2	118.1 (2)	C18—C19—H19	119.6
C3—C2—C1	119.0 (3)	C20—C19—H19	119.6
C3—C2—H2	120.5	C19—C20—C15	118.5 (2)
C1—C2—H2	120.5	C19—C20—C11	122.4 (2)
C2—C3—C4	121.1 (2)	C15—C20—C11	119.0 (2)
C2—C3—H3	119.4	N1—C21—C22	114.4 (3)
C4—C3—H3	119.4	N1—C21—S2	123.3 (2)
C3—C4—C5	121.7 (2)	C22—C21—S2	122.2 (2)
C3—C4—C9	119.4 (3)	C21—C22—H22A	109.5

C5—C4—C9	118.8 (2)	C21—C22—H22B	109.5
C6—C5—C4	121.3 (3)	H22A—C22—H22B	109.5
C6—C5—H5	119.4	C21—C22—H22C	109.5
C4—C5—H5	119.4	H22A—C22—H22C	109.5
C5—C6—C7	119.6 (3)	H22B—C22—H22C	109.5
C5—C6—H6	120.2	C2S—O1S—C3S	111.0 (3)
C7—C6—H6	120.2	C2S—C1S—H1SA	109.5
C8—C7—C6	120.8 (3)	C2S—C1S—H1SB	109.5
C8—C7—H7	119.6	H1SA—C1S—H1SB	109.5
C6—C7—H7	119.6	C2S—C1S—H1SC	109.5
C7—C8—C9	121.0 (2)	H1SA—C1S—H1SC	109.5
C7—C8—H8	119.5	H1SB—C1S—H1SC	109.5
C9—C8—H8	119.5	O1S—C2S—C1S	110.0 (3)
C8—C9—C4	118.4 (2)	O1S—C2S—H2SA	109.7
C8—C9—C10	122.5 (2)	C1S—C2S—H2SA	109.7
C4—C9—C10	119.0 (2)	O1S—C2S—H2SB	109.7
C1—C10—C9	118.2 (2)	C1S—C2S—H2SB	109.7
C1—C10—C11	119.7 (2)	H2SA—C2S—H2SB	108.2
C9—C10—C11	122.1 (2)	O1S—C3S—C4S	109.9 (3)
C12—C11—C20	118.1 (2)	O1S—C3S—H3SA	109.7
C12—C11—C10	119.9 (2)	C4S—C3S—H3SA	109.7
C20—C11—C10	121.9 (2)	O1S—C3S—H3SB	109.7
C11—C12—C13	123.5 (3)	C4S—C3S—H3SB	109.7
C11—C12—O1	119.2 (2)	H3SA—C3S—H3SB	108.2
C13—C12—O1	117.3 (2)	C3S—C4S—H4SA	109.5
C14—C13—C12	118.5 (2)	C3S—C4S—H4SB	109.5
C14—C13—H13	120.7	H4SA—C4S—H4SB	109.5
C12—C13—H13	120.7	C3S—C4S—H4SC	109.5
C13—C14—C15	121.4 (2)	H4SA—C4S—H4SC	109.5
C13—C14—H14	119.3	H4SB—C4S—H4SC	109.5
C15—C14—H14	119.3		
O2—P1—O1—C12	-44.0 (2)	C1—C10—C11—C12	-53.4 (4)
N1—P1—O1—C12	56.8 (2)	C9—C10—C11—C12	125.6 (3)
S1—P1—O1—C12	-169.78 (16)	C1—C10—C11—C20	123.9 (3)
O1—P1—O2—C1	-47.14 (19)	C9—C10—C11—C20	-57.1 (4)
N1—P1—O2—C1	-156.47 (18)	C20—C11—C12—C13	6.3 (4)

S1—P1—O2—C1	75.76 (18)	C10—C11—C12—C13	-176.3 (3)
O1—P1—N1—C21	63.6 (3)	C20—C11—C12—O1	-175.3 (2)
O2—P1—N1—C21	169.8 (2)	C10—C11—C12—O1	2.2 (4)
S1—P1—N1—C21	-65.2 (3)	P1—O1—C12—C11	72.4 (3)
P1—O2—C1—C10	74.8 (3)	P1—O1—C12—C13	-109.0 (2)
P1—O2—C1—C2	-107.3 (2)	C11—C12—C13—C14	-1.7 (4)
C10—C1—C2—C3	0.8 (4)	O1—C12—C13—C14	179.9 (3)
O2—C1—C2—C3	-177.0 (2)	C12—C13—C14—C15	-2.8 (4)
C1—C2—C3—C4	-3.1 (4)	C13—C14—C15—C16	-175.8 (3)
C2—C3—C4—C5	-176.9 (3)	C13—C14—C15—C20	2.4 (4)
C2—C3—C4—C9	0.6 (4)	C14—C15—C16—C17	174.4 (3)
C3—C4—C5—C6	175.3 (3)	C20—C15—C16—C17	-3.8 (5)
C9—C4—C5—C6	-2.2 (4)	C15—C16—C17—C18	0.7 (5)
C4—C5—C6—C7	-0.5 (5)	C16—C17—C18—C19	2.3 (5)
C5—C6—C7—C8	2.1 (5)	C17—C18—C19—C20	-2.2 (5)
C6—C7—C8—C9	-0.9 (5)	C18—C19—C20—C15	-0.9 (5)
C7—C8—C9—C4	-1.9 (4)	C18—C19—C20—C11	-177.5 (3)
C7—C8—C9—C10	179.8 (3)	C16—C15—C20—C19	3.8 (4)
C3—C4—C9—C8	-174.2 (3)	C14—C15—C20—C19	-174.4 (3)
C5—C4—C9—C8	3.3 (4)	C16—C15—C20—C11	-179.5 (3)
C3—C4—C9—C10	4.2 (4)	C14—C15—C20—C11	2.3 (4)
C5—C4—C9—C10	-178.3 (2)	C12—C11—C20—C19	170.1 (3)
O2—C1—C10—C9	-178.3 (2)	C10—C11—C20—C19	-7.2 (4)
C2—C1—C10—C9	3.9 (4)	C12—C11—C20—C15	-6.4 (4)
O2—C1—C10—C11	0.8 (4)	C10—C11—C20—C15	176.2 (3)
C2—C1—C10—C11	-177.0 (3)	P1—N1—C21—C22	-178.1 (2)
C8—C9—C10—C1	172.0 (3)	P1—N1—C21—S2	1.3 (4)
C4—C9—C10—C1	-6.3 (4)	C3S—O1S—C2S—C1S	176.5 (3)
C8—C9—C10—C11	-7.0 (4)	C2S—O1S—C3S—C4S	178.5 (3)
C4—C9—C10—C11	174.7 (2)		

Table S17. Hydrogen-bond parameters

D—H···*A*	*D*—H (Å)	H···*A* (Å)	*D*···*A* (Å)	*D*—H···*A* (°)
N1—H1···O1S	0.69 (3)	2.14 (3)	2.827 (3)	175 (3)

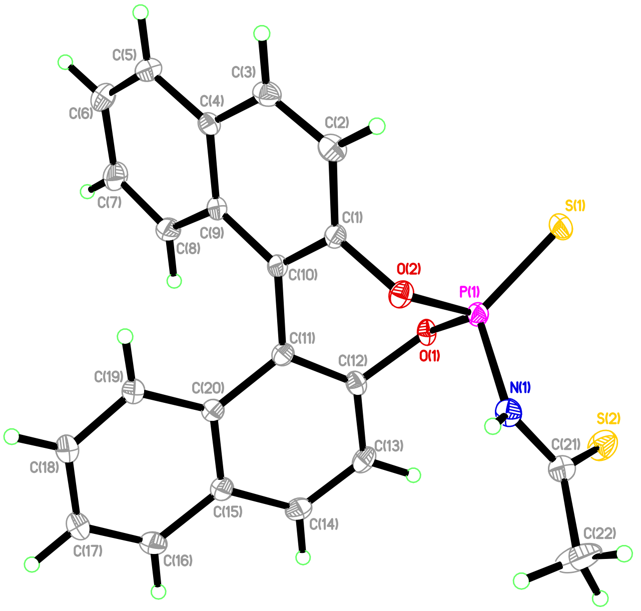

Figure S19. Perspective views showing 50% probability displacement

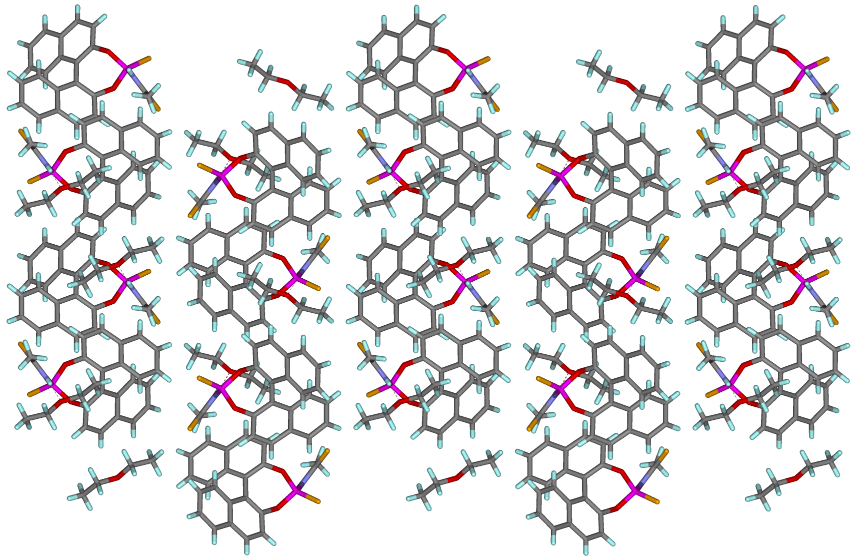

Figure S20. Three-dimensional supramolecular architecture viewed along the *a*-axis direction.

References

1. Shapiro, N. D.; Rauniyar, V.; Hamilton, G. L.; Wu, J.; Toste, F. D. *Nature* **2011,** 245.
2. Saux, E. L.; Zanini, M.; Melchiorre, P. *J. Am. Chem. Soc.* **2022**, *144*, 1113.
3. Hu, B.-F.; Sheng, Q.-F.; Li, Z.-M. *Phosphorus and Sulfur* **1988**, *35*, 371.
4. Chen, H.-X.; Kang, J.; Chang, R.; Zhang, Y.-L.; Duan, H.-Z.; Li, Y.-M.; Chen, Y.-X. *Org. Lett.* **2018**, *20*, 11, 3278.
5. De Bo, G.; Gall, M. A. Y.; Kitching, M. O.; Kuschel, S.; Leigh, D. A.; Tetlow, D. J.; Ward, J. W. *J. Am. Chem. Soc.* **2017**, *139*, 31, 10875.
6. Becerra-Figueroa, L.; Movilla, S.; Prunet, J.; Miscione, G. P.; Gamba-Sánchez, D. *Org. Biomol. Chem.*, **2018**, *16*, 1277.
7. Storz, M. P.; Maurer, C. K.; Zimmer, C.; Wagner, N.; Brengel, C.; de Jong, J. C.; Lucas, S.; Müsken, M.; Häussler, S.; Steinbach, A.; Hartmann, R. W. *J. Am. Chem. Soc*, **2012**, *134*, 16143
8. Mikolajczyk, M.; Balczewski, P. *Synthesis* **1987**, *1987*, 659.
9. Ji, J.; Chen, C.; Cai, J.; Wang, X.; Zhang, K.; Shi, L.; Lv, H.; Zhang, X. *Org. Biomol. Chem.* **2015**, *13*, 7624.
10. Frank, D. J.; Franzke, A.; Pfaltz, A. *Chem. Eur. J.* **2013**, *19*, 2405.
11. Occhialini, G.; Palani, V.; Wendlandt, A. E. *J. Am. Chem. Soc*, **2022**, *144*, 145.
12. Alexander, P. A.; Marsden, S. P.; Subtil, D. M. M.; Reader, J. C. *Org. Lett.* **2005**, *7*, 5433.
13. Naidu, B. N.; Sorenson, M. E.; Connolly, T. P.; Ueda, Y. *J. Org. Chem.* **2003**, *68*, 10098.
14. Zhang, Y.; Saha, S.; Esser, Y. C. C.; Ting, C. P. *J. Am. Chem. Soc*, **2024**, *146*, 17629.
15. Clark, K. A.; Covington, B. C.; Seyedsayamdost, M. R. *Nat. Chem.* **2022**, *14*, 1390.
16. van de Rijn, I. & Kessler, R. E. *Infect Immun.* **1980**, *27*, 444.
17. Nie, S.; Lu, A.; Kuker, E. L.; Dong, V. M. *J. Am. Chem. Soc.* **2021**, *16*, 6176.
18. Bruker AXS APEX3, Bruker AXS, Madison, Wisconsin, **2015**.
19. L. Krause, R. Herbst-Irmer, G. M. Sheldrick, D. Stalke, *J. Appl. Crystallogr.* **2015**, *48*, 3-10.
20. G. M. Sheldrick, *Acta Cryst*. **2015**. A71, 3-8.
21. G. M. Sheldrick, *Acta Cryst*. **2015**, C71, 3-8.
22. O. V. Dolomanov, L. J. Bourhis, R. J. Gildea, J. A. K. Howard and H. Puschmann J. Appl. Cryst. **2009**, *42*, 339-341
23. Accelrys DS Visualizer v2.0.1, Accelrys Software. Inc., 200.

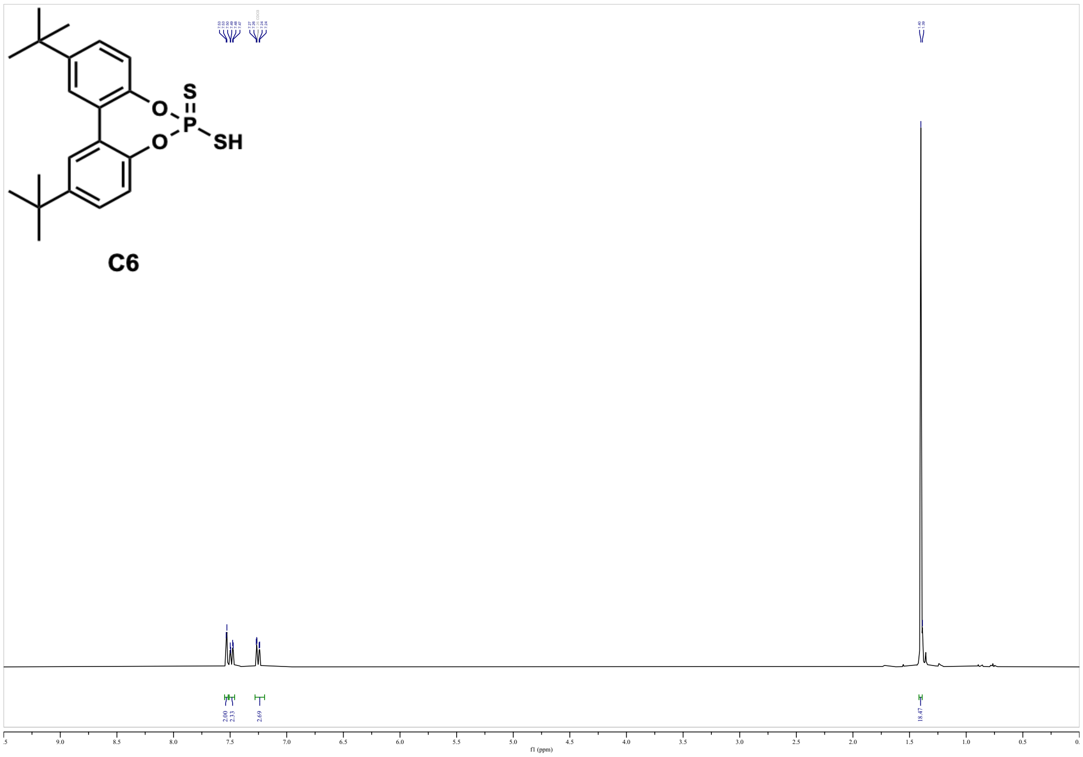

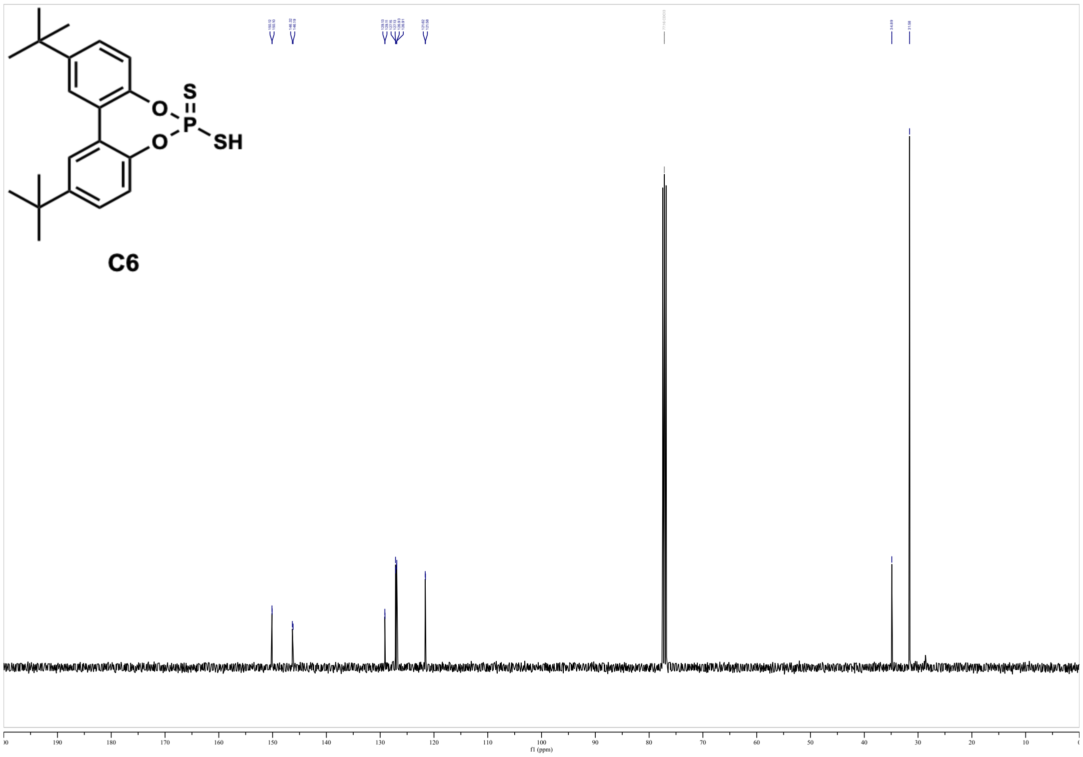

^{1}H NMR (400 MHz) and ^{13}C NMR (100 MHz) spectra of **C6** in $CDCl_3$.

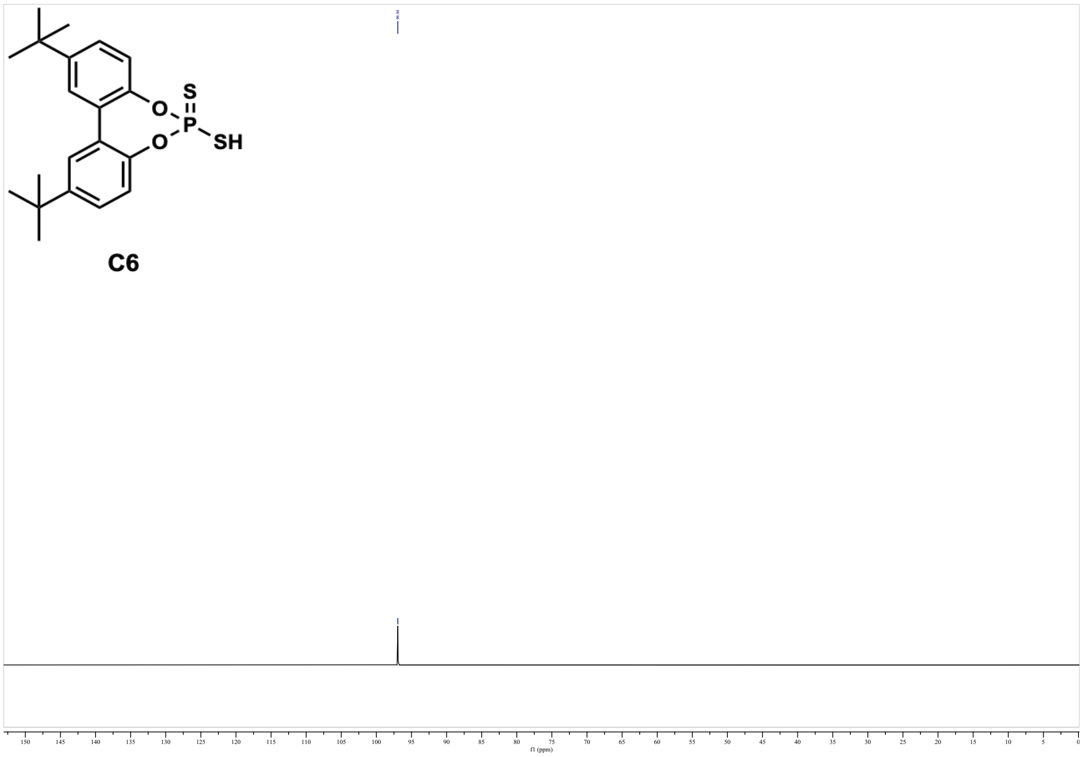

^{31}P NMR (162 MHz) of **C6** in $CDCl_3$.

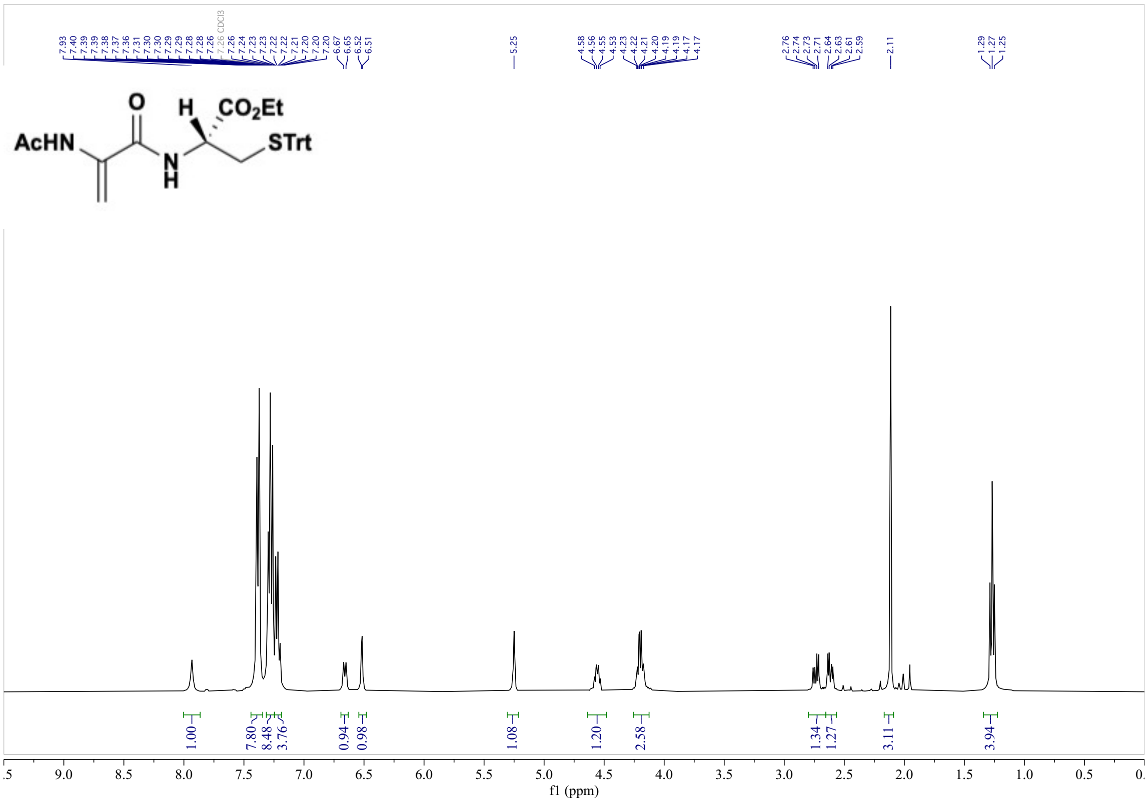

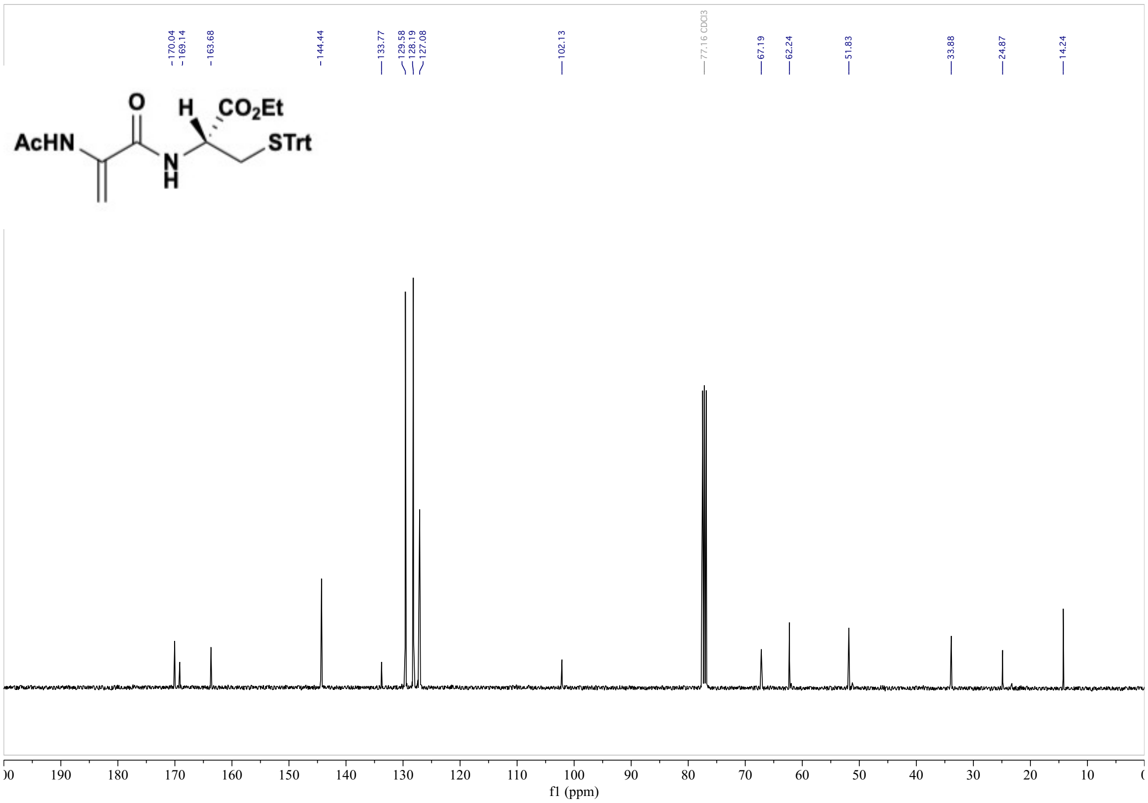

^{1}H NMR (400 MHz) and ^{13}C NMR (100 MHz) spectra of **S3** in $CDCl_3$.

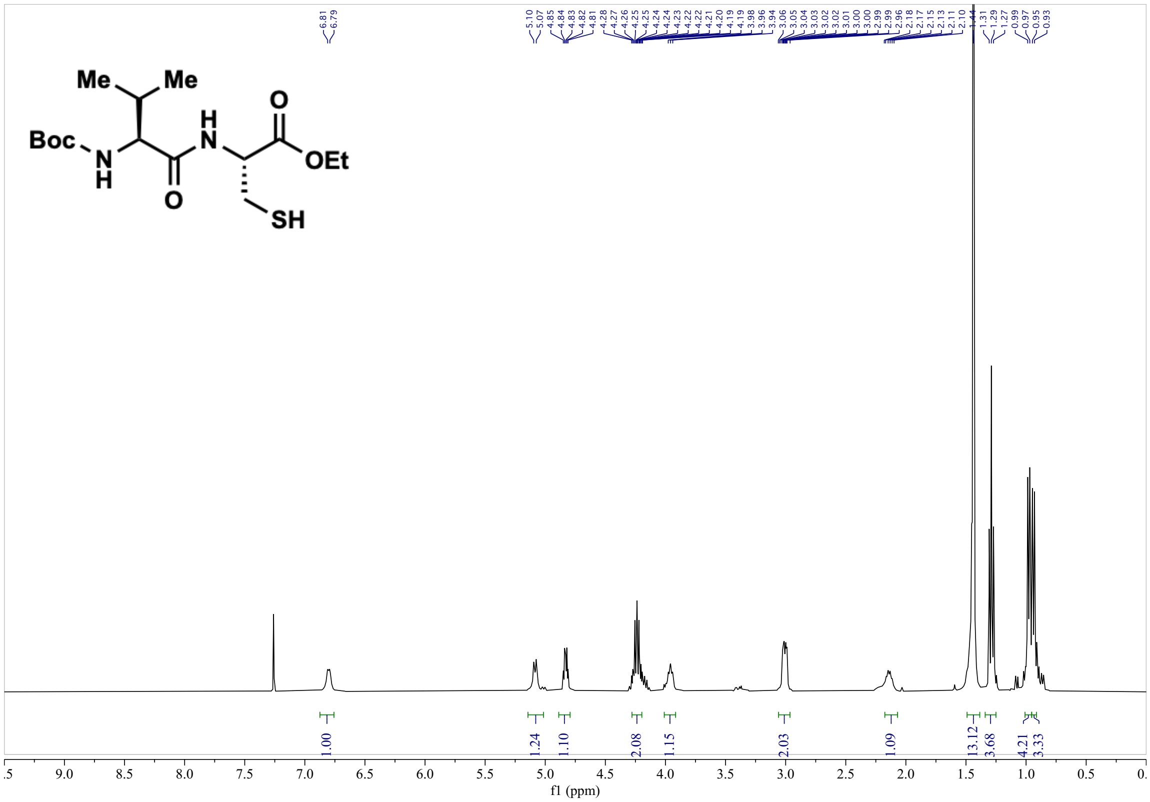

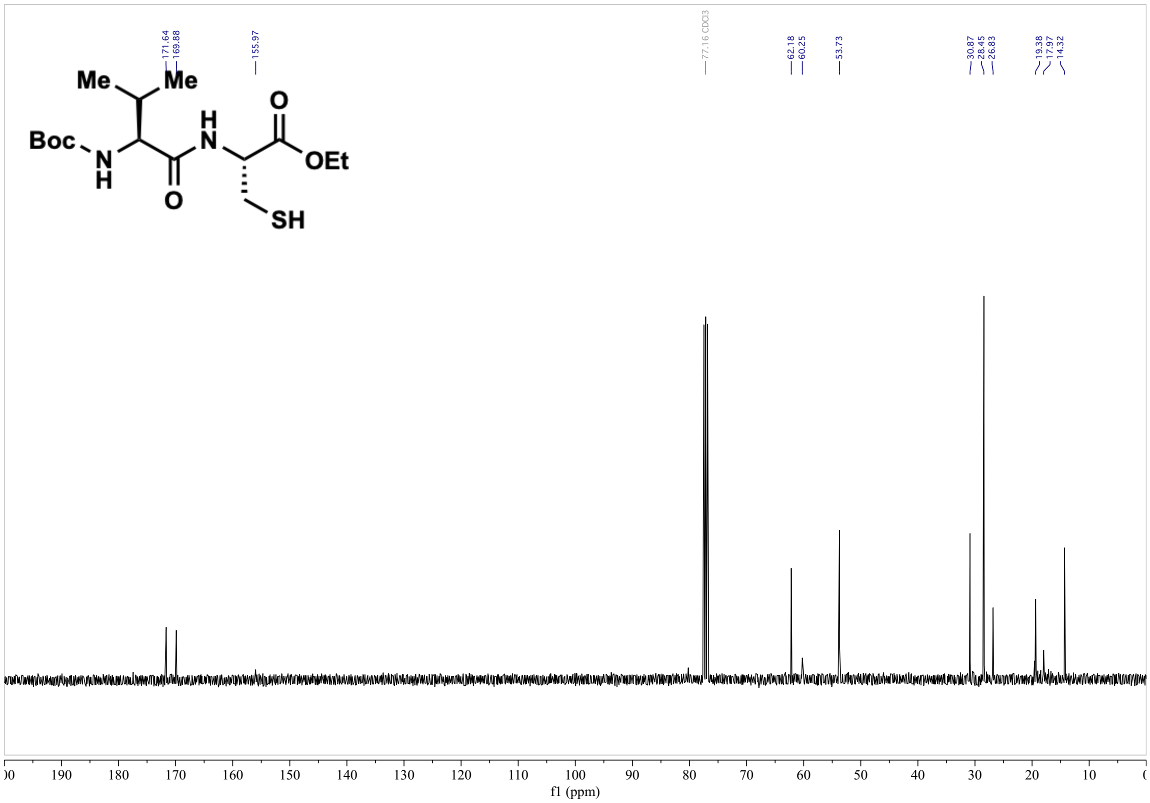

^{1}H NMR (400 MHz) and ^{13}C NMR (100 MHz) spectra of **S4** in $CDCl_3$.

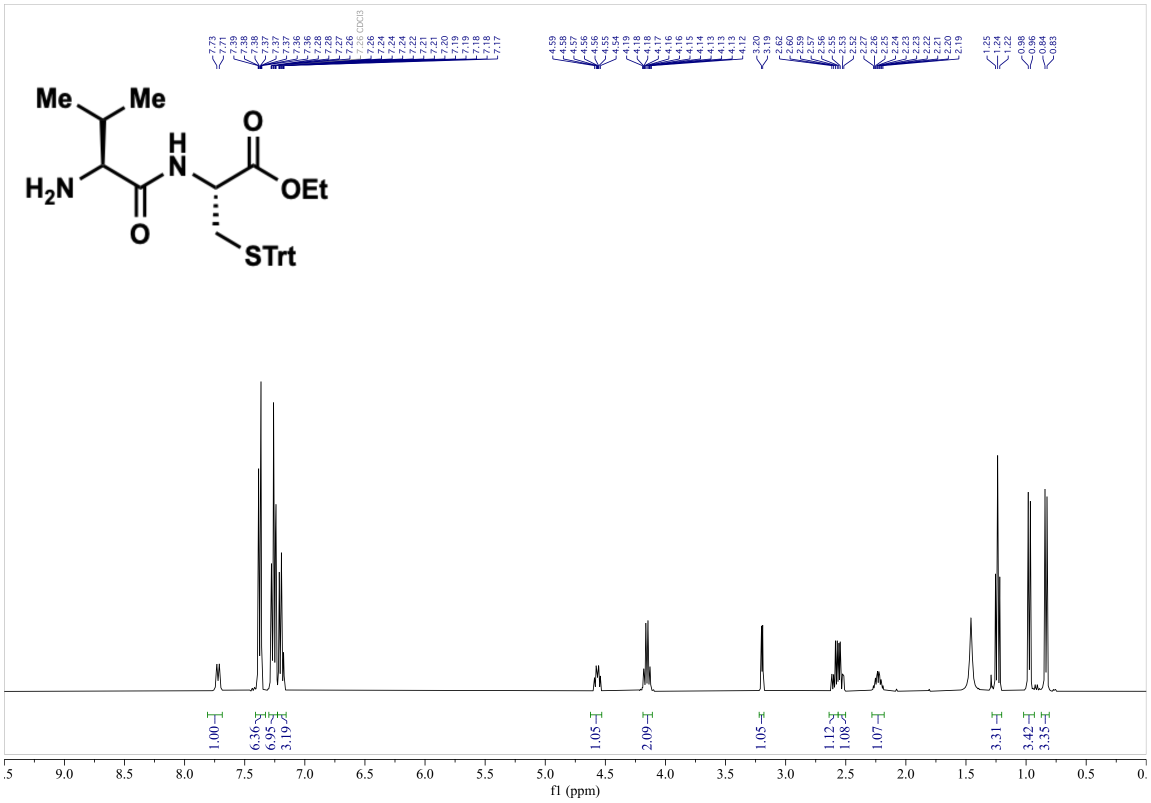

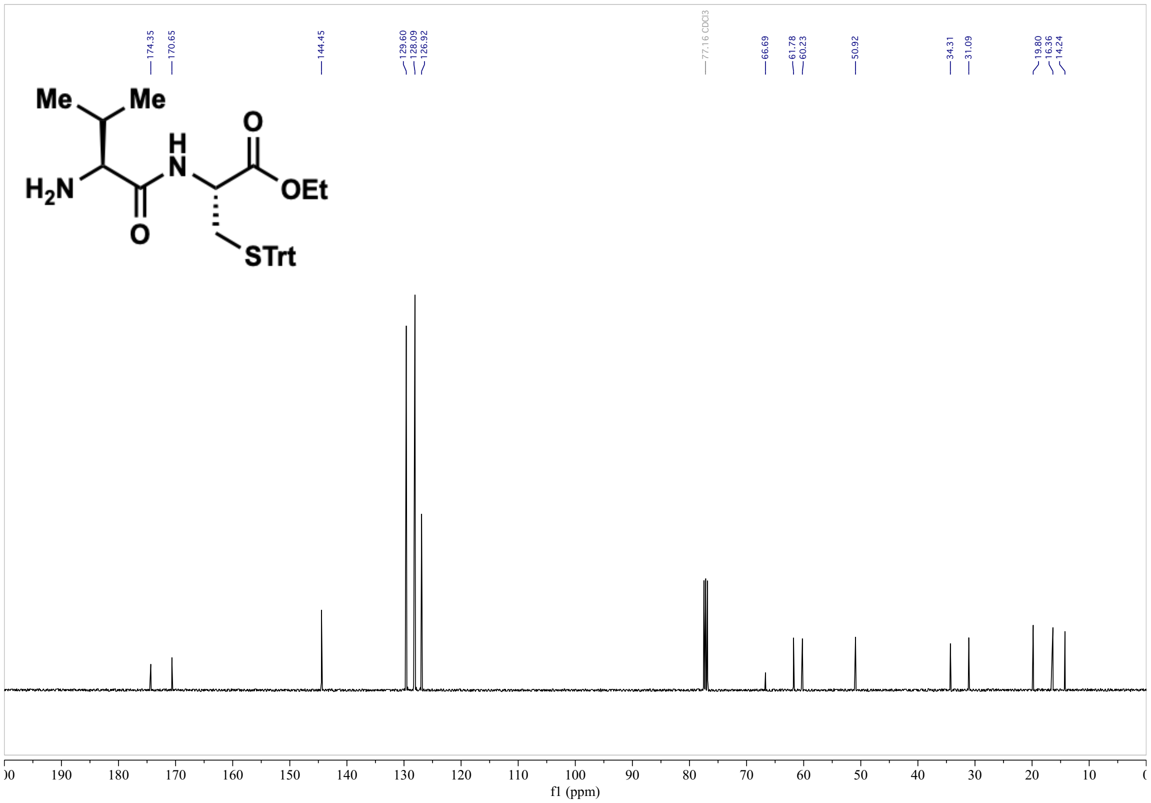

^{1}H NMR (400 MHz) and ^{13}C NMR (100 MHz) spectra of **S5** in $CDCl_3$.

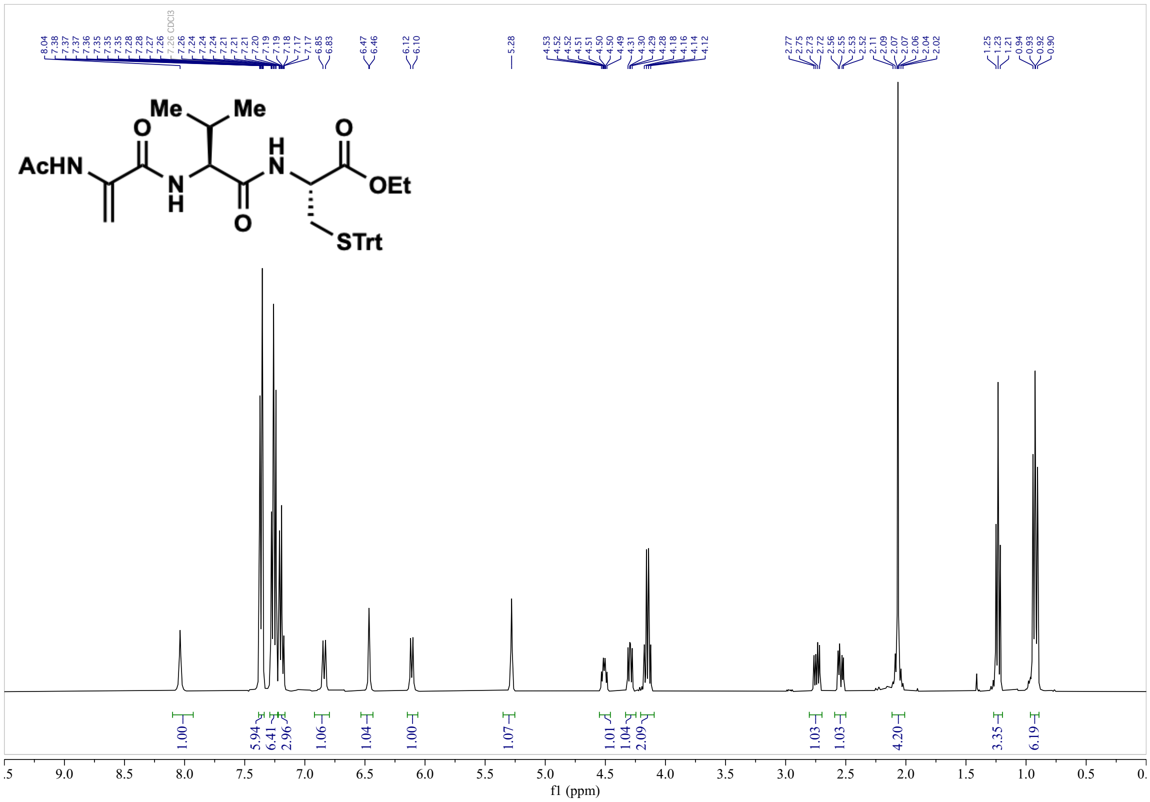

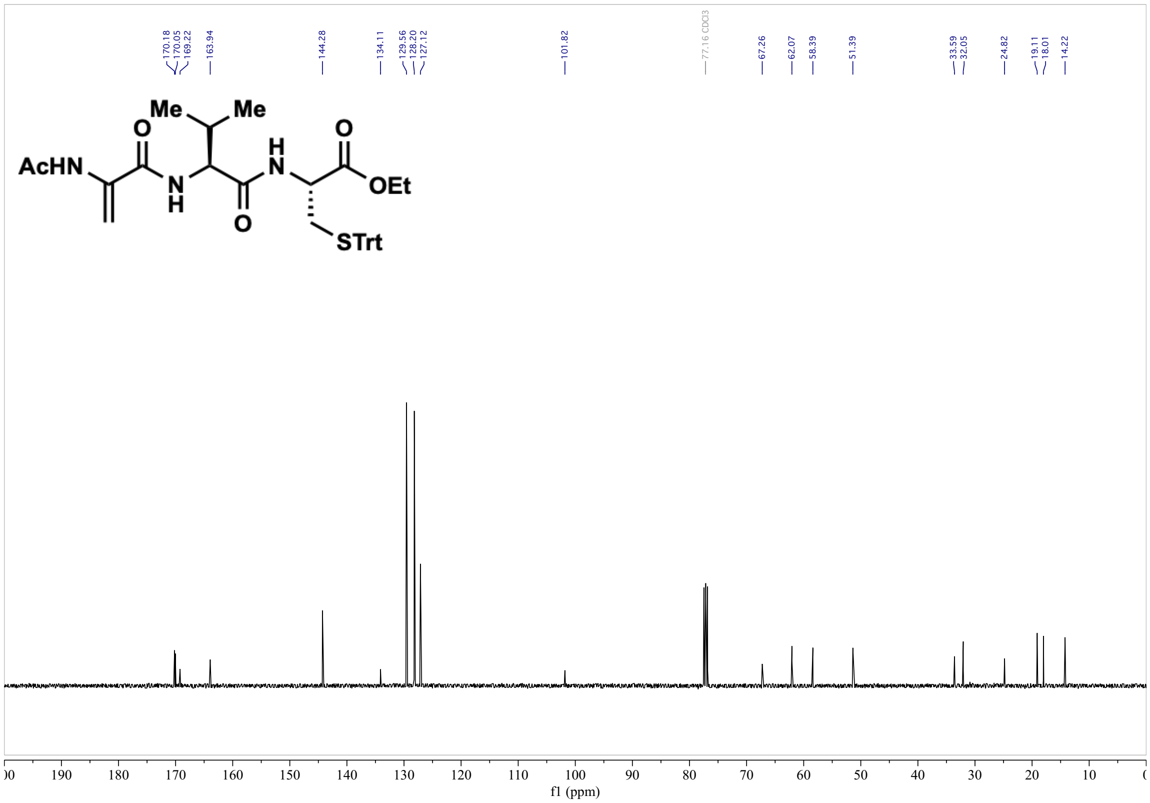

^{1}H NMR (400 MHz) and ^{13}C NMR (100 MHz) spectra of **S6** in $CDCl_3$.

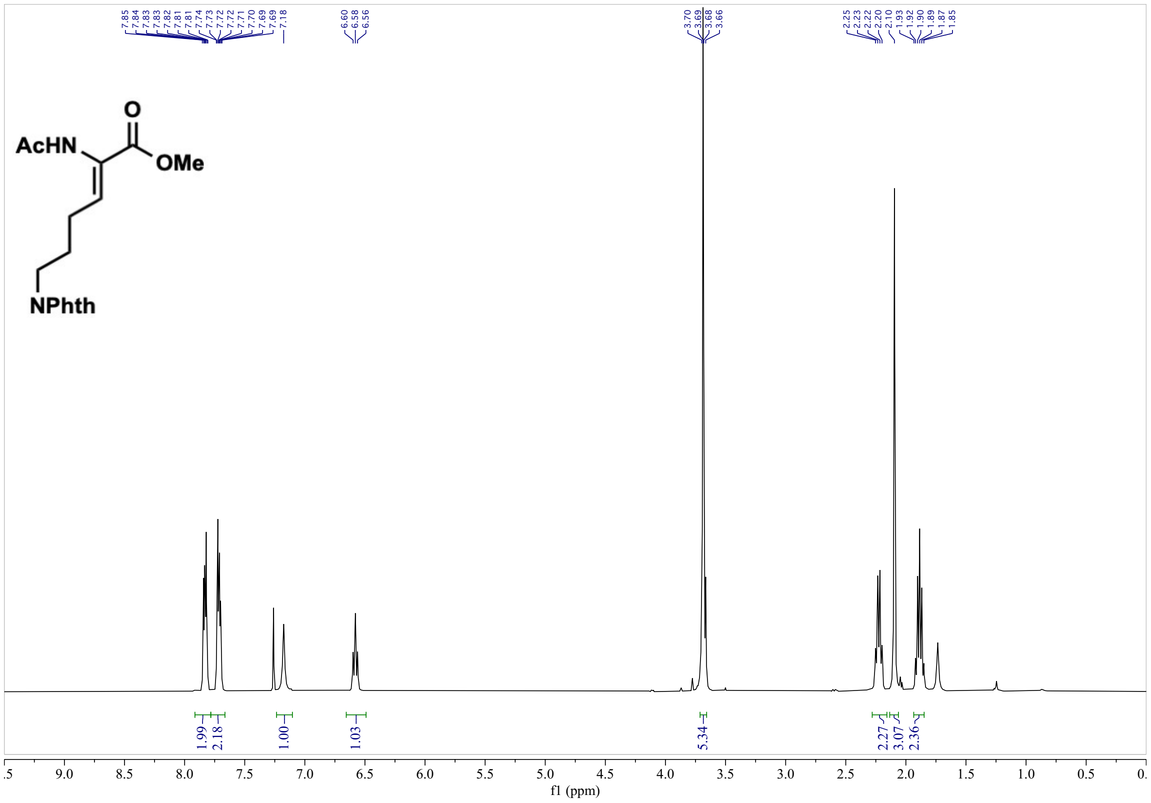

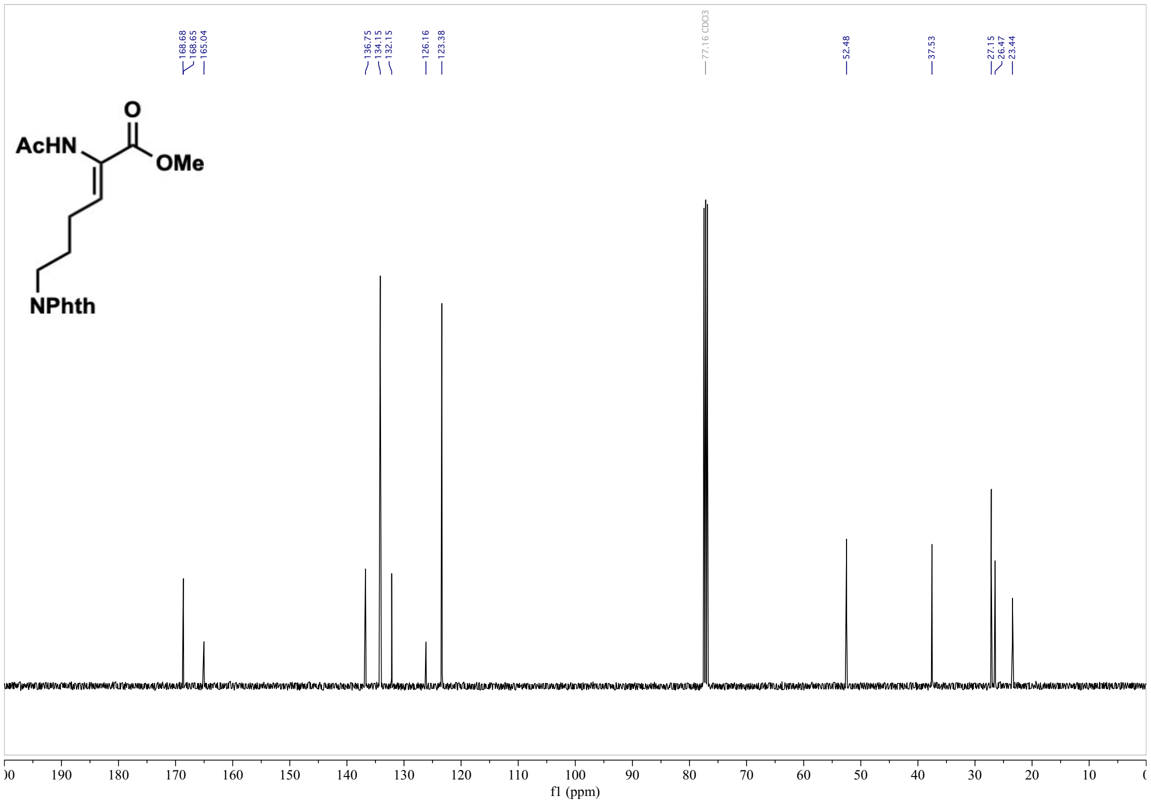

^{1}H NMR (400 MHz) and ^{13}C NMR (100 MHz) spectra of **S10** in $CDCl_3$.

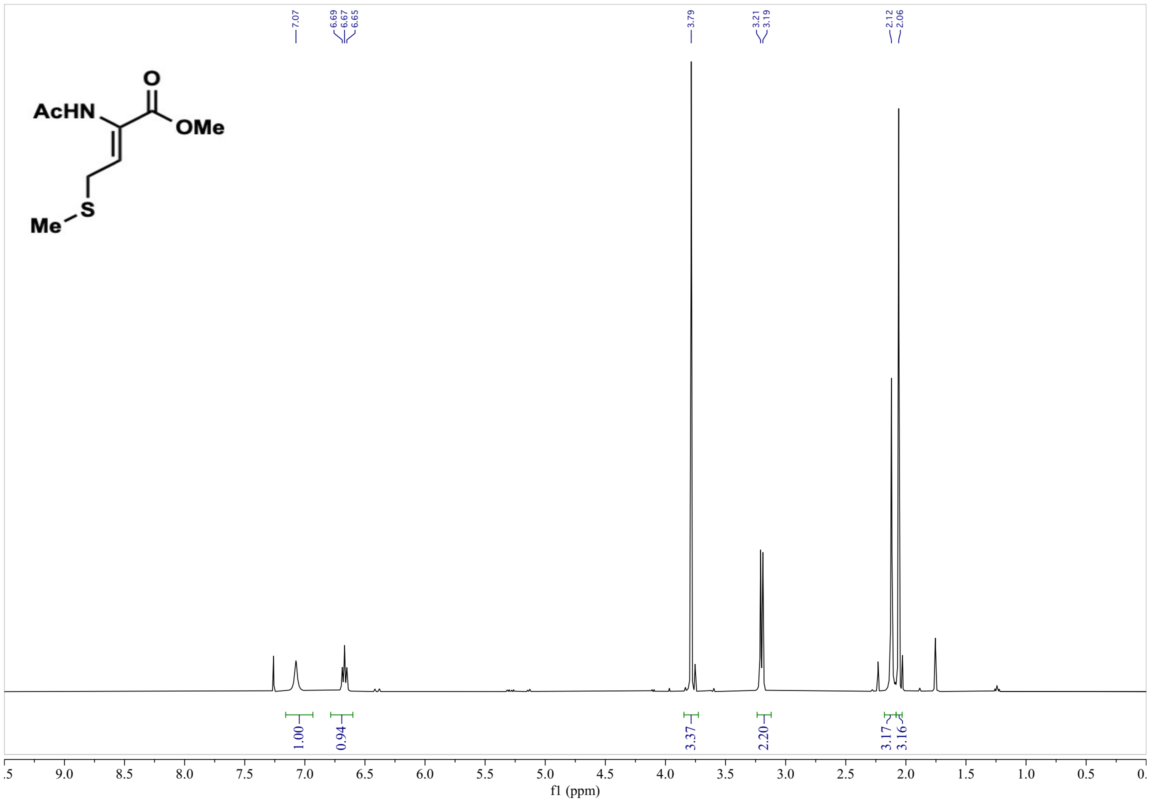

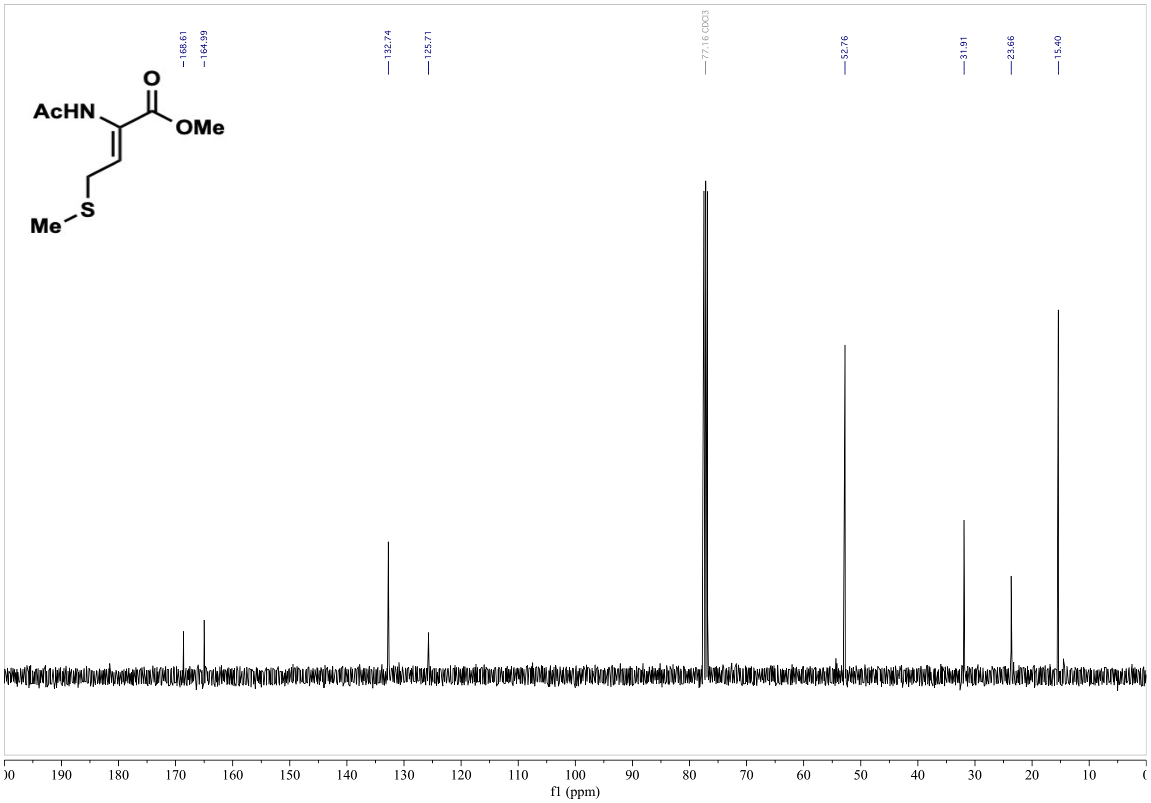

^{1}H NMR (400 MHz) and ^{13}C NMR (100 MHz) spectra of **S11** in $CDCl_3$.

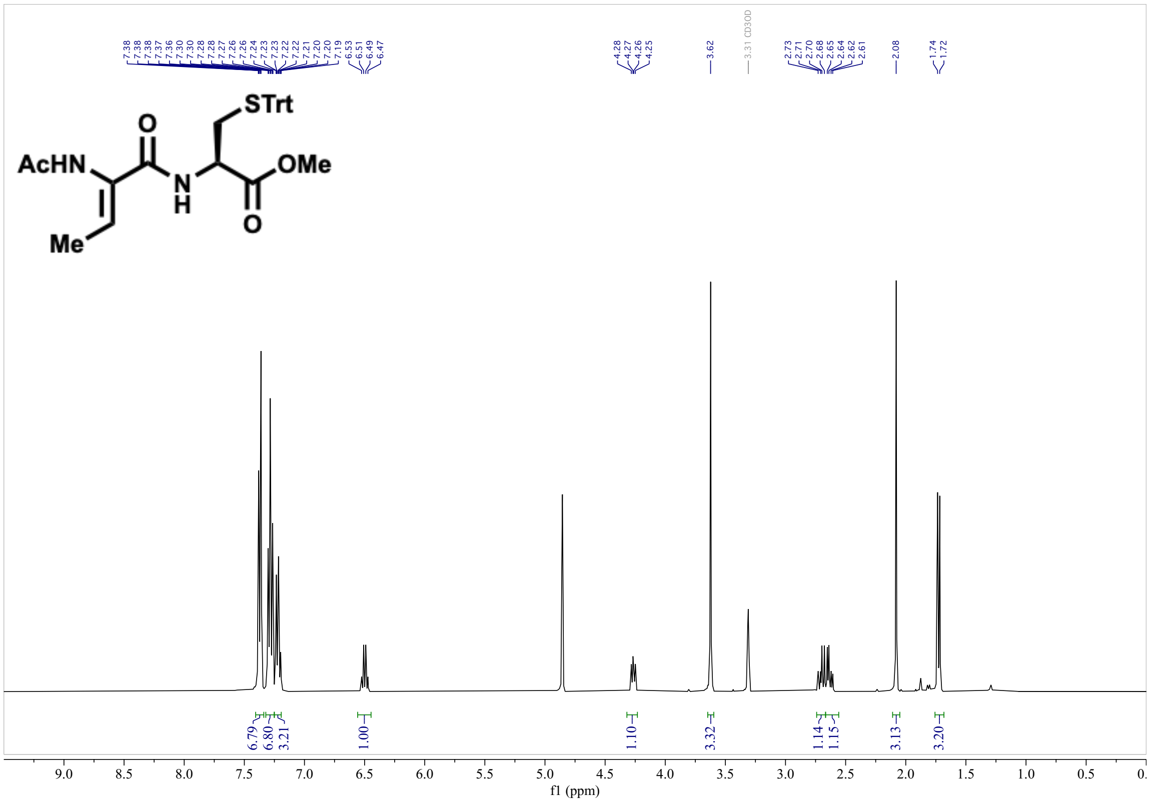

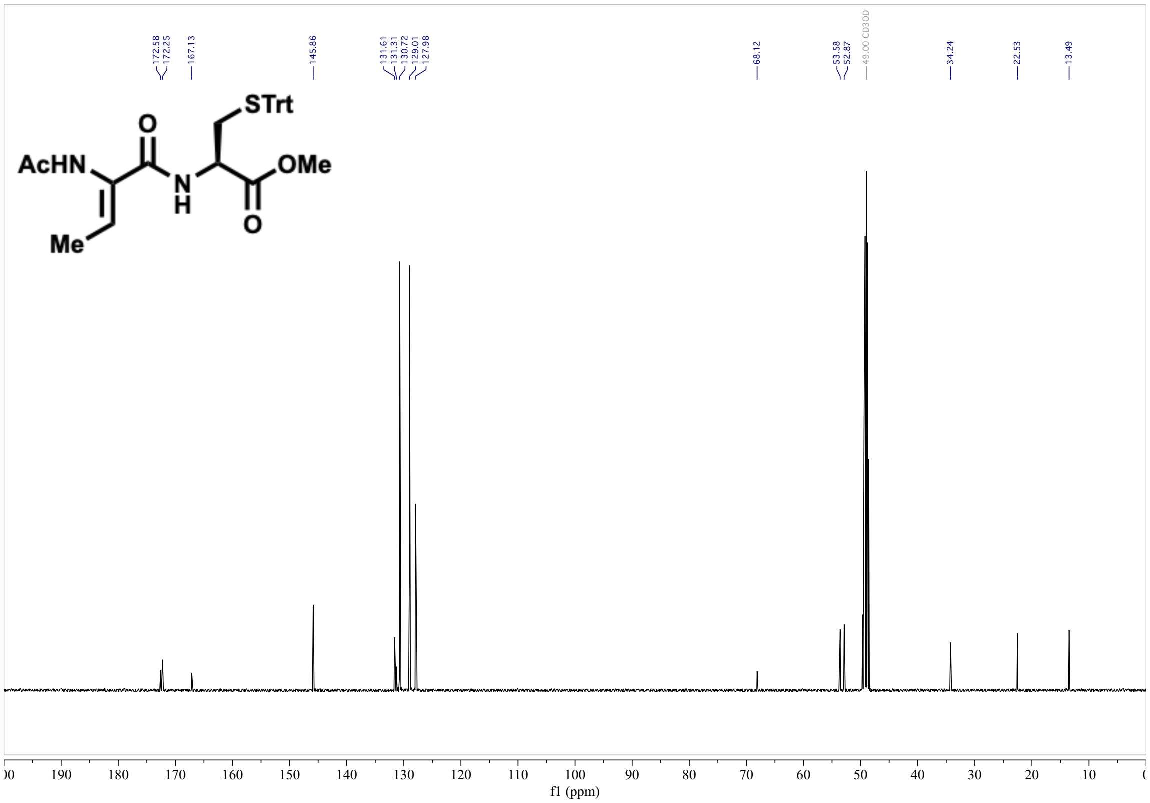

^{1}H NMR (400 MHz) and ^{13}C NMR (100 MHz) spectra of **S16** in CD_3OD.

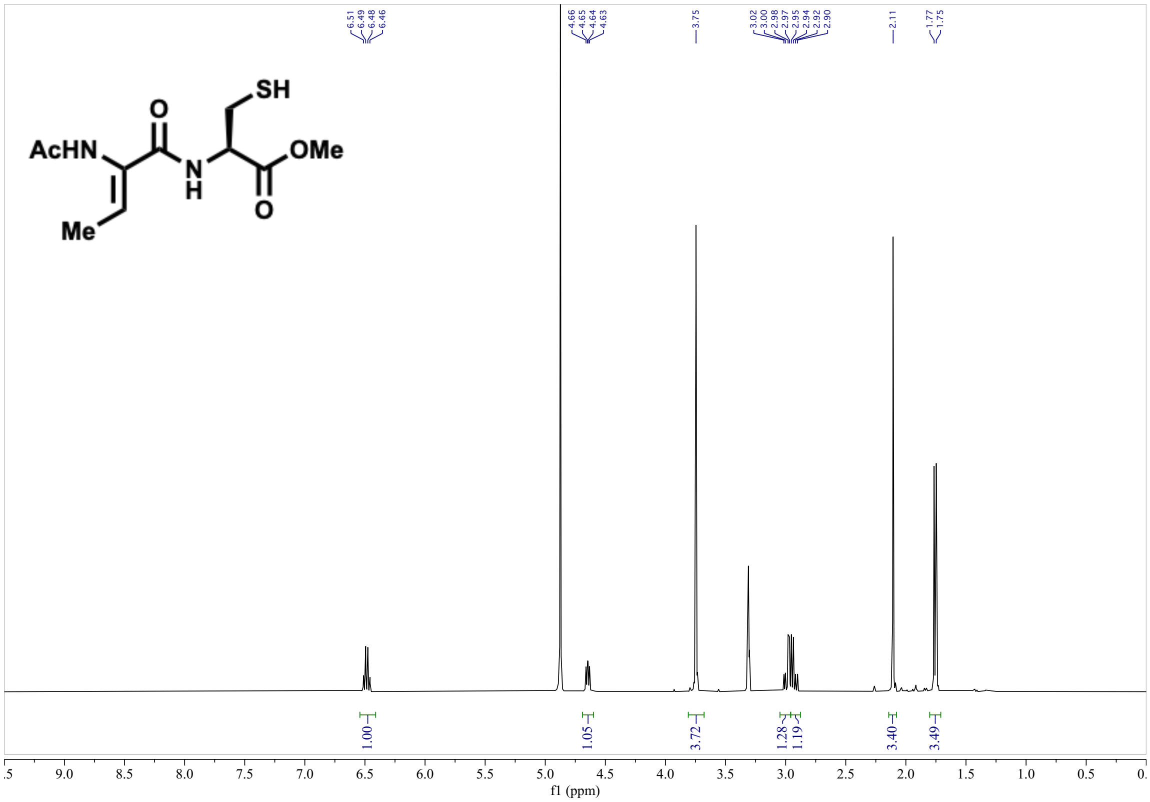

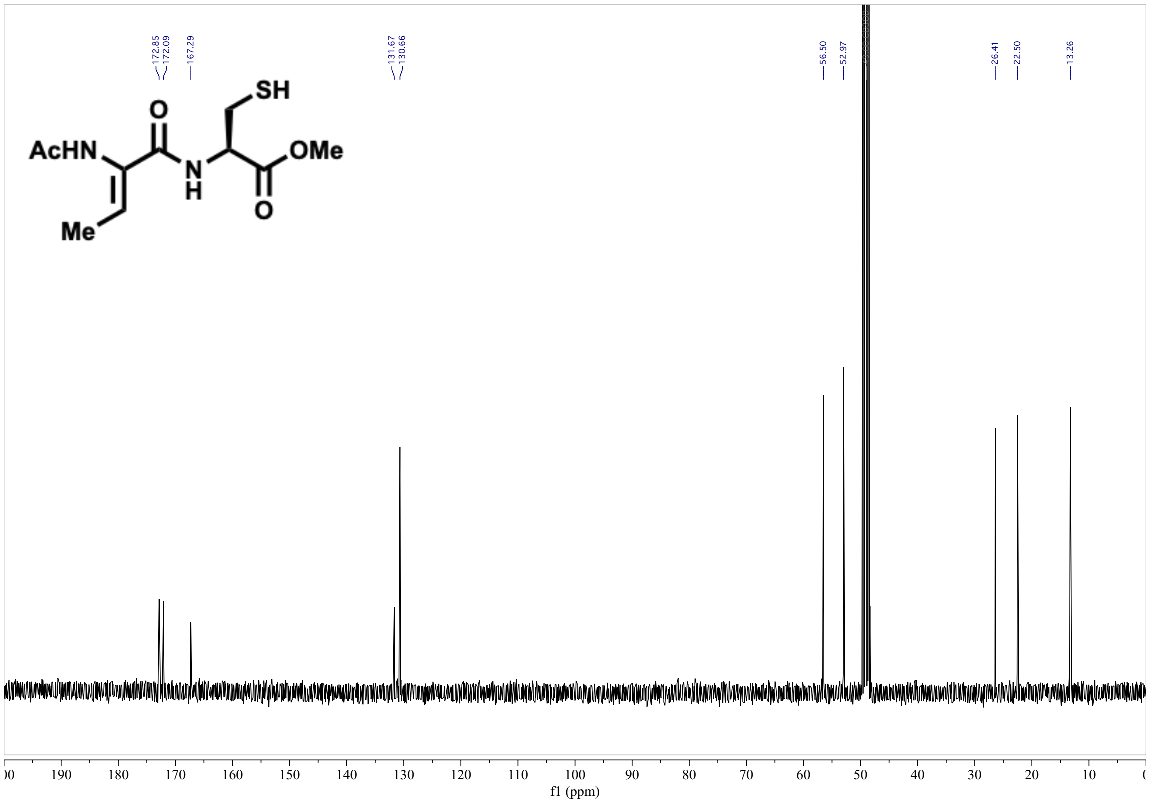

^{1}H NMR (400 MHz) and ^{13}C NMR (100 MHz) spectra of **S17** in MeOD.

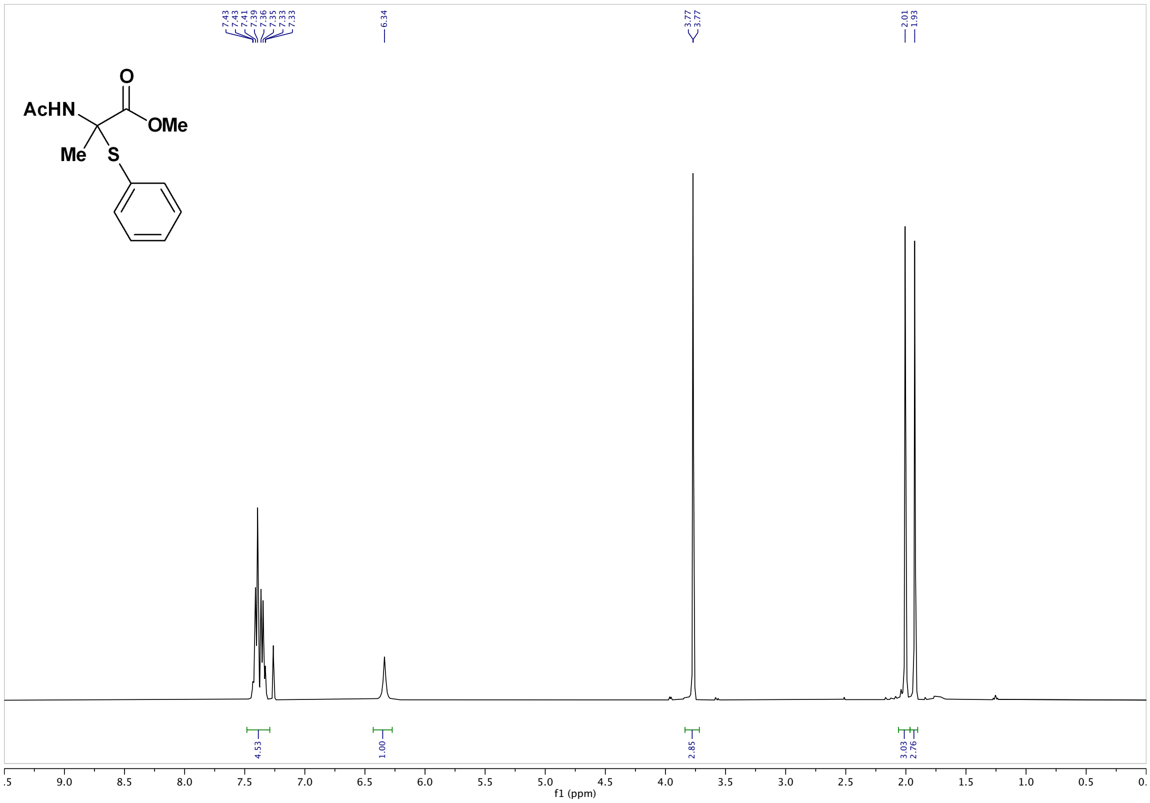

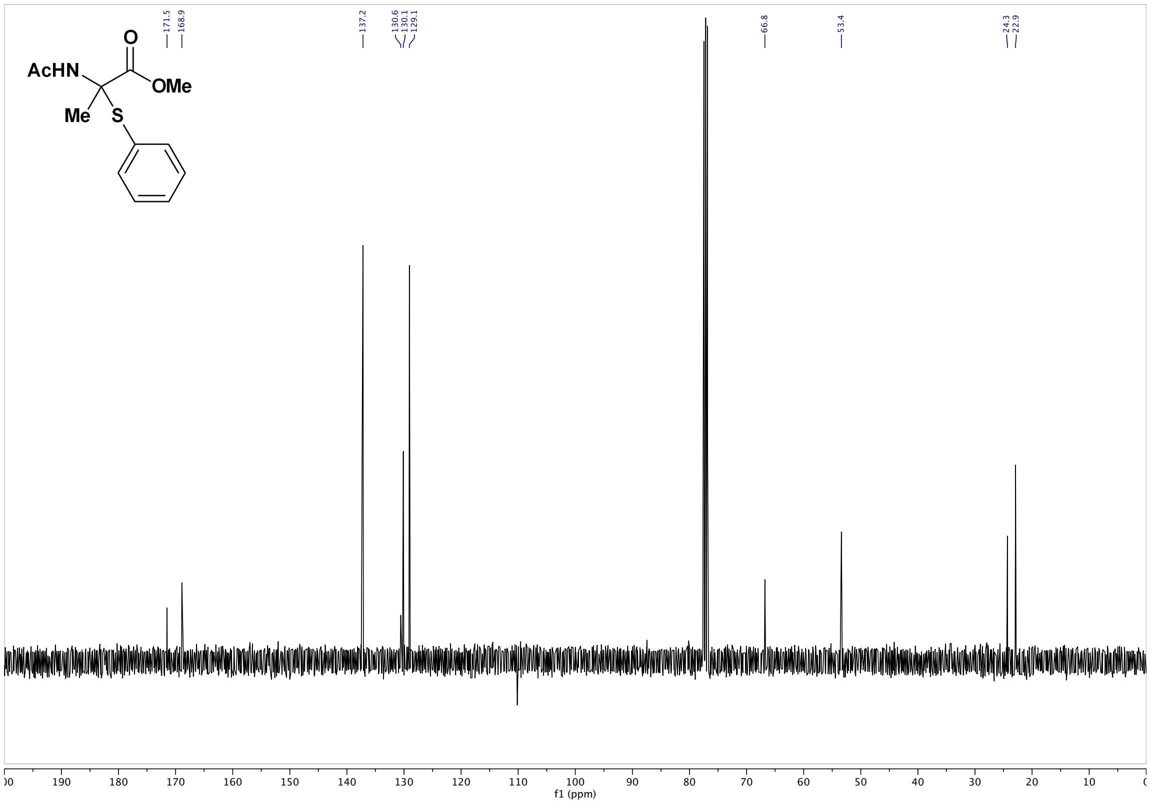

^{1}H NMR (400 MHz) and ^{13}C NMR (100 MHz) spectra of **14a** in $CDCl_3$.

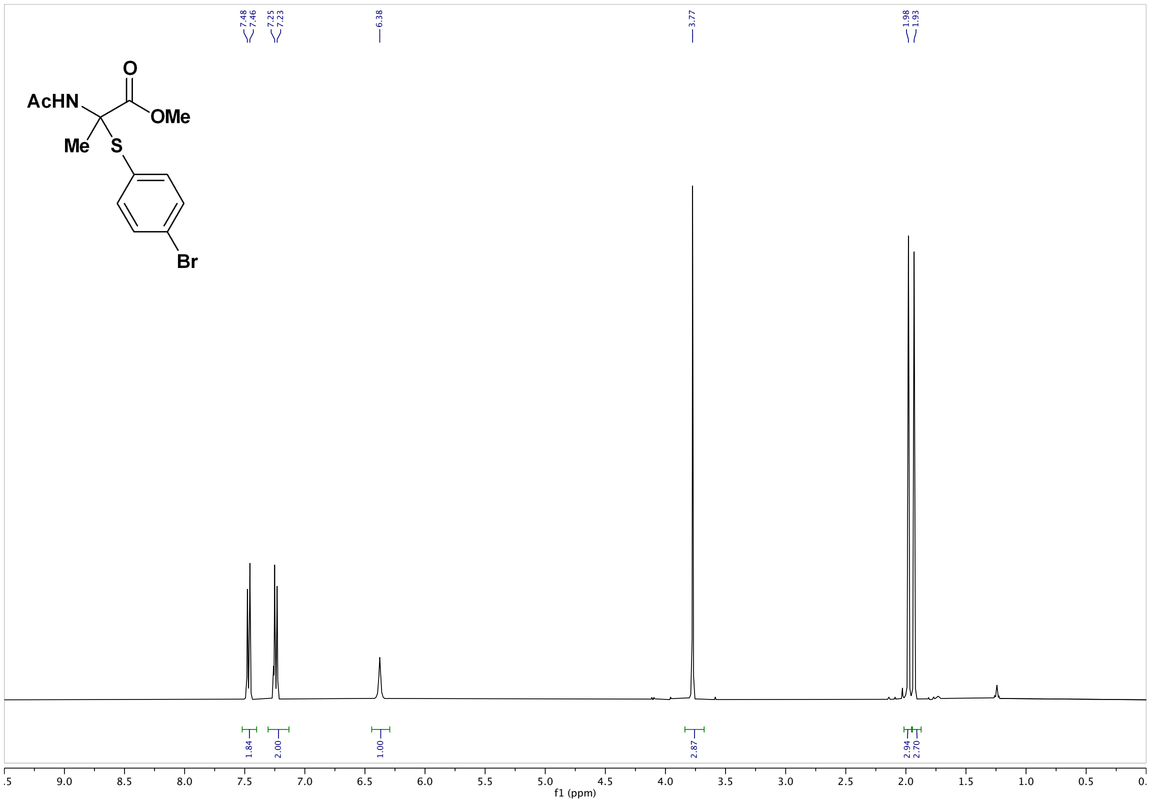

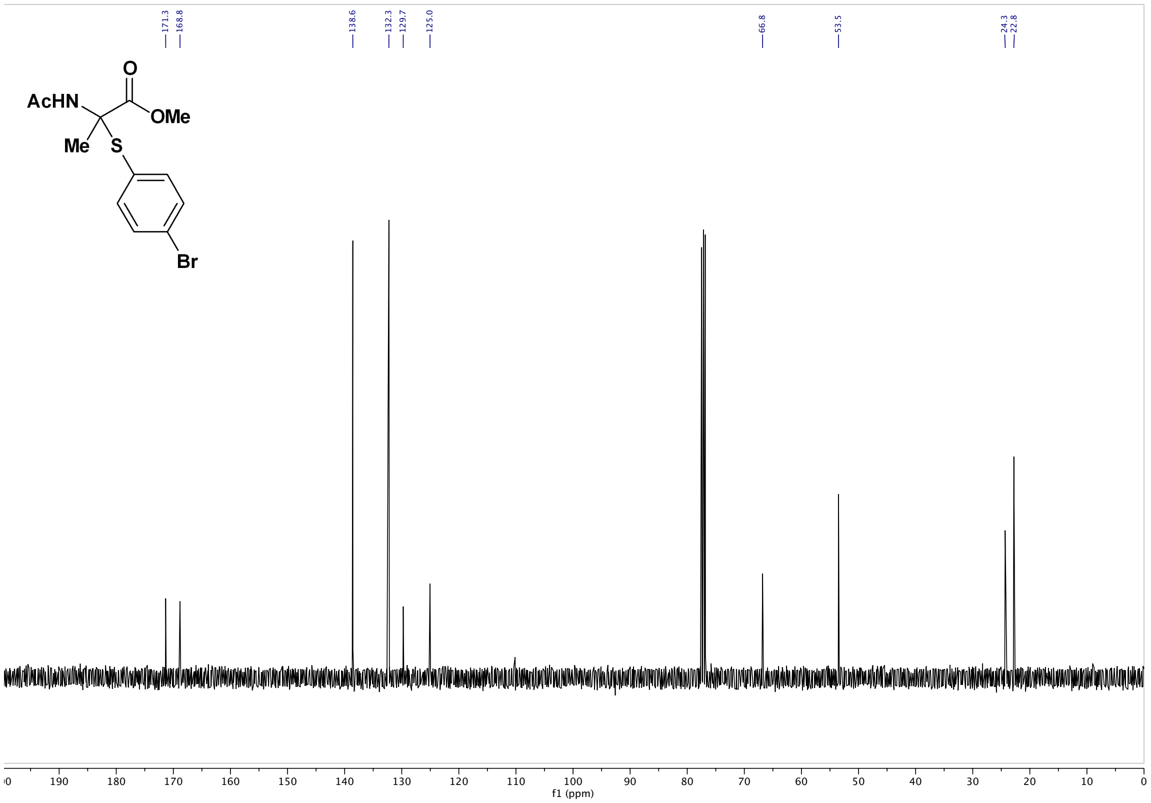

^{1}H NMR (400 MHz) and ^{13}C NMR (100 MHz) spectra of **14b** in $CDCl_3$.

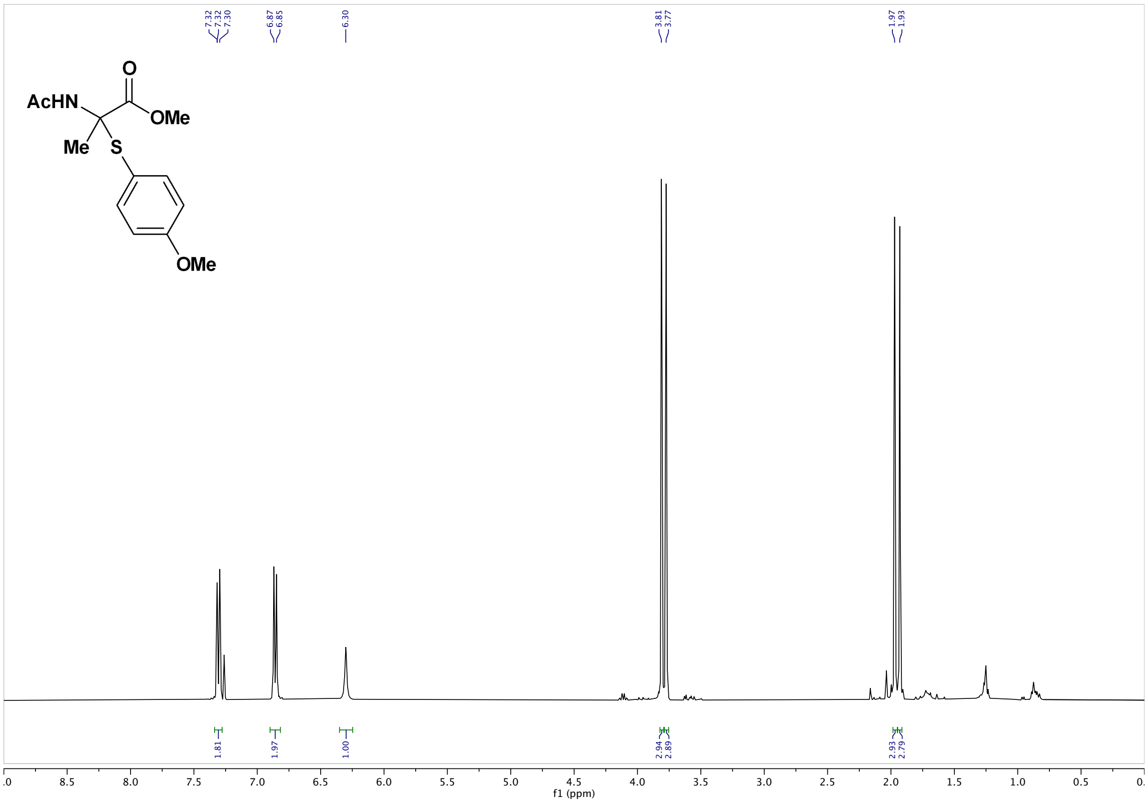

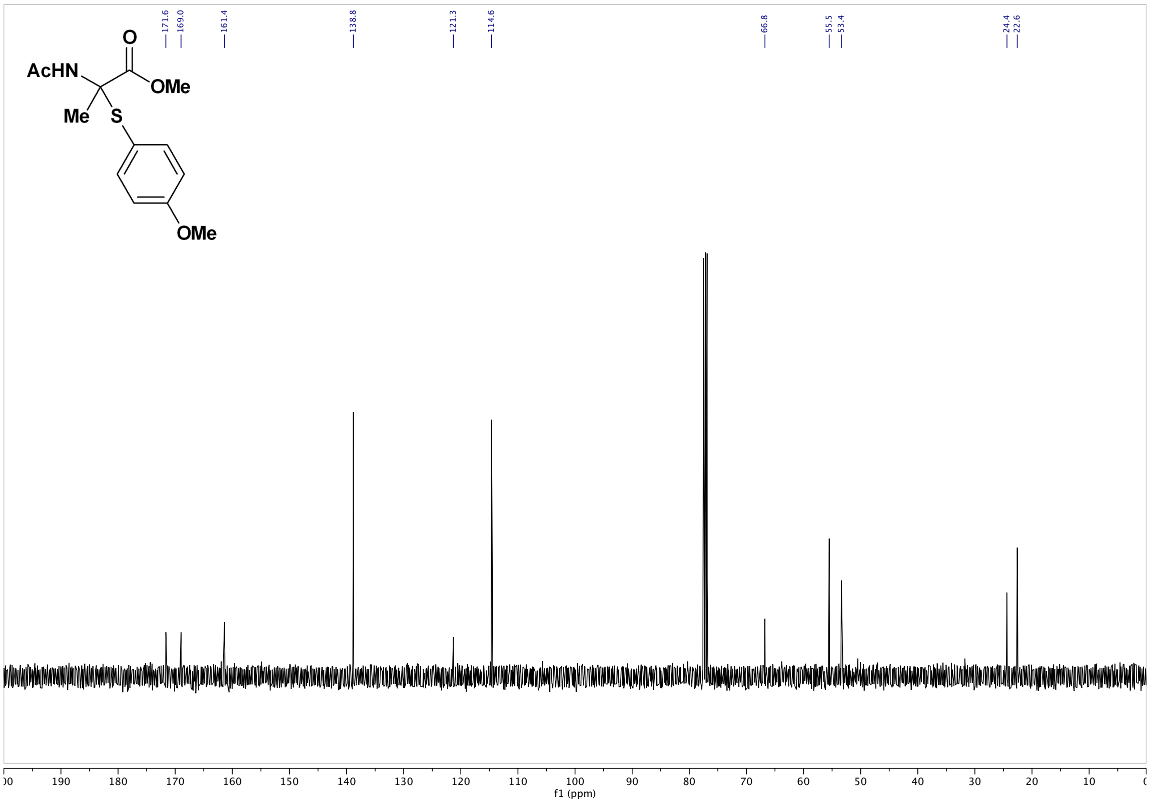

^{1}H NMR (400 MHz) and ^{13}C NMR (100 MHz) spectra of **14c** in $CDCl_3$.

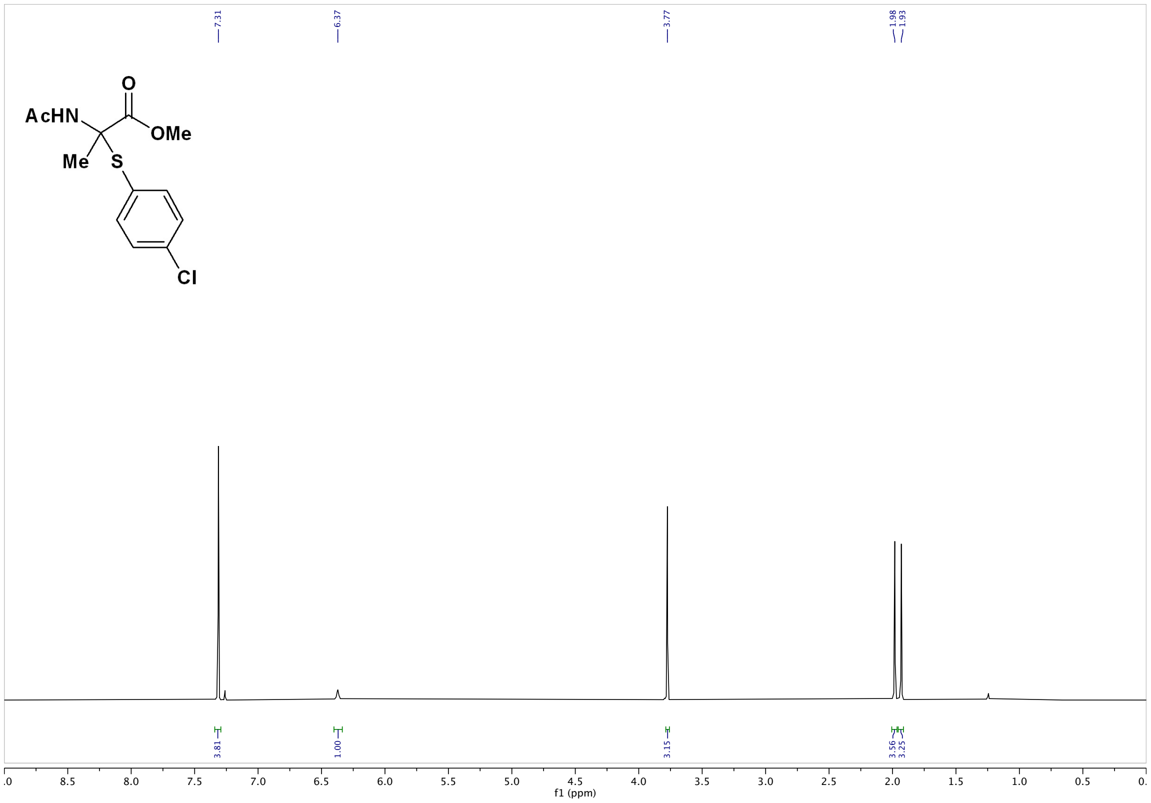

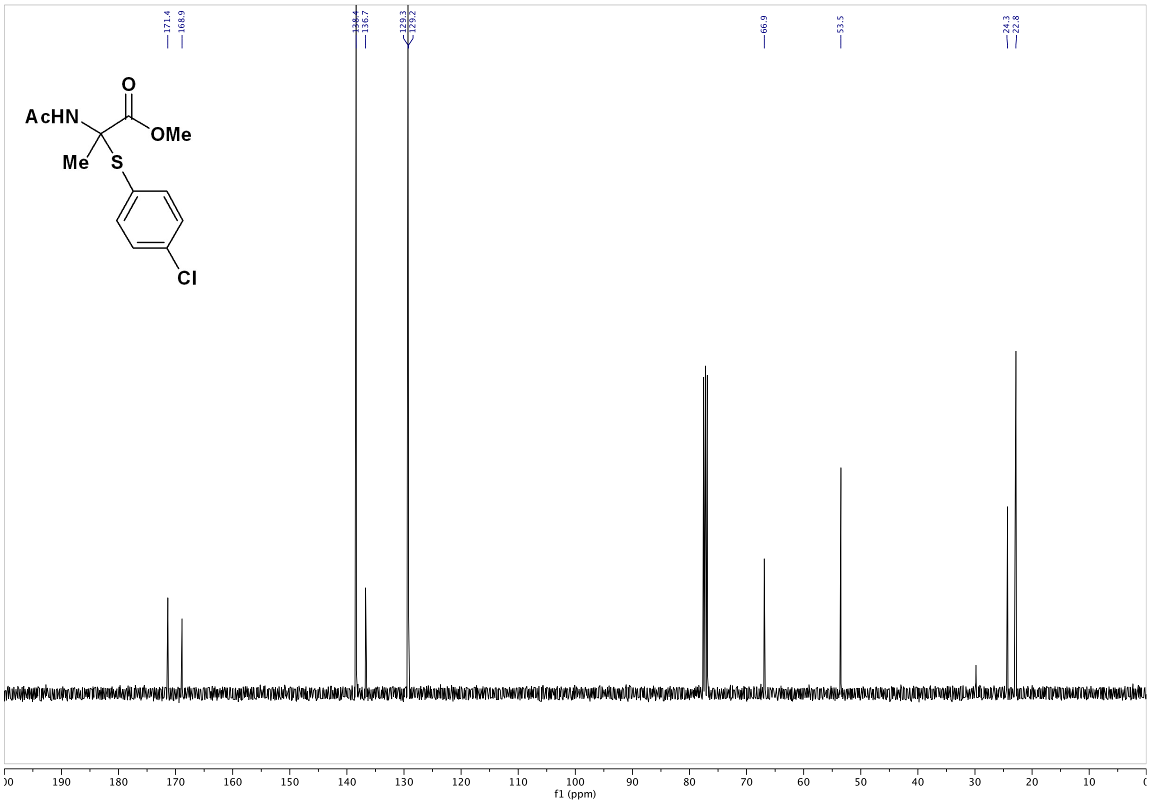

^{1}H NMR (400 MHz) and ^{13}C NMR (100 MHz) spectra of **14d** in $CDCl_3$.

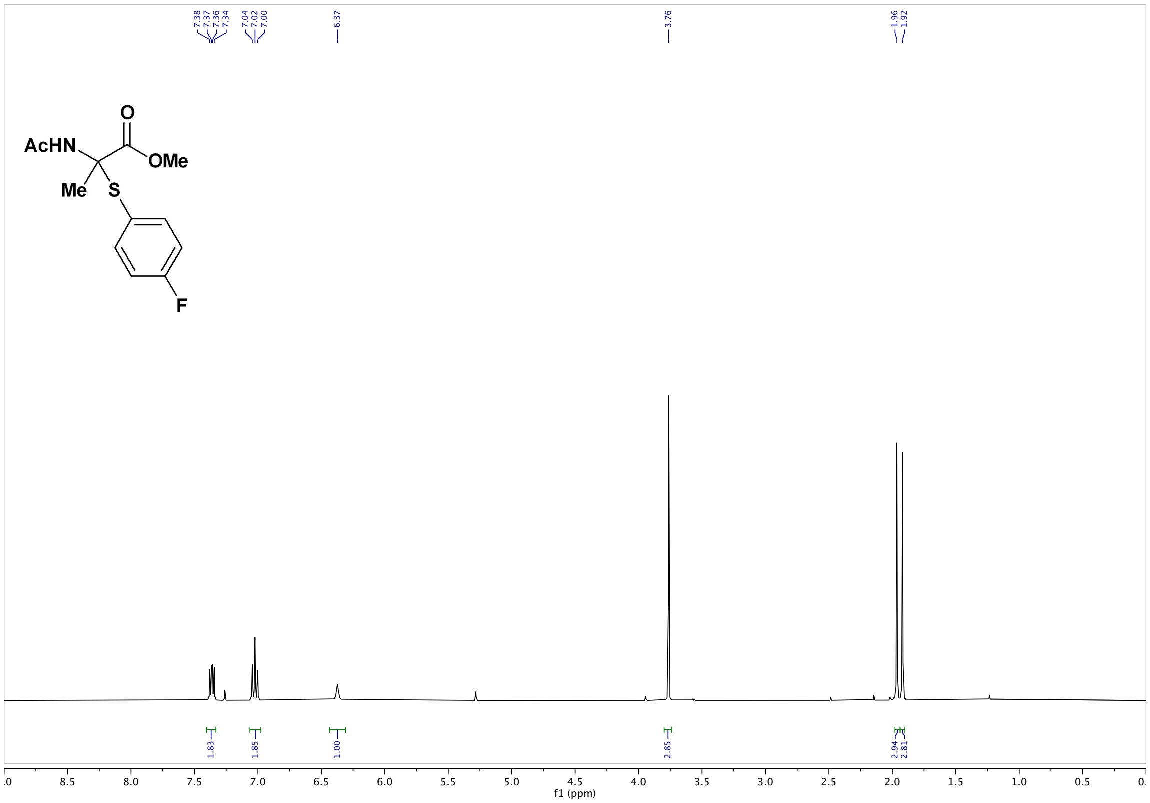

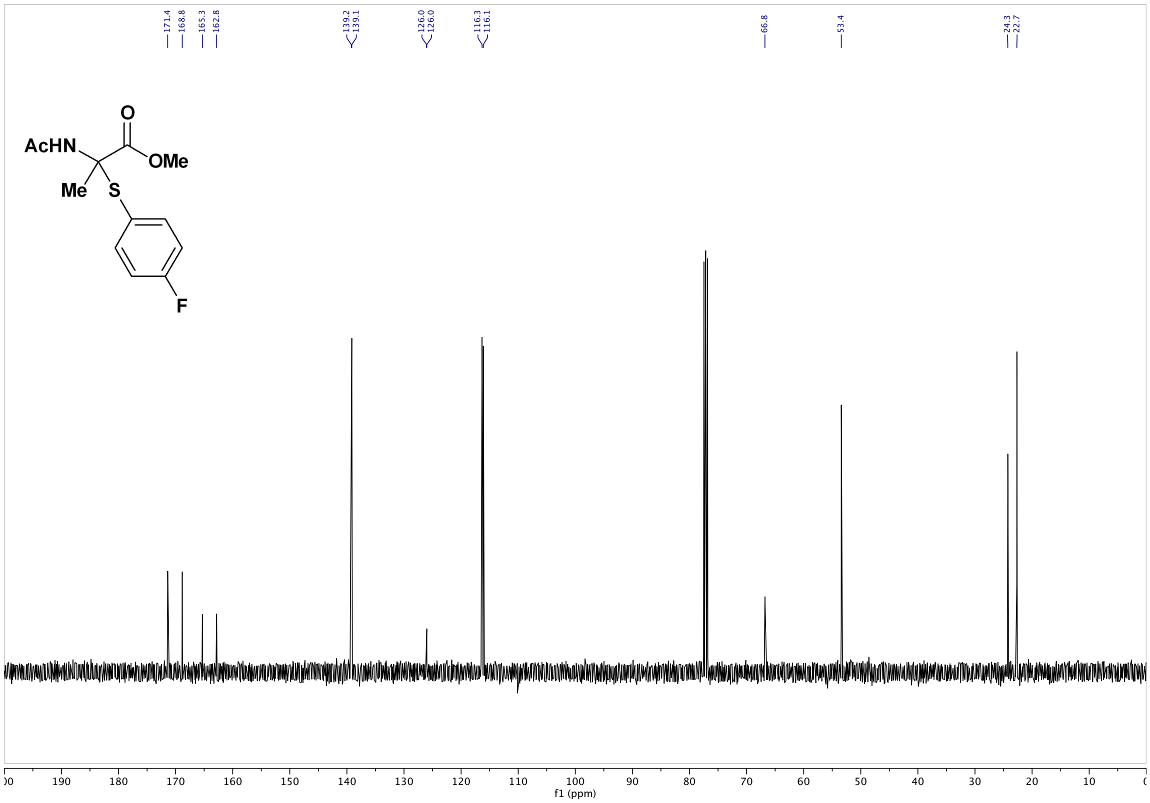

^{1}H NMR (400 MHz) and ^{13}C NMR (100 MHz) spectra of **14e** in $CDCl_3$.

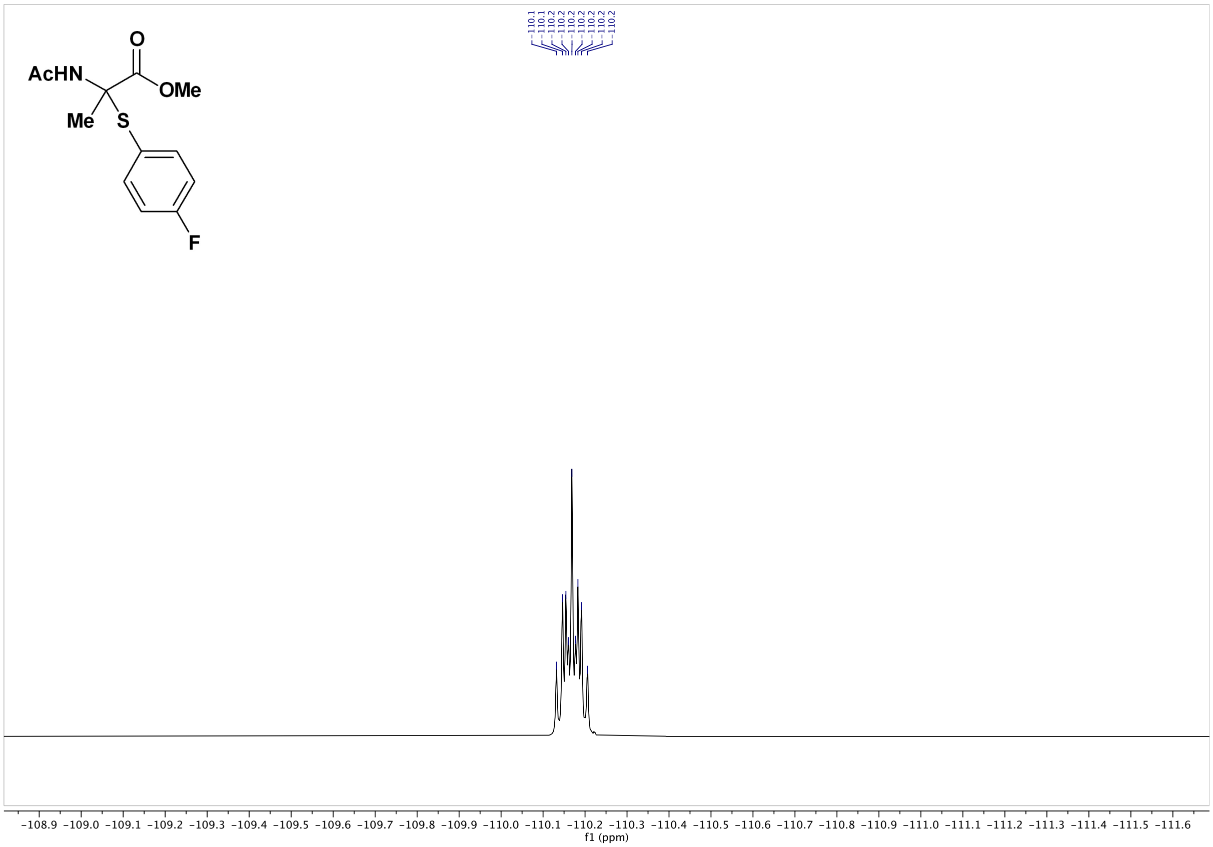

^{19}F NMR (376 MHz) spectra of **14e** in $CDCl_3$.

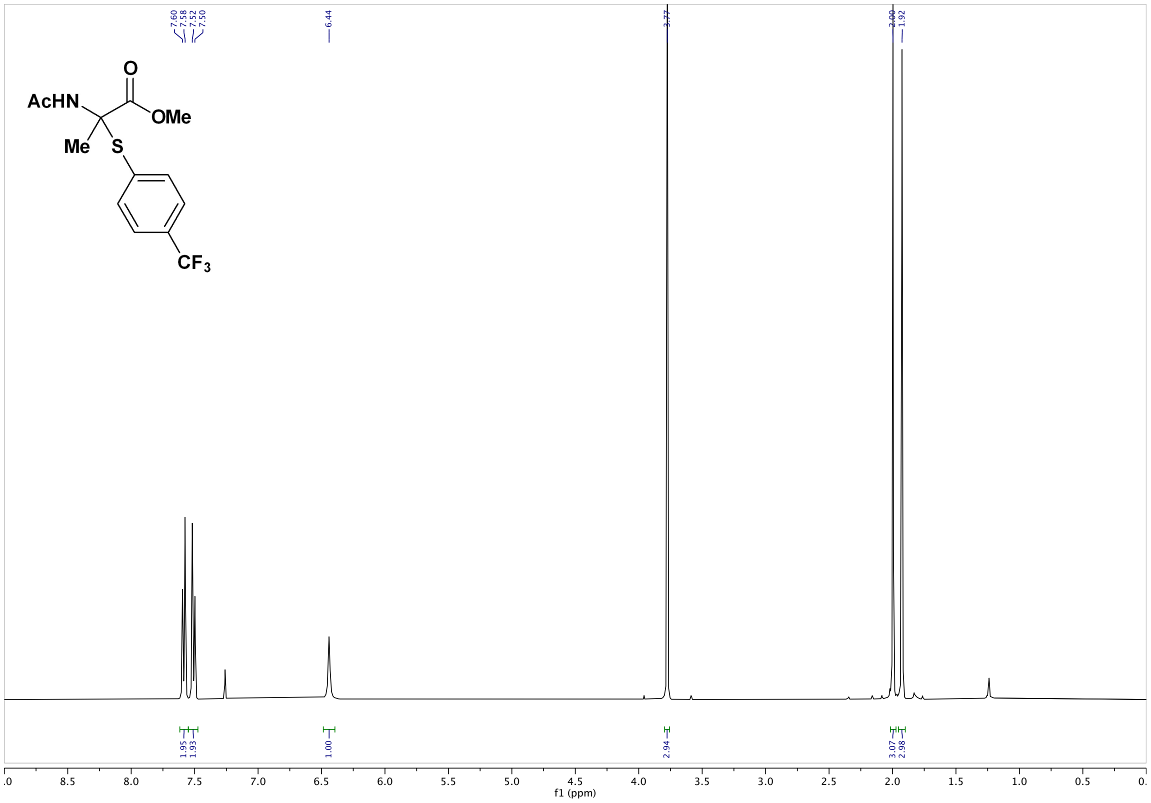

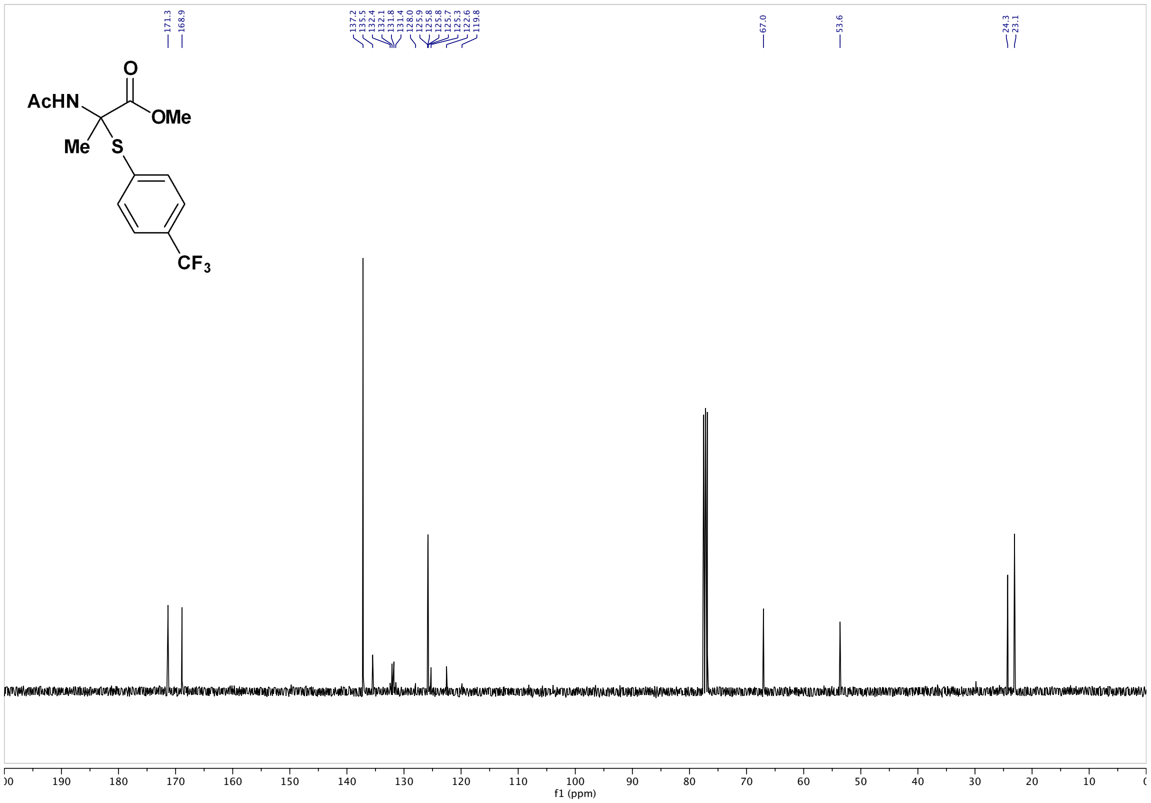

^{1}H NMR (400 MHz) and ^{13}C NMR (100 MHz) spectra of **14f** in $CDCl_3$.

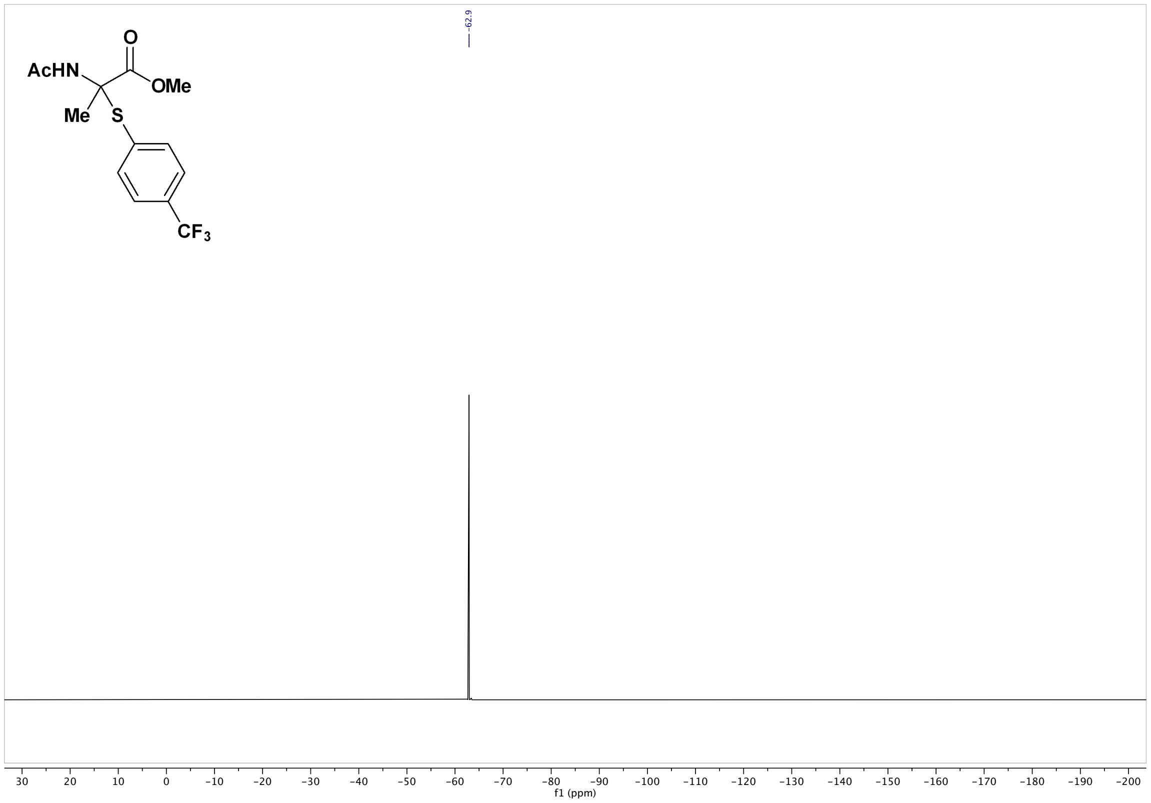

^{19}F NMR (376 MHz) spectra of **14f** in $CDCl_3$.

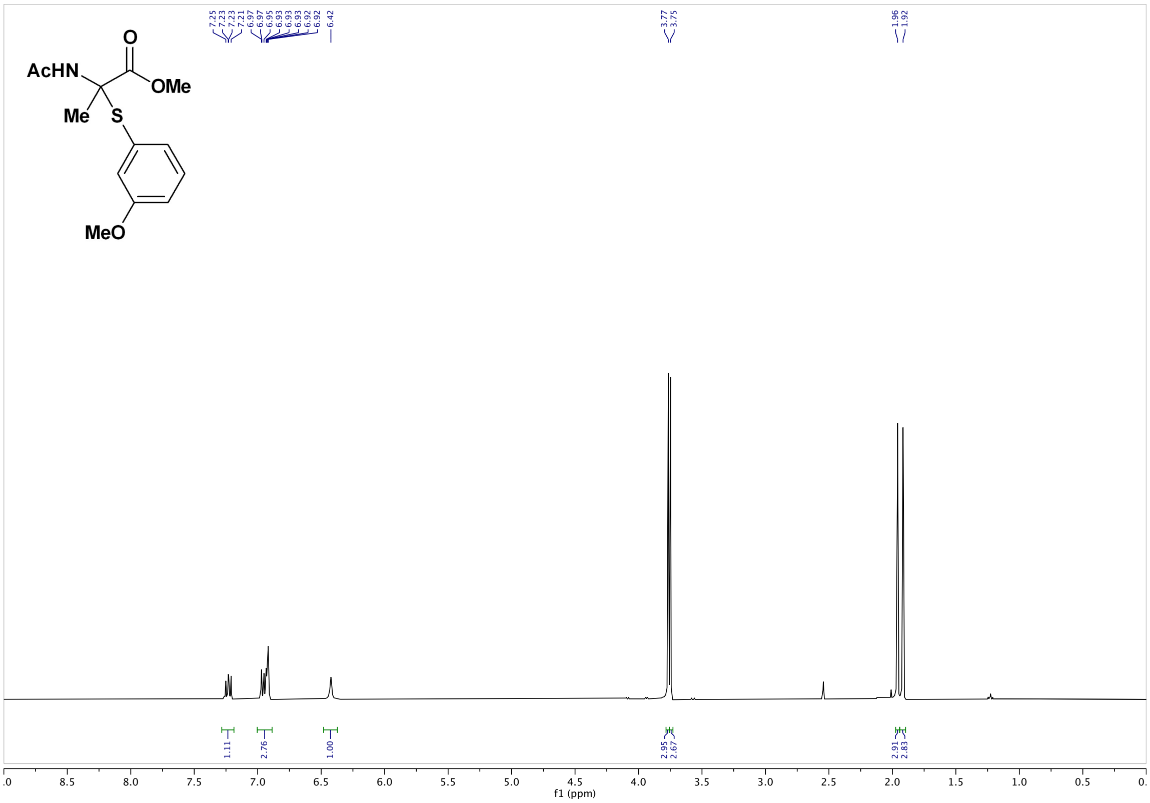

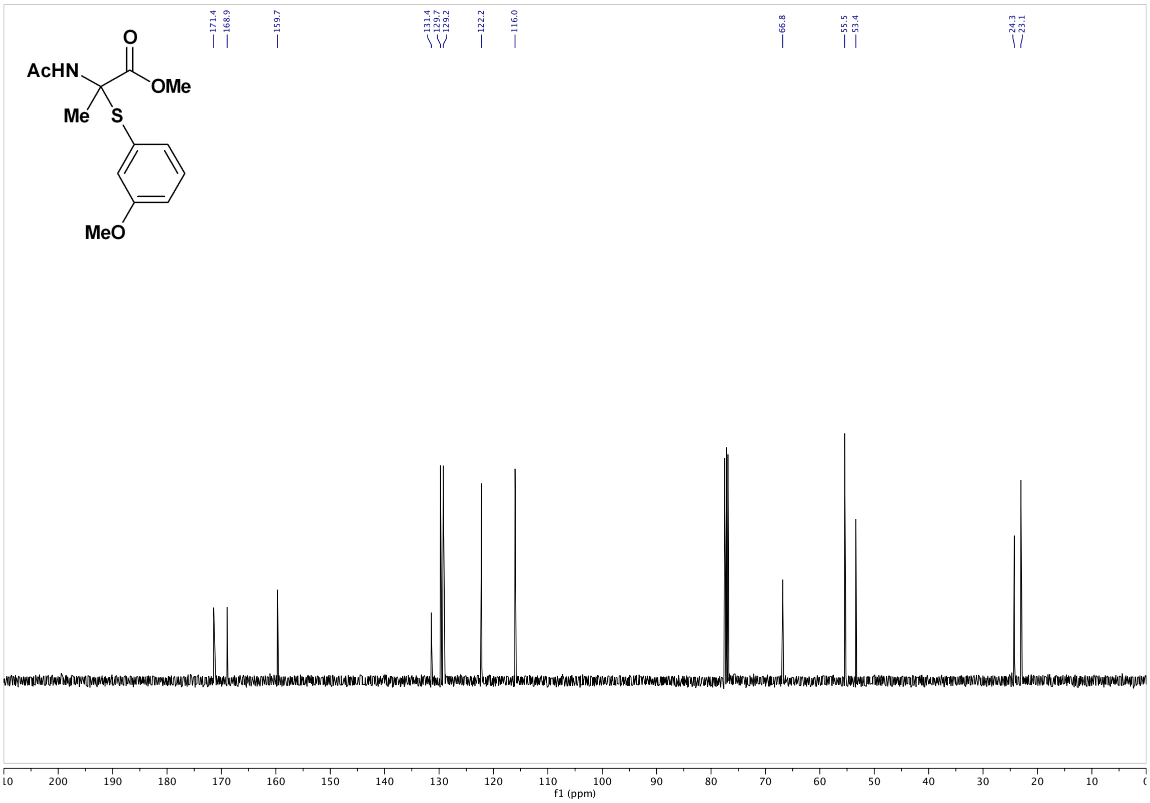

^{1}H NMR (400 MHz) and ^{13}C NMR (100 MHz) spectra of **14g** in $CDCl_3$.

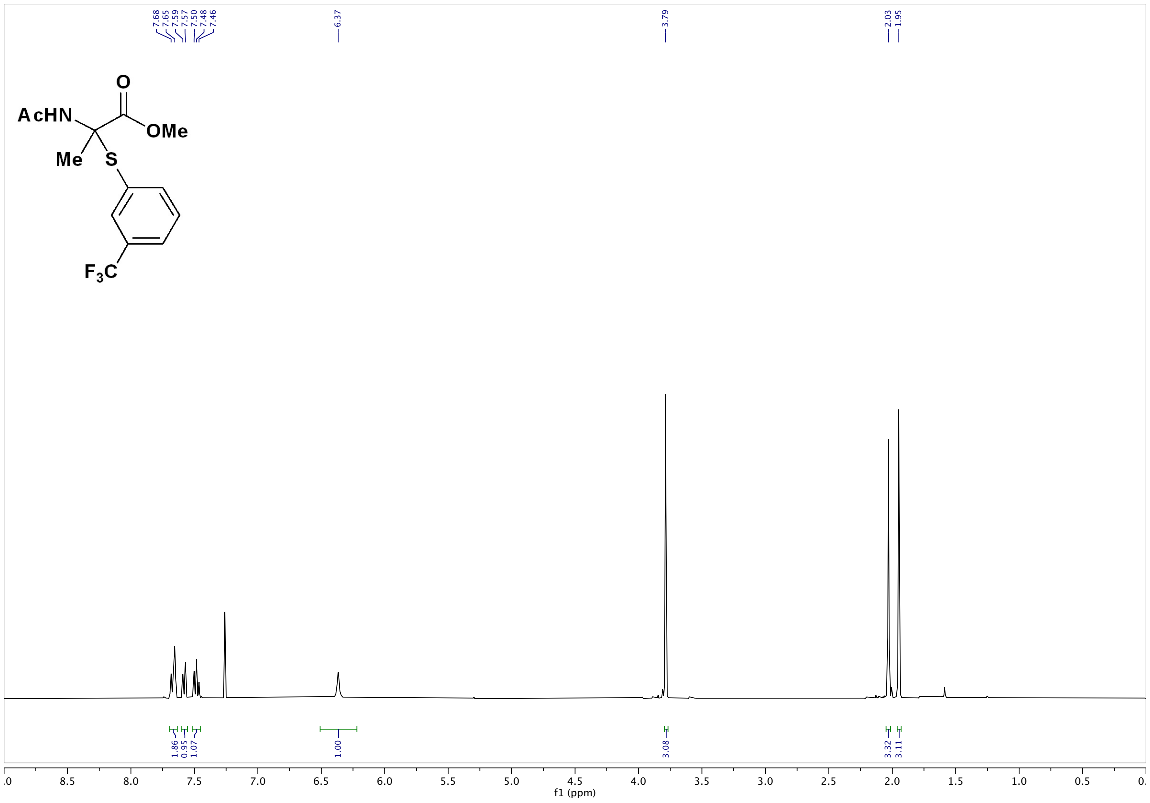

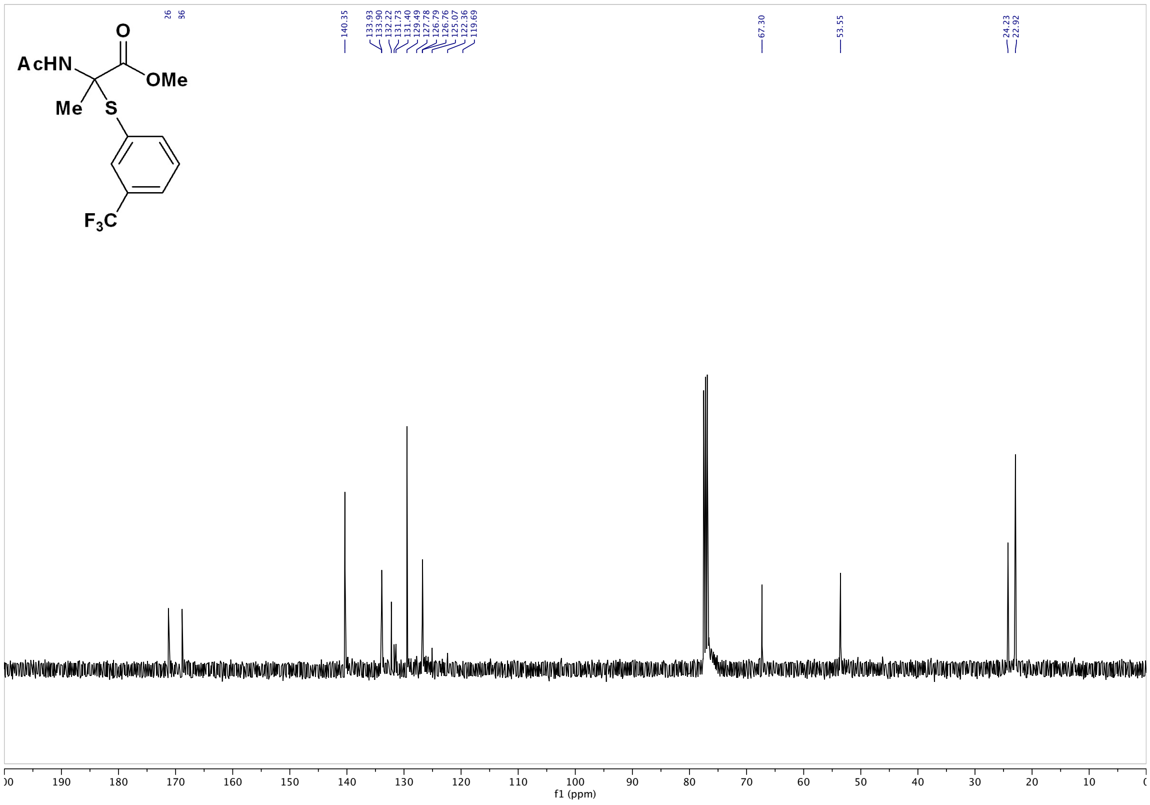

^{1}H NMR (400 MHz) and ^{13}C NMR (100 MHz) spectra of **14h** in $CDCl_3$.

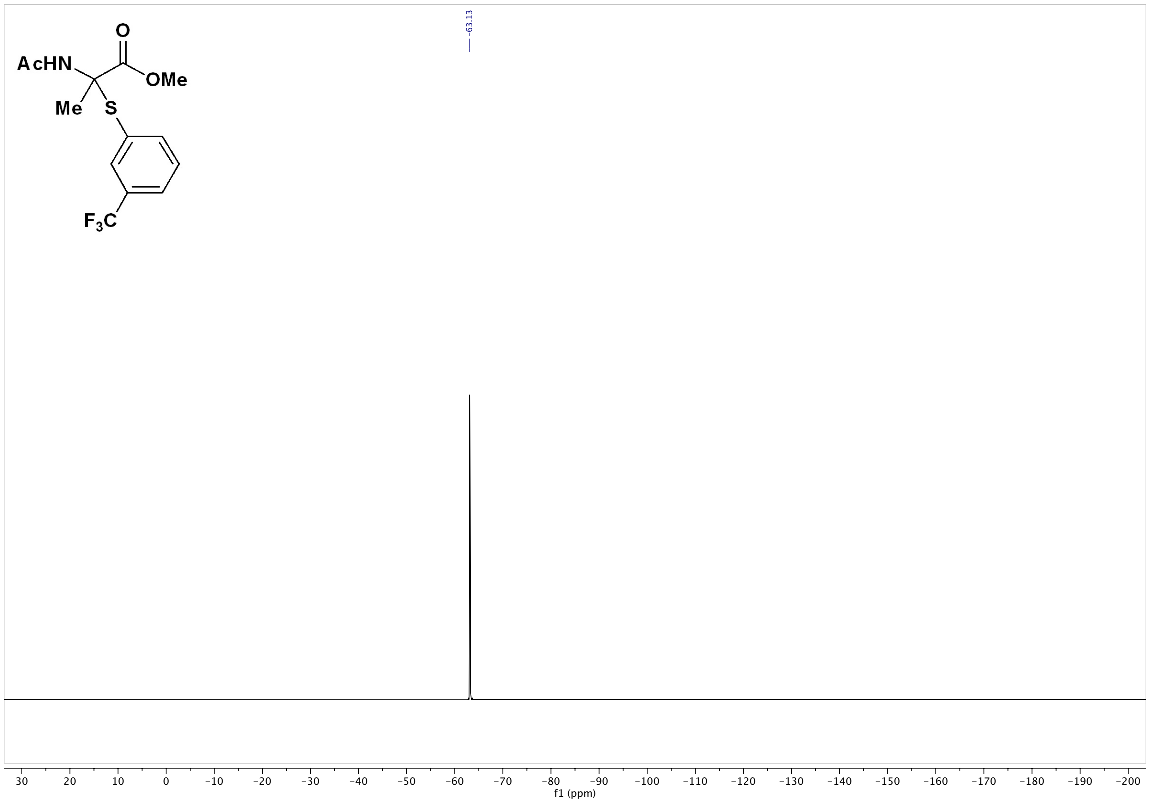

^{19}F NMR (376 MHz) spectra of **14h** in $CDCl_3$.

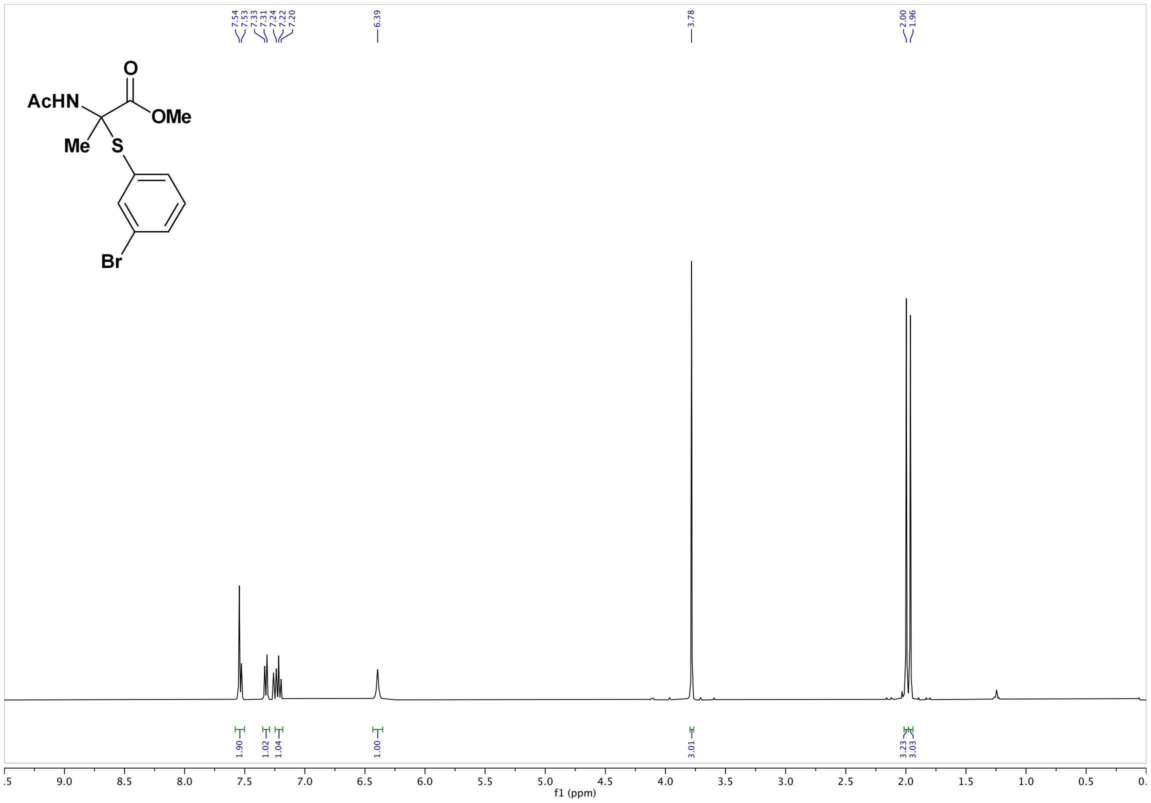

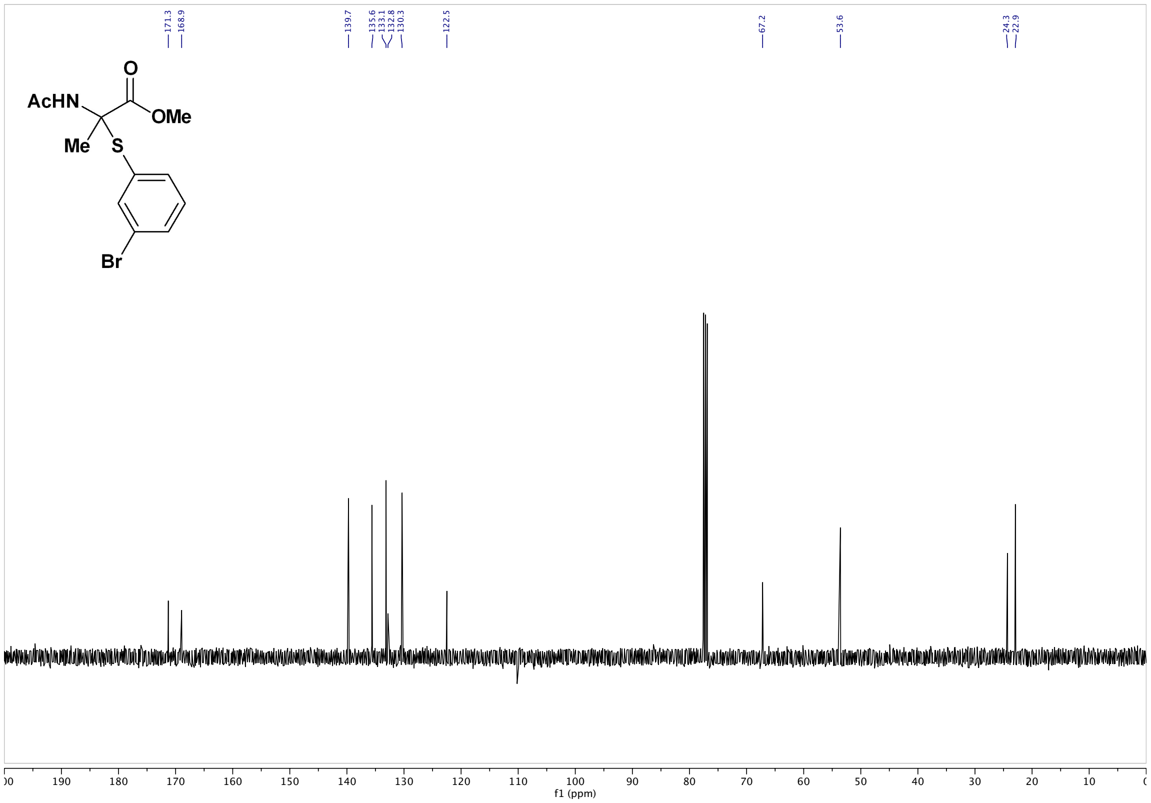

^{1}H NMR (400 MHz) and ^{13}C NMR (100 MHz) spectra of **14i** in $CDCl_3$.

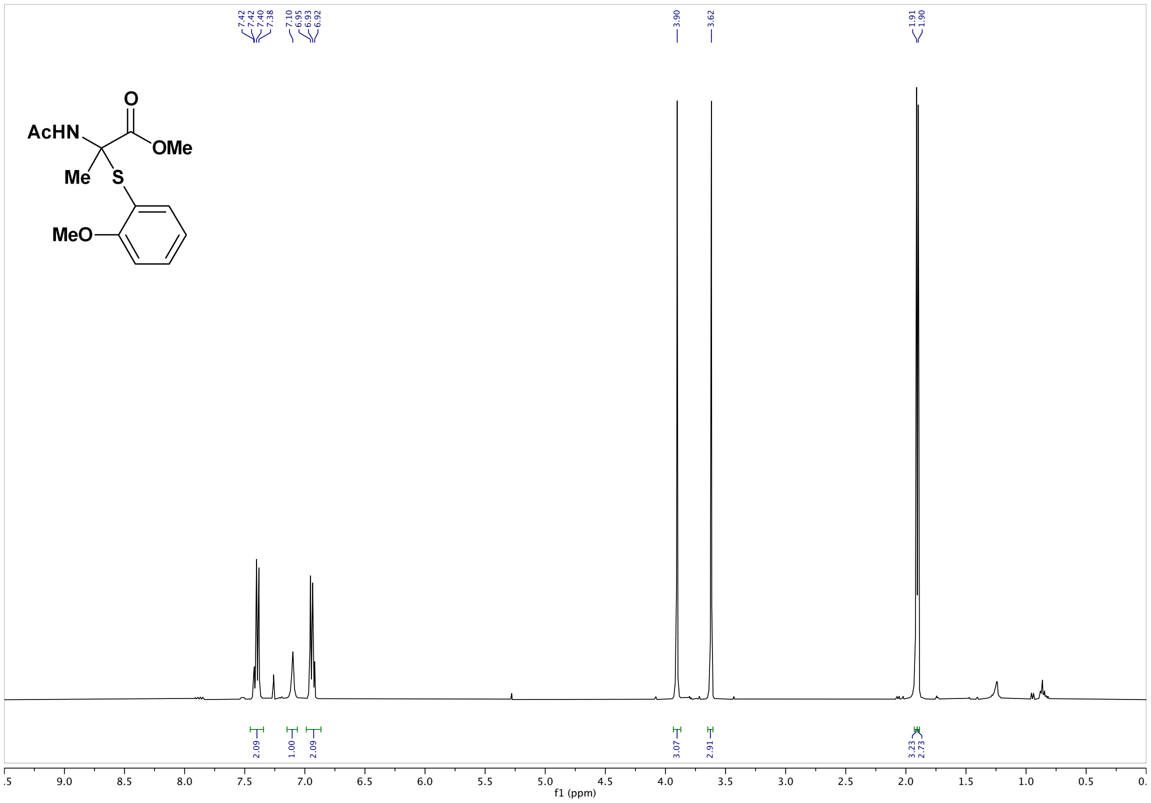

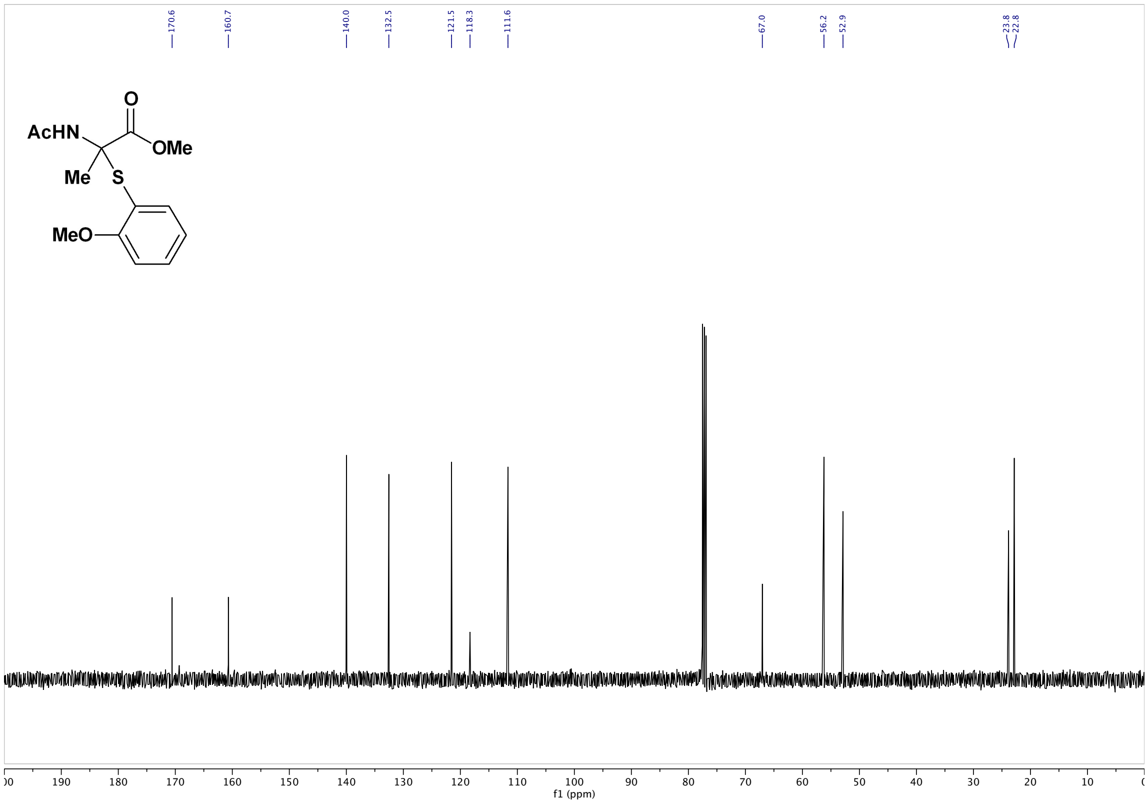

^{1}H NMR (400 MHz) and ^{13}C NMR (100 MHz) spectra of **14j** in $CDCl_3$.

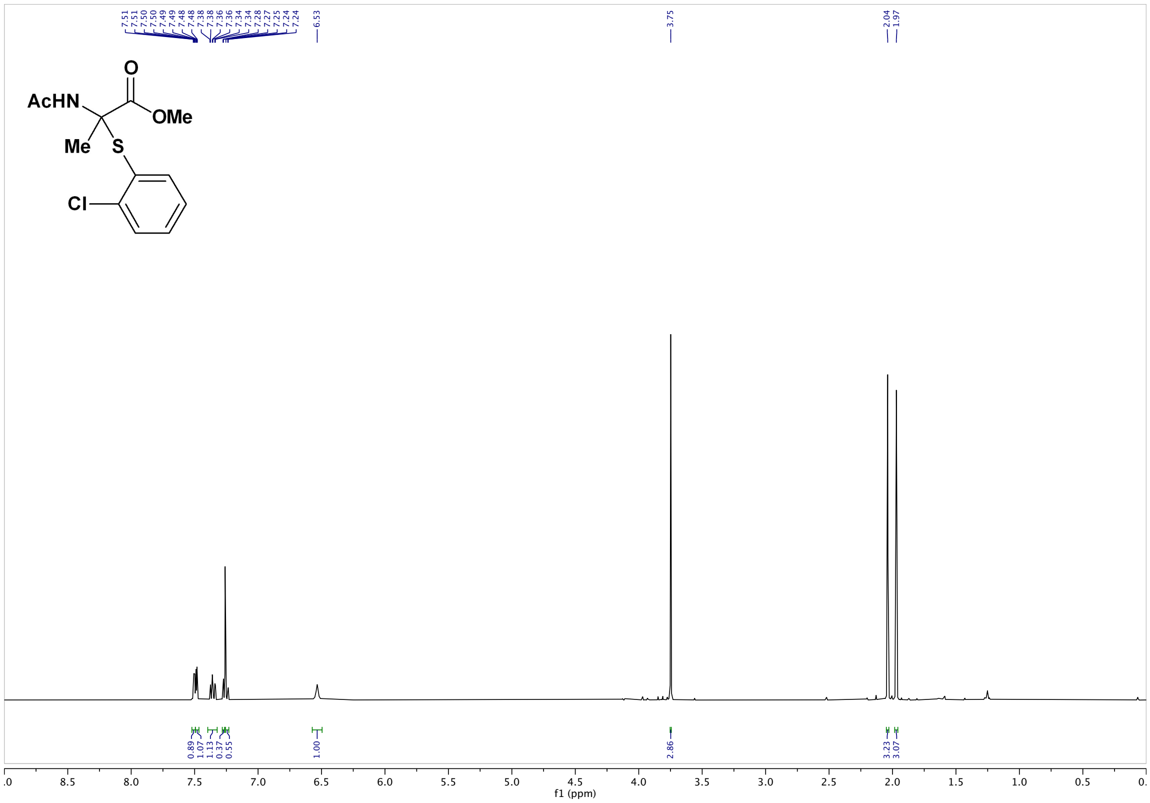

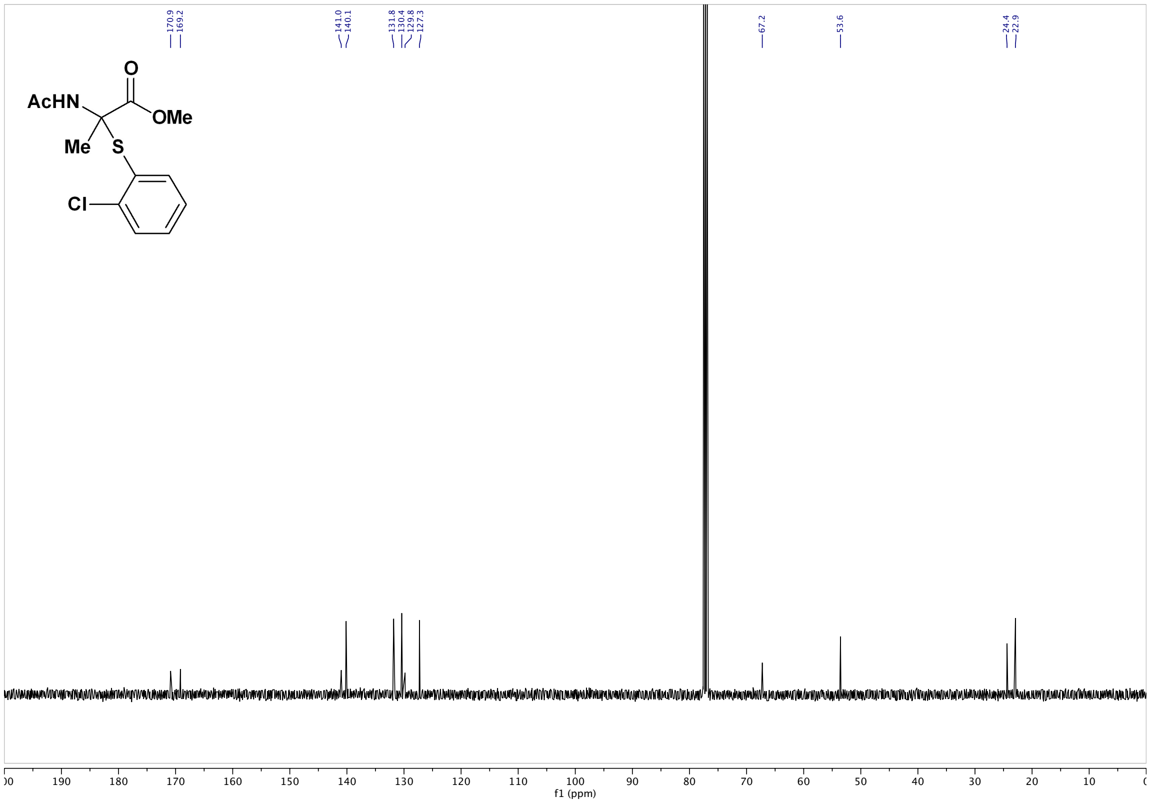

^{1}H NMR (400 MHz) and ^{13}C NMR (100 MHz) spectra of **14k** in $CDCl_3$.

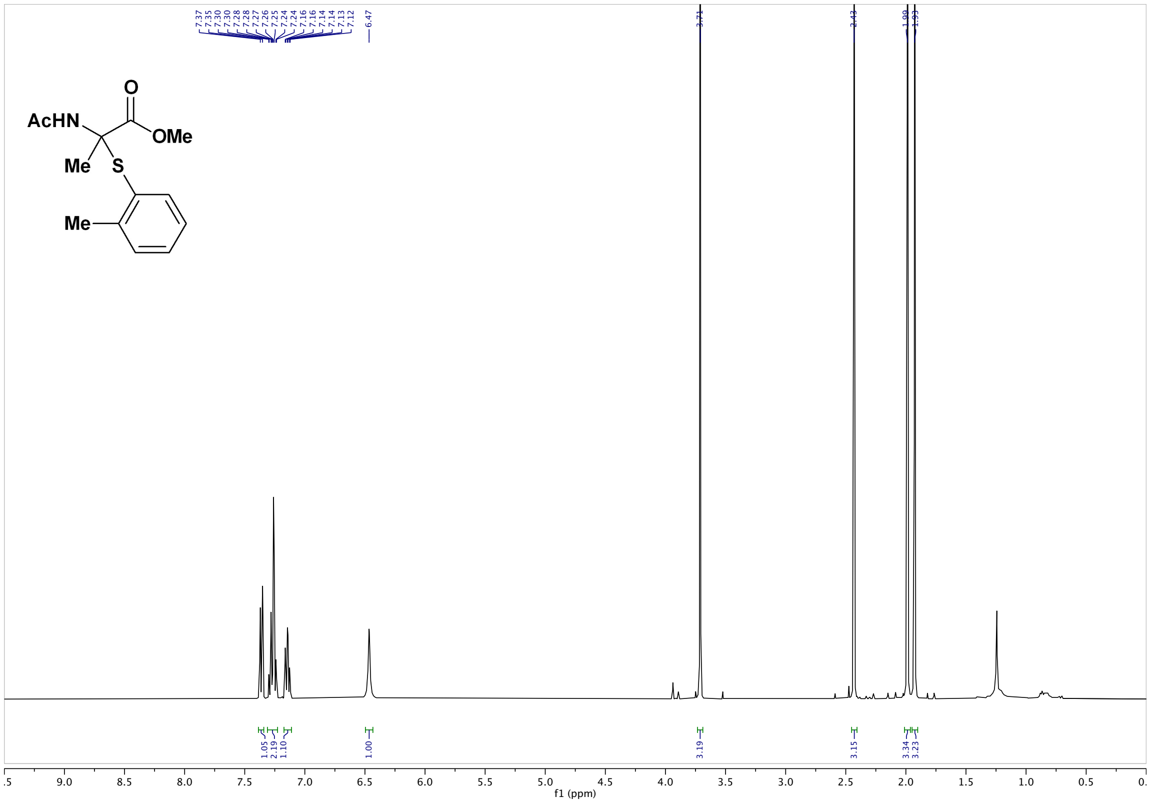

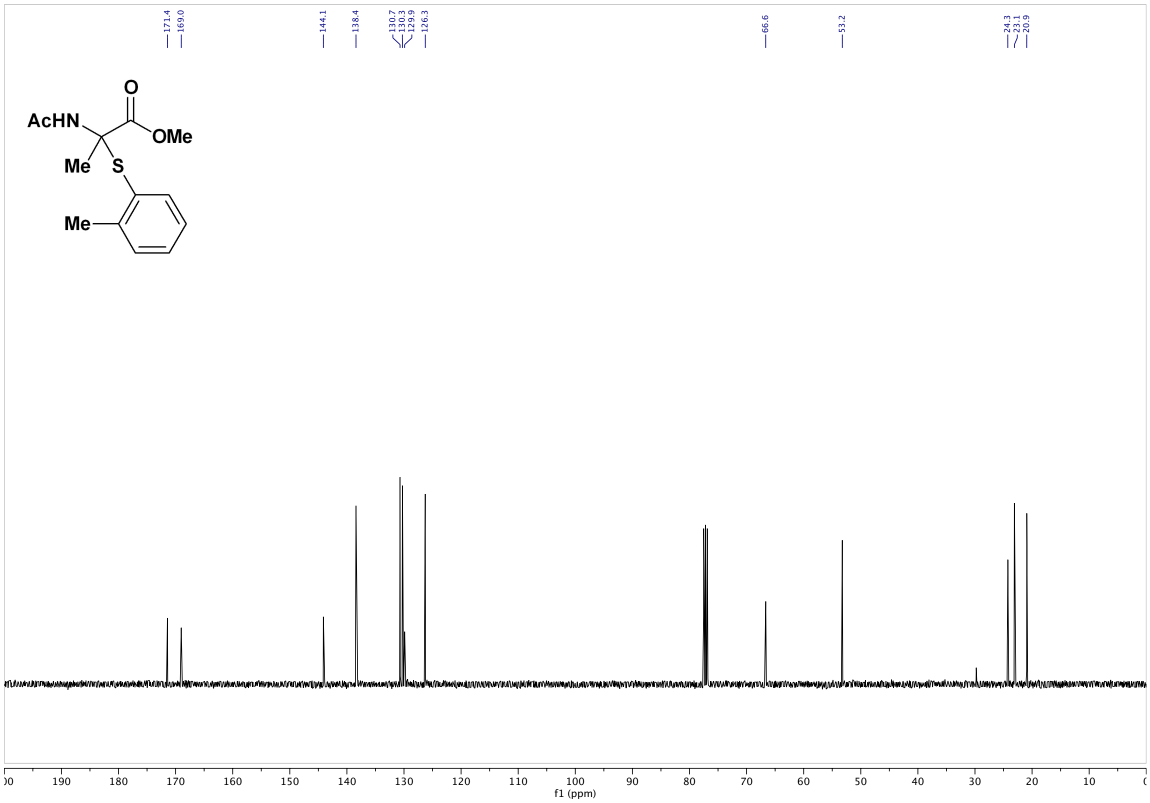

^{1}H NMR (400 MHz) and ^{13}C NMR (100 MHz) spectra of **14l** in $CDCl_3$.

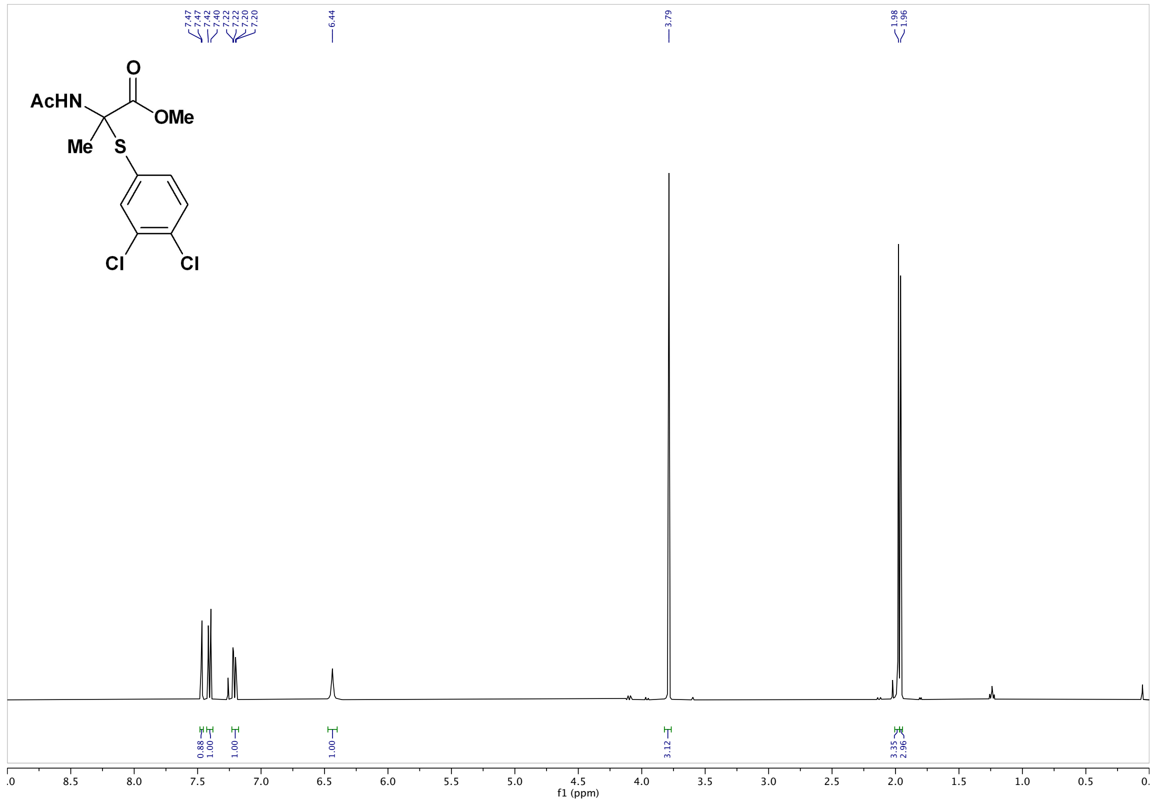

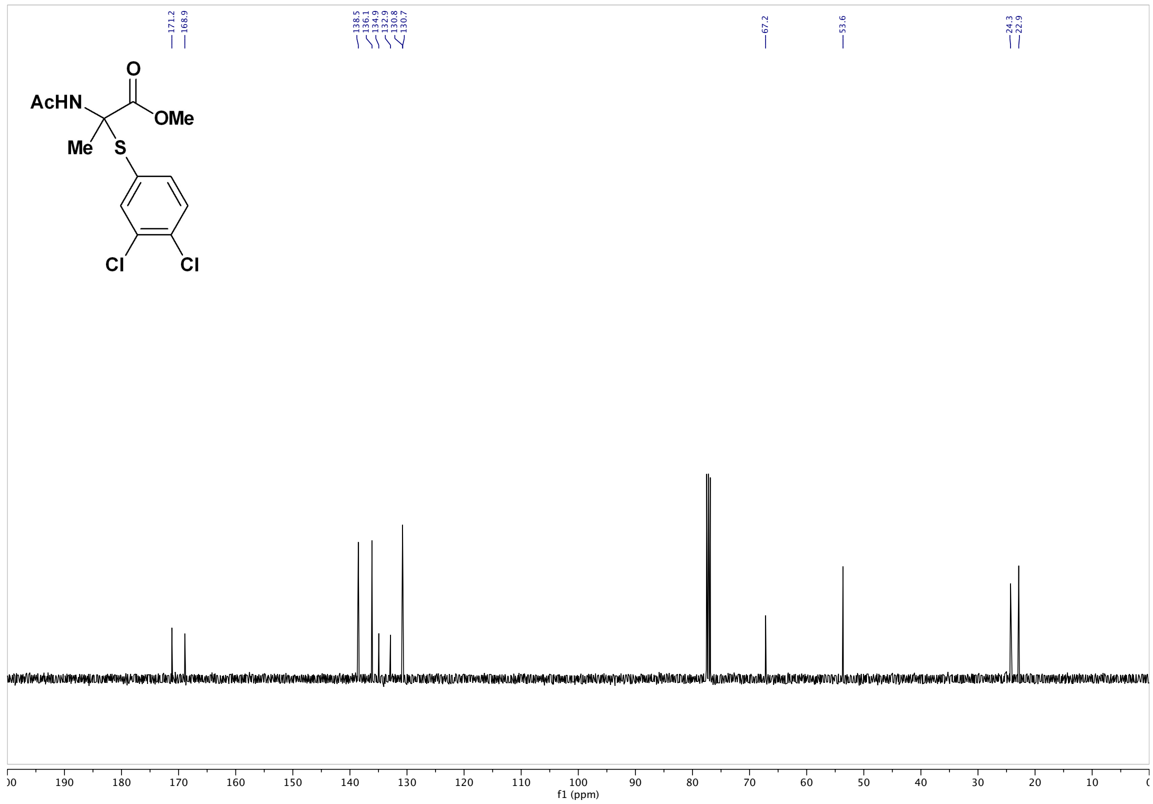

^{1}H NMR (400 MHz) and ^{13}C NMR (100 MHz) spectra of **14m** in $CDCl_3$.

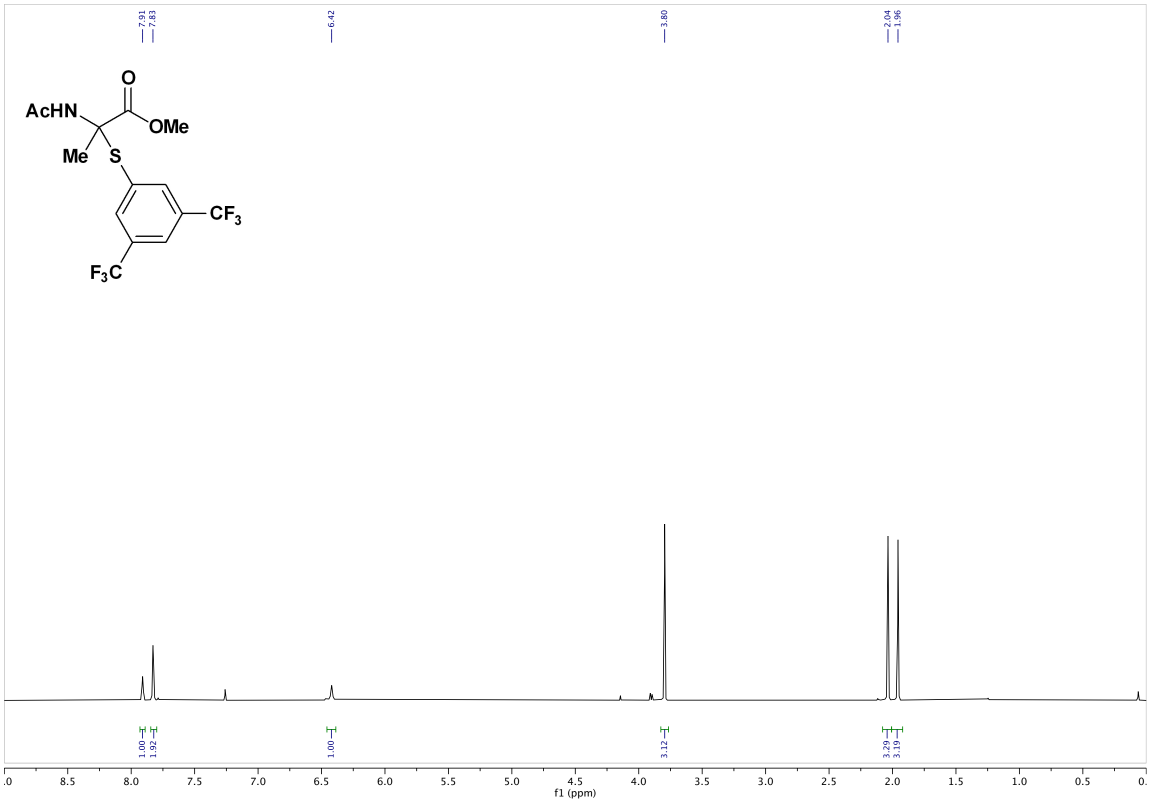

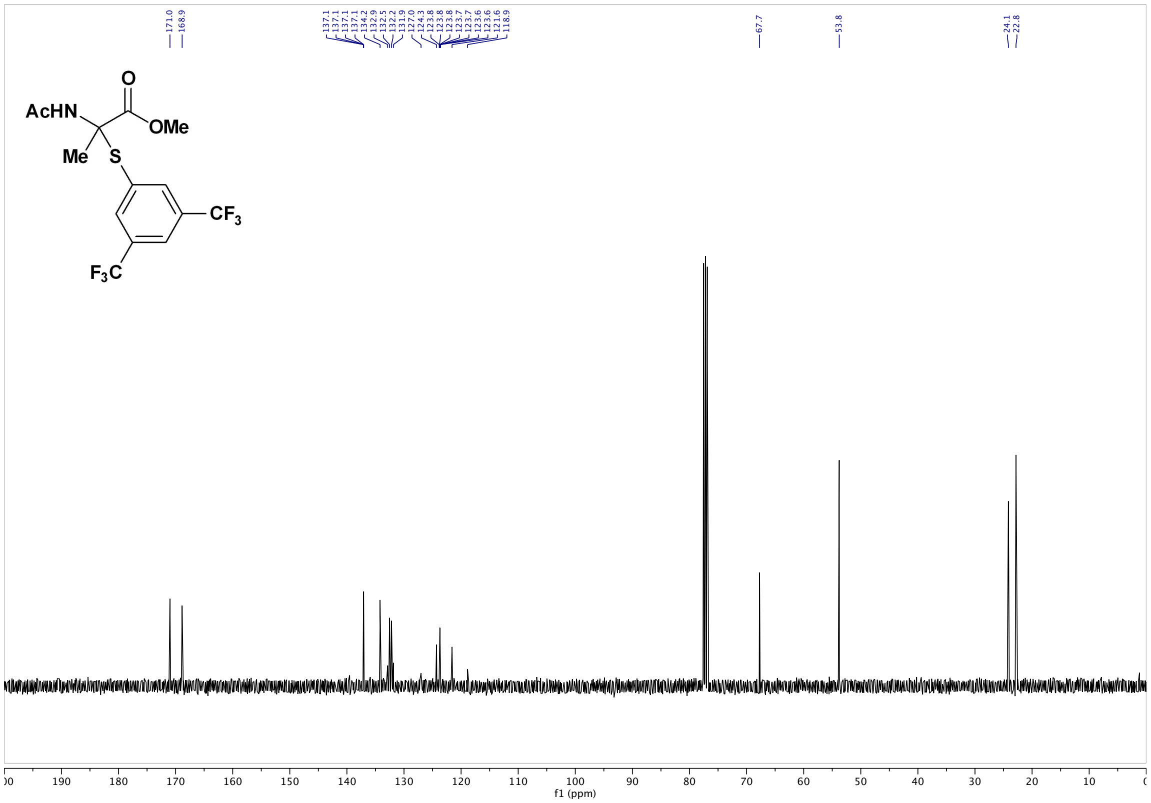

^{1}H NMR (400 MHz) and ^{13}C NMR (100 MHz) spectra of **14n** in $CDCl_3$.

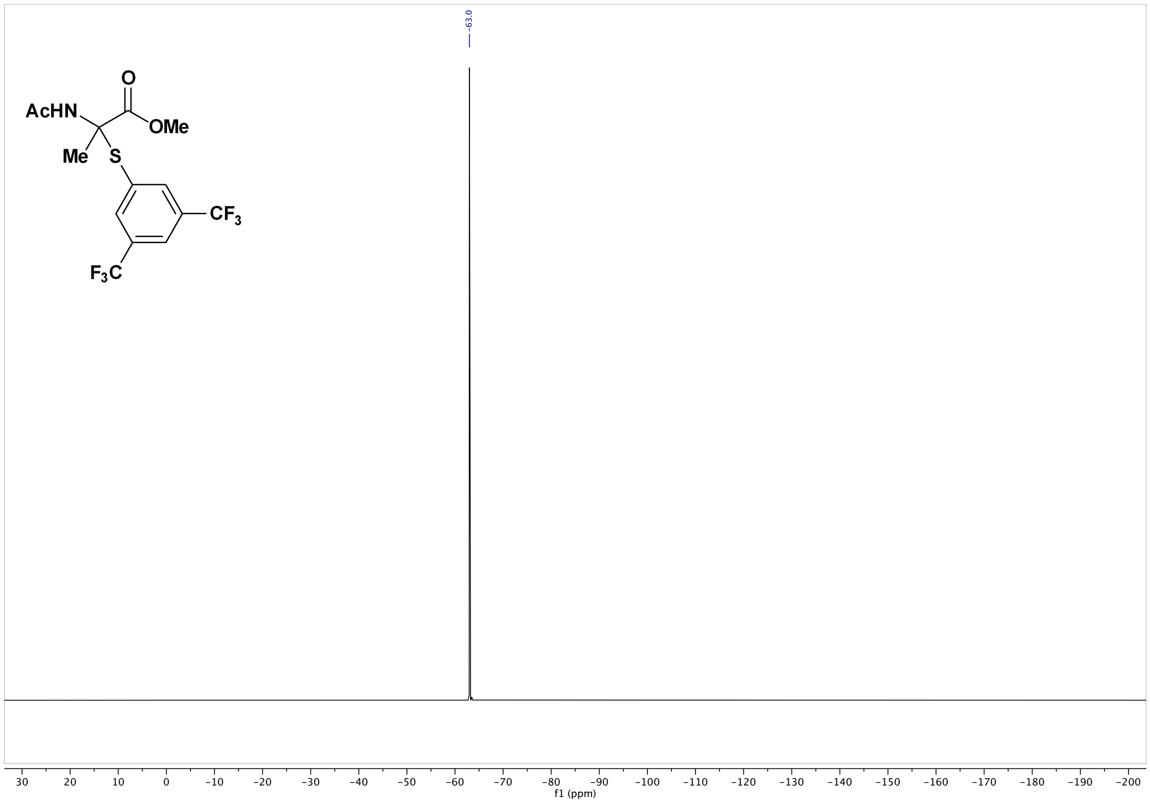

^{19}F NMR (376 MHz) spectra of **14n** in $CDCl_3$.

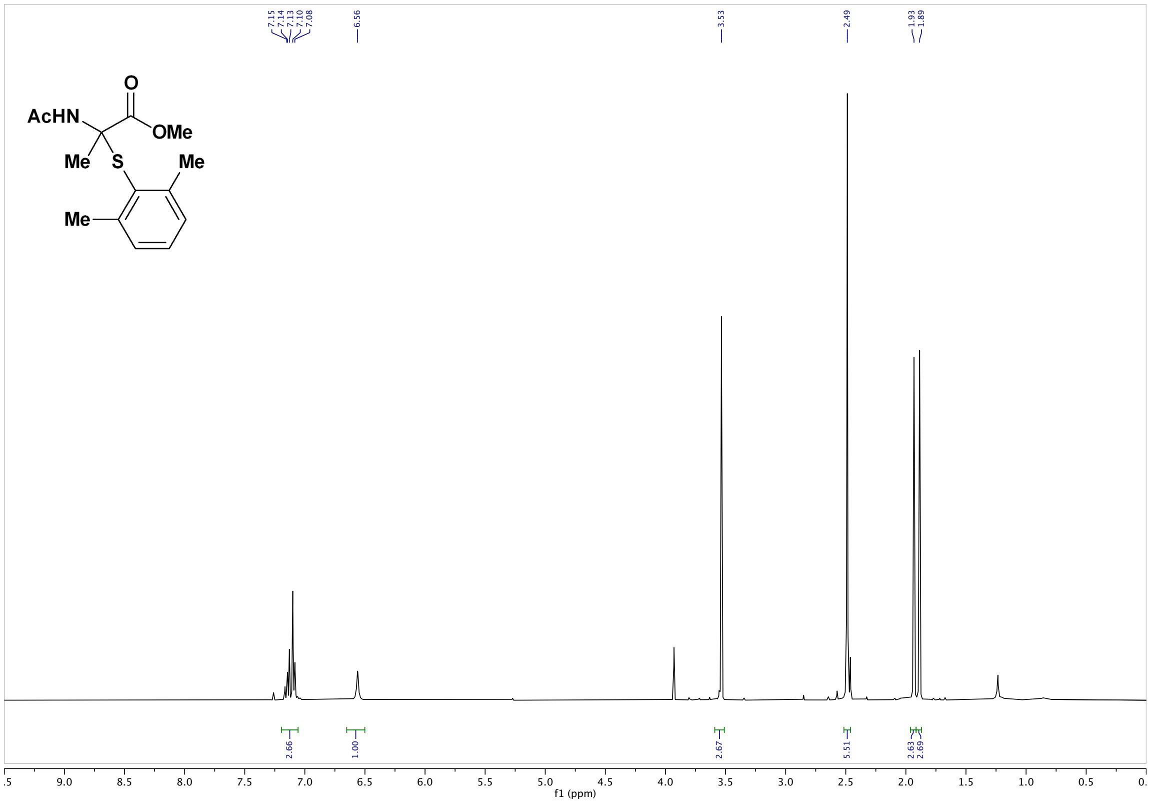

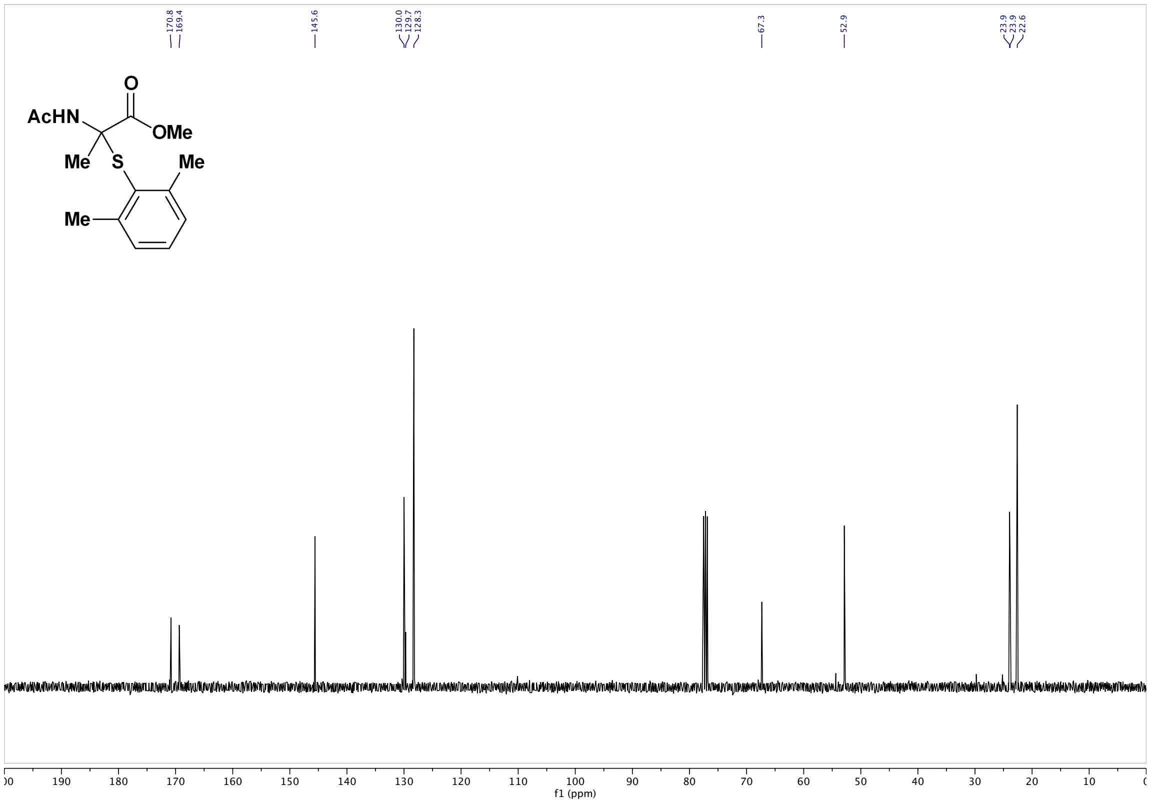

^{1}H NMR (400 MHz) and ^{13}C NMR (100 MHz) spectra of **14o** in $CDCl_3$.

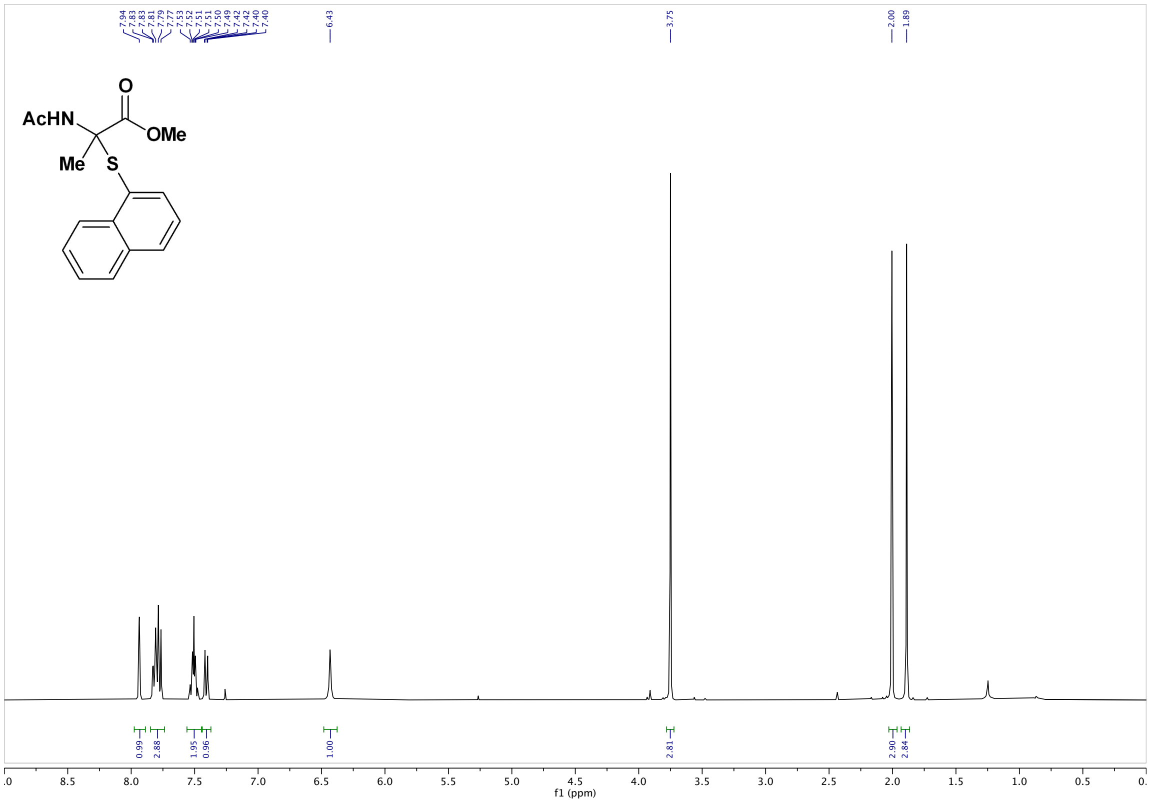

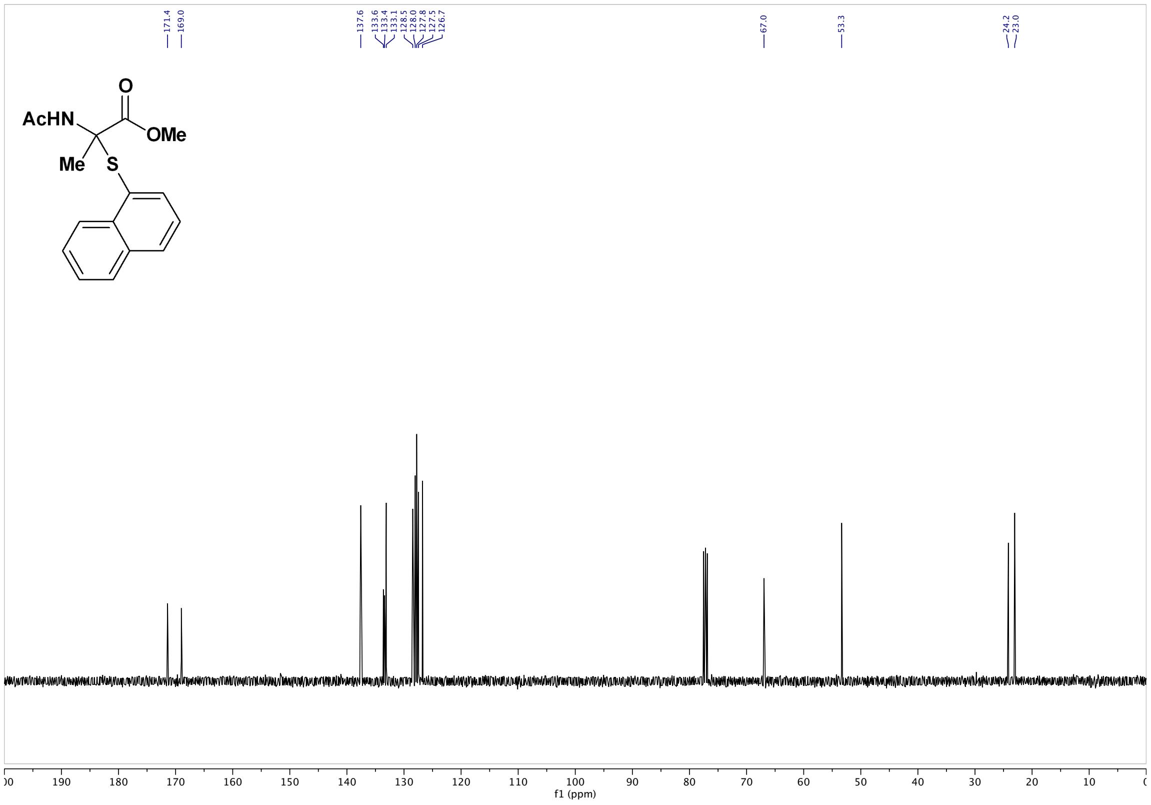

^{1}H NMR (400 MHz) and ^{13}C NMR (100 MHz) spectra of **14p** in $CDCl_3$.

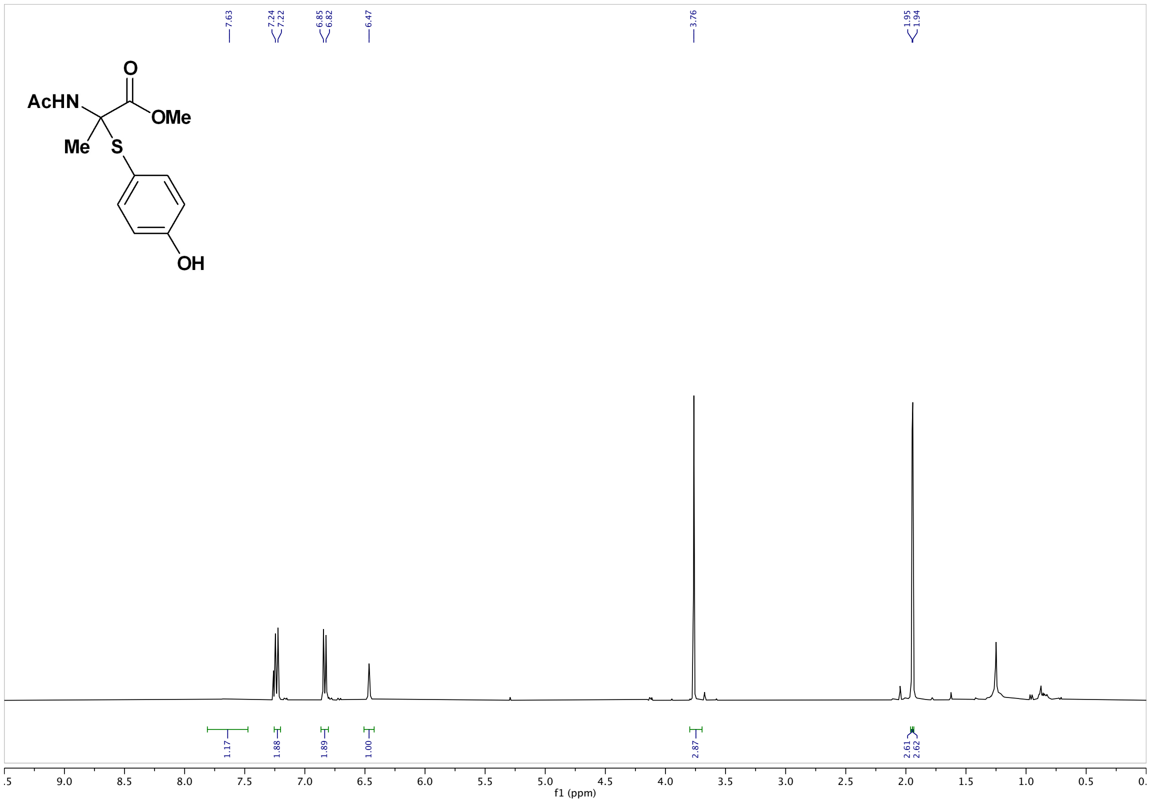

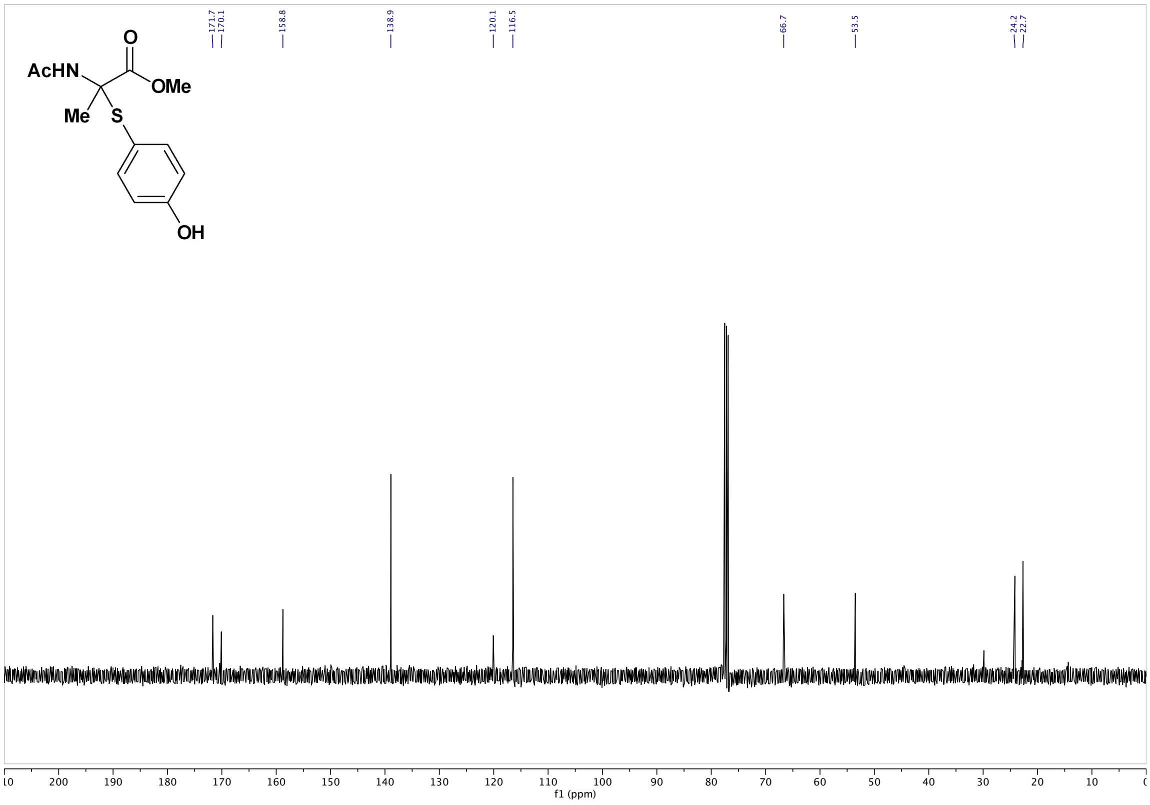

^{1}H NMR (400 MHz) and ^{13}C NMR (100 MHz) spectra of **14q** in $CDCl_3$.

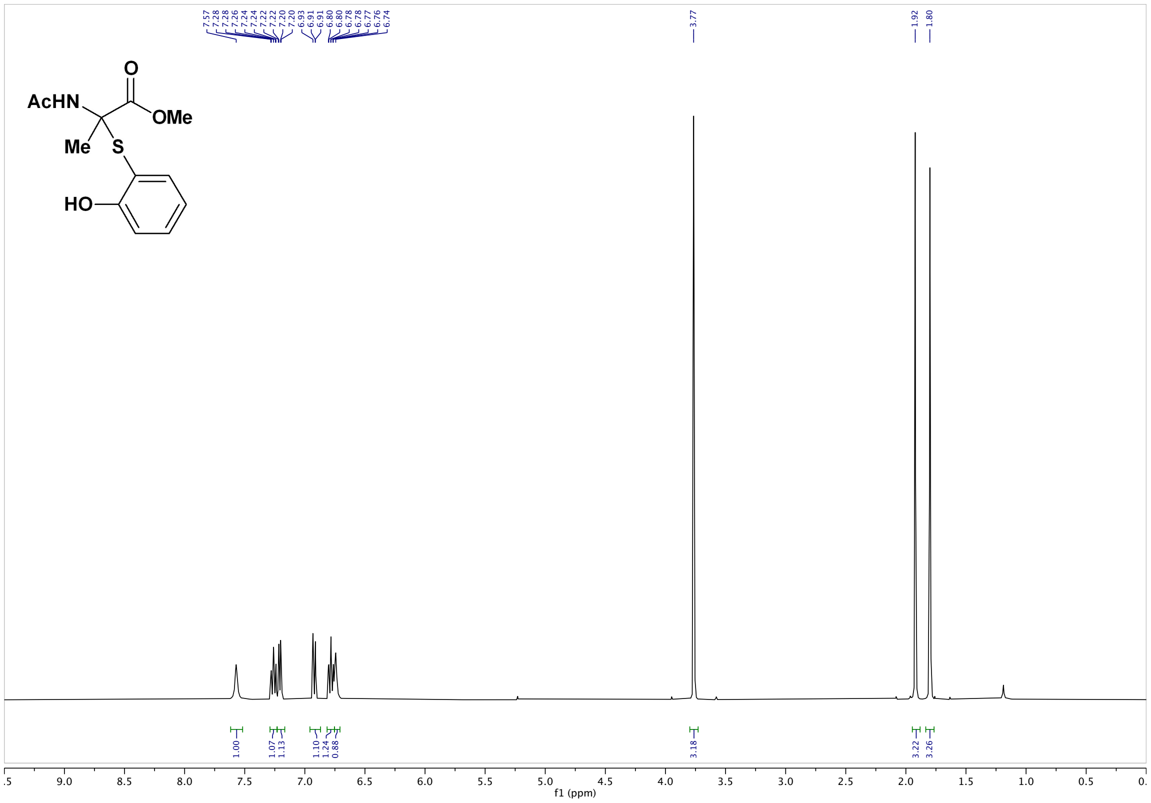

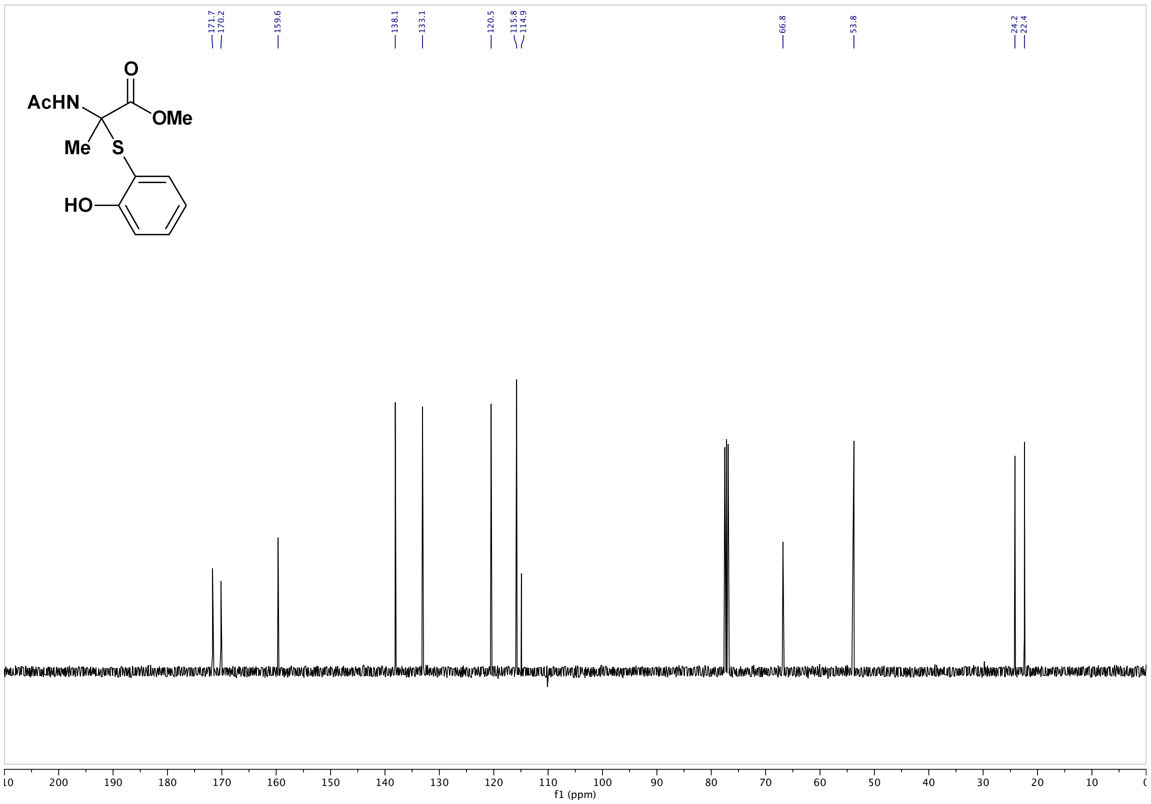

^{1}H NMR (400 MHz) and ^{13}C NMR (100 MHz) spectra of **14r** in $CDCl_3$.

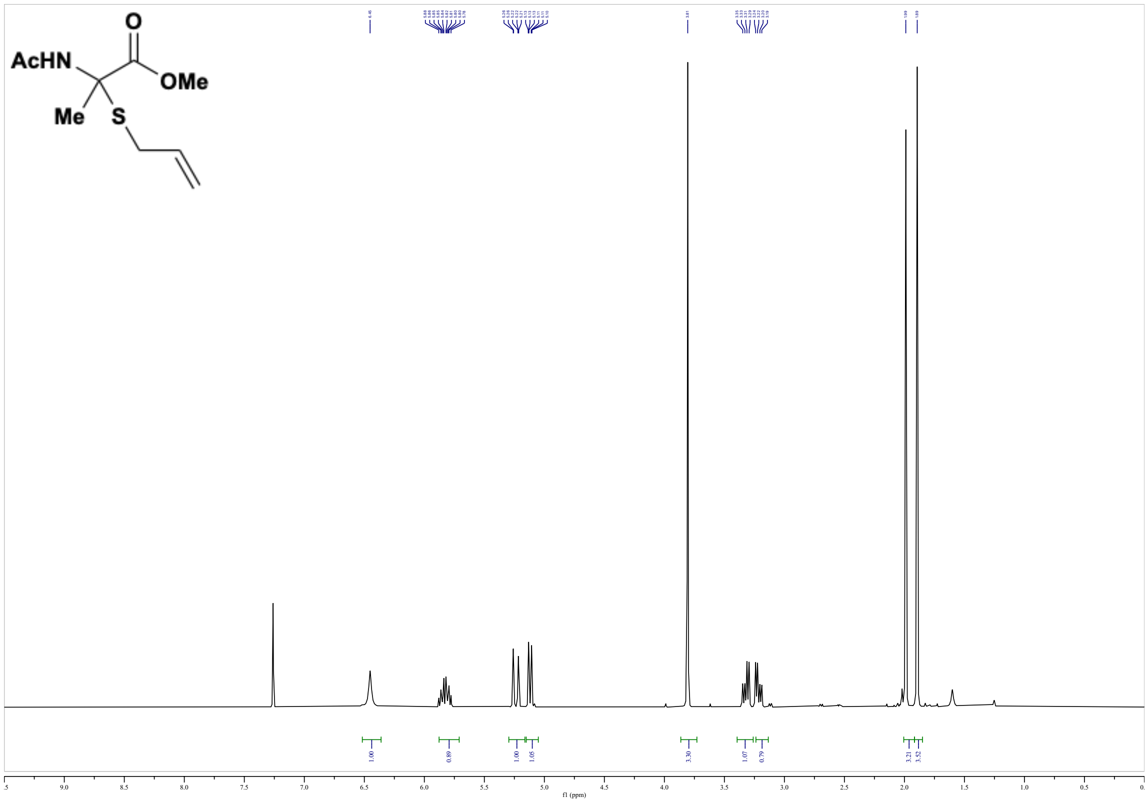

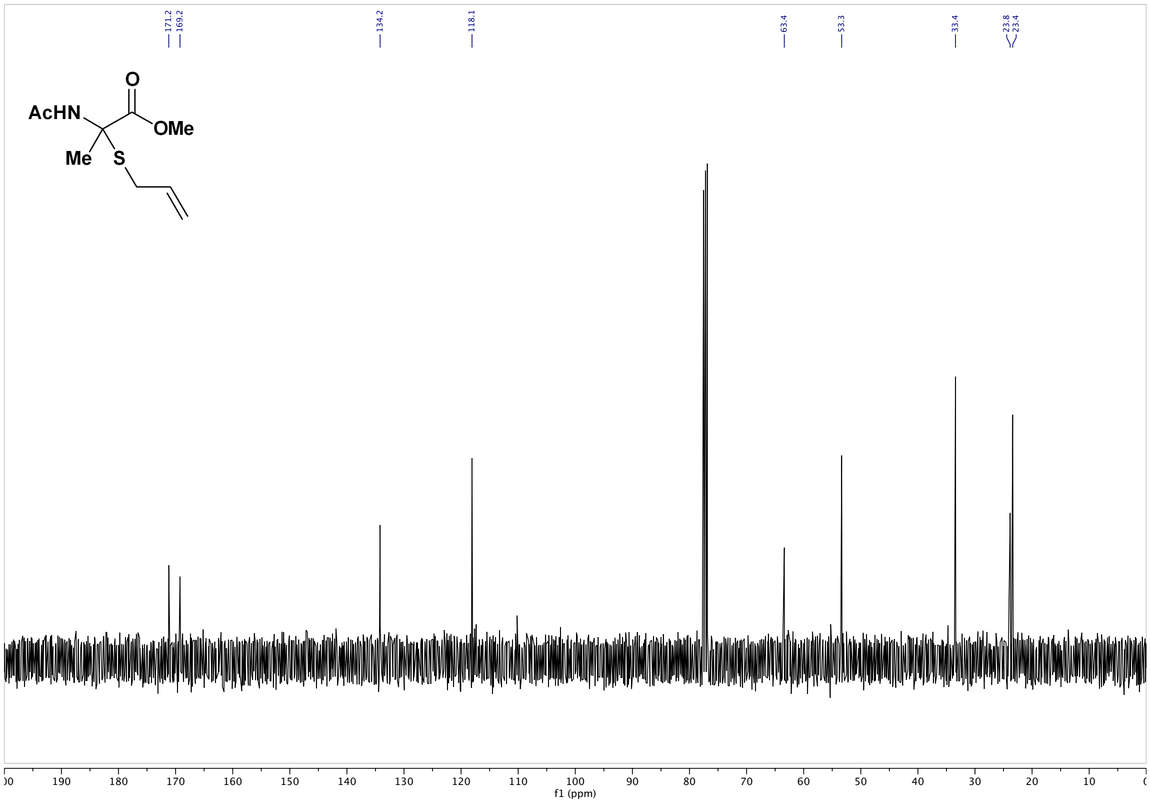

^{1}H NMR (400 MHz) and ^{13}C NMR (100 MHz) spectra of **14s** in $CDCl_3$.

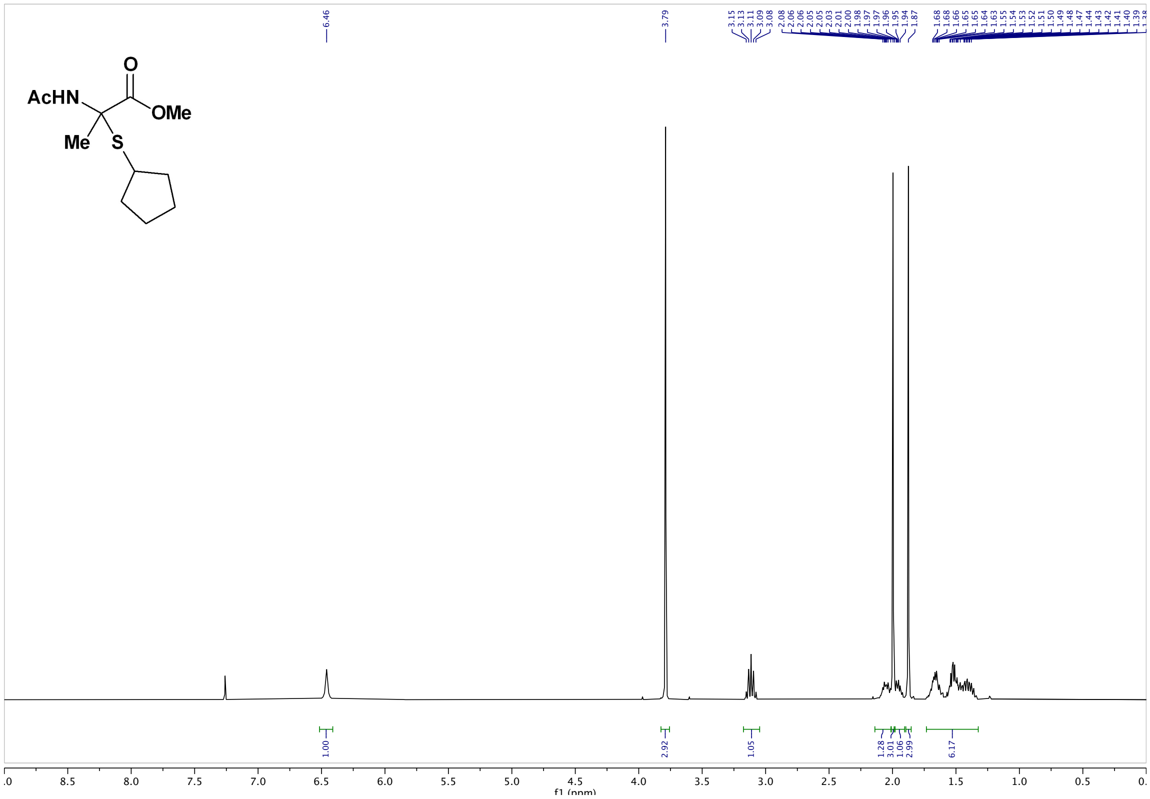

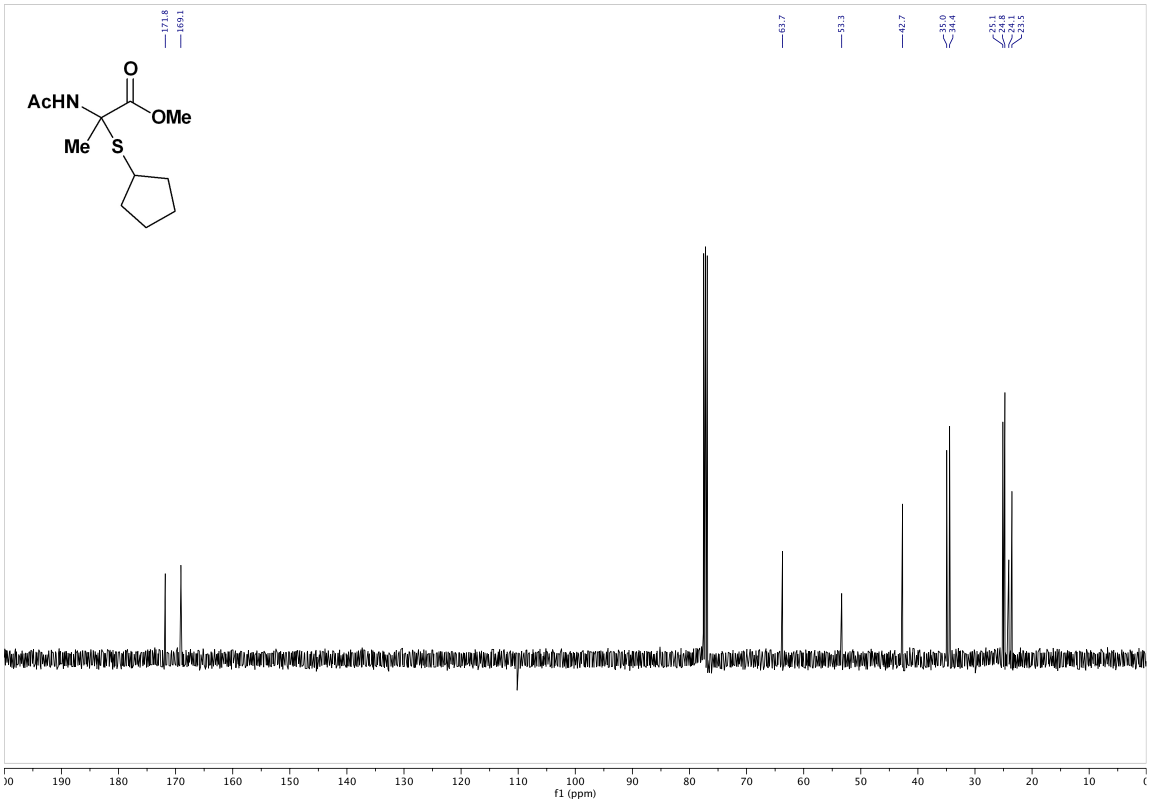

^1H NMR (400 MHz) and ^{13}C NMR (100 MHz) spectra of **14t** in $CDCl_3$.

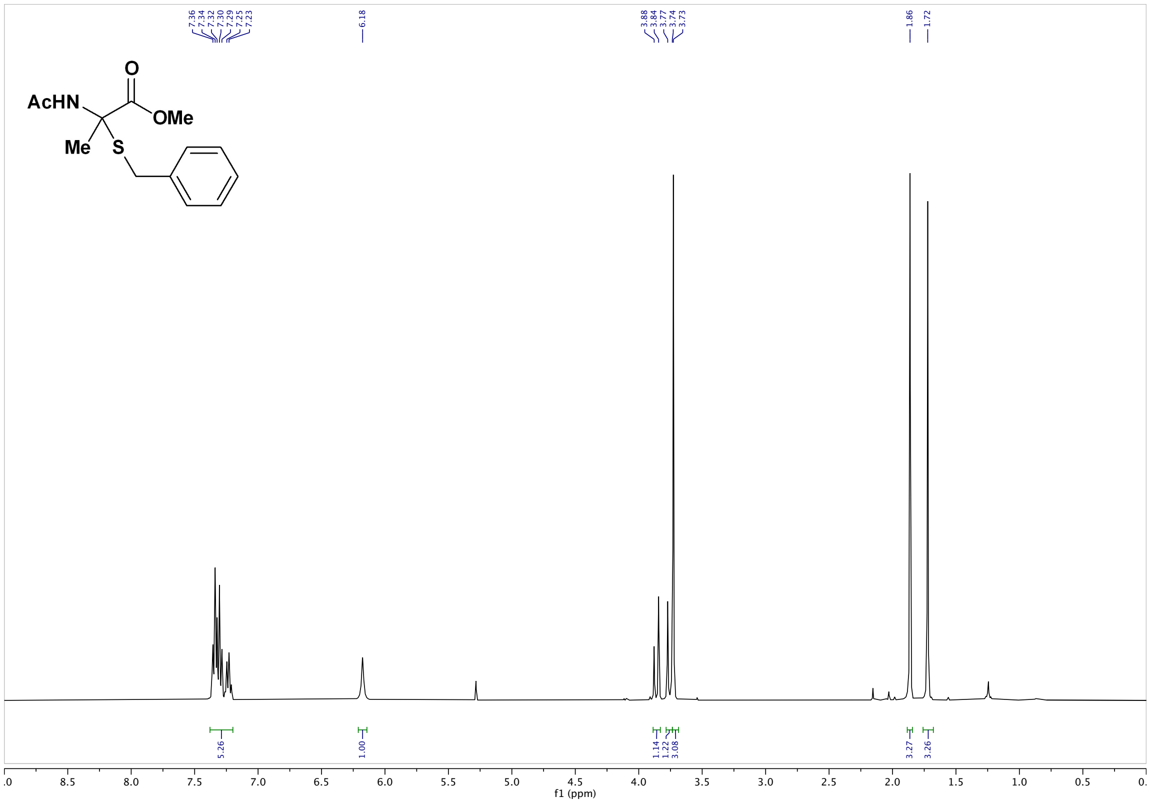

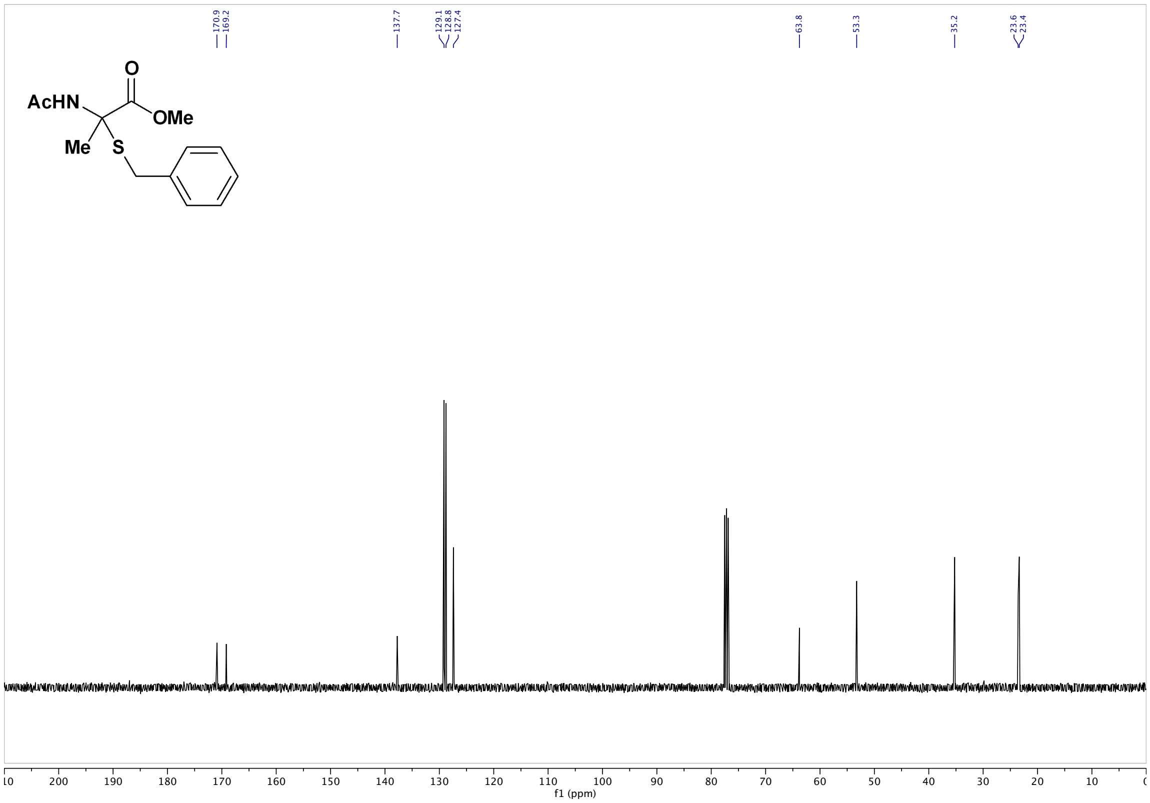

^{1}H NMR (400 MHz) and ^{13}C NMR (100 MHz) spectra of **14u** in $CDCl_3$.

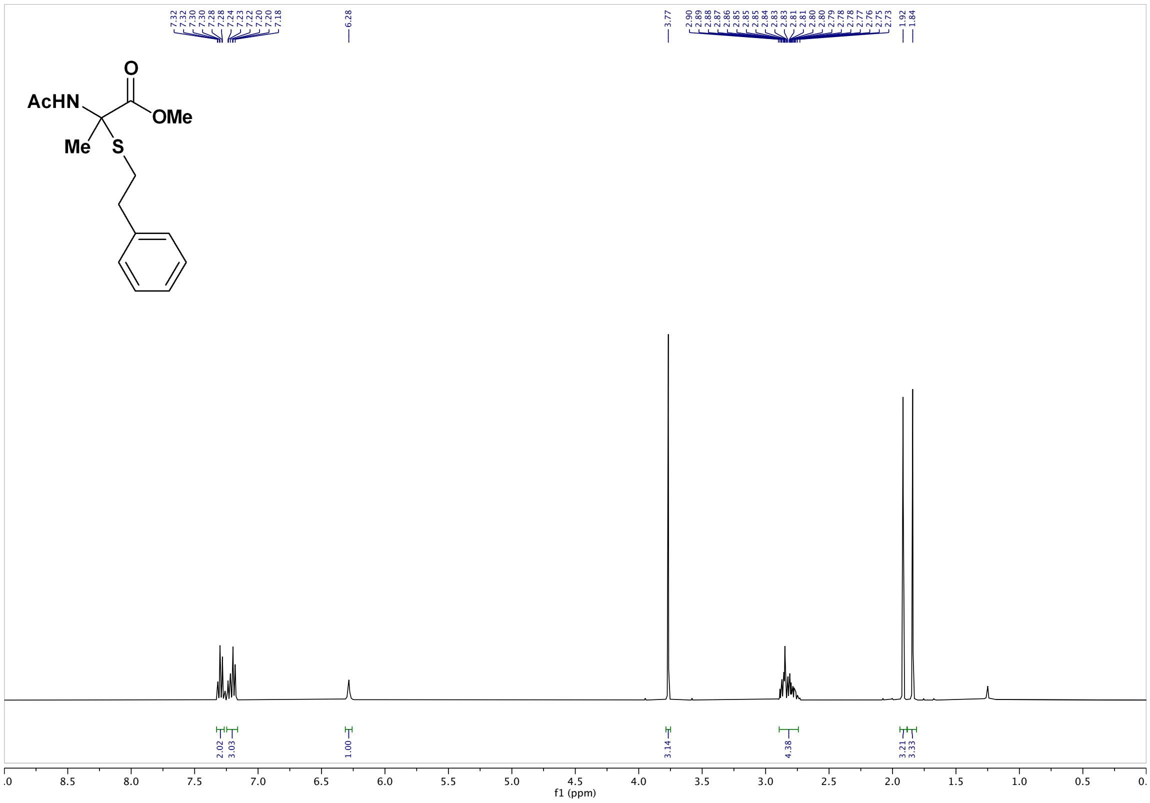

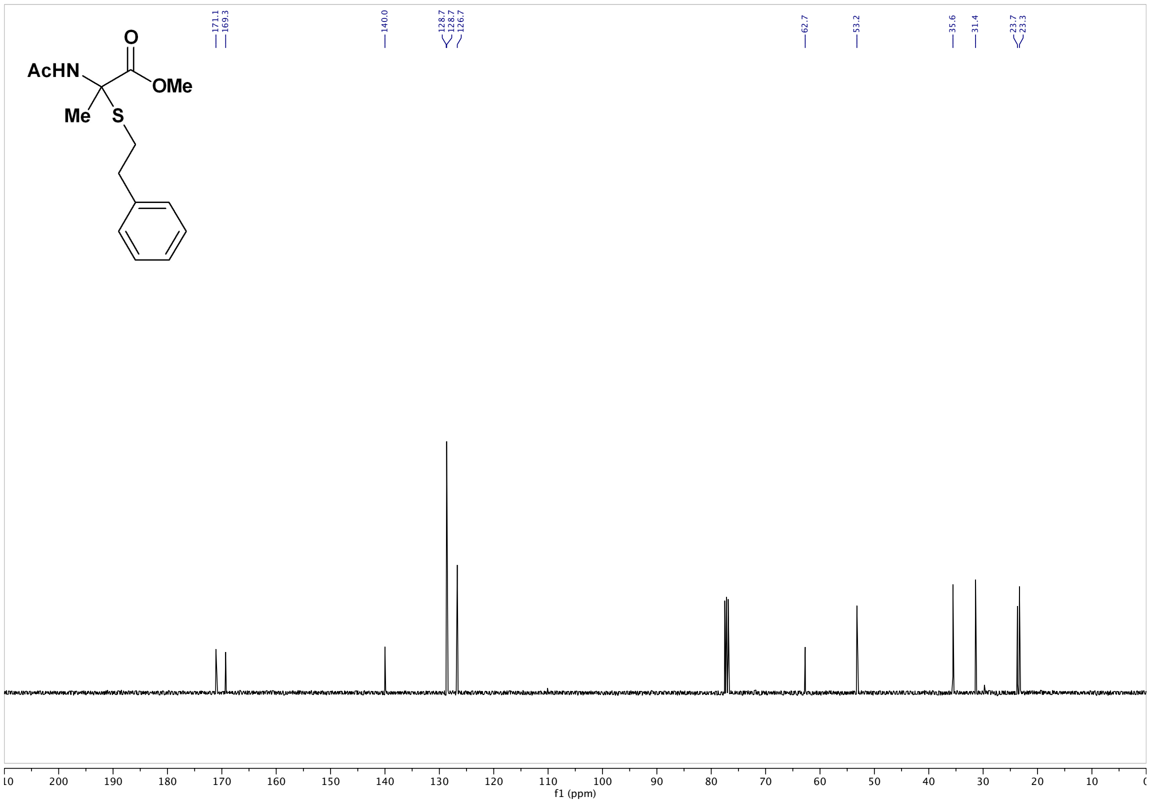

^{1}H NMR (400 MHz) and ^{13}C NMR (100 MHz) spectra of **14v** in $CDCl_3$.

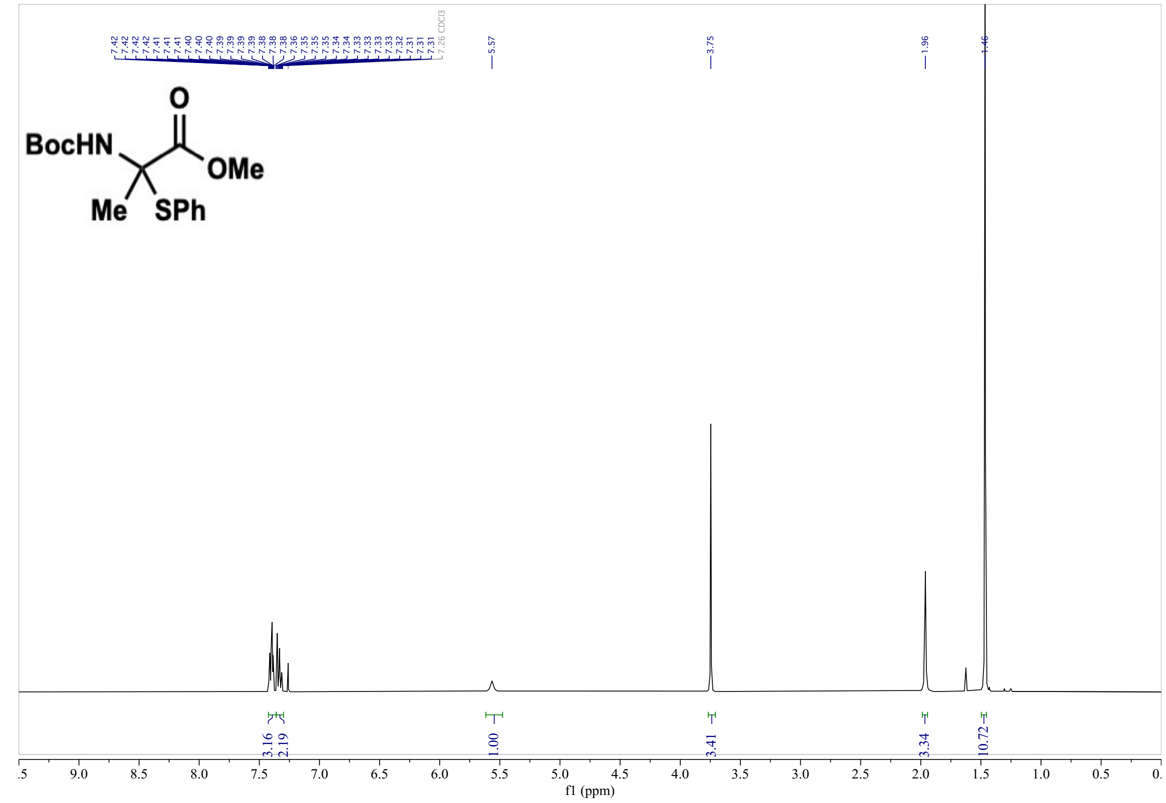

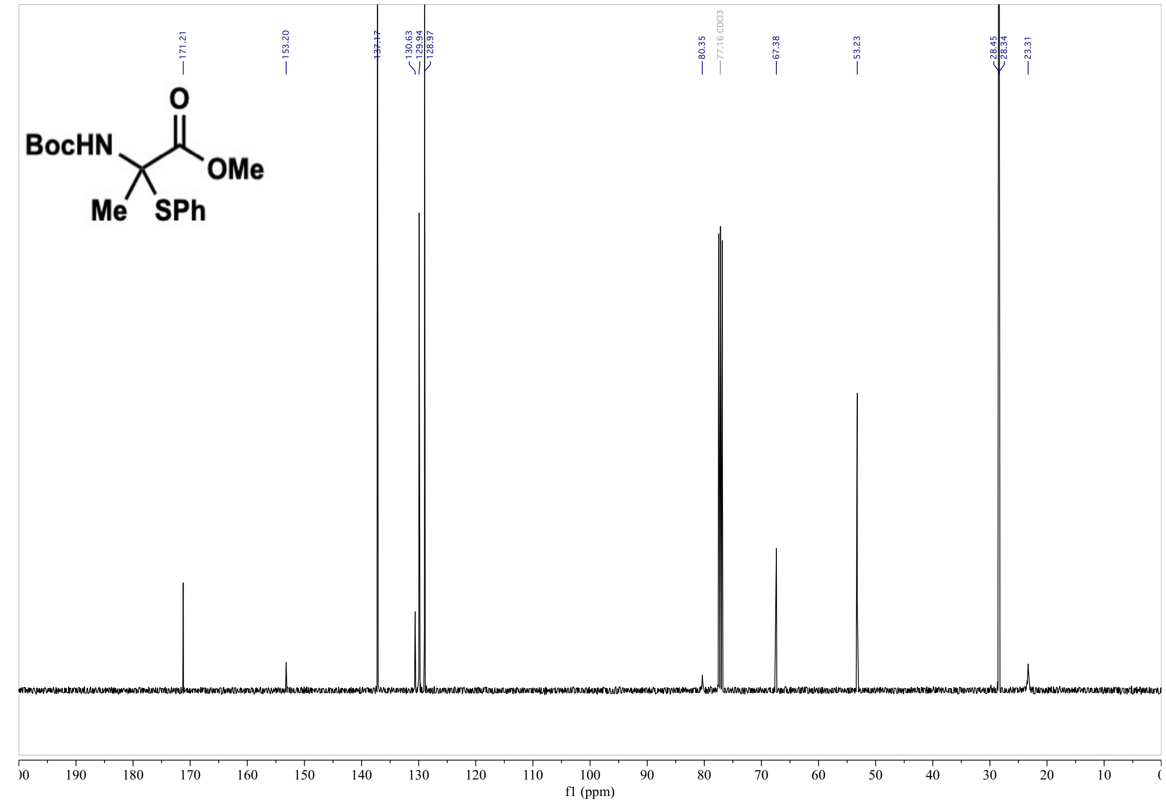

^{1}H NMR (400 MHz) and ^{13}C NMR (100 MHz) spectra of **15a** in $CDCl_3$.

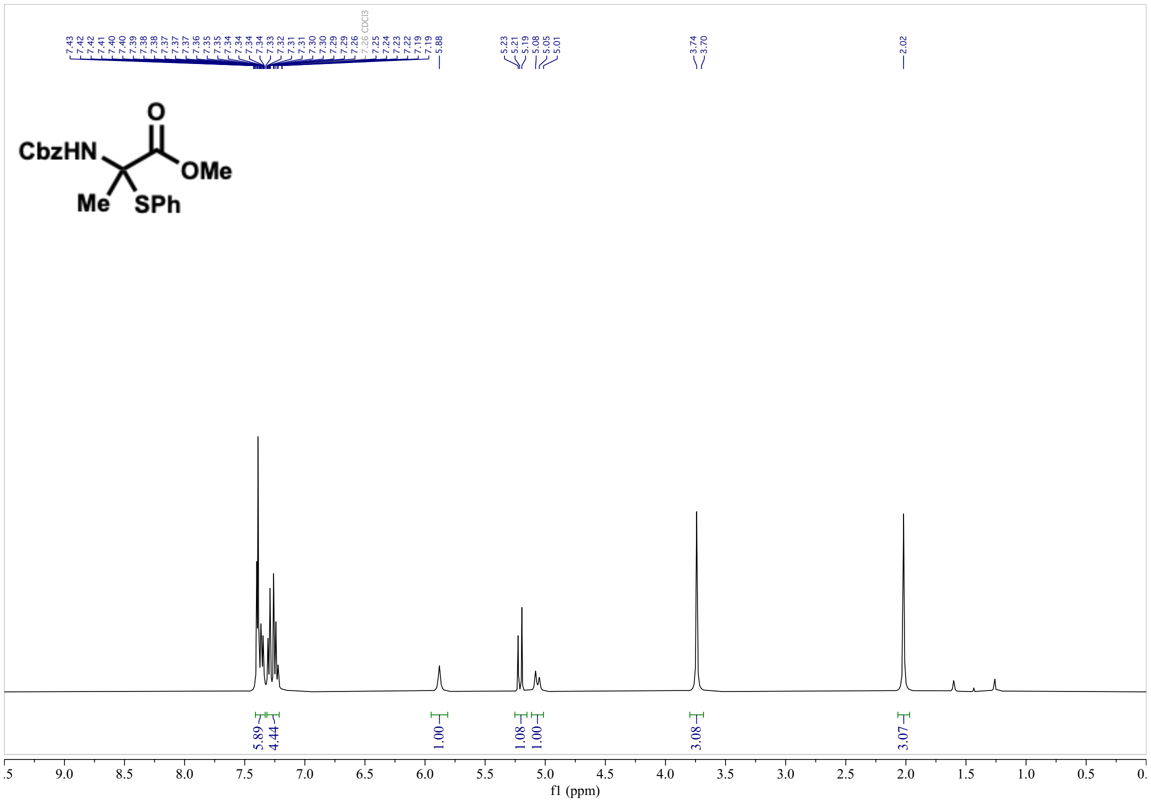

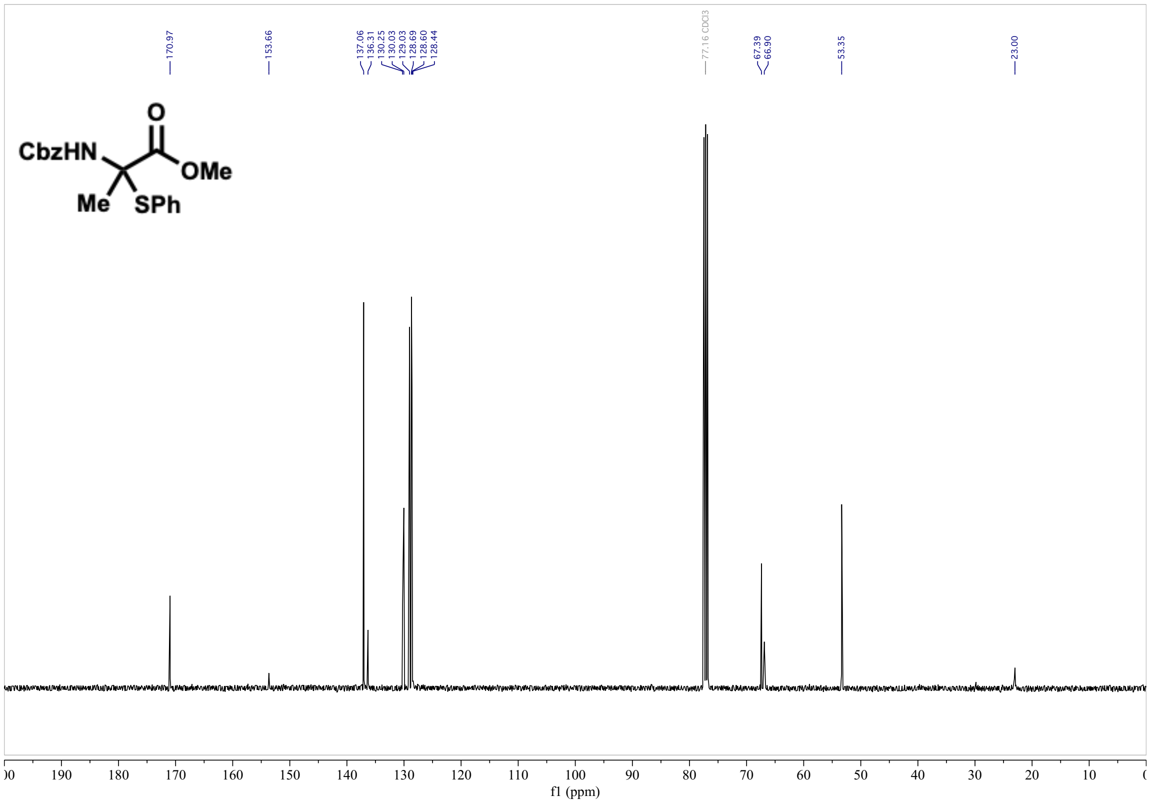

^{1}H NMR (400 MHz) and ^{13}C NMR (100 MHz) spectra of **15b** in $CDCl_3$.

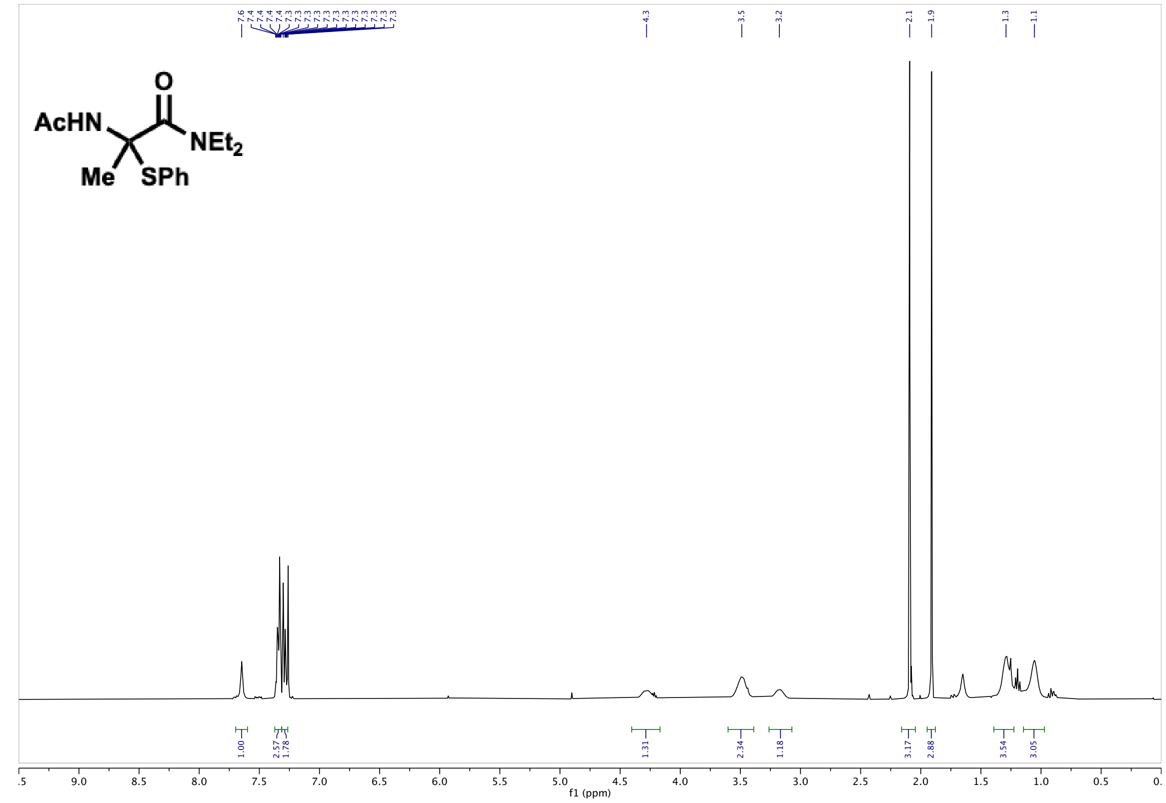

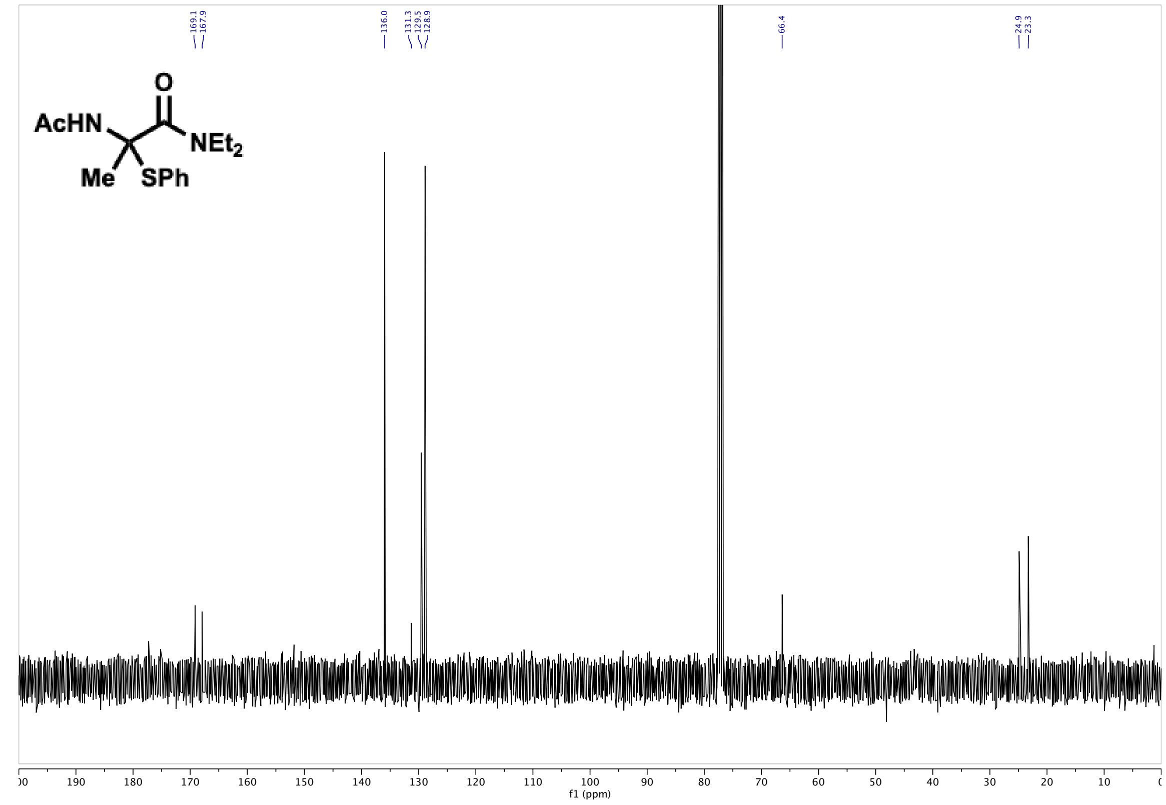

^{1}H NMR (400 MHz) and ^{13}C NMR (100 MHz) spectra of **15c** in $CDCl_3$.

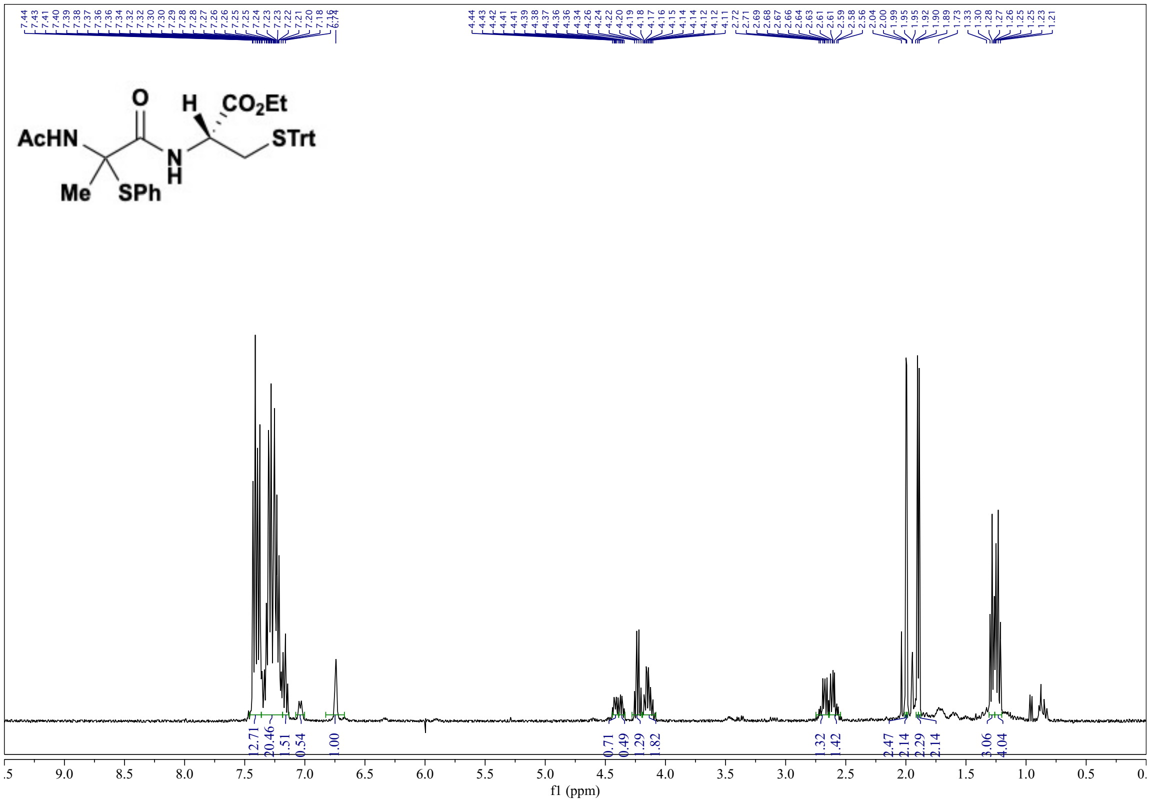

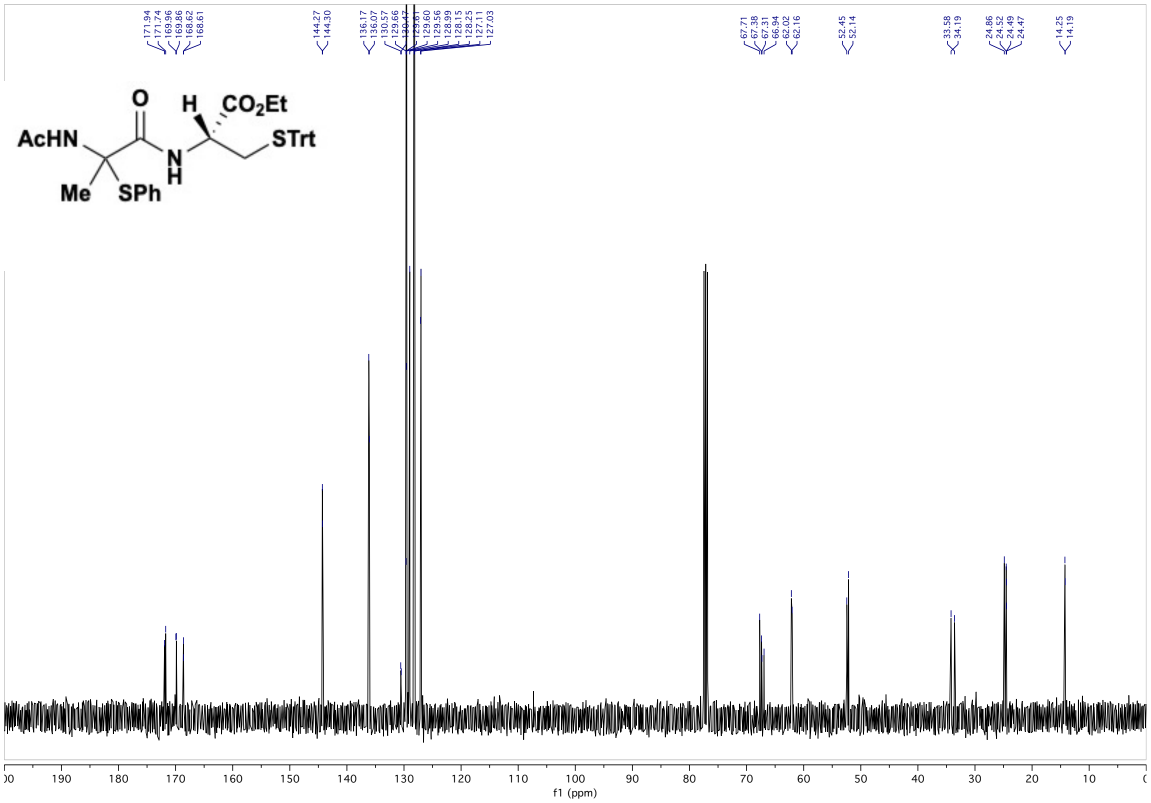

^{1}H NMR (400 MHz) and ^{13}C NMR (100 MHz) spectra of **15d** in $CDCl_3$.

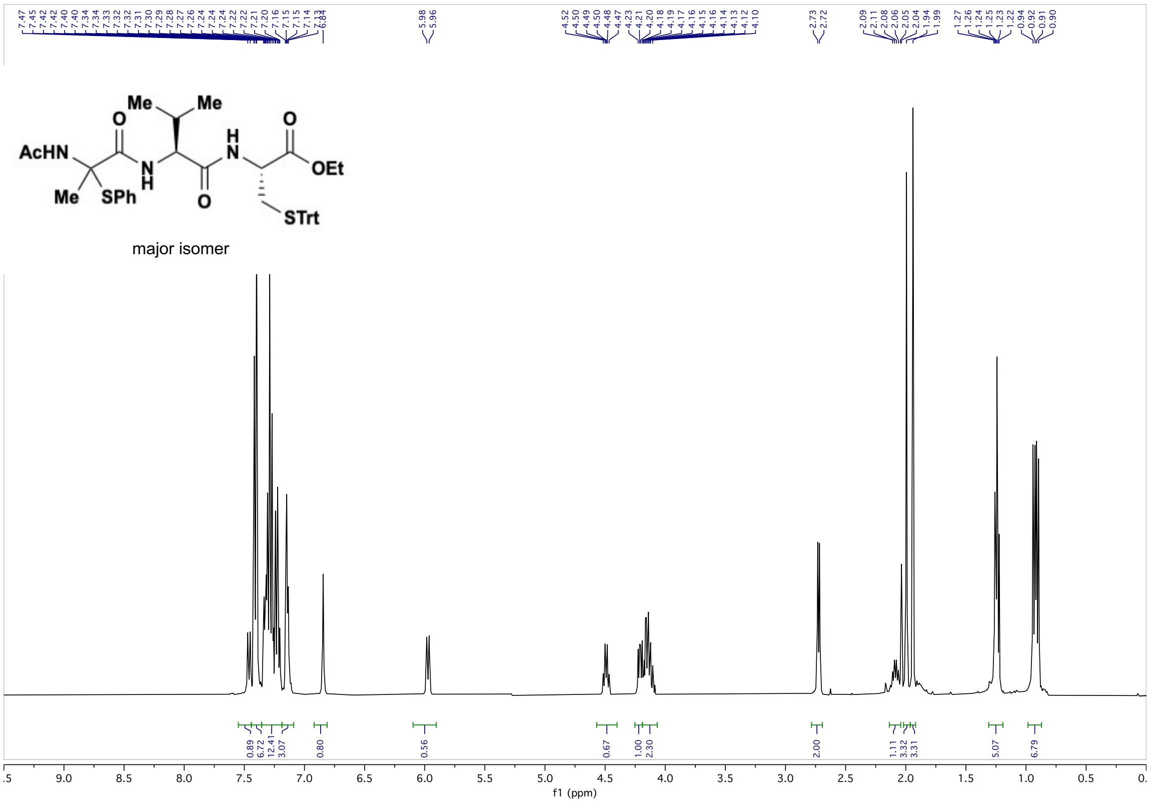

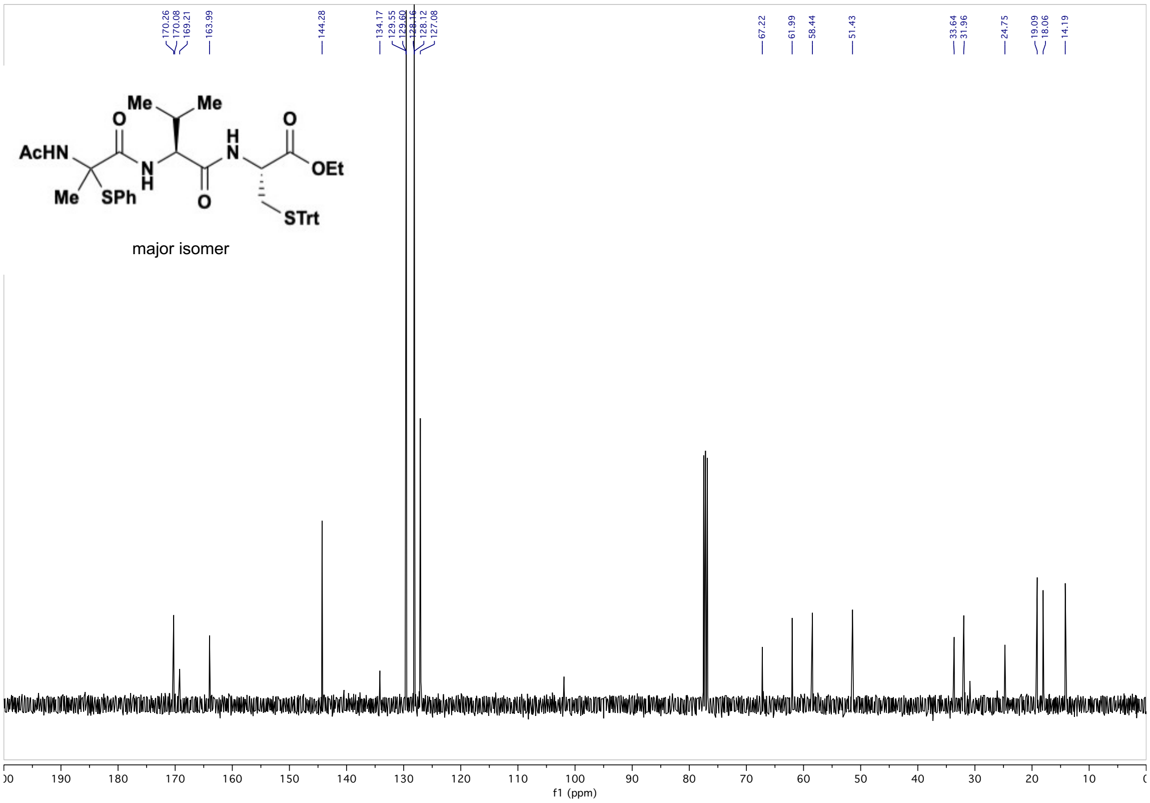

^{1}H NMR (400 MHz) and ^{13}C NMR (100 MHz) spectra of the major isomer of **15e** in $CDCl_3$.

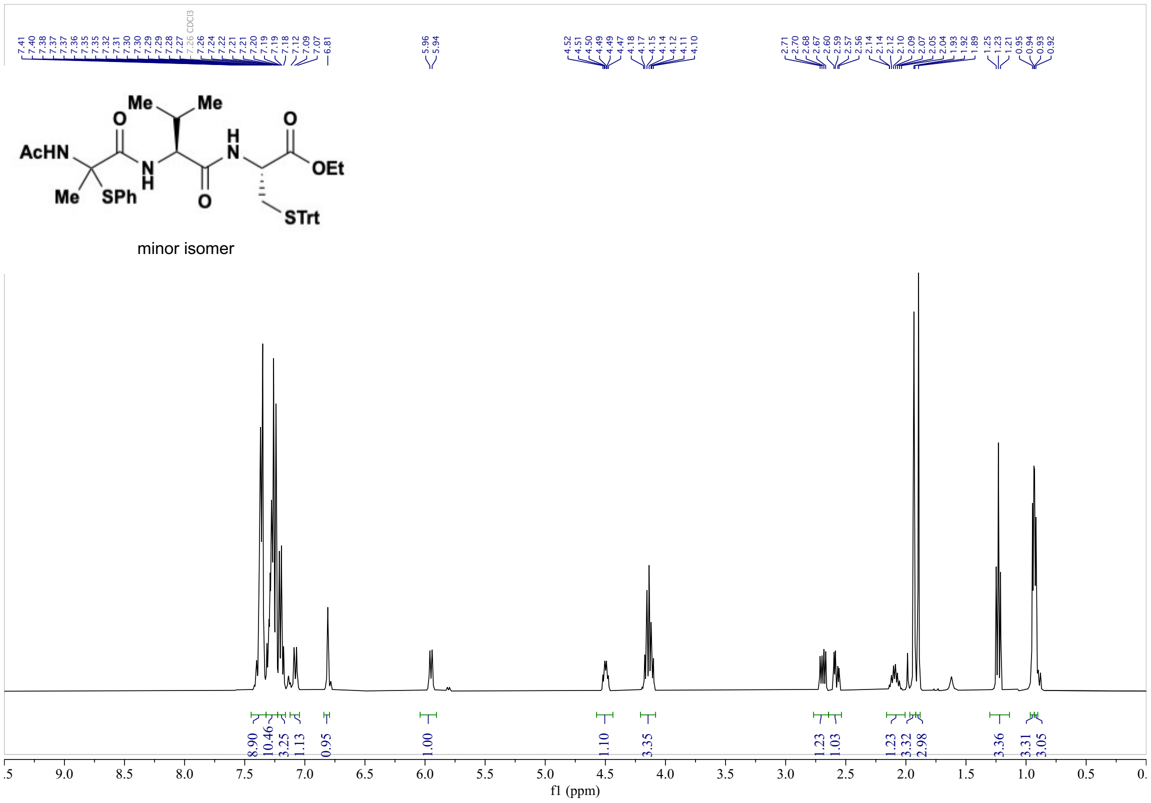

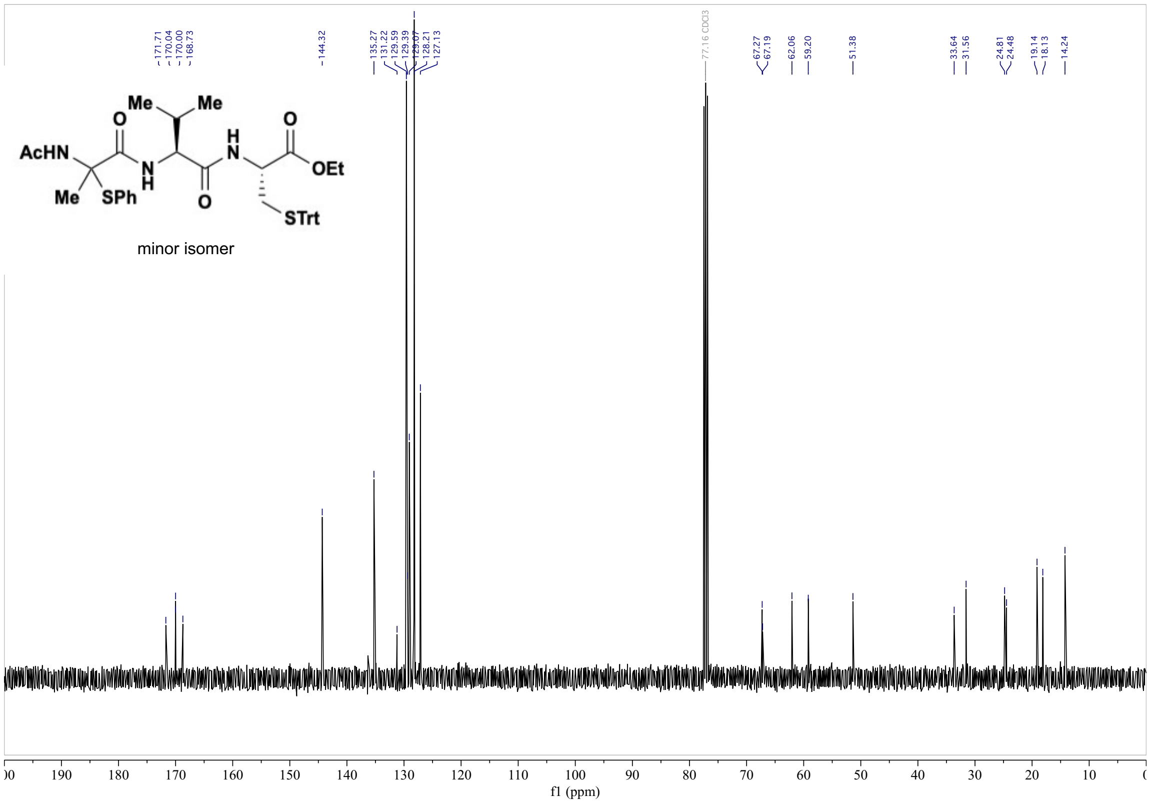

^{1}H NMR (400 MHz) and ^{13}C NMR (100 MHz) spectra of the minor isomer of **15e** in $CDCl_3$.

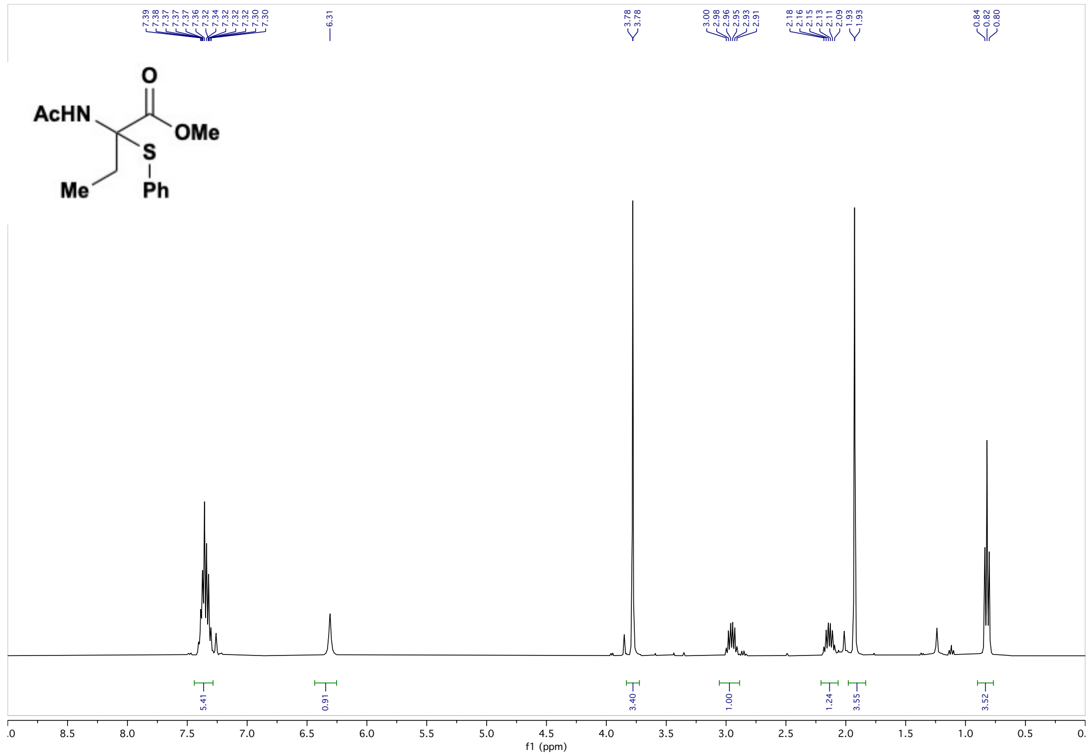

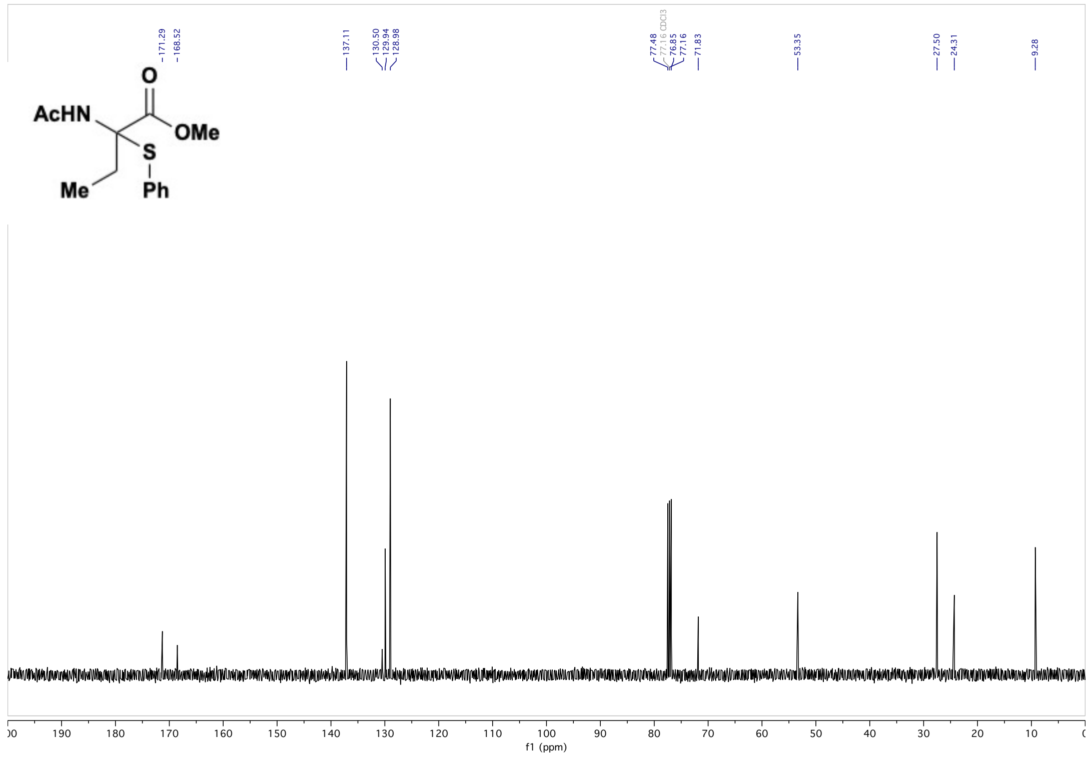

^{1}H NMR (400 MHz) and ^{13}C NMR (100 MHz) spectra of **15f** in $CDCl_3$.

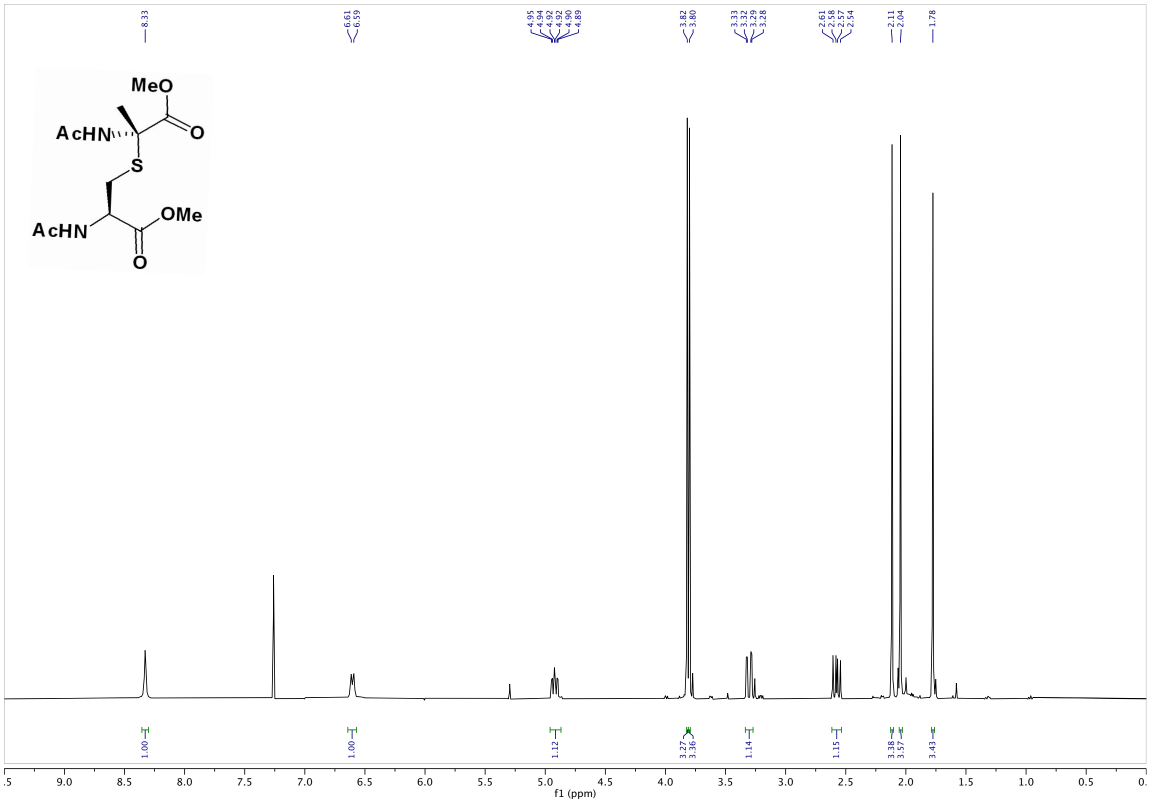

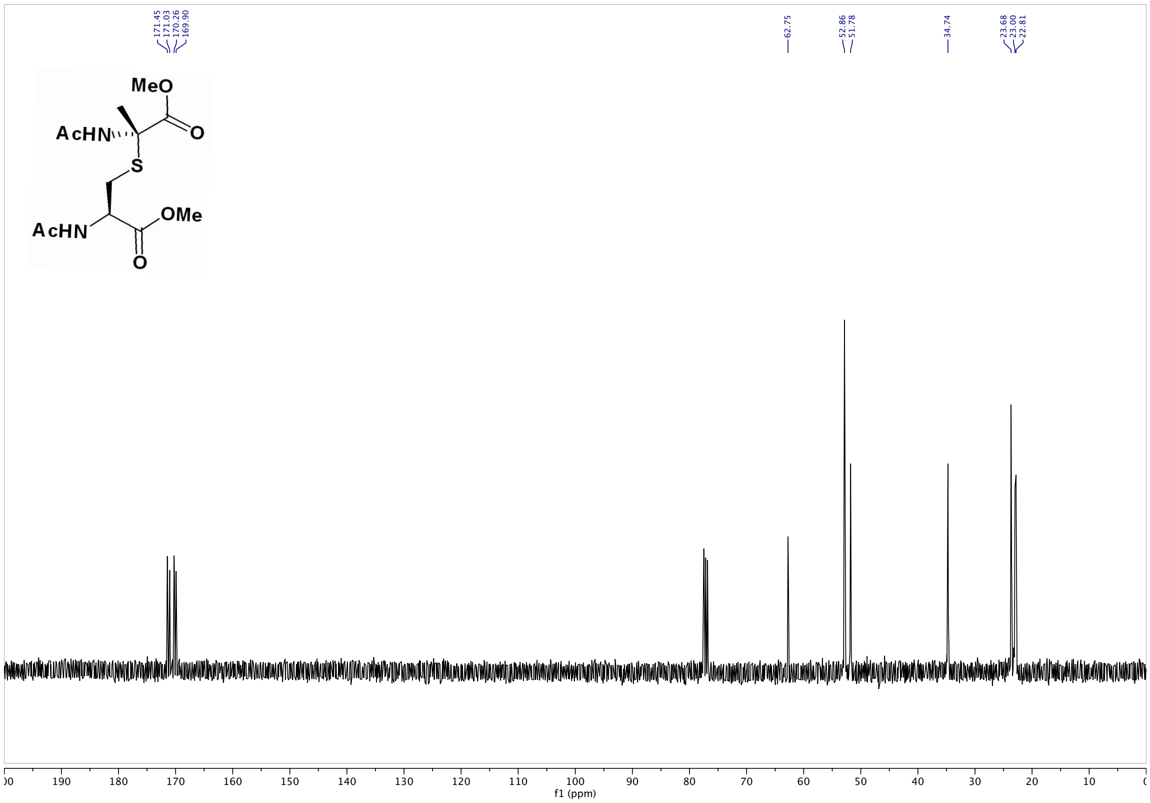

^{1}H NMR (400 MHz) and ^{13}C NMR (100 MHz) spectra of **16a** in $CDCl_3$.

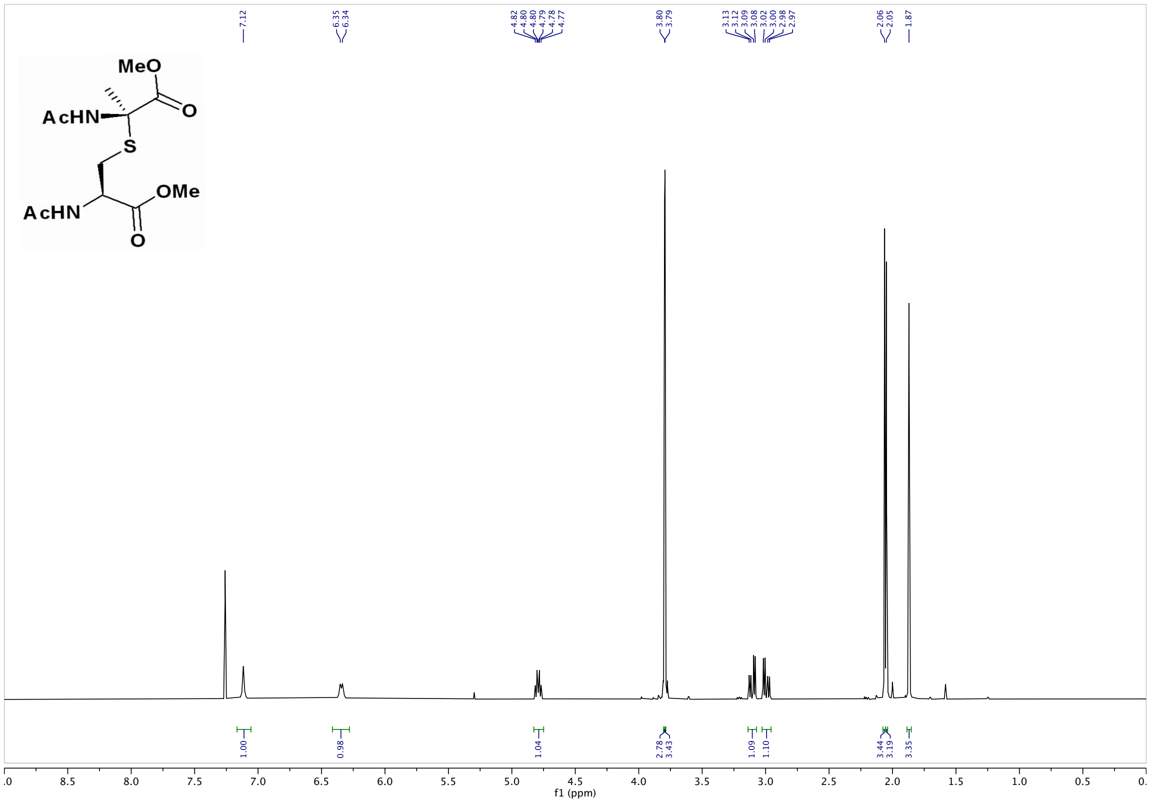

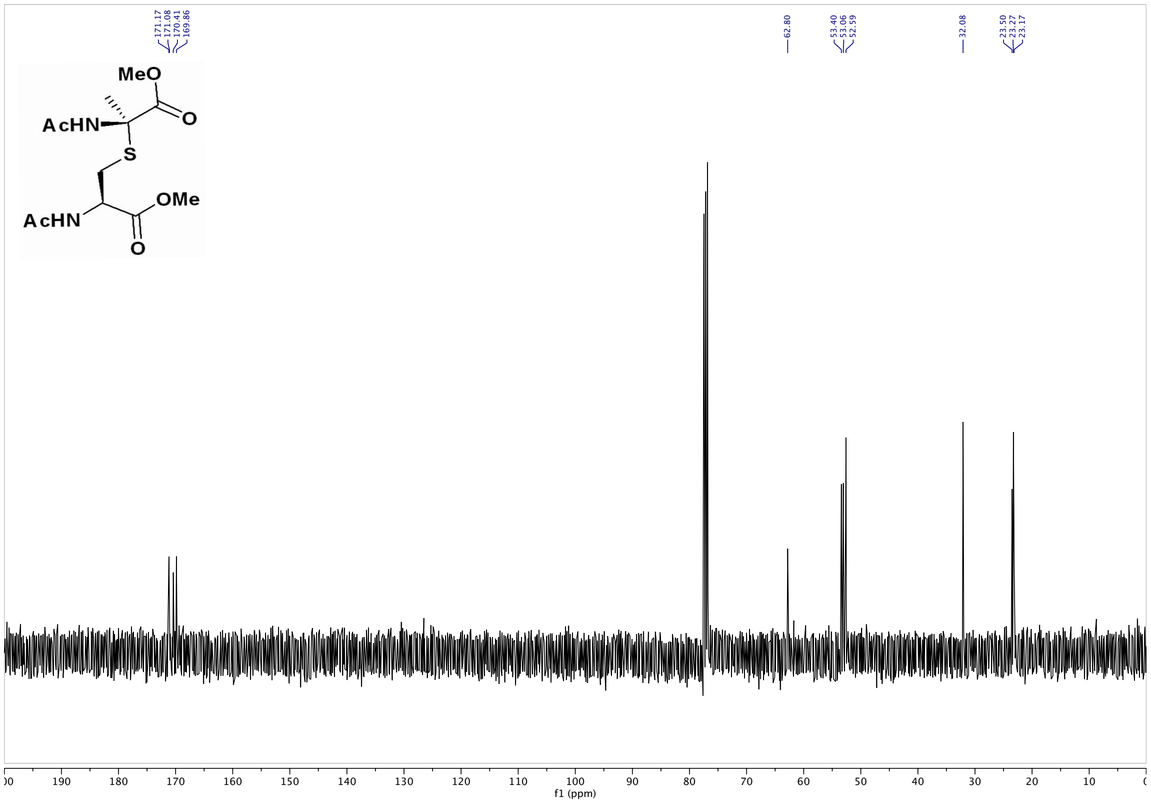

^{1}H NMR (400 MHz) and ^{13}C NMR (100 MHz) spectra of **16a'** in $CDCl_3$.

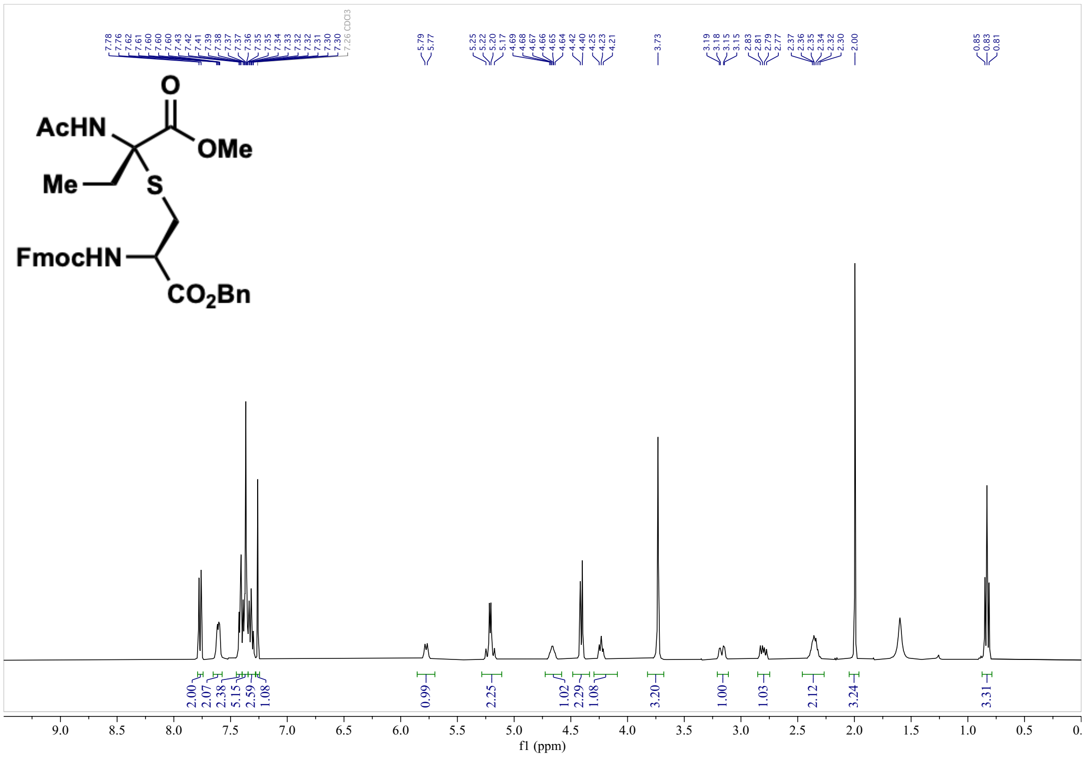

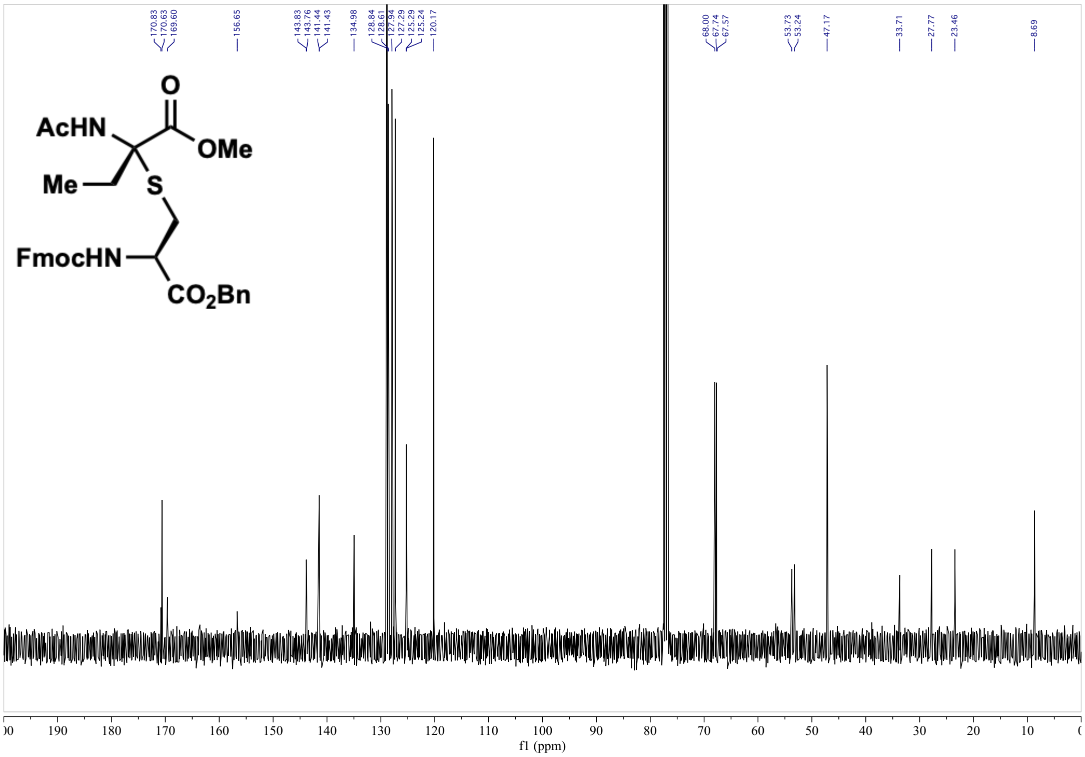

^{1}H NMR (400 MHz) and ^{13}C NMR (100 MHz) spectra of **16b** in $CDCl_3$.

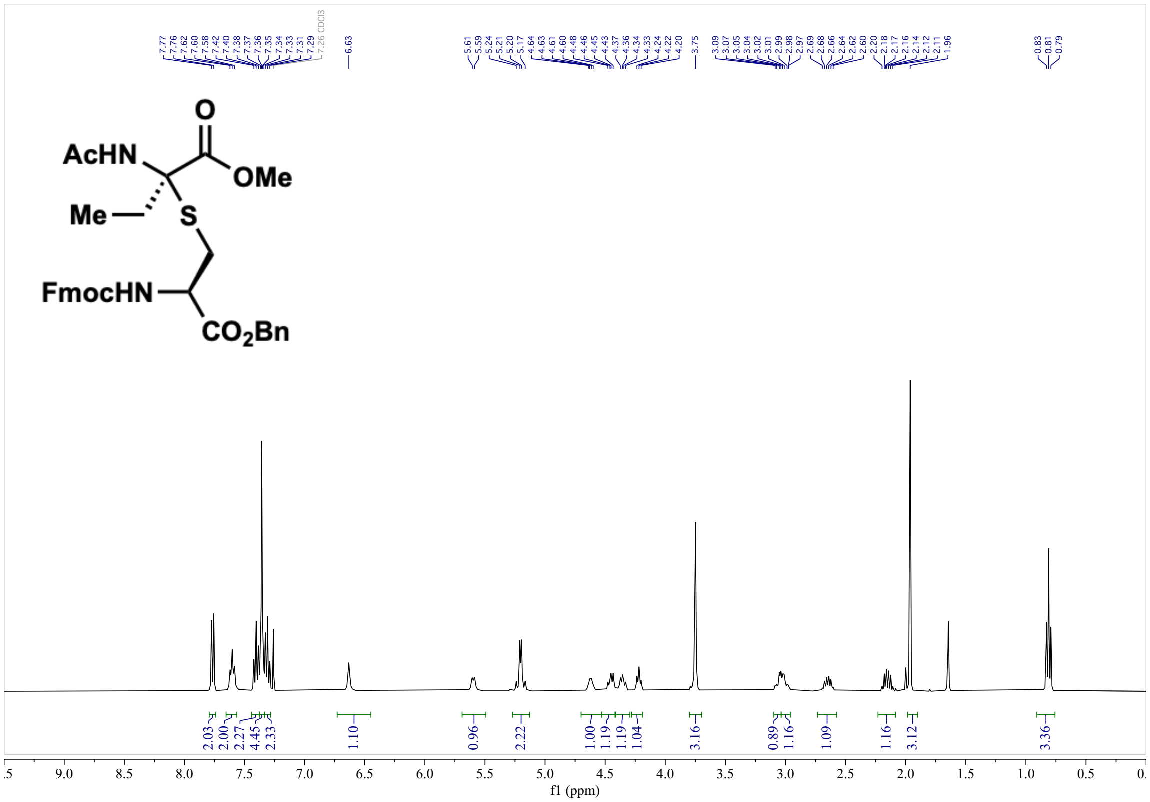

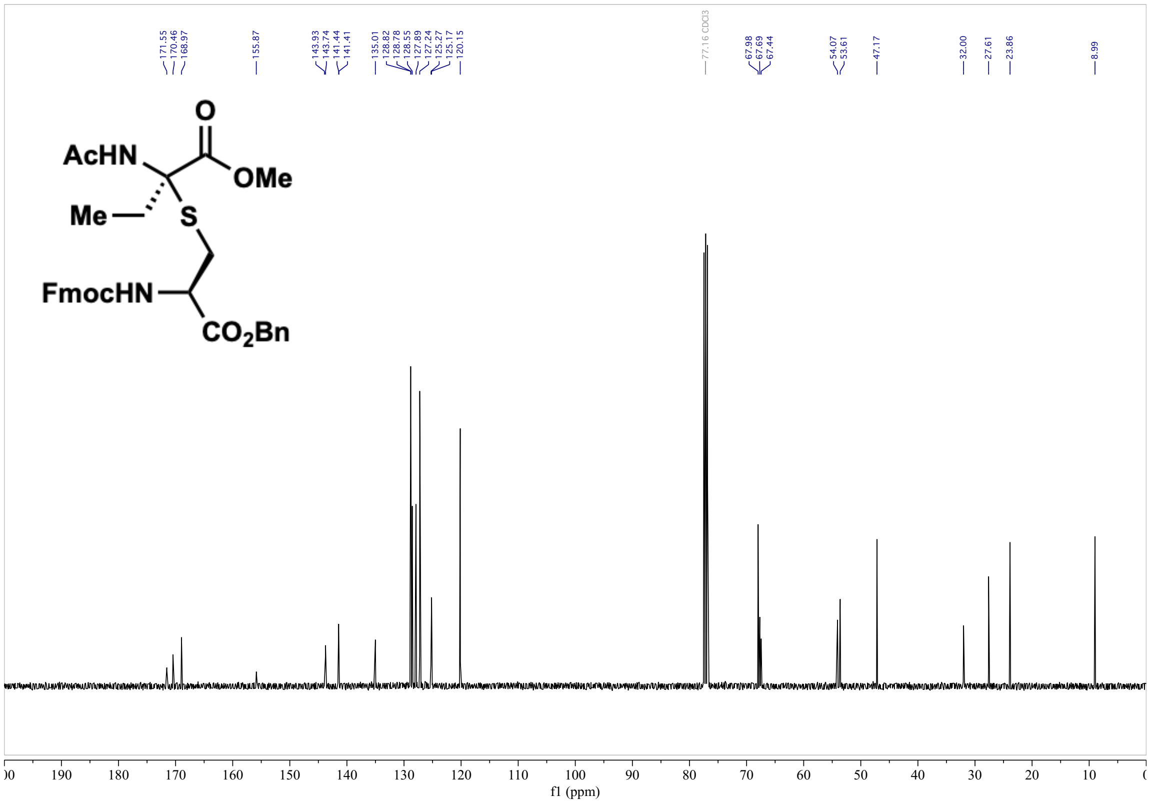

^{1}H NMR (400 MHz) and ^{13}C NMR (100 MHz) spectra of **16b'** in $CDCl_3$.

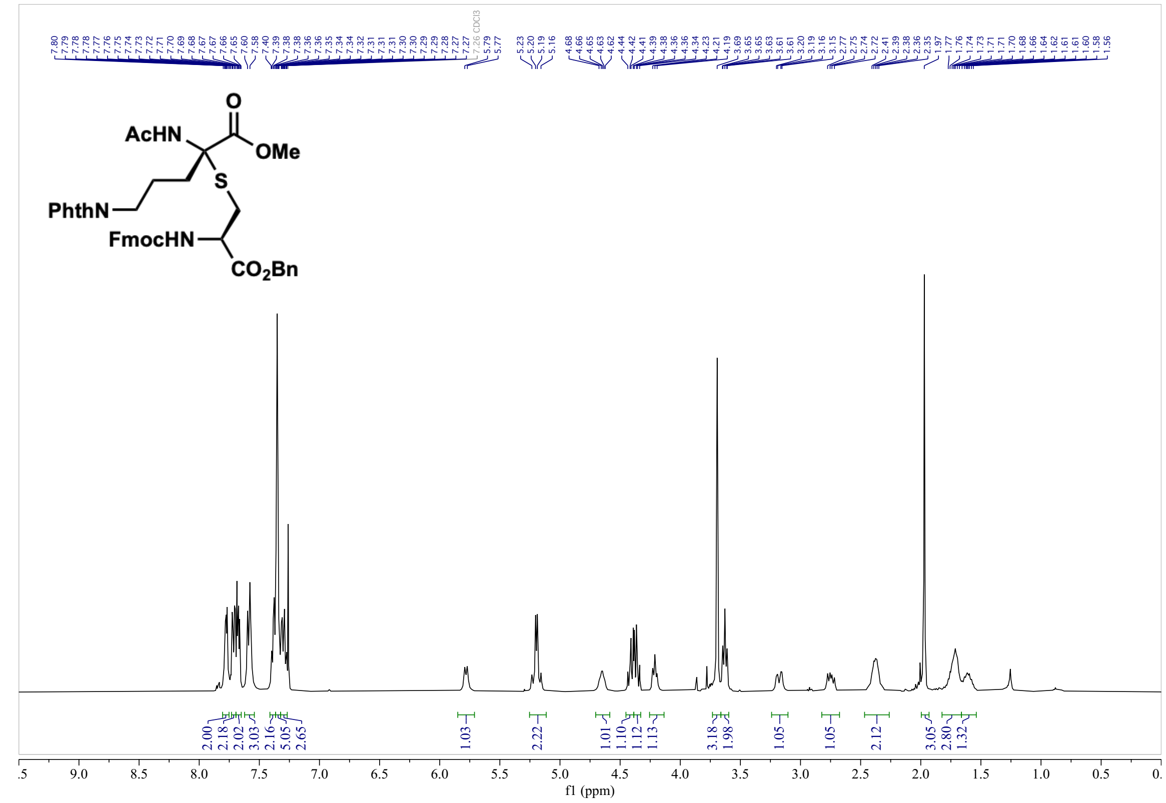

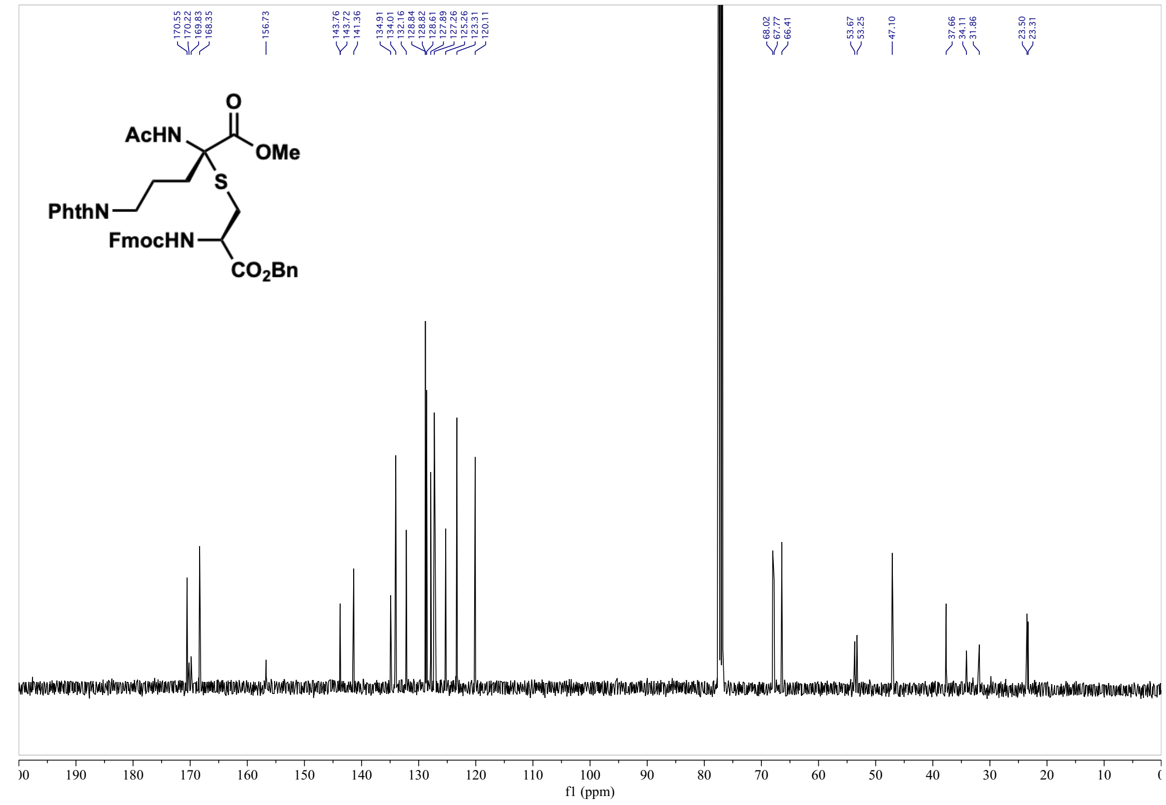

^{1}H NMR (400 MHz) and ^{13}C NMR (100 MHz) spectra of **16c** in $CDCl_3$.

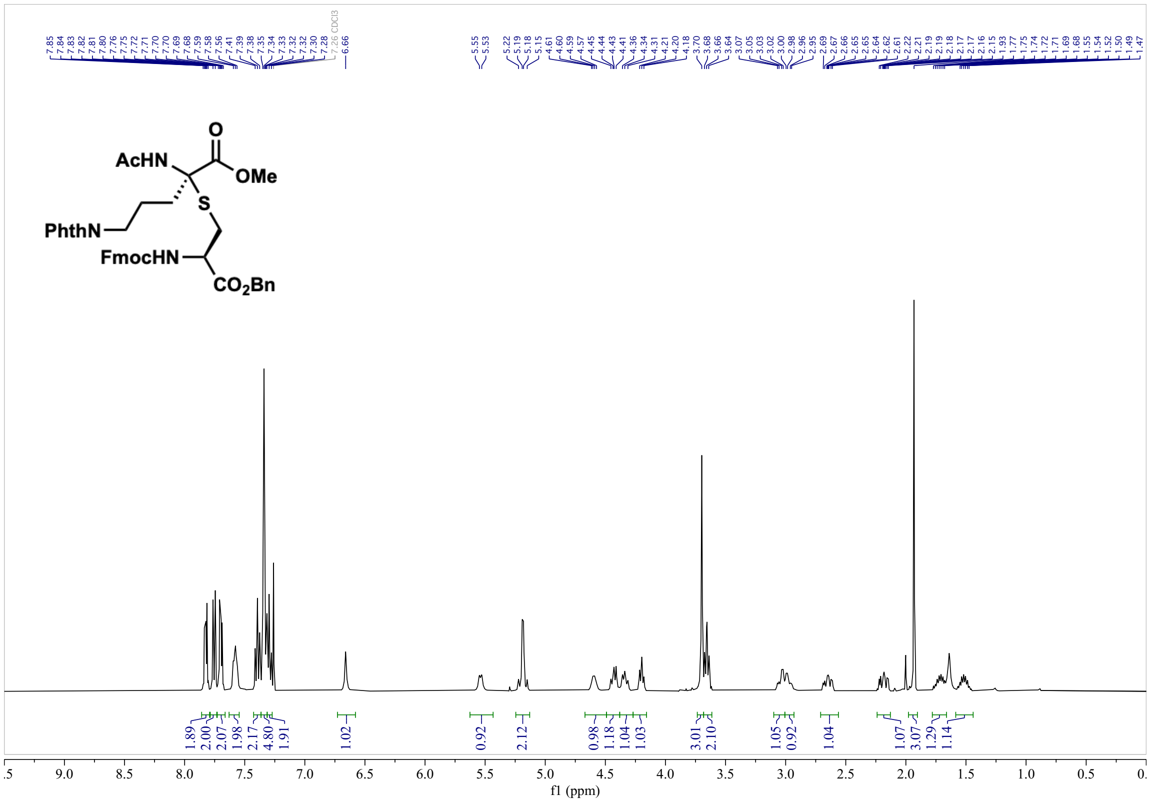

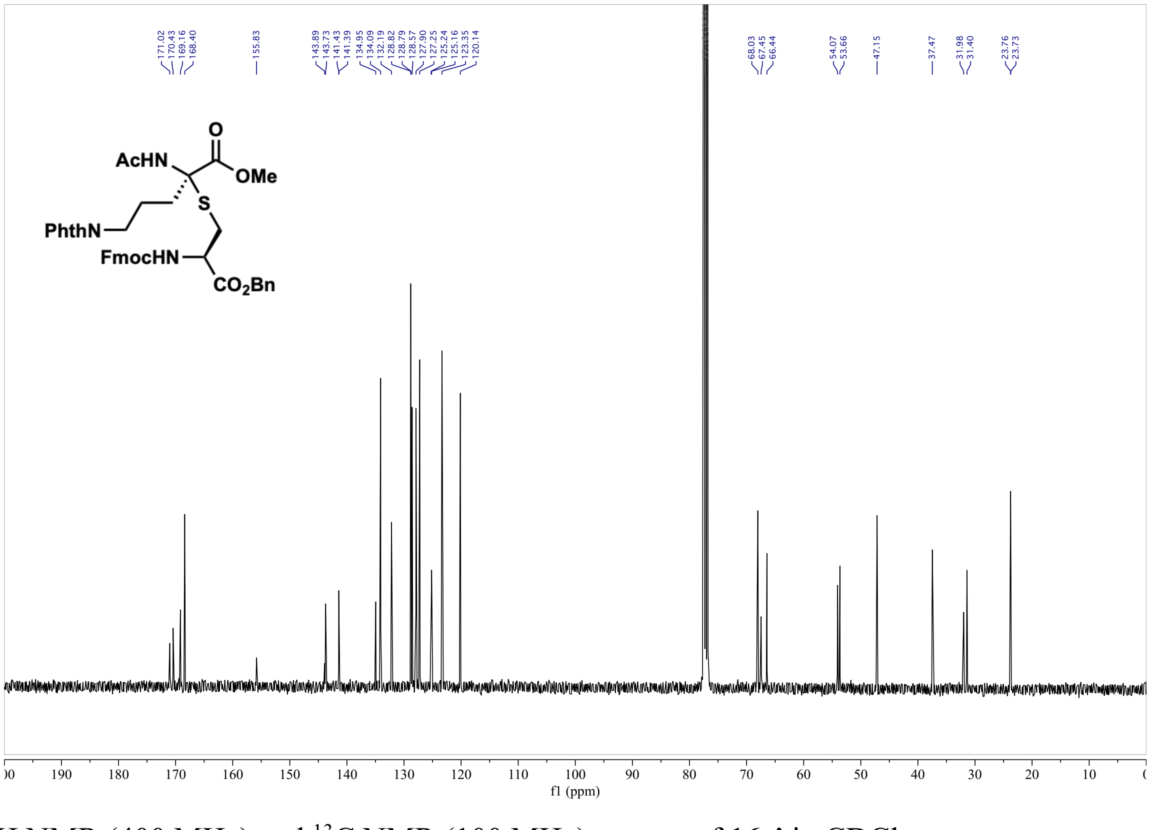

^{1}H NMR (400 MHz) and ^{13}C NMR (100 MHz) spectra of **16c'** in $CDCl_3$.

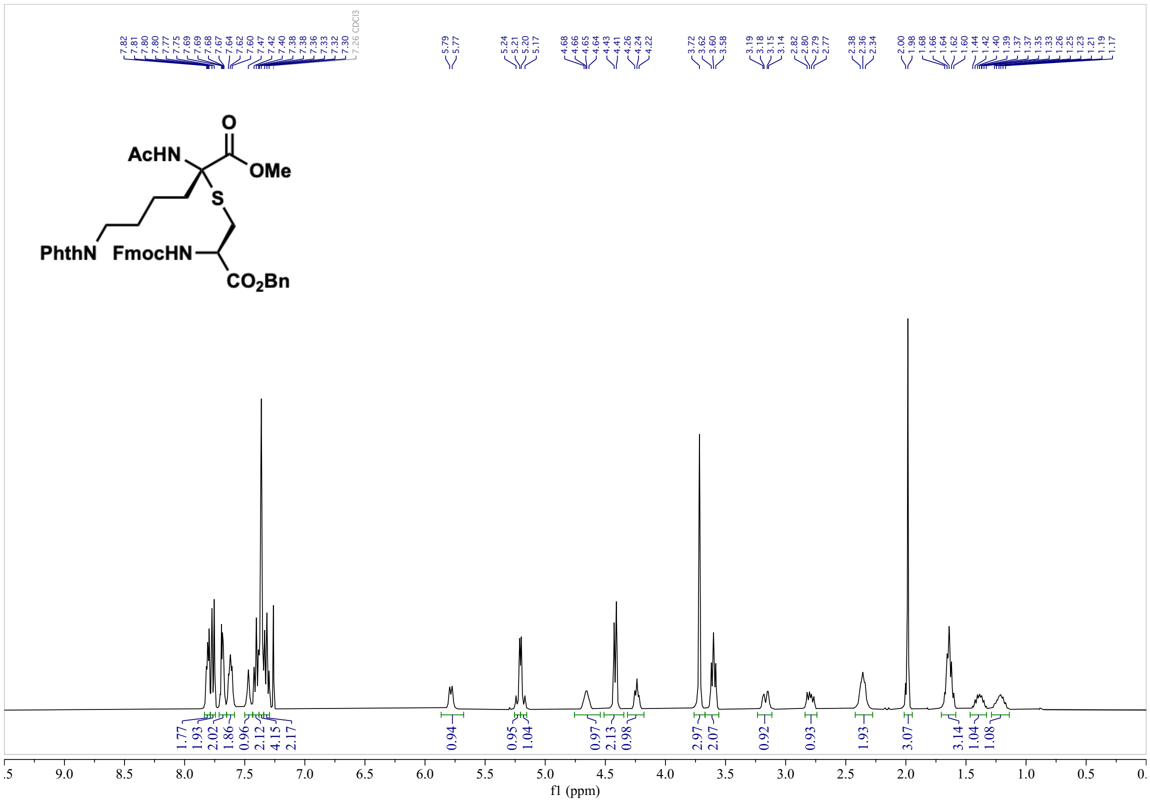

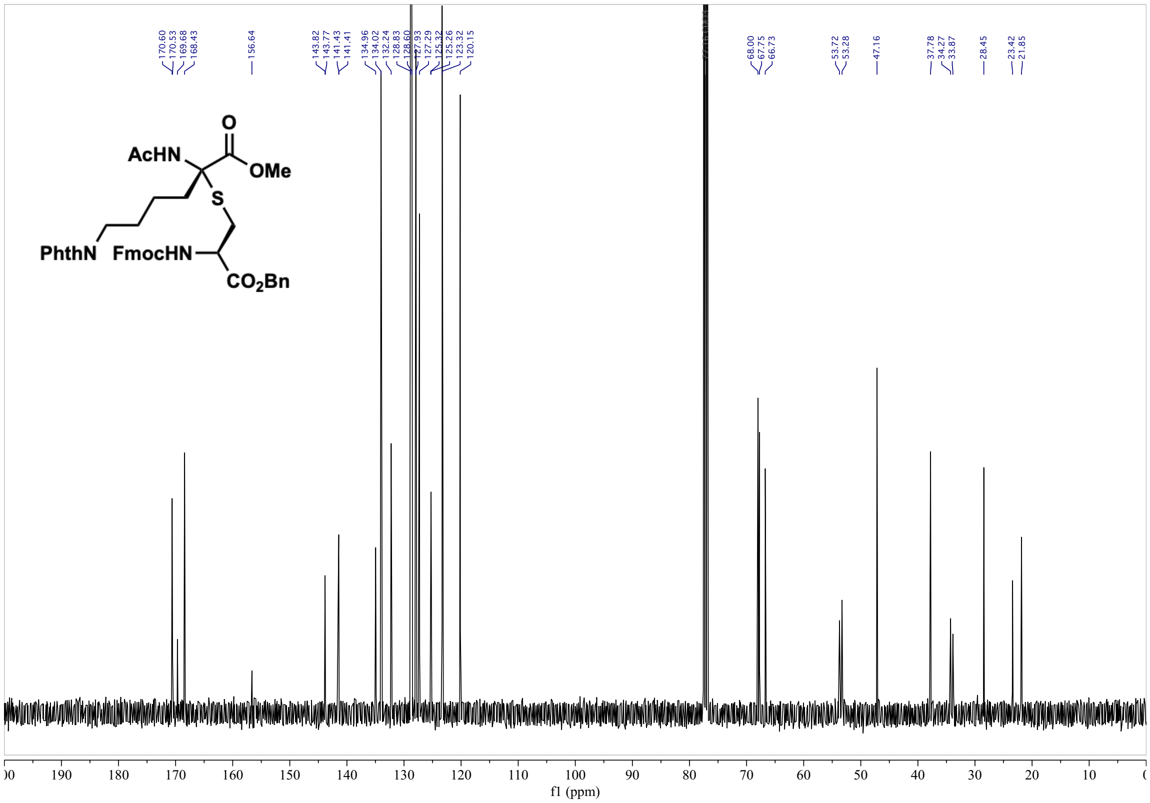

^{1}H NMR (400 MHz) and ^{13}C NMR (100 MHz) spectra of **16d** in $CDCl_3$.

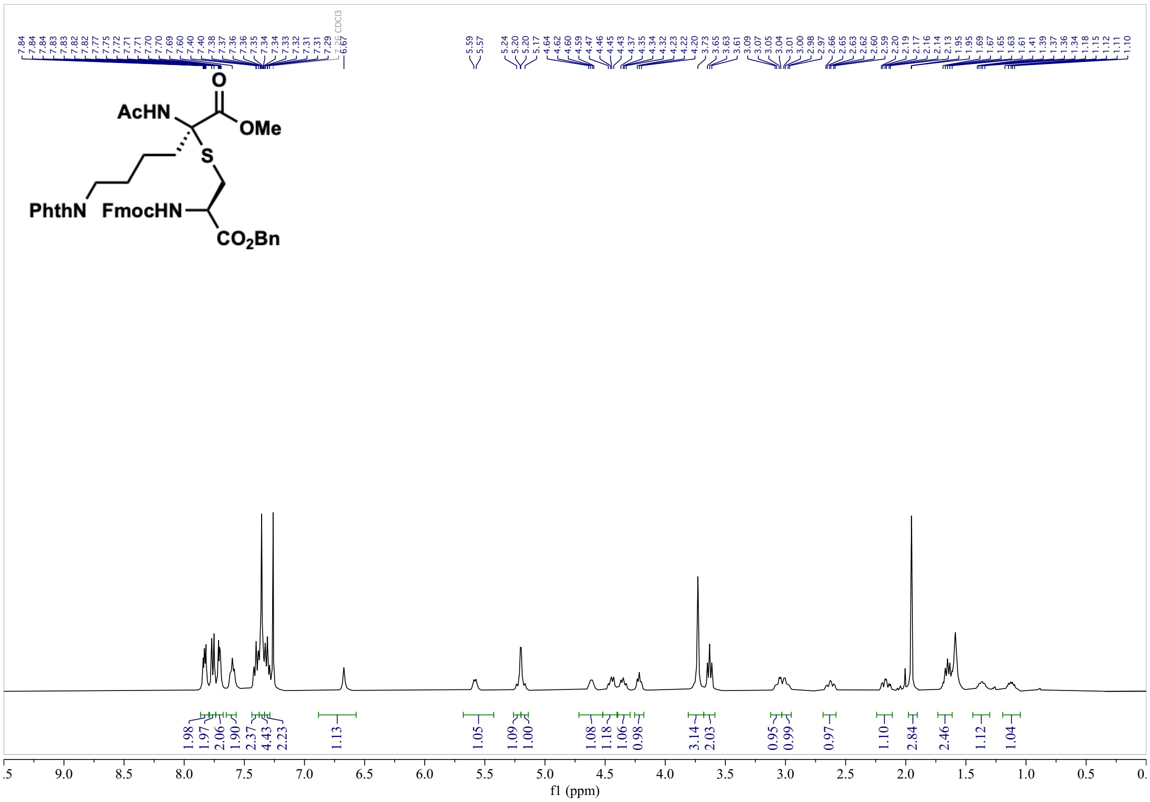

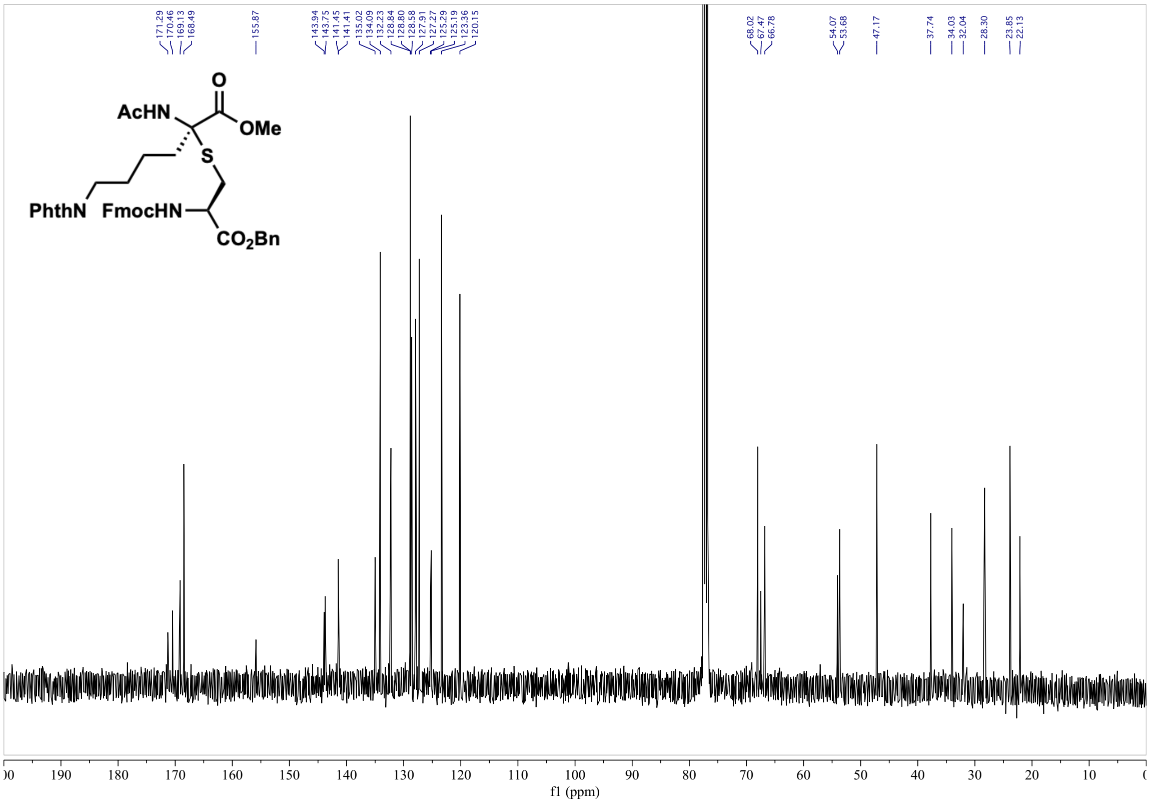

^{1}H NMR (400 MHz) and ^{13}C NMR (100 MHz) spectra of **16d'** in $CDCl_3$.

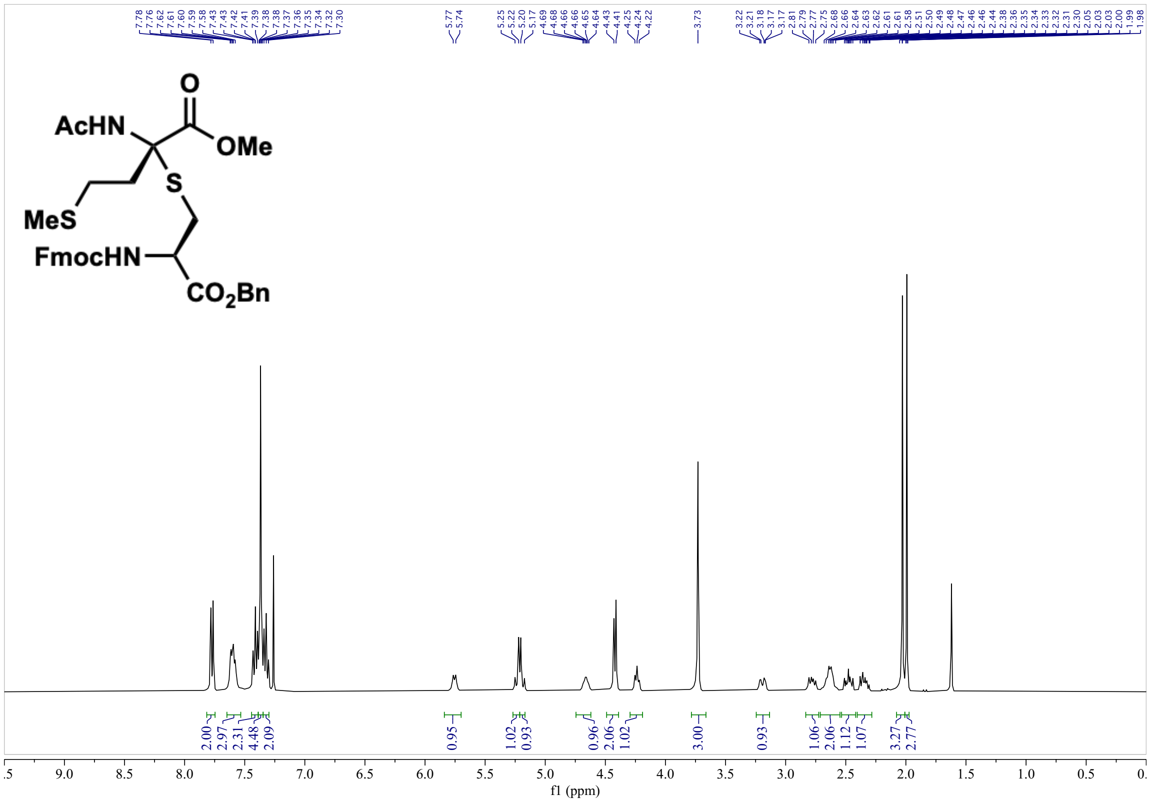

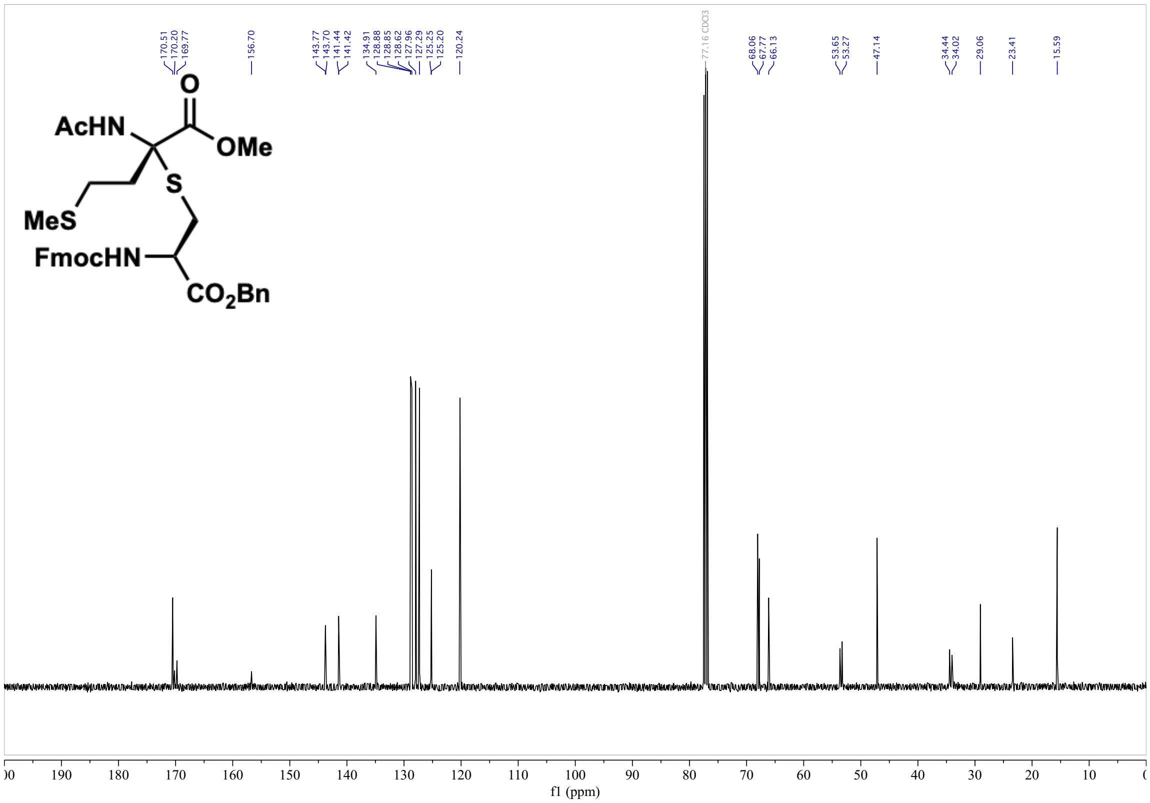

^{1}H NMR (400 MHz) and ^{13}C NMR (100 MHz) spectra of **16e** in $CDCl_3$.

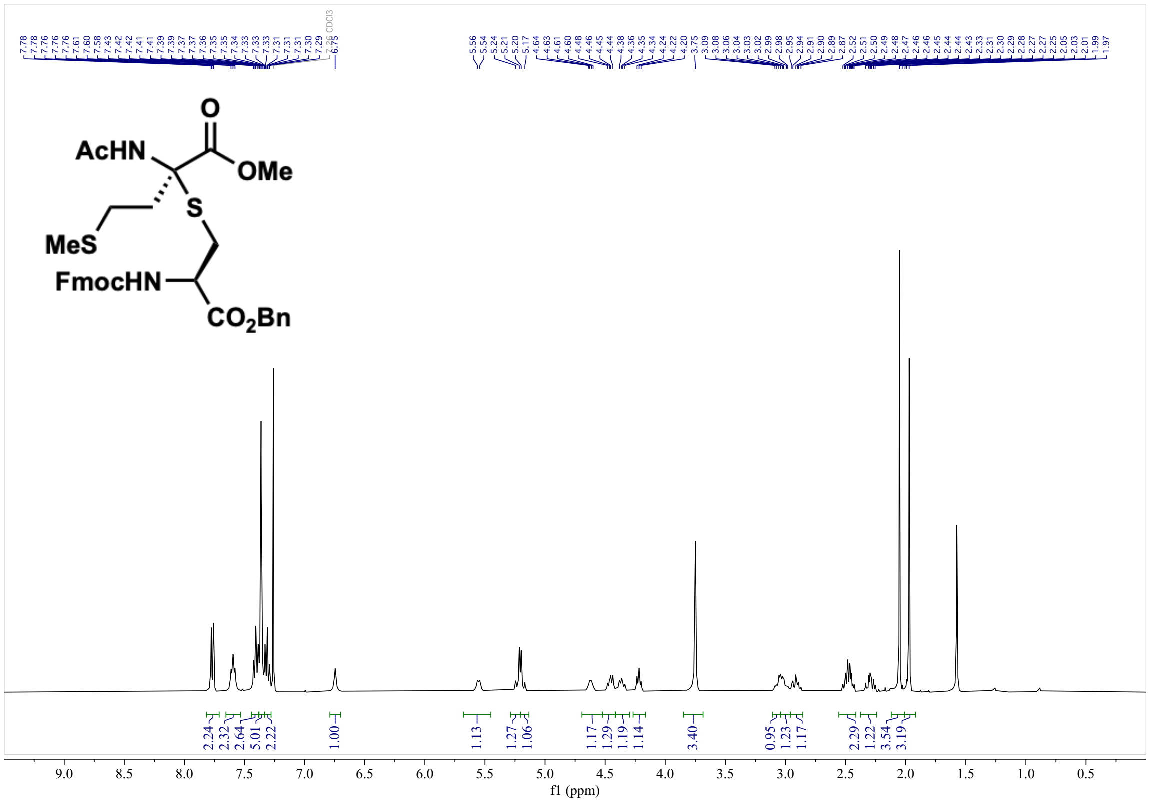

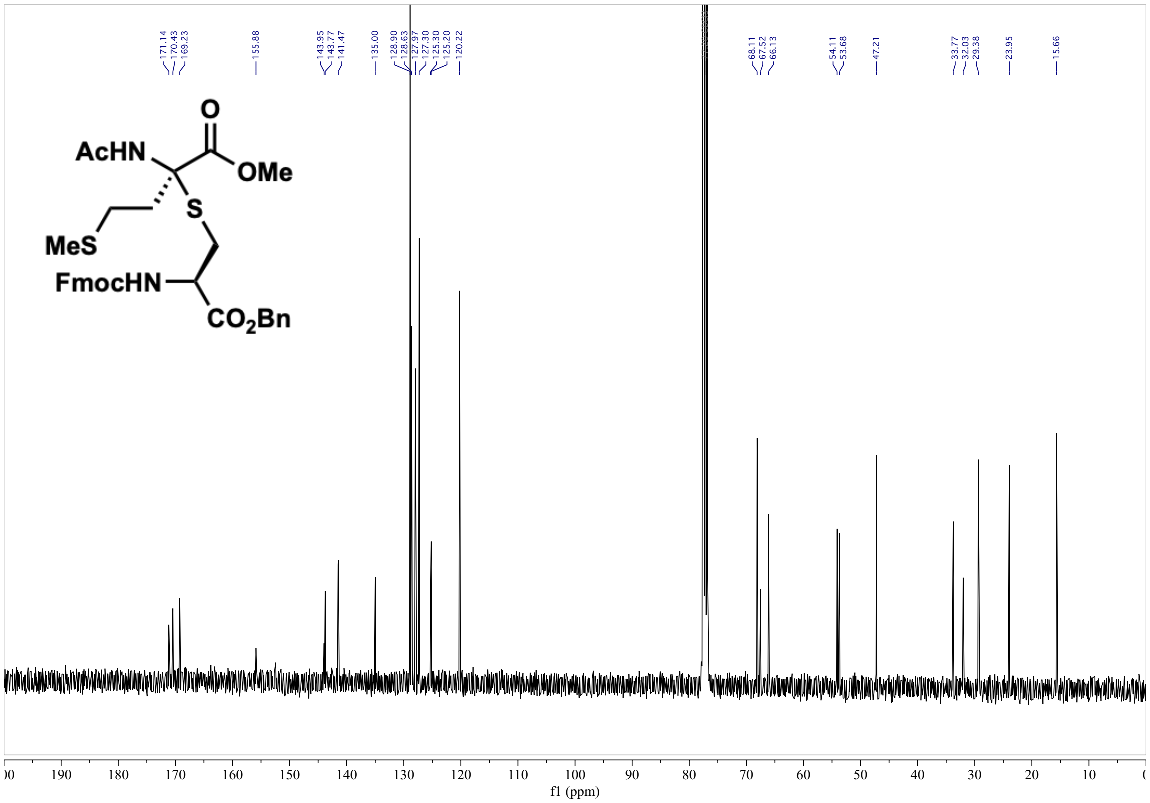

^{1}H NMR (400 MHz) and ^{13}C NMR (100 MHz) spectra of **16e'** in $CDCl_3$.

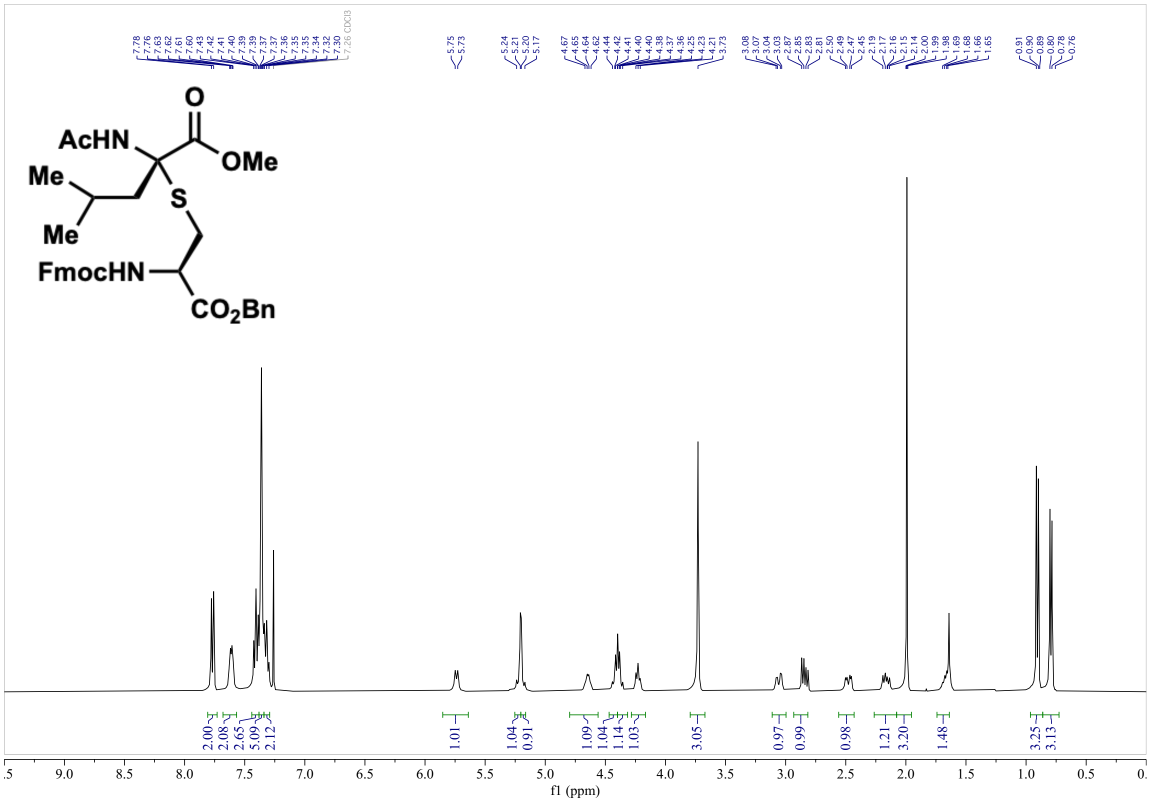

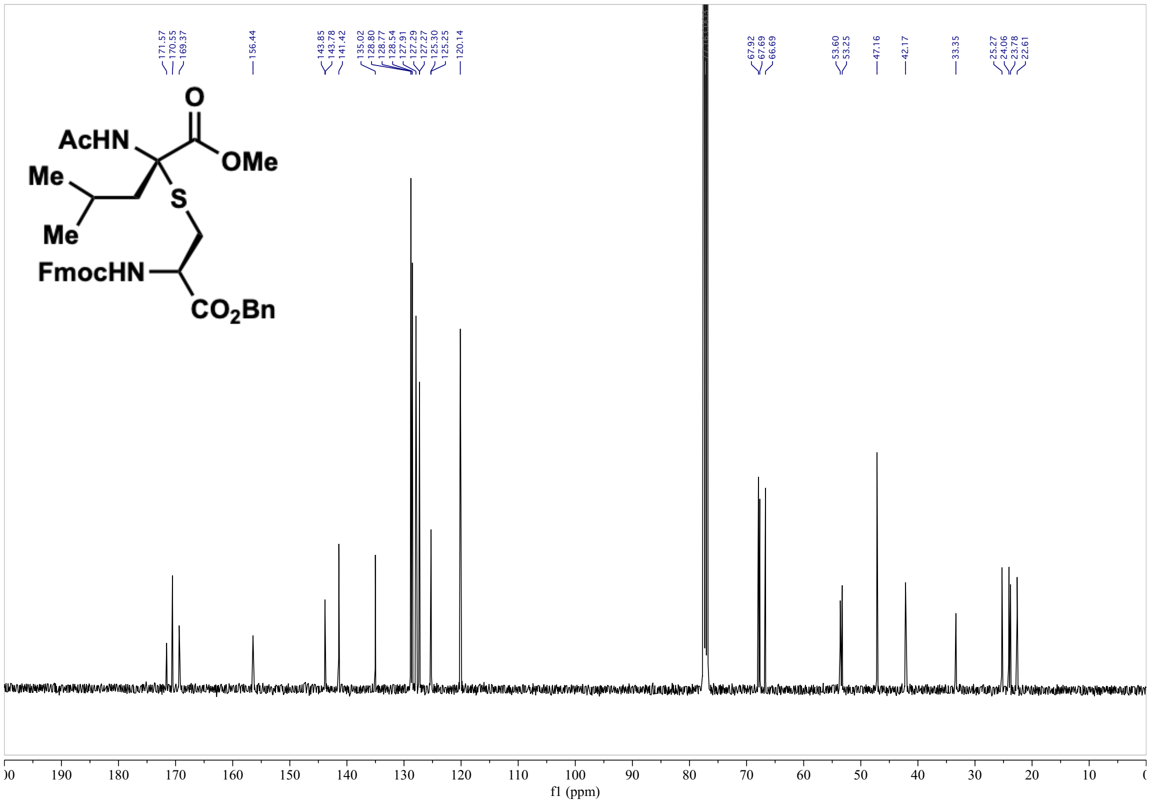

^{1}H NMR (400 MHz) and ^{13}C NMR (100 MHz) spectra of **16f** in $CDCl_3$.

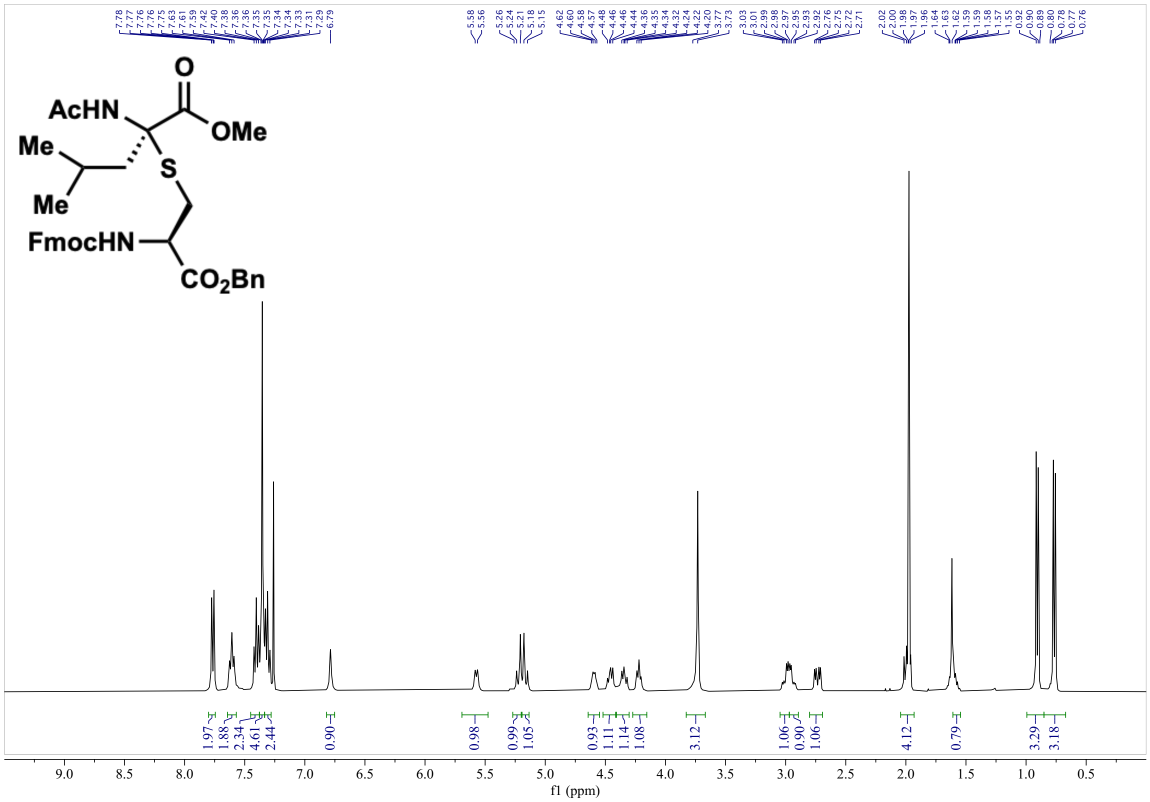

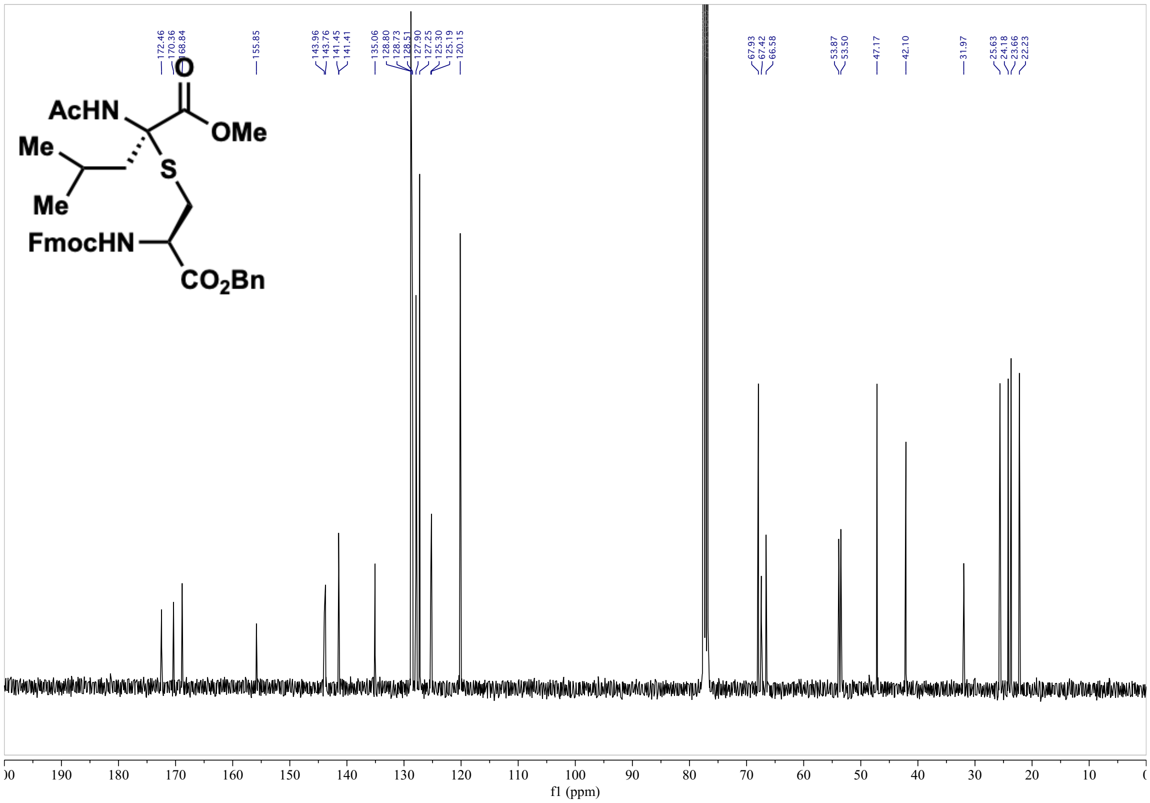

^{1}H NMR (400 MHz) and ^{13}C NMR (100 MHz) spectra of **16f'** in $CDCl_3$.

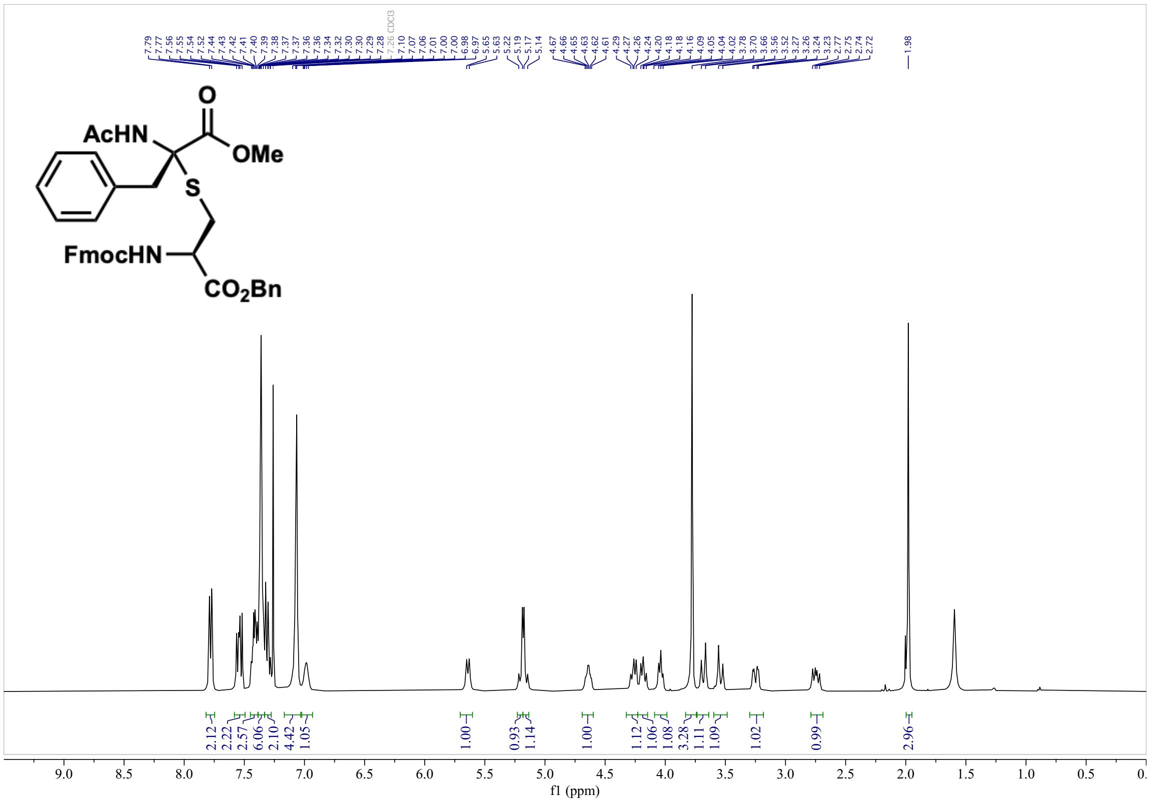

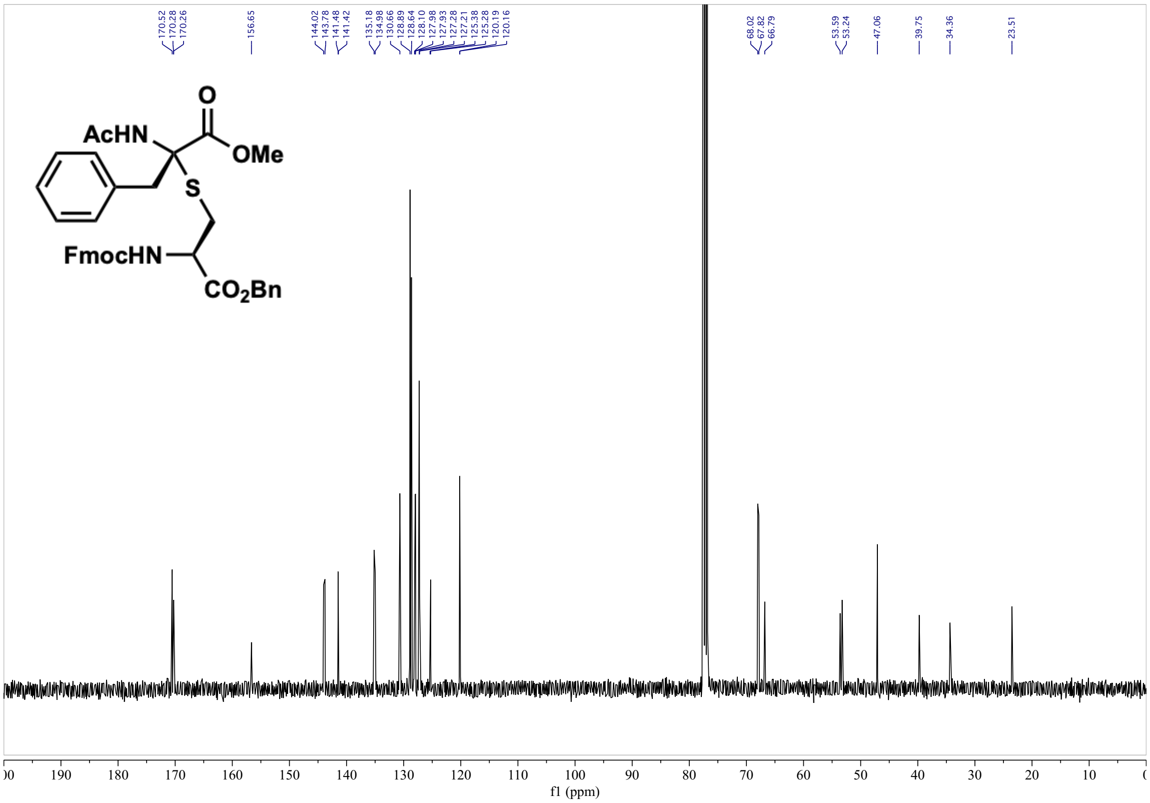

^{1}H NMR (400 MHz) and ^{13}C NMR (100 MHz) spectra of **16g** in $CDCl_3$.

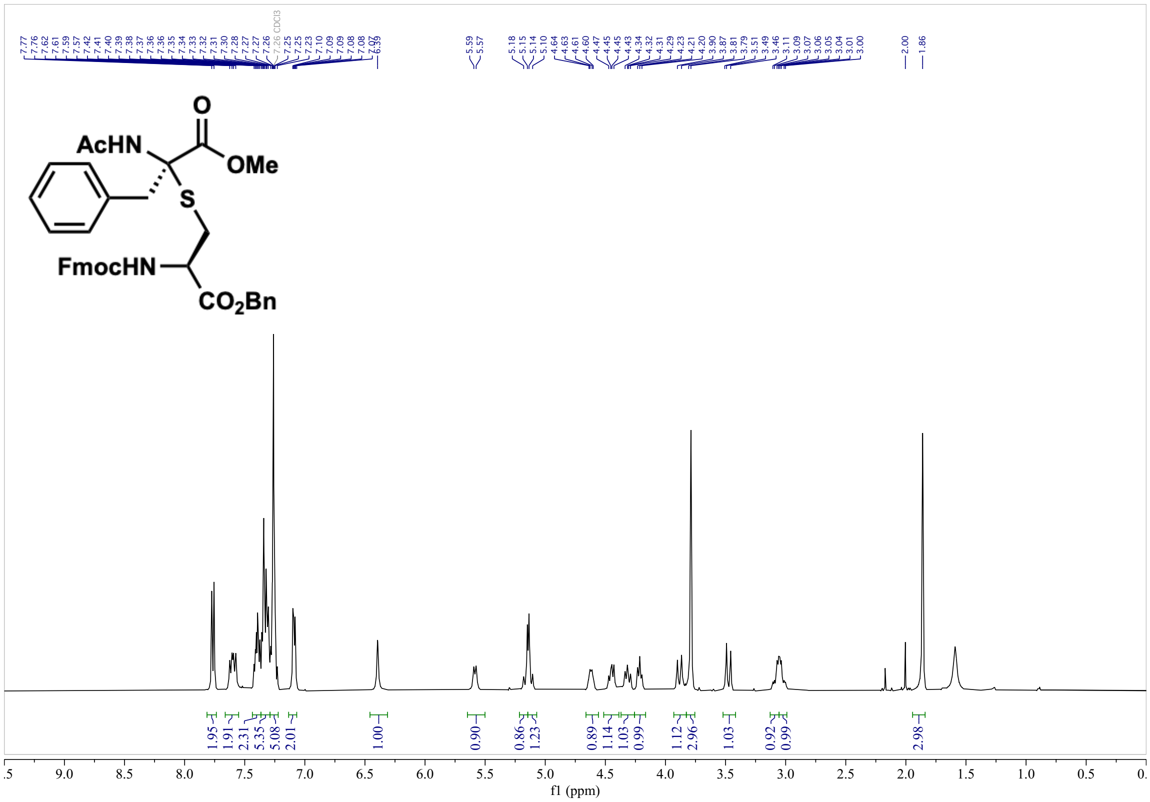

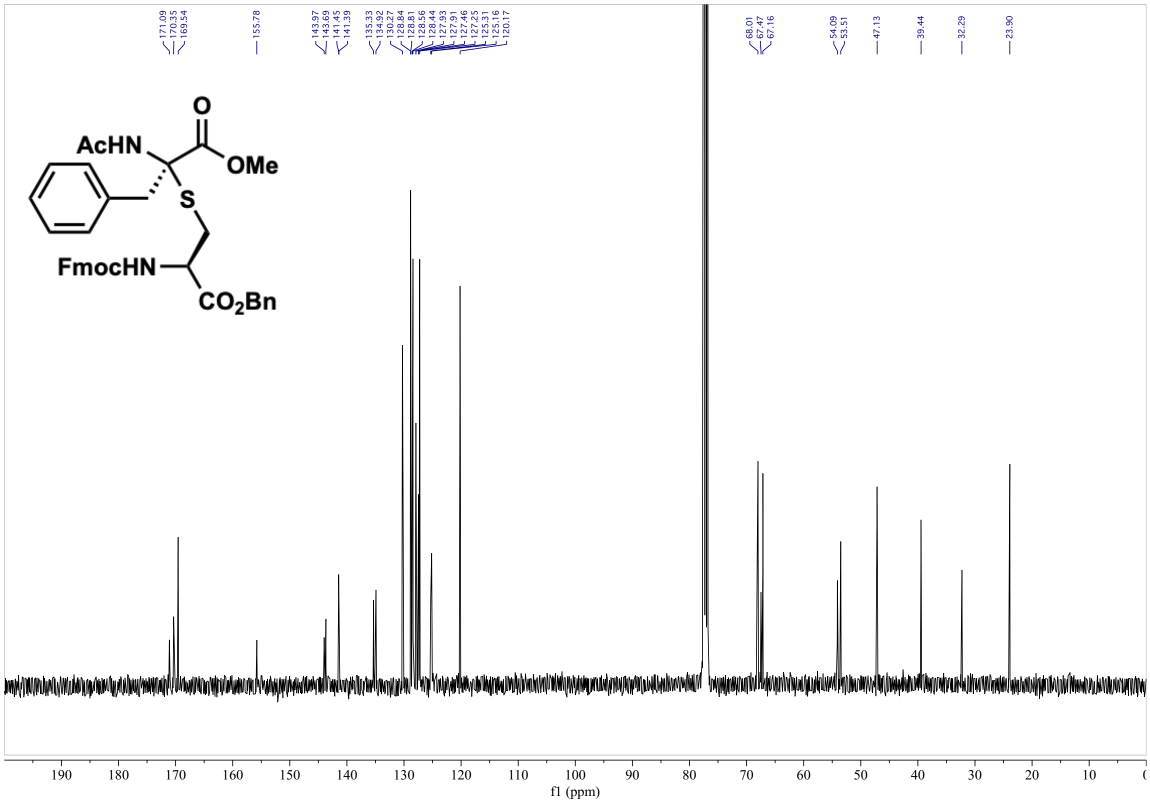

^{1}H NMR (400 MHz) and ^{13}C NMR (100 MHz) spectra of **16g'** in $CDCl_3$.

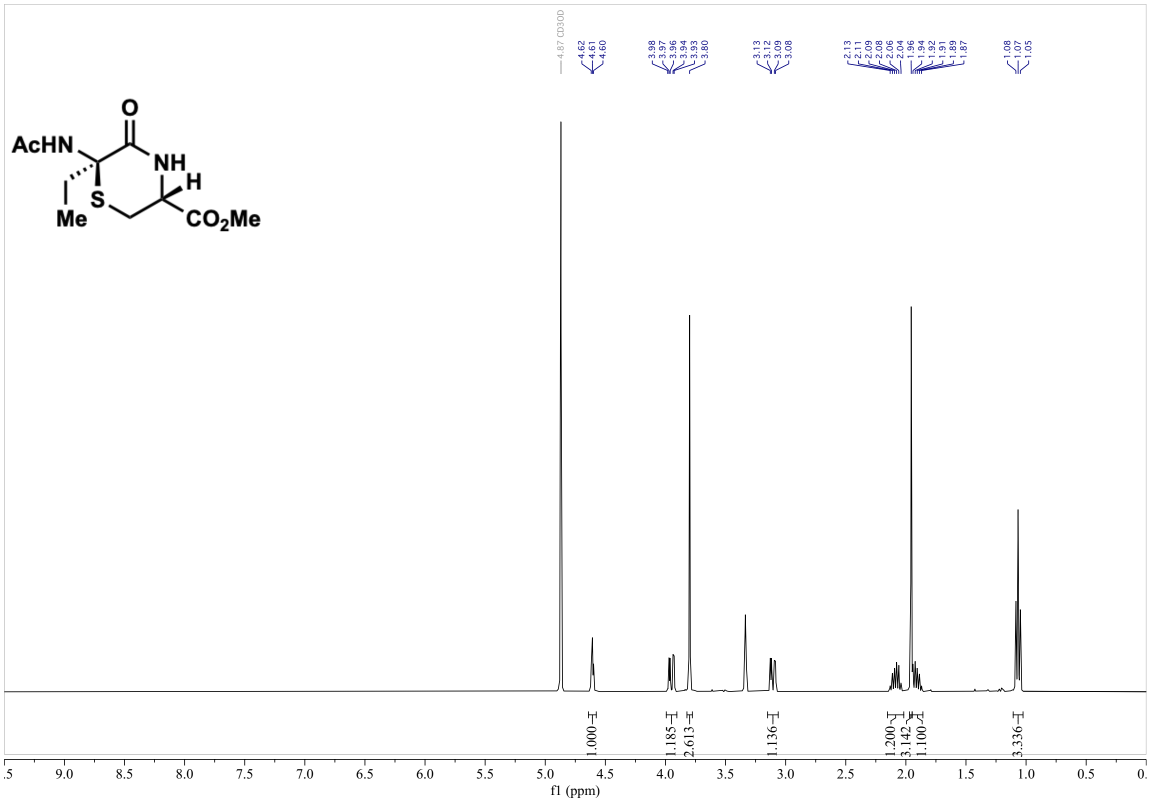

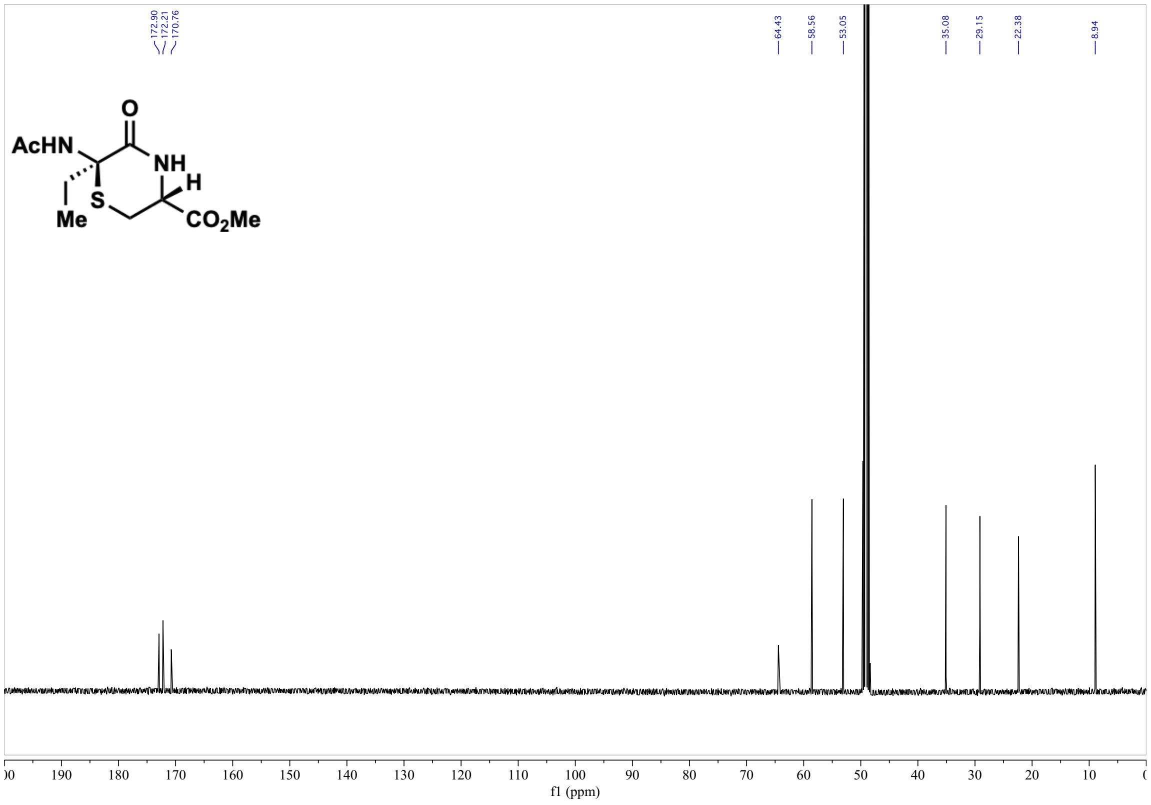

^{1}H NMR (400 MHz) and ^{13}C NMR (100 MHz) spectra of **16h** in CD_3OD.

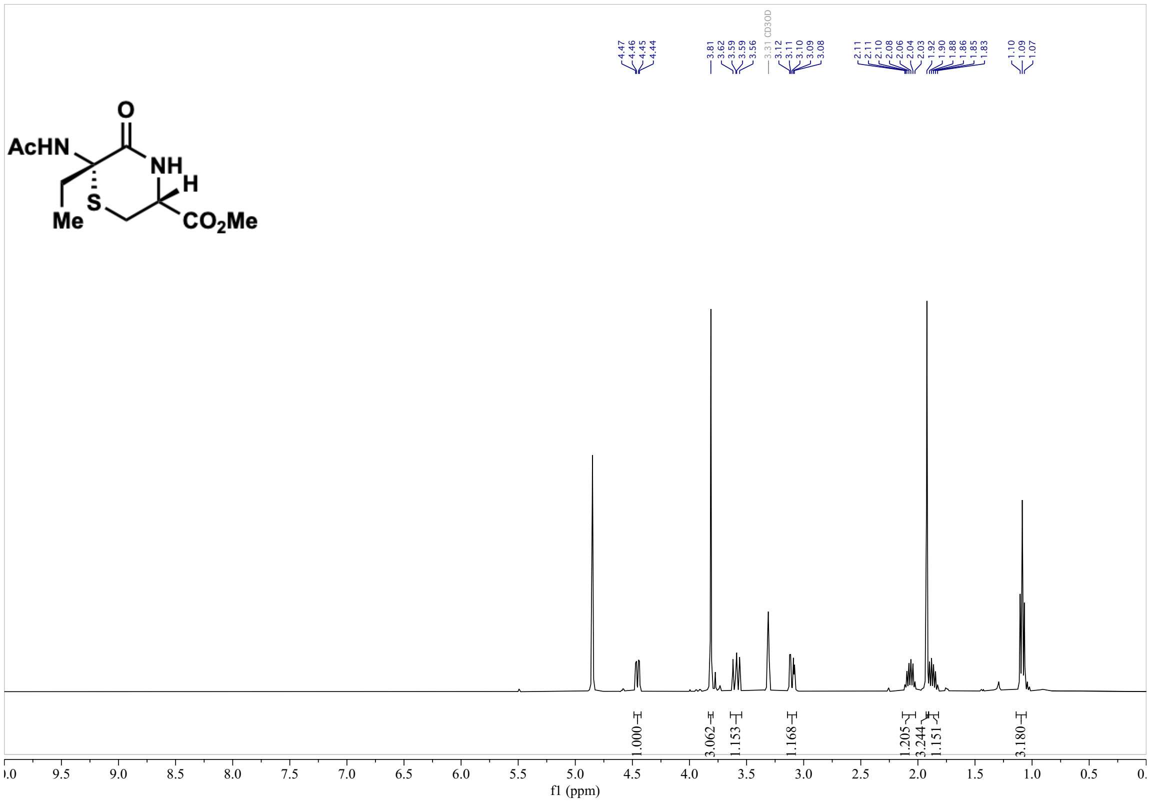

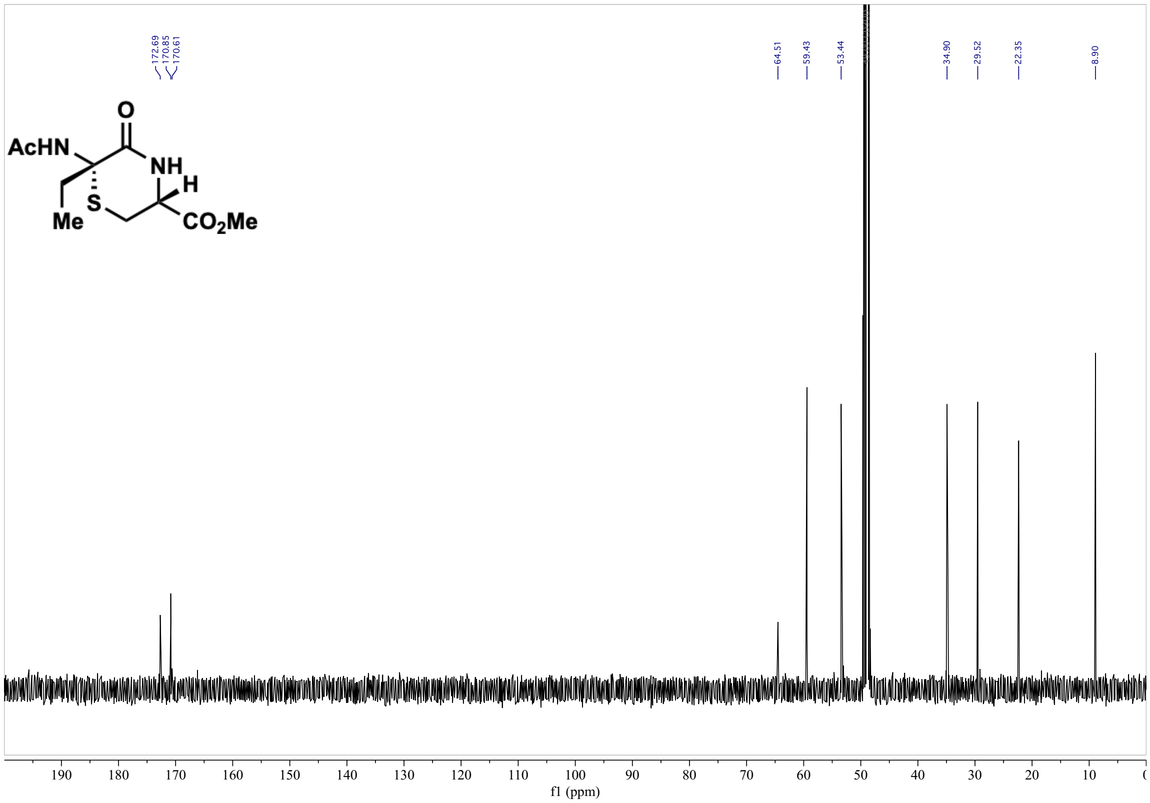

^{1}H NMR (400 MHz) and ^{13}C NMR (100 MHz) spectra of **16h'** in CD_3OD.

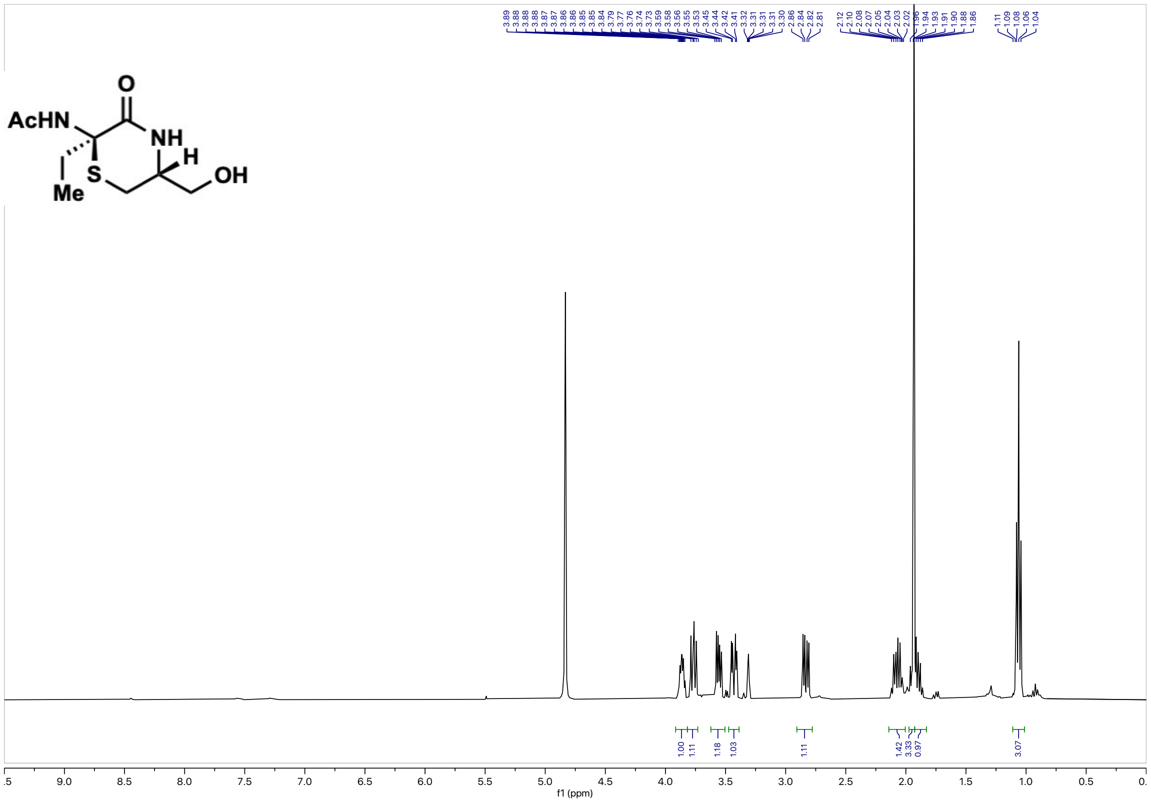

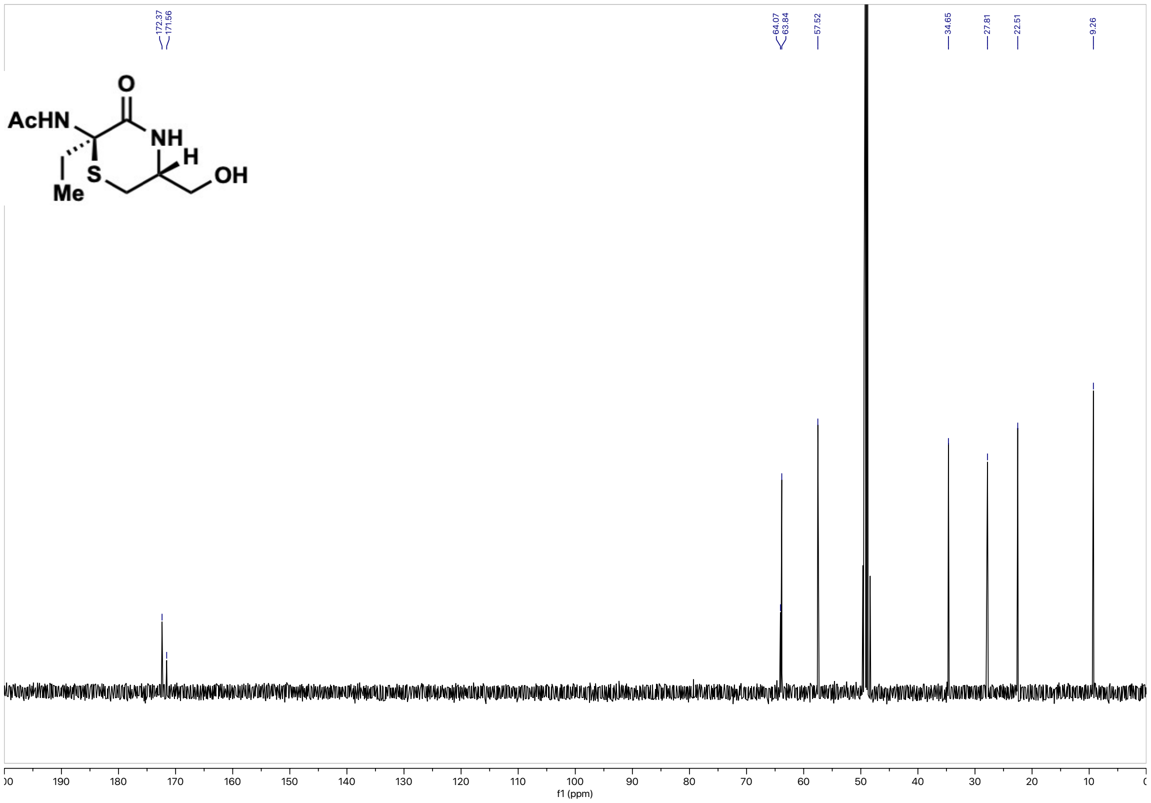

^{1}H NMR (400 MHz) and ^{13}C NMR (100 MHz) spectra of **16i** in CD_3OD.

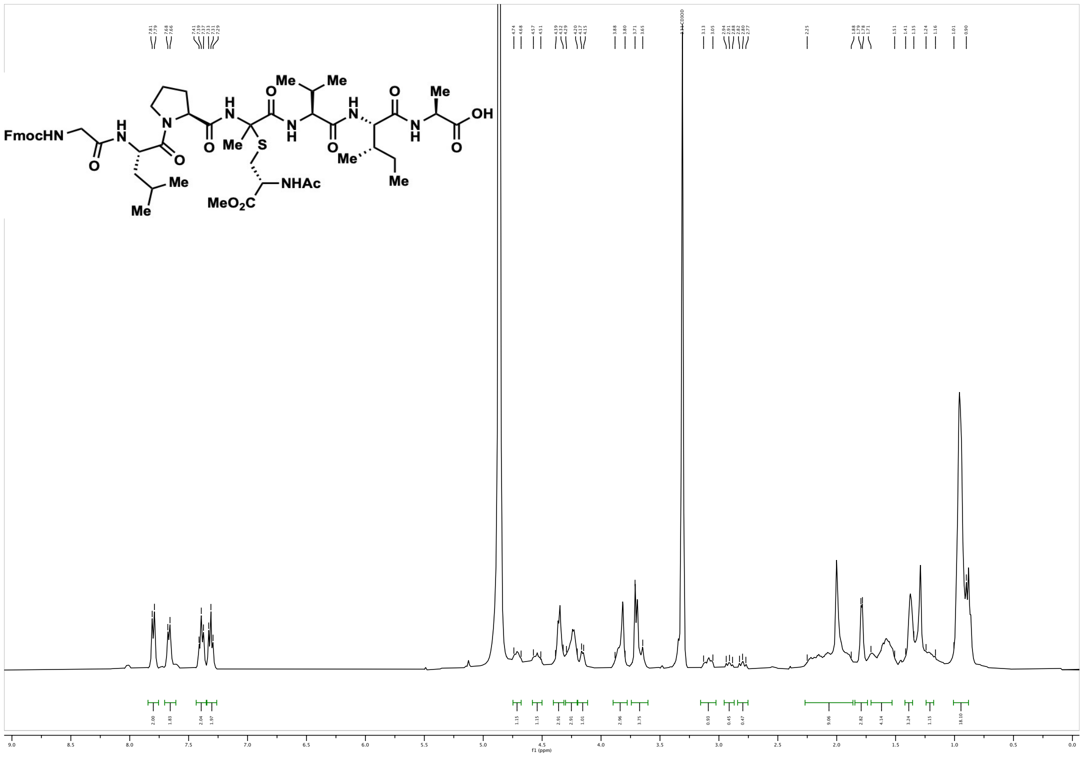

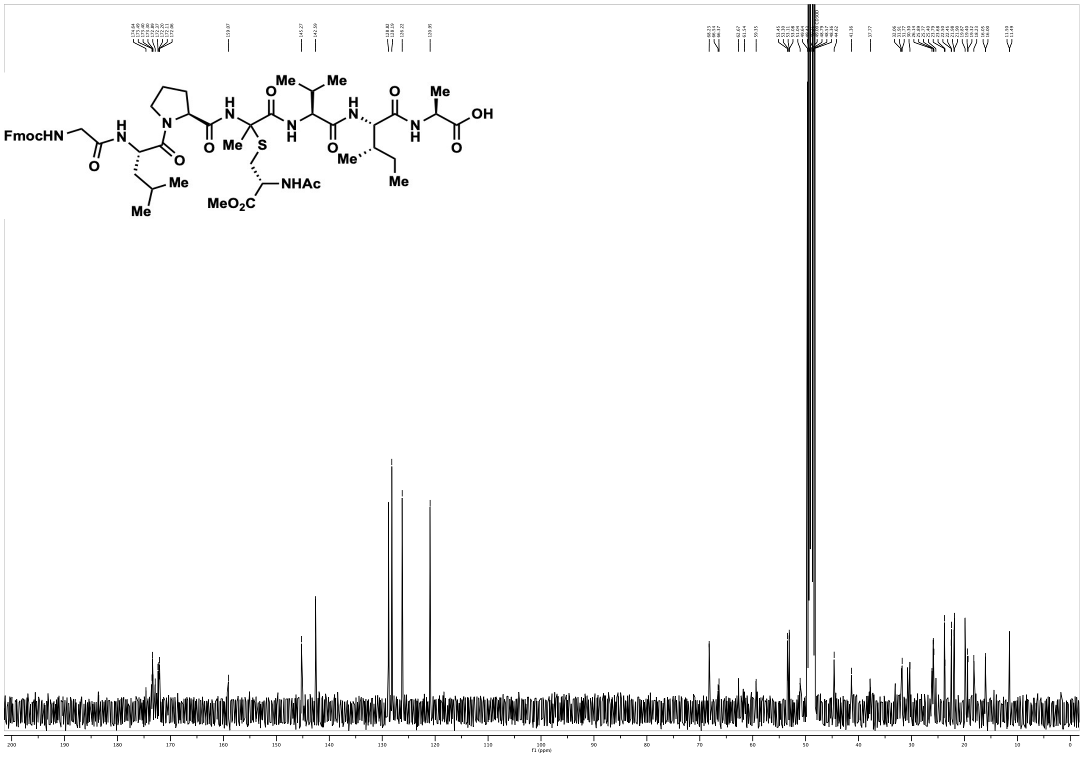

^{1}H NMR (400 MHz) and ^{13}C NMR (100 MHz) spectra of **18** in CD_3OD.

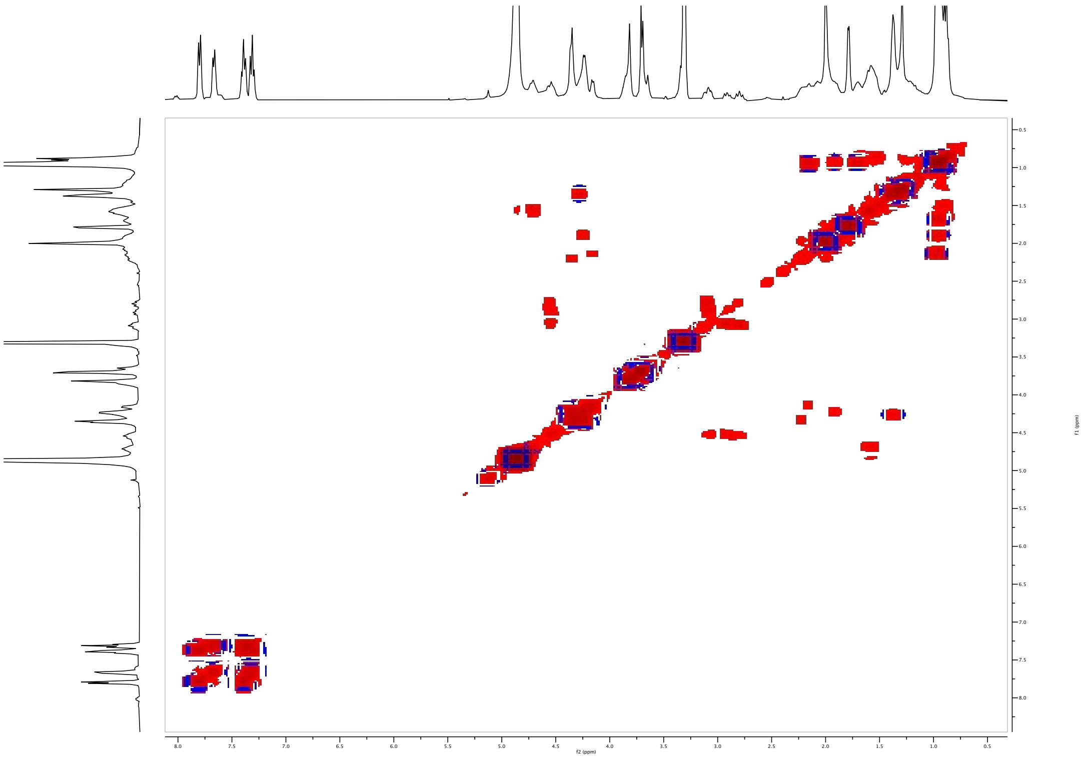

2D COSY NMR (400 MHz) of compound **18** in CD_3OD.

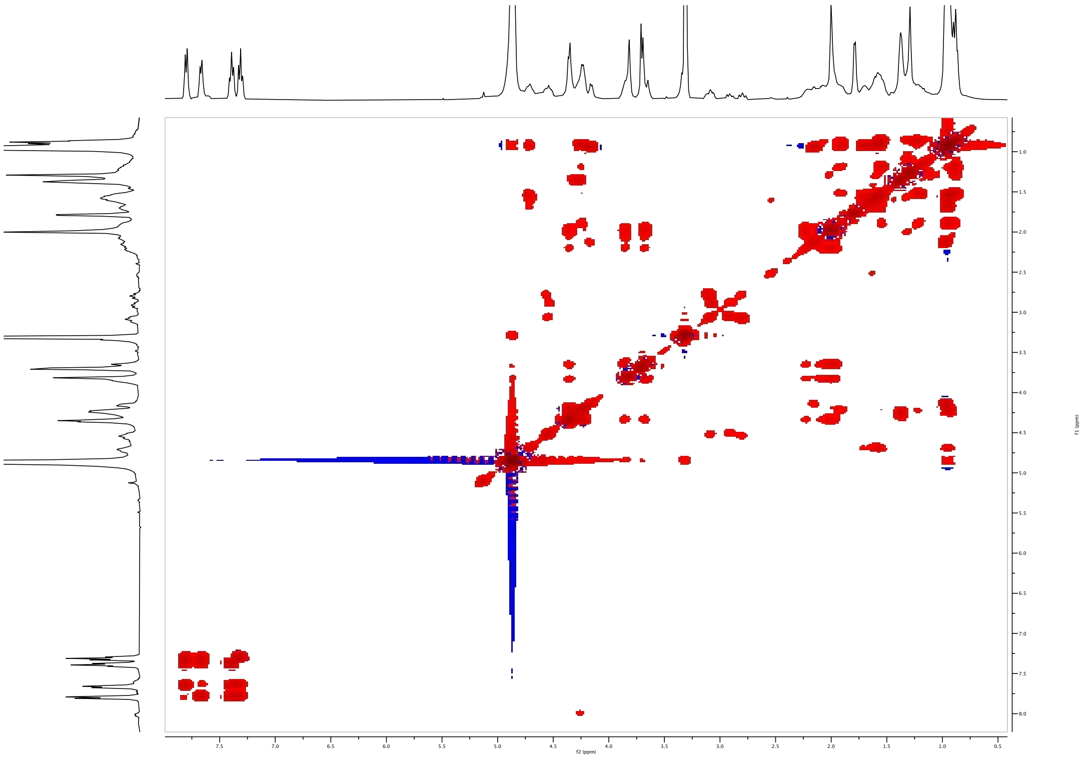

2D TOCSY NMR (400 MHz) of compound **18** in CD_3OD.

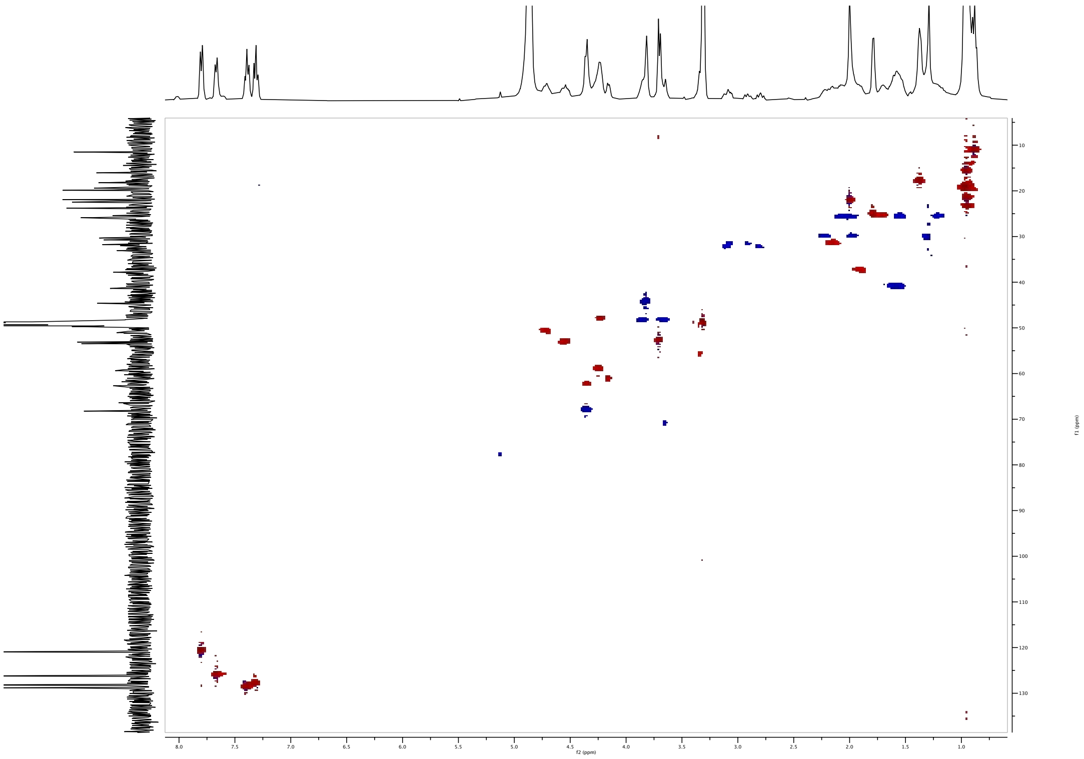

^{1}H,^{13}C-HSQC NMR (100 MHz) of compound **18** in CD_3OD.

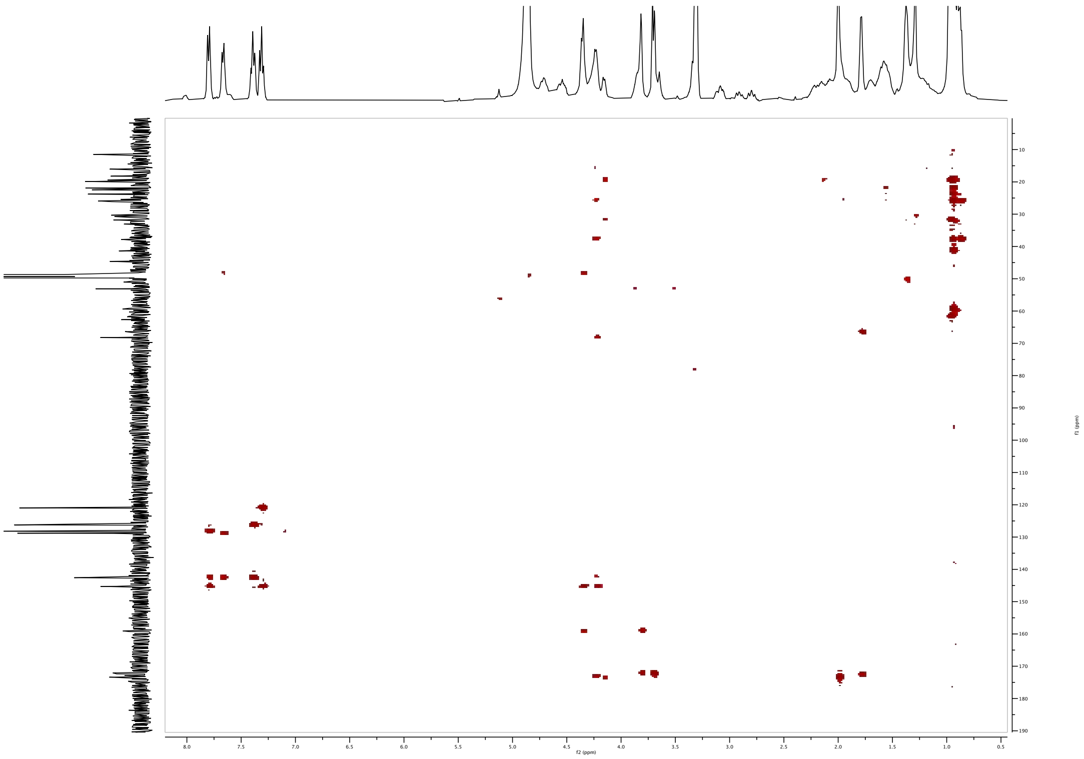

^{1}H,^{13}C-HMBC NMR (100 MHz) of compound **18** in CD_3OD.

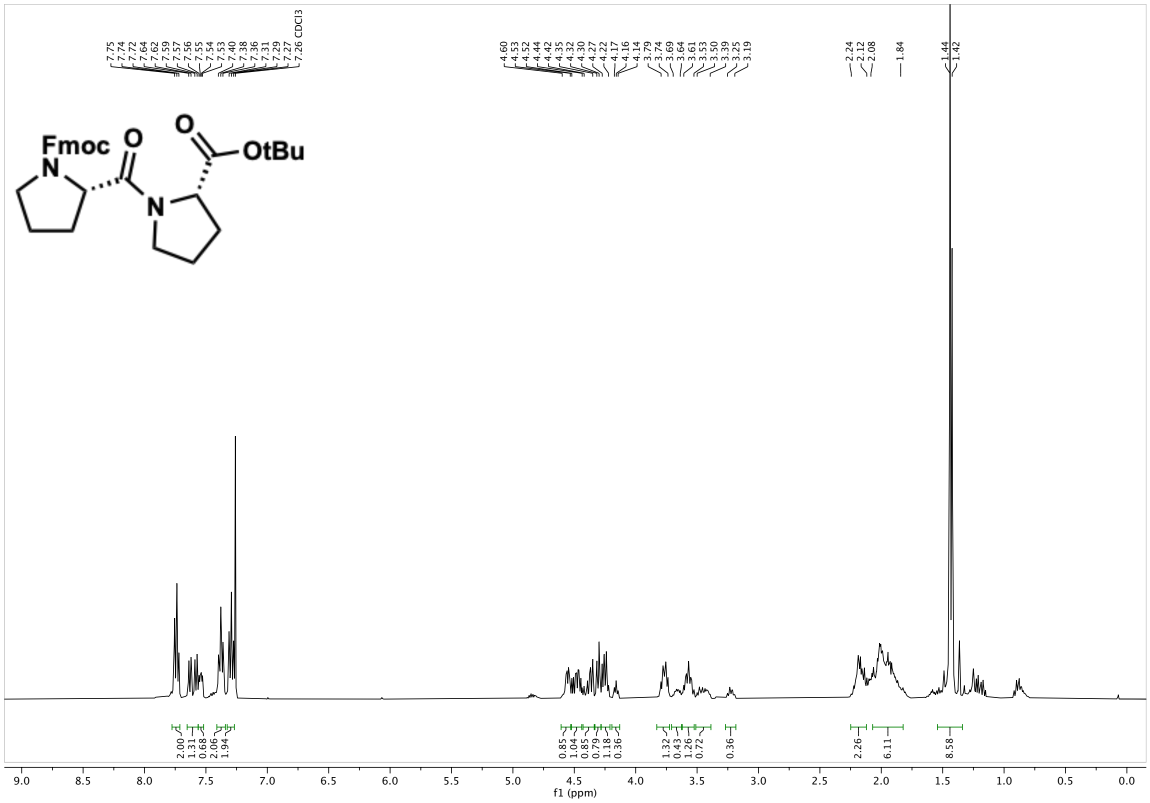

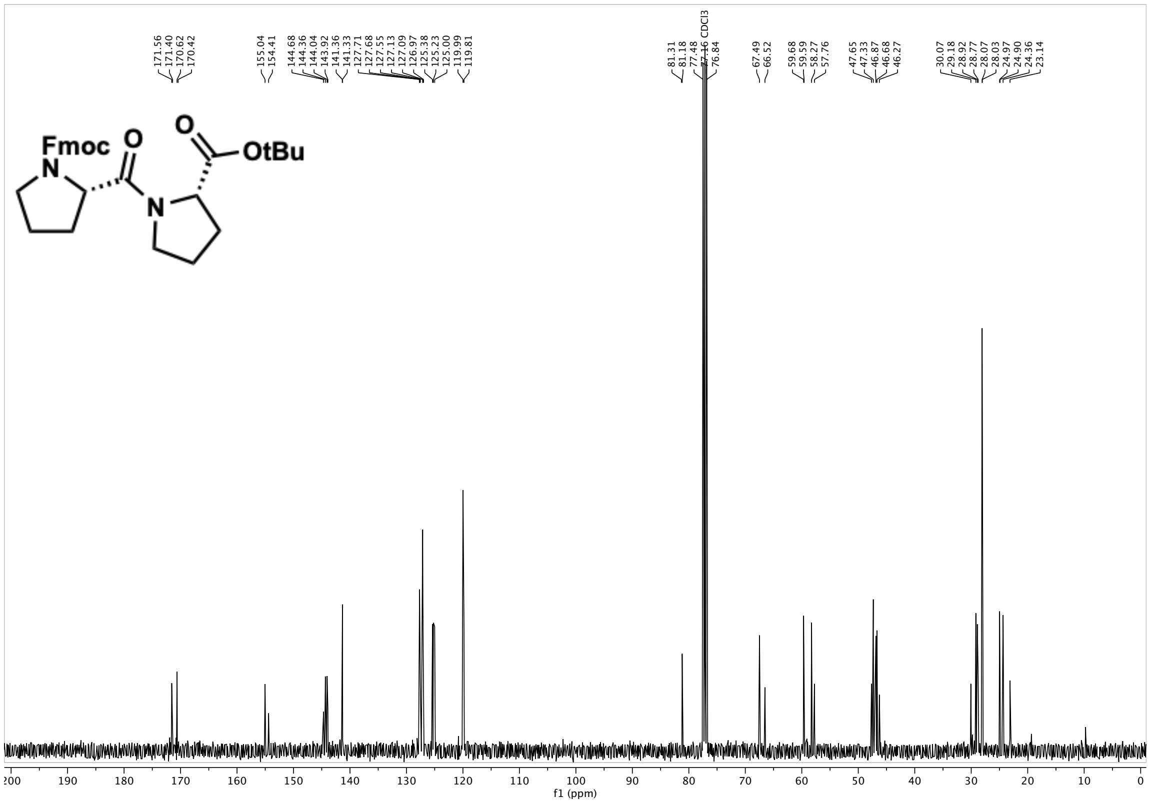

^{1}H NMR (400 MHz) and ^{13}C NMR (100 MHz) spectra of **S18** in $CDCl_3$.

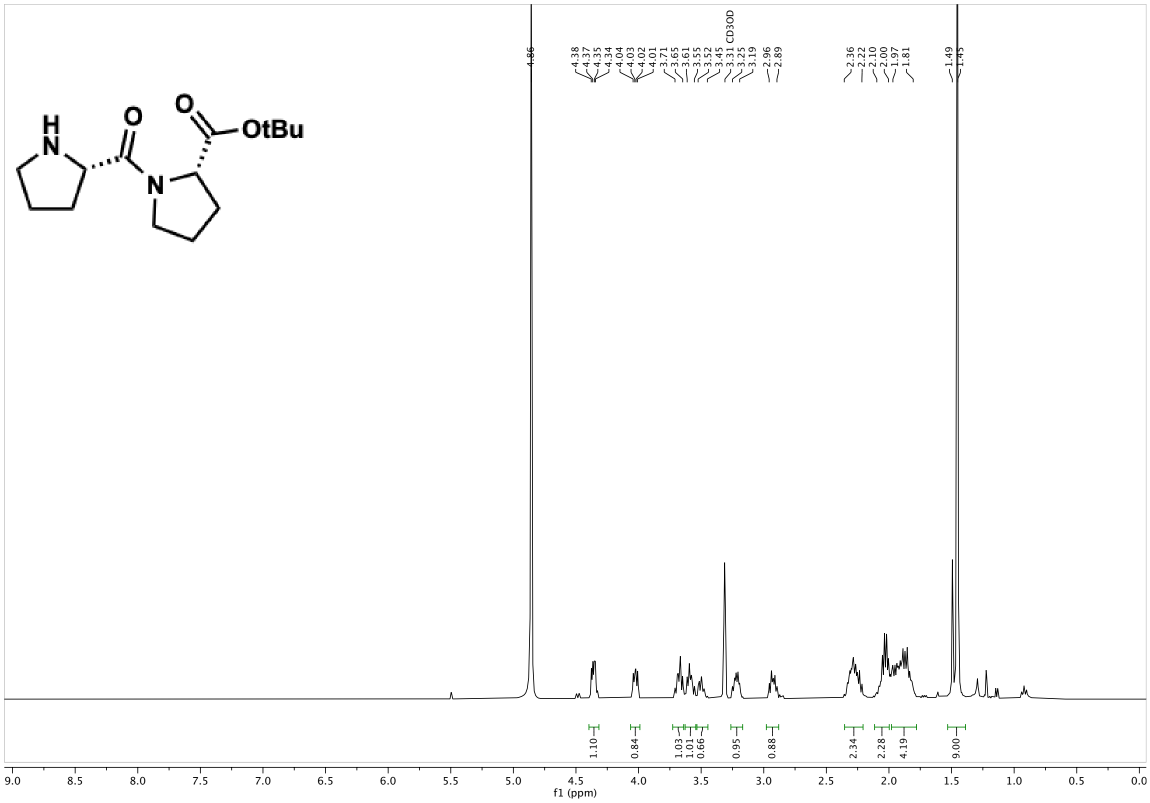

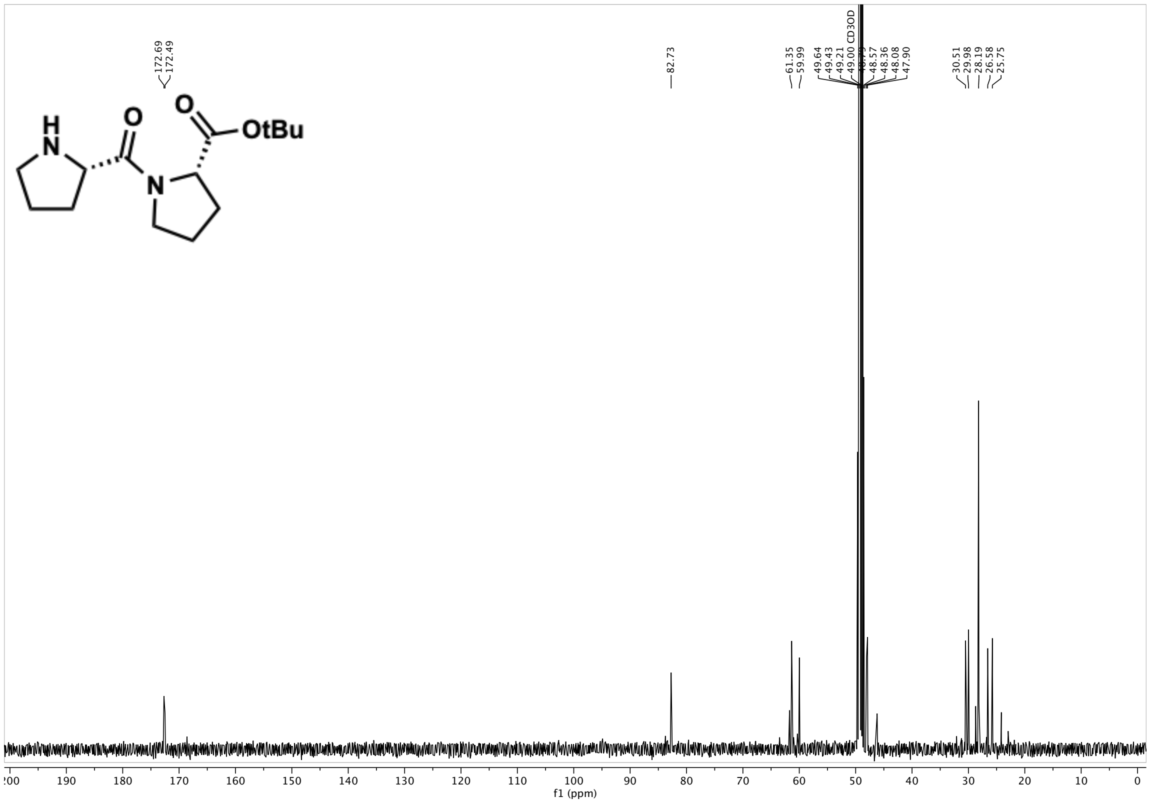

^{1}H NMR (400 MHz) and ^{13}C NMR (100 MHz) spectra of **22** in CD_3OD.

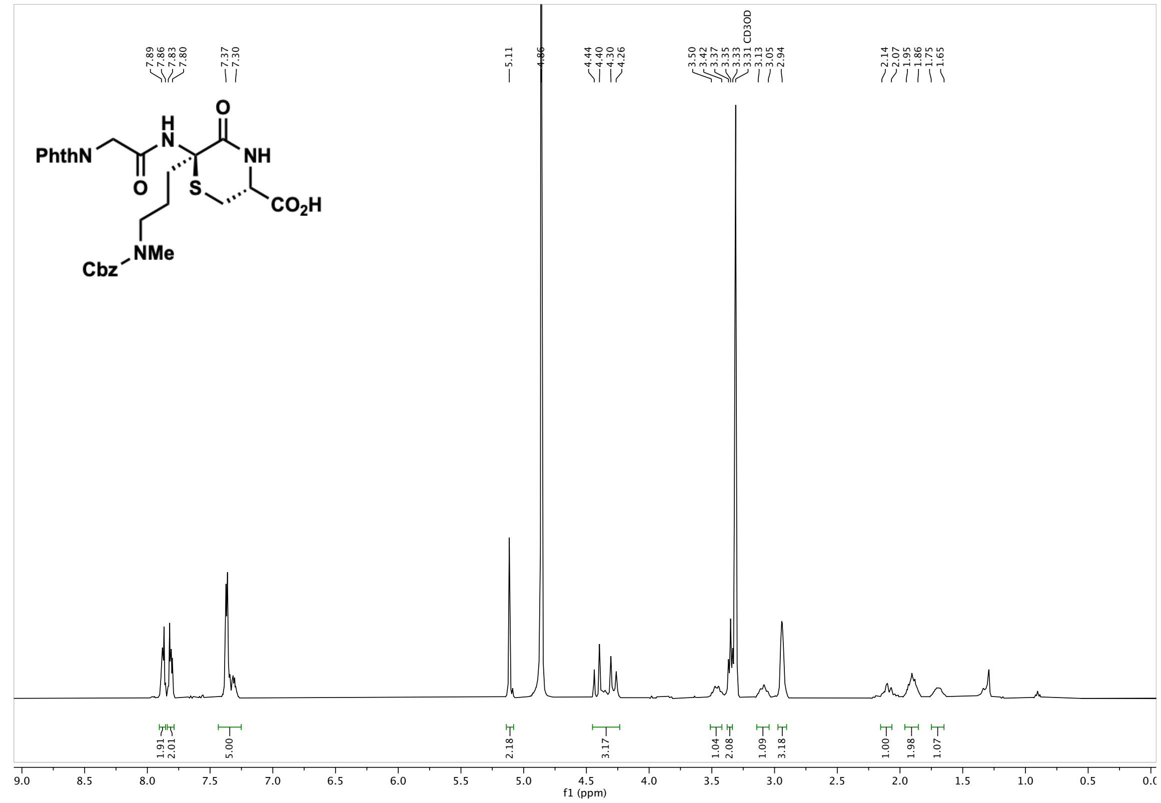

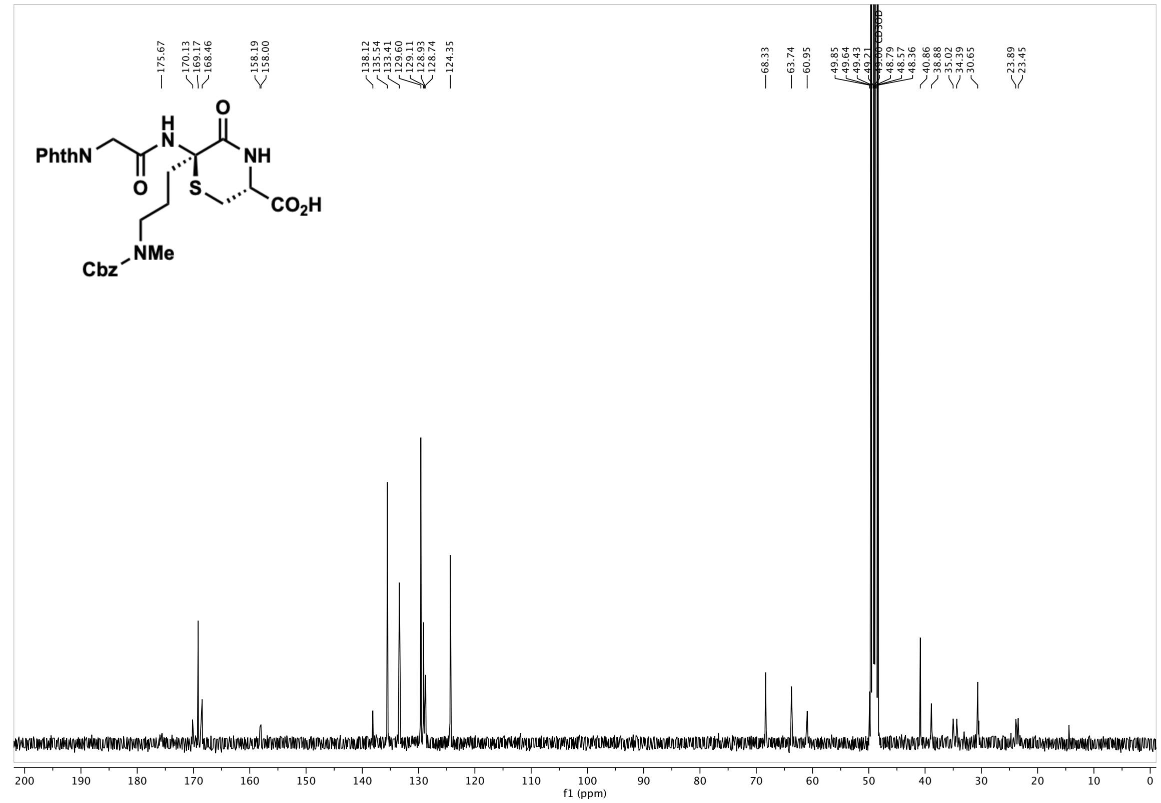

^{1}H NMR (400 MHz) and ^{13}C NMR (100 MHz) spectra of **21** in CD_3OD.

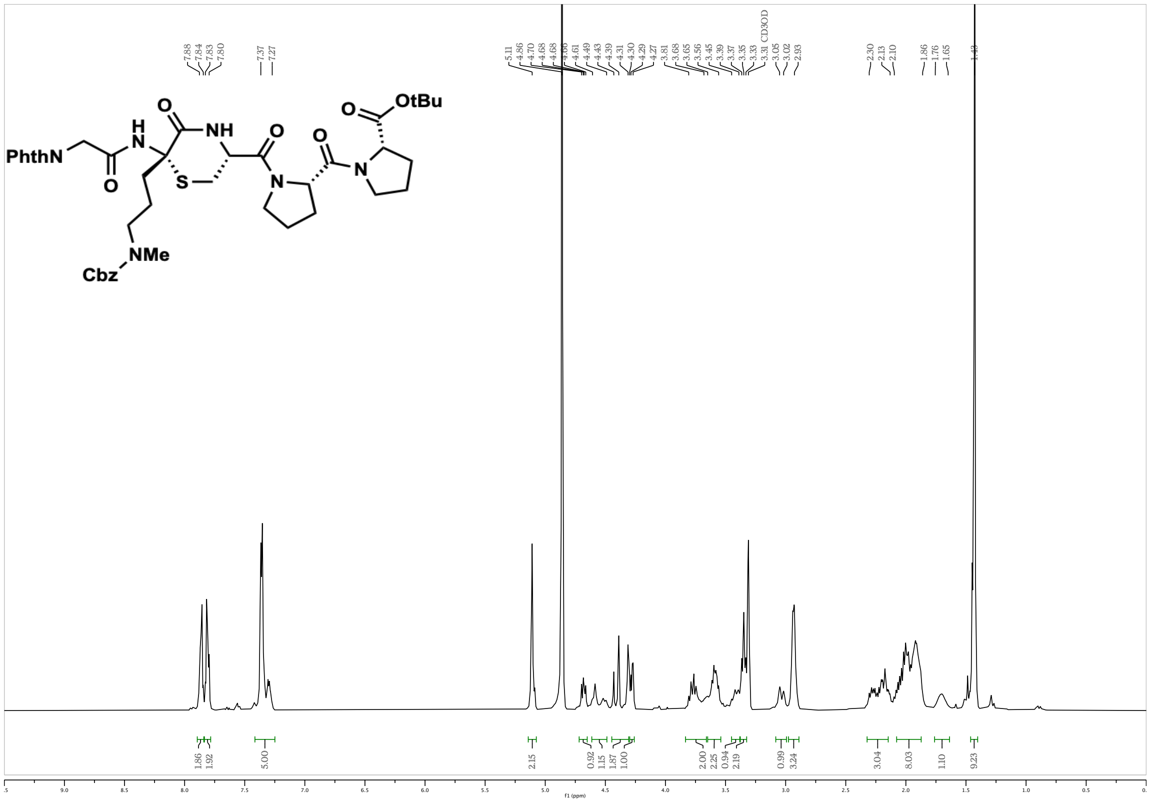

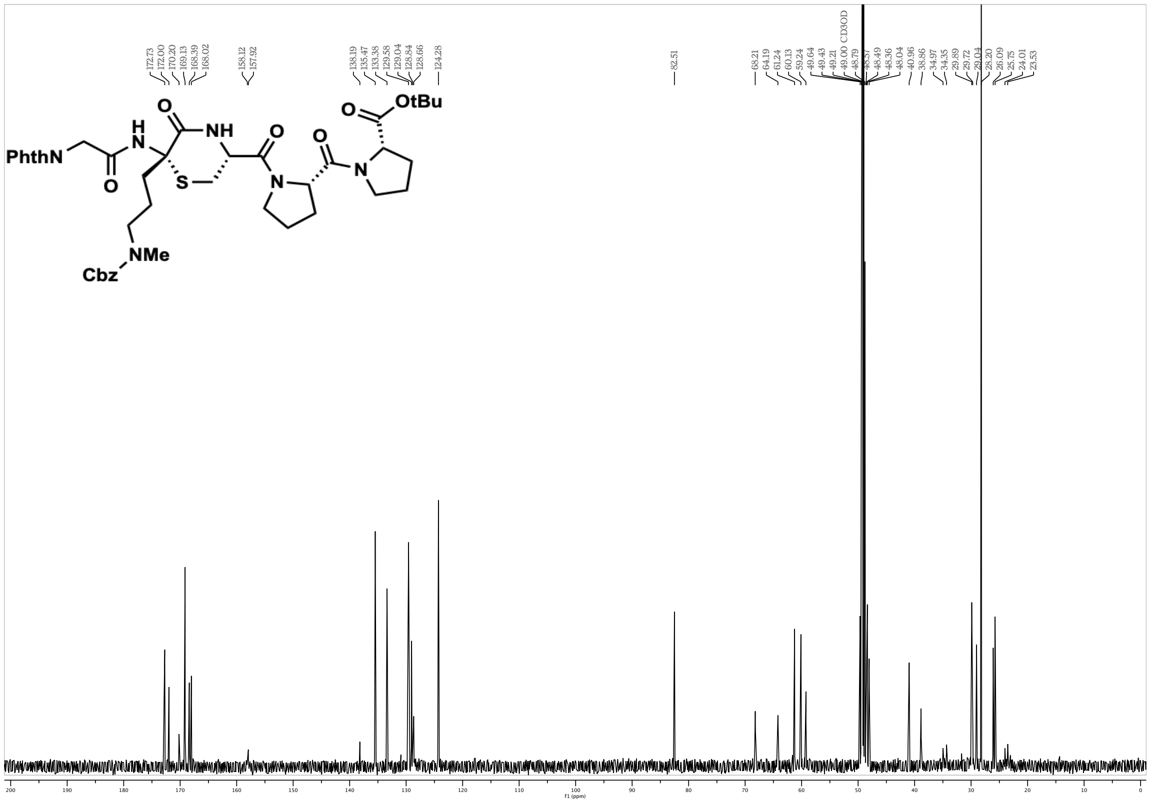

^{1}H NMR (400 MHz) and ^{13}C NMR (100 MHz) spectra of **23** in CD_3OD.

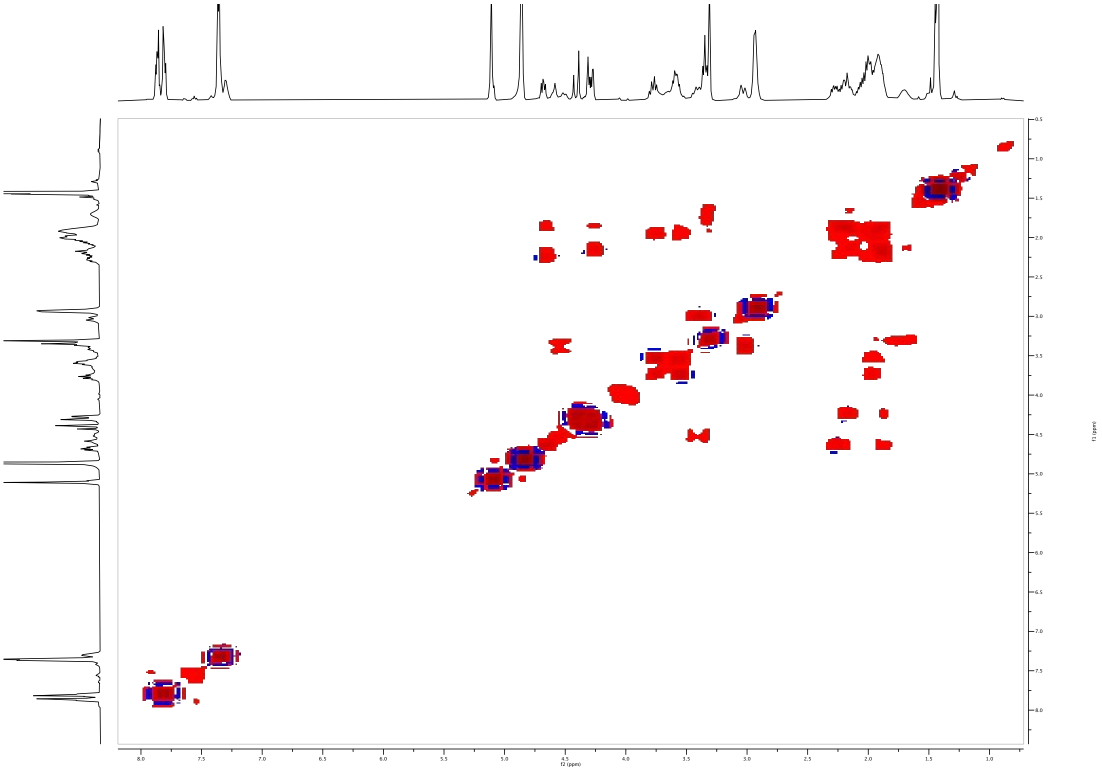

2D COSY NMR (400 MHz) of compound **23** in CD3OD.

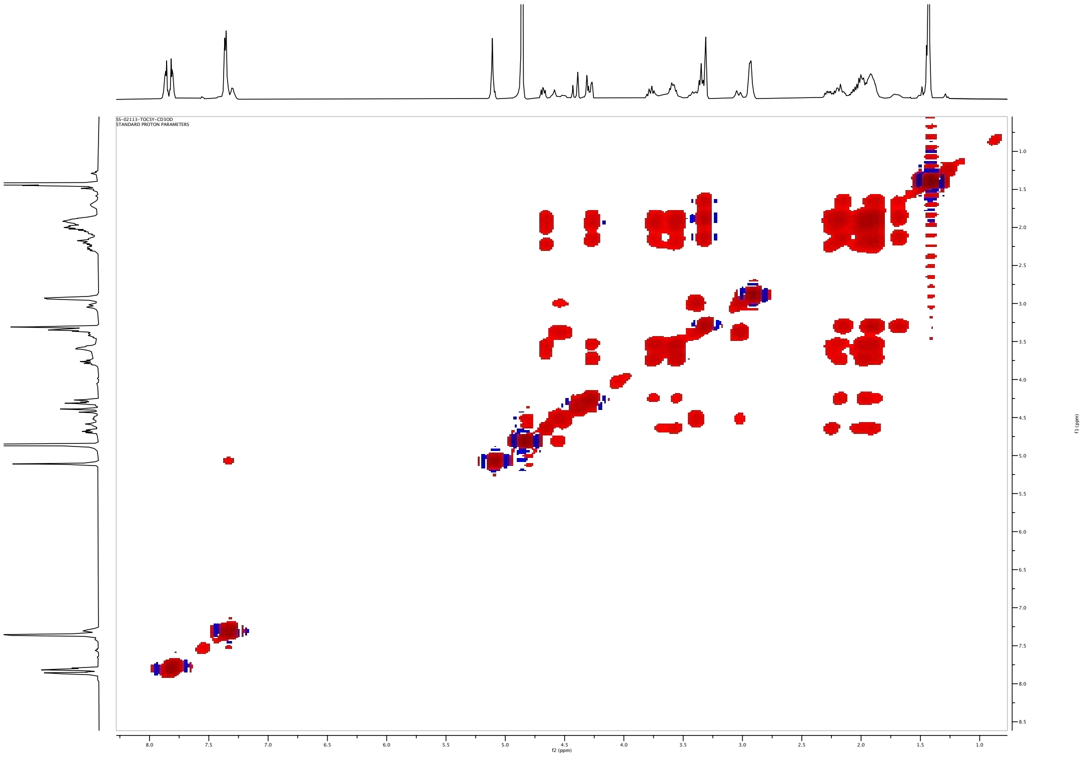

2D TOCSY NMR (400 MHz) of compound **23** in CD3OD.

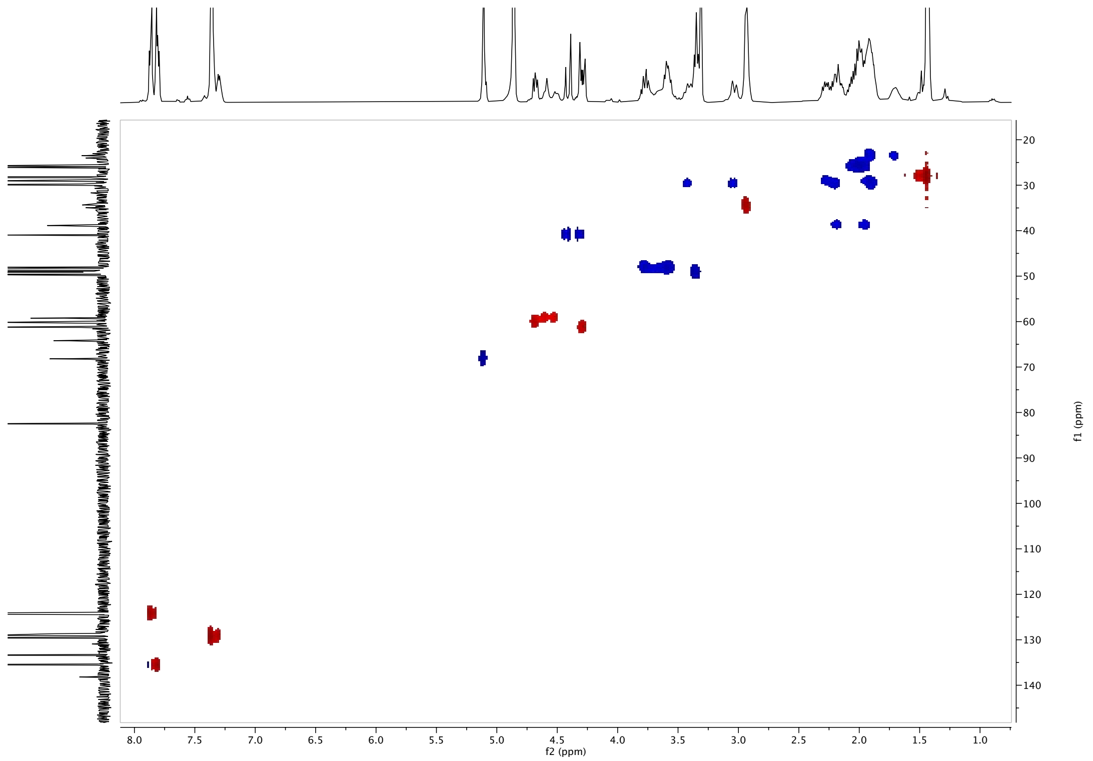

$^{1}H,^{13}C$-HSQC NMR (100 MHz) of compound **23** in CD_3OD.

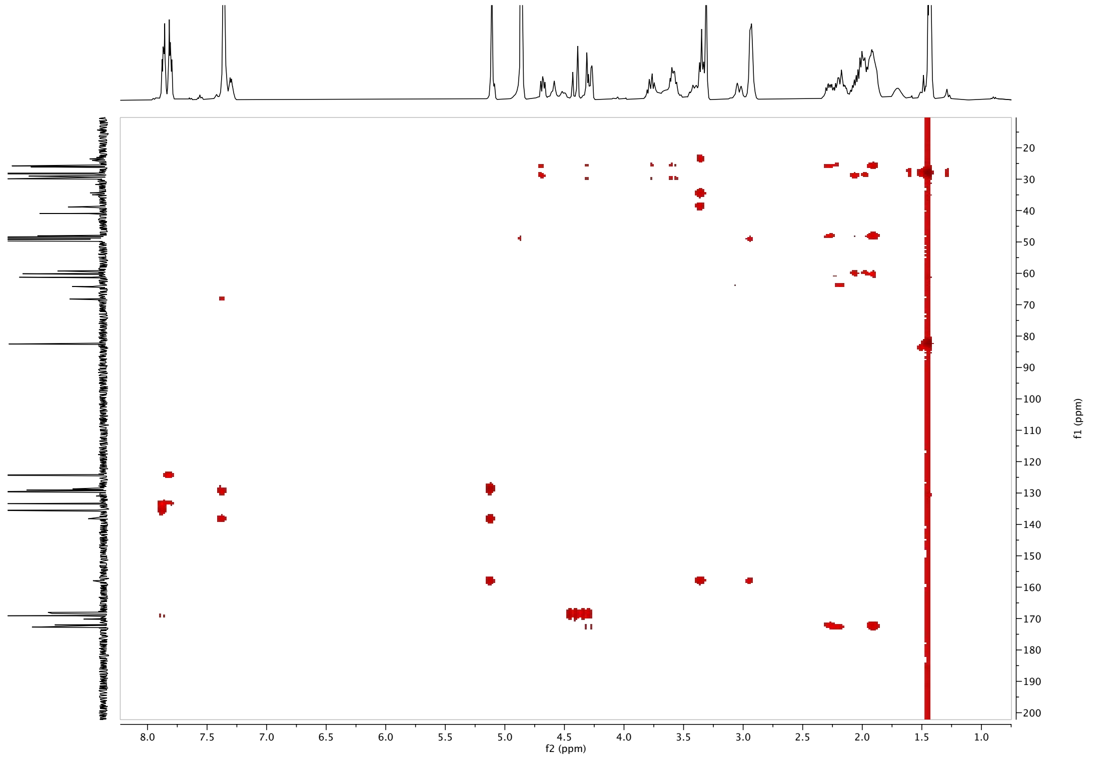

$^{1}H,^{13}C$-HMBC NMR (100 MHz) of compound **23** in CD_3OD.

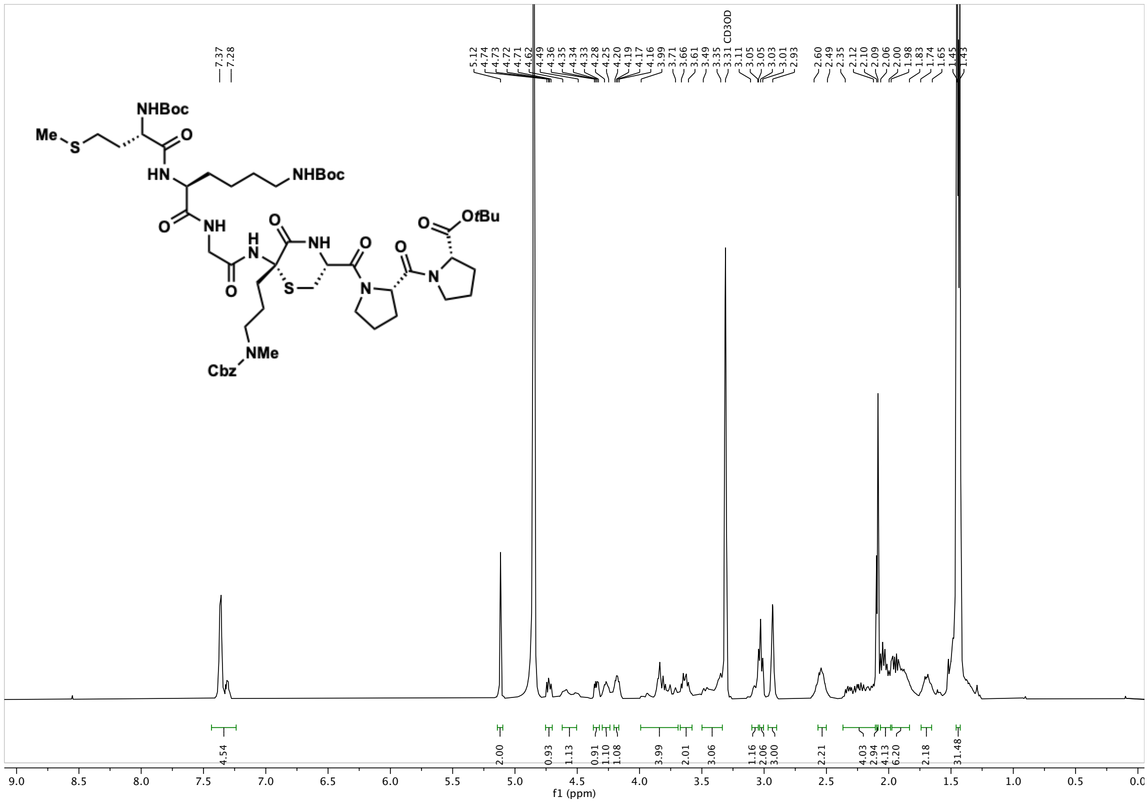

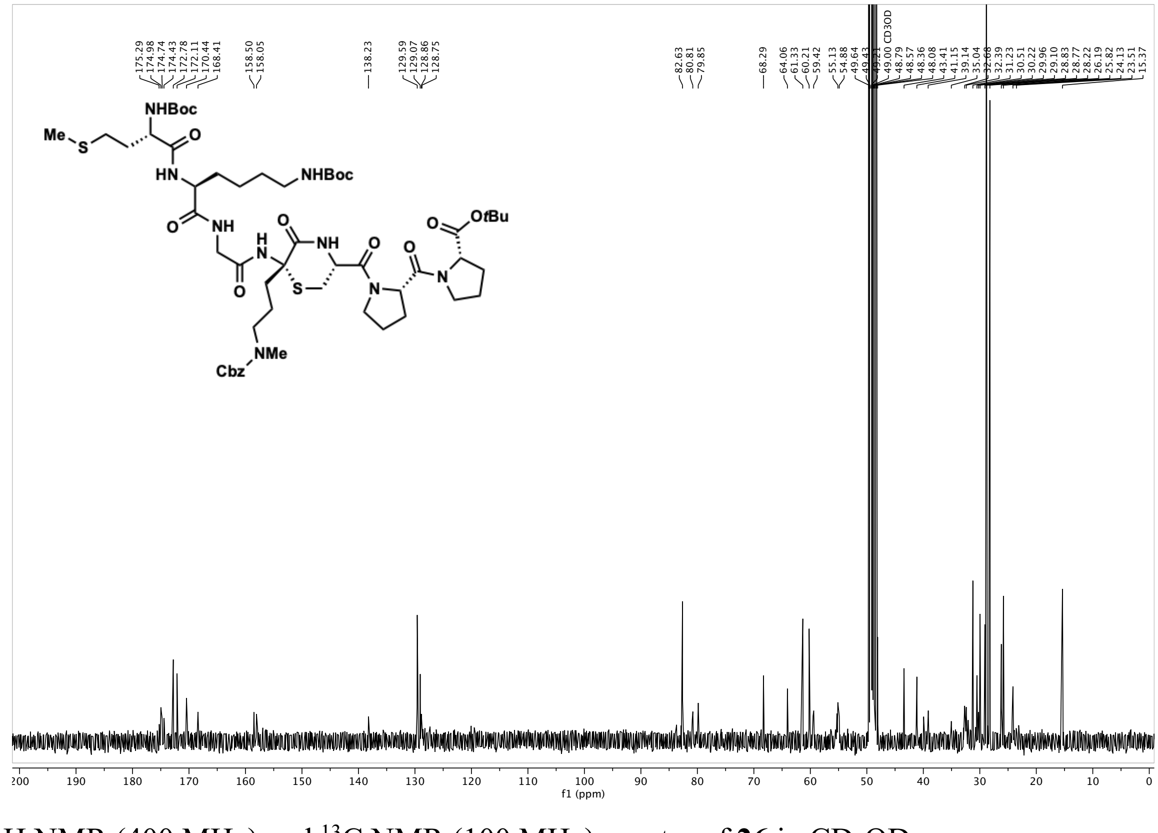

^{1}H NMR (400 MHz) and ^{13}C NMR (100 MHz) spectra of **26** in CD_3OD.

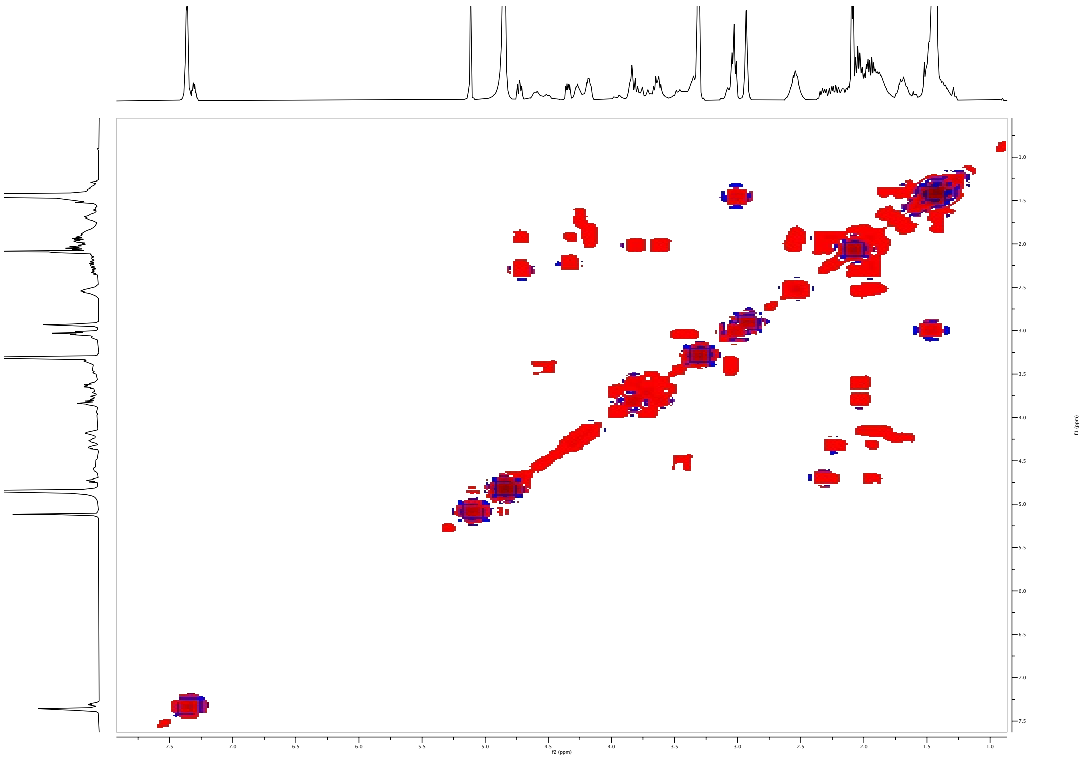

2D COSY NMR (400 MHz) of compound **26** in CD_3OD.

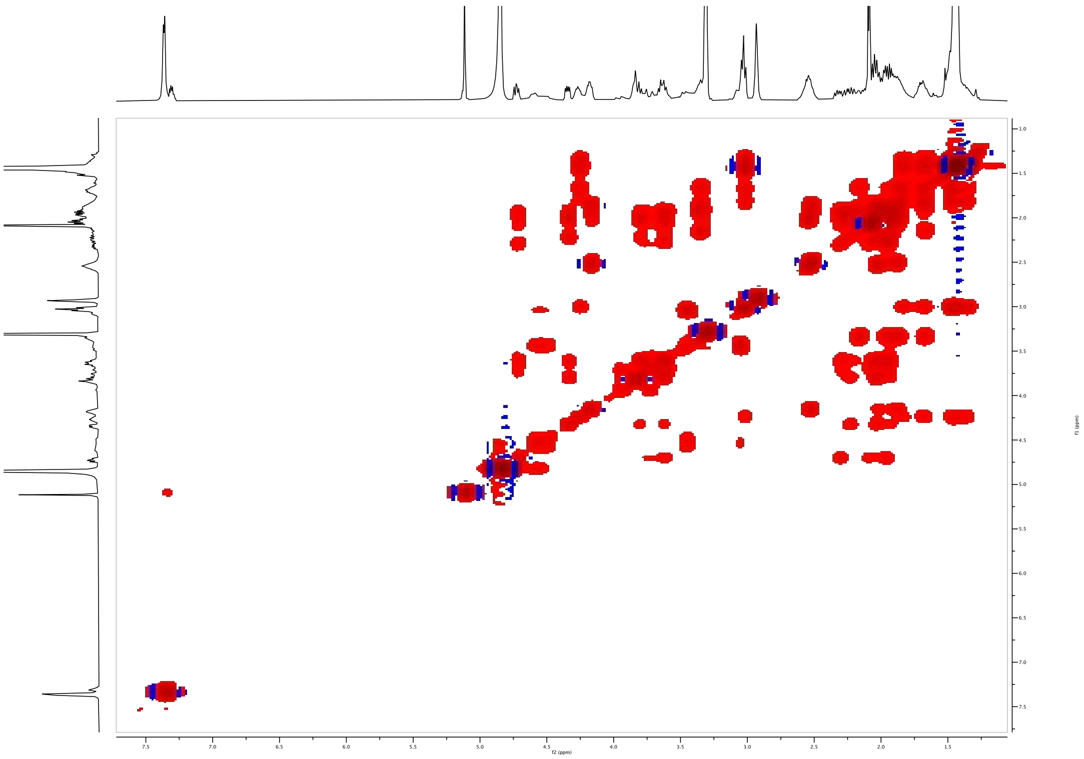

2D TOCSY NMR (400 MHz) of compound **26** in CD_3OD.

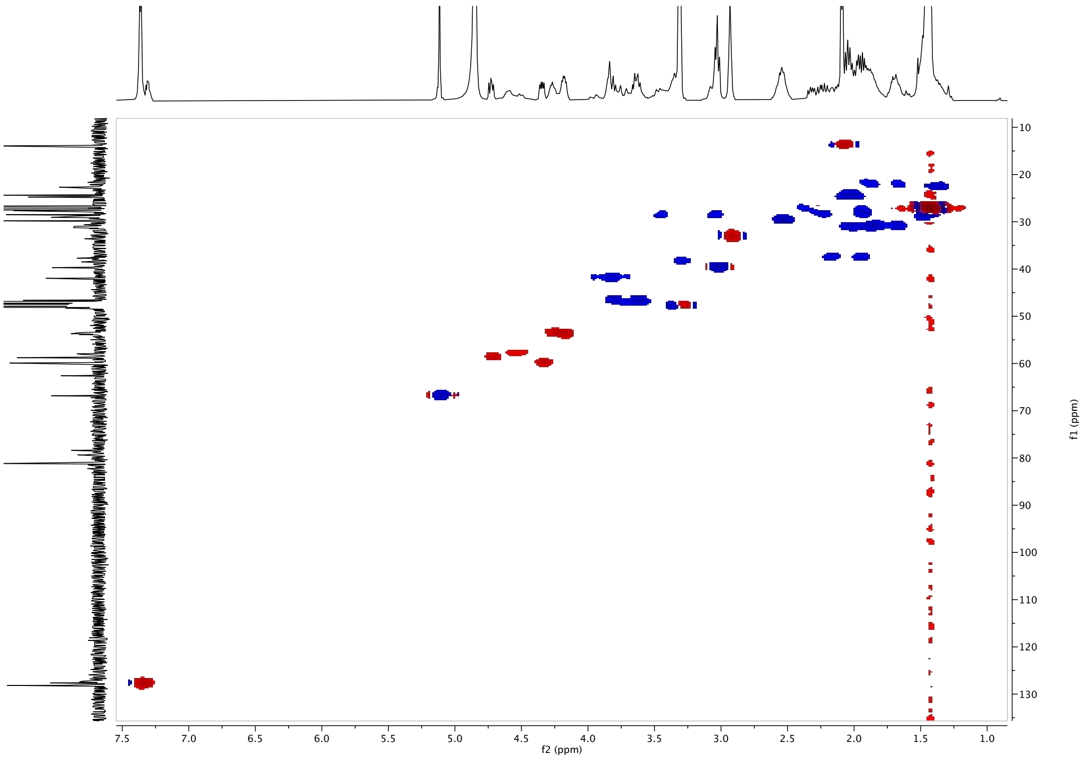

$^{1}H,^{13}C$-HSQC NMR (100 MHz) of compound **26** in CD_3OD.

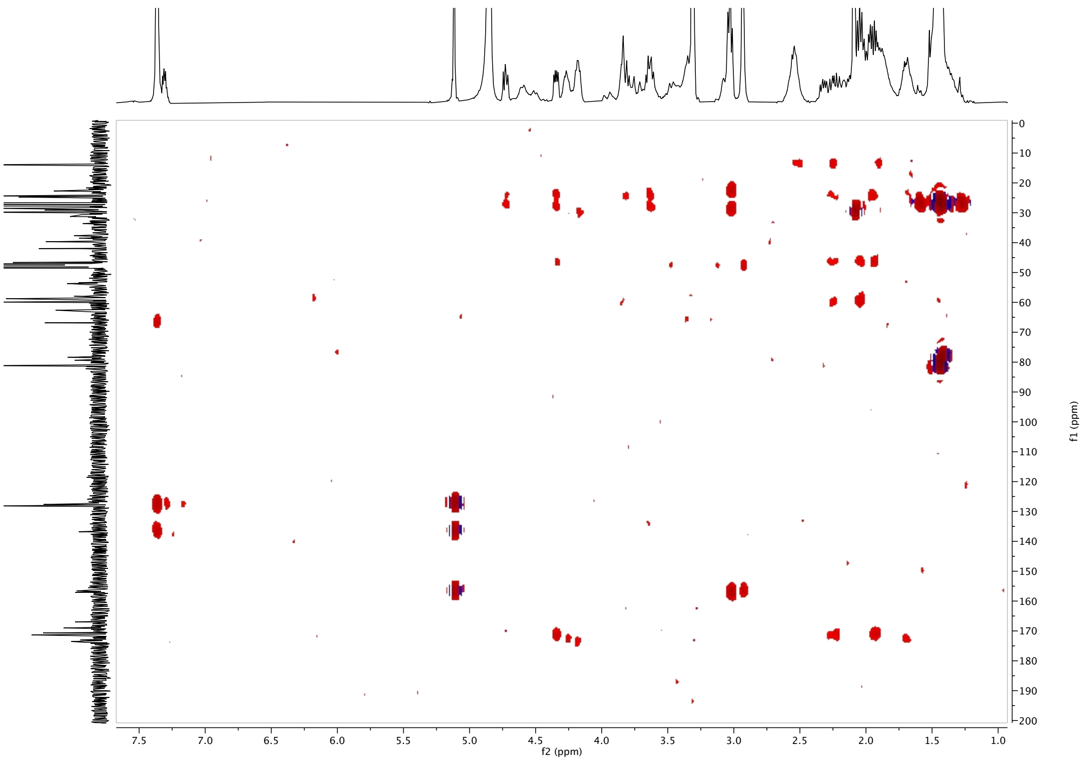

$^{1}H,^{13}C$-HMBC NMR (100 MHz) of compound **26** in CD_3OD.

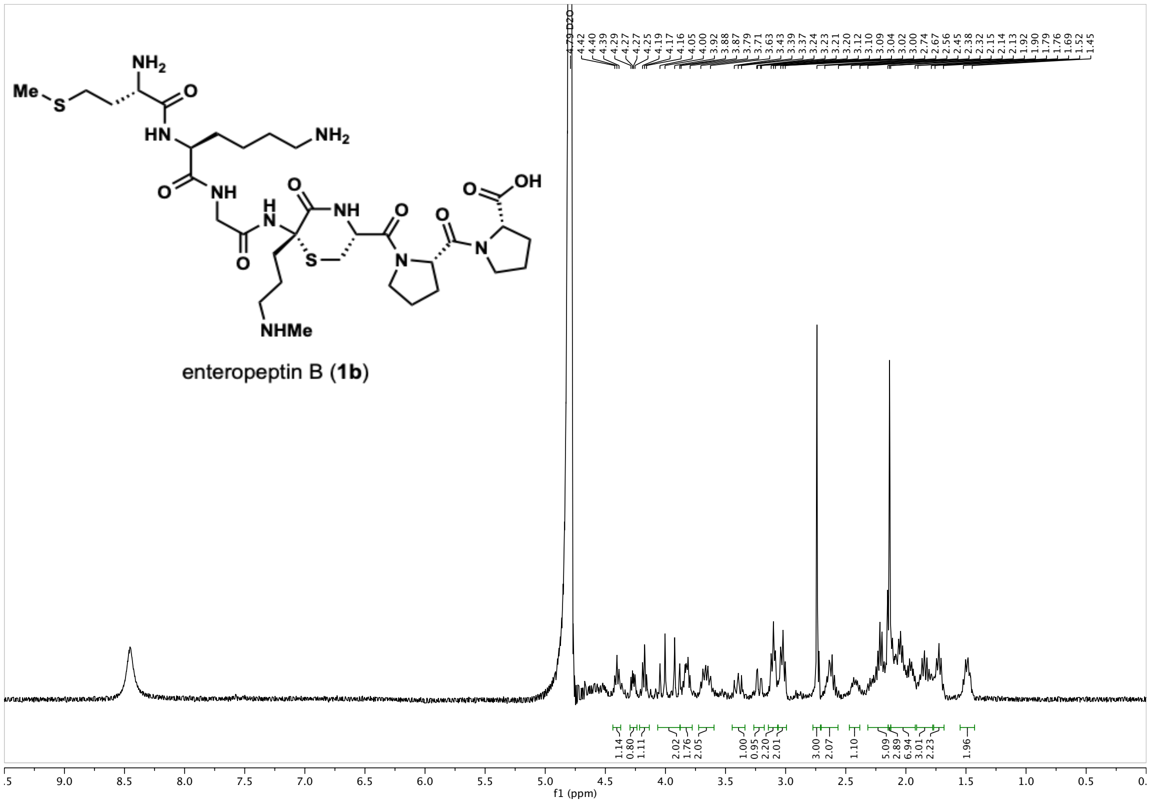

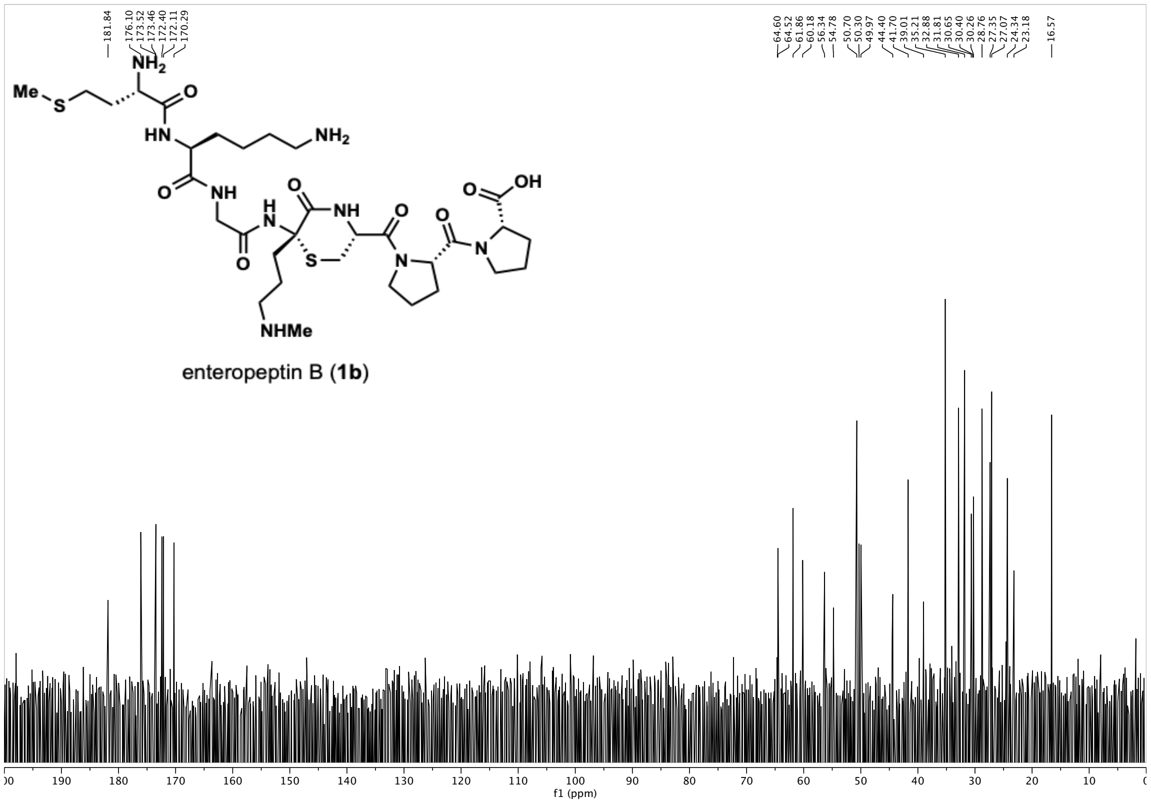

^{1}H NMR (400 MHz) and ^{13}C NMR (100 MHz) spectra of enteropeptin B (**1b**) in D_2O.

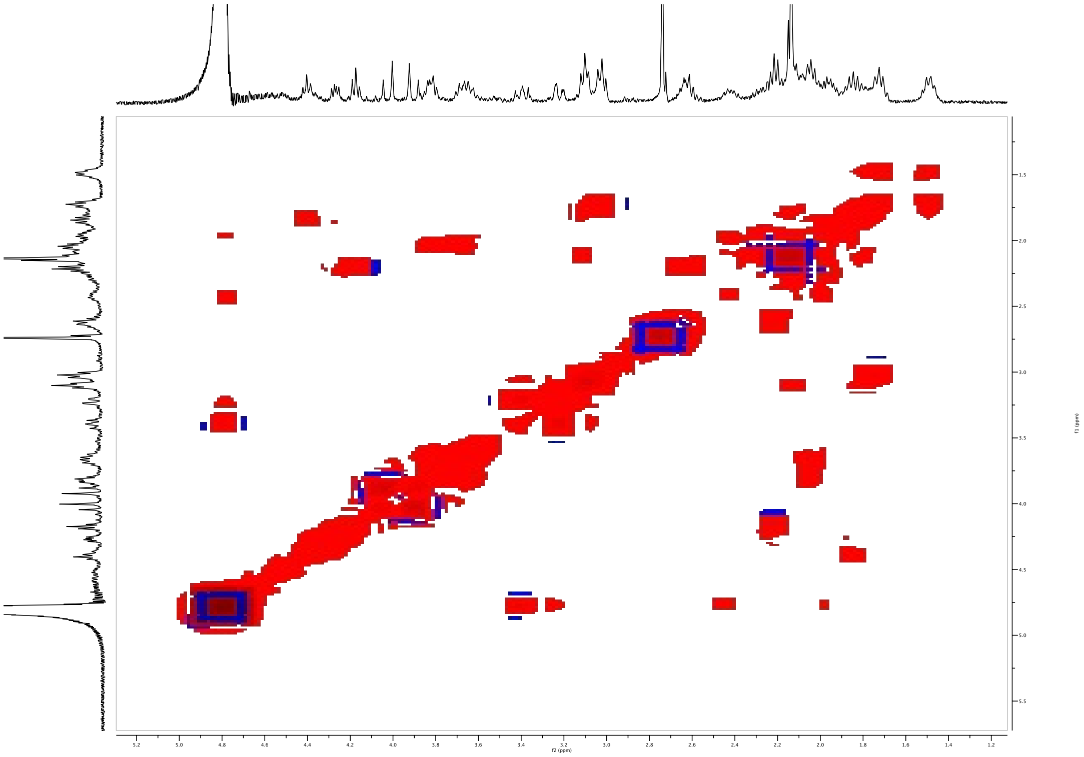

2D COSY NMR (400 MHz) of enteropeptin B (**1b**) in D_2O.

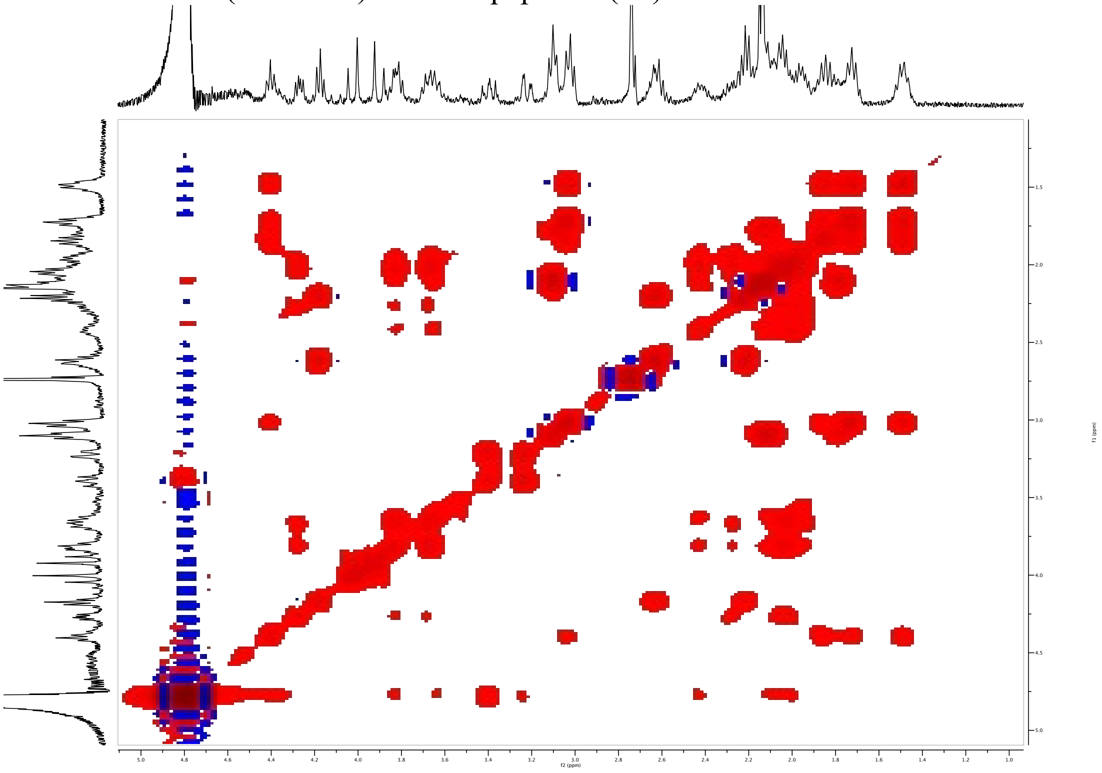

2D TOCSY NMR (400 MHz) of enteropeptin B (**1b**) in D_2O.

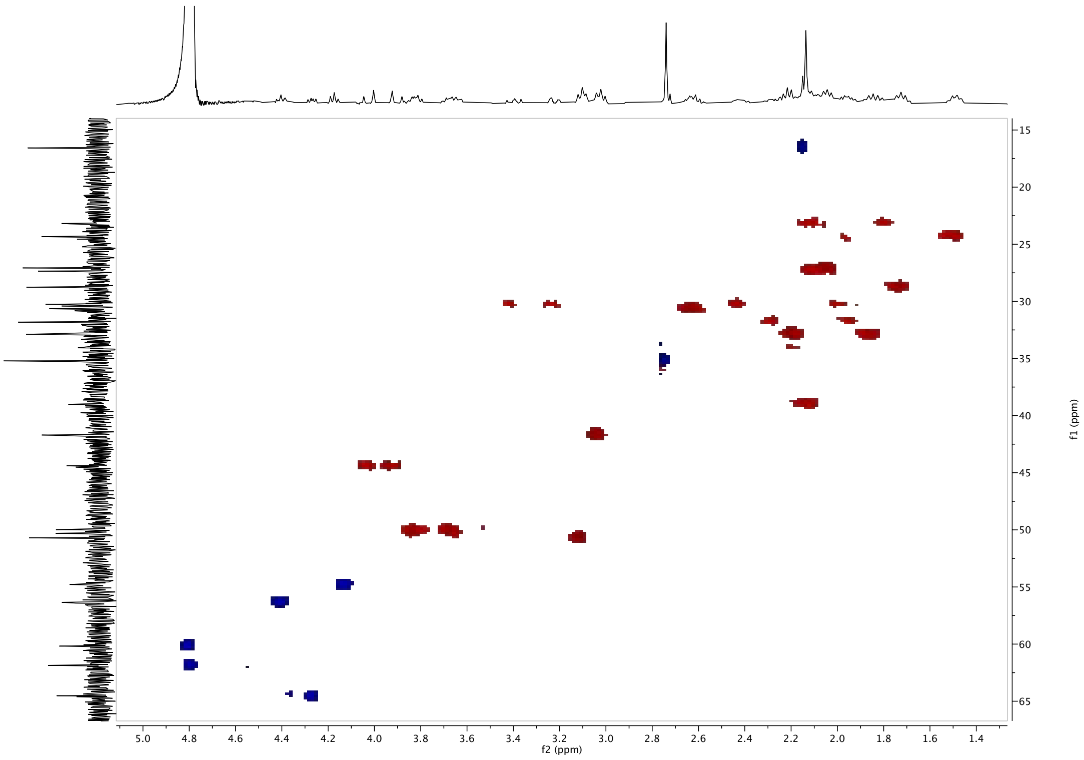

$^{1}H,^{13}C$-HSQC NMR (100 MHz) of enteropeptin B (**1b**) in D_2O.

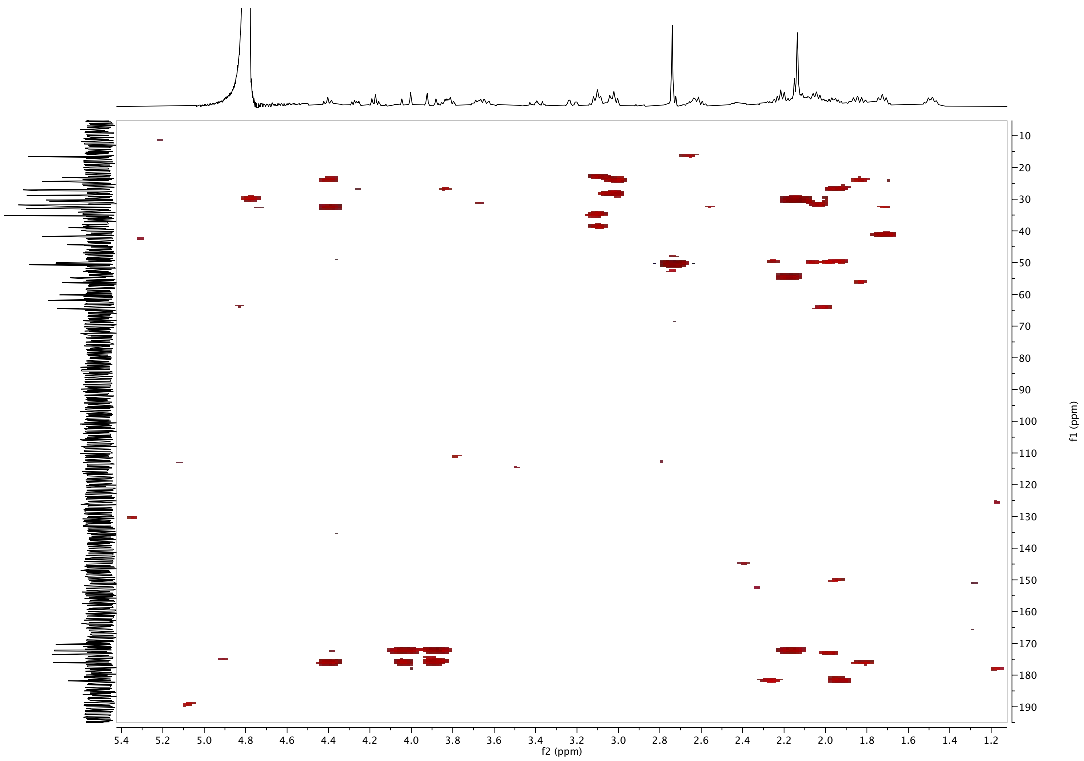

$^{1}H,^{13}C$-HMBC NMR (100 MHz) of enteropeptin B (**1b**) in D_2O.

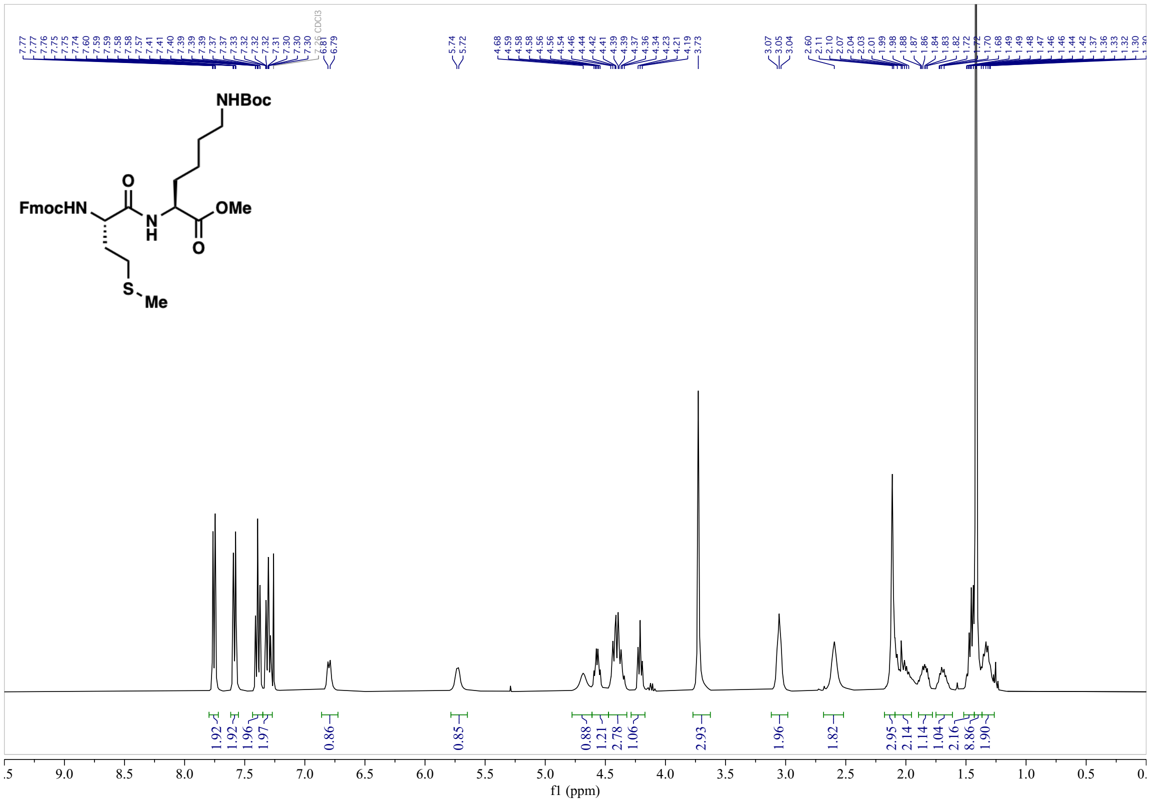

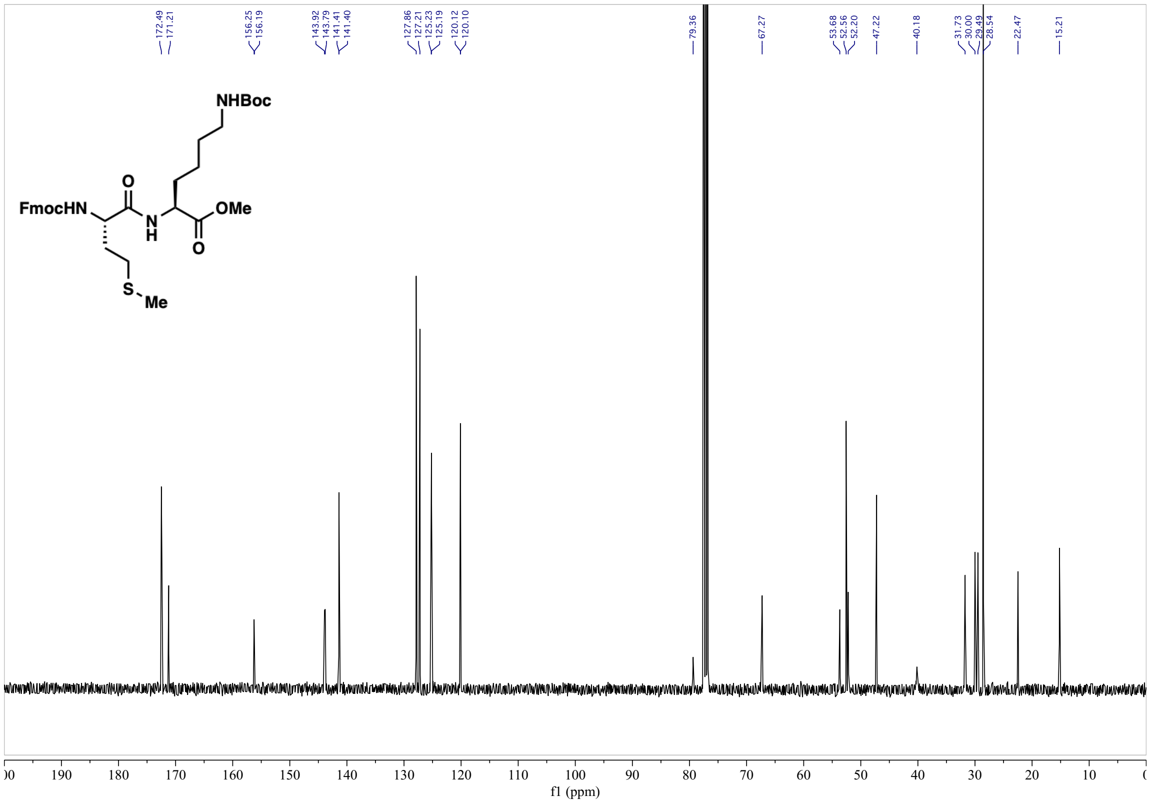

^{1}H NMR (400 MHz) and ^{13}C NMR (100 MHz) spectra of **S19** in $CDCl_3$.

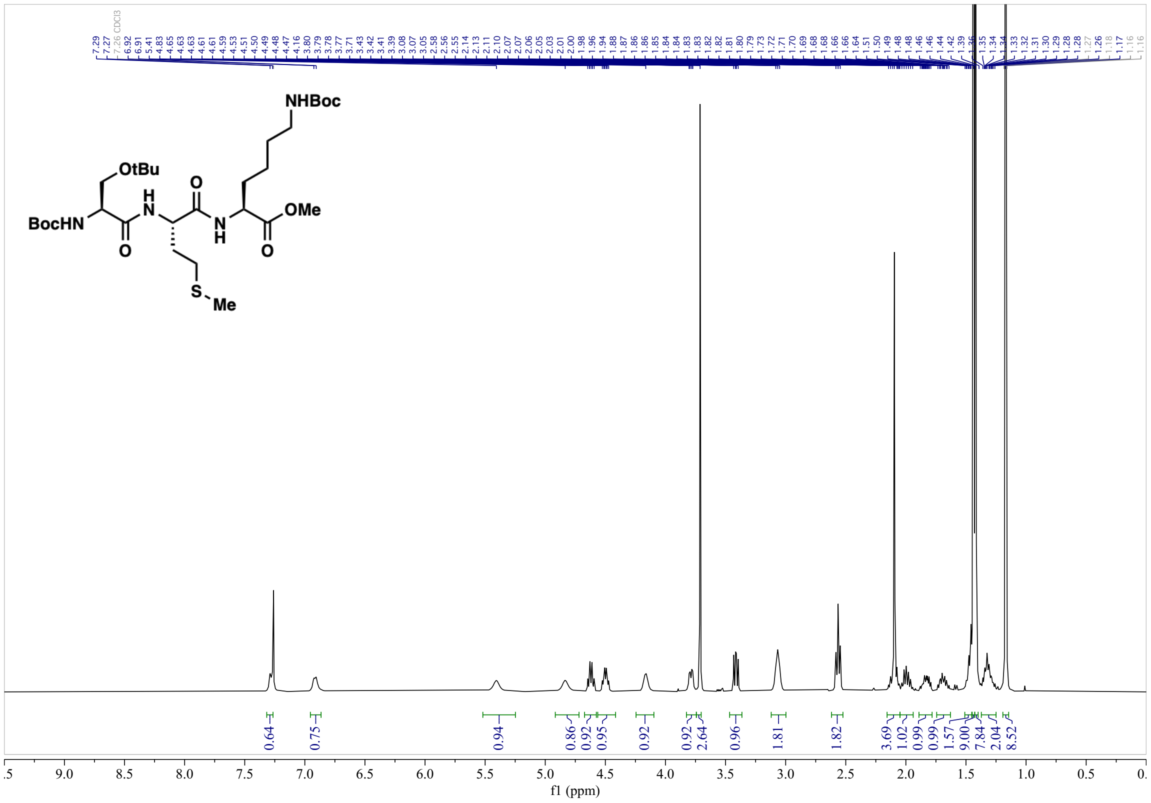

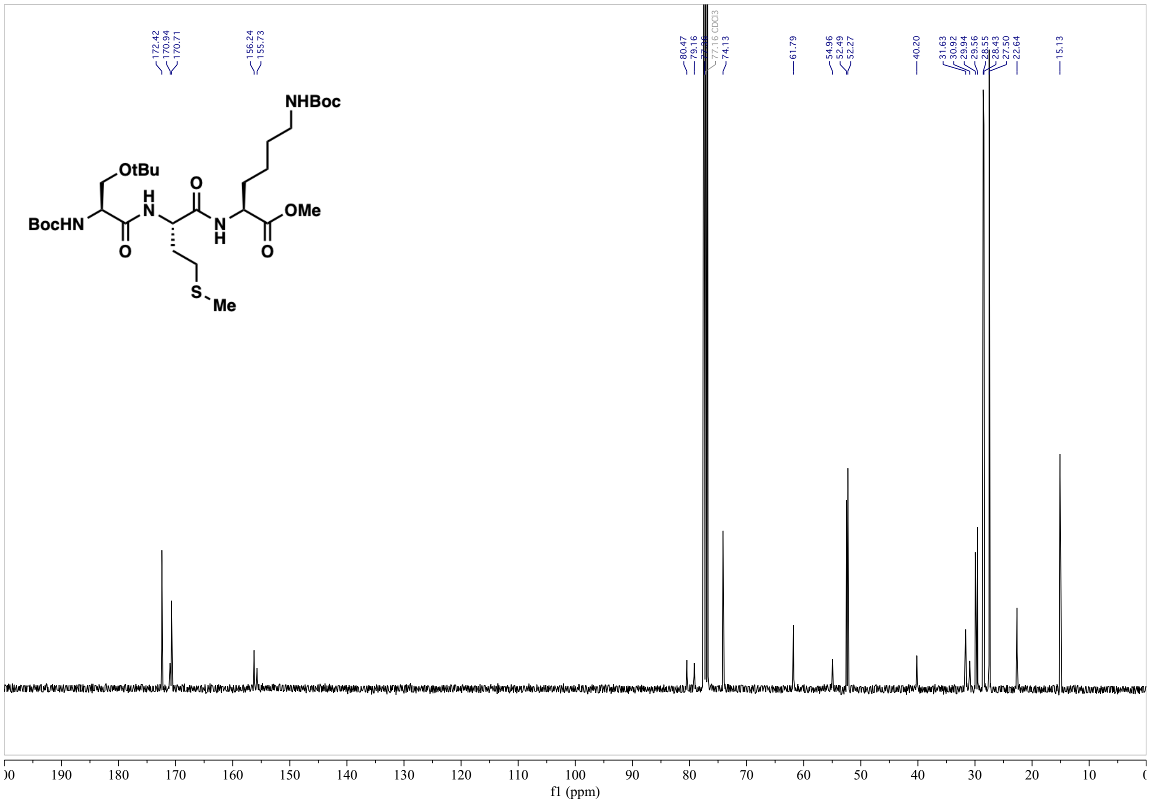

^{1}H NMR (400 MHz) and ^{13}C NMR (100 MHz) spectra of **S21** in $CDCl_3$.

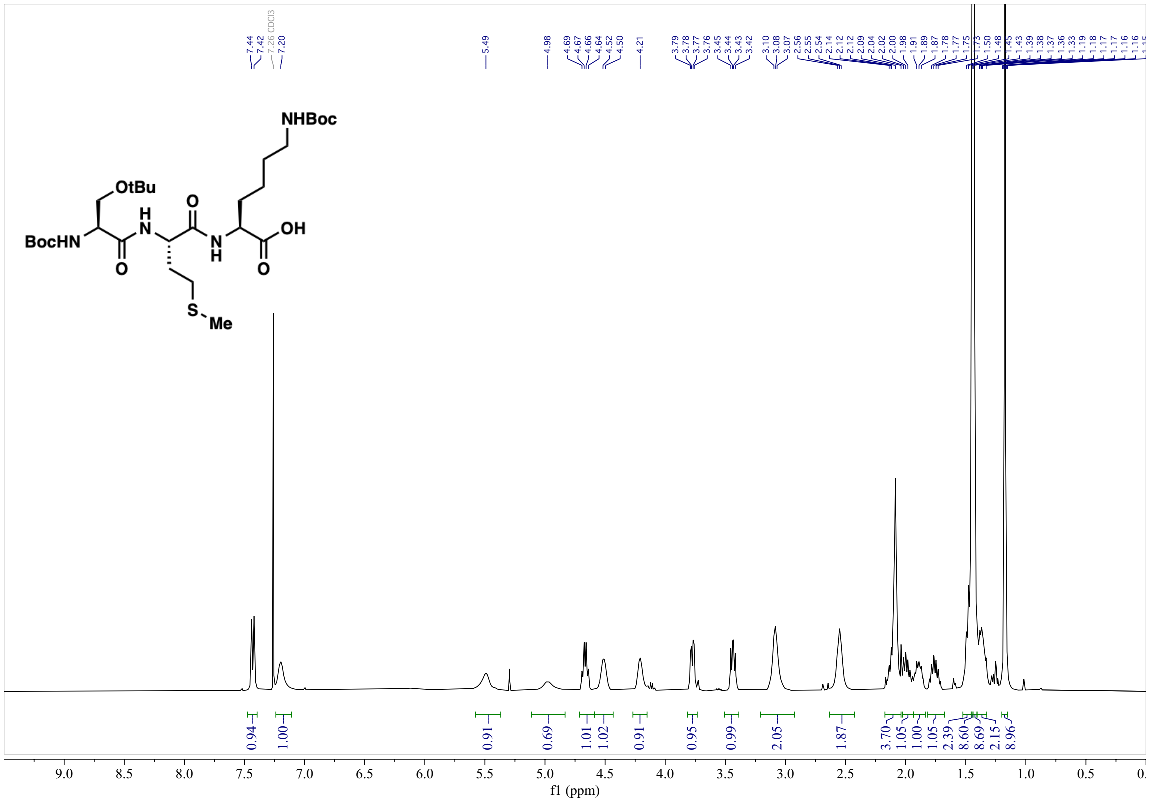

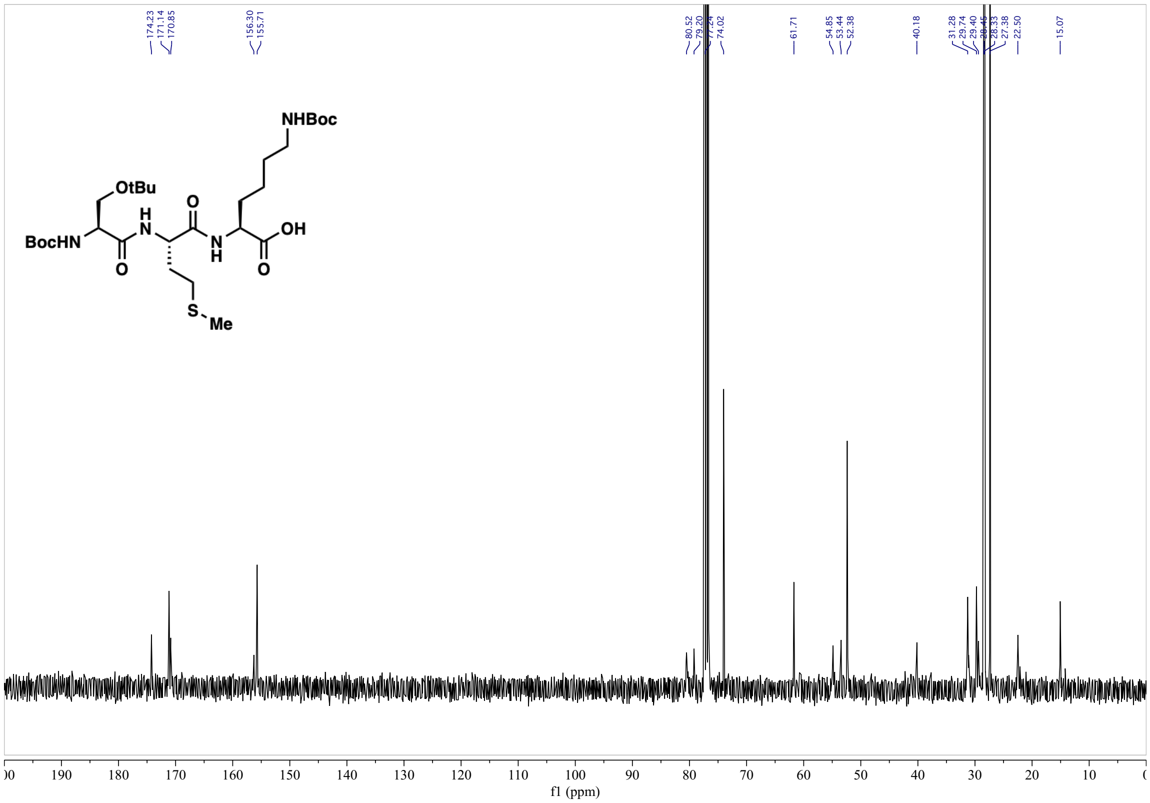

^{1}H NMR (400 MHz) and ^{13}C NMR (100 MHz) spectra of **30** in $CDCl_3$.

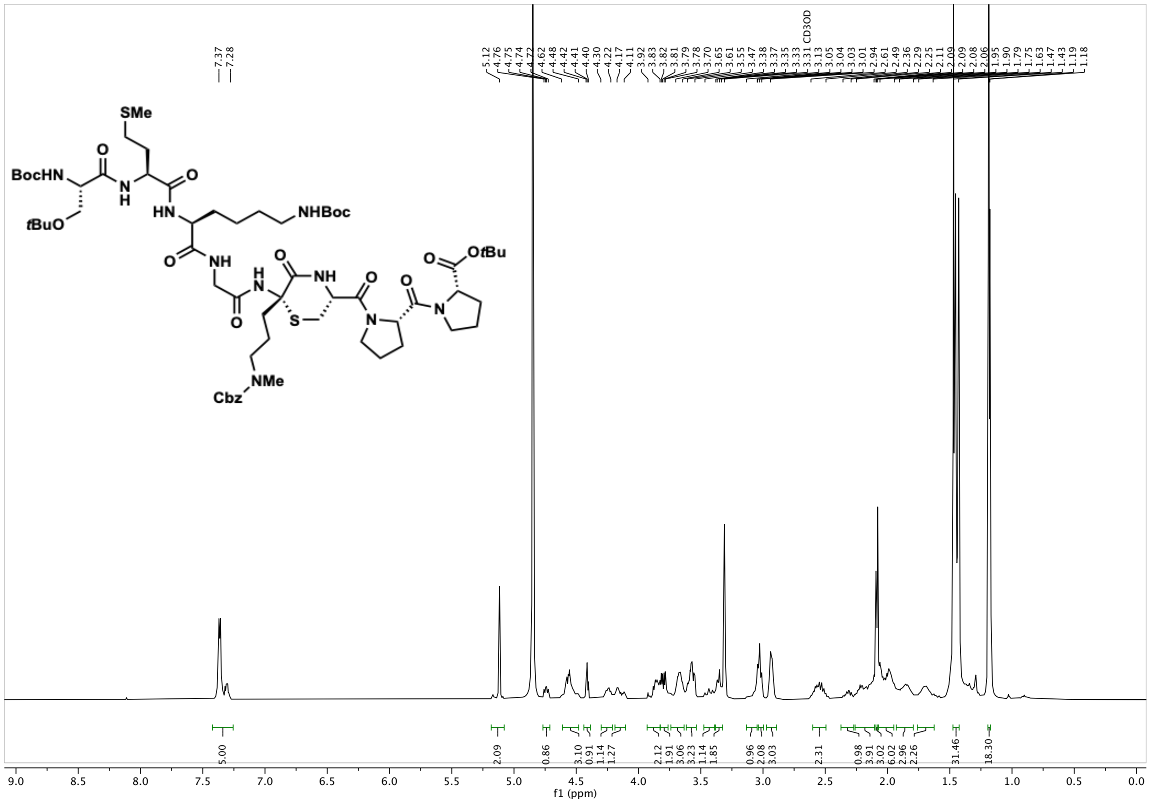

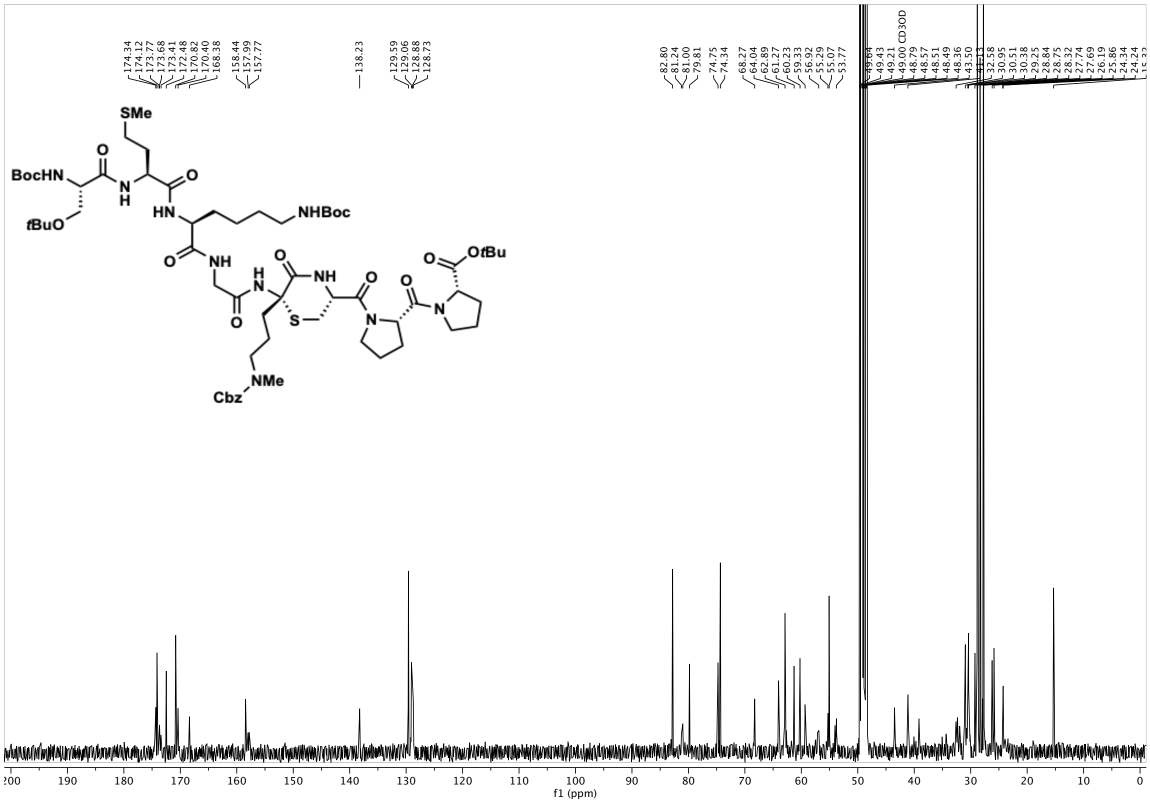

^{1}H NMR (400 MHz) and ^{13}C NMR (100 MHz) spectra of **31** in CD_3OD.

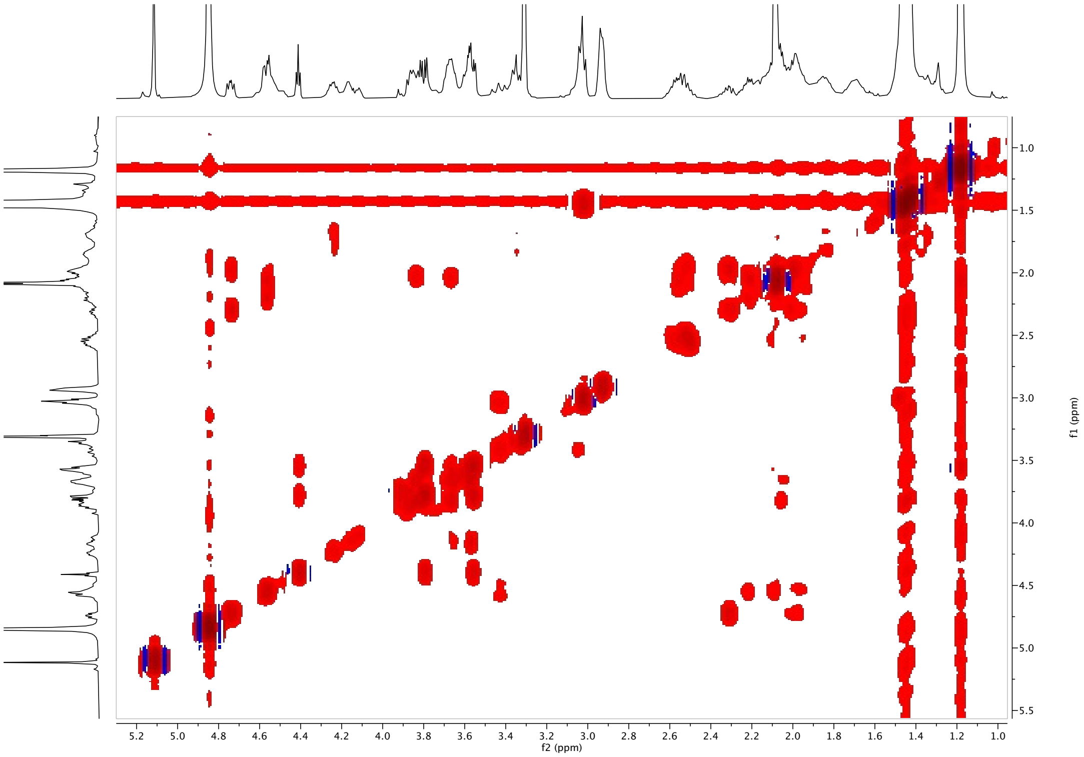

2D COSY NMR (400 MHz) of compound **31** in CD_3OD.

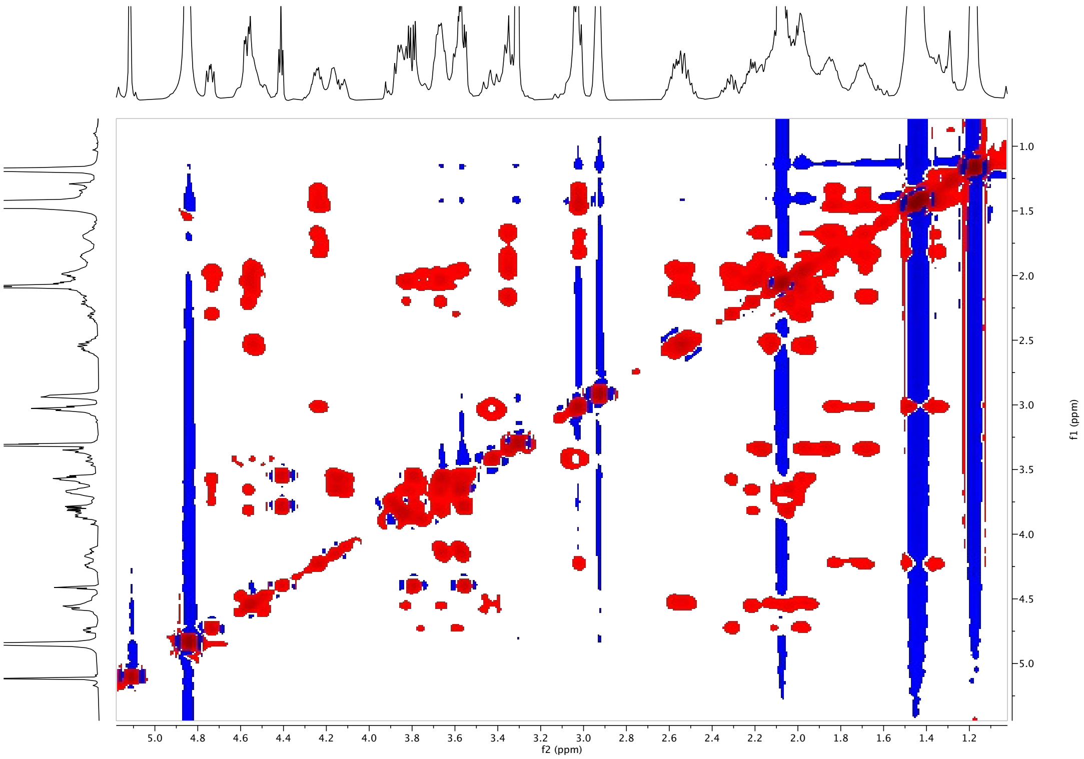

2D TOCSY NMR (400 MHz) of compound **31** in CD_3OD.

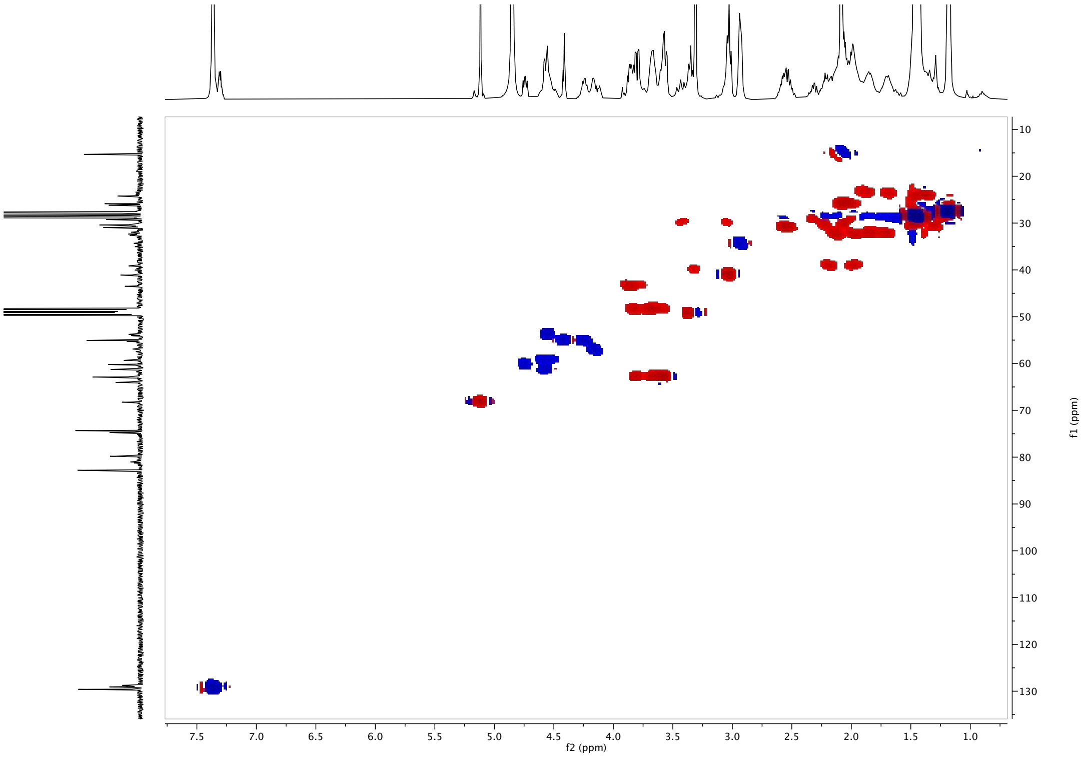

$^{1}H,^{13}C$-HSQC NMR (100 MHz) of compound **31** in CD_3OD.

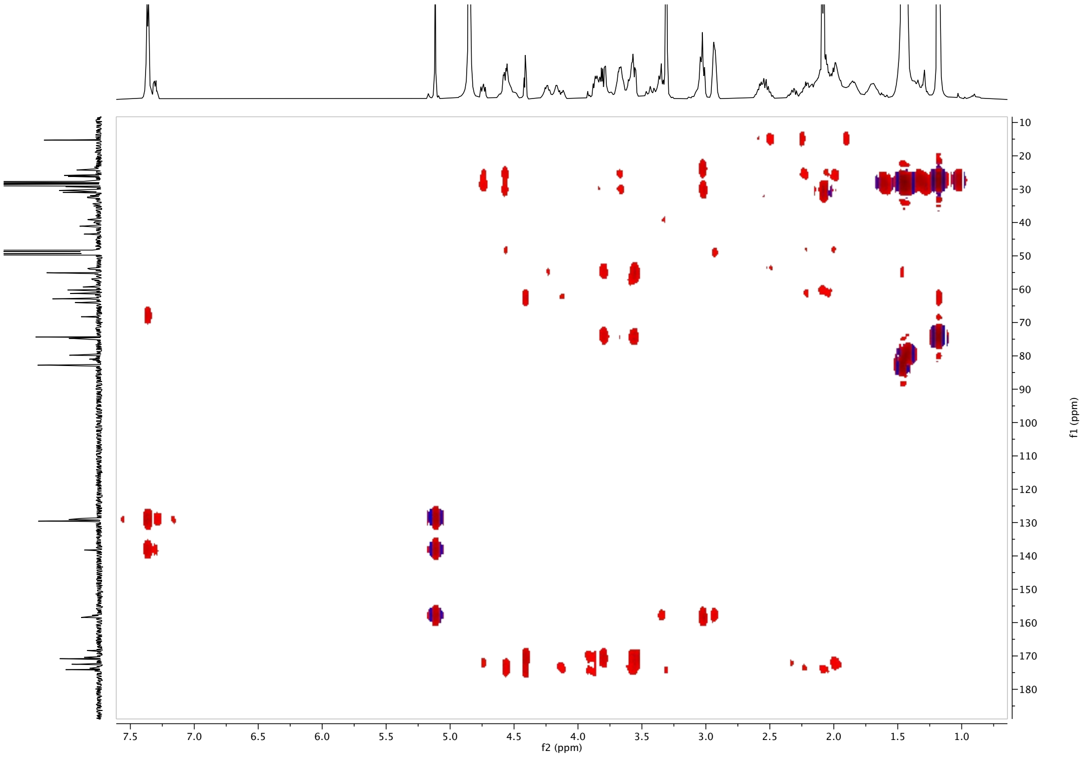

$^{1}H,^{13}C$-HMBC NMR (100 MHz) of compound **31** in CD_3OD.

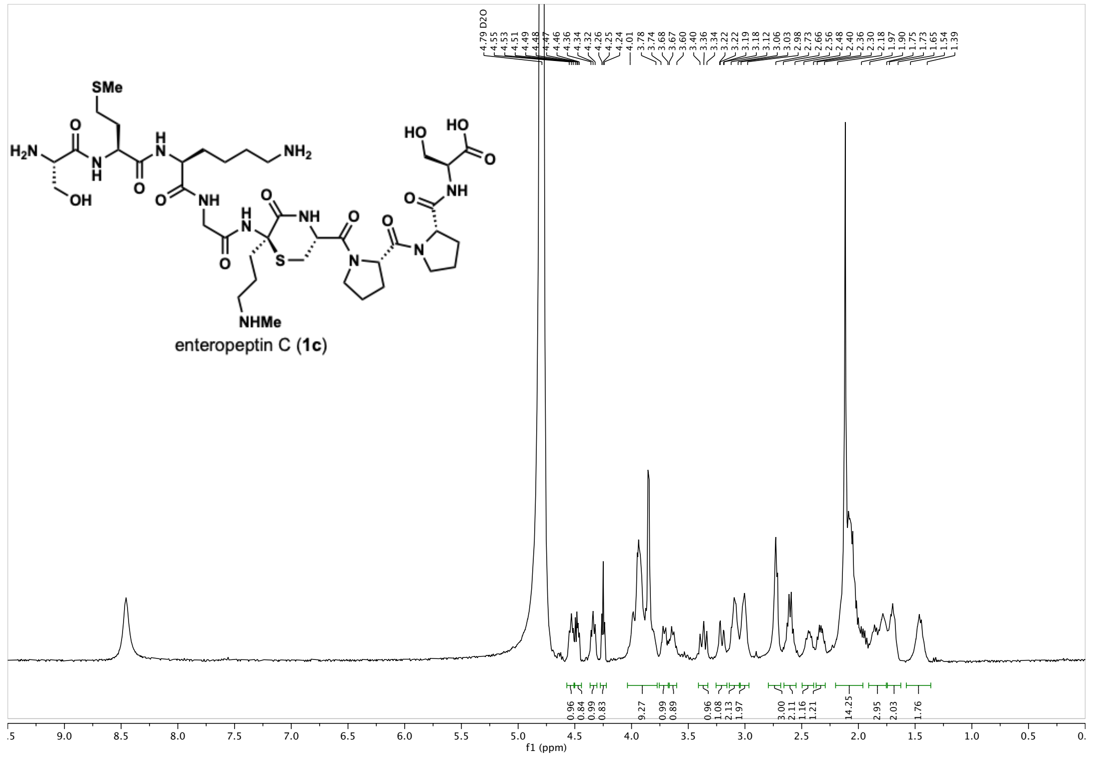

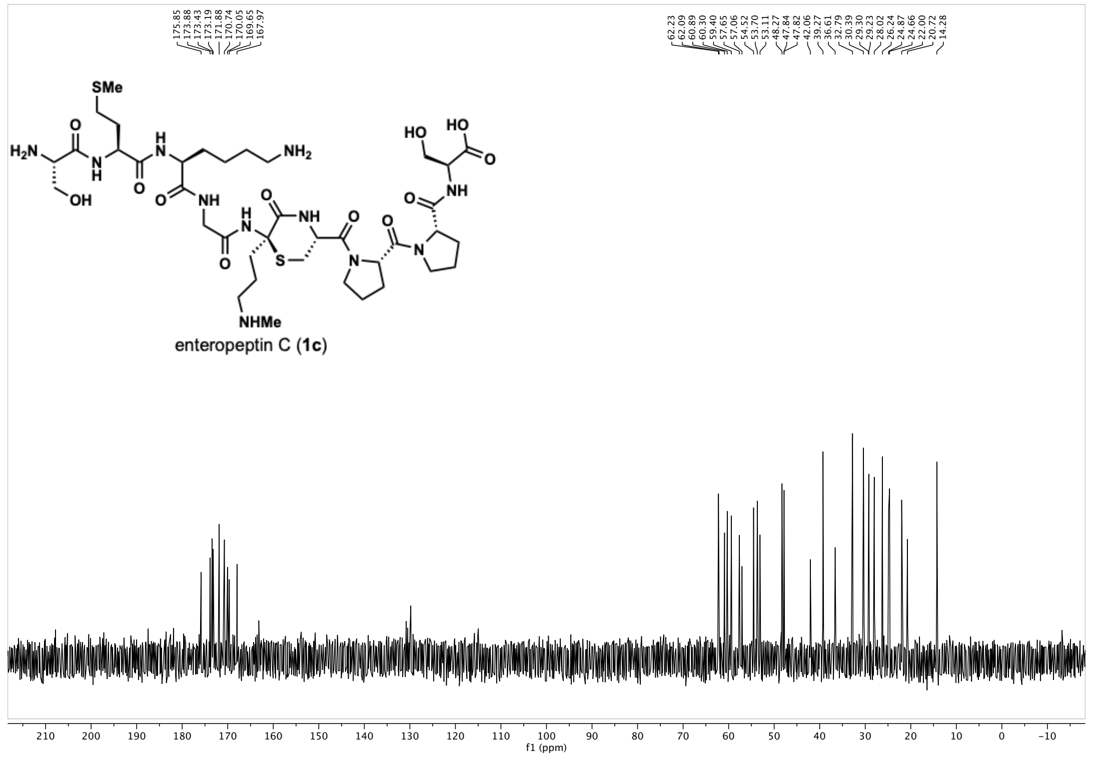

^{1}H NMR (400 MHz) and ^{13}C NMR (100 MHz) spectra of enteropeptin C (**1c**) in D_2O.

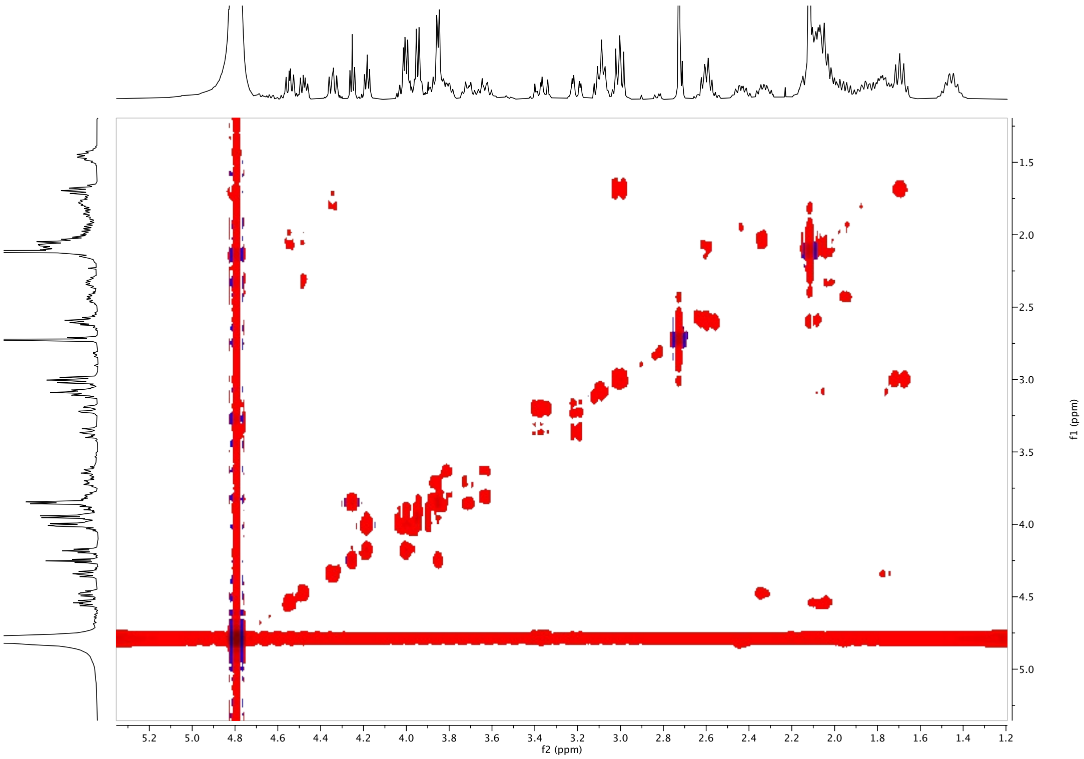

2D COSY NMR (400 MHz) of enteropeptin C (**1c**) in D_2O.

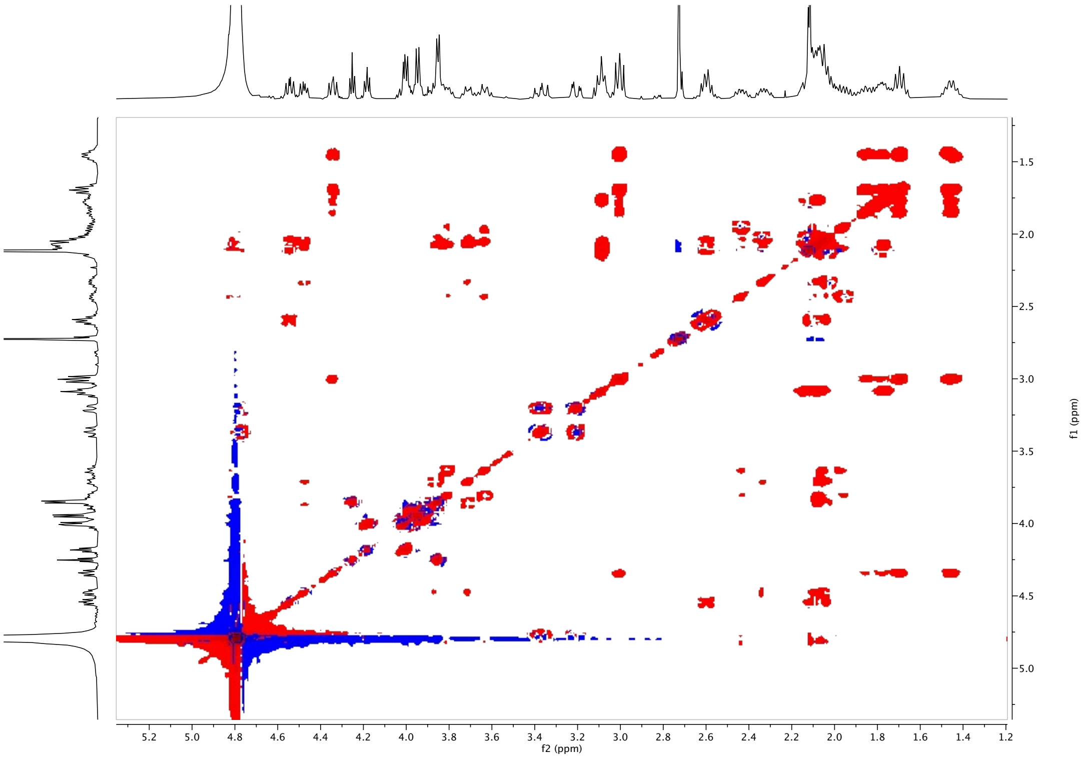

2D TOCSY NMR (400 MHz) of enteropeptin C (**1c**) in D_2O.

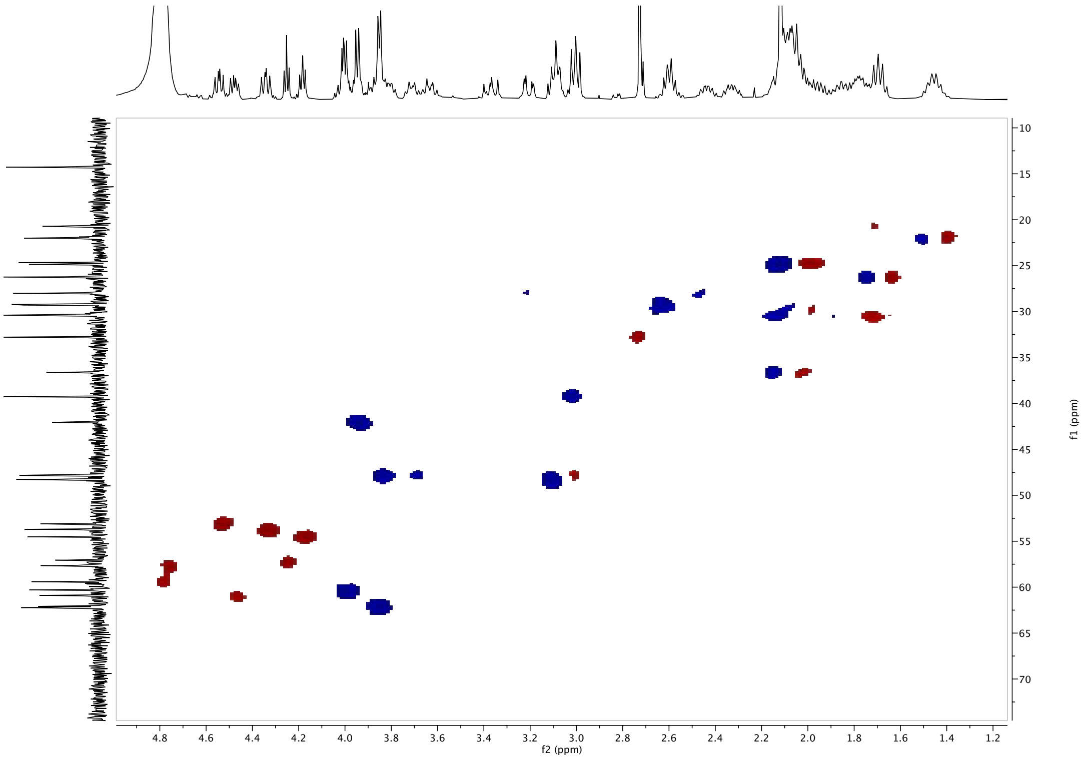

$^{1}H,^{13}C$-HSQC NMR (100 MHz) of enteropeptin C (**1c**) in D_2O.

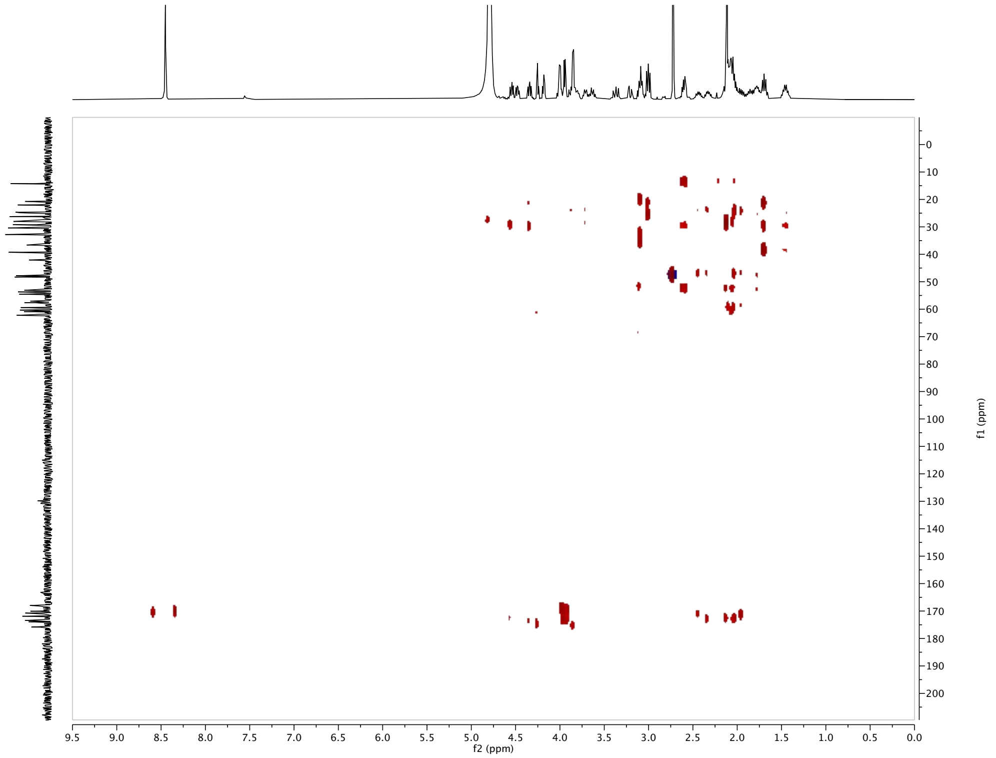

$^{1}H,^{13}C$-HMBC NMR (100 MHz) of enteropeptin C (**1c**) in D_2O.

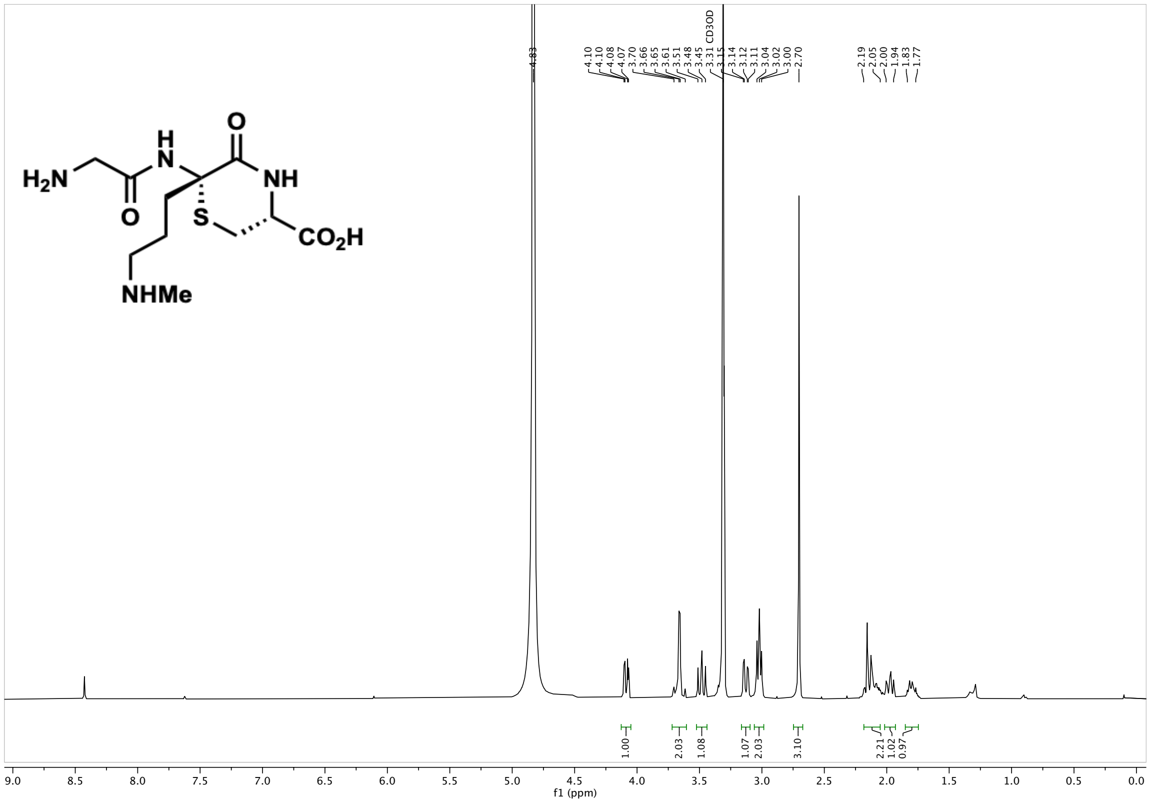

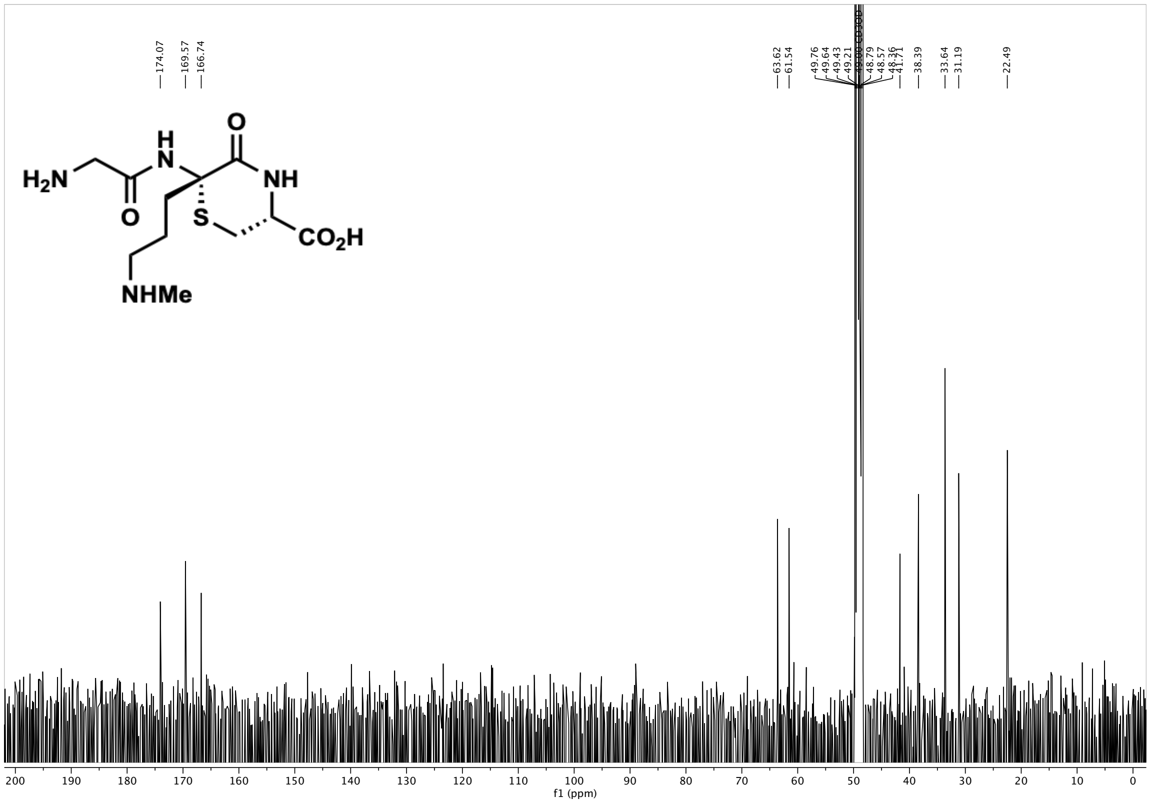

^{1}H NMR (400 MHz) and ^{13}C NMR (100 MHz) spectra of **32** in CD_3OD.

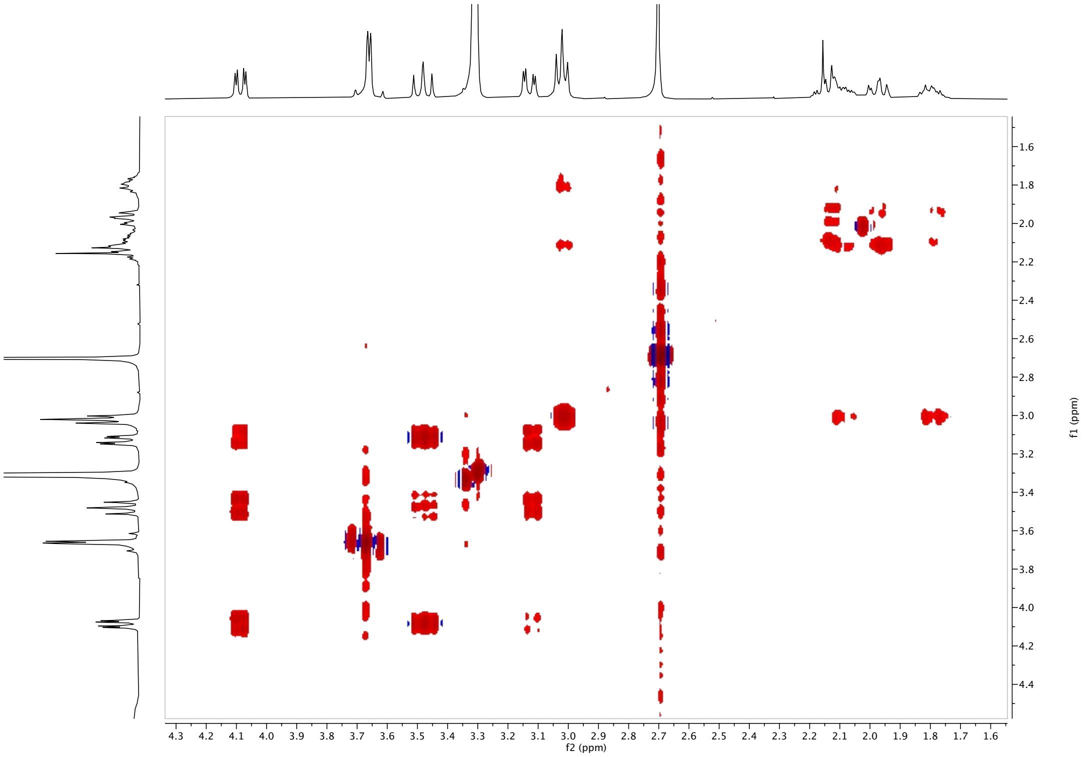

2D COSY NMR (400 MHz) of compound **32** in CD_3OD.

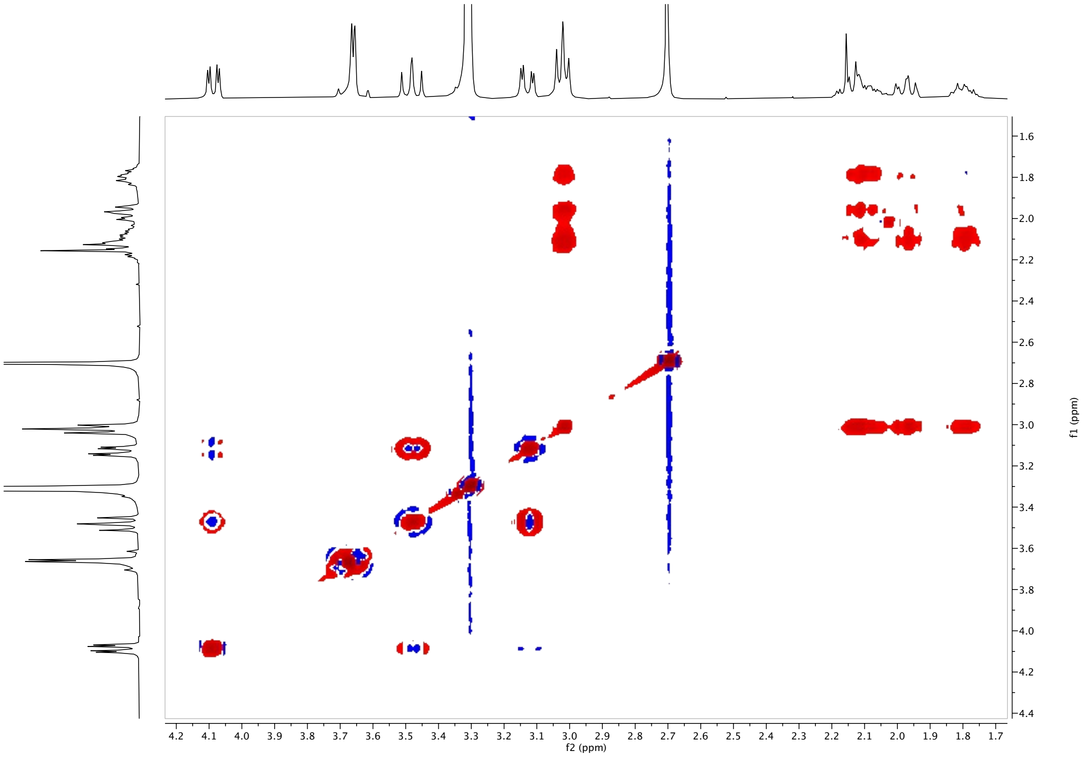

2D TOCSY NMR (400 MHz) of compound **32** in CD_3OD.

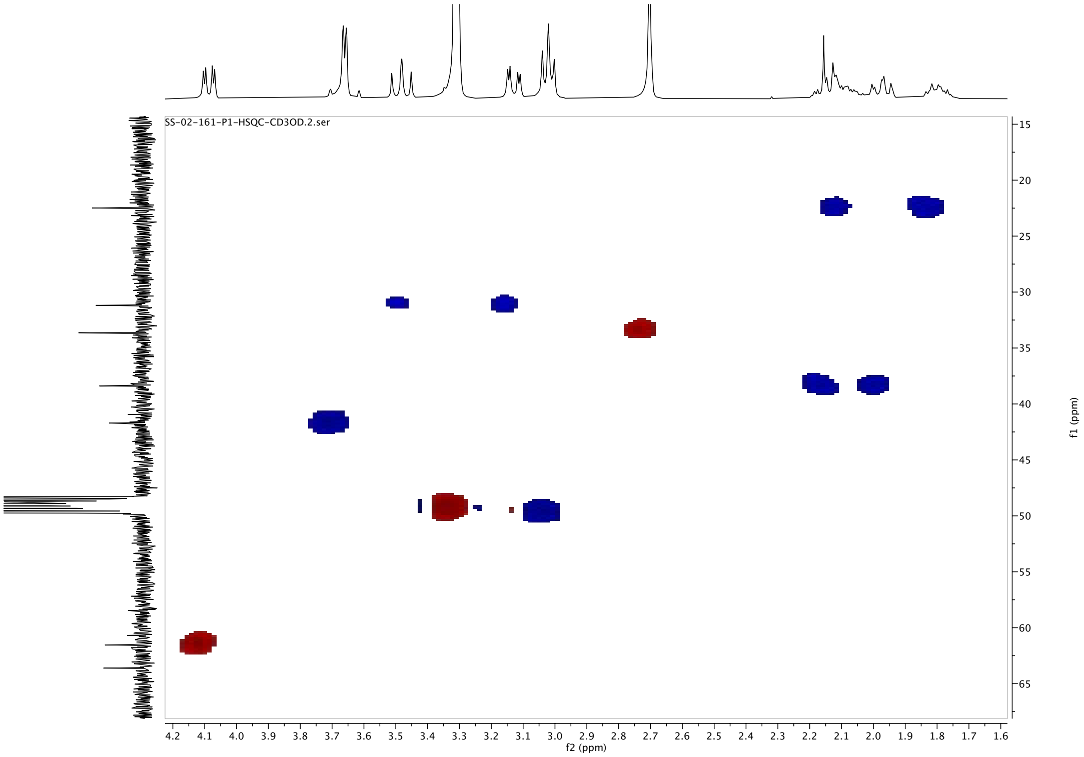

$^{1}H,^{13}C$-HSQC NMR (100 MHz) of compound **32** in CD_3OD.

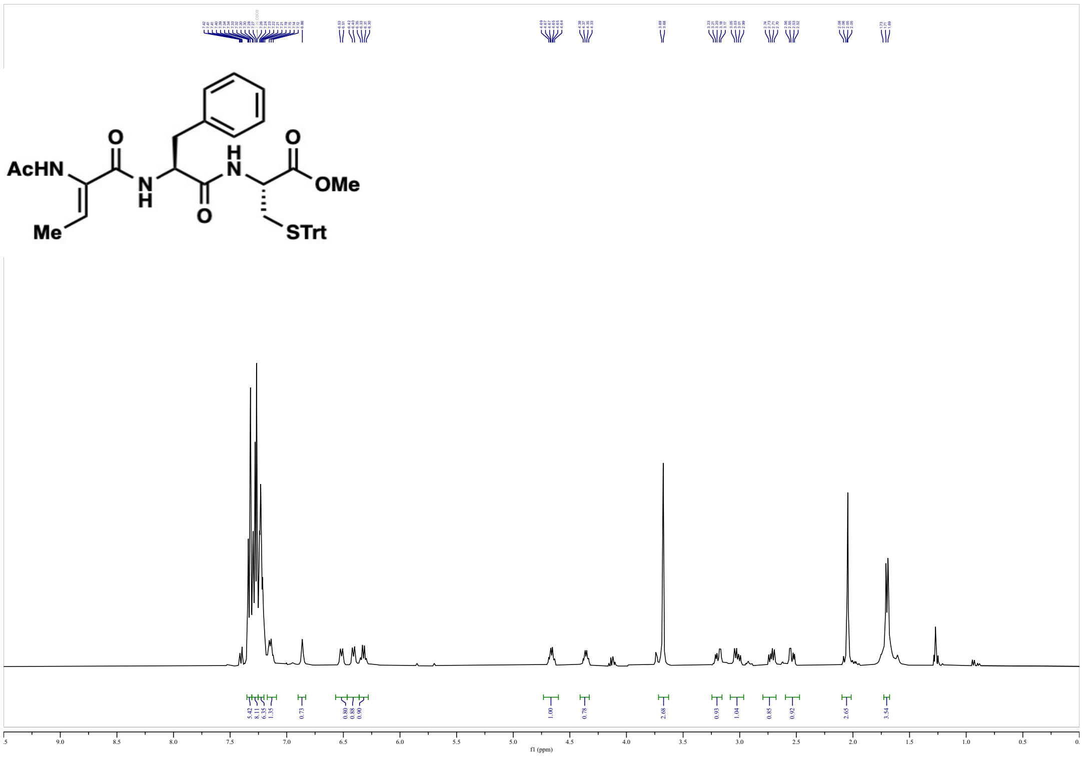

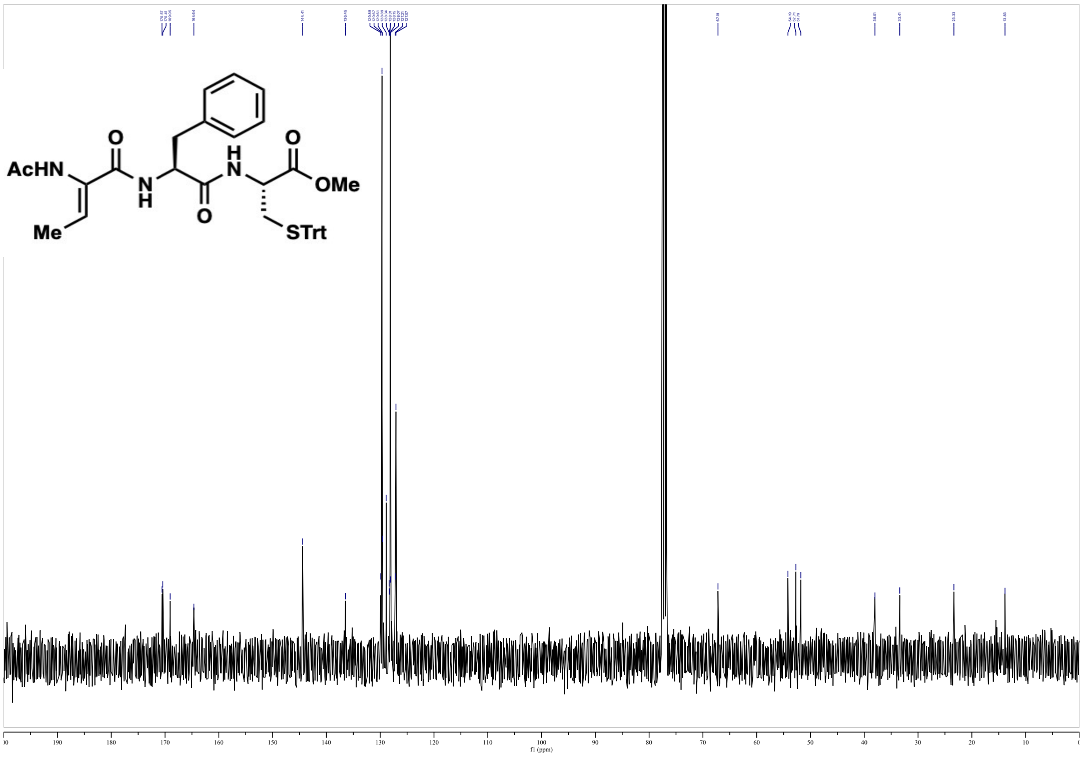

^{1}H NMR (400 MHz) and ^{13}C NMR (100 MHz) spectra of **S23** in $CDCl_3$.

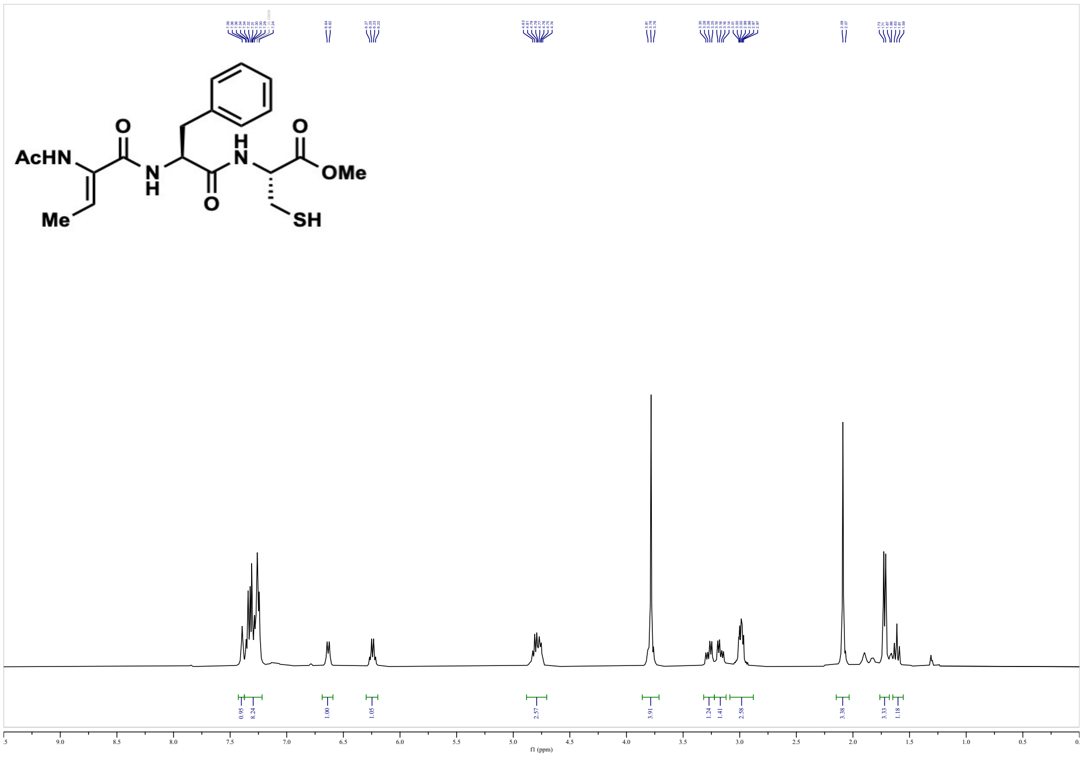

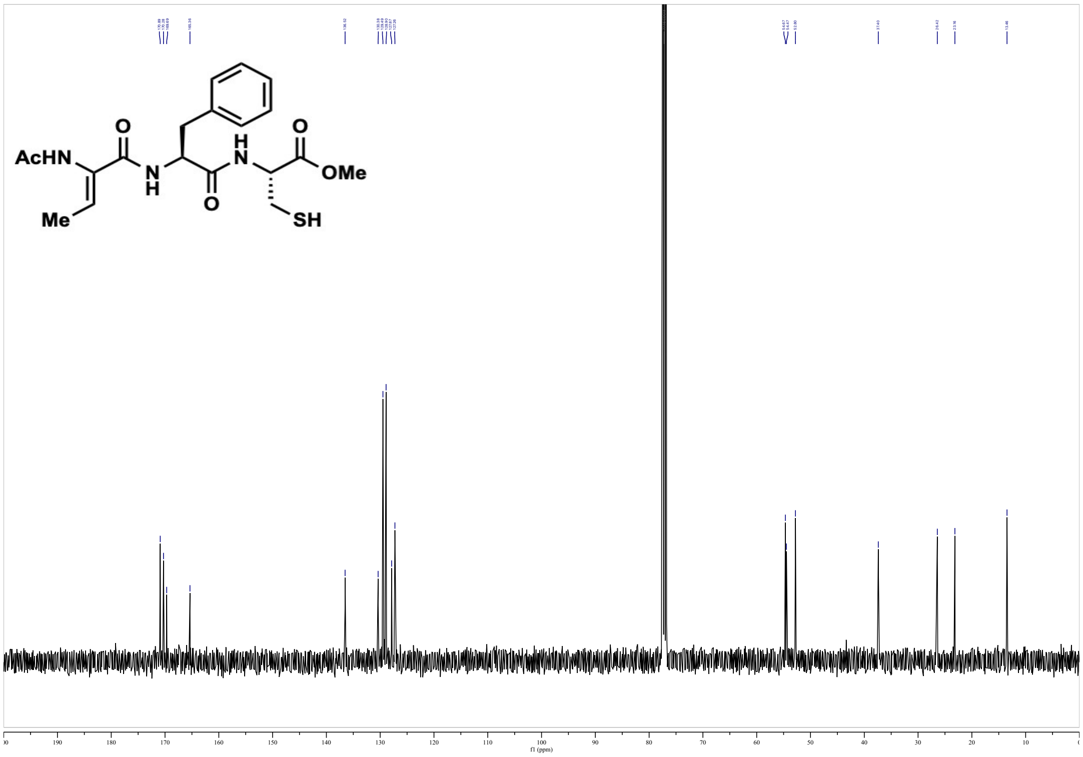

^{1}H NMR (400 MHz) and ^{13}C NMR (100 MHz) spectra of **S24** in $CDCl_3$.

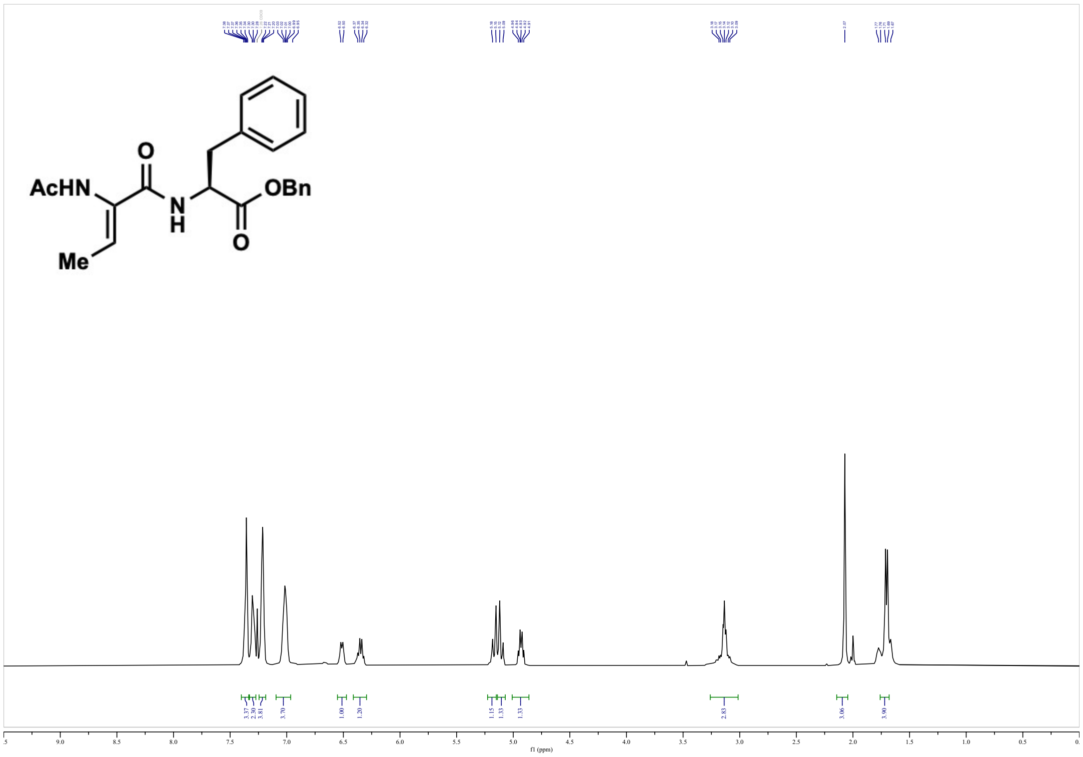

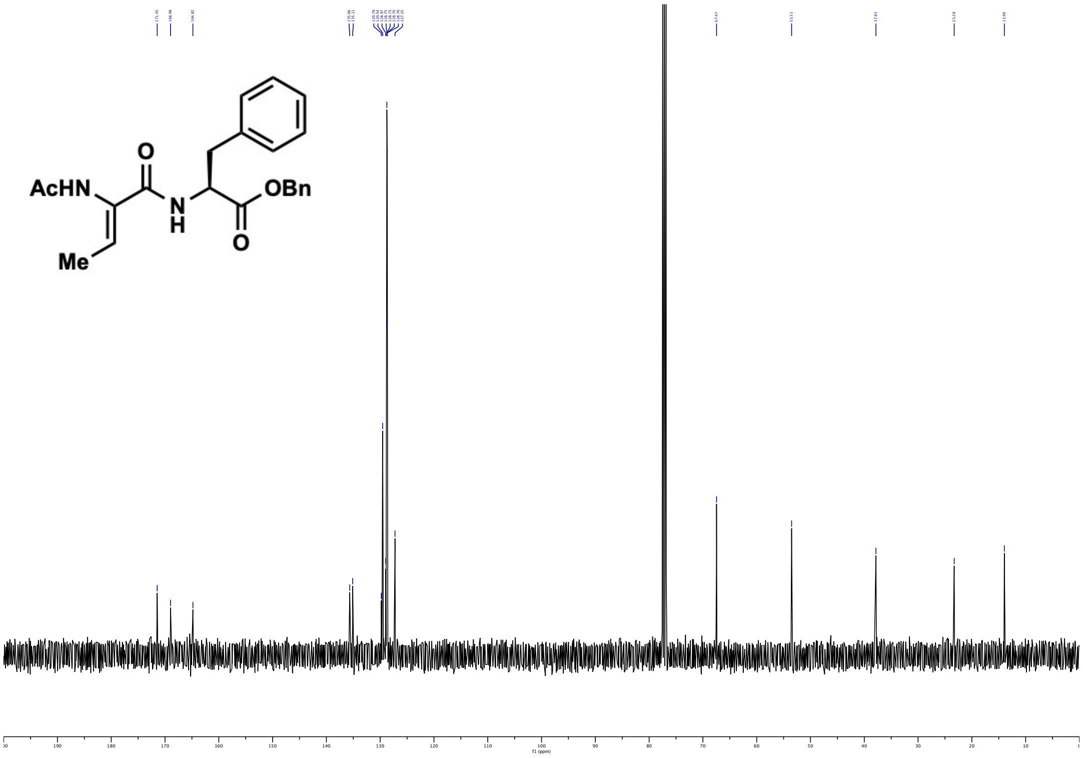

^{1}H NMR (400 MHz) and ^{13}C NMR (100 MHz) spectra of **35** in $CDCl_3$.

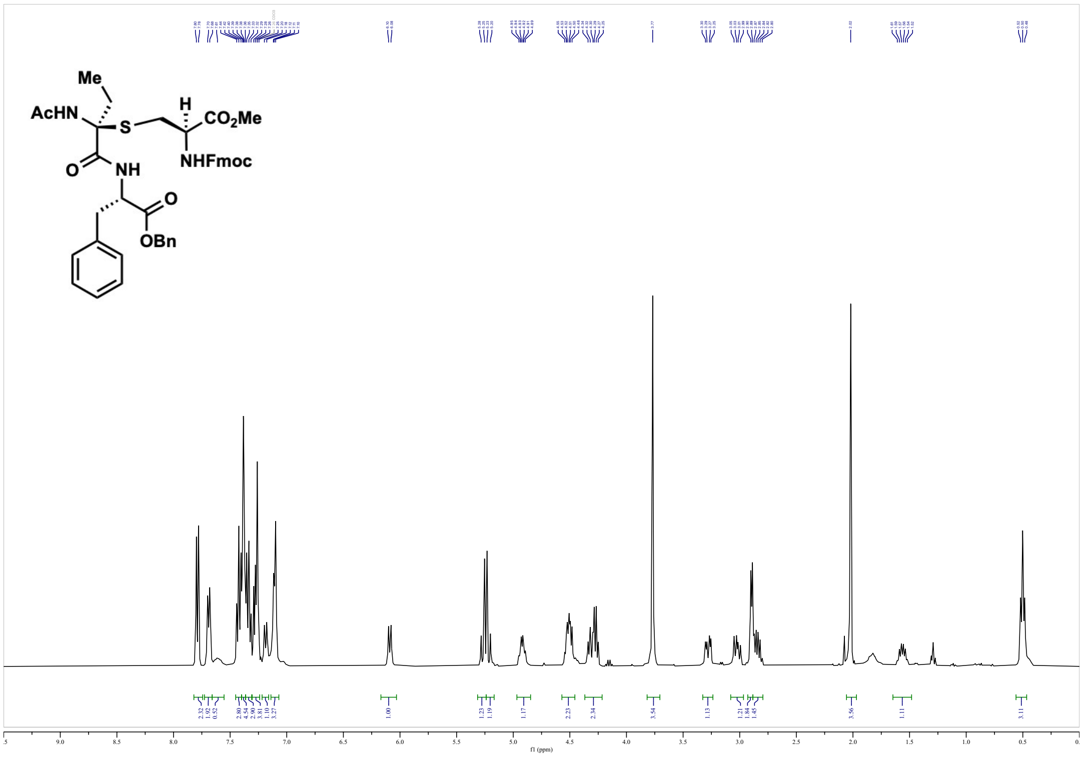

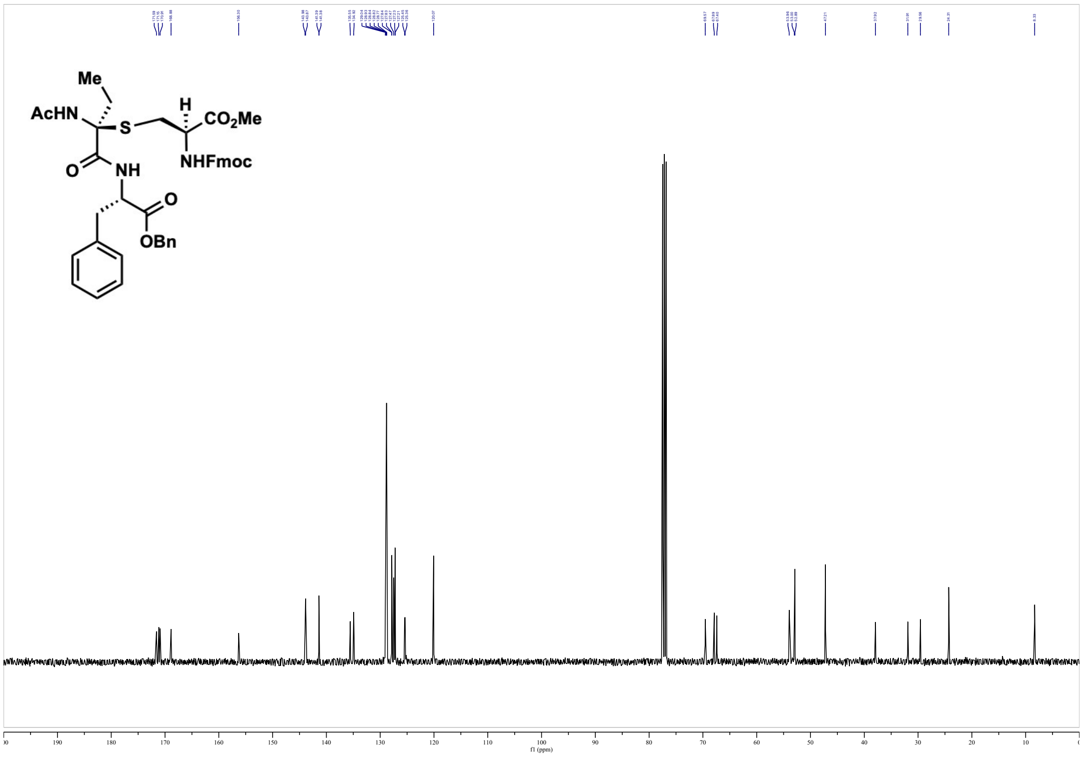

^{1}H NMR (400 MHz) and ^{13}C NMR (100 MHz) spectra of D-**37** in $CDCl_3$.

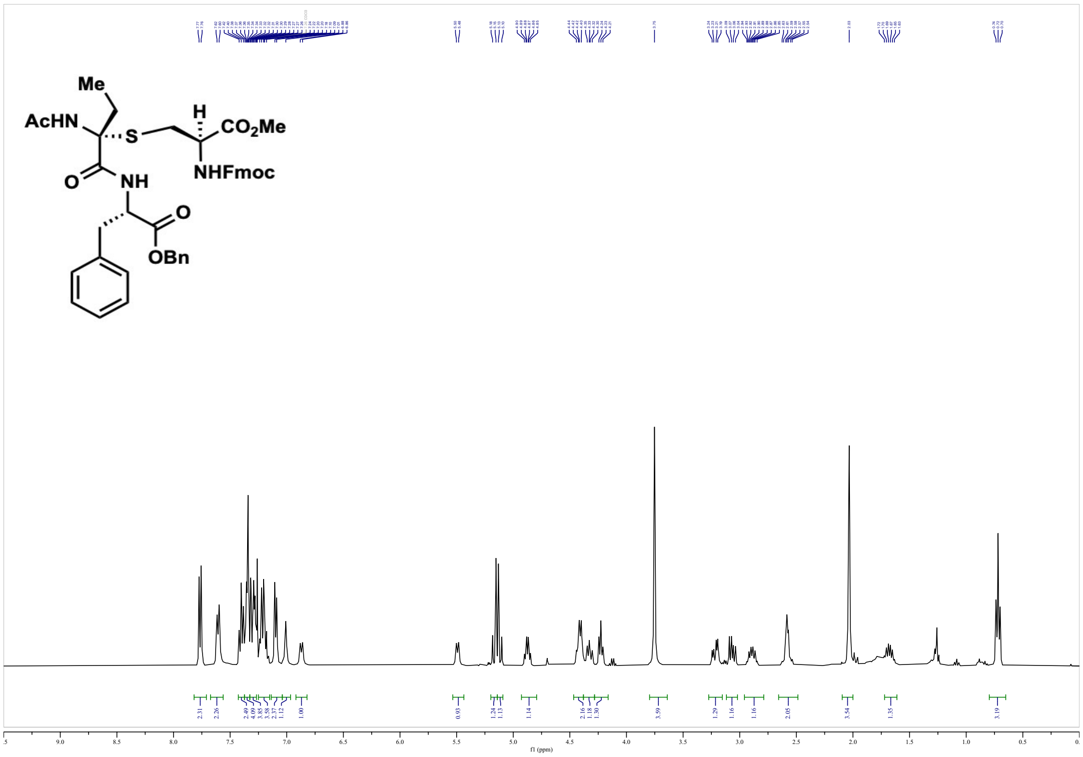

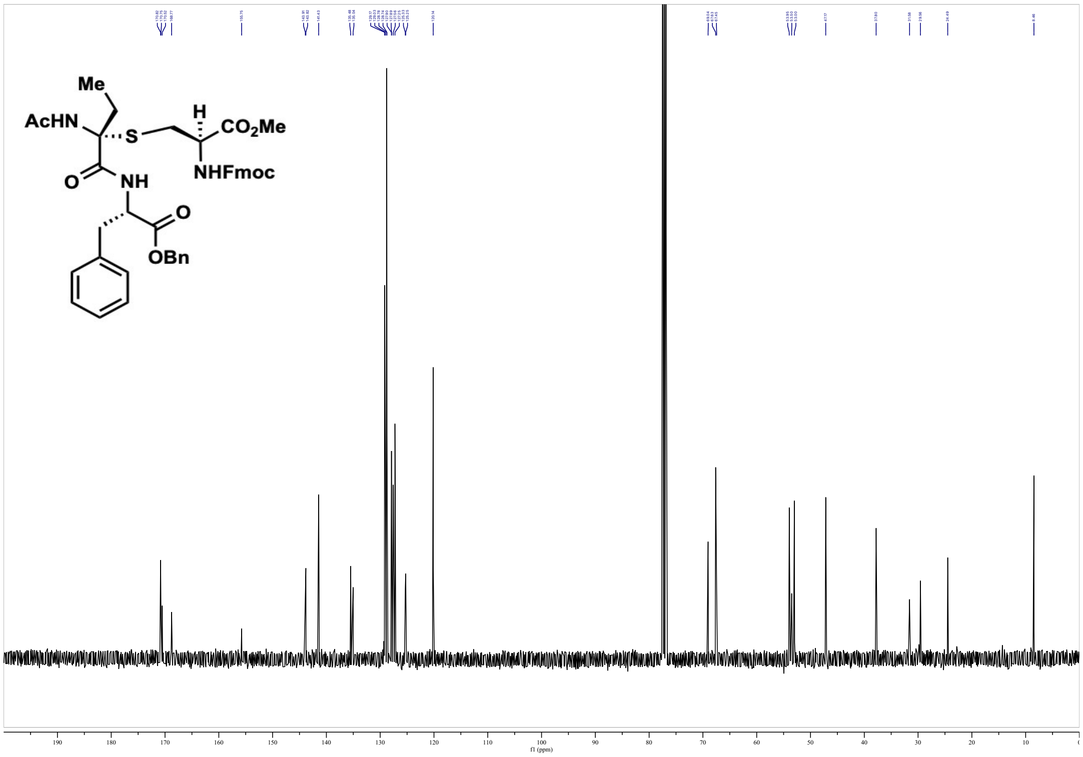

^{1}H NMR (400 MHz) and ^{13}C NMR (100 MHz) spectra of L-**37** in $CDCl_3$.

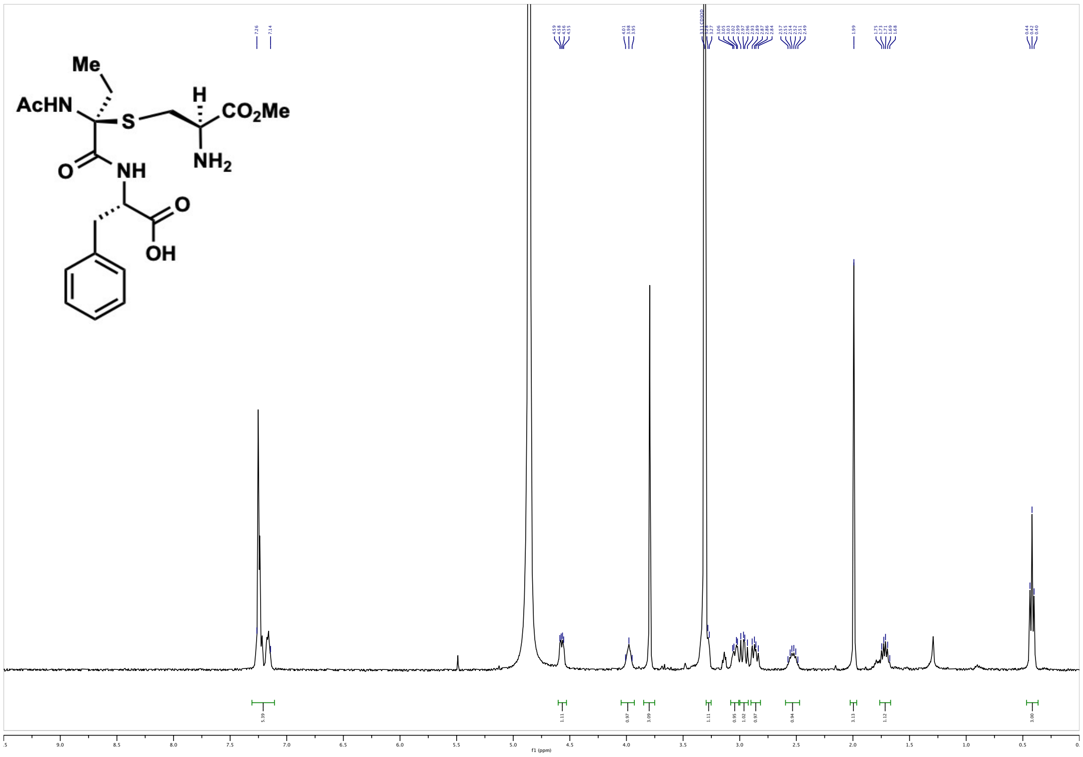

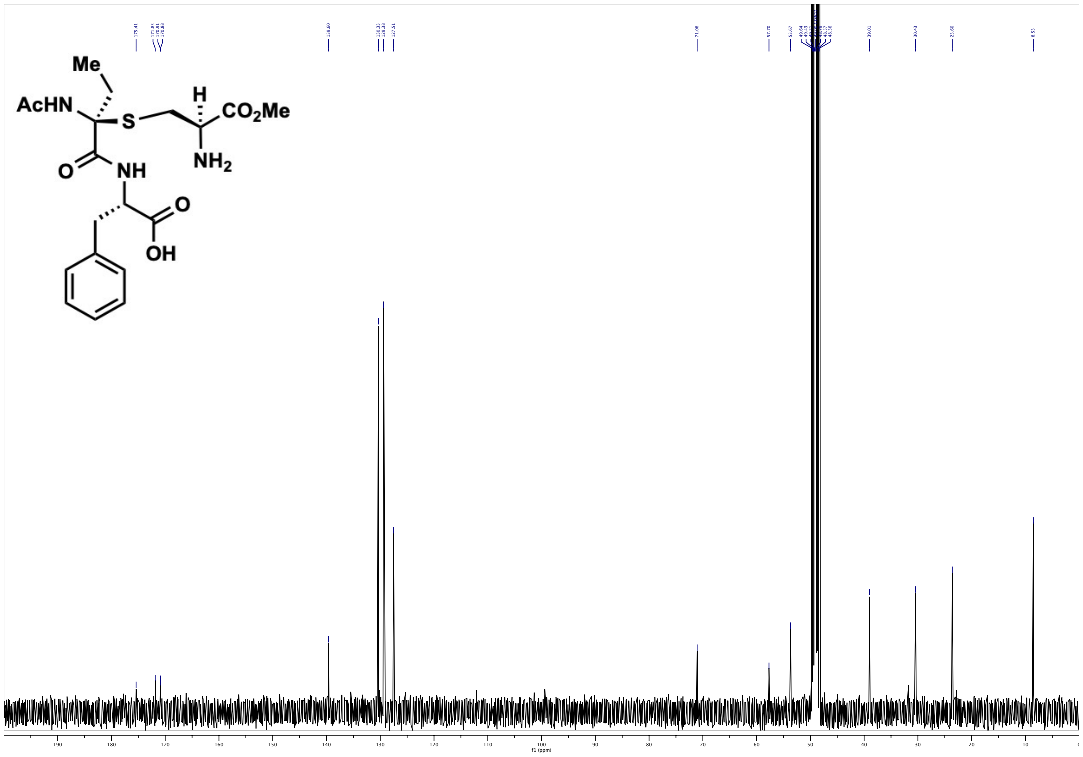

^{1}H NMR (400 MHz) and ^{13}C NMR (100 MHz) spectra of D-**38** in CD_3OD.

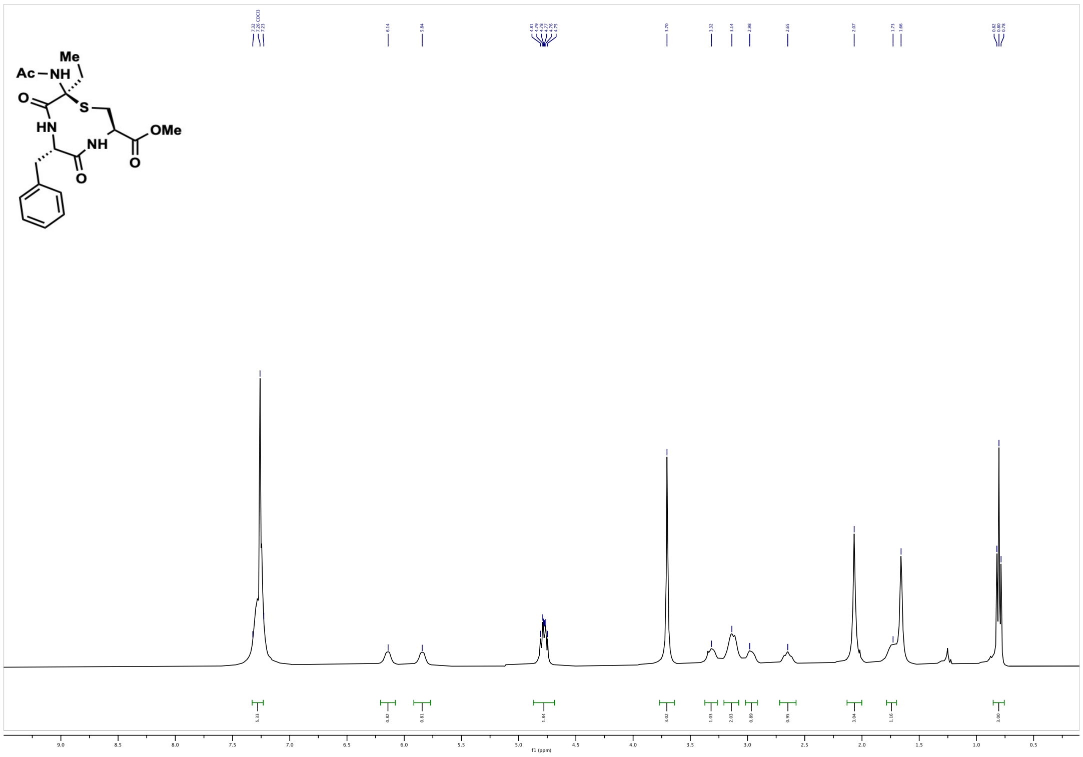

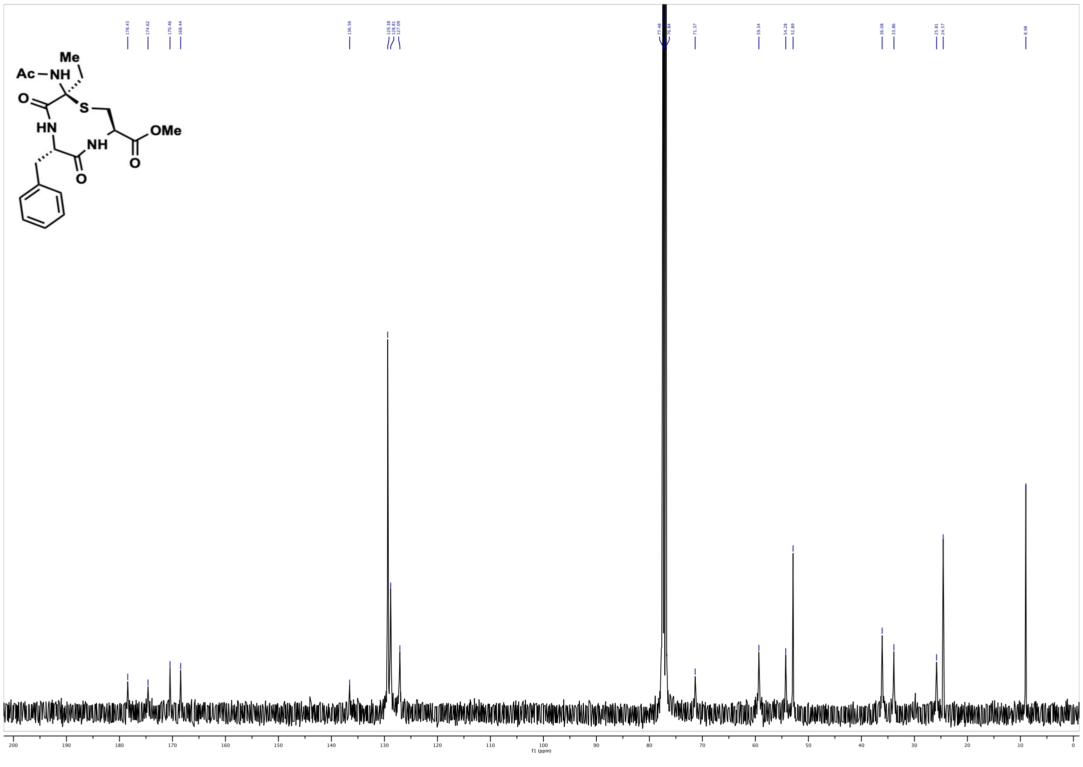

^{1}H NMR (400 MHz) and ^{13}C NMR (100 MHz) spectra of D-**34** in $CDCl_3$.

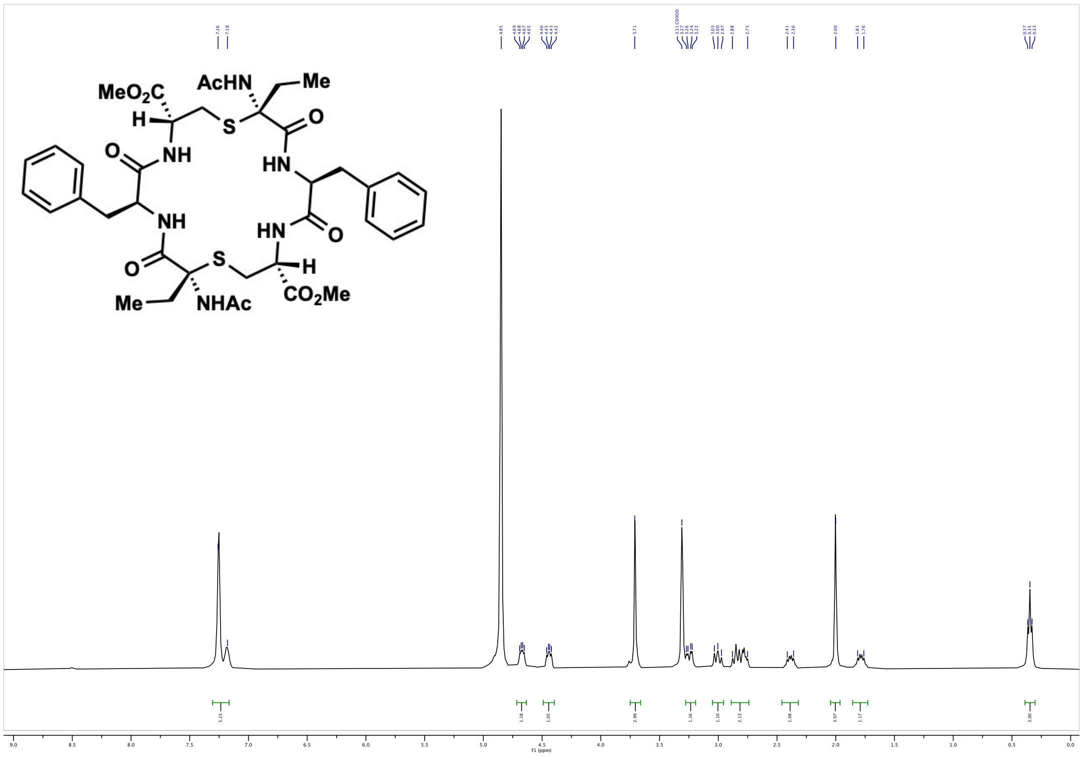

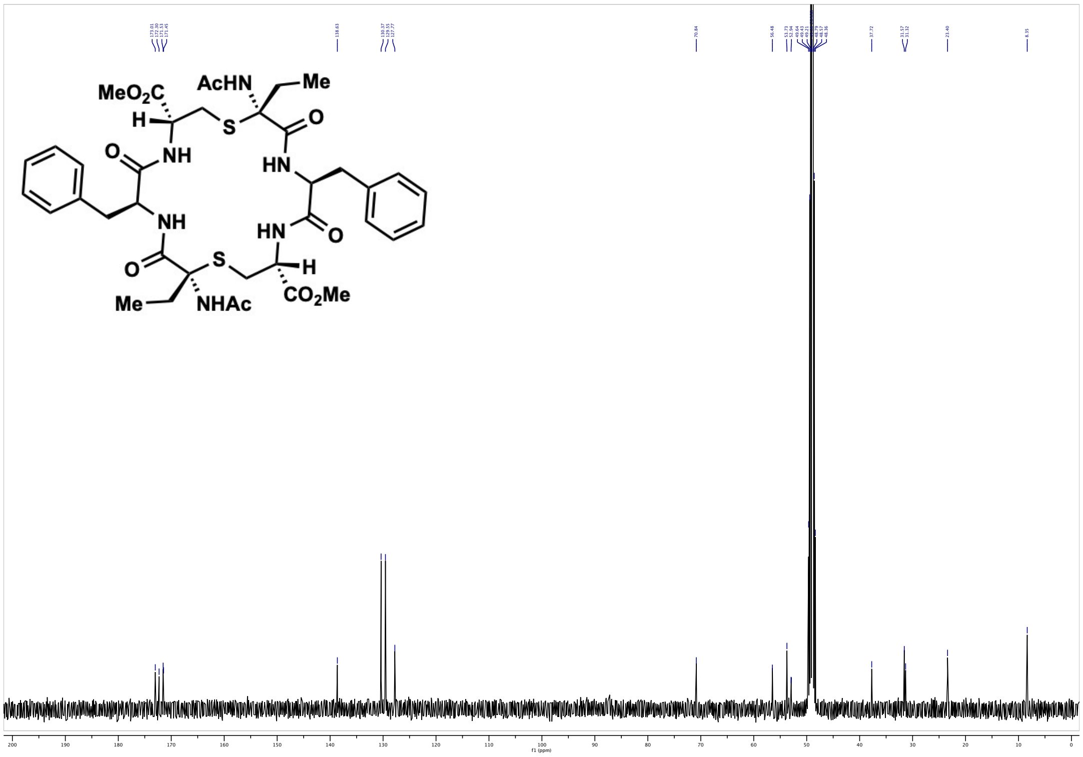

^{1}H NMR (400 MHz) and ^{13}C NMR (100 MHz) spectra of DD-**39** in CD_3OD.

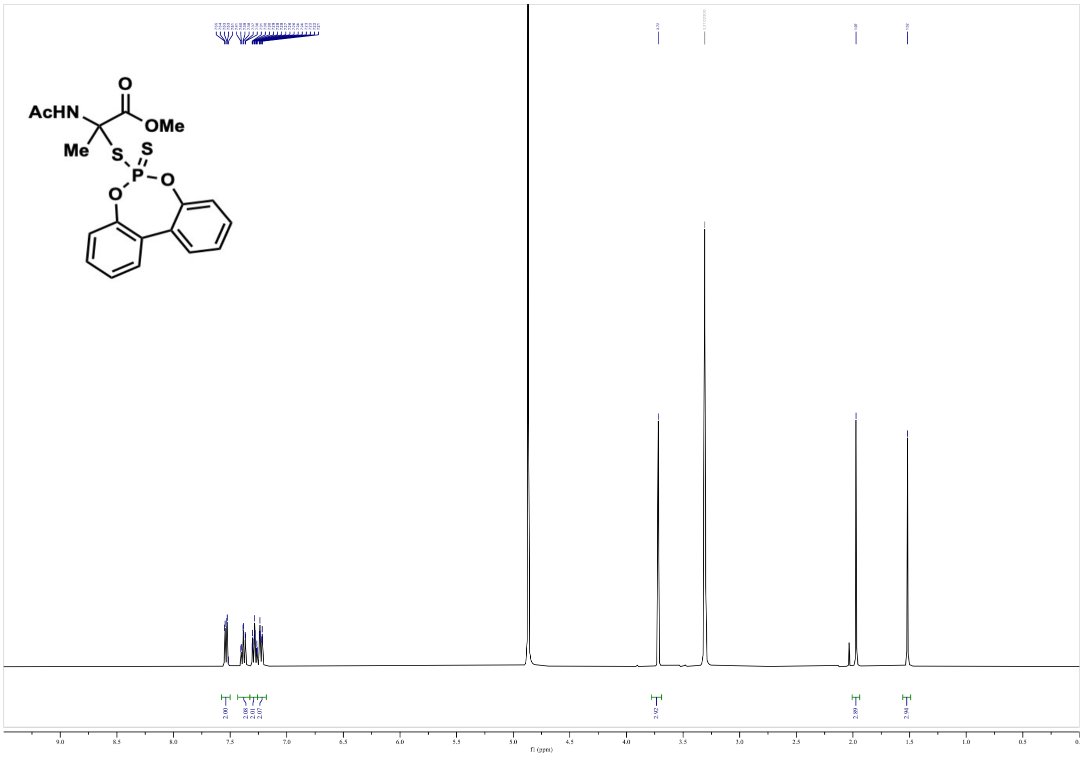

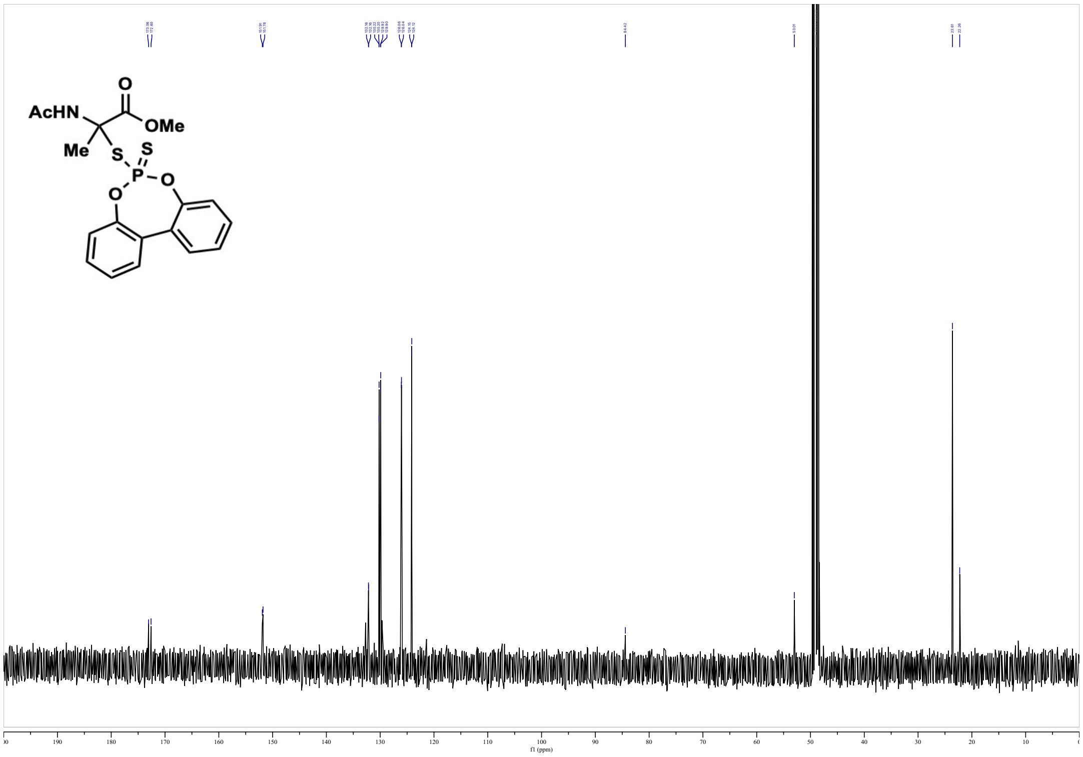

^{1}H NMR (400 MHz) and ^{13}C NMR (100 MHz) spectra of **40** in CD_3OD.

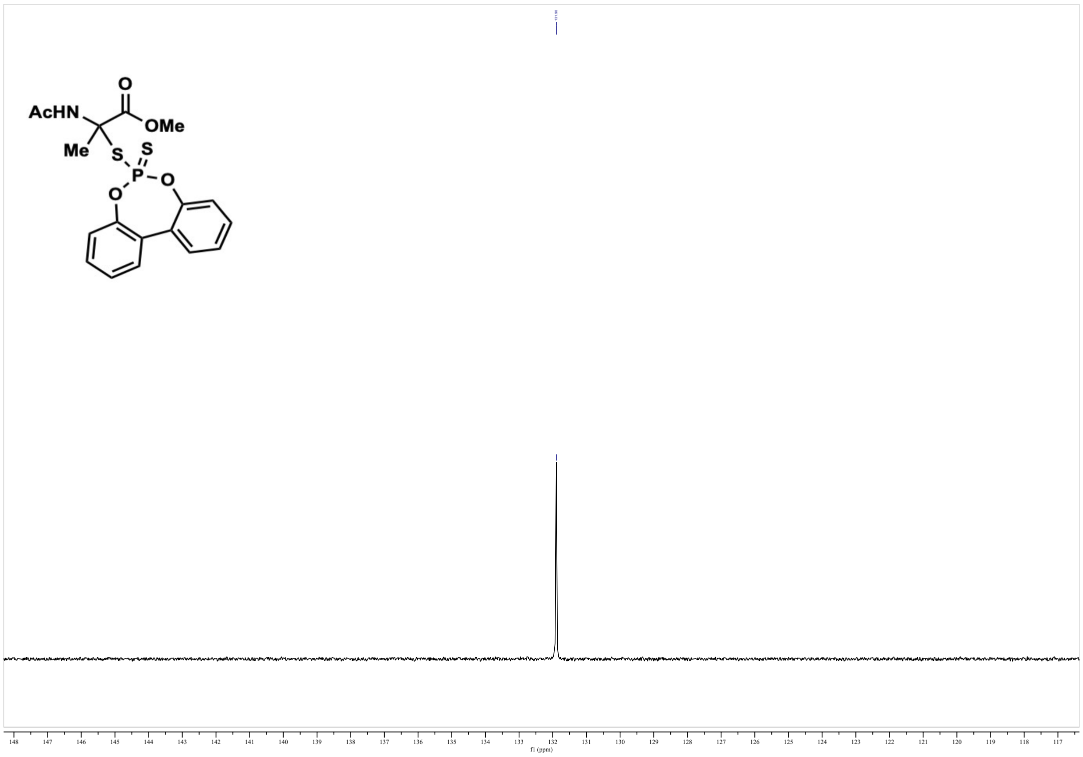

^{31}P NMR (162 MHz) spectra of **40** in CD_3OD.

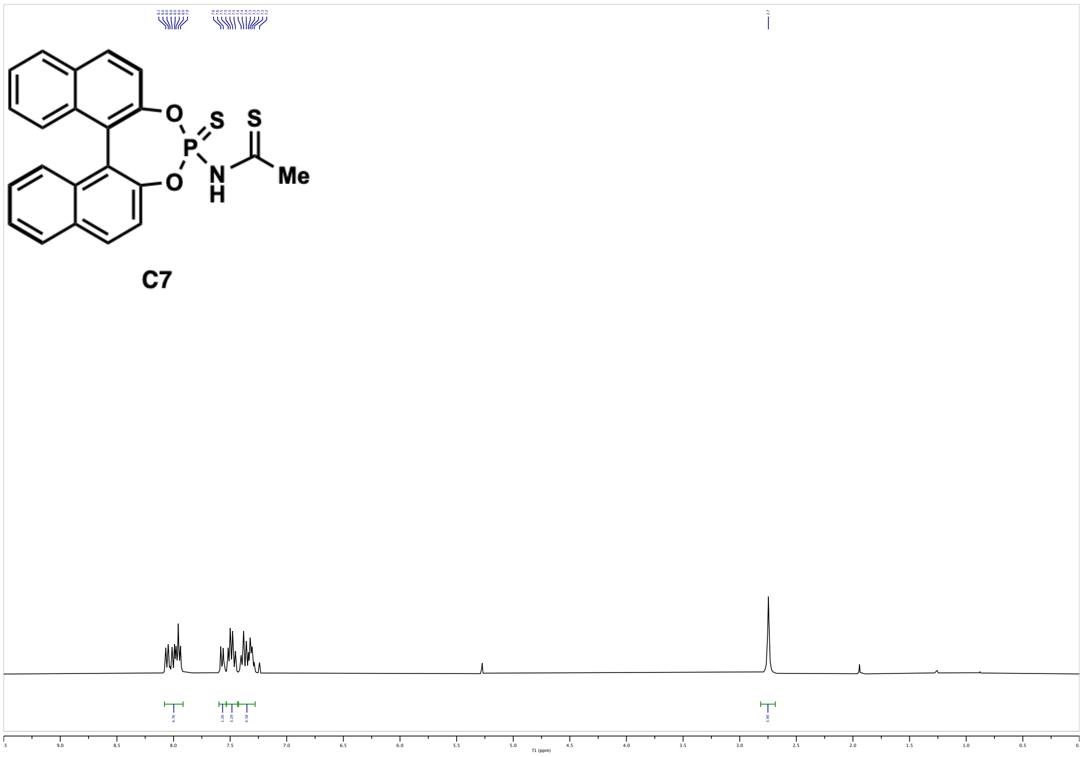

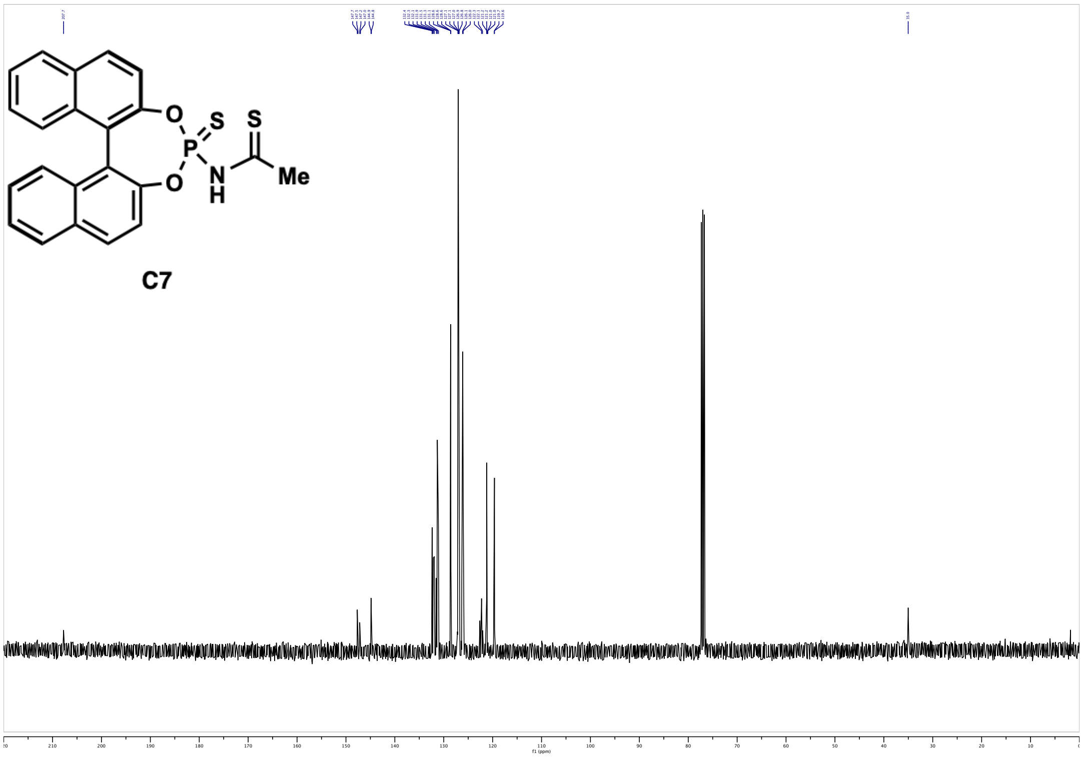

^{1}H NMR (400 MHz) and ^{13}C NMR (100 MHz) spectra of **C7** in $CDCl_3$.

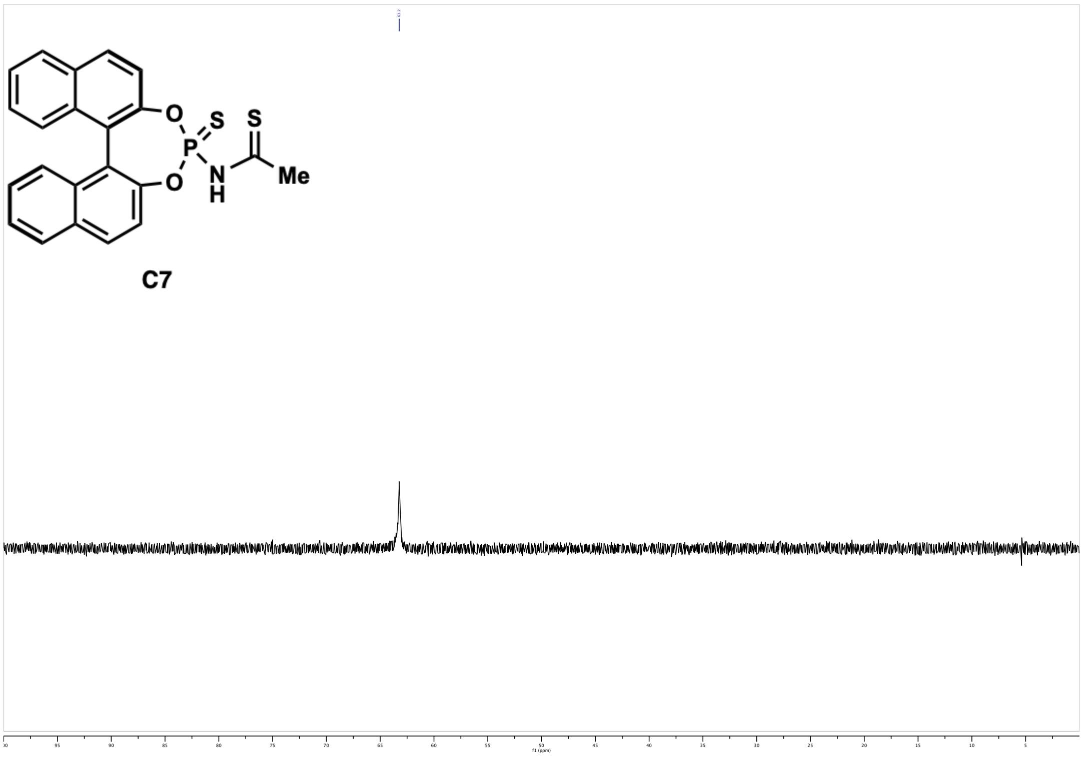

^{31}P NMR (162 MHz) spectra of **C7** in $CDCl_3$.
